# Use of “MGE Enhancers” for Labeling and Selection of Embryonic Stem Cell-Derived Medial Ganglionic Eminence (MGE) Progenitors and Neurons

**DOI:** 10.1371/journal.pone.0061956

**Published:** 2013-05-01

**Authors:** Ying-Jiun J. Chen, Daniel Vogt, Yanling Wang, Axel Visel, Shanni N. Silberberg, Cory R. Nicholas, Teruko Danjo, Joshua L. Pollack, Len A. Pennacchio, Stewart Anderson, Yoshiki Sasai, Scott C. Baraban, Arnold R. Kriegstein, Arturo Alvarez-Buylla, John L. R. Rubenstein

**Affiliations:** 1 Department of Psychiatry and the Nina Ireland Laboratory of Developmental Neurobiology, University of California San Francisco, San Francisco, California, United States of America; 2 Genomics Division, Lawrence Berkeley National Laboratory, Berkeley, California, and Department of Energy Joint Genome Institute, Walnut Creek, California, United States of America; 3Children's Hospital of Philadelphia, University of Pennsylvania, School of Medicine, Philadelphia, Pennsylvania, United States of America; 4 Neurogenesis and Organogenesis Group, RIKEN Center for Developmental Biology, Kobe, Japan; 5 Department of Neurological Surgery, University of California San Francisco, San Francisco, California, United States of America; 6 Eli and Edythe Broad Center of Regeneration Medicine and Stem Cell Research, University of California San Francisco, San Francisco, California, United States of America; 7 Department of Neurosurgery and Eli and Edythe Broad Center for Regeneration Medicine and Stem Cell Research, University of California San Francisco, San Francisco, California, United States of America; 8 Lung Biology Center, Department of Medicine, University of California San Francisco, San Francisco, California, United States of America; Stanford University School of Medicine, United States of America

## Abstract

The medial ganglionic eminence (MGE) is an embryonic forebrain structure that generates the majority of cortical interneurons. MGE transplantation into specific regions of the postnatal central nervous system modifies circuit function and improves deficits in mouse models of epilepsy, Parkinson's disease, pain, and phencyclidine-induced cognitive deficits. Herein, we describe approaches to generate MGE-like progenitor cells from mouse embryonic stem (ES) cells. Using a modified embryoid body method, we provided gene expression evidence that mouse ES-derived Lhx6^+^ cells closely resemble immature interneurons generated from authentic MGE-derived Lhx6^+^ cells. We hypothesized that enhancers that are active in the mouse MGE would be useful tools in detecting when ES cells differentiate into MGE cells. Here we demonstrate the utility of enhancer elements [*422* (*DlxI12b*), *Lhx6*, *692*, *1056*, and *1538*] as tools to mark MGE-like cells in ES cell differentiation experiments. We found that enhancers *DlxI12b*, *692*, and *1538* are active in Lhx6-GFP^+^ cells, while enhancer *1056* is active in Olig2^+^ cells. These data demonstrate unique techniques to follow and purify MGE-like derivatives from ES cells, including GABAergic cortical interneurons and oligodendrocytes, for use in stem cell-based therapeutic assays and treatments.

## Introduction

Cortical interneuron dysfunction may contribute to the risk of developing autism, epilepsy, bipolar disorder, schizophrenia, and dementia [1], [2], [3], [4], [5]. Cortical interneurons are born in the progenitor zones of the medial ganglionic eminence (MGE), the caudal ganglionic eminence (CGE) and preoptic area (POA), and migrate tangentially into the cortex [6], [7], [8] (abbreviations are listed in Table S1 in File S2). Several transcription factors, such as *Dlx1&2, Nkx2-1* and *Lhx6*, regulate interneuron development. For instance, *Dlx1&2* are required for interneuron migration to the cortex [6], [9], [10], [11], [12], [13]. *Dlx1^−/−^* mice are viable, but, due to late-onset interneuron loss, develop cortical dysrhythmias and epilepsy [9]. *Nkx2-1* specifies MGE identity; in *Nkx2-1* null mice the MGE acquires lateral ganglionic eminence (LGE)/CGE identity and lacks MGE-derived interneurons, in part because they fail to express *Lhx6* [14], [15], [16], [17]. In turn, *Lhx6* is required for differentiation of Parvalbumin^+^ and Somatostatin^+^ interneurons [18], [19].

Heterochronic transplantation of rodent embryonic MGE cells into neonatal cortex or adult hippocampus results in their efficient dispersion and integration within host circuits [20], [21], [22], [23]. Furthermore, studies have demonstrated a therapeutic proof of concept that transplantation of MGE cells into rodent models of neuropsychiatric or neurological disorders can suppress seizures, reduce injury induced neuropathic pain, ameliorate phencyclidine-induced cognitive deficits and partially rescue Parkinsonian symptoms [22], [23], [24], [25], [26], [27], [28], [29].

While fetal MGE is a potential source for human transplantation, generating MGE cells from stem cells is advantageous due to limited availability and ethical issues surrounding the use of fetal tissue. Thus, several groups have embarked on generating MGE cells from embryonic stem (ES) cells [30], [31].

Subsequently, mouse and human ES cells lines were generated expressing GFP under the control of loci that mark MGE cells. The J14 mouse ES cell line expresses GFP from an *Lhx6* bacterial artificial chromosome (BAC) transgene and can differentiate into mature Lhx6-GFP^+^ cortical interneurons after transplantation [32]. Human NKX2-1^GFP/w^ ES cells express GFP from the endogenous *NKX2-1* locus and were shown to differentiate into NKX2-1-GFP^+^ basal forebrain progenitors that further differentiated into GABA^+^ and TH^+^ neurons, and PDGFRα^+^ oligodendrocytes [33]. We hypothesize that small enhancer elements active in the mouse MGE will be useful in detecting when ES cells differentiate into MGE cells. Thus, we describe our alternative approach to label specific stages of stem cell differentiation using four small enhancer elements that drive expression in a highly restricted repertoire of cell states related to the MGE and its derivatives.

## Materials and Methods

### Ethics Statement

All animals were treated in accordance with the protocols approved by the Institutional Animal Care and Use committee (IACUC) at University of California, San Francisco (UCSF approval number: AN083918-03A; Approval Date: August 29, 2012; Expiration Date: July 26, 2013). In all the animal experiments, animals of either sex were used.

### ES cells maintenance and differentiation

Mouse Foxg1::venus [30] and E14 embryonic stem (ES) (a kind gift from Jeremy Reiter, UCSF; from Bay Genomics) cells maintenance medium was GMEM medium supplemented with 10% Knock Out Serum Replacement (KSR) (Invitrogen), 1% Fetal Bovine Serum (Hyclone, Define Serum), 1 mM sodium pyruvate (Sigma), 0.1 mM MEM nonessential amino acids (NEAA, Invitrogen), 0.1 mM 2-ME (2-mercaptoethanol, Sigma, freshly prepared each time). For J14 cells [32], maintenance medium was Knockout DMEM (Invitrogen) supplemented with 15% FBS, 2 mM glutamate (UCSF Cell Culture Facility), 0.1 mM NEAA, 1X Pen/Strep (UCSF Cell Culture Facility), 0.1 mM 2-ME. In all ES cells, 2000 U/ml Leukaemic Inhibitory Factor (LIF, Millipore) was added freshly every other day. For feeder cells (SNL and SNLB, see below in the section of **Generation of lentivirus-transduced ES cell clones**) media: DMEM with 10% FBS with glutamate and 1X Pen/Strep. For all ES cell differentiation media: GMEM medium supplemented with 10% KSR, 1 mM sodium pyruvate, 0.1 mM NEAA, 0.1 mM 2-ME; different lots of KSR produced different percentage of Lhx6-GFP^+^ cells (and Foxg1::venus^+^ cells) and therefore required testing for inclusion in the differentiation media. For improved "serum-free embryoid body-like" (SFEBq) culture (see **Results**, modified from the study of [34]), ES cells were dissociated into single cells with 0.25% trypsin-EDTA (Invitrogen) and quickly re-aggregated in the differentiation media containing 100 ng/ml Dickkopf-1(Dkk-1) (5000 cells/100 µl/well) using 96-well low cell adhesion plates (Lipidure-coat plate A-U96, NOF America). On day 3 of differentiation (D3), 20 µl of differentiation media containing SAG (Alexis Biochemicals) was added into each well so that the final concentration of SAG was 6 nM. On D6, ES cell aggregates (embryoid body (EB) aggregates) were transferred to a 10-cm bacterial-grade dish with DMEM/F12 (Invitrogen) supplemented with N2 (Invitrogen) and 6 nM SAG.

### Immunohistochemistry

ES EB aggregates at various time points of differentiation were collected and fixed with 4% paraformaldehyde, then cryoprotected with 15% sucrose overnight before embedding in optimal cutting temperature (O.C.T.; Tissue-Tek, Sakura Finetek) media. Each aggregate was frozen and cryostat sectioned into 30×10 µm sections for immunofluorescent analyses. For antibody staining, glass slides with sections were washed with PBS three times and permeabilized with 0.3% Triton X-100 before blocking with 2% skim milk (Difco). Primary antibodies were, chicken anti-GFP (1∶500, Aves Labs), rabbit anti-Ds-Red (for mCherry staining) (1∶500, Clontech), rat anti-Ds-Red (1∶500, ChromoTeK), mouse anti-Nkx2-1 (1∶200, Leica microsystems), rabbit anti-Nkx2-1 (1∶200, Santa Cruz Biotechnology, Inc.), guinea pig anti-Dlx2 (1∶2000, kindly provided by Kazuaki Yoshikawa, Osaka University, Osaka, Japan) [35], rabbit anti-Foxg1 (1∶2000) [31], mouse anti-Islet1 (1∶250, IOWA Hybridoma Bank), mouse anti-human Ki67 (1∶200, BD Pharmingen), rabbit anti-Tbr1 (1∶1000, Millipore), rabbit anti-Olig2 (1∶500, Millipore), mouse anti-Mash1 (1∶500, BD Pharmingen), rabbit anti-GABA (1∶1000, Sigma), rabbit anti-Calbindin (1∶2000, Swant), rabbit anti-Mafb (1∶1000, Bethyl Laboratories), rabbit anti-PV (1∶2000, Swant), rat anti-Sst (1∶250, Millipore), goat anti-Sst (1∶200, Santa Cruz Biotechnology, Inc.), rabbit anti-NPY (1∶250, ImmunoStar), mouse anti-β-III-Tubulin (TUBIII) (1∶1000, TUJ1, Covance). Alexa 488 and Alexa 594 secondary antibodies (1∶500, Invitrogen) were used according to the primary antibody species. Sections were counterstained with 4′, 6-diamidino-2-phenylindole (DAPI, 5 ng/ml, Invitrogen).

### Image analyses

Immunofluorescent images were taken using a Nikon Eclipse 80i microscope (Nikon), a CoolSnap camera (Photometrics), and NIS Elements BR 3.00 software (Nikon). For marker co-localization, we used image J for cell counting in the red, green and red+green channels. Counting was performed on 200× magnification images. At least three images were counted to obtain mean ± SEM. The degree of differentiation inside each EB aggregate is different; thus we performed our quantification on those EBs with high expression of GFP and Nkx2-1.

For co-localization of various markers with Lhx6-GFP^+^, DlxI12b-βg-mCherry^+^, 692-mCherry^+^ (692-βg-mCherry^+^) and 1056- βg-mCherry^+^ cells we wrote a Macro (set of instructions) in Image J to perform automated cell counting using each color channel (red and green) and the red/green co-localized channel. The threshold was set at 81–255 for the green channel, and 69–255 for the red channel; then the Macro was set to “convert to mask”, “watershed”, and “analyze particle size = 15–200; circularity = 0.20–1.00″ for the individual color and combined color channels. For the co-localized channel the Macro was set to: “colocalization”, "channel1 = red; channel2 = green, ratio = 50, threshold channel 1 = 50, threshold channel 2 = 50, display = 255, co-localized” (see Results section for the results of the automated cell counting).

For co-localization of 692-mCherry^+^, 692-βg-mCherry^+^ cells with Lhx6-GFP^+^, we manually counted cells from images taken from immunofluorescent staining (the data was comparable to that done by Image J analyses but included more in depth analyses). GFP^+^ and mCherry^+^ cells were counted according to its expression level as bright cells or dim cells (there were 3–10 times more of dim mCherry^+^ cells than bright mCherry^+^ cells, whereas there were usually 2–3 times more of bright GFP^+^ cells than dim GFP^+^ cells). The percentage of co-localization in the result sections considered all cells. From one of the clones from each construct (J6M1 and J6βM31) we also calculated the percentage of co-localization among bright GFP^+^ and mCherry^+^ cells. In summary, 92.94%±9.85% of 692-mCherry^+^ cells were Lhx6-GFP^+^; 88.09%±4.7% of 692-βg-mCherry^+^ cells were Lhx6-GFP^+^; among Lhx6-GFP^+^ cells, 35.44%±9.22% were 692-mCherry^+^ and 31.05%±3.59% were 692-βg-mCherry^+^.

For co-localization of 1538-βg-mCherry^+^ cells with Lhx6-GFP^+^, we manually counted cells from 6 images taken from immunofluorescent staining on D14 (see **Results** section).

### Transplantation

On D12 (day 12 of differentiation), ES EB aggregates from 20 96-wells plates were collected (1920 aggregates) and dissociated with the enzyme solution of the Neural Tissue Dissociation Kit (Sumitomo Bakelite, MB-X9901) [34]. Rock inhibitor Y-27632 (10 µM) was added in all the solutions to prevent cell death. Cells were stained with Sytox Blue (Invitrogen, to eliminate dead cells) in 1% BSA/HBSS 15 minutes before sorting to distinguish dead vs. live cells. Lhx6-GFP^+^ cells were sorted with BD FACSAria II using 100 µm nozzle and collected in 10% FBS/DMEM/F-12. Fifty to one hundred thousand sorted ES-Lhx6-GFP^+^ cells were microinjected into the cortex of P0–P2 anesthetized CD-1 mice (iced for 3 min) using pulled capillary glass pipettes. To anesthetize young pups, mice were placed on the ice for 3 minutes. For each experiment, 5–12 animals were injected depending on the cells collected on that day and the pups born in one litter. Cells were transplanted into three sites in each hemisphere at a depth of ∼1 mm from the surface of skull. The pups were then warmed at 37°C on a warm plate before being returned to the dam. After transplantation (4 days, 1 month, or 2 months), the mice were deeply anesthetized (in a CO_2_ chamber) and then perfused transcardially with 4% paraformaldehyde, post-fixed in 4% paraformaldehyde overnight, and cryo-protected in 30% sucrose/PBS overnight before frozen in the optimal cutting temperature (O.C.T.; Tissue-Tek, Sakura Finetek) compound. Fifty µm brain cryo-sections were obtained with cryostat for immunostaining.

### RNA microarray analyses

RNA was isolated from fluorescent activated cell sorting (FACS) purified ES-Lhx6-GFP^+^, ES-Lhx6-GFP^−^, and MGE-Lhx6-GFP^+^ cells using RNeasy Micro kit (QIAGEN) according manufacturer's instructions. The procedures of EB aggregates dissociation, FACS purification and collection of cells were the same as described above for cell transplantation. Embryonic 12.5 (E12.5) MGE from Lhx6-GFP transgenic mouse (in CD-1 background) brains were dissected and dissociated into single cells with 0.05% Trypsin/EDTA (UCSF CCF) with 10 µg/ml DNase I (Roche) at 37°C for 15 min. Purified total RNA was submitted to the Genomic Core at UCSF (http://www.arrays.ucsf.edu), for quality assessment using a RNA Pico Chip on an Agilent 2100 Bioanalyzer (Agilent Technologies). Total RNA was amplified using the Sigma whole transcriptome amplification kits following the manufacturer's protocol (Sigma) and Cy3-CTP labeled with NimbleGen one-color labeling kits (Roche-NimbleGen Inc). Equal amounts of Cy3 labeled targets were hybridized to Agilent whole mouse genome 8×60 K Ink-jet arrays. The data was extracted with Feature Extraction v10.1 software (Agilent).

### Genome coordinates of enhancers

Enhancer *422* is located between *Dlx1* and *Dlx2* genes (human: chr2:172,955,879–172,957,052[hg19]; corresponding to mouse: chr2:71,373,435–71,374,614[mm9]), and encompasses the *Dlx1* and *Dlx2* intragenic enhancer, *DlxI12b*, (mouse: chr2:71,374,047–71,374,552[mm9]) [36], [37]. Enhancer *692* is located on human chromosome 11 (chr11:15,587,041–15,588,314[hg19]) near *Sox6* gene. Enhancer *1056* is on human chromosome 18 (human coordinates: chr18:76,481,720–76,483,257[hg19]) near *Sall3* gene. Enhancer *1538* is on human chromosome 14 (ch14: 36,911,162–36,914,360[hg19]) near *Nkx2-1* gene. The 2.1 kb mouse *Lhx6* enhancer with proximal promoter was described by Du et al., 2008; it extends from the 5' non-coding sequence through the end of intron 1 of *Lhx6* gene.

### Transgenic mouse enhancer assay

Enhancer candidates were amplified by polymerase chain reaction (PCR) from human genomic DNA (Clontech) and cloned into the *Hsp68 promoter-β-galactosidase* (*LacZ*) reporter vector as previously described [38]. Transgenic mouse embryos were generated by pronuclear injection and F0 embryos were collected at E11.5 and stained for β-galactosidase (β-gal) activity with 5-bromo-4-chloro-3-indolyl β-D-galactopyranoside (X-Gal). Since a sufficient number of embryos expressed *LacZ* (had β-gal blue staining in some structures) were obtained, no PCR genotyping was done. We used "blue in any structure" as the transgenic count, and the proportion of embryos with forebrain staining (as assessed from the whole-mount) as a measure of reproducibility. Only patterns that were observed in at least three different embryos resulting from independent transgenic integration events of the same construct were considered reproducible. Here are the numbers for each enhancer. In all cases, the [x/y/z] numbers below indicate E11.5 embryos with staining in that feature/the total number of blue embryos (embryos with blue staining in at least some structures, regardless of the pattern)/the total numbers of embryos that were collected at E11.5. Enhancer *422*: midbrain (mesencephalon) [7/7/38]; forebrain [6/7/38]; nose [6/7/38]. Enhancer *692*: forebrain [9/9/83]. Enhancer *1056*: neural tube [5/8/40]; midbrain [5/8/40]; forebrain [7/8/40]. Enhancer *1538*: forebrain [4/4/34]. In summary, all of the enhancers exhibited greater than 80% of consistent patterns in transgenic mouse enhancer assay. For detailed section analyses, embryos collected at E11.5 were fixed in 4% paraformaldehyde and stained with X-Gal overnight. X-Gal–stained embryos were then embedded in paraffin using standard methods. Coronal sections of the head were cut using standard methods, counterstained with Eosin for visualization of X-Gal -negative embryonic structures and photographed.

### Lentiviral vector generation

The *DlxI12b* DNA fragment was PCR amplified from the DlxI12b-βglobin-Cre vector [37] with introduced 5′ BamHI and 3′ AgeI sites in the primers: (forward: 5′-CTCTGGATCCACACAGCTTAATGATTATC-3′, reverse: 5′-GAGAACCGGTGCAGGAATTCATCGATGATA-3′). The *692*, *1056* and *1538* DNA fragments were PCR amplified from human genomic DNA (Roche) with introduced 5′ BamHI and 3′ AgeI sites in the primers: (692 forward: 5′-ACAAGGATCCCACATCTCAGTGGCTCAT-3′, reverse: 5′-TCTAACCGGTCAGGGTGTCTGTGTTGATG-3′), (1056 forward: 5′-GACAGGATCCGTCCCTCACAGAACTCAG-3′, reverse: 5′-GACAACCGGTGATGCCTGCCTTGAAGTC-3′), (1538 forward: 5′-TCTAGGATCCTGCTGCCTCAAACAAGAATG-3′, reverse: 5′-AGTTACCGGTTTGGATGAGGGAAAGACCTG-3′). Digested DNA fragments of enhancers were cloned into the BamHI and AgeI sites of the *pLenti-mcs-mCherry_Rex1-Blasticidin^r^* vector [39]. The *β-globin* minimal promoter (template: *DlxI12b-β-globin-Cre*) and the *hsp68* minimal promoter [40] were PCR amplified with the following primers: (β-globin forward: 5′-CTATACCGGTAGCCCGGGCTGGGCATAA-3′, reverse: 5′-GAGAACCGGTCGCCGCGCTCTGCTTCTGG-3′), (hsp68 forward: 5′-GAGAACCGGTGCATCGGCGCGCCGACC-3′, reverse: 5′-ATATTCCGGAGGCGCCGCGCTCTGCTTC-3′). See Table S10 in File S2 for full list of primers. The minimal promoters were inserted into the AgeI site that preceded the mCherry gene. The *Dlx-I12b-β-globin* fragment was PCR amplified directly from [37], using the *Dlx-I12b* forward and *β-globin* reverse primers described above. All of the primer sequences are in Table S10 in File S2. All PCR fragments and lentiviral constructs were verified by restriction enzyme digests and DNA sequencing.

### Lentivirus production

HEK293T cells (a gift from Daniel Lim, UCSF; from Thermo Scientific) grown in DMEM with 10% FBS were transfected using Fugene 6 transfection reagent (Roche) with four plasmids to generate lentivirus particles. Plasmids used for a 10 cm tissue culture plate of HEK293T cells (at about 50–70% confluence): 6.4 µg of lentiviral vector DNA and 1.2 µg each of 3 helper plasmids (*pVSV-g*, *pRSVr* and *pMDLg-pRRE*). Media was completely replaced 4 hours after transfection, and cells were grown for four days before harvesting. On day four, all the media was collected and filtered through a 0.45 low protein binding membrane to remove cells and large debris. Filtered media was either aliquoted then stored at −80°C (unconcentrated), or pooled and ultracentrifuged at 100,000× g for 2.5 hours at 4°C. The concentrated viral pellet was resuspended overnight in sterile PBS (adding 50 µl of PBS to the pellet for each 10 cm plate used), then stored at −80°C.

### Generation of lentivirus-transduced ES cell clones

To generate ES cell clones containing lentiviral constructs, proliferating cells (E14 or J14) were dissociated and 400,000 cells were incubated with concentrated virus in a 1.5-ml microcentrifuge tube at 37°C for 1 hour (mixing every 15 min). The cells/virus were then transferred into ES maintenance media containing LIF for an overnight incubation [E14 cells were seeded onto gelatin coated plates without feeders; for J14, cells were seeded onto mitomycin C-treated SNLB feeder cells (see below)]. The next day, the supernatant/virus was removed and fresh media with LIF was supplied for another day before adding blasticidin (20 µg/ml for E14 cells and 4 µg/ml for J14) for 1 week of selection (changing media daily or every other day depending on cell density). Individual colonies emerged ∼1 week after virus infection and were picked up by blunt 10 µl tips, then trypsinized into one well of a 96-well plates. Each clone was expanded and frozen down for further analysis. To establish blasticidin-resistant feeder cells SNLB, an STO cell line (SNL76/7, a kind gift from Louis Reichardt, UCSF; from ATCC) that expresses a *Neomycin resistance* gene and a *LIF* gene, was transfected with *pcDNA6/V5-His ABC* plasmid (Invitrogen, empty vector with *Blasticidin resistance* gene driven by EM7). Mixed colonies of blasticidin-resistant SNLB cells were expanded for frozen aliquots, or treated with mitomycin C for J14 enhancer cell line selection and maintenance.

## Results

### Dissociated MGE cells cultured *in vitro* lose Lhx6-GFP expression

We first attempted to expand MGE progenitors directly from dissociated embryonic mouse MGE tissue. Since previous studies had been successful in expanding neural stem cells in serum-free or serum-containing media with the addition of epidermal growth factor (EGF) and basic fibroblast growth factor (bFGF, or FGF-2) [41], [42], we tested these different protocols for MGE cells. We used MGE cells dissociated from E12.5/E13.5 transgenic embryos that expressed β-Galactosidase (β-Gal) or GFP in postmitotic MGE neurons, including immature cortical interneurons, under the control of a zebrafish *Dlx5/6* enhancer or a mouse *Lhx6-GFP* BAC transgene [43], [44], [45]. Prolonged MGE culture (more than 10 days *in vitro*), or passage of cells that involved trypsinization, resulted in a marked decrease in Nkx2-1 (data not shown) and Lhx6-GFP expression (See Text T1, Methods M1, M2 and Figure S1 in File S1). Neonatal cortical transplantation of MGE-derived cells grown for 21 days in culture resulted in no detectable GFP^+^ cells in the adult cortex. Because we were unable to produce stable pools of Lhx6-GFP^+^ neurons from MGE primary dissociated cultures, we concentrated on using embryonic stem cells to generate Lhx6-GFP^+^ MGE-like neurons.

### Using embryonic stem cells to generate cortical interneuron precursors

Embryonic stem (ES) cells, grown feeder-free in suspension or as adherent culture, can be expanded and differentiated into forebrain progenitors and neurons [31], [32], [33], [46]. The serum-free, floating culture of embryoid body-like aggregates (‘SFEB’) method is an efficient approach for converting ES cells into neural stem cells [31]. In particular, addition of two growth factor inhibitors, the anti-Wnt reagent Dickkopf-1 (Dkk-1) and the anti-Nodal reagent Lefty-A (or SB431542), during the early time points of differentiation efficiently generates Foxg1^+^ telencephalic neural stem cells from ES cells [30], [31]. An improved serum-free embryoid body-like (SFEBq) method using low cell-adhesion U-shape 96-well plates facilitates the aggregation of mouse ES cells after dissociation, generating aggregates of uniform size during differentiation and higher efficiency of production of Foxg1^+^ cells [30]. To convert neural stem cells into ventral telencephalic cells, Sonic hedgehog (Shh) recombinant protein or SAG, a small molecule that binds to Smoothened and activates Shh downstream pathway, was added on days 3 and 6 (D3 and D6) after differentiation [34].

**Figure 1:**
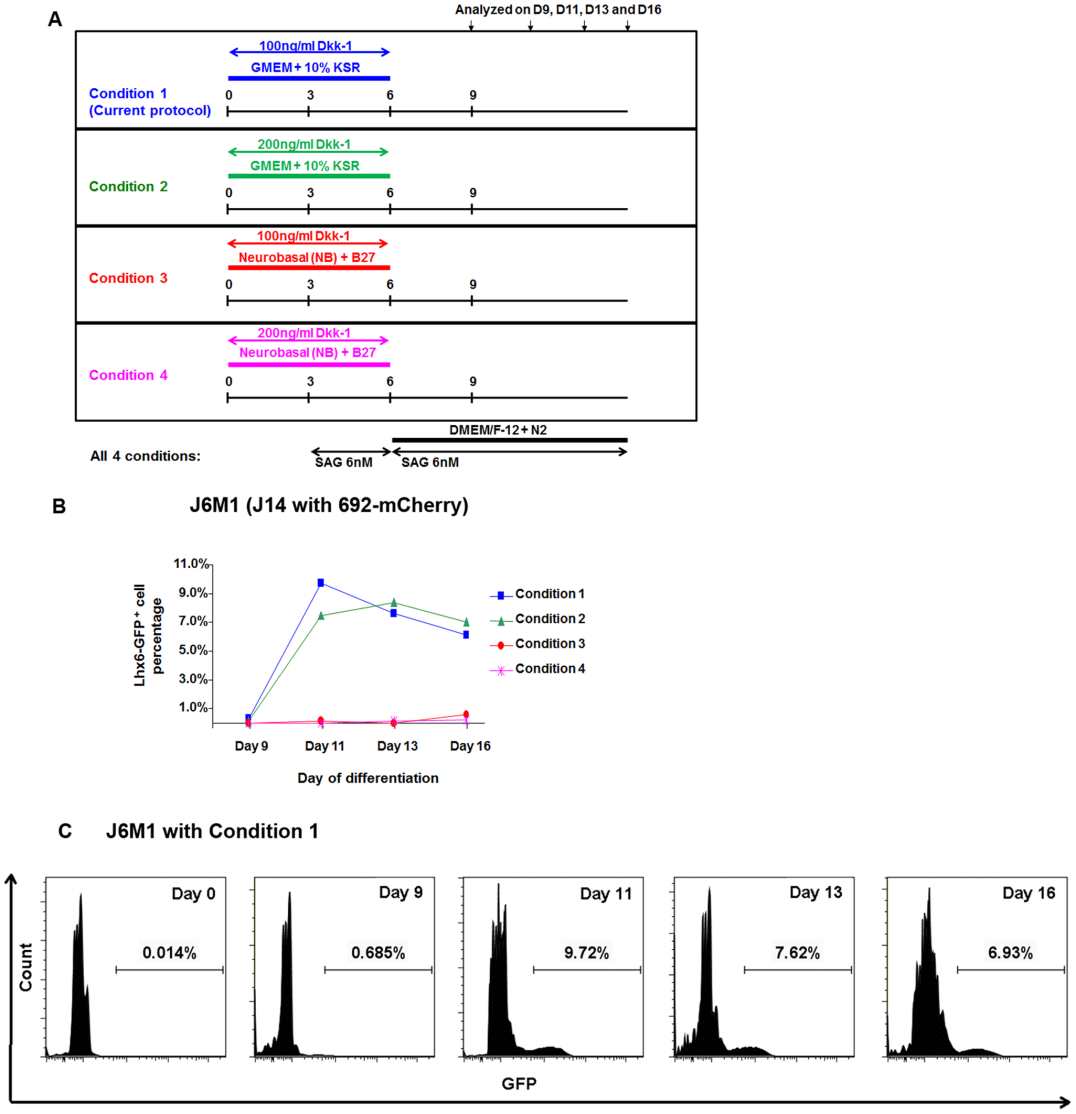
Comparison of various conditions for mouse ES cells differentiation using Lhx6-GFP^+^ cell percentage as a criteria for optimization. J14-derived ES cell line J6M1 (J14 carrying lentiviral enhancer 692-mCherry) were tested for differentiation using four conditions listed. (A): In condition 1 and 2 (shown in blue and green), cells were differentiated in GMEM+10% KSR media while in condition 3 and 4 (shown in red and purple), cells were differentiated in Neurobasal media supplemented with B27 without retinoic acid (NB/B27), a commonly used media for neural progenitor differentiation [69]. Either 100 or 200 ng/ml Dkk-1 was added on day 0 of differentiation (D0), (B): Among all four conditions, KSR-containing media surpassed NB/B27 media in the generation of Lhx6-GFP^+^ cells. Addition of 2× more Dkk-1 on D0 did not improve the efficiency of Lhx6-GFP^+^ cells with KSR-containing media. (C): FACS analyses of Lhx6-GFP^+^ cells with Condition 1. The X-axis showed green fluorescent gating with the log scale.

**Figure 2:**
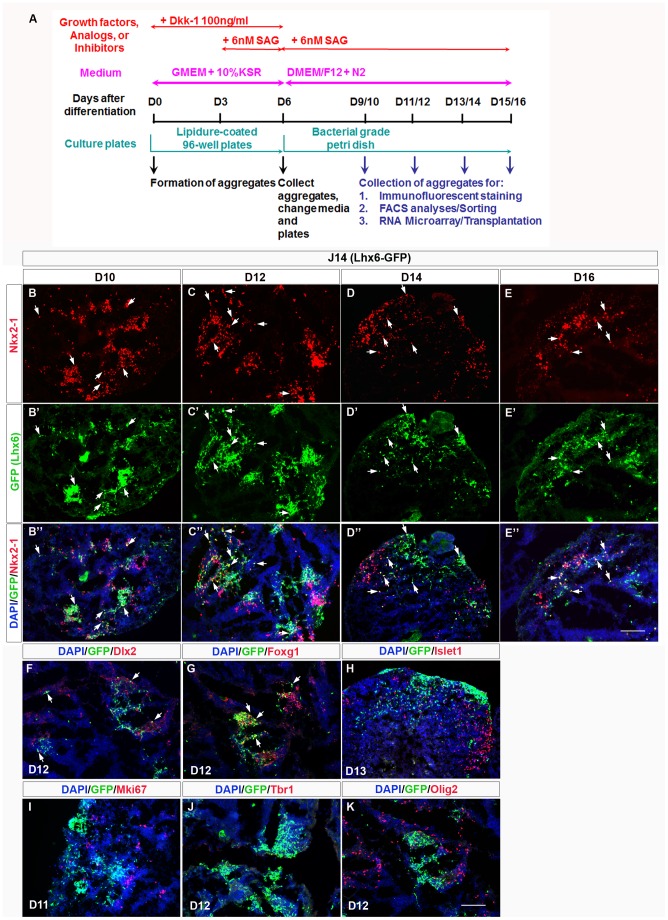
MGE differentiation protocol from mouse ES cells (ES-MGE) and characterization of MGE-like differentiated J14 (Lhx6-GFP) cells. (**A**): Schema outlining the ES-MGE differentiation protocol. The black horizontal line: time line of days after initiation of differentiation. Days when a treatment was introduced are indicated (see Materials and Methods for details). From day 0 (D0) to day 6 (D6), cells were cultured with GMEM and 10% KSR (shown in purple) in a lipidure-coated 96-well plate (shown in cyan). Dkk-1 (100 ng/ml) was added on D0 and SAG (6 nM) was added on D3 shown in red. On D6, cell aggregates were collected and transferred to a bacterial grade sterile petri dish in DMEM/F-12 supplemented with N2. Additional SAG (6 nM) was added to the medium on D6. Starting on D9 (and the following days), aggregates were collected either for immunofluorescent staining, FACS analysis, or FACS purification followed by gene expression microarray analysis, or transplantation. (**B–E″**): Nkx2-1 expression is shown in red; Lhx6-GFP expression is shown in green; DAPI stains the nucleus blue. **B-B″**: D10; **C-C″**: D12; **D-D″**: D14; **E-E″**: D16. White arrows indicate cells co-expressing Nkx2-1 and Lhx6-GFP. (**F**): Dlx2 (red) and Lhx6-GFP (green) expression on D12. White arrows indicate co-localization of Dlx2 and Lhx6-GFP. (**G**): Foxg1 (red) and Lhx6-GFP (green) expression on D12. White arrows indicate co-localization of Foxg1 and Lhx6-GFP. (**H**): Islet1 (red) and Lhx6-GFP (green) expression on D12. (**I**): There were only a few Mki67^+^ (red) cells that expressed Lhx6-GFP (green) on D11. (J): No Tbr1^+^ (red) cells were detected on D12. (**K**): Olig2^+^ (red) cells and Lhx6-GFP^+^ (green) cells were mutually exclusive on D12. Scale bar for all panels: 100 µm.

We used the SFEBq method (Figure 1A and Figure 2A) to generate MGE progenitor-like cells with three mouse ES cell lines: Foxg1::venus [30], E14 (the parental cell line for Foxg1::venus) and J14 (*Lhx6-GFP* transgenic line) [32]. We optimized concentrations of Dkk-1, Shh, SAG, and other growth factors for MGE-like cell production based on Nkx2-1, Lhx6-GFP, and/or Foxg1 expression (Figure 1A and Figure S2 in File S1). We found that a modification of Danjo et al., 2011 [34] (Condition 1 in Figure 1A: using KSR-based media, adding 100 ng/ml Dkk-1 on day 0, and adding 6 nM SAG on day 3 and day 6 of differentiation; now referred to as the ES-MGE differentiation protocol, Figure 1A, Figure 2A and Figure S2 in File S1) was the best procedure for generating Lhx6-GFP^+^ cells from J14 and J14-derived cells (see below for J14 enhancer cell lines) (Figure 1B-C). In addition SAG was more efficient and reproducible than recombinant Shh at generating Nkx2-1^+^ cells (data not shown). The efficiency of the ES-MGE differentiation protocol for induction of Lhx6-GFP expression at D15 (day 15 of differentiation) was ∼2-fold greater than the protocol of Danjo et al., 2011 [34] (Figure S2A-A′ in File S1). Among all the protocol tested, ES-MGE differentiation protocol was most efficient in generating Lhx6-GFP^+^ and Foxg1::venus^+^ cells (Figure S2B-B′ and S2C-C″ in File S1).

On D9 (day 9 of differentiation), the E14 cells expressed markers of MGE and POA ventricular zone (VZ) and subventricular zone (SVZ) progenitors (such as Nkx2-1, Mash1, and Islet 1; Figure S3A and B in File S1); by D15 (day 15 of differentiation), there was a reduction of the number of Nkx2-1 expressing cells (Figure S3A′ and B′ in File S1), suggesting a reduction in MGE and POA-type progenitors. On the other hand, between D9–D15, there was an increase in cells expressing GABA and Calbindin, markers of MGE and POA-type neurons (Figure S3C–D′ in File S1). To obtain better evidence for MGE neuronal differentiation we needed more specific markers for this cell type, and therefore turned to the J14 ES cell line.

MGE progenitor cells give rise to Lhx6^+^ cortical interneurons, striatal interneurons, and globus pallidus neurons [47], [48], [49]. To examine if *Lhx6* was expressed in our ES-MGE differentiation protocol, we studied GFP expression in J14 cells. Using the SFEBq method with our ES-MGE differentiation protocol, we found that Lhx6-GFP^+^ cells began to emerge on D9–10 of differentiation, when there was robust induction of Nkx2-1 expression (Figure 2B-B″). The number of Lhx6-GFP^+^ cells peaked on D12–13 (Figure 2C′) with a slight decline on D15–16 (Figure 2E′). By contrast, the number of Nkx2-1^+^ cells peaked on D9–D10 and gradually decreased from D12 to D16 (Figure 2B–E). We measured the fraction of Nkx2-1^+^ cells that expressed Lhx6-GFP by immunofluorescence. On D10, about 50% of Nkx2-1^+^ cells were Lhx6-GFP^+^ (mean ± SEM: 48.9±3.8%, n = 3), whereas 70% of Lhx6-GFP^+^ cells were Nkx2-1^+^ (72.1±15.0%). On D12, 75% of Nkx2-1^+^ cells were Lhx6-GFP^+^ (75.3±12.9%), and 63% of Lhx6-GFP^+^ cells were Nkx2-1^+^ (62.9±6.0%, n = 3). By D14 and D16, the percentage of Nkx2-1 and Lhx6-GFP co-expression decreased; only 43.3±1.9% and 42.8±5.2% of Nkx2-1^+^ cells were Lhx6-GFP^+^, and 34.7±1.8% and 47.3±13.8% of Lhx6-GFP^+^ cells were Nkx2-1^+^ on D14 and D16 respectively (n = 3). Thus, by using an optimized SFEBq method (our ES-MGE differentiation protocol), J14 and E14 ES cells can be differentiated into MGE-like Nkx2-1^+^ progenitors and Lhx6-GFP^+^ neurons.

### Comparison of RNA expression profiles between ES-Lhx6-GFP^+^ cells and ES-Lhx6-GFP^−^ cells generated from mouse J14 ES cells

**Figure 3:**
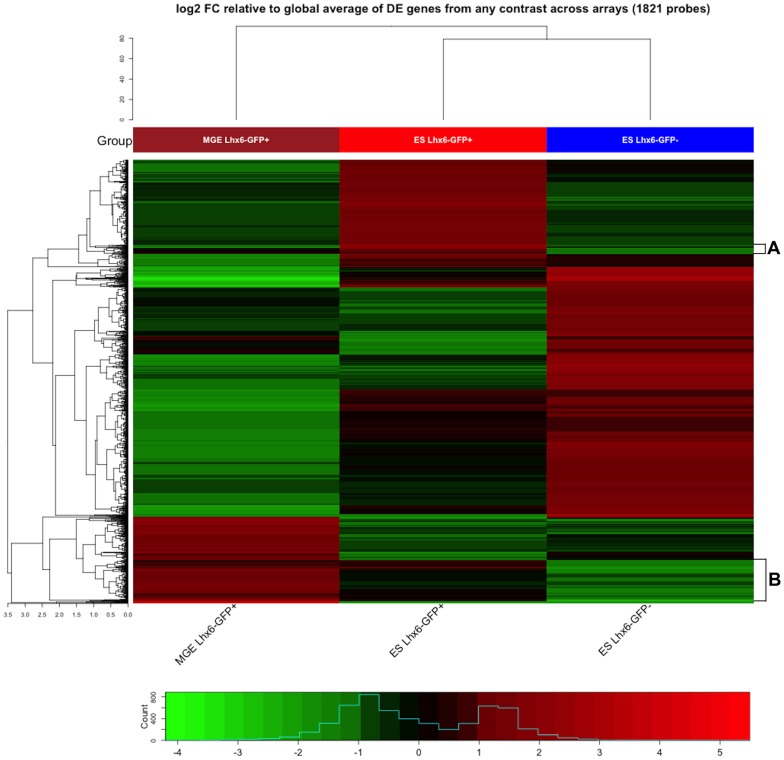
Supervised clustering showing all differentially expressed (DE) genes. Microarray comparison of RNA expression from primary E12.5 MGE Lhx6-GFP^+^ cells, ES-Lhx6-GFP^+^ and ES-Lhx6-GFP^−^ cells. Heatmap includes 1821 probes that exhibit a fold change (FC) of greater than 4 in any one of the possible 3 pairwise comparisons. Heatmap colors correspond to the signal intensity relative to the global average for that probe. Color spectrum ranges from red (5) to black (0) to green (-4): red blocks represent sample-specific expression that is elevated relative to the average across all samples; green blocks represent genes whose transcripts are relatively less abundant. Two areas (A and B, bracketed on the right side) in the supervised heatmap contain many of the genes that regulate and/or mark developing cortical interneurons (see Tables S2 and S3 in File S2).

To further define the molecular properties of the Lhx6-GFP^+^ cells, we used RNA expression array to investigate molecular properties of Lhx6-GFP^+^ (ES-Lhx6-GFP^+^) cells generated from J14 cells at D12 of the ES-MGE differentiation protocol. ES-Lhx6-GFP^+^ cells and ES-Lhx6-GFP^−^ cells (both from D12 EB aggregates) were isolated by fluorescent activated cell sorting (FACS) and were subjected to RNA expression microarray analyses (Figure 3, Tables 1; Table S2 and Table S6 in File S2). Compared to ES-Lhx6-GFP^−^ cells, the ES-Lhx6-GFP^+^ cells had lower expression of neural progenitor markers such as the *HES* genes (*HES5* in Table 1 and *HES1* in Table S2 in File S2), suggesting that the ES-Lhx6-GFP^−^ cells were in a more proliferative state. Consistently, the expression level of the proliferation marker *Mki67* (recognized by the Ki67antibody) was lower in ES-Lhx6-GFP^+^ cells (Table S6 in File S2). Subpallial-specific genes *Dlx1*, *Dlx2*, *Dlx5*, *Dlx6*, *GAD1* (*GAD67*) and *GAD2* (*GAD65*) were present at higher levels in the ES-Lhx6-GFP^+^ cells, consistent with its ventral telencephalic identity (Table 1; Table S2 in File S2). There were also higher mRNA expression of *Nkx2-1*, *Lhx6*, *Lhx8* and *Sox6* (Table 1) in the ES-Lhx6-GFP^+^ cells, consistent with MGE identity. Markers present in migrating immature interneurons such as *ErbB4*, *MafB*, *Npas1*, *Sst* (*Somatostatin*) (Table 1), *NPY* (*Neuropeptide Y*) and *Calb1* (*Calbindin*) (Table S2 in File S2) were also expressed at higher levels in ES-Lhx6-GFP^+^ cells. In contrast, genes expressed in oligodendrocyte precursors and oligodendrocytes, such as *Olig2* and *Sox10*, were expressed at higher levels in the ES-Lhx6-GFP^−^ cells (Table 1; Table S2 in File S2). There was also higher expression of pallial markers (*Pax6*, *Tbr1*, *Tbr2* and *Neurod1*) and LGE (striatal) markers (*Ebf1* and *FoxP1*) in the ES-Lhx6-GFP^−^ cells (Table 1; Table S2 in File S2).

**Table 1:** Select marker genes expression from differentiated ES cells (ES Lhx6-GFP^+^ and ES Lhx6-GFP^−^) and E12.5 MGE cells (MGE Lhx6-GFP^+^) and the comparisons (fold change) of ES Lhx6-GFP^+^ vs. ES Lhx6-GFP^−^, MGE Lhx6-GFP^+^ vs. ES Lhx6-GFP^−^, and MGE Lhx6-GFP^+^ vs. ES Lhx6-GFP^+^.

1	2	3	4	5	6	7
Areas or cells of interest		Expression levels			Comparison between groups (fold changes)	
Genes of interest	ES Lhx6-GFP^+^	ES Lhx6-GFP^−^	MGE Lhx6-GFP^+^	ES-GFP^+^ vs ES-GFP^−^	MGE-GFP^+^ vs ES-GFP^−^	MGE-GFP^+^ vs ES-GFP^+^
**Ventricular Zone**						
*Hes5*	11.62	13.39	11.72	0.29	0.31	1.07
**Oligodendrocytes**						
*Olig2*	8.80	11.63	9.59	0.14	0.24	1.73
**Pallial**						
*Neurog2*	6.46	9.77	6.22	0.10	0.09	0.85
*Pax6*	6.38	7.65	5.97	0.43	0.33	0.75
**Subpallial**						
***Dlx1***	**14.14**	**12.14**	**14.36**	**4.00**	**4.67**	**1.17**
***GAD1***	**13.89**	**11.59**	**13.28**	**4.92**	**3.22**	**0.65**
**LGE/striatum**						
*Ebf1*	8.67	10.35	8.25	0.31	0.23	0.75
**MGE & CGE progenitors**						
***Nkx2-1***	**11.67**	**10.09**	**12.94**	**2.98**	**7.20**	**2.41**
***NR2F1*** ** (dorsal MGE & CGE)**	**13.23**	**12.62**	**12.71**	**1.52**	**1.07**	**0.70**
**MGE subpallial neurons & globus pallidus**						
***Lhx6***	**13.16**	**9.20**	**14.02**	**15.50**	**28.20**	**1.83**
***Lhx8***	**11.49**	**7.55**	**13.13**	**15.31**	**47.56**	**3.11**
***Sox6***	**11.86**	**9.89**	**13.21**	**3.92**	**10.00**	**2.55**
**Globus pallidus**						
*Etv1 (ER81)*	7.04	8.51	11.12	0.43	6.75	17.20
**MGE interneurons**						
***ErbB4***	**10.16**	**8.39**	**10.13**	**3.46**	**3.33**	**1.01**
***MafB***	**11.63**	**9.68**	**11.78**	**3.86**	**4.28**	**1.11**
***Maf (cMaf, vMaf)***	**9.94**	**8.22**	**10.29**	**3.30**	**4.19**	**1.27**
***Npas1***	**10.69**	**7.86**	**8.31**	**7.13**	**1.57**	**0.22**
***Sst***	**14.22**	**11.79**	**13.21**	**5.39**	**2.69**	**0.50**
**Hypothalamus**						
*Nkx2-2*	9.24	10.61	6.68	0.44	0.07	0.19
*Otp*	6.92	7.16	6.84	0.85	0.80	0.95
*Rax*	9.10	7.21	7.05	3.70	0.89	0.24

Column 1 lists marker genes for specific cell types and regions. Note that many of these are not specific for those cells states, but are recognized as useful markers.

The expression levels in the columns 2–4 represent the averaged normalized log2 intensity for each gene. The numbers in columns 5–7 (the fold change) are ratios of the average signal intensity (unlogged) of the two groups in comparison.

Genes that are underlined (shown in red in Table S2 in File S2) are enriched in ES Lhx6-GFP^−^ cells whereas bold-typed genes (shown in green in Table S2 in File S2) are enriched in both MGE Lhx6-GFP^+^ and ES Lhx6-GFP^+^ cells. For most of the genes, the expression in the ES Lhx6-GFP^+^ cells and MGE Lhx6-GFP^+^ cells show similar expression trends, in comparison to ES Lhx6-GFP^−^ cells. However, there are a few genes (shown in regular font here, and in black in Table S2 in File S2) that don't follow this trend.

We also examined hypothalamic and retinal marker expression by microarray analyses. Rax (Rx) expression was higher in the ES-Lhx6-GFP^+^ cells than in the ES-Lhx6-GFP^−^ cells (Table 1), suggesting that some of these cells have either hypothalamic or retinal properties as Rax is essential for early retinal and hypothalamic development [50], [51], [52]. On the other hand, Nkx2-2 expression was lower in the ES-Lhx6-GFP^+^ cells compared to the ES-Lhx6-GFP^−^ cells (Table 1). Nkx2-2 is a marker of the hypothalamus and not the early retina [53], [54], although at mature stages it is expressed in retinal glia [55]. Finally, Otp (a marker of paraventricular nucleus anlage) [50], [56] is expressed near background levels in all three samples (Table 1). Since Lhx6 is expressed in a small domain of the caudoventral hypothalamus (Allen Brain Atlas), it is possible that some of the ES Lhx6-GFP^+^ cells differentiated towards a hypothalamic fate.

To confirm these data, we analyzed protein expression with immunostaining on ES embryoid body (EB) aggregates collected 9–16 days after differentiation (D9–D16) (Fig. 2F–K and Figure S4–S9 in File S1). Consistent with our microarray data, ∼50% of the Lhx6-GFP^+^ cells co-expressed Dlx2 and ∼75% of the Lhx6-GFP^+^ cells co-expressed Foxg1 at D12 (Figure 2F and G), few Lhx6-GFP^+^ cells expressed Islet1 (Figure 2H), and none co-expressed Mki67, Tbr1 or Olig2 (Figure 2I–J) on D11–D13. Thus the RNA expression array and immunostaining result provide strong evidence that Lhx6-GFP^+^ cells from J14 ES cells resemble MGE-derived neurons.

### Comparing RNA expression profiles between Lhx6-GFP^+^ MGE cells and ES-derived Lhx6-GFP^+^ cells

To further investigate how closely ES cells-derived Lhx6-GFP^+^ cells resembled authentic Lhx6^+^ MGE cells, we compared their gene expression profiles. We used fluorescent activated cell sorting (FACS) to purify GFP^+^ cells from the E12.5 MGE of Lhx6-GFP transgenic mice, and from J14 differentiated ES cells at D12 (see above). RNA was isolated from the cells and analyzed by gene expression array. We focused on the expression levels of genes with known regulatory functions and/or expression within the forebrain. We compared expression between the MGE Lhx6-GFP^+^ (MGE-GFP^+^) and J14 Lhx6-GFP^+^ (ES-GFP^+^) cells, and between MGE-GFP^+^ cells and J14 Lhx6-GFP^−^(ES-GFP^−^) cells (Tables 1; Table S2, Table S7 and Table S8 in File S2). There was a remarkable similarity in the properties of the MGE-GFP^+^ and ES-GFP^+^ cells (genes shown in bold typed had higher expression in both MGE-GFP^+^ and ES-GFP^+^ compared to ES-GFP^−^ while genes underlined had the opposite trends in Table 1). MGE-GFP^+^ and ES-GFP^+^ cells both had relatively high expression (>10 arbitrary units) of MGE progenitor markers (*Dlx1*, *Lhx6*, *Lhx8*, *Nkx2-1* and *Sox6*) and markers of immature MGE-derived pallial interneurons (*ErbB4*, *GAD1*, *Lhx6*, *MafB*, *Sox6*, and *Sst*). High levels of *Coup-TFI* (*NR2F1*) suggest that the cells have properties of the dorsal MGE and/or the caudal MGE and CGE.

While MGE-GFP^+^ and ES-GFP^+^ cells shared properties of the MGE and immature cortical interneurons, only the MGE-GFP^+^ cells showed robust expression of globus pallidus markers (Tables 1; Table S2 and Table S7 in File S2), including *Etv1* (*ER81*), *Gbx2*, *Kctd12*, *Lhx8* and *Zic1* [49] [57]. Furthermore, markers of the ventricular zone (*Hes5*), oligodendrocytes (*Olig2* and *Sox10*), pallium (i.e. cortex; *Pax6* and *Neurod1*), LGE/striatum (*Ebf1*) and hypothalamus (*Nkx2-2*) were expressed lower in both MGE-GFP^+^ and ES-GFP^+^ cells than in ES-GFP^−^ (shown in red in Table S2 in File S2). Therefore, *in vitro* D12 differentiated J14-GFP^+^ cells exhibit an expression profile similar to immature MGE-derived interneurons, but do not resemble MGE-derived projection neurons (i.e. globus pallidus) or other MGE-derived cells such as oligodendrocytes.

We generated a supervised heatmap (Figure 3; Table S9 in File S2) and an unsupervised clustering (Figure S12 in File S3) to compare differences in global mRNA levels. The supervised heatmap showed that while the MGE Lhx6-GFP^+^ and ES Lhx6-GFP^+^ cells share commonalities (Table 1; Table S2 and Table S7 in File S2) in their RNA expression profiles, they also have differences. Importantly, both highly expressed genes that are known to be required for the development of MGE-derived cortical interneurons, including *Arx*, *Dlx1*, *Dlx2*, *Dlx5*, *ErbB4*, *Foxg1*, *GAD1*, *Lhx6*, *Lhx8*, *Maf*, *MafB*, *Mef2C*, *Nkx2-1*, *Nrp1*,and *Sox6* (in block A and B in the supervised heatmap in Figure 3; these genes are included in Tables S2 and S3 in File S2). None-the-less, the fact that the clustering of MGE Lhx6-GFP^+^ and ES Lhx6-GFP^+^ cells differed provide evidence that additional work needs to be done to increase the fidelity of the differentiation/purification protocols to obtain MGE-like cells from ES cells. All of the data from this microarray study was submitted to GEO database as **GSE43508** (http://www.ncbi.nlm.nih.gov/geo/query/acc.cgi?acc=GSE43508).

### Lhx6-GFP^+^ cells derived from mouse J14 ES cells become cortical interneurons after transplantation into mouse neonatal cortices

We next studied the properties of these cells *in vivo*. Our analyses indicated that our ES-MGE differentiation protocol generates MGE-type immature interneurons from J14 ES cells. Previous study of J14 ES cells showed that they can become cortical interneurons after transplantation into neonatal brains [32]. We confirmed this by transplanting sorted D12 Lhx6-GFP^+^ cells from our ES-MGE differentiation protocol. Four days after transplantation (4 DAT), about 20% of these Lhx6-GFP^+^ cells expressed markers of migrating cortical interneurons including GABA, Calbindin and MafB (Figure S13A–C in File S3). Thirty to sixty-nine days after transplantation, the Lhx6-GFP^+^ cells had a very low survival rate (∼1%), similar to the previous report [32]. Among Lhx6-GFP^+^ cells that survived at 69 DAT, 22% (mean ± SEM: 22.38±5.01%, n = 4) also expressed Parvalbumin; 58% (57.96±11.50%, n = 3) expressed Somatostatin; and 16% (15.51±6.57%, n = 4) co-expressed Neuropeptide Y (Figure S13D–G in File S3 and Table S4 in File S2). Our results are similar to Maroof et al., 2010 [32]. Therefore, the Lhx6-GFP^+^ cells derived from J14 ES cells have properties of MGE cells based on gene expression profiling (see above) and have properties of cortical interneurons based on transplantation analysis.

### Novel small enhancers that can be used to enrich MGE-derived cells

Multiple small DNA enhancer elements that drive expression in mouse MGE cells have been identified. These include *Dlx1* & *Dlx2* (*Dlx1/2*) intergenic enhancer, *Dlx5* & *Dlx6* (*Dlx5/6*) intergenic enhancer, and *Lhx6* promoter/enhancers [15], [36], [37], [58]. In addition, we have been characterizing novel human telencephalic enhancers, some of which drive expression in MGE cells [59] (http://enhancer.lbl.gov/). Although none of the enhancers is entirely specific to MGE cells, their unique expression patterns may be extremely useful in stem cell studies. Thus, we have explored their utility in identifying cell types using the ES-MGE differentiation protocol in mouse E14 and J14 ES cells. We compared the enhancer activities with markers of MGE cell identity, including expression of Lhx6-GFP.

**Figure 4:**
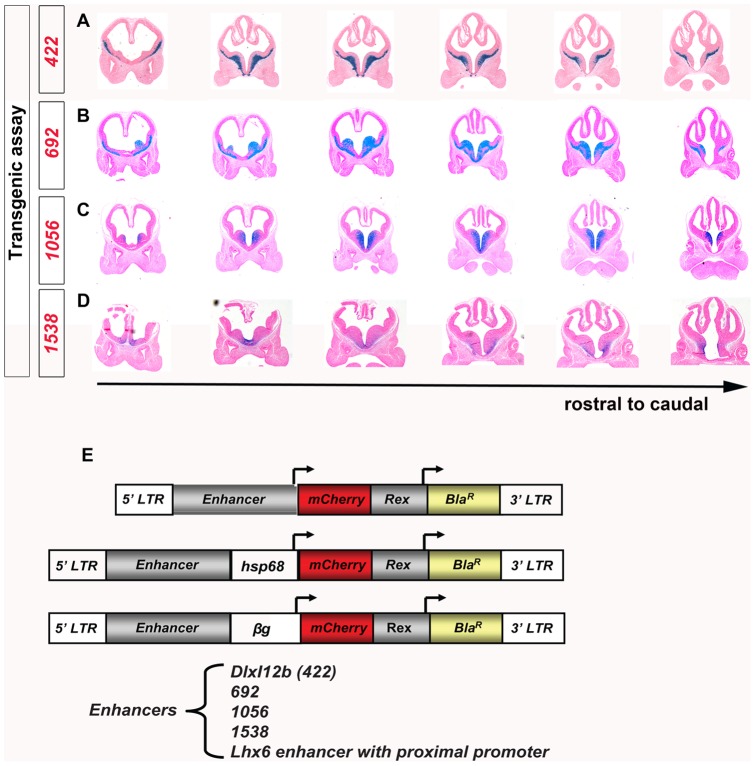
Expression of MGE enhancers in embryonic forebrains, and lentiviral constructs used to transduce them into primary MGE cells and ES cells. (**A–D**): MGE enhancers driving *β-galactosidase* expression (X-Gal staining) of E11.5 telencephalic sections from transient transgenic mice. Coronal sections are shown from rostral to caudal (left to right). Each transgene is composed of one enhancer element *422* (**A**), *692* (**B**), *1056* (**C**), or *1538* (**D**), followed by an *hsp68* minimal promoter that drives expression of *LacZ* (*β-galactosidase*). (**E**): Lentiviral constructs harboring each enhancer reporter cassette for making stable mouse embryonic stem cell clones. Each construct is flanked by a lentiviral 5′LTR and a 3′LTR, and contains two separated gene expression cassettes: the first is the enhancer/promotor driving a *mCherry* reporter gene; the second is *Rex-1* promoter driving the *Blasticidin resistant* gene (*Bla^R^*) [39]. Three lentiviral constructs differed in the first cassette were tested: one without minimal promoter, one with the *heat shock protein 68* (*hsp68*) minimal promoter, and the last one with the *β-globin* (*βg*) minimal promoter. The enhancers tested in this study were: mouse *DlxI12b* enhancer (a shorter version of enhancer *422*), three novel human enhancers (*692*, *1056*, and *1538*), and a mouse *Lhx6* proximal enhancer/promoter DNA element [15].

We focused on five enhancers that show expression in the mouse embryonic MGE (Figure 4, see **Materials and Methods** for genome coordinates and details). Enhancer *422* is located between human *Dlx1* and *Dlx2*, and includes the *Dlx1/2* intergenic enhancer *DlxI12b* that drives expression in forebrain GABAergic neurons, including those derived from the MGE [36]. Similar to the reported mouse enhancer *DlxI12b* activity [37], human enhancer *422* (driving β-Gal expression) was active in MGE subventricular zone (SVZ) and mantle zones (MZ), as well as in the LGE/striatum region of E11.5 transgenic mouse brains (Figure 4A). Enhancer *692* is located on human chromosome 11∼500 Kb away from *Sox6*, a gene that is expressed in the MGE, its derived neurons, and progenitors of cortical projection neurons [60], [61]. Enhancer *692* drove β-Gal expression in VZ, SVZ, and MZ of MGE, as well as in migrating neurons of E11.5 transgenic embryonic brains (Figure 4B). Enhancer *1056* was active only in the ventral part of the E11.5 MGE VZ and SVZ region (Figure 4C). The nearest gene from enhancer *1056* is *Sal-like 3* (*Sall3*), at about 250 Kb away. Enhancer *1538* was active in the VZ, SVZ and MZ of the ventral E11.5 MGE (Figure 4D) and resides in the vicinity of the *Nkx2-1* gene (∼70 Kb away). *Lhx6* enhancer with proximal promoter (*Lhx6 E/P*) sits just 5′ to the *Lhx6* translational start site and presumably contains an *Lhx6* promoter [15]. The *Lhx6 E/P* is active in regions where endogenous *Nkx2-1* is expressed; it responds to exogenous *Nkx2-1* induction in brain slices and its activities were lost in *Nkx2-1*-null brain slices [15].

To determine if these enhancers could be used in labeling mouse ES cells differentiated toward an MGE fate, we utilized a lentiviral vector, α-MHC-mCherry_Rex-Blasticidin^r^, that previously was used to detect and isolate specific populations of differentiated ES cells [39]. As mouse *DlxI12b* enhancer is smaller than the human enhancer *422* (see **Materials and Methods**), and its activities are well documented, we used mouse *DlxI12b* instead of *422* for the lentiviral constructs. Three versions of the lentiviral vector for each enhancer, with different minimal promoters or none at all, were tested (Figure 4E). Enhancer activities were evaluated using lentiviruses carrying three different vectors for *DlxI12b* & *692* in dissociated primary E13.5 MGE cells.

As shown in Figure S14 in File S3, both enhancer *DlxI12b* and enhancer *692* drove mCherry expression in dissociated primary MGE cells in the absence of an introduced minimal promoter (Figure S14A and D in File S3; *DlxI12b-mCherry* and *692-mCherry*). In the presence of the *heat shock promoter 68* minimal promoter (*hsp68*), both *DlxI12b* and *692* produced mCherry^+^ cell clusters; however, these cells had no 4′, 6-diamidino-2-phenylindole (DAPI) nuclear stains, suggesting cell death (Figure S14B and E in File S3). The addition of a *β-globin* minimal promoter (*βg*) resulted in stronger mCherry expression driven by *DlxI12b*, and increased the number of mCherry^+^ cells compared to *DlxI12b-mCherry* (Figure S14C and A in File S3). By contrast, no obvious effect was observed from the addition of the *β-globin* promoter to the enhancer *692* construct (*692-βg-mCherry*, Figure S14F in File S3). We also tested enhancer-less *hsp68-mCherry* and *βg-mCherry* lentiviral constructs in dissociated primary MGE cells. We found that *hsp68* promoter alone drove mCherry expression, whereas *β-globin* promoter did not (data not shown). Thus, the *β-globin* promoter appeared to be more suitable for our experiments.

We also tested these lentiviruses by infecting MGE-like differentiated and dissociated mouse ES cells (infected on D11, and harvested on D14) with the various lentiviral constructs for *DlxI12b* and *692*, and found results similar to dissociated primary MGE cells (data not shown).

Enhancer *1056* with or without a β-globin promoter produced similar numbers of mCherry^+^ cells in dissociated primary MGE cells (data not shown). In contrast, enhancer *1538* without a minimal promoter did not drive mCherry expression in dissociated primary MGE cells (data not shown).

### Enhancer *DlxI12b* drives mCherry expression in ∼30% of Lhx6-GFP^+^ mouse ES-derived MGE-like cells

**Figure 5:**
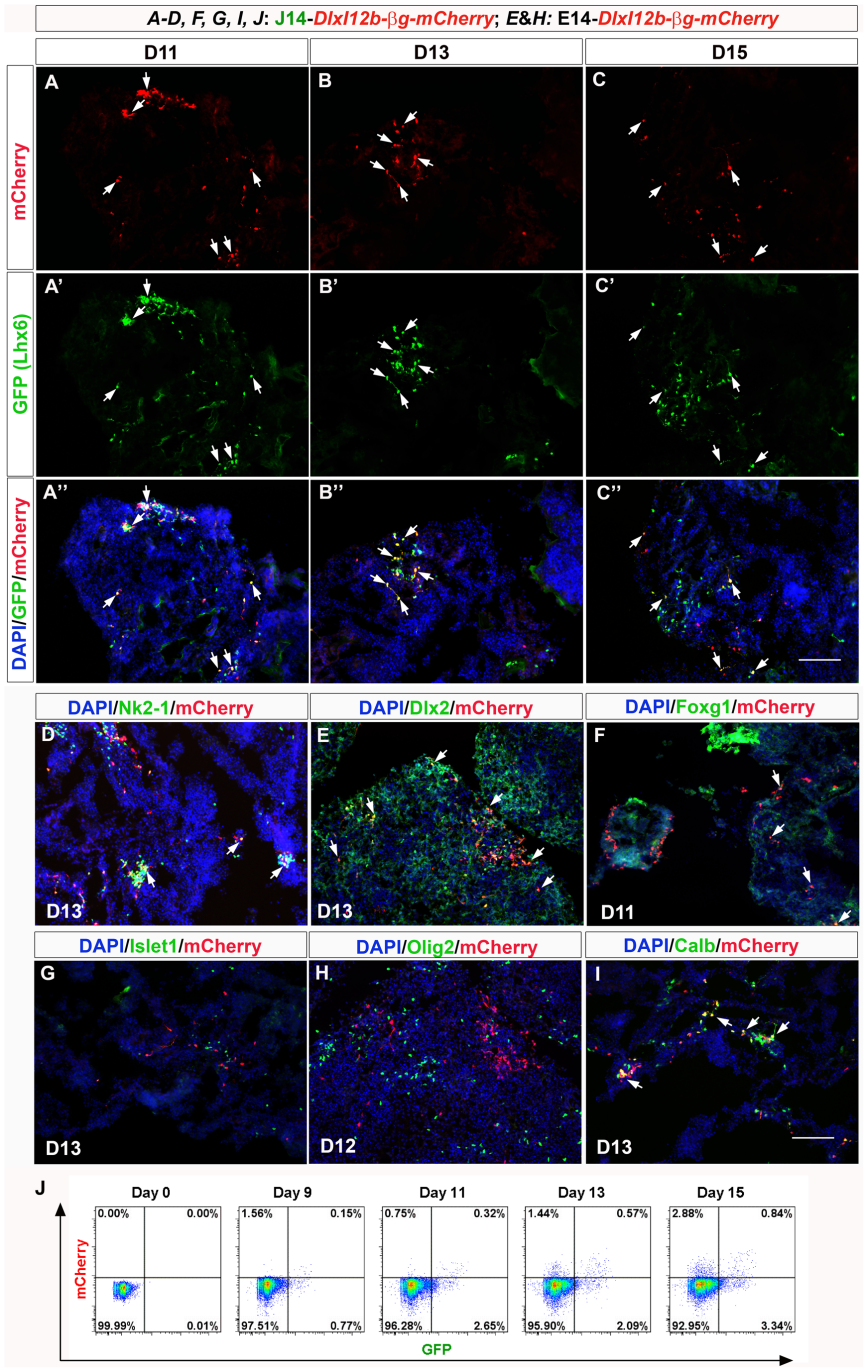
Characterization of *DlxI12b-βglobin-mCherry* in E14 & J14 ES cells differentiated with ES-MGE protocol. Marker expression analyses were done with immunofluorescence of sections from aggregates of differentiated ES cells (ES EBs). (**A–C**): mCherry expression (red) driven by the *DlxI12b-βglobin* enhancer/promoter; Lhx6-GFP expression (green) in panels **A-A″** (D11 EBs), **B-B″** (D13), **C-C″** (D15). (**D**): DlxI12b-βg-mCherry (red) and Nkx2-1 (green) expression on D13 of differentiation. (**E**): DlxI12b-βg-mCherry (red) and Dlx2 (green) expression on D13. (**F**): DlxI12b-βg-mCherry (red) and Foxg1 (green) expression on D11. (**G**): DlxI12b-βg-mCherry (red) and Islet1 (green) expression on D13. (**H**): DlxI12b-βg-mCherry (red) and Olig2 (green) expression on day 12. (**I**): Most of the DlxI12b-βg-mCherry^+^ (red) cells also express Calbindin (green). Scale bar, 100 µm. White arrows indicate markers co-labeling. (**J**) FACS analyses of Lhx6-GFP^+^ cells (on x-axis) and DlxI12b-βg-mCherry^+^ cells (y-axis) from day 0, day 9, day 11, day 13 and day 15 of ES-MGE differentiation.

To explore *DlxI12b* enhancer activities in MGE-like, differentiated mouse ES cells, we generated stable mouse ES clones from both the E14 and J14 (Lhx6-GFP) cell lines with the *DlxI12b-βg-mCherry_Rex-Blasticidin^r^* lentiviral vector (the Foxg1::venus cell line is blasticidin-resistant and cannot be used for this purpose). We analyzed mCherry expression from two independent stable clones from each cell line (EI12bBM7, EI12bBM8; JI12bBM11, JI12bBM12). All four clones produced similar numbers of mCherry^+^ cells in MGE-like differentiated ES cells (using our ES-MGE differentiation protocol). We then analyzed the expression of mCherry along the time course of ES cells differentiation. We started to detect a few DlxI12b-βg-mCherry^+^ cells at D9 (Figure 5J). Their number increased substantially on D11 and D13, but by D15 there was little increase (Figure 5A–C). Double staining of mCherry with Lhx6-GFP revealed frequent mCherry/GFP co-expression on D11, D13 and D15 (Figure 5A″–C″). FACS analyses provided quantification of mCherry/GFP co-expression and individual protein expression (Figure 5J). The percentage of Lhx6-GFP^+^, DlxI12b-βg-mCherry^+^ and GFP^+^/mCherry^+^ cells was low on D9 and increased from D11. From D11–D15, about 22–30% of the DlxI12b-βg-mCherry^+^ cells co-expressed Lhx6-GFP (Figure 5J).

Examining DlxI12b-βg-mCherry expression with markers of telencephalic cell types showed that 49% of the mCherry^+^ cells co-expressed Nkx2-1 on D13, and 55% of the Nkx2-1^+^ cells co-expressed mCherry (Figure 5D and Figure S15A-A″ in File S4). The vast majority of DlxI12b-βg-mCherry^+^ cells co-expressed Dlx2 and Calbindin on D11, D13, and D15 (Figure 5E&I; Figure S15B-B″ and F-F″ in File S4). Some of the DlxI12b-βg-mCherry^+^ cells also express Foxg1, although to a smaller extent (Figure 5F and Figure S15C-C″ in File S4), perhaps because DlxI12b-βg-mCherry expression increased after D9, whereas Foxg1 expression decreased after D9. None of the DlxI12b-βg-mCherry^+^ cells expressed Islet1 or Olig2 (Figure 5G & H; Figure S15D-D″ and E-E″ in File S4), providing evidence that DlxI12b enhancer was active in the MGE-derived cortical interneuron progenitors, rather than LGE (Islet1 is expressed in LGE neurons), or oligodendrocytes (Olig2 is an early marker of oligodendrocytes).

### Enhancer *692* drives mCherry expression in >70% of Lhx6-GFP^+^ mouse ES-derived MGE-like cells

To analyze enhancer *692* activity we attempted to generate stable ES clones from all three lentiviral vectors (*692-mCherry_Rex-Blasticidin^r^*, *692-hsp68-mCherry_Rex-Blasticidin^r^*, and *692- βg-mCherry_Rex-Blasticidin^r^*). With the *692-mCherry_Rex-Blasticidin^r^* lentivirus, 8 out of the 13 E14 clones (from two different screens) and 6 out of the 7 J14 clones analyzed contained mCherry^+^ cells. With the *692-hsp68-mCherry_Rex-Blasticidin^r^* lentivirus, none of the 6 E14 clones and none of the only 2 J14 clones analyzed contained mCherry^+^ cells. With the *692-βg-mCherry_Rex-Blasticidin^r^* lentivirus, 1 out of the 3 E14 clones and 4 out of 8 J14 clones (from two different screens) contained mCherry^+^ cells. The lack of mCherry^+^ cells in *692-hsp68-mCherry* clones may reflect the hsp68-dependent toxicity we identified in transiently infected MGE cells (Figure S14B and F in File S3). Thus, we focused on the 692-mCherry and 692-βg-mCherry clones.

**Figure 6:**
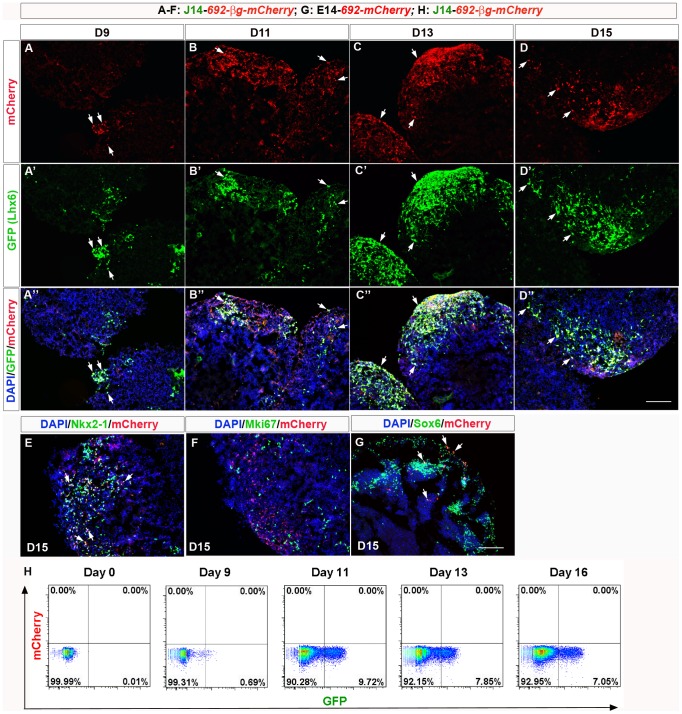
Enhancer *692-βg-mCherry* was active in 70% of Lhx6 GFP^+^ cells. (**A–D″**): mCherry expression (red) driven by *692-βg* and Lhx6-GFP (green) expression on D9 (**A-A″**), D11 (**B-B″**), D13(**C-C″**), and D15 (**D-D″**) ES EB aggregates. On D13 and D15, about 70% of the 692-mCherry^+^ cells were labeled with Lhx6-GFP (white arrows). (**E**): 692-βg-mCherry (red) and Nkx2-1 (green) expression on D15. (**F**): 692-βg-mCherry^+^ (red) cells are postmitotic, as they don't express Mki67 (green) on D15 (and other earlier time points). (**G**): E14 cells line carrying *692-mCherry* was examined with Sox6 expression. All of the 692-mCherry^+^ (red) cells express Sox6 (green). White arrows indicate markers co-labeling. Scale bar, 100 µm. (**H**) FACS analyses of Lhx6-GFP^+^ cells (on x-axis) and 692-mCherry^+^ cells (y-axis) from day 0, day 9, day 11, day 13 and day 16 of ES-MGE differentiation. Though 692-mCherry^+^ (and 692-βg-mCherry^+^) cells was detected with immunostaining and showed extensive co-localization with Lhx6-GFP signals, their endogenous intensity was too low to be detected by FACS (no staining was done with FACS analyses).

We began by studying the time course of mCherry expression. Both 692-mCherry and 692-βg-mCherry expression began in a few cells at D9 in all of the clones examined (Figure 6A; Figure S16A & E in File S4). By D11, a few more 692-βg-mCherry^+^ and 692-mCherry^+^ cells appeared (Figure 6B; Figure S16B, F and I in File S4). By D13, D15, and D17 there were increasing numbers of 692-mCherry^+^ and 692-βg-mCherry^+^ cells (Figure 6C & D; Figure S16C, D, G, H, and J in File S4).

The emergence of 692-mCherry^+^ and 692-βg-mCherry^+^ cells was positively correlated with the increase of Lhx6-GFP^+^ cells. Indeed more than 50% of the Lhx6-GFP^+^ cells co-localized with the 692-mCherry^+^ and 692-βg-mCherry^+^ cells at all the time points examined. This was particularly obvious when the fraction of mCherry^+^ cells reached its highest on D15 and D17 (Figure 6D–D″; Figure S17A–C in File S4). Image analyses on three J14 692-mCherry clones (J6M1, J6M2, and J6M7) on D17 and three J14 692-βg-mCherry clones (J6βM31, J6βM32, J6βM33) on D15 indicated that 692-mCherry and 692-βg-mCherry were present in similar numbers of cells and the percentages of co-localization between Lhx6-GFP and mCherry were comparable (43.28%±6.13% of 692-mCherry^+^ cells were Lhx6-GFP^+^; 51.04%±8.48% of 692-βg-mCherry^+^ cells were Lhx6-GFP^+^; among Lhx6-GFP^+^ cells, 72.87%±5.22% were 692-mCherry^+^ and 70.08%±4.02% were 692- βg-mCherry^+^).

About 30–50% of 692-mCherry^+^ and 692-βg-mCherry^+^ cells co-expressed Nkx2-1 on D15 and D17; among Nkx2-1^+^ cells, 63% were 692-mCherry^+^ or 692-βg-mCherry^+^ (white arrows in Figure 6E; Figure S17A″ in File S4). On the other hand, we did not detect co-expression of mCherry with Mki67 (Figure 6F; Figures S16I & J and S17C″ in File S4), suggesting that *692* enhancer was active only in postmitotic cells. Essentially all 692-mCherry^+^ cells were Sox6^+^ (Figure 6G), an MGE marker as well as a marker for precursors of cortical projection neurons [60], [61]. This is interesting considering that enhancer *692* resides near the *Sox6* gene.

Unfortunately, mCherry expression from enhancer *692* was not robust enough to be seen by mCherry's intrinsic fluorescence (Figure 6H); all of our analyses required immunofluoresence. Thus, we could not use FACS to isolate 692-mCherry^+^ or 692-βg-mCherry^+^ cells.

### Enhancer *1056* drives mCherry expression in Olig2^+^ cells and not Lhx6-GFP^+^ cells

**Figure 7:**
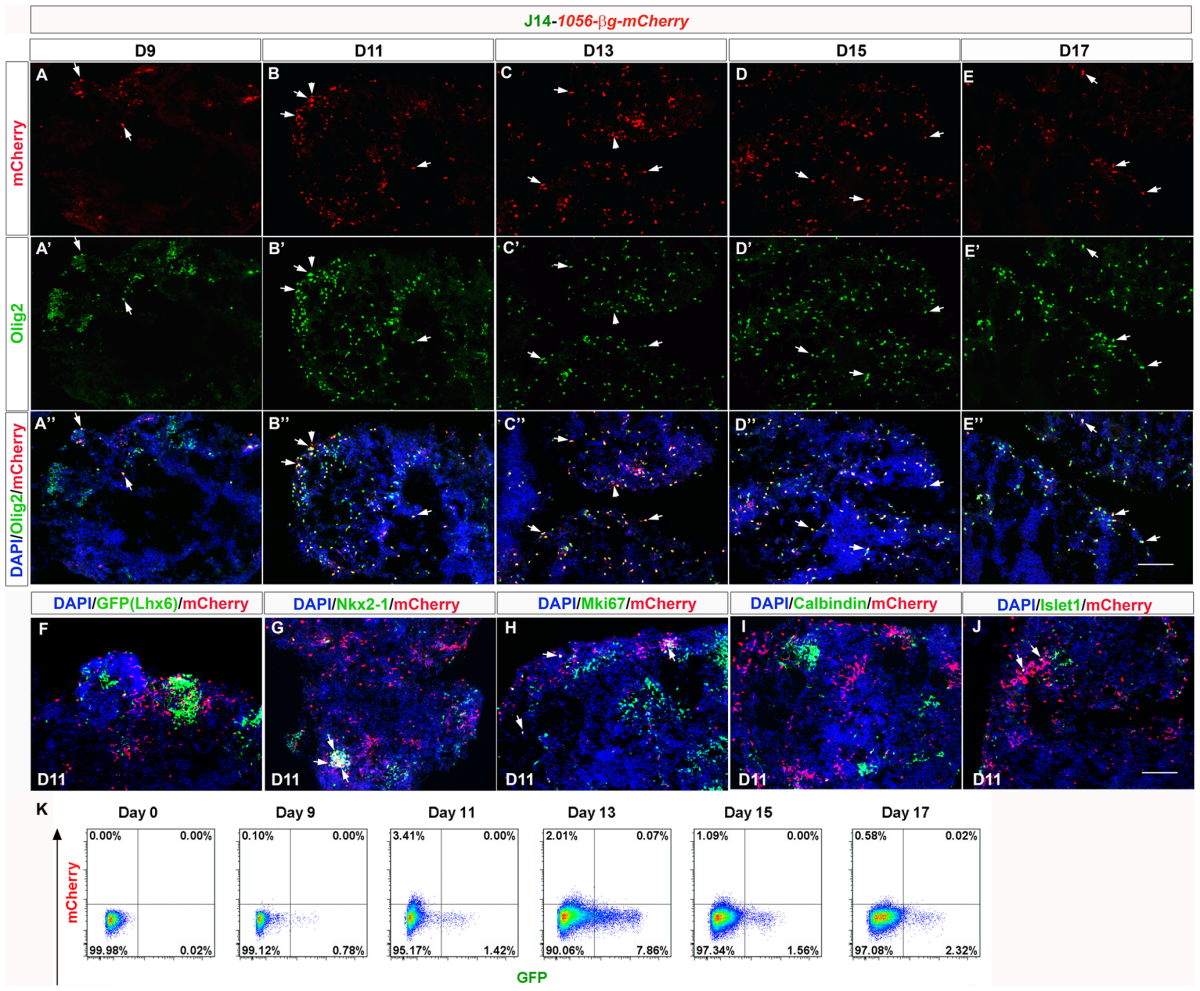
Characterization of *1056-βg-mCherry* in J14 ES cells differentiated with ES-MGE protocol. Enhancer 1056-βg-mCherry^+^ cells are Olig2^+^ and don't express markers of MGE-derived neurons. (**A–E**″): mCherry expression (red) driven by the *1056-βg* and Olig2 (green) expression are shown in panels **A-A″** (D9 aggregates), **B-B″** (D11), **C-C″** (D13), **D-D″** (D15) and **E-E″** (D17). Almost all of the 1056-βg-mCherry^+^ cells express Olig2 (white arrows) on all the time points examined. Only a few 1056-βg-mCherry^+^ cells are Olig2^−^ (white arrowheads in **B-B″** and **C-C″**). (**F**): 1056-βg-mCherry (red) and Lhx6-GFP (green) expression on D11. (**G**):, 1056-βg-mCherry (red) and Nkx2-1 (green) expression on D11. Some of the Nkx2-1^+^ cells are also 1056-βg-mCherry^+^. (**H**): A few 1056-βg-mCherry (red)^+^ cells are still mitotically active, as indicated by Mki67^+^ (green) staining on D11. (**I**): 1056-βg-mCherry (red) and Calbindin (green) expression on D11. (**J**): 1056-βg-mCherry (red) and Islet1 (green) expression on D11. White arrows indicate co-labeling of respective markers shown. Scale bar for all panels, 100 µm. (**K**) FACS analyses of Lhx6-GFP^+^ cells (on x-axis) and 1056-βg-mCherry^+^ cells (y-axis) from day 0, day 9, day 11, day 13, day 15 and day 17 of ES-MGE differentiation. There was no detectable GFP/mCherry double positive cell.

Next we made J14 ES cell clones with *1056-βg-mCherry_Rex-Blasticidin^r^*. From the 4 colonies that we picked and analyzed, just 1 of them expressed mCherry. To our surprise, 1056-βg-mCherry expression did not co-localize with Lhx6-GFP expression (Figure 7F & K; Figure S18A-E in File S4). Nor did 1056-βg-mCherry^+^ cells express Calbindin or GABA at any of the time points examined, despite the fact that there were substantial numbers of mCherry^+^ cells (Figure 7I and data not shown). Nkx2-1 was co-expressed in less than 5% of mCherry^+^ cells (Figure 7G; Figure S18F-J in File S4). Likewise, very few of 1056-βg-mCherry^+^ cells co-expressed Islet1^+^ (Figure 7J).

The MGE generates GABAergic neurons and oligodendrocytes [11], [62]. Thus, we tested whether 1056-βg-mCherry^+^ cells had properties of immature oligodendrocytes, by studying Olig2 expression. As shown in Figure 7A–E″, 65–80% of 1056-βg-mCherry^+^ cells expressed Olig2. Among Olig2^+^ cells, 20% of them were 1056-βg-mCherry^+^ on day 9 and 56%–70% of them were 1056-βg-mCherry^+^ on day 11-day 17. In addition, we found 1056-βg-mCherry/Mki67 double positive cells at all of time points examined (white arrows in Figure 7H and in Figure S18K-O in File S4 on D9–D17). This suggested some of the 1056 enhancer-labeled cells continued to divide at late time points of differentiation.

Interestingly, 1056-βg-mCherry^+^ (as well as Olig2^+^) cells appeared randomly distributed inside the EB aggregates, whereas Lhx6-GFP^+^ cells and Nkx2-1^+^ cells were usually clustered at the outer surface of the EB aggregate. Consistent with 1056's selective activity in Olig2^+^ cells, FACS analyses of the 1056-βg-mCherry J14 clone showed that Lhx6-GFP^+^ cells did not overlap with the 1056-βg-mCherry^+^ cells population from D9–D17 (Figure 7K).

### Enhancer *1538* drives mCherry expression in >40% of Lhx6-GFP^+^ mouse ES-derived MGE-like cells

**Figure 8:**
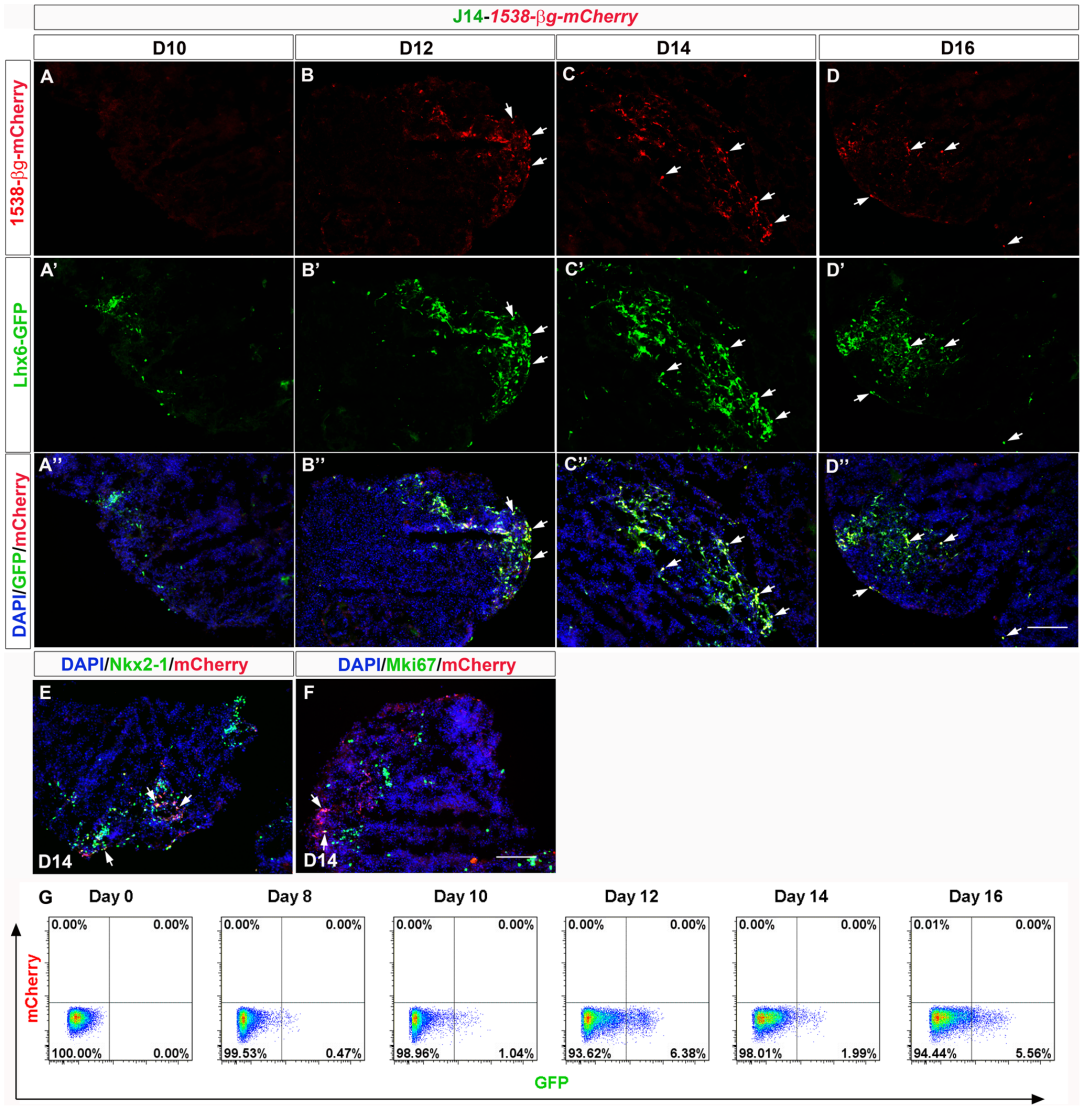
Enhancer 1538-βg-mCherry^+^ labeled 40% of Lhx6-GFP^+^ cells. (**A–D″**): mCherry expression (red) driven by *1538-βg* and Lhx6-GFP (green) expression in panels **A-A″** (D10 aggregates), **B-B″** (D12), **C-C″** (D14), and **D-D″** (D16). On D14, 40% of Lhx6-GFP^+^ cells are 1538-mCherry^+^ and more than 90% of the 1538-βg-mCherry^+^ cells were also labeled with Lhx6-GFP (white arrows). (**E**): 1538-βg-mCherry (red) and Nkx2-1 (green) expression on D14. (**F**): Most of the 1538-βg-mCherry (red)^+^ cells were postmitotic, as they don't express Mki67 (green) on D14 (and other earlier time points). There were a few exceptions (white arrows). Scale bar, 100 µm. (**G**) FACS analyses of Lhx6-GFP^+^ cells (on x-axis) and 1538-βg-mCherry^+^ cells (y-axis) from day 0, day 8, day 10, day 12, day 14 and day 16 of ES-MGE differentiation. Similar to the enhancer *692*, the mCherry expression activity of enhancer *1538* appeared too low to be detected by FACS (no staining was done with FACS analyses).

To test enhancer *1538* activity, we generated J14 stable ES lines with *1538-βg-mCherry_Rex-Blasticidin^r^*. We analyzed 5 clones; 2 of the clones had mCherry expression starting at D12 (Figure 8A & B). There were almost no mCherry^+^ cells on D10 (Figure 8A). On D12–D14, many 1538-βg-mCherry^+^ cells appeared (Figure 8B & C). Though *1538* enhancer resides in close proximity to *Nkx2-1* gene locus, its activity in the differentiating ES cells did not fully correlate with that of Nkx2-1 expression (Figure 8E; Figure S19A–D in File S4): endogenous Nkx2-1 expression appeared early at D9 (Figure S10 in File S1) whereas 1538-driven mCherry expression was not detected at D10, but was found later on D12 (Figure 8A & B). There were more Nkx2-1^+^ cells than 1538-βg-mCherry^+^ cells (Figure 8E; Figure S19A–D in File S4); nevertheless, all of the 1538-βg-mCherry^+^ cells appeared to be Nkx2-1^+^. A few cells with enhancer 1538 activity expressed MKi67^+^, suggesting that they were mitotically active (arrows in Figure 8F; Figure S19E–H in File S4). 1538-driven mCherry expression was highly correlated with Lhx6-GFP expression (Figure 8B″–D″). We analyzed the Lhx6-GFP^+^ and mCherry^+^ cells on D14: 40% (41.18%±4.32%) of Lhx6-GFP^+^ cells were mCherry^+^; 90% (92.26%±3.78%) of mCherry^+^ cells were Lhx6-GFP^+^. *1538* driven mCherry expression was too low for FACS detection (Figure 8G); therefore our analyses required mCherry detection by immunofluoresence.

### No mCherry expression was detected with *Lhx6* enhancer/promoter constructs

We also generated a lentiviral vector with a putative *Lhx6* promoter/enhancer DNA fragment (*Lhx6 E/P-mCherry_Rex-blasticidin^r^*) hoping that it could substitute *Lhx6-GFP* BAC's activities. Unfortunately despite the fact that it was active in dissociated MGE cells (Figure S14G in File S3), we did not see any mCherry^+^ cells from MGE-like differentiated ES cells in any of the 7 stable J14 ES clones infected with this construct.

### The *DlxI12b* enhancer continued to be active in the adult cortex

While our work focused on the activity of the enhancers in MGE-like differentiated ES cells *in vitro*, we explored whether the *DlxI12b* and *692* enhancers maintained their expression *in vivo* following transplantation into neonatal mouse cortex. We used FACS to purify GFP^+^ cells from differentiated (D12) J14 ES cells that also carried either enhancer *DlxI12b* [line: DlxI12b-βg-mCherry (JI12bβM11)] or *692*: [line: 692-mCherry (J6M1)]. As described above, *in vitro* (on D12) 30% of these Lhx6-GFP+ cells are DlxI12b-βg-mCherry^+^ (for JI12bβM11), and 70% of the Lhx6-GFP+ cells are 692-mCherry^+^ (for J6M1).

Analyses of seven transplants from JI12bβM11 [4 animals from 69 DAT, and 3 animals from 33 DAT] found 28.33±2.81% (mean ± SEM, n = 7) of Lhx6-GFP^+^ cells were DlxI12b-βg-mCherry^+^ (Figure S20A–B″ in File S5), showing that the *DlxI12b* enhancer continued to be active in the adult cortex. On the other hand, we had difficulty finding 692-mCherry^+^/Lhx6-GFP^+^ cells in 4 transplants from J6M1 (33 DAT) suggesting that either enhancer 692 is not active, or had low activity, in mature neurons (data not shown). Thus, enhancer *DlxI12b*, but not *692*, is effective for labeling ES cell-derived MGE-derived mature neurons in the adult cortex.

## Discussion

MGE-derived interneuron progenitors have tremendous potential for regenerative medicine [24], [26], [63]. Towards this end, we explored methods to generate and purify these MGE-like interneuron progenitors.

### Generation of MGE-like Cells *in vitro*

Our approach to expand MGE-derived neurons *in vitro* from dissociated primary cells failed. We found that while MGE cells became Dlx2^+^ GABAergic neurons, they lost Lhx6-GFP expression, highlighting the need to identify factors necessary to maintain Lhx6 expression. In contrast, methods to differentiate ES cells into MGE-like progenitors and neurons have been evolving [31], [32], [33], [34]. Using a modified SFEBq protocol (ES-MGE) we improved the efficiency (about 2-fold increase; Fig. S2A′ in File S1) of inducing Lhx6-GFP^+^ cells compared to that of Danjo et al., 2011.

Our ES-MGE differentiation protocol generated progenitors and neurons with MGE-like molecular properties. At D12, cell clusters within the ES aggregates (EBs) expressed markers of immature MGE-derived neurons (Nkx2-1^+^/Lhx6^+^) (Figure 2*B*″). While many cells did not express MGE markers, they lacked detectable expression of pallial markers (Tbr1 and *Pax6*) (Figure 2J; Table S2 in File S2). The Nkx2-1^−^/Lhx6^−^ regions may contain LGE/striatal cells (Islet1^+^ and CTIP2^+^ expression, Figure 2H and data not shown), but neither marker is LGE-specific. Olig2^+^ cells contributed to some of the Nkx2-1^−^/Lhx6-GFP^−^ cells (Figure 2K; Figures S9, and S11 in File S1), and probably correspond to immature oligodendrocytes. Therefore, our ES-MGE differentiation protocol, while relatively specific for MGE specification, is not highly efficient.

The Nkx2-1^+^ MGE-like domains within the EBs appeared around D8–9, similar to previous reports [31], [34]. More than 50% of these Nkx2-1^+^ cells were proliferating at D9 based on Mki67 expression (Figure S10 in File S1). From D10 to D12, there was an increase of Nkx2-1^+^/Lhx6^+^ cells (Figure 2B–C″); this expansion of the “MGE” clusters from D9–D13 suggested that Nkx2-1^+^ cells continued to divide. Later at D14–D16, the EBs expressed makers of immature MGE-derived neurons (Lhx6, GABA and Calbindin; Figure 2D′ and Figure S3 in File S1). Furthermore, transplantation of FAC-sorted Lhx6-GFP^+^ cells generated neurons that expressed markers of mature interneurons (Figure S13D–F″ in File S3) as reported by Maroof et al., 2010.

Comprehensive gene expression analysis showed that partial RNA profiles of ES-derived Lhx6-GFP^+^ cells (at D12 of differentiation) were similar to E12.5 mouse Lhx6-GFP^+^ MGE cells: both types of Lhx6-GFP^+^ cells resembled immature MGE-derived interneurons, and lacked prominent expression of markers of other MGE-derived cells such as oligodendrocytes (Table 1; Table S2 & S3 in File S2).

Since the ES-Lhx6-GFP^+^ cells expressed Nkx2-1 and Lhx8 RNAs (Table 1), they probably correspond to cells that can differentiate into several lineages of MGE-derived neurons, including pallial interneurons, striatal interneurons and the globus pallidus neurons [49], [64], [65]. However, the gene expression array data showed lower expression of markers of globus pallidus neurons (e.g. *ER81*; Table 1; Table S2 in File S2); therefore, we postulate that the ES-Lhx6-GFP^+^ cells are most similar to bi-potential (pallial and striatal) immature interneurons. Furthermore, we suggest that these cells do not differentiate into subpallial cholinergic neurons because they have low expression of Islet1 and *Gbx2* [65], [66], [67] based on immunofluorescence (Figure 2H) and array data (Table S2 in File S2). Finally, we found higher expression of MGE-derived cortical interneuron markers *MafB* and *cMaf* (Table 1; [57]) in the ES-Lhx6-GFP^+^ cells, providing evidence that they are biased towards pallial vs. striatal GABAergic interneurons.

We showed that ES-Lhx6-GFP^+^ cells transplantation into neonatal mouse produced cortical interneurons (Figure S13 in File S3). We did not test striatal transplantation; it is possible that these cells would produce some striatal interneurons, as found for MGE transplantation [28]. Future studies are needed to establish methods to promote pallial interneuron differentiation from these bi-potential progenitors. For instance, we have evidence that Zfhx1b transcription factor participates in the switch between pallial and striatal interneuron identity [57]. *Zfhx1b* was expressed 3-fold higher in MGE-Lhx6-GFP^+^ cells than the ES-Lhx6-GFP^+^ cells (Table S2 in File S2); perhaps increased *Zfhx1b* function would repress *Nkx2-1* and *Lhx8*, and potentiate the differentiation of pallial interneurons.

### Use of “MGE Enhancers” to monitor MGE cell differentiation

The use of molecular markers of specific cell states is a powerful tool for studying cell differentiation. In particular, expression of fluorescent proteins, from specific endogenous gene loci, or from transgenes (e.g. BACs), is an effective method to identify and purify cells. Two published ES cell lines mark MGE differentiation: 1) mouse J14 ES cells that express GFP from an *Lhx6* BAC [32]; 2) human ES cells that express GFP from the *Nkx2-1* locus [33]. Our alternative approach, driving reporter expression using cell/tissue-specific promoters and/or small enhancer elements [39], has several potential advantages: 1) the small size of the enhancers, often less than 1 kb, makes them ideal for insertion into viral vectors; 2) the small enhancers often have a more restricted range of tissue and cell type expression; 3) the approach is ideal for marking multiple cell lines, which would be extremely difficult using BAC transgenic or knock-in strategies; 4) knock-in strategies can alter gene function.

We have identified a large number of enhancer elements in the human genome that drive expression in specific subdivisions of the E11.5 mouse telencephalon, including the MGE [68] (see http://enhancer.lbl.gov/). We explored the function of three of these (novel enhancers *692*, *1056*, and *1538*), in addition to the mouse *DlxI12b* and *Lhx6* promoter/enhancers [15], [36], [37]. We introduced each of these five enhancers into the E14 and J14 mouse ES cells [32] using the vector described by Kita-Matsuo et al., (2009), subjected them to the ES-MGE differentiation protocol, and analyzed mCherry expression in EBs. Four of the enhancers drove mCherry expression in MGE-like cells; only the *Lhx6* enhancer did not work. Enhancer *1056* drove expression in Olig2^+^/Lhx6-GFP^−^ cells (Figure 7). As the MGE also generates oligodendrocytes [11], [62], we predict that enhancer *1056* will be useful for driving expression in oligodendrocyte progenitors. A subset of enhancer *1056* ^+^ cells also expressed Nkx2-1 (Figure 7G); these may be progenitors capable of generating MGE-type neurons, as Olig2 is co-expressed with Nkx2-1 in the ventricular zone of the E11.5 MGE (Figure S21 in File S5; also see Figure S11 in File S1 for differentiated ES data).

Enhancers *DlxI12b*, *692*, and *1538* drove mCherry expression in MGE-like neurons (Nkx2-1^+^/Lhx6-GFP^+^), but not Olig2^+^ cells (Figure 5, 6 & 8). Given that ES-Lhx6-GFP^+^ cells have properties of immature pallial interneurons, based on gene expression array analysis (Table 1 and Table S2 in File S2) and in transplantation assays (Figure S13 in File S3; [32]), we propose that *DlxI12b*, *692*, and *1538* drive expression in cells with properties of MGE-derived interneurons.

*DlxI12b* enhancer was active in both immature and mature pallial interneurons (Figure S20 in File S5), whereas enhancer *692* appeared to be active only in immature MGE cells (data not shown). Furthermore, it will be of interest to follow the fate of enhancer *1056* marked cells (1056-βg-mCherry^+^ cells) following cortical transplantation to determine whether they develop into mature oligodendrocytes [62].

The survival rate of FACS sorted cells followed by cortical transplantation was ∼1% (similar to Maroof et al., 2010[32]). Because we suspect that FACS cell sorting contributes to the low viability, it will be beneficial to pursue other methods for cell purification, including magnetic bead-conjugated antibodies, and the use of enhancer-driven drug selectable markers.

Overall, comparing enhancer activities in transgenic mice and in differentiated mouse ES cells (Table S5 in File S2), only enhancer *DlxI12b* faithfully conveys its activity from *in vivo* to *in vitro*. It is not active in VZ and SVZ (cells from VZ and SVZ are actively dividing) of embryonic MGE in transgenic mice (Figure 4A), nor in mitotically active mKi67^+^ differentiated ES cells (data not shown). It is active in MZ (cells from MZ are mostly postmitotic cells) of embryonic MGE in transgenic mice (Figure 4A) and is active in mKi67^−^ differentiated ES cells (data not shown). In transgenic mice three of the enhancers (*692*, *1056*, *and 1538*) are active in the VZ and SVZ of embryonic MGE (Figure 4B–D); however only two of the enhancers have some activity in mitotically active differentiated ES cells (i.e. *1056* and *1538*) (Figure 7H and 8F). Enhancer *1056* is active in VZ and SVZ of MGE, but it is not active in MZ of MGE in transgenic embryos (Figure 4C). In contrast, about 5% of 1056-mCherry^+^ cells are mitotically active in differentiated ES cells in vitro (Figure S18K-O in File S4). We hypothesize that as 1056-mCherry^+^ cells mature, they turn off Nkx2-1 and differentiate along the oligodendrocyte lineage.

It will be helpful to identify an “MGE enhancer” that more effectively drives expression in dividing cells, to enable selection with a drug-resistance gene. This would greatly facilitate generating large numbers of homogeneous MGE interneuron progenitors for further study and, ultimately, for transplantation in diseased states.

Our approach of using highly specific small enhancers has general utility for generating diverse types of CNS cells. For instance, we have identified enhancers for the LGE and pallium, including its regional subdivisions [68] (see http://enhancer.lbl.gov/) that can be used for selecting these types of progenitors and their derivatives. Introducing these enhancer constructs into ES and iPS cells may facilitate identification and isolation of many different neural cell lineages for basic and translational studies.

## Supporting Information

File S1 **Text T1, Method M1 & M2, Figure S1–S11.** Text T1: Dissociated MGE cells grown *in vitro* lose Lhx6-GFP expression. Method M1: MGE primary culture and antibodies for immunofluorescent studies. Method M2: Transient lentiviral infection in primary MGE and dissociated/differentiated ES cells. Figure S1: Primary MGE cells in vitro differentiate into Dlx2^+^ GABAergic neurons, but lose Lhx6 expression. *A*–*D”*, E13.5 MGE (ventricular and subventricular zone of the MGE) from Dlx5/6-LacZ^+^ embryos was removed from the telencephalon, dissociated and cultured *in vitro* using the media as described (Walton et al., 2006). Ten days after growing in the proliferation media (10 DIV_pro_), the cells were differentiated using differentiation media (DIV_diff_). The state of differentiation was compared during proliferation (*A-A″* and *C-C″*) or after 4 days of differentiation (*B-B″* and *D-D″*) by immunostaining with antibodies to b-Galactosidase (β-Gal), GAD1 (GAD67), Dlx2 and Class III β-Tubulin (Tuj1). Scale bar, 100 µm. *E*–*K*, Lhx6-GFP expression in cells derived from E12.5 MGE (ventricular and subventricular zone of the MGE). The Lhx6-GFP^+^ MGEs were dissociated and cultured *in vitro*. Top row: MGE cells grown in proliferation media for 3 (*E*), 7 (*F*), 10 (*G*) and 13 (*H*) days. Cells were passaged by trypsinization and expansion onto bigger culture dishes on day 7. Bottom row: MGE cells cultured in proliferation media for seven days and then in differentiation media for 0 (*I*), 3 (*J*), and 6 (*K*) days. Images are overlay of DIC images and green fluorescent images. Scale bar, 150 µm. Figure S2: Comparison of various conditions for mouse ES cells differentiation. (A and A′): J14 was subjected to three different conditions for differentiation (the schematic was the same as shown in main figure 2 A) as shown. On D12 and D15, cells were analyzed by FACS analyses to determine the percentage of Lhx6-GFP^+^ cells. Both condition 1 (ES-MGE differentiation protocol, our current protocol, shown in blue) and condition 2 (MGE-type cells protocol, shown in red, (Danjo et al., 2011)) included Shh pathway activators and promoted MGE-like progenitor cells. Condition 3 (cortical cell protocol, in grey, (Eiraku et al., 2008): addition of Dkk-1, without Shh or SAG) generated very few Lhx6-GFP^+^ cells from J14 cell line. (A′): Compared to condition 2 and 3, condition 1 produced the most of Lhx6-GFP^+^ cells on D12 and D15 of differentiation. (B and B′): Four conditions were used to differentiate JI12bBM11 cell line (J14 carrying *DlxI12b-βg-mCherry*). In Condition 1 (shown in blue) 6 nM SAG was applied while in condition 2 (in green) 2 µM purmophamine was used to promote MGE-like progenitor cells. In condition 3 (in purple), 1 µg/ml Dkk-1 was added on D0, in compared to 100 ng/ml Dkk-1 in condition 1 and 2. In condition 4 (shown in red), additional growth factors and small molecule (BMPR1A and SB431542) was added on day 0. (B′): Much more purmophamine is required (2 µM) (Condition 2) to reach the same efficiency generated by SAG (6 nM) (Condition 1). Ten times more Dkk-1 (1 µg/ml) (Condition 3) only produced slightly more Lhx6-GFP^+^ cells on D12 but not on D14. Addition of activin/nodal inhibitor SB431542 and BMP activator BMPR1A on D0 (Condition 4) diminished the effects of 1 µg/ml Dkk-1 on Lhx6-GFP^+^ cells production. (C, C′ and C*″*): Foxg1::venus and JI12bBM11 (J14 carrying lentiviral enhancer DlxI12b-βg-mCherry) were tested for differentiation using four conditions listed (as shown in Figure 1). In condition 1 and 2 (shown in blue and green), cells were differentiated in GMEM+10% KSR media while in condition 3 and 4 (shown in red and purple), cells were differentiated in Neurobasal media supplemented with B27 without retinoic acid (NB/B27), a commonly used media for neural progenitor differentiation (Turksen and Troy, 2006). Either 100 or 200 ng/ml Dkk-1 was added on D0. For all three cell lines tested (including J6M1 in Figure 1), KSR-containing media surpassed NB/B27 media in the generation of Foxg1::venus^+^ cells or Lhx6-GFP^+^ cells. Addition of 2× more Dkk-1 on D0 did not improve the efficiency of Lhx6-GFP^+^ cells or Foxg1::venus^+^ production with KSR media. Figure S3: Characterization of differentiated E14 cells. Expression of Nkx2-1 (red in all panels) with other markers (shown in green): Mash1 (*A, A′*), Islet1 (*B, B*′), GABA (*C, C′*), and Calbindin (*D, D′*), in E14 cell line on D9 and D15 after differentiation. DAPI nucleus staining was shown in blue in all panels. There are more Mash1^+^ cells than Nkx2-1^+^ cells (and some of them express both proteins) on D9. On D15, both protein expressions are reduced with more Nkx2-1^+^ cells than Mash1^+^ cells. Scale bar: 100 µm. Figure S4: Expression of Lhx6-GFP and Dlx2 in J14 cells. J14 cells were differentiated with our ES-MGE protocol. On day 10 (D10; A, A*′*, A″), D12 (B, B′, B″), D14 (C, C′, C″) and D16 (D, D′, D″), cell aggregates were collected for analyses by immunofluorescent staining for Dlx2 (red) and GFP (green). Similar to Lhx6-GFP^+^ cells, the number of Dlx2^+^ cells peaked on D12–14. Arrows indicate co-localization of Dlx2 and Lhx6-GFP. Scale bar, 100 µm. Figure S5: Expression of Lhx6-GFP and Foxg1 in J14 cells. J14 cells were differentiated with our ES-MGE protocol. Expression of Foxg1 (red) and Lhx6-GFP (green) were examined from cell aggregates collected on D10 (A, A′, A″), D12 (B, B′, B″), D14 (C, C′, C″) and D16 (D, D′, D″). The expression of Foxg1 was highest at D10–D12 of differentiation and went down at D14–D16. Scale bar, 100 µm. Figure S6: Expression of Lhx6-GFP and Islet1 in J14 cells. J14 cells were differentiated with our ES-MGE protocol. On D9 (A, A′, A″), D11 (B, B′, B″), D13 (C, C′, C″) and D15 (D, D′, D″), cell aggregates were collected for analyses by immunofluorescent staining: Islet1 (red) and GFP (green). Note the random distribution of Islet^+^ cells within the aggregates compared to the clustered Lhx6-GFP^+^ cells. Scale bar, 100 µm. Figure S7: Expression of Lhx6-GFP and Mki67 in J14 cells. J14 cells were differentiated with our ES-MGE protocol. On D9 (A, A′, A″), D11 (B, B′, B″), D13 (C, C′, C″) and D15 (D, D′, D″), cell aggregates were collected for analyses by immunofluorescent staining: Mki67 (red) and GFP (green). All of the Lhx6-GFP^+^ cells were postmitotic. Scale bar, 100 µm. Figure S8: Expression of Lhx6-GFP and Tbr1 in J14 cells. J14 cells were differentiated with our ES-MGE protocol. On D10 (A, A′, A″), D12 (B, B′, B″), D14 (C, C′, C″) and D16 (D, D′, D″), cell aggregates were collected for analysesby immunofluorescent staining: Tbr1 (red) and GFP (green). There was no Tbr1^+^ cell in any time point examined. Scale bar, 200 µm. Figure S9: Expression of Lhx6-GFP and Olig2 in J14 cells. J14 cells were differentiated with our ES-MGE protocol. On D10 (A, A′, A″), D12 (B, B′, B″), D14 (C, C′, C″) and D16 (D, D′, D″), cell aggregates were collected for analyses by immunofluorescent staining: Olig2 (red) and GFP (green). Similar to Lhx6-GFP^+^ cells, the number of Olig2^+^ cells peaked on D12–14. All of the Lhx6-GFP^+^ cells were Olig2^−^. Scale bar, 100 µm. Figure S10: Expression of Nkx2-1 and Mki67 in J14 cells. J14 cells were differentiated with our ES-MGE protocol. On D9 (A, A′, A″), D11 (B, B′, B″), D13 (C, C′, C″) and D15 (D, D′, D″), cell aggregates were collected for analyses by immunofluorescent staining: Mki67 (red) and Nkx2-1 (green). Plenty of Mki67^+^ cells were present and co-labeled with Nkx2-1 on day 9 and the number of Ki67^+^ cells went down on subsequent time points. There were significantly lower Nkx2-1^+^ that were also Mki67^+^ on D11 and D13. Scale bar, 100 µm. Figure S11: Expression of Nkx2-1 and Olig2 in J14 cells. J14 cells were differentiated with our ES-MGE protocol. On D10 (A, A′, A″), D12 (B, B′, B″), D14 (C, C′, C″) and D16 (D, D′, D″), cell aggregates were collected for analyses by immunofluorescent staining: Nkx2-1(red) and Olig2 (green). About 5% of Olig2^+^ cells co-labeled with Nkx2-1 on D9 and the number of Nkx2-1^+^/Olig2^+^ (double positive) cells decreased on subsequent time points. Scale bar, 100 µm.
(PDF)

File S2 **Table S1–S10.** Table S1: Abbreviated Names. Table S2: Select marker genes expression from differentiated ES cells (ES Lhx6-GFP^+^ and ES Lhx6-GFP^−^) and E12.5 MGE cells (MGE Lhx6-GFP^+^) and the comparisons (fold change) of ES Lhx6-GFP^+^ vs. ES Lhx6-GFP^−^, MGE Lhx6-GFP^+^ vs. ES Lhx6-GFP^−^, and MGE Lhx6-GFP^+^ vs. ES Lhx6-GFP^+^. Column 1 lists marker genes for specific cell types and regions. Note that many of these are not specific for those cells states, but are recognized as useful markers. The expression levels in the columns 2–4 represent the averaged normalized log2 intensity for each gene. The numbers in columns 5–7 (the fold change) are ratios of the average signal intensity (unlogged) of the two groups in comparison. Red colored genes are enriched in ES Lhx6-GFP^−^ cells whereas green colored genes are enriched in both MGE Lhx6-GFP^+^ and ES Lhx6-GFP^+^ cells. For most of the genes, the expression in the ES Lhx6-GFP^+^ cells and MGE Lhx6-GFP^+^ cells show similar expression trends, in comparison to ES Lhx6-GFP^−^ cells. However, there are a few genes (shown in black) that don't follow this trend. Table S3: Block A and Block B from the supervised clustering map (Figure 3). Genes from block A and B of the supervised heatmap (Figure 3) are list below (the order of the genes are the same as in the map (from top to bottom). Many of the genes that regulate and/or mark developing cortical interneurons as shown in Tables 1 and S2 are bold-typed. Table S4: Cell counts from 69 days after transplantation. Four transplants (mice that received Lhx6-GFP+ cells and had at least 10 Lhx6-GFP+ cells in the cortex: N1, R3, R5 and R6) were examined for co-labeling of Lhx6-GFP with parvalbumin (PV), somatostatin (SOM), and neuropeptide Y (NPY). Total numbers, average, and standard errors from 3–4 transplants were shown. Table S5: Comparison of enhancer activities in transgenic embryos and differentiated ES cells. Table S6: Differentially expressed (Fold change are > = 4) genes between ES Lhx6-GFP+ vs ES Lhx6-GFP- cells. Table S7: Differentially expressed (Fold change are > = 4) genes between MGE Lhx6-GFP+ vs ES Lhx6-GFP+ cells. Table S8: Differentially expressed (Fold change are > = 4) genes between MGE Lhx6-GFP+ vs ES Lhx6-GFP- cells. Table S9: Supervised heatmap genes (1821 probes). Table S10: Primer sequences used for lentiviral construct.
(PDF)

File S3 **Figure S12–S14.** Figure S12: Unsupervised clustering showing 1000 most variable probes.Microarray comparison of RNA expression from primary E12.5 MGE Lhx6-GFP^+^ cells, ES-Lhx6-GFP^+^ and ES-Lhx6-GFP^−^ cells. Show here are 1000 most variable probes. Figure S13: Transplanted Lhx6-GFP^+^ cells express cortical interneuron markers in the cortex. (A–C) Four days after transplantation, some of the Lhx6-GFP^+^ cells were also GABA^+^ (A), Calbindin^+^ (B), or MafB^+^ (C). White arrows indicate double positive cells. (D–F”) Sixty-nine days after transplantation, Lhx6-GFP^+^ cells expressed parvalbumin (PV) (D, D′, D″), somatostatin (SST) (E, E′, E″) and neuropeptide Y (NPY) (F, F′, F″). Arrows indicate markers co-labeling. In PV/GFP co-staining, there were some GFP^+^ cells that have weak PV expression (white arrowheads). Scale bar, 100 µm. (G) Average (data are mean ± SEM) percentages of parvalbumin^+^ (PV^+^/GFP^+^), somatostain^+^ (SST^+^/GFP^+^), neuropeptide Y^+^ (NPY^+^/GFP^+^) cells among all Lhx6-GFP^+^ cells (n = 3–4). Figure S14: Test of lentiviral constructs in dissociated primary MGE cells. Dissociated primary MGE cells (E13.5) were infected with each of the lentiviruses indicated (A: *DlxI12b-mCherry*, B: *DlxI12b-hsp68-mCherry*, C: *DlxI12b-βg-mCherry*, D:*692-mCherry*, E: *692-hsp68-mCherry*, F:*692-βg-mCherry*, *G*: *Lhx6-E/P-mCherry*) for three days before being fixed for immunostaining. Pictures are composites from several different fields (A–F) or from one single field (G). Shown here are Nkx2-1 staining in green, mCherry in red, and DAPI nuclear stain in blue. Scale bar, 50 µm.
(PDF)

File S4 **Figure S15–S19.** Figure S15: Additional characterization of the enhancer *DlxI12b*. Mouse ES cell lines E14 (B-B″ & E-E″) and J14 (A-A″, C-D″ & F-F″) carrying enhancer *DlxI12b-βg-mCherry* were differentiated with our ES-MGE protocol. Expression of DlxI12b-βg-mCherry (red) was examined on D11, D13, and D15 together with other markers (shown in green): (A) Nkx2-1, (B) Dlx2, (C) Foxg1, (D) Islet1, (E) Olig2, (F) Calbindin. Scale bar, 200 µm. Figure S16: Additional characterization of the enhancer *692*. (A–D) Mouse ES cell lines J14 carrying enhancer *692-βg-mCherry* were differentiated with our ES-MGE protocol. Expression of 692-βg-mCherry (red) was examined together with Nkx2-1 (shown in green) on D9, D11, D13, and D15. Scale bar, 200 µm. (E–J) Mouse ES cell lines E14 carrying *692-mCherry* were differentiated with our current MGE protocol. Expression of 692-mCherry (red) was examined with Nkx2-1 (E–H) and Mki67 (I, J) (shown in green) on days indicated. Scale bar, 100 µm. White arrows indicate co-labeling of respective markers shown. Figure S17: Additional characterization of the enhancer *692*. Mouse ES cell lines J14 carrying enhancer *692-mCherry* were differentiated with our ES-MGE protocol. Expression of 692-mCherry (red) was examined on D17 together with other markers (shown in green): (A) Nkx2-1, (B) Lhx6-GFP, (C) Mki67. White arrows indicate co-labeling of respective markers shown. Scale bar, 100 µm. Figure S18: Additional characterization of the enhancer *1056*. Mouse ES cell line J14 carrying enhancer *1056-βg-mCherry* were differentiated with our ES-MGE protocol. Expression of 1056-βg-mCherry (red) was examined on D9, 11, 13, 15 and 17 together with other markers (shown in green): (A–E) Lhx6-GFP, (F–J) Nkx2-1, (K-O) Mki67. Scale bar, 100 µm. Figure S19: Additional characterization of the enhancer *1538*. Mouse ES cell line J14 carrying enhancer *1538-βg-mCherry* were differentiated with our ES-MGE protocol. Expression of 1538-βg-mCherry (red) was examined on D10, 12, 14 and 16 together with other markers (shown in green): (A–D) Nkx2-1, (E–H) Mki67. Scale bar, 100 µm.
(PDF)

File S5 **Figure S20–S21.** Figure S20: All of the DlxI12b-βg-mCherry^+^ cells express Lhx6-GFP thirty-three days after transplantation into the neocortex (white arrows in A-A″). About 28% of Lhx6-GFP^+^ cells are also DlxI12b-mCherry^+^. One of the double positive cells (DlxI12b-βg-mCherry^+^, Lhx6-GFP^+^) is shown in B-B″. Scale bar for A-A″: 200 µm; for B-B″: 50 µm. Figure S21: Expression and colocalization of Olig2 and Nkx2-1 in the progenitor zones of the embryonic MGE. E11.5 coronal section through mouse forebrain showing Nkx2-1 (red), Olig2 (green), and DAPI (blue) as visualized by indirect immunofluorescence at the level of the MGE and LGE. At the ventricular zone and subventricular zone of the MGE, all of the cells are labeled by both Nkx2-1 and Olig2 (as shown by double labeling on the lower right panel). The images were taken at a Zeiss Confocal Microscope LSM 510 NLO Meta. Scale bar, 50 µm.
(PDF)
