# Supplementary material for: Use of “MGE Enhancers” for Labeling and Selection of Embryonic Stem Cell-Derived Medial Ganglionic Eminence (MGE) Progenitors and Neurons
Source: PLoS One. 2013 May 1;8(5):e61956. doi: 10.1371/journal.pone.0061956 (PMC3641041; doi:10.1371/journal.pone.0061956)
Supplement: File S1 — Text T1, Method M1 & M2, Figure S1–S11. Text T1: Dissociated MGE cells grown in vitro lose Lhx6-GFP expression. Method M1: MGE primary culture and antibodies for immunofluorescent studies. Method M2: Transient lentiviral infection in primary MGE and dissociated/differentiated ES cells. Figure S1: Primary MGE cells in vitro differentiate into Dlx2+ GABAergic neurons, but lose Lhx6 expression. A–D”, E13.5 MGE (ventricular and subventricular zone of the MGE) from Dlx5/6-LacZ+ embryos was removed from the telencephalon, dissociated and cultured in vitro using the media as described (Walton et al., 2006). Ten days after growing in the proliferation media (10 DIVpro), the cells were differentiated using differentiation media (DIVdiff). The state of differentiation was compared during proliferation (A-A″ and C-C″) or after 4 days of differentiation (B-B″ and D-D″) by immunostaining with antibodies to b-Galactosidase (β-Gal), GAD1 (GAD67), Dlx2 and Class III β-Tubulin (Tuj1). Scale bar, 100 µm. E–K, Lhx6-GFP expression in cells derived from E12.5 MGE (ventricular and subventricular zone of the MGE). The Lhx6-GFP+ MGEs were dissociated and cultured in vitro. Top row: MGE cells grown in proliferation media for 3 (E), 7 (F), 10 (G) and 13 (H) days. Cells were passaged by trypsinization and expansion onto bigger culture dishes on day 7. Bottom row: MGE cells cultured in proliferation media for seven days and then in differentiation media for 0 (I), 3 (J), and 6 (K) days. Images are overlay of DIC images and green fluorescent images. Scale bar, 150 µm. Figure S2: Comparison of various conditions for mouse ES cells differentiation. (A and A′): J14 was subjected to three different conditions for differentiation (the schematic was the same as shown in main figure 2 A) as shown. On D12 and D15, cells were analyzed by FACS analyses to determine the percentage of Lhx6-GFP+ cells. Both condition 1 (ES-MGE differentiation protocol, our current protocol, shown in blue) and condition 2 (M [file pone.0061956.s001.pdf]

Supplementary Text T1

Dissociated MGE cells grown *in vitro* lose Lhx6-GFP expression

We initially attempted to expand MGE progenitors directly from dissociated embryonic mouse MGE tissue. Because previous studies had been successful in expanding neural stem cells in serum-free or serum-containing media with the addition of epidermal growth factor (EGF) and basic fibroblast growth factor (bFGF, or FGF-2) (Conti et al., 2005; Walton et al., 2006), we tested several different protocols for MGE cells. We used MGE cells dissociated from E12.5/E13.5 transgenic embryos that expressed β-Galactosidase (β-Gal) or GFP in postmitotic MGE neurons, including immature cortical interneurons, under the control of a zebrafish *Dlx5/6* enhancer or a mouse *Lhx6-GFP* BAC transgene (Stuhmer et al., 2002; Gong et al., 2003; Cobos et al., 2006).

In serum containing media (proliferation media) (Walton et al., 2006), on adherent cultures, dissociated MGE ventricular zone (VZ) and subventricular zone (SVZ) cells from *Dlx5/6-β-gal* mice continued to proliferate *in vitro* for ~3 weeks (5 passages). Removal of growth factors and serum from the media (differentiation media) promotes neural differentiation (Walton et al., 2006), which similarly resulted in a significant increase in the number of β-Gal$^+$, GAD67$^+$, Dlx2$^+$ and Tuj1$^+$ cells from the MGE cultures (Figure S1A-D''). GAD67 and Dlx2 expression mark forebrain GABAergic cells; similarly, β-Gal driven by *Dlx5/6* enhancer is expressed by basal ganglia GABAergic progenitors and neurons. Tuj1 is a pan-neuronal marker. Therefore, this protocol can generate forebrain GABAergic neurons. To test whether these cells maintain MGE identity we followed the expression of *Lhx6*.

Using MGE cell cultures (grown and differentiated as above) from Lhx6-GFP transgenic mice, we found Lhx6-GFP$^+$ cells were present for 3-7 days *in vitro*. In proliferation media the MGE cells formed aggregates in which 30-50% of the cells were Lhx6-GFP$^+$ (Figure S1E-F). Prolonged culture (more than 10 days *in vitro*), or passage of cells that involved trypsinization (even with just one passage) resulted in a marked decrease in Lhx6-GFP$^+$ cells (Figure S1G-H). Neonatal cortical transplantation of MGE-derived cells after 21 days in culture resulted in no detectable GFP$^+$ cells in the adult cortex (N=10). Furthermore, despite the increase of GABAergic neurons generated in the differentiation protocol, we found that numbers of Lhx6-GFP$^+$ cells progressively decreased (Figure S1I-K). Therefore, this protocol was not effective at producing stable pools of MGE-derived Lhx6-GFP$^+$ neurons.

Finally, we attempted to maintain MGE identity using growth factors implicated in basal ganglia development (EGF, FGF-8, WNT-3a and Sonic hedgehog, individually and in combination) in a serum free media. However, this approach also failed to maintain Nkx2-1 and Lhx6-GFP expression, even after 1 passage (data not shown). Thus, we were unable to expand or maintain the identity of embryonic MGE cells *in vitro* with all of the methods (as described above), and concentrated on using ES cells to generate MGE-like neurons.

Materials and Methods

M1: MGE primary culture and antibodies for immunofluorescent studies

E12.5 or E13.5 MGE from transgenic mouse brains were dissected and dissociated into single cells with 0.05% Trypsin with 10 µg/ml DNase I at 37°C for 15 min. Defined proliferating media (Walton et al., 2006) included DMEM/F-12 glutamax (Invitrogen) with 5% FBS (Hyclone Defined Serum), 1X N2 (Invitrogen), 1X Pen/Strep (Cell Culture Facility at UCSF), 35 µg/ml bovine pituitary gland extract (Invitrogen), 20 ng/ml human bFGF (Peprotech) and 20ng/ml human EGF (Peprotech). For differentiation media, serum, pituitary gland extracts and both growth factors were removed from the proliferation media. For the serum free media, RHB-basal media (Stem Cell Sciences) was supplemented with 1X N2 (Millipore), 10ng/ml EGF, 100ng/ml FGF-8 (Peprotech), 5ng/ml WNT-3a (R&D System) and 250ng/ml Sonic hedgehog N-terminus (Shh-N) (R&D System). With the serum free media MGE cells required either gelatin or laminin-treated culture plates. MGE cells under the influence of all 4 growth factors continued to proliferate *in vitro* for up to 10 passages (~7 weeks).

Primary antibodies were guinea pig anti-b-Gal (1:500, kindly provided by Thomas Finger, University of Colorado) (Yee et al., 2003), mouse ant-GAD67 (1:1000, Millipore).

M2: Transient lentiviral infection in primary MGE and dissociated/differentiated ES cells

E13.5 MGE from wild type mouse brains were dissected and dissociated into single cells as described in the main text. For differentiated ES cells, D11 aggregates were collected and dissociated with 0.05% Trypsin with 10 µg/ml DNase I for 20 min. Twenty thousand primary MGE or ES cells were incubated with each of the lentiviruses for one hour in a 1.5ml microcentrifuge tube at 37ºC water bath, and then cells were seeded in poly-L-lysine/laminin coated 16-well slide chambers overnight in DMEM media (10% FBS) with the viruses. The next day, viral-containing media was removed and new media added. For MGE primary cells, the defined proliferation media was added; for differentiated ES cells, DMEM/F-12 with N2 supplement was added. Three days after infection, cells were washed and fixed with 4% paraformaldehyde before immunostaining.

References for the Text T1:

Cobos I, Long JE, Thwin MT, Rubenstein JL (2006) Cellular patterns of transcription factor expression in developing cortical interneurons. Cereb Cortex 16 Suppl 1:i82-88.

Conti L, Pollard SM, Gorba T, Reitano E, Toselli M, Biella G, Sun Y, Sanzone S, Ying QL, Cattaneo E, Smith A (2005) Niche-independent symmetrical self-renewal of a mammalian tissue stem cell. PLoS biology 3:e283.

Gong S, Zheng C, Doughty ML, Losos K, Didkovsky N, Schambra UB, Nowak NJ, Joyner A, Leblanc G, Hatten ME, Heintz N (2003) A gene expression atlas of the central nervous system based on bacterial artificial chromosomes. Nature 425:917-925.

Stuhmer T, Puelles L, Ekker M, Rubenstein JL (2002) Expression from a Dlx gene enhancer marks adult mouse cortical GABAergic neurons. Cereb Cortex 12:75-85.

Walton NM, Sutter BM, Laywell ED, Levkoff LH, Kearns SM, Marshall GP, 2nd, Scheffler B, Steindler DA (2006) Microglia instruct subventricular zone neurogenesis. Glia 54:815-825.

Yee CL, Jones KR, Finger TE (2003) Brain-derived neurotrophic factor is present in adult mouse taste cells with synapses. The Journal of comparative neurology 459:15-24.

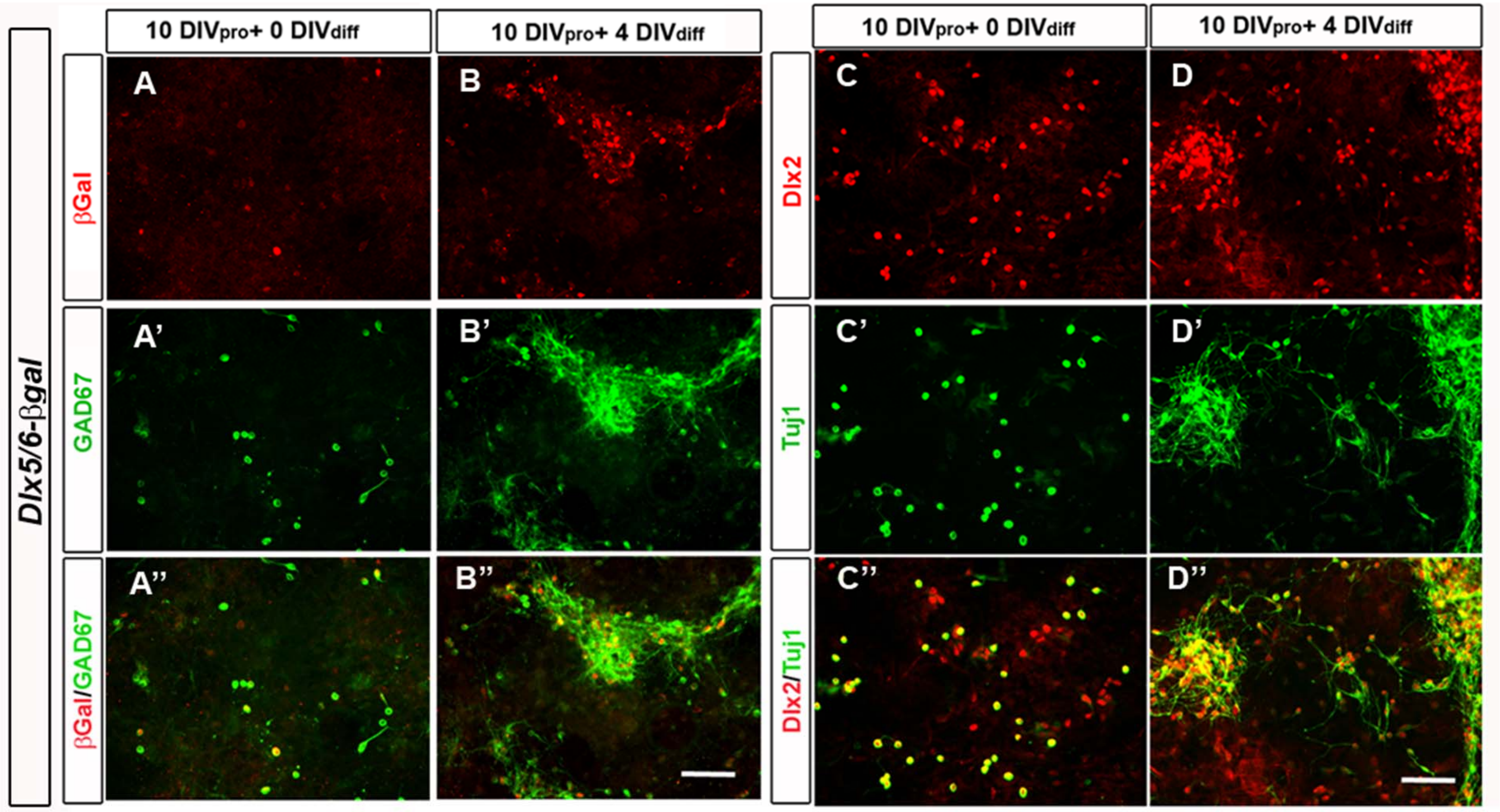

Figure S1: Primary MGE cells in vitro differentiate into Dlx2⁺ GABAergic neurons, but lose Lhx6 expression. ***A-D″***, E13.5 MGE (ventricular and subventricular zone of the MGE) from Dlx5/6-LacZ⁺ embryos was removed from the telencephalon, dissociated and cultured *in vitro* using the media as described (Walton et al., 2006). Ten days after growing in the proliferation media (10 DIV_{pro}), the cells were differentiated using differentiation media (DIV_{diff}). The state of differentiation was compared during proliferation (***A- A″*** and ***C-C″***) or after 4 days of differentiation (***B-B″*** and ***D-D″***) by immunostaining with antibodies to β-Galactosidase (β-Gal), GAD1 (GAD67), Dlx2 and Class III β-Tubulin (Tuj1). Scale bar, 100 μm.

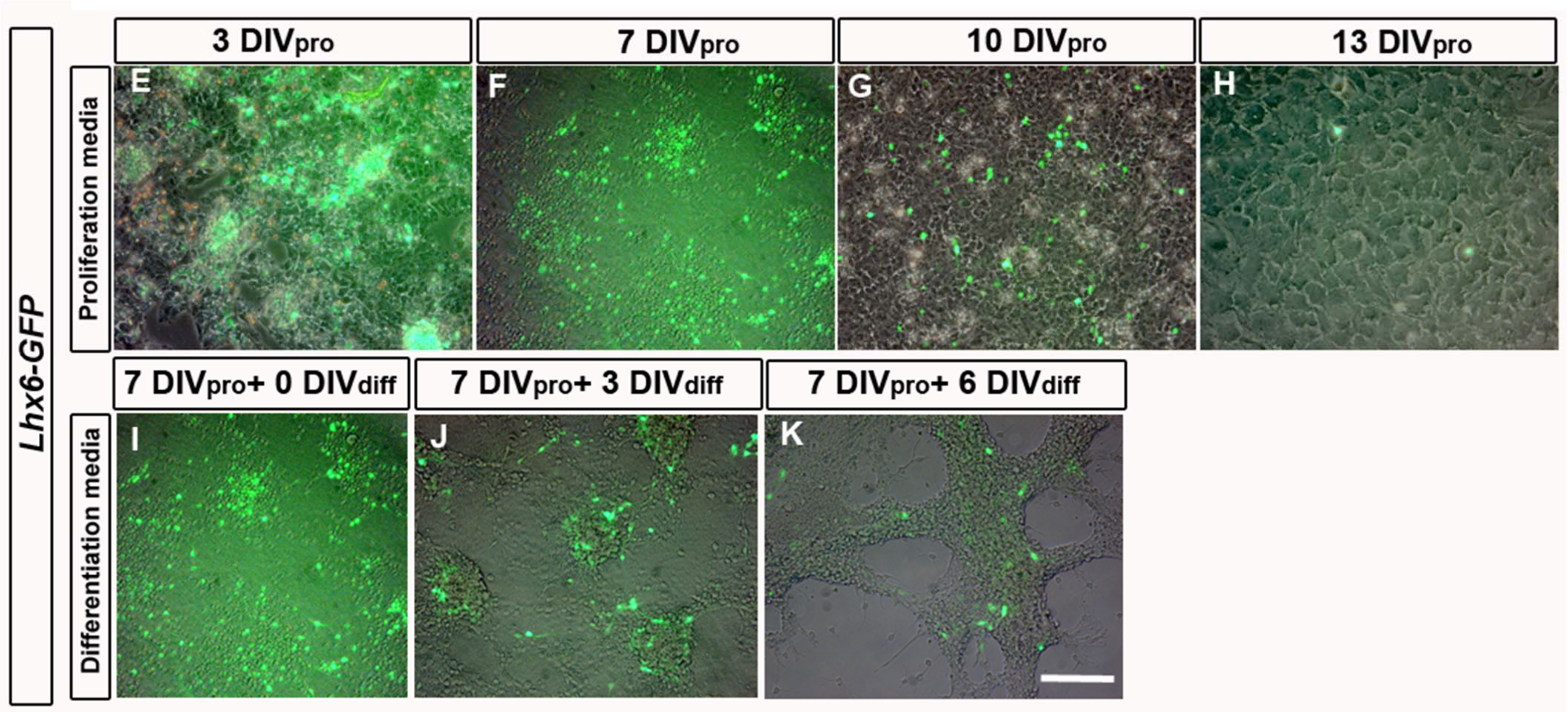

Figure S1: Primary MGE cells in vitro differentiate into Dlx2^{+} GABAergic neurons, but lose Lhx6 expression.
E-K, Lhx6-GFP expression in cells derived from E12.5 MGE (ventricular and subventricular zone of the MGE). The Lhx6-GFP^{+} MGEs were dissociated and cultured *in vitro.* Top row: MGE cells grown in proliferation media for 3 (***E***), 7 (***F***), 10 (***G***) and 13 (***H***) days. Cells were passaged by trypsinization and expansion onto bigger culture dishes on day 7. Bottom row: MGE cells cultured in proliferation media for seven days and then in differentiation media for 0 (***I***), 3 (***J***), and 6 (***K***) days. Images are overlay of DIC images and green fluorescent images. Scale bar, 150µm.

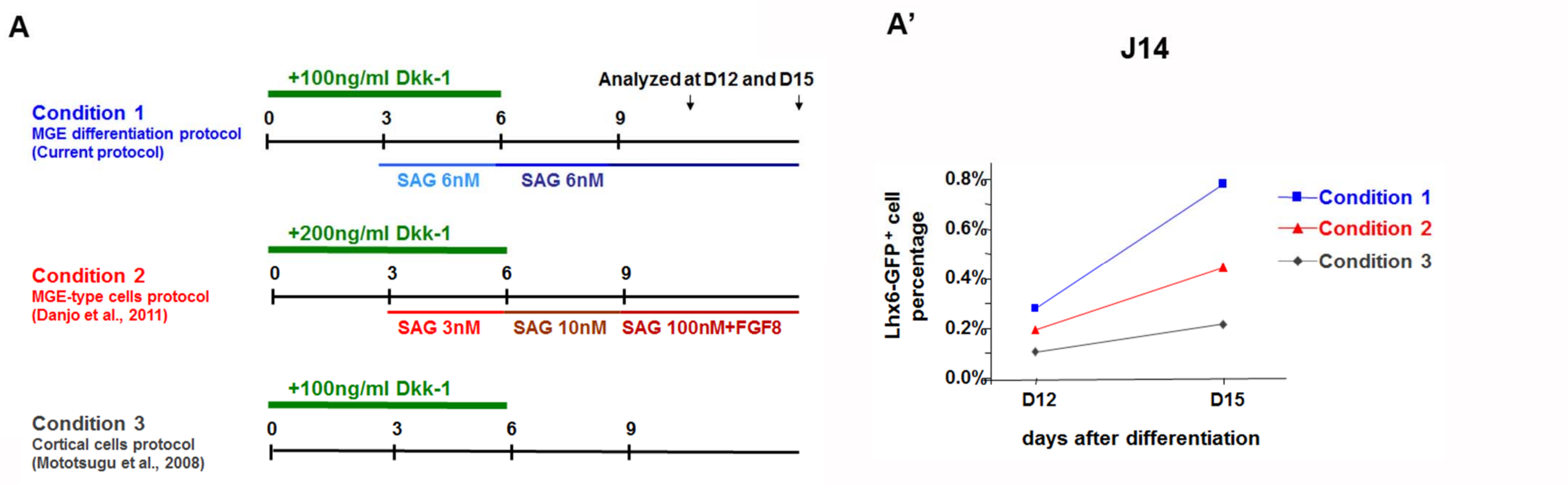

Figure S2: Comparison of various conditions for mouse ES cells differentiation.
(A and A'): J14 was subjected to three different conditions for differentiation (the schematic was the same as shown in main figure 2 A) as shown. On D12 and D15, cells were analyzed by FACS analyses to determine the percentage of Lhx6-GFP$^+$ cells. Both condition 1 (ES-MGE differentiation protocol, our current protocol, shown in blue) and condition 2 (MGE-type cells protocol, shown in red, (Danjo et al., 2011)) included Shh pathway activators and promoted MGE-like progenitor cells. Condition 3 (cortical cell protocol, in grey, (Eiraku et al., 2008): addition of Dkk-1, without Shh or SAG) generated very few Lhx6-GFP$^+$ cells from J14 cell line. (A') Compared to condition 2 and 3, condition 1 produced the most of Lhx6-GFP$^+$ cells on D12 and D15 of differentiation.

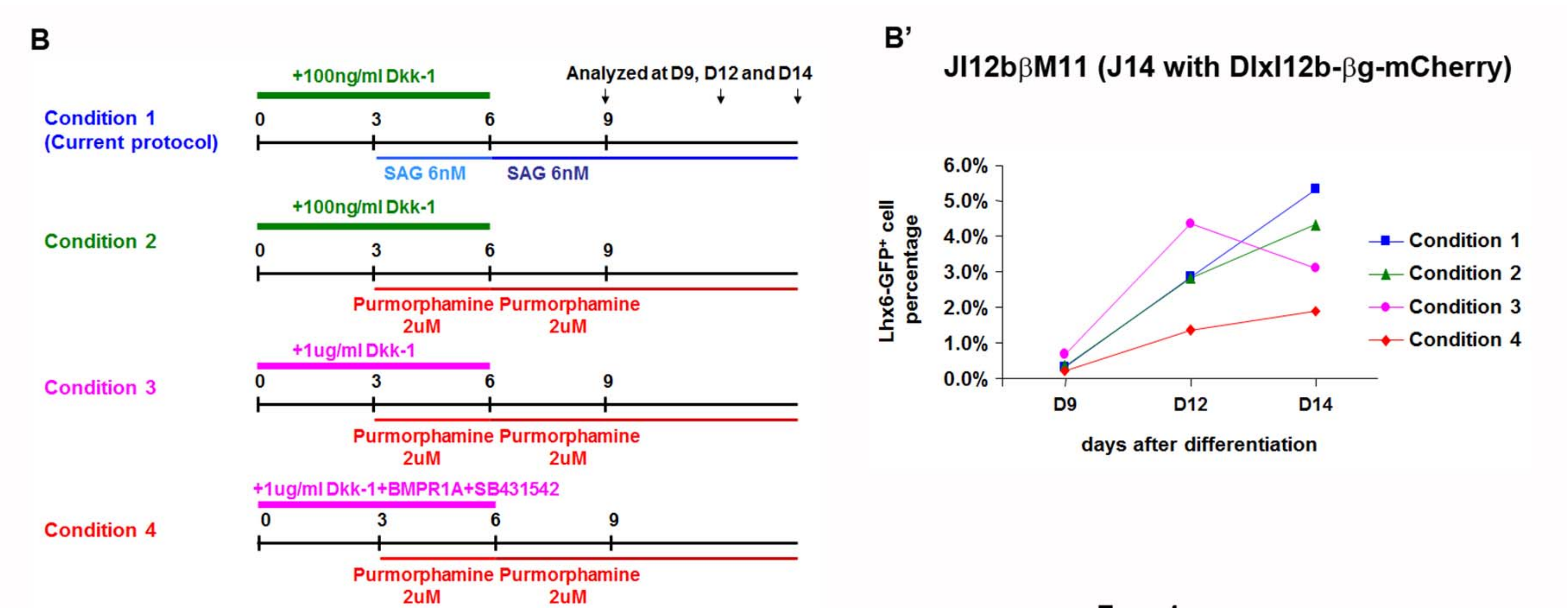

Figure S2: Comparison of various conditions for mouse ES cells differentiation.
(B and B'): Four conditions were used to differentiate JI12bBM11 cell line (J14 carrying *Dlxl12b-βg-mCherry*). In Condition 1 (shown in blue) 6nM SAG was applied while in condition 2 (in green) 2μM purmophamine was used to promote MGE-like progenitor cells. In condition 3 (in purple), 1μg/ml Dkk-1 was added on D0, in compared to 100ng/ml Dkk-1 in condition 1 and 2. In condition 4 (shown in red), additional growth factors and small molecule (BMPR1A and SB431542) was added on day 0. (B') Much more purmophamine is required (2μM) (Condition 2) to reach the same efficiency generated by SAG (6nM) (Condition 1). Ten times more Dkk-1 (1μg/ml) (Condition 3) only produced slightly more Lhx6-GFP+ cells on D12 but not on D14. Addition of activin/nodal inhibitor SB431542 and BMP activator BMPR1A on D0 (Condition 4) diminished the effects of 1μg/ml Dkk-1 on Lhx6-GFP+ cells production.

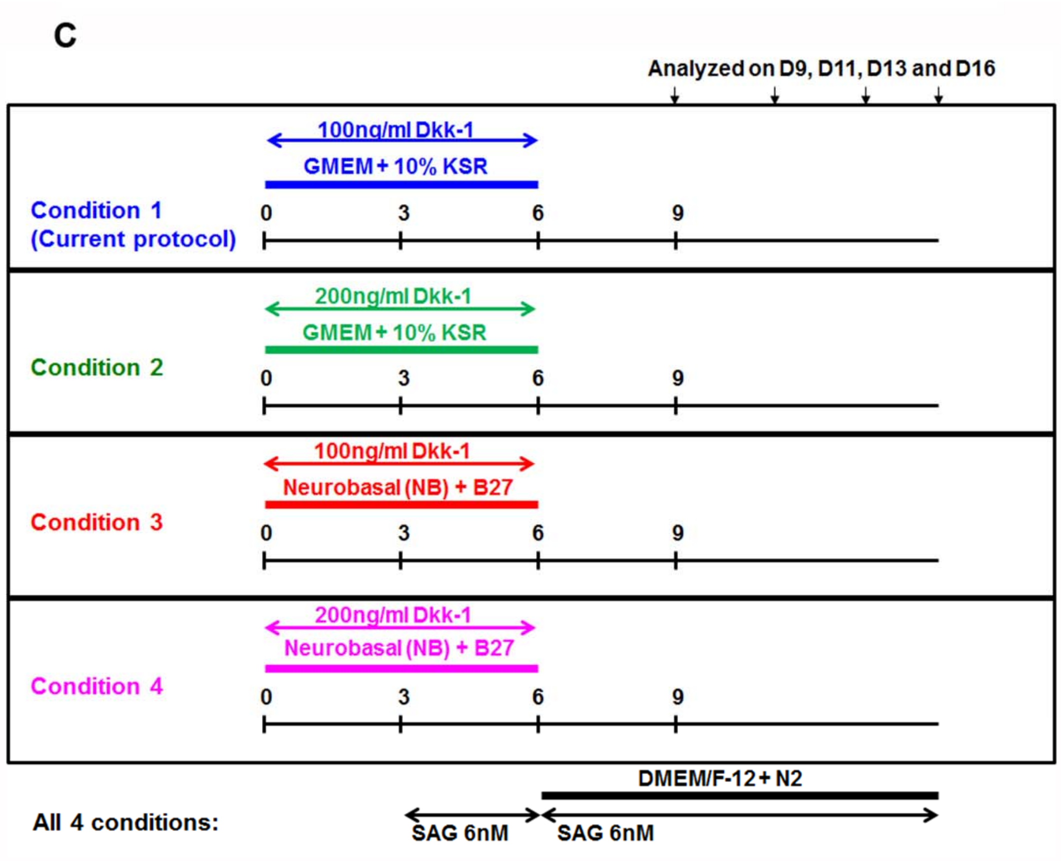

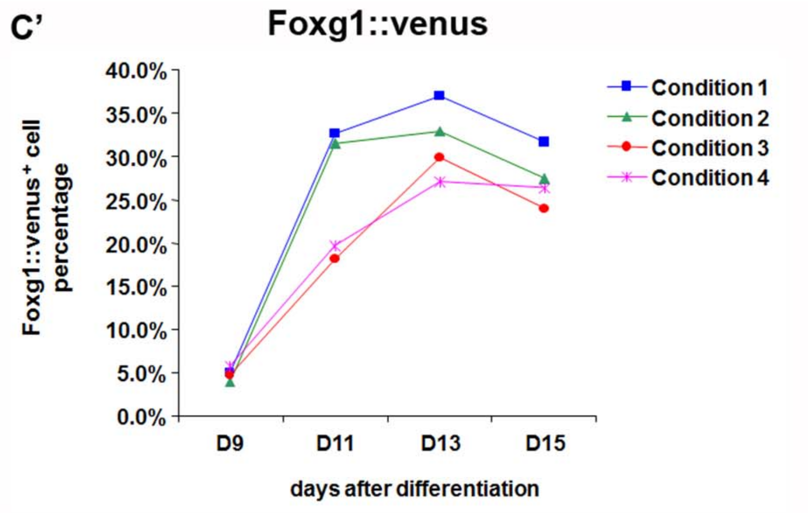

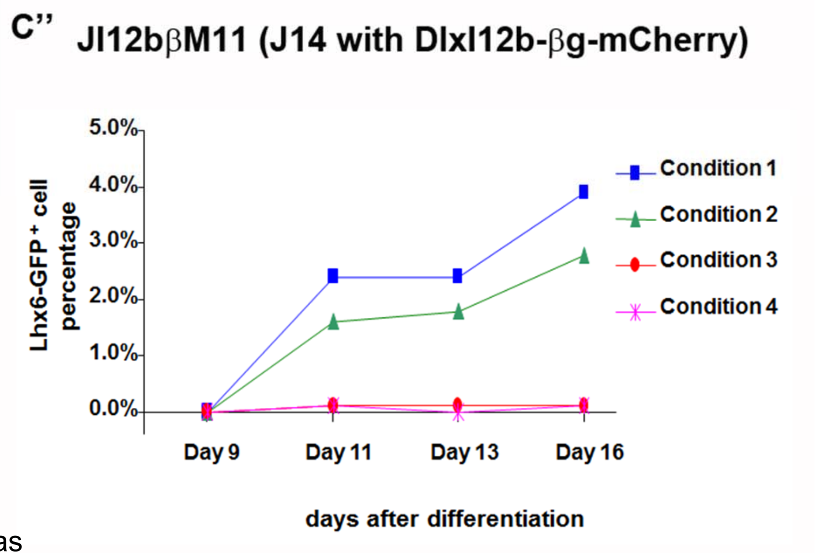

Figure S2: Comparison of various conditions for mouse ES cells differentiation.

(C, C' and C''): Foxg1::venus and Jl12bBM11 (J14 carrying lentiviral enhancer Dlxl12b-βg-mCherry) were tested for differentiation using four conditions listed (as shown in Figure 1). In condition 1 and 2 (shown in blue and green), cells were differentiated in GMEM+10% KSR media while in condition 3 and 4 (shown in red and purple), cells were differentiated in Neurobasal media supplemented with B27 without retinoic acid (NB/B27), a commonly used media for neural progenitor differentiation (Turksen and Troy, 2006). Either 100 or 200ng/ml Dkk-1 was added on D0. For all three cell lines tested (including J6M1 in Figure 1), KSR-containing media surpassed NB/B27 media in the generation of Foxg1::venus^{+} cells or Lhx6-GFP^{+} cells. Addition of 2X more Dkk-1 on D0 did not improve the efficiency of Lhx6-GFP^{+} cells or Foxg1::venus^{+} production with KSR media.

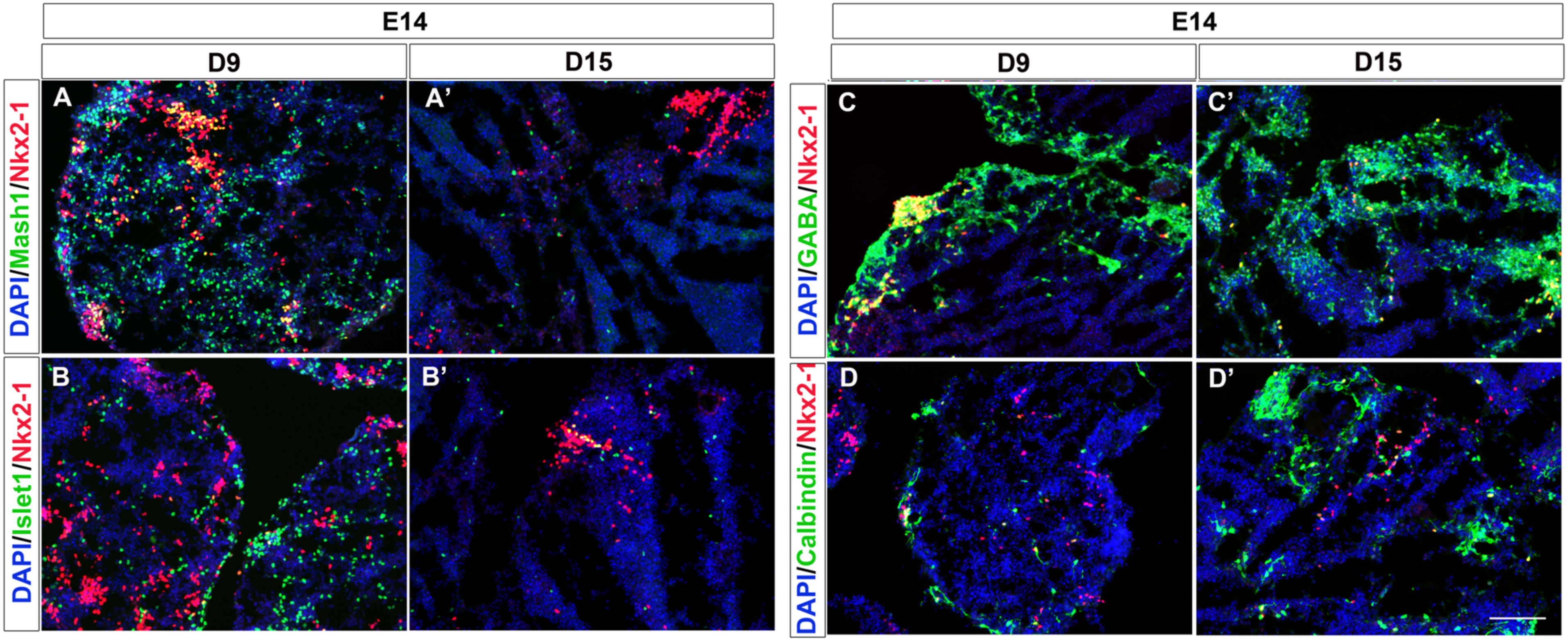

Figure S3: Characterization of differentiated E14 cells
Expression of Nkx2-1 (red in all panels) with other markers (shown in green): Mash1 (***A, A'***), Islet1 (***B, B'***), GABA (***C, C'***), and Calbindin (***D, D'***), in E14 cell line on D9 and D15 after differentiation. DAPI nucleus staining was shown in blue in all panels. There are more Mash1$^+$ cells than Nkx2-1$^+$ cells (and some of them express both proteins) on D9. On D15, both protein expressions are reduced with more Nkx2-1$^+$ cells than Mash1$^+$ cells. Scale bar: 100µm.

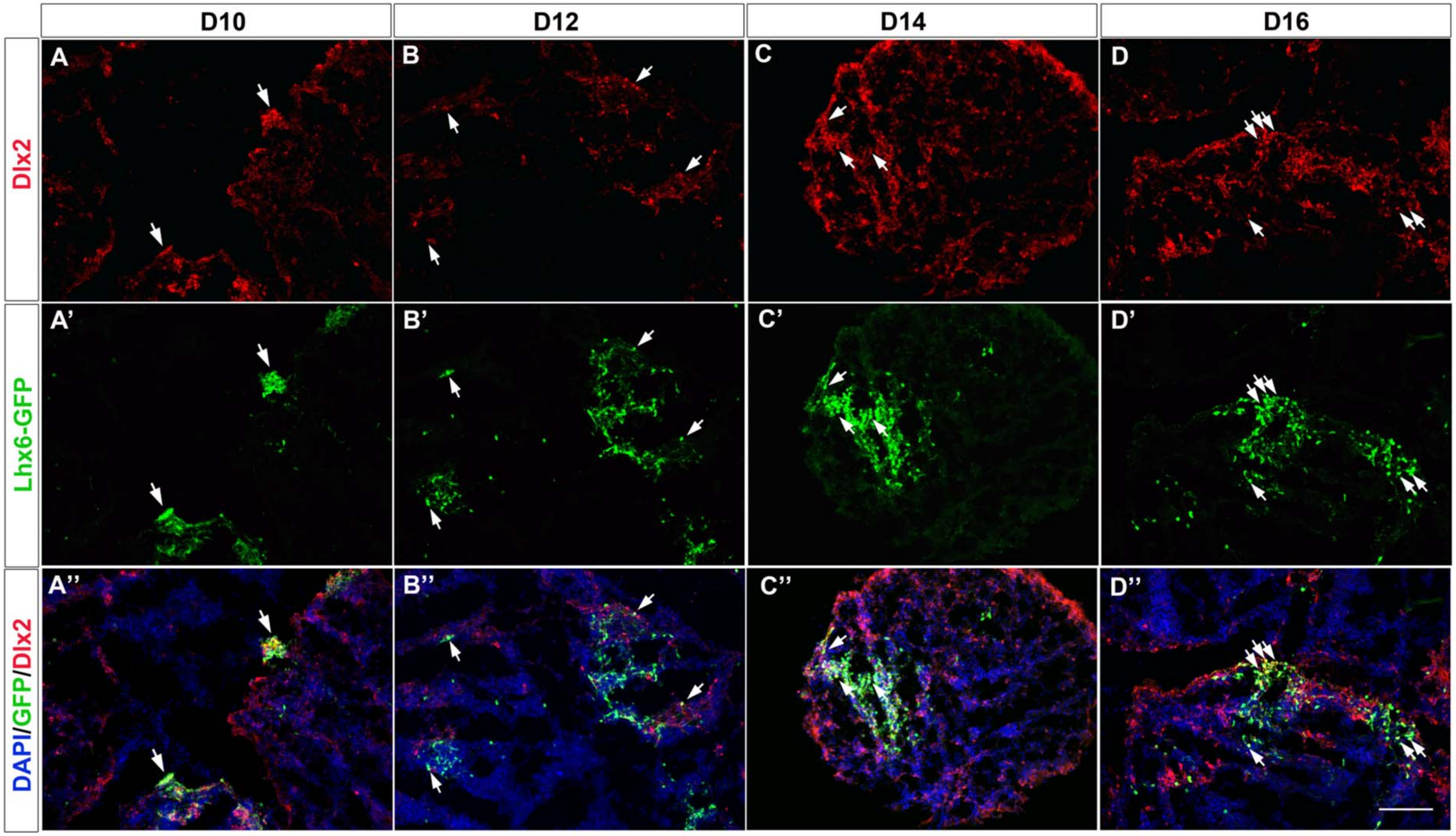

Figure S4: Expression of Lhx6-GFP and Dlx2 in J14 cells.
J14 cells were differentiated with our ES-MGE protocol. On day 10 (D10; A, A', A''), D12 (B, B', B''), D14 (C, C', C'') and D16 (D, D', D''), cell aggregates were collected for analyses by immunofluorescent staining for Dlx2 (red) and GFP (green). Similar to Lhx6-GFP$^+$ cells, the number of Dlx2$^+$ cells peaked on D12-14. Arrows indicate co-localization of Dlx2 and Lhx6-GFP. Scale bar, 100μm.

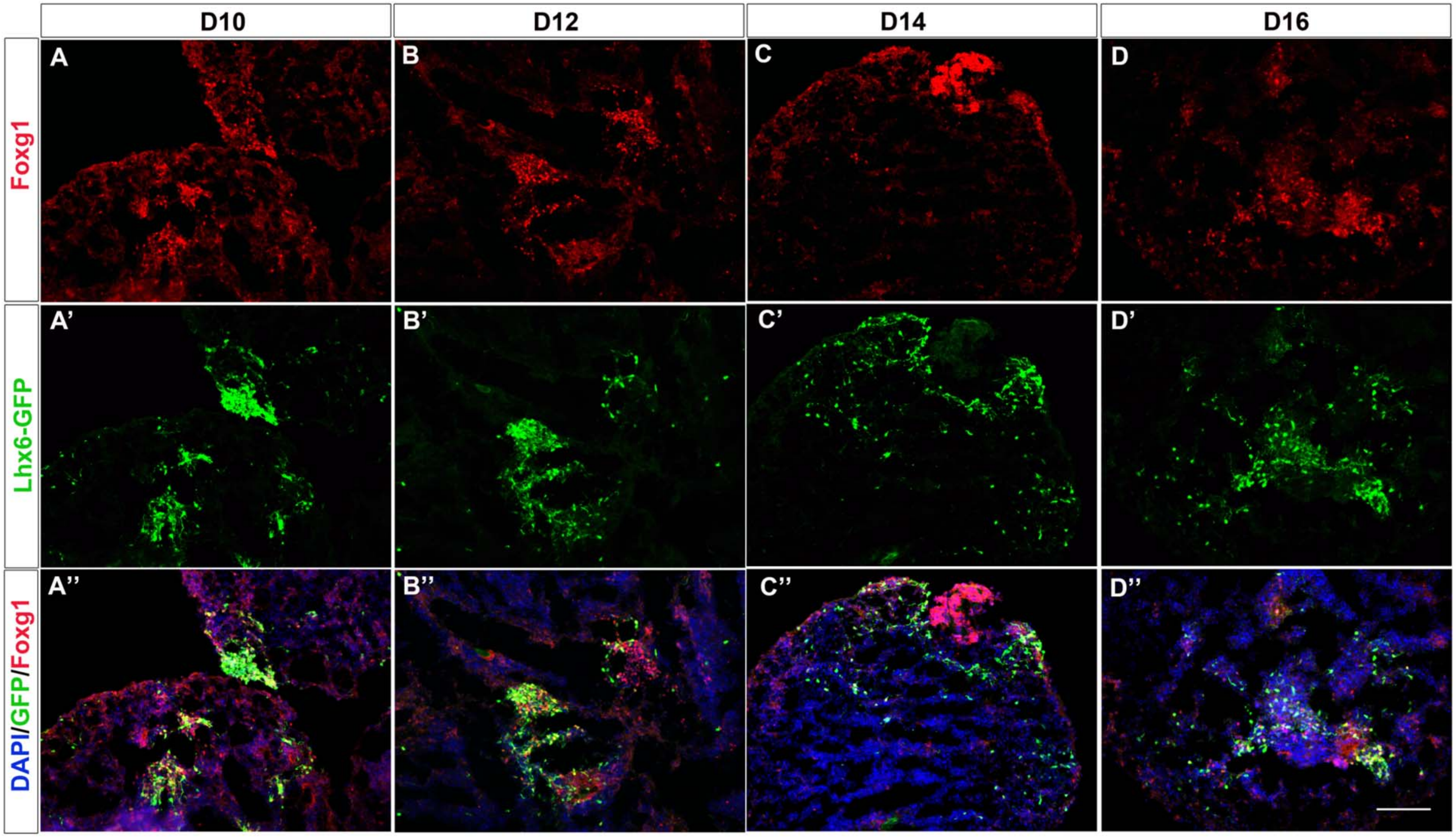

Figure S5: Expression of Lhx6-GFP and Foxg1 in J14 cells.
J14 cells were differentiated with our ES-MGE protocol. Expression of Foxg1 (red) and Lhx6-GFP (green) were examined from cell aggregates collected on D10 (A, A', A''), D12 (B, B', B''), D14 (C, C', C'') and D16 (D, D', D''). The expression of Foxg1 was highest at D10-D12 of differentiation and went down at D14-D16. Scale bar, 100μm.

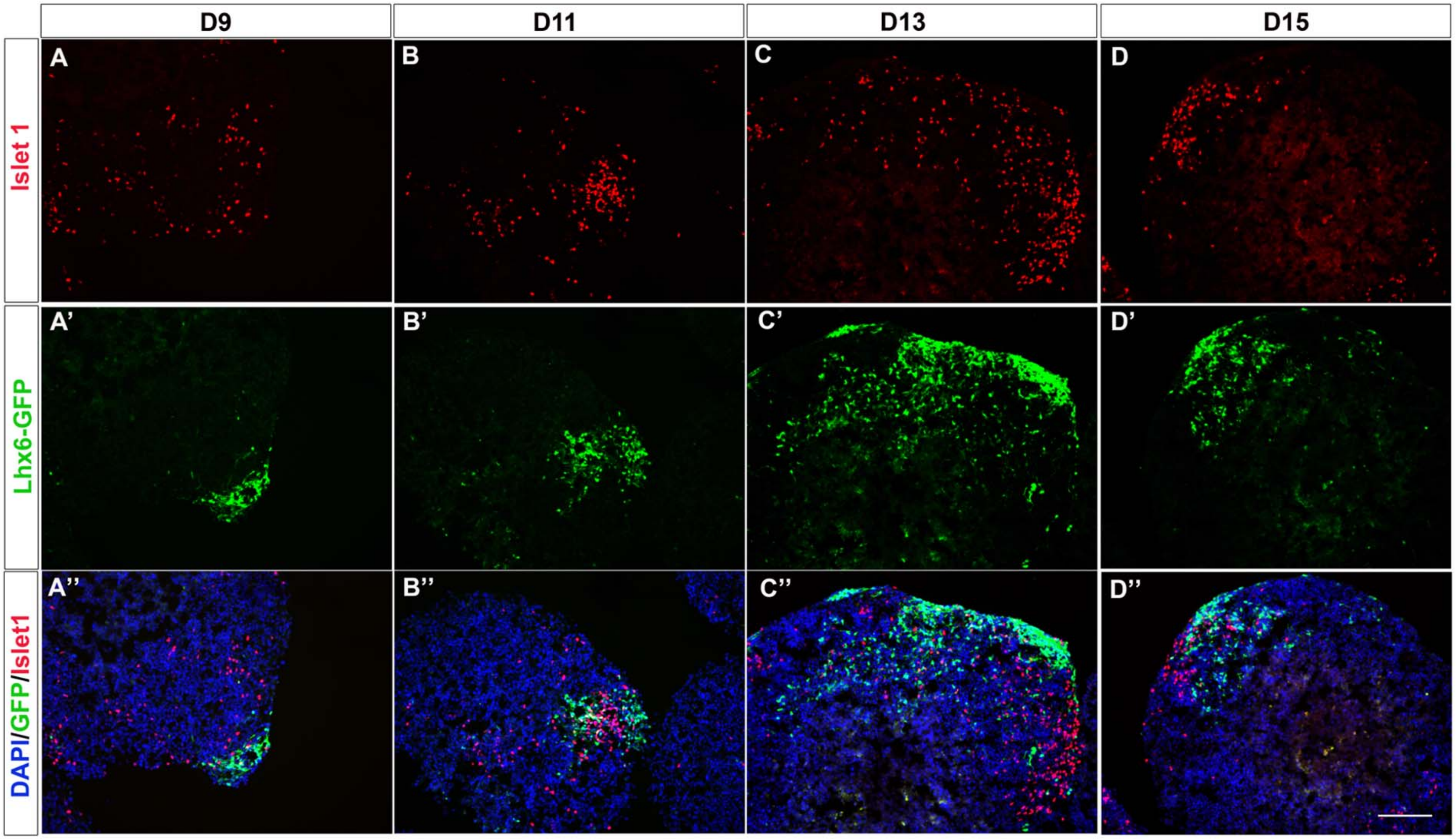

Figure S6: Expression of Lhx6-GFP and Islet1 in J14 cells.
J14 cells were differentiated with our ES-MGE protocol. On D9 (A, A', A''), D11 (B, B', B''), D13 (C, C', C'') and D15 (D, D', D''), cell aggregates were collected for analyses by immunofluorescent staining: Islet1 (red) and GFP (green). Note the random distribution of Islet^{+} cells within the aggregates compared to the clustered Lhx6-GFP^{+} cells. Scale bar, 100μm.

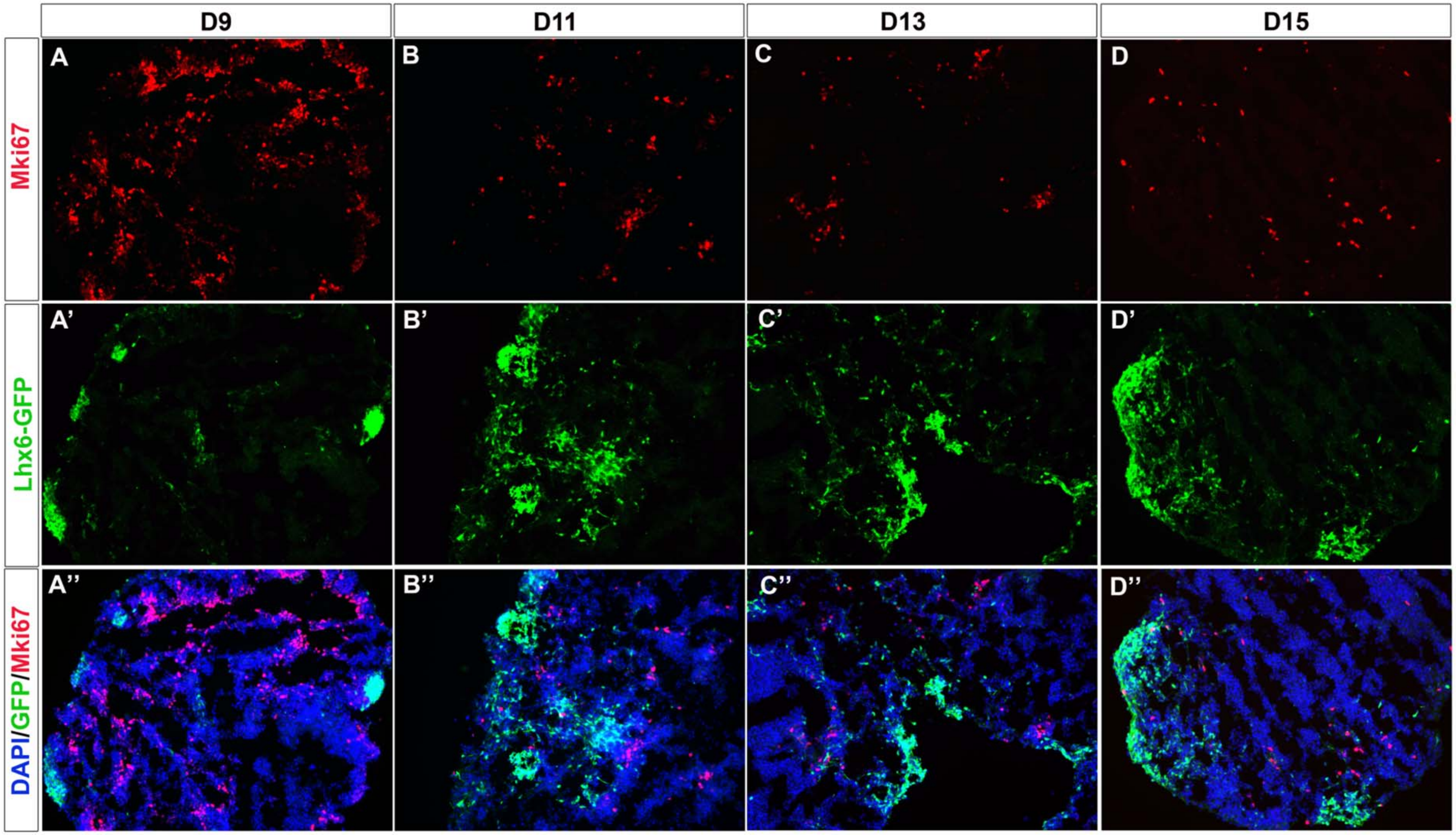

Figure S7: Expression of Lhx6-GFP and Mki67 in J14 cells.
J14 cells were differentiated with our ES-MGE protocol. On D9 (A, A', A''), D11 (B, B', B''), D13 (C, C', C'') and D15 (D, D', D''), cell aggregates were collected for analyses by immunofluorescent staining: Mki67 (red) and GFP (green). All of the Lhx6-GFP^{+} cells were postmitotic. Scale bar, 100µm.

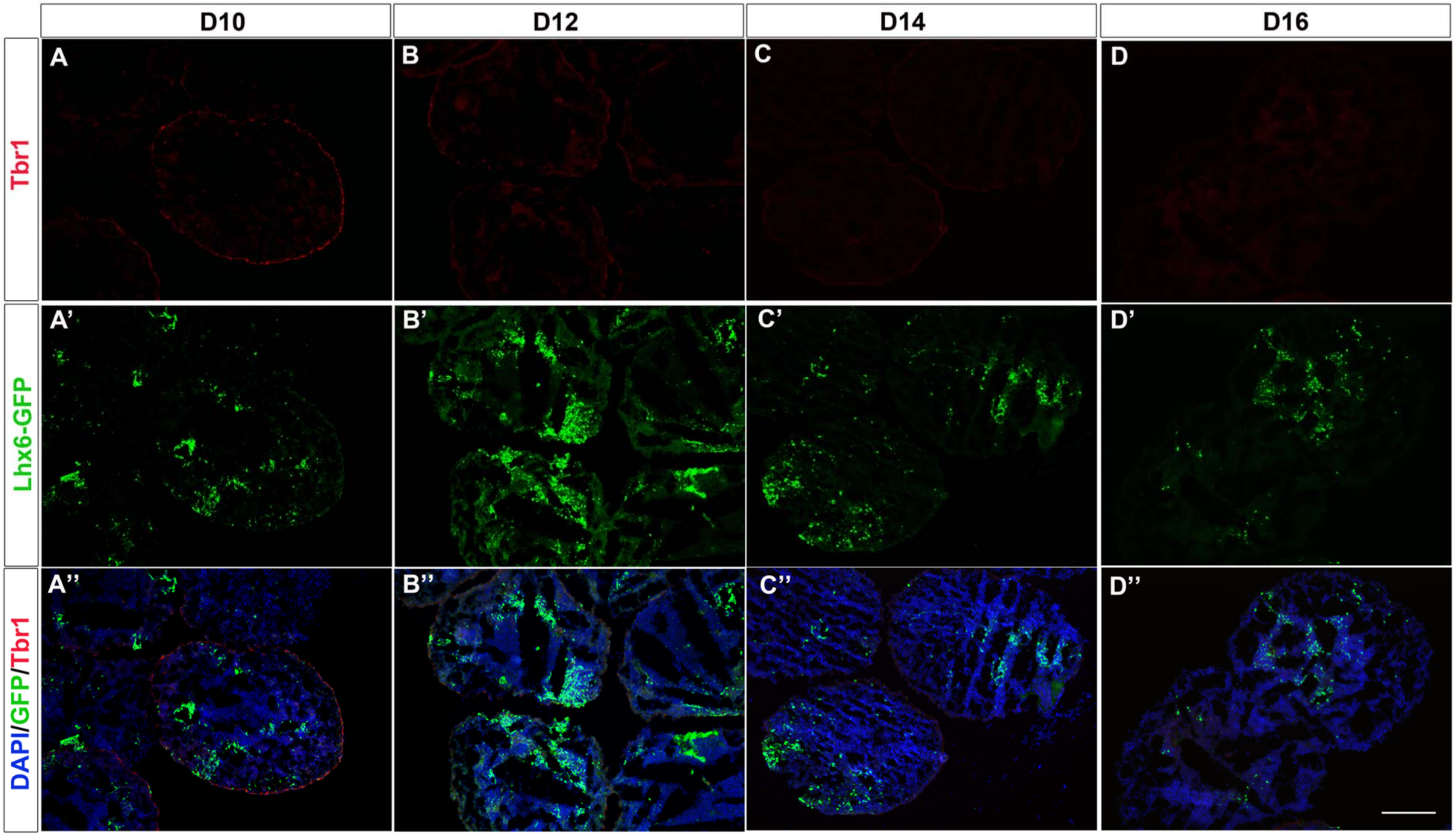

Figure S8: Expression of Lhx6-GFP and Tbr1 in J14 cells.
J14 cells were differentiated with our ES-MGE protocol. On D10 (A, A', A''), D12 (B, B', B''), D14 (C, C', C'') and D16 (D, D', D''), cell aggregates were collected for analyses by immunofluorescent staining: Tbr1 (red) and GFP (green). There was no $Tbr1^{+}$ cell in any time point examined. Scale bar, 200µm.

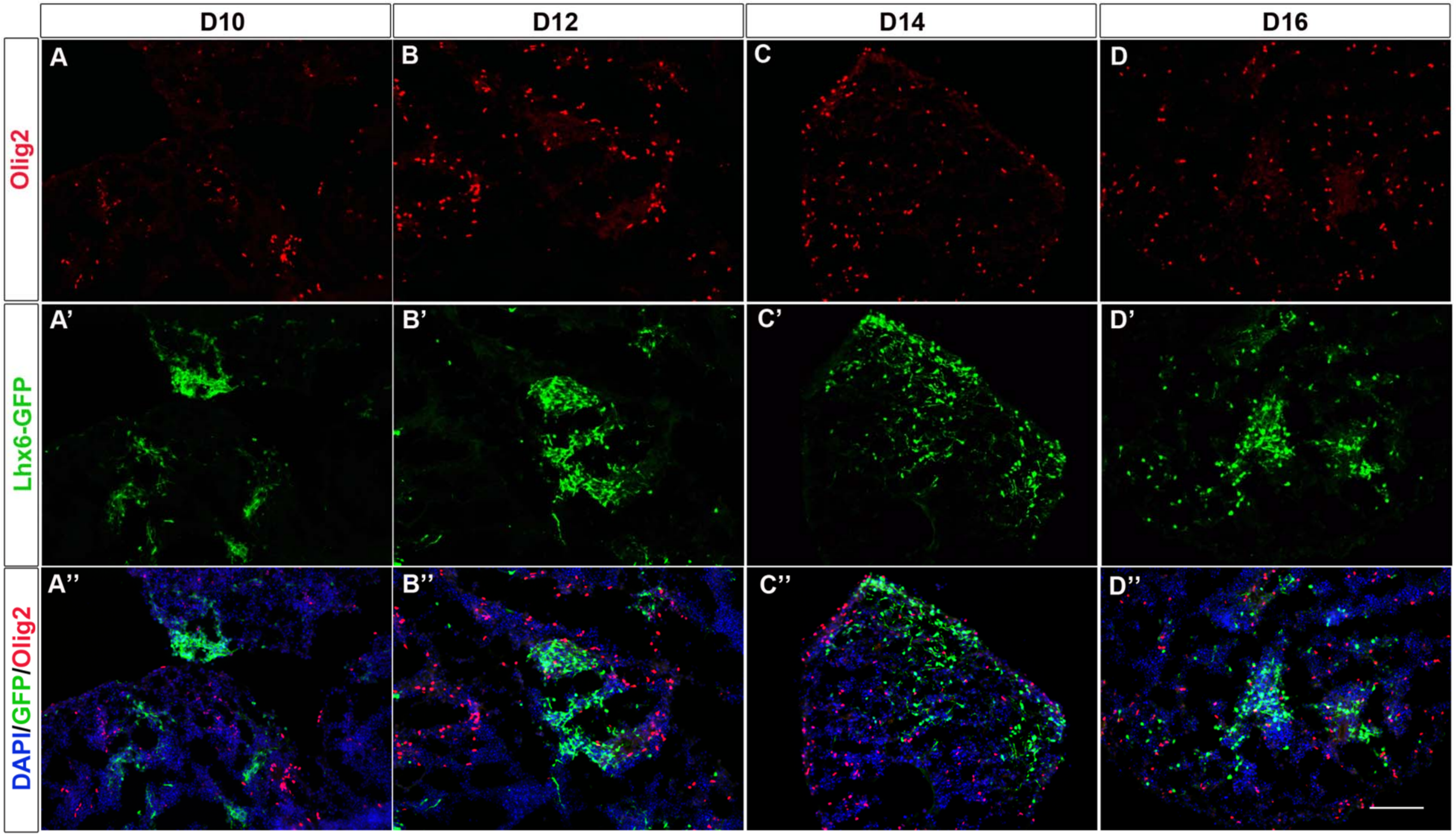

Figure S9: Expression of Lhx6-GFP and Olig2 in J14 cells.
J14 cells were differentiated with our ES-MGE protocol. On D10 (A, A', A''), D12 (B, B', B''), D14 (C, C', C'') and D16 (D, D', D''), cell aggregates were collected for analyses by immunofluorescent staining: Olig2 (red) and GFP (green). Similar to Lhx6-GFP$^+$ cells, the number of Olig2$^+$ cells peaked on D12-14. All of the Lhx6-GFP$^+$ cells were Olig2$^-$. Scale bar, 100μm.

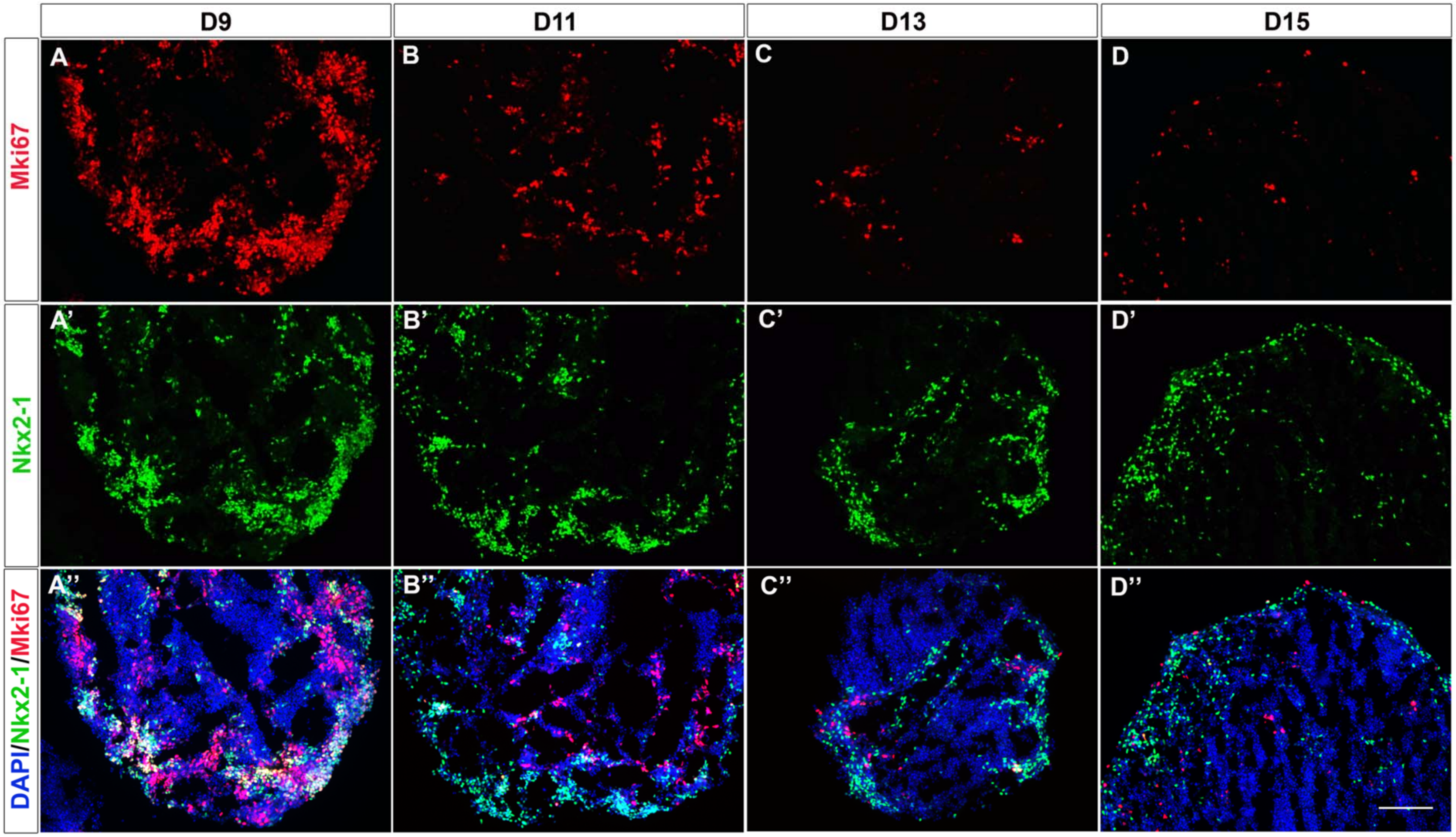

Figure S10: Expression of Nkx2-1 and Mki67 in J14 cells.
J14 cells were differentiated with our ES-MGE protocol. On D9 (A, A', A''), D11 (B, B', B''), D13 (C, C', C'') and D15 (D, D', D''), cell aggregates were collected for analyses by immunofluorescent staining: Mki67 (red) and Nkx2-1 (green). Plenty of Mki67^{+} cells were present and co-labeled with Nkx2-1 on day 9 and the number of Ki67^{+} cells went down on subsequent time points. There were significantly lower Nkx2-1^{+} that were also Mki67^{+} on D11 and D13. Scale bar, 100μm.

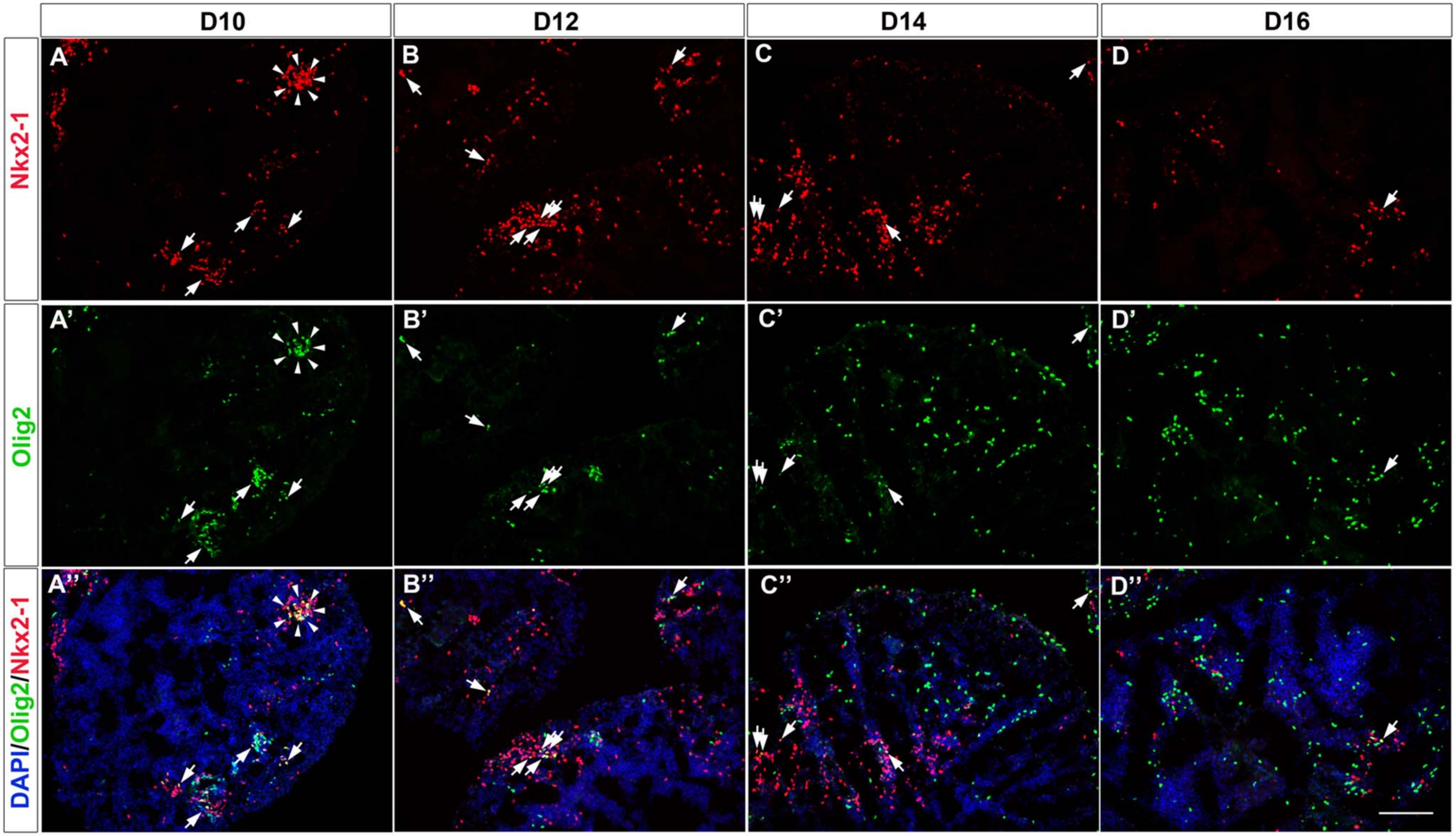

Figure S11: Expression of Nkx2-1 and Olig2 in J14 cells.
J14 cells were differentiated with our ES-MGE protocol. On D10 (A, A', A''), D12 (B, B', B''), D14 (C, C', C'') and D16 (D, D', D''), cell aggregates were collected for analyses by immunofluorescent staining: Nkx2-1(red) and Olig2 (green). About 5% of Olig2^{+} cells co-labeled with Nkx2-1 on D9 and the number of Nkx2-1^{+}/Olig2^{+} (double positive) cells decreased on subsequent time points. Scale bar, 100μm.
