# Supplementary material for: Use of “MGE Enhancers” for Labeling and Selection of Embryonic Stem Cell-Derived Medial Ganglionic Eminence (MGE) Progenitors and Neurons
Source: PLoS One. 2013 May 1;8(5):e61956. doi: 10.1371/journal.pone.0061956 (PMC3641041; doi:10.1371/journal.pone.0061956)
Supplement: File S2 — Table S1–S10. Table S1: Abbreviated Names. Table S2: Select marker genes expression from differentiated ES cells (ES Lhx6-GFP+ and ES Lhx6-GFP−) and E12.5 MGE cells (MGE Lhx6-GFP+) and the comparisons (fold change) of ES Lhx6-GFP+ vs. ES Lhx6-GFP−, MGE Lhx6-GFP+ vs. ES Lhx6-GFP−, and MGE Lhx6-GFP+ vs. ES Lhx6-GFP+. Column 1 lists marker genes for specific cell types and regions. Note that many of these are not specific for those cells states, but are recognized as useful markers. The expression levels in the columns 2–4 represent the averaged normalized log2 intensity for each gene. The numbers in columns 5–7 (the fold change) are ratios of the average signal intensity (unlogged) of the two groups in comparison. Red colored genes are enriched in ES Lhx6-GFP− cells whereas green colored genes are enriched in both MGE Lhx6-GFP+ and ES Lhx6-GFP+ cells. For most of the genes, the expression in the ES Lhx6-GFP+ cells and MGE Lhx6-GFP+ cells show similar expression trends, in comparison to ES Lhx6-GFP− cells. However, there are a few genes (shown in black) that don't follow this trend. Table S3: Block A and Block B from the supervised clustering map (Figure 3). Genes from block A and B of the supervised heatmap (Figure 3) are list below (the order of the genes are the same as in the map (from top to bottom). Many of the genes that regulate and/or mark developing cortical interneurons as shown in Tables 1 and S2 are bold-typed. Table S4: Cell counts from 69 days after transplantation. Four transplants (mice that received Lhx6-GFP+ cells and had at least 10 Lhx6-GFP+ cells in the cortex: N1, R3, R5 and R6) were examined for co-labeling of Lhx6-GFP with parvalbumin (PV), somatostatin (SOM), and neuropeptide Y (NPY). Total numbers, average, and standard errors from 3–4 transplants were shown. Table S5: Comparison of enhancer activities in transgenic embryos and differentiated ES cells. Table S6: Differentially expressed (Fold change are > = 4) genes between ES Lhx6-GFP+ vs ES [file pone.0061956.s002.pdf]

Supplementary Table S1: Abbreviated Names

Abbreviated name	Full name
BAC	bacterial artificial chromosome
β-Gal	β -Galactosidase
Calb1	Calbindin
CGE	caudal ganglionic eminence
DAPI	4’, 6-diamidino-2-phenylindole
Dkk-1	Dickkopf-1
DlxI12b	*Dlx1* and *Dlx2* intragenic enhancer
EB	embryoid body
ES cells	Embryonic stem cells
FBS	fetal bovine serum
FACS	fluorescent activated cell sorting
GMEM	Glasgow Minimum Essential Medium
KSR	Knockout Serum Replacement
LGE	lateral ganglionic eminence
LIF	leukaemic Inhibitory Factor
MGE	medial ganglionic eminence
MZ	mantle zones
NB	Neurobasal
NEAA	nonessential amino acids
NPY	Neuropeptide Y
OCT	optimal cutting temperature
POA	preoptic area
SFEB	serum-free embryoid body-like
SFEBq	improved "serum-free embryoid body-like"
Shh	Sonic hedgehog
Sst	Somatostatin

SVZ	subventricular zone
VZ	ventricular zone
X-Gal	5-bromo-4-chloro-3-indolyl β-D-galactopyranoside

Supplementary Table S2. Select marker genes expression from differentiated ES cells (ES Lhx6-GFP^{+} and ES Lhx6-GFP^{-}) and E12.5 MGE cells (MGE Lhx6-GFP^{+}) and the comparisons (fold change) of ES Lhx6-GFP^{+} vs. ES Lhx6-GFP^{-}, MGE Lhx6-GFP^{+} vs. ES Lhx6-GFP^{-}, and MGE Lhx6-GFP^{+} vs. ES Lhx6-GFP^{+}.

Column 1 lists marker genes for specific cell types and regions. Note that many of these are not specific for those cells states, but are recognized as useful markers.

The expression levels in the columns 2-4 represent the averaged normalized log2 intensity for each gene. The numbers in columns 5-7 (the fold change) are ratios of the average signal intensity (unlogged) of the two groups in comparison.

Red colored genes are enriched in ES Lhx6-GFP^{-} cells whereas green colored genes are enriched in both MGE Lhx6-GFP^{+} and ES Lhx6-GFP^{+} cells. For most of the genes, the expression in the ES Lhx6-GFP^{+} cells and MGE Lhx6-GFP^{+} cells show similar expression trends, in comparison to ES Lhx6-GFP^{-} cells. However, there are a few genes (shown in black) that don't follow this trend.

1	2	3	4	5	6	7
Areas or cells of interest	Expression levels			Comparison between groups (fold changes)		
Genes of interest	ES Lhx6-GFP^{+}	ES Lhx6-GFP^{-}	MGE Lhx6-GFP^{+}	ES-GFP^{+} vs ES-GFP^{-}	MGE-GFP^{+} vs ES-GFP^{-}	MGE-GFP^{+} vs ES-GFP^{+}
Ventricular Zone						
Hes1	6.54	8.88	6.58	0.20	0.20	1.03
Hes5	11.62	13.39	11.72	0.29	0.31	1.07
Oligodendrocytes						
Olig1	6.29	9.15	5.99	0.14	0.11	0.81
Olig2	8.80	11.63	9.59	0.14	0.24	1.73
PDGFRα	6.55	7.49	5.78	0.52	0.30	0.59
Sox10	6.95	9.96	6.69	0.12	0.10	0.84
Telencephalic						
Arx	13.31	11.99	14.43	2.49	5.42	2.18
Bcl11b (Ctip2)	9.51	7.49	11.02	4.24	14.56	3.09
Cux2	9.11	8.67	9.91	1.38	2.37	1.75
Foxg1	13.75	11.08	14.87	6.35	13.77	2.17
FoxP1	9.21	9.91	8.84	0.62	0.48	0.78
NR2F1 (CoupTF1)	13.23	12.62	12.71	1.52	1.07	0.70
NR2F2 (coupTF2)	11.96	11.60	10.28	1.29	0.40	0.31
Sox6	11.86	9.89	13.21	3.92	10.00	2.55
Zeb2 (Zfhx1b)	7.92	8.30	9.50	0.77	2.31	3.00
Pallial						
Emx1	7.57	6.83	6.11	1.67	0.61	0.36
Neurod1	6.15	7.62	6.42	0.37	0.44	1.22
Neurog2	6.46	9.77	6.22	0.10	0.09	0.85
Pax6	6.38	7.65	5.97	0.43	0.33	0.75
Tbr1	6.74	7.54	9.48	0.58	4.43	7.89
Tbr2 (Eomes)	6.47	7.21	7.03	0.62	0.92	1.48
Subpallial						
Dlx1	14.14	12.14	14.36	4.00	4.67	1.17
Dlx2	11.41	10.06	12.79	2.55	6.64	2.61
Dlx5	10.80	9.46	11.98	2.54	5.74	2.26
Dlx6	12.21	10.32	12.19	3.70	3.67	0.99
GAD1	13.89	11.59	13.28	4.92	3.22	0.65

GAD2	14.07	12.21	13.96	3.63	3.37	0.93
LGE progenitors						
Gsx2	6.17	6.38	6.72	0.86	1.26	1.46
LGE/striatum						
cdh8	8.09	7.69	8.54	1.32	1.81	1.37
Ebf1	8.67	10.35	8.25	0.31	0.23	0.75
Etv1 (ER81)	7.04	8.51	11.12	0.43	6.75	17.20
FoxP1	9.21	9.91	8.84	0.62	0.48	0.78
Islet1	7.12	7.63	7.91	0.70	1.21	1.73
Penk	12.05	13.37	7.96	0.40	0.02	0.06
MGE & CGE progenitors						
Nkx2-1	11.67	10.09	12.94	2.98	7.20	2.41
NR2F1 (dorsal MGE & CGE)	13.23	12.62	12.71	1.52	1.07	0.70
NR2F2 (dorsal MGE & CGE)	11.96	11.60	10.28	1.29	0.40	0.31
MGE subpallial neurons/globus pallidus						
Cdh8	8.09	7.69	8.54	1.32	1.81	1.37
Etv1 (ER81)	7.04	8.51	11.12	0.43	6.75	17.20
Gbx1	8.58	6.95	9.28	3.13	5.81	1.76
Gbx2	8.78	8.27	10.70	1.42	5.40	3.79
Kctd12	9.03	7.80	11.11	2.34	9.94	4.24
Lhx6	13.16	9.20	14.02	15.50	28.20	1.83
Lhx8	11.49	7.55	13.13	15.31	47.56	3.11
LMO3	10.47	9.71	10.68	1.69	1.96	1.16
Nkx2-1	11.67	10.09	12.94	2.98	7.20	2.41
Shh	11.48	11.13	11.08	1.28	0.97	0.76
Sox6	11.86	9.89	13.21	3.92	10.00	2.55
Zic1	10.81	9.58	12.86	2.42	10.87	4.26
MGE interneurons						
Calb1 (Calbindin)	8.18	6.73	8.06	2.76	2.64	1.02
Cux2	9.11	8.67	9.91	1.38	2.37	1.75
Cxcr4	11.74	13.32	11.10	0.33	0.21	0.64
Cxcr7	6.66	6.14	7.29	1.44	2.22	1.55
ErbB4	10.16	8.39	10.13	3.46	3.33	1.01
Lhx6	13.16	9.20	14.02	15.50	28.20	1.83

Lhx8	11.49	7.55	13.13	15.31	47.56	3.11
Maf (cMaf, vMaf)	9.94	8.22	10.29	3.30	4.19	1.27
MafB	11.63	9.68	11.78	3.86	4.28	1.11
Npas1	10.69	7.86	8.31	7.13	1.57	0.22
NPY	15.39	12.80	13.32	5.99	1.43	0.24
Nrxn3	12.28	10.95	12.13	2.55	2.29	0.93
Nxph1	12.78	11.12	12.22	3.16	2.15	0.69
Sst (Somatostatin)	14.22	11.79	13.21	5.39	2.69	0.50
Sox6	11.86	9.89	13.21	3.92	10.00	2.55
**Hypothalamus**						
Nkx2-2	9.24	10.61	6.68	0.44	0.07	0.19
Nr5a1 (Steroidogenic factor 1)	10.76	10.59	10.75	1.13	1.12	0.99
Otp	6.92	7.16	6.84	0.85	0.80	0.95
Rax	9.10	7.21	7.05	3.70	0.89	0.24

Supplementary Table S3 Block A and Block B from the supervised clustering map (Figure 3)

Genes from block A and B of the supervised heatmap (Figure 3) are list below (the order of the genes are the same as in the map (from top to bottom). Many of the genes that regulate and/or mark developing cortical interneurons as shown in Tables 1 and S2 are bold-typed.

Block A Genes:

Probe ID	Gene Symbol	MGE Lhx6-GFP+	ES Lhx6-GFP+	ES Lhx6-GFP-
A_55_P1981604	Olfr670	6.0444	9.2288	6.0839
A_55_P2101137	Olfr1123	5.7726	8.9213	5.8989
A_55_P2050173	LOC676534	6.9523	11.1117	8.5424
**A_55_P2031721**	**Npas1**	**9.1230**	**10.7072**	**7.8918**
A_52_P609695	Lgi1	8.6300	10.1618	7.2683
A_51_P402443	Olfr836	6.5392	8.4798	5.4594
A_55_P1975260	Copg2as2	12.7268	13.9985	11.7174
A_52_P52849	Cpxm2	7.7062	9.0274	6.6842
A_30_P01032598	A_30_P01032598	8.7131	10.0656	7.5999
A_66_P102110	LOC100045717	6.8704	8.1989	6.0732
A_51_P160083	A_51_P160083	8.7211	9.8289	7.6202
A_55_P1973833	Astl	7.4374	8.5925	6.3866

A_55_P1999923	6430598A04Rik	12.6034	13.7696	11.6627
A_55_P2178834	A_55_P2178834	7.1241	8.2815	6.2605
A_55_P2152225	Ihh	7.3531	8.4094	6.3866
A_51_P400217	Vpreb1	8.1514	9.0877	7.0643
A_52_P609695	Lgi1	8.6300	10.1618	7.2683
A_52_P376169	Lypd6	9.8325	10.7585	8.5346
**A_51_P262489**	**Sst**	**13.3488**	**14.3391**	**11.9283**
**A_52_P144310**	**Gad1**	**13.4108**	**14.0227**	**11.7300**
**A_51_P469285**	**Nrp1**	**9.5160**	**10.2473**	**7.9830**
A_52_P71624	Olfr1410	12.3270	12.9115	10.8143
A_66_P125873	Nup93	9.9654	10.5392	8.4818
A_51_P474551	Cyp19a1	8.7330	9.4788	7.3531
**A_55_P2119888**	**Erbb4**	**9.7762**	**10.1885**	**8.1361**
A_55_P2113758	Gria1	9.9154	10.9938	7.7347
A_51_P320357	Grin2b	9.9822	10.6132	8.0325
A_52_P38011	Ralyl	10.1408	10.8027	8.0353

Block B Genes:

ProbeID	GeneSymbol	MGE Lhx6-GFP+	ES Lhx6-GFP+	ES Lhx6-GFP-
A_51_P145948	Neto1	11.3061	10.9922	8.0679
A_55_P1973377	Vstm2a	8.9787	8.9991	6.9944
A_55_P2016114	Fasl	9.0892	9.1624	7.0732
A_52_P335466	Dscaml1	11.7172	11.8987	9.8908
A_55_P2032458	Gm6934	9.0265	9.2416	7.1782
A_55_P2119957	Mlxipl	13.8611	14.1169	11.8121
A_30_P01027135	A_30_P01027135	10.0405	8.1378	7.9317
A_55_P2155620	Mef2c	9.4094	9.1059	7.2256
A_55_P2038101	Dock11	10.2513	10.0300	8.2368
A_55_P2112524	Kcnmb2	8.5372	8.2668	6.4649
A_51_P223724	Khdrbs2	10.3803	10.2384	8.2288
**A_52_P464831**	**Dlx1**	**14.4818**	**14.2616**	**12.2785**
A_51_P126437	Enc1	12.7605	12.5423	10.6330
A_51_P394847	Gm11346	8.3354	8.1766	6.2605
**A_52_P495869**	**Mafb**	**11.9209**	**11.7720**	**9.8235**
**A_51_P405167**	**Maf**	**10.4252**	**10.0802**	**8.3583**

A_30_P01021129	A_30_P01021129	10.2475	8.0444	7.8918
**A_51_P236738**	**Dlx6as**	**12.2603**	**11.8408**	**10.1764**
A_52_P244637	Fam65b	9.9598	9.5549	7.7901
A_66_P137501	Tmem90b	9.3249	8.9059	7.1865
A_55_P2315762	A_55_P2315762	11.6909	11.1866	9.2336
A_52_P399990	B4galt2	10.8031	10.4487	8.3859
A_55_P2157794	9430031J16Rik	9.0688	8.1931	6.4838
A_51_P228817	A_51_P228817	10.7595	10.0078	8.1540
A_55_P2063505	Gm4235	13.1017	12.7583	10.0501
A_51_P145948	Neto1	11.3061	10.9922	8.0679
A_55_P2061119	Gm8570	14.7600	14.8825	11.9137
A_55_P2157794	9430031J16Rik	9.0688	8.1931	6.4838
A_55_P2020612	Arl4d	11.2669	10.6649	8.4009
A_55_P2054967	A_55_P2054967	8.8714	8.6760	6.2095
A_52_P432969	Pcdh19	11.4341	11.1262	8.7571
A_55_P2177406	Gm2813	8.5071	8.2328	5.9464
A_55_P2120141	Gm4522	14.0782	13.7493	11.2591
A_51_P105068	Lypd6b	9.8320	9.6569	7.0008

**A_66_P119376**	**Kctd12**	**11.2545**	**9.1641**	**7.9386**
A_55_P1974845	Pde1a	10.8267	8.9134	7.6558
A_55_P2004442	Nr2e1	9.7944	8.0353	6.4972
A_52_P22763	Mtap2	13.6213	11.9944	10.6598
A_55_P1988872	A_55_P1988872	13.4339	11.8536	10.4371
A_52_P502228	Zic4	9.8542	8.1649	6.7504
A_55_P2020612	Arl4d	11.2669	10.6649	8.4009
**A_52_P112463**	**Dlx6os1**	**11.3515**	**10.0573**	**8.3671**
A_51_P279100	Ptgs1	8.9873	7.6257	5.9149
**A_52_P475356**	**Sox6**	**13.3468**	**11.9988**	**10.0307**
A_55_P2024888	Ctss	8.5999	7.4136	5.4594
**A_55_P2051787**	**Gbx1**	**9.3495**	**8.0728**	**6.1964**
A_66_P126459	Gm13429	9.8875	8.8523	6.9029
A_55_P1979873	Phactr3	9.3465	8.0980	7.2288
NA	NA	NA	NA	NA
A_51_P432950	Sstr1	9.0782	7.2127	6.6912
A_55_P1990200	Gm1568	12.7829	11.4616	10.5803
A_55_P1973339	Mapk1	10.7293	9.4105	8.5482

A_30_P01022232	A_30_P01022232	8.7906	7.4703	6.6606
A_55_P1963980	Lrrc16a	10.9709	9.6187	8.9071
A_55_P2081178	Msl3l2	9.5071	8.1590	7.4717
A_52_P209944	A_52_P209944	12.6260	11.2714	10.5786
A_66_P120770	Ywhaz	14.3250	12.9554	12.2921
A_52_P374897	Arg2	8.7715	7.3823	6.7051
A_52_P395280	Lrrc55	8.5281	7.0928	6.4512
A_51_P315042	Avpr1a	8.7016	7.3007	6.5443
A_51_P191726	Efcab6	8.1137	6.7142	5.9696
A_52_P502267	Epc2	11.3289	9.9669	9.2046
A_52_P612137	Runx1t1	14.4972	13.0676	12.4899
A_55_P2036007	Rai2	12.7359	11.3009	10.7188
A_55_P2053132	A_55_P2053132	8.4374	6.9600	6.4179
A_30_P01030456	A_30_P01030456	7.6558	9.7922	7.4037
A_55_P1984178	Pde4dip	10.2414	8.6677	8.1972
A_55_P1991160	Mrrf	10.2619	8.7217	8.1514
A_52_P450934	Paqr9	9.8502	8.3450	7.6993
A_55_P1984178	Pde4dip	10.2414	8.6677	8.1972

A_30_P01033342	A_30_P01033342	7.7682	9.7786	7.7257
A_55_P2090429	Dync1i1	12.5939	11.1812	10.3276
A_52_P160005	Pcyt1b	10.8256	9.4788	8.5507
A_52_P244132	Isoc1	11.6968	10.3593	9.3907
A_55_P2170605	A_55_P2170605	8.5085	7.1782	6.1632
A_30_P01027392	A_30_P01027392	6.8308	8.5209	6.4838
A_52_P39157	Bmi1	11.3540	10.0625	9.0638
A_55_P2392485	9330159F19Rik	11.6409	10.3519	9.2594
A_66_P120555	Grik3	11.5936	10.2804	9.1662
A_51_P183283	Aff2	11.8028	10.5456	9.4730
A_55_P1961320	Tes	11.0802	9.9328	8.8424
A_30_P01031530	A_30_P01031530	6.7966	9.0989	6.4346
A_55_P2049373	Pde3a	9.1712	7.7302	6.6342
A_52_P106789	Rbm18	15.2482	13.5194	13.0616
A_55_P1953753	Cnr1	11.4495	9.7687	9.3266
A_55_P2095488	Syncrip	11.4439	9.7756	9.3313
A_66_P114461	Grm7	10.5975	8.9302	8.4471
A_30_P01026130	A_30_P01026130	6.6606	8.7102	6.0371

A_55_P2071384	Crh	8.4416	6.6558	6.2854
A_55_P2077914	5730494M16Rik	9.6046	7.7302	7.4094
A_55_P1965939	A_55_P1965939	8.9773	7.1782	6.6390
A_55_P2034968	A_55_P2034968	9.4865	7.7279	7.1699
A_51_P432950	Sstr1	9.0782	7.2127	6.6912
A_30_P01017450	A_30_P01017450	5.9069	8.2574	5.8497
A_55_P1991160	Mrrf	10.2619	8.7217	8.1514
A_66_P139946	E030019B13Rik	11.6943	9.8499	9.3661
A_55_P2261772	C230098O21Rik	10.7790	8.9089	8.4485
A_55_P1965694	Gabrg2	12.0781	9.8727	9.1374
**A_51_P179578**	**Gbx2**	**10.8437**	**8.9174**	**8.4115**
A_52_P250555	Dynll1	10.6207	9.0398	8.1137
A_52_P114260	C1rb	10.3554	8.7638	7.9199
**A_51_P194230**	**Zic1**	**13.8774**	**11.5037**	**9.9006**
A_30_P01031788	A_30_P01031788	9.6126	6.9542	6.4730
A_51_P204053	Hba-x	8.1207	6.5288	5.7726
A_52_P502228	Zic4	9.8542	8.1649	6.7504
A_55_P2090429	Dync1i1	12.5939	11.1812	10.3276

A_55_P2038540	Hbb-b2	8.0498	6.3634	5.6196
A_55_P2036999	Zic3	10.9726	9.2029	8.5301
A_55_P2096023	Gm2490	11.6182	9.8132	8.7808
A_30_P01032159	A_30_P01032159	9.3740	7.6172	6.6534
A_55_P2130660	Slitrk4	9.8017	8.0224	7.0407
A_51_P218774	Rgs10	12.6209	10.7749	9.9998
A_51_P229602	Plxna2	13.7306	11.8454	11.0538
A_55_P1961152	Pou3f1	11.0859	9.2167	8.3597
**A_51_P480202**	**Dlx2**	**12.9321**	**11.5535**	**10.2036**
A_52_P843948	Gm9804	9.1068	7.7279	6.4179
A_51_P507333	Kcnc2	9.3283	7.9109	6.6653
A_52_P161630	St6gal1	11.1220	9.7841	8.3339
**A_52_P216427**	**Nkx2-1**	**13.0755**	**11.8058**	**10.2306**
A_55_P2077483	Adarb2	11.5513	10.6657	9.1211
A_55_P2199737	9030205G03Rik	9.1809	8.2931	6.7616
A_30_P01023611	A_30_P01023611	9.0611	8.1874	6.6269
A_51_P171772	Bcl11b	9.5380	8.6408	7.0334
A_55_P1958275	Bcl11a	12.4005	11.2803	9.7830

A_55_P2033480	Gm13298	10.2660	9.1762	7.6958
**A_51_P261051**	**Dlx5**	**12.1193**	**10.9429**	**9.5949**
A_52_P554703	Gprin3	10.5776	9.3779	8.1361
A_55_P1987430	Fam196a	9.4932	8.4311	7.0643
**A_55_P2057587**	**Arx**	**14.5528**	**13.4424**	**12.1349**
A_66_P130601	Foxp2	9.1762	8.2005	6.8538
A_55_P1999945	Dlgap2	10.0471	9.0503	7.7682
A_52_P220810	Trib2	12.4663	11.4332	10.1304
A_55_P2176076	Lrrc7	10.5579	9.8981	8.3823
A_52_P6057	Chst2	9.9794	9.4016	7.7504
A_55_P2052006	Nol4	12.5701	12.0119	10.4903
A_55_P2176898	Gm2185	8.2368	7.7222	6.1964
A_51_P461031	Ostm1	10.0846	9.3417	7.9744
A_51_P513661	Slc4a8	12.4039	11.7047	10.3127
A_51_P358894	Ttc9b	10.2591	9.5430	8.1649
A_52_P164286	Dnm3	9.7861	9.1048	7.7504
A_52_P27122	Eif2c4	9.9828	9.3272	7.9238
A_52_P481423	Cttnbp2nl	11.5454	10.7951	9.3797

A_30_P01026072	A_30_P01026072	10.4108	8.3678	8.0047
A_52_P589568	Foxo6	11.9888	10.8328	9.9837
A_55_P2053374	Sorbs1	12.0593	11.2234	10.0379
A_51_P303868	Klhl29	10.0203	9.1442	7.9962
A_51_P295237	Lrp11	12.4521	11.5265	10.4350
A_55_P2148574	LOC100047593	10.4910	9.5017	8.4291
A_51_P423709	Fam84a	11.5311	10.5347	9.3216
A_51_P267494	Cdc42ep3	11.1259	10.1308	8.9586
A_52_P285024	Sertad4	10.6181	9.6878	8.4374
A_30_P01029070	A_30_P01029070	5.6582	8.2127	5.6390
A_51_P291078	Sel1l3	11.5097	10.5907	9.3506
A_55_P1985410	Reps2	9.1068	8.1649	7.0224
A_55_P2102260	Has3	10.0085	9.1155	7.9099
A_66_P136097	Trim13	10.6730	9.7364	8.5463
**A_55_P2094158**	**Lhx6**	**14.6550**	**13.9569**	**9.9307**
**A_51_P298615**	**Lhx8**	**13.2640**	**11.6320**	**7.6958**
**A_55_P2053127**	**Foxg1**	**14.9794**	**13.8841**	**11.2244**
A_51_P171772	Bcl11b	9.5380	8.6408	7.0334

A_55_P1954693	Tac1	11.8150	9.7588	7.4811

Supplementary Table S4: Cell counts from 69 days after transplantation

Four transplants (mice that received Lhx6-GFP+ cells and had at least 10 Lhx6-GFP+ cells in the cortex: N1, R3, R5 and R6) were examined for co-labeling of Lhx6-GFP with parvalbumin (PV), somatostatin (SOM), and neuropeptide Y (NPY). Total numbers, average, and standard errors from 3-4 transplants were shown.

PV/GFP	animal	cortex GFP+	GFP+/PV+	% of colabeling
	N1	68	23	33.82%
	R6	17	3	17.65%
	R5	26	7	26.92%
	R3	9	1	11.11%
	Total	120	34	28.33%
	Average			22.38%
	SE			5.01%

SOM/GFP	animal	cortex GFP+	GFP+/SOM+	% of colabeling
	N1	46	36	78.26%
	R6	7	4	57.14%
	R5	26	10	38.46%
	R3	0	0	NA
	Total	79	50	63.29%
	Average			57.96%
	SE			11.50%

NPY/GFP	animal	cortex GFP+	GFP+/NPY+	% of colabeling
	N1	55	17	30.91%
	R6	15	0	0.00%
	R5	35	7	20.00%
	R3	9	1	11.11%
	Total	114	25	21.93%
	Average			15.51%
	SE			6.57%

Supplementary Table S5: Comparison of enhancer activities in transgenic embryos and differentiated ES cells

	Transgenic Expression		Differentiated mES cell expression	
**Enhancer ID**	**VZ & SVZ**	**MZ**	**mKi67^{+}**	**mKi67^{-}**
DlxI12b	No	Yes	No	Yes
692	Yes	Yes	No	Yes
1056	Yes	No	Few	Yes
1538	Yes	Yes	Few	Yes

Table S6

ES Lhx6-GFP+ vs ES Lhx6-GFP-: Fold Change>= 4

No	Probe ID	Accession	Gene Symbol	Description	Genomic Coordinates	ES Lhx6-GFP+	ES Lhx6-GFP-	Fold Change
1	A_55_P2094158	NM_008500	Lhx6	Mus musculus LIM homeobox protein 6 (Lhx6), transcript variant 1, mRNA [NM_008500]	chr2:35937529-35937470	13.82	9.79	16.38
2	A_51_P298615	NM_010713	Lhx8	Mus musculus LIM homeobox protein 8 (Lhx8), mRNA [NM_010713]	chr3:153969436-153969377	11.49	7.56	15.31
3	A_55_P2094149	NM_001083125	Lhx6	Mus musculus LIM homeobox protein 6 (Lhx6), transcript variant 2, mRNA [NM_001083125]	chr2:35958438-35958379	12.49	8.62	14.63
4	A_51_P261569	NM_198674	Fbxw26	Mus musculus F-box and WD-40 domain protein 26 (Fbxw26), mRNA [NM_198674]	chr9:109624730-109624671	9.64	5.90	13.33
5	A_55_P2328082	AK142074	AI606473	Mus musculus 12 days embryo eyeball cDNA, RIKEN full-length enriched library, clone:D230021C23 product:unclassifiable, full insert sequence. [AK142074]	chr3:153997592-153997651	10.85	7.13	13.16
6	A_55_P1971398	XM_980161	Rpl17-ps2	PREDICTED: Mus musculus similar to Ribosomal protein L17 (LOC667688), mRNA [XM_980161]	chr12:96929241-96929300	9.70	6.04	12.68
7	A_55_P2094484	NM_001039223	Gm14137	Mus musculus predicted gene 14137 (Gm14137), mRNA [NM_001039223]	chr2:119002407-119002466	9.75	6.29	11.06
8	A_52_P515769	NM_017378	Pcdh12	Mus musculus protocadherin 12 (Pcdh12), mRNA [NM_017378]	chr18:38426927-38426868	8.84	5.55	9.77
9	A_55_P2113758	NM_001113325	Gria1	Mus musculus glutamate receptor, ionotropic, AMPA1 (alpha 1) (Gria1), transcript variant 1, mRNA [NM_001113325]	chr11:57143613-57143672	10.85	7.59	9.57
10	A_55_P1981604	NM_207146	Olfr670	Mus musculus olfactory receptor 670 (Olfr670), mRNA [NM_207146]	chr7:112108365-112108306	9.09	5.99	8.60
11	A_55_P2151950	NM_146763	Olfr1406	Mus musculus olfactory receptor 1406 (Olfr1406), mRNA [NM_146763]	chr1:175113999-175113940	8.51	5.44	8.40
12	A_55_P2061119	XM_001003594	Gm8570	PREDICTED: Mus musculus predicted gene, EG667314 (EG667314), mRNA [XM_001003594]	chr10:92660479-92660420	14.76	11.77	7.94
13	A_55_P2101137	NM_146350	Olfr1123	Mus musculus olfactory receptor 1123 (Olfr1123), mRNA [NM_146350]	chr2:87259119-87259178	8.78	5.81	7.83
14	A_51_P436068	NM_007412	Gpr182	Mus musculus G protein-coupled receptor 182 (Gpr182), mRNA [NM_007412]	chr10:127186816-127186757	9.51	6.56	7.72
15	A_51_P402443	NM_146564	Olfr836	Mus musculus olfactory receptor 836 (Olfr836), mRNA [NM_146564]	chr9:18926286-18926345	8.34	5.40	7.66
16	A_51_P145948	NM_144946	Neto1	Mus musculus neuropilin (NRP) and tolloid (TLL)-like 1 (Neto1), mRNA [NM_144946]	chr18:86670386-86670445	10.85	7.93	7.59
17	A_30_P01028270	chr15:3946037-3972747_F		lincRNA:chr15:3946037-3972747 forward strand	chr15:3946069-3946128	9.87	6.95	7.58
18	A_52_P609695	NM_020278	Lgi1	Mus musculus leucine-rich repeat LGI family, member 1 (Lgi1), mRNA [NM_020278]	chr19:38382319-38382378	10.02	7.13	7.42

19	A_55_P2150966	NM_008718	Npas1	Mus musculus neuronal PAS domain protein 1 (Npas1), mRNA [NM_008718]	chr7:17046739-17046680	10.82	7.97	7.23
20	A_55_P2021089	XM_001472541	Ighg	PREDICTED: Mus musculus similar to immunoglobulin gamma 2A chain (LOC100048855), mRNA [XM_001472541]		8.61	5.77	7.17
21	A_30_P01031637	chr1:183951156-183986156_F		lincRNA:chr1:183951156-183986156 forward strand	chr1:183958368-183958427	8.91	6.08	7.12
22	A_55_P2286493	AK016664	4933405E24Rik	Mus musculus adult male testis cDNA, RIKEN full-length enriched library, clone:4933405E24 product:unclassifiable, full insert sequence. [AK016664]	chr11:53991350-53991291	14.35	11.52	7.11
23	A_51_P449638	NR_033616	Pldi	Mus musculus polymorphic derived intron containing (Pldi), non-coding RNA [NR_033616]	chr10:60391434-60391375	9.14	6.31	7.10
24	A_51_P176086	NM_146187	Ffar2	Mus musculus free fatty acid receptor 2 (Ffar2), transcript variant 1, mRNA [NM_146187]	chr7:31604208-31604149	10.38	7.56	7.05
25	A_55_P2031721	NM_008718	Npas1	Mus musculus neuronal PAS domain protein 1 (Npas1), mRNA [NM_008718]	chr7:17041158-17041099	10.57	7.75	7.04
26	A_30_P01026521	chr17:7838192-7853103_F		lincRNA:chr17:7838192-7853103 forward strand	chr17:7853044-7853103	8.36	5.55	6.99
27	A_30_P01024857	chr18:5951953-5952386_F		lincRNA:chr18:5951953-5952386 forward strand	chr18:5951997-5952056	9.89	7.12	6.81
28	A_52_P38011	NM_178631	Ralyl	Mus musculus RALY RNA binding protein-like (Ralyl), transcript variant 1, mRNA [NM_178631]	chr3:14181384-14181443	10.66	7.90	6.80
29	A_51_P151126	NM_013706	Cd52	Mus musculus CD52 antigen (Cd52), mRNA [NM_013706]	chr4:133649564-133649505	8.75	6.02	6.62
30	A_55_P2126072	XM_001472689	Gm2160	PREDICTED: Mus musculus similar to SPPL3 protein (LOC100039327), mRNA [XM_001472689]	chr6:35882916-35882975	7.92	5.20	6.58
31	A_55_P2063505	XM_001479508	Gm4235	PREDICTED: Mus musculus hypothetical protein LOC100043109 (LOC100043109), mRNA [XM_001479508]	chr12:33682931-33682872	12.62	9.91	6.57
32	A_30_P01018620	chr17:39980973-39981876_R		lincRNA:chr17:39980973-39981876 reverse strand	chr17:39981446-39981387	13.15	10.43	6.56
33	A_55_P2104312	NM_013618	Olfr66	Mus musculus olfactory receptor 66 (Olfr66), mRNA [NM_013618]	chr7:111029879-111029820	8.80	6.09	6.52
34	A_51_P375754	NM_010462	Hoxc10	Mus musculus homeobox C10 (Hoxc10), mRNA [NM_010462]	chr15:102801857-102801916	9.02	6.33	6.44
35	A_66_P118212	NM_001011832	Olfr1490	Mus musculus olfactory receptor 1490 (Olfr1490), mRNA [NM_001011832]	chr19:13729818-13729877	8.38	5.70	6.44
36	A_55_P1967733	NM_001033276	Mll2	Mus musculus myeloid/lymphoid or mixed-lineage leukemia 2 (Mll2), mRNA [NM_001033276]	chr15:98663049-98662990	11.16	8.47	6.44
37	A_55_P2053127	NM_001160112	Foxg1	Mus musculus forkhead box G1 (Foxg1), transcript variant 2, mRNA [NM_001160112]	chr12:50487766-50487825	13.75	11.08	6.35
38	A_51_P105068	NM_027990	Lypd6b	Mus musculus LY6/PLAUR domain containing 6B (Lypd6b), mRNA [NM_027990]	chr2:49804191-49804250	9.52	6.87	6.29
39	A_30_P01031530	chr9:96664617-96683617_R		lincRNA:chr9:96664617-96683617 reverse strand	chr9:96676202-96676143	8.96	6.32	6.26

40	A_30_P01026130	chr17:32075308-32075792_R		lincRNA:chr17:32075308-32075792 reverse strand	chr17:32075532-32075473	8.57	5.94	6.18
41	A_30_P01021775	chr17:39960567-39992817_F		lincRNA:chr17:39960567-39992817 forward strand	chr17:39982106-39982165	16.14	13.53	6.12
42	A_55_P1992218	XM_001476624	LOC100046688	PREDICTED: Mus musculus hypothetical protein LOC100046688 (LOC100046688), mRNA [XM_001476624]	chrX:75111329-75111388	9.23	6.61	6.11
43	A_55_P2019799	NM_146543	Olfr1360	Mus musculus olfactory receptor 1360 (Olfr1360), mRNA [NM_146543]	chr13:21765929-21765870	8.85	6.24	6.10
44	A_30_P01019562	chr10:116814196-116920696_F		lincRNA:chr10:116814196-116920696 forward strand	chr10:116846961-116847020	8.26	5.66	6.05
45	A_30_P01029897	chr9:43738583-43757007_F		lincRNA:chr9:43738583-43757007 forward strand	chr9:43747333-43747392	9.38	6.78	6.05
46	A_55_P2373774	AK021026	B430319H21Rik	Mus musculus 4 days neonate male adipose cDNA, RIKEN full-length enriched library, clone:B430319H21 product:hypothetical protein, full insert sequence. [AK021026]	chr11:63001848-63001907	9.31	6.72	6.04
47	A_51_P454873	NM_023456	Npy	Mus musculus neuropeptide Y (Npy), mRNA [NM_023456]	chr6:49773743-49777505	15.39	12.80	5.99
48	A_51_P320357	NM_008171	Grin2b	Mus musculus glutamate receptor, ionotropic, NMDA2B (epsilon 2) (Grin2b), mRNA [NM_008171]	chr6:135680125-135680066	10.47	7.89	5.98
49	A_30_P01026942	chr9:30805020-30810645_R		lincRNA:chr9:30805020-30810645 reverse strand	chr9:30805314-30805255	7.97	5.40	5.94
50	A_55_P2050173	XM_990857	LOC676534	PREDICTED: Mus musculus similar to ITGBL1 protein (LOC676534), mRNA [XM_990857]	chr4:120537534-120537593	10.97	8.40	5.93
51	A_55_P2075510	ENSMUST00000103508		Mus musculus partial mRNA for immunoglobulin heavy chain variable region (IGHV gene), clone 3. [FN652764]	chr12:116002805-116002746	8.34	5.77	5.93
52	A_30_P01019652	chr1:92412246-92423471_F		lincRNA:chr1:92412246-92423471 forward strand	chr1:92419303-92419362	8.99	6.43	5.90
53	A_52_P120132	ENSMUST00000066875		RIKEN cDNA 6030452D12 gene Gene [Source:MGI Symbol;Acc:MGI:3045356] [ENSMUST00000066875]	chr8:109032939-109032998	8.33	5.77	5.90
54	A_52_P203770	NM_001105058	Vmn2r61	Mus musculus vomeronasal 2, receptor 61 (Vmn2r61), mRNA [NM_001105058]	chr7:49555654-49555713	7.87	5.32	5.85
55	A_55_P1993213	NM_146860	Olfr161	Mus musculus olfactory receptor 161 (Olfr161), mRNA [NM_146860]	chr16:3593280-3593339	8.52	5.98	5.83
56	A_55_P2141256	XM_001474975	Gm1758	PREDICTED: Mus musculus gene model 1758, (NCBI) (Gm1758), mRNA [XM_001474975]	chr16:14509296-14509355	8.90	6.36	5.81
57	A_52_P334562	NM_009504	Vdr	Mus musculus vitamin D receptor (Vdr), mRNA [NM_009504]	chr15:97685195-97685136	8.30	5.77	5.80
58	A_30_P01032464	chr10:69667411-69678033_F		lincRNA:chr10:69667411-69678033 forward strand	chr10:69677914-69677973	8.72	6.19	5.78
59	A_55_P2099565	ENSMUST00000084758		olfactory receptor 471 Gene [Source:MGI Symbol;Acc:MGI:3030305] [ENSMUST00000084758]	chr7:115004629-115004570	8.50	5.98	5.75
60	A_52_P265979	NM_029702	Arfrp1	Mus musculus ADP-ribosylation factor related protein 1 (Arfrp1), transcript variant 2, mRNA [NM_029702]	chr2:181093013-181092954	10.03	7.51	5.74

61	A_30_P01033185	chr4:88604248-88645948_R		lincRNA:chr4:88604248-88645948 reverse strand	chr4:88641776-88641717	8.85	6.33	5.72
62	A_55_P2279927	AK148051	5930430L01Rik	Mus musculus B16 F10Y cells cDNA, RIKEN full-length enriched library, clone:G370004A16 product:unclassifiable, full insert sequence. [AK148051]	chr5:149801708-149801649	8.53	6.03	5.67
63	A_55_P2120141	XM_001480410	Gm4522	PREDICTED: Mus musculus similar to reproductive homeobox on X chromosome 8 (LOC100043565), mRNA [XM_001480410]	chr6:126244172-126244231	13.62	11.12	5.66
64	A_30_P01029070	chr18:35204950-35216650_F		lincRNA:chr18:35204950-35216650 forward strand	chr18:35213972-35214031	8.07	5.57	5.65
65	A_55_P1999888	NM_011998	Chst4	Mus musculus carbohydrate (chondroitin 6/keratan) sulfotransferase 4 (Chst4), mRNA [NM_011998]	chr8:112553204-112553145	9.70	7.21	5.61
66	A_51_P486217	NM_175638	Wnk4	Mus musculus WNK lysine deficient protein kinase 4 (Wnk4), mRNA [NM_175638]	chr11:101138198-101138364	9.56	7.07	5.59
67	A_30_P01032598	chr14:28231577-28242027_F		lincRNA:chr14:28231577-28242027 forward strand	chr14:28238826-28238885	9.92	7.46	5.52
68	A_30_P01033301	chr12:111322030-111323387_F		lincRNA:chr12:111322030-111323387 forward strand	chr12:111322843-111322902	7.99	5.52	5.52
69	A_51_P230298	NM_008232	Hdgfl1	Mus musculus hepatoma derived growth factor-like 1 (Hdgfl1), mRNA [NM_008232]	chr13:26860852-26860793	8.92	6.47	5.46
70	A_52_P925277	NM_001079883	Bcl11b	Mus musculus B-cell leukemia/lymphoma 11B (Bcl11b), transcript variant 1, mRNA [NM_001079883]	chr12:109148991-109148932	10.53	8.09	5.44
71	A_55_P2054967	ENSMUST00000053171		leucine rich repeat containing 49 Gene [Source:MGI (curated);Acc:MGI:2442689] [ENSMUST00000053171]	chr9:60417103-60417044	8.53	6.10	5.39
72	A_51_P262489	NM_009215	Sst	Mus musculus somatostatin (Sst), mRNA [NM_009215]	chr16:23889835-23889776	14.22	11.79	5.39
73	A_52_P540350	ENSMUST00000118300		Novel protein similar to testis derived transcript (Tes)MCG8129 ; [Source:UniProtKB/TrEMBL;Acc:A2ALC6] [ENSMUST00000118300]	chrX:23535114-23535055	8.29	5.86	5.38
74	A_52_P175634	NM_001077424	A4gnt	Mus musculus alpha-1,4-N-acetylglucosaminyltransferase (A4gnt), mRNA [NM_001077424]	chr9:99521093-99521152	8.85	6.42	5.37
75	A_30_P01019146	chr2:128211990-128215223_R		lincRNA:chr2:128211990-128215223 reverse strand	chr2:128212284-128212225	8.93	6.50	5.37
76	A_55_P2304010	BG083149	AU015230	H3085A07-5 NIA Mouse 15K cDNA Clone Set Mus musculus cDNA clone H3085A07 5', mRNA sequence [BG083149]	chr17:62871151-62871092	8.62	6.20	5.36
77	A_55_P2241299	NM_001025613	Otud7b	Mus musculus OTU domain containing 7B (Otud7b), transcript variant 1, mRNA [NM_001025613]	chr3:95962836-95962895	9.37	6.95	5.35

78	A_55_P2187899	AK015708	4930505N22Rik	Mus musculus adult male testis cDNA, RIKEN full-length enriched library, clone:4930505N22 product:hypothetical protein, full insert sequence. [AK015708]	chr19:46087803-46087862	9.53	7.11	5.35
79	A_55_P2406802	AK015759	4930511J24Rik	Mus musculus adult male testis cDNA, RIKEN full-length enriched library, clone:4930511J24 product:unclassifiable, full insert sequence. [AK015759]	chr12:111151646-111151705	7.57	5.16	5.31
80	A_55_P2340820	BY714998	4930452L12Rik	BY714998 RIKEN full-length enriched, adult male testis Mus musculus cDNA clone 4930452L12 5'. [BY714998]	chr10:56156080-56156021	9.54	7.14	5.27
81	A_52_P235880	NM_198635	Gm5134	Mus musculus predicted gene 5134 (Gm5134), mRNA [NM_198635]	chr10:75471327-75471386	8.68	6.29	5.27
82	A_55_P2031317	NM_146945	Olfr345	Mus musculus olfactory receptor 345 (Olfr345), mRNA [NM_146945]	chr2:36496437-36496496	8.33	5.93	5.26
83	A_55_P2146837	NM_146925	Olfr481	Mus musculus olfactory receptor 481 (Olfr481), mRNA [NM_146925]	chr7:115225189-115225248	9.05	6.66	5.25
84	A_30_P01030456	chr6:131263850-131314850_F		lincRNA:chr6:131263850-131314850 forward strand	chr6:131272426-131272485	9.65	7.26	5.24
85	A_51_P408227	NM_030218	9130017N09Rik	Mus musculus RIKEN cDNA 9130017N09 gene (9130017N09Rik), mRNA [NM_030218]	chr10:79290730-79292170	9.43	7.04	5.22
86	A_55_P1957363	XM_001472920	Gm2249	PREDICTED: Mus musculus similar to mucin (LOC100039458), mRNA [XM_001472920]	chr18:81886366-81886307	9.50	7.12	5.22
87	A_52_P432969	NM_001105245	Pcdh19	Mus musculus protocadherin 19 (Pcdh19), transcript variant 1, mRNA [NM_001105245]	chrX:130122415-130122356	10.98	8.62	5.16
88	A_55_P1999110	NM_010031	Defa1	Mus musculus defensin, alpha 1 (Defa1), mRNA [NM_010031]		8.12	5.76	5.16
89	A_30_P01028264	chr3:93159107-93159613_F		lincRNA:chr3:93159107-93159613 forward strand	chr3:93159438-93159497	8.99	6.63	5.16
90	A_55_P2002998	NM_145921	Olah	Mus musculus oleoyl-ACP hydrolase (Olah), mRNA [NM_145921]	chr2:3259348-3259289	9.27	6.90	5.16
91	A_55_P2180504	NM_027697	4933413G19Rik	Mus musculus RIKEN cDNA 4933413G19 gene (4933413G19Rik), mRNA [NM_027697]	chr6:128334947-128335006	8.44	6.08	5.15
92	A_30_P01028725	chr14:25924039-25924372_F		lincRNA:chr14:25924039-25924372 forward strand	chr14:25924056-25924115	8.50	6.14	5.15
93	A_30_P01020592	chrX:123306246-123307974_F		lincRNA:chrX:123306246-123307974 forward strand	chrX:123307746-123307805	8.06	5.70	5.14
94	A_30_P01024241	chr7:133402766-133415516_F		lincRNA:chr7:133402766-133415516 forward strand	chr7:133403902-133403961	8.28	5.92	5.14
95	A_30_P01019187	chr11:22510175-22522025_F		lincRNA:chr11:22510175-22522025 forward strand	chr11:22518673-22518732	8.75	6.39	5.13
96	A_55_P2048270	XM_001478335	LOC100047523	PREDICTED: Mus musculus hypothetical protein LOC100047523 (LOC100047523), mRNA [XM_001478335]	chr11:88964796-88964855	8.82	6.47	5.11
97	A_30_P01017450	chr1:94077221-94091396_F		lincRNA:chr1:94077221-94091396 forward strand	chr1:94091065-94091124	8.12	5.77	5.09
98	A_55_P2415387	NM_026668	Lrriq4	Mus musculus leucine-rich repeats and IQ motif containing 4 (Lrriq4), mRNA [NM_026668]	chr3:30571162-30571221	8.88	6.54	5.07

99	A_55_P2005307	NM_010695	Lcn4	Mus musculus lipocalin 4 (Lcn4), mRNA [NM_010695]	chr2:26524927-26524868	7.96	5.62	5.07
100	A_52_P52849	NM_018867	Cpxm2	Mus musculus carboxypeptidase X 2 (M14 family) (Cpxm2), mRNA [NM_018867]	chr7:139234732-139234673	8.89	6.56	5.04
101	A_66_P103124	ENSMUST00000103482		predicted gene 7175 Gene [Source:MGI Symbol;Acc:MGI:3646379] [ENSMUST00000103482]	chr12:115538364-115538305	9.15	6.83	5.00
102	A_52_P373694	NM_177049	Jph4	Mus musculus junctophilin 4 (Jph4), transcript variant a, mRNA [NM_177049]	chr14:55726051-55725992	12.29	9.97	4.99
103	A_55_P2121735	XM_975980	Gm7578	PREDICTED: Mus musculus predicted gene, EG665304 (EG665304), mRNA [XM_975980]	chr7:74573986-74573927	8.66	6.34	4.99
104	A_52_P298394	NM_020278	Lgi1	Mus musculus leucine-rich repeat LGI family, member 1 (Lgi1), mRNA [NM_020278]	chr19:38380730-38380789	11.13	8.81	4.98
105	A_51_P194503	NM_146717	Olfr433	Mus musculus olfactory receptor 433 (Olfr433), mRNA [NM_146717]	chr1:175972960-175973019	8.99	6.68	4.97
106	A_55_P2119957	NM_021455	Mlxipl	Mus musculus MLX interacting protein-like (Mlxipl), mRNA [NM_021455]	chr5:135613705-135613764	13.98	11.67	4.96
107	A_55_P2001324	NM_146855	Olfr985	Mus musculus olfactory receptor 985 (Olfr985), mRNA [NM_146855]	chr9:39934668-39934609	8.29	5.98	4.96
108	A_30_P01030847	chr1:94655789-94657057_R		lincRNA:chr1:94655789-94657057 reverse strand	chr1:94655909-94655850	9.19	6.89	4.95
109	A_55_P2408651	AK051100	4933439C10Rik	Mus musculus 9 days embryo whole body cDNA, RIKEN full-length enriched library, clone:D030071E17 product:unclassifiable, full insert sequence. [AK051100]	chr11:59322945-59323004	9.45	7.14	4.94
110	A_55_P2374337	AK038155	A130071D04Rik	Mus musculus 16 days neonate thymus cDNA, RIKEN full-length enriched library, clone:A130082N01 product:unclassifiable, full insert sequence. [AK038155]	chr1:140058573-140058514	8.57	6.27	4.93
111	A_52_P144310	NM_008077	Gad1	Mus musculus glutamic acid decarboxylase 1 (Gad1), mRNA [NM_008077]	chr2:70439699-70439758	13.89	11.59	4.92
112	A_66_P111871	XM_001477068	Gm3336	PREDICTED: Mus musculus similar to mucin 2 precursor (LOC100046923), mRNA [XM_001477068]	chr8:73246088-73246147	9.33	7.03	4.92
113	A_30_P01031633	chr10:120058696-120076196_R		lincRNA:chr10:120058696-120076196 reverse strand	chr10:120069221-120069162	7.62	5.32	4.91
114	A_55_P2254769	AK137666	4931402G19Rik	Mus musculus adult female vagina cDNA, RIKEN full-length enriched library, clone:9930017J12 product:unclassifiable, full insert sequence. [AK137666]	chr2:120298106-120298165	9.64	7.35	4.88
115	A_30_P01024971	chr6:118250184-118258737_R		lincRNA:chr6:118250184-118258737 reverse strand	chr6:118250244-118250185	8.40	6.11	4.88
116	A_55_P1975260	NR_002845	Copg2as2	Mus musculus coatomer protein complex, subunit gamma 2, antisense 2 (Copg2as2), non-coding RNA [NR_002845]		13.86	11.58	4.88

117	A_55_P1954693	NM_009311	Tac1	Mus musculus tachykinin 1 (Tac1), mRNA [NM_009311]	chr6:7512893-7512952	9.62	7.34	4.85
118	A_55_P1994812	NM_001163503	2010001E11Rik	Mus musculus RIKEN cDNA 2010001E11 gene (2010001E11Rik), mRNA [NM_001163503]	chr10:39640282-39640223	8.66	6.39	4.84
119	A_55_P1987326	NM_025896	Prl3a1	Mus musculus prolactin family 3, subfamily a, member 1 (Prl3a1), mRNA [NM_025896]	chr13:27368470-27368529	9.69	7.41	4.84
120	A_55_P2016888	ENSMUST00000105884			chr4:133614852-133614911	8.35	6.08	4.83
121	A_55_P2120566	XM_001474650	LOC100045635	PREDICTED: Mus musculus similar to Ubtf protein (LOC100045635), mRNA [XM_001474650]	chr18:68117608-68117549	8.07	5.80	4.83
122	A_30_P01033311	chr1:183951156-183986156_F		lincRNA:chr1:183951156-183986156 forward strand	chr1:183959051-183959110	9.31	7.04	4.82
123	A_52_P290799	NM_022320	Gpr35	Mus musculus G protein-coupled receptor 35 (Gpr35), transcript variant 1, mRNA [NM_022320]	chr1:94881911-94881970	8.60	6.33	4.82
124	A_55_P1953618	NM_146974	Olfr1262	Mus musculus olfactory receptor 1262 (Olfr1262), mRNA [NM_146974]	chr2:89843420-89843479	8.41	6.14	4.81
125	A_51_P469285	NM_008737	Nrp1	Mus musculus neuropilin 1 (Nrp1), mRNA [NM_008737]	chr8:131026650-131026709	10.11	7.84	4.81
126	A_55_P2020612	NM_025404	Arl4d	Mus musculus ADP-ribosylation factor-like 4D (Arl4d), mRNA [NM_025404]	chr11:101528989-101529048	10.52	8.26	4.80
127	A_55_P2125376	NM_001083616	Cacna1d	Mus musculus calcium channel, voltage-dependent, L type, alpha 1D subunit (Cacna1d), transcript variant 2, mRNA [NM_001083616]	chr14:30855318-30855259	9.43	7.17	4.80
128	A_30_P01031366	chr17:84470764-84486339_F		lincRNA:chr17:84470764-84486339 forward strand	chr17:84478363-84478422	8.33	6.07	4.79
129	A_55_P2008434	XM_001476747	Gm3450	PREDICTED: Mus musculus hypothetical protein LOC100041646 (LOC100041646), mRNA [XM_001476747]	chr5:64439444-64439503	8.19	5.93	4.79
130	A_55_P2151728	NM_146542	Olfr11	Mus musculus olfactory receptor 11 (Olfr11), mRNA [NM_146542]	chr13:21730508-21730449	7.29	5.03	4.79
131	A_55_P2182675	NM_013524	Fut7	Mus musculus fucosyltransferase 7 (Fut7), transcript variant 1, mRNA [NM_013524]	chr2:25281521-25281580	7.81	5.55	4.78
132	A_30_P01029228	chr18:75132417-75141201_R		lincRNA:chr18:75132417-75141201 reverse strand	chr18:75139745-75139686	8.19	5.93	4.77
133	A_51_P203675	NM_021407	Trem3	Mus musculus triggering receptor expressed on myeloid cells 3 (Trem3), mRNA [NM_021407]	chr17:48397990-48398049	7.41	5.16	4.76
134	A_55_P2045417	NM_001011764	Olfr106	Mus musculus olfactory receptor 106 (Olfr106), mRNA [NM_001011764]	chr17:37532396-37532455	8.00	5.76	4.73
135	A_55_P2246235	NM_022314	Tpm3	Mus musculus tropomyosin 3, gamma (Tpm3), mRNA [NM_022314]	chr3:89877669-89877728	8.78	6.54	4.72
136	A_55_P2144115	NM_028589	1700125H20Rik	Mus musculus RIKEN cDNA 1700125H20 gene (1700125H20Rik), mRNA [NM_028589]	chr11:84994547-84994606	8.48	6.25	4.70
137	A_55_P2177406	XM_001472931	Gm2813	PREDICTED: Mus musculus hypothetical protein LOC100044833 (LOC100044833), mRNA [XM_001472931]	chr5:92192410-92192351	8.09	5.86	4.70

138	A_55_P1974073	ENSMUST00000136202		fibrous sheath-interacting protein 2 Gene [Source:MGI (curated);Acc:MGI:2664111] [ENSMUST00000136202]	chr2:82805590-82805647	7.75	5.52	4.69
139	A_52_P376169	NM_177139	Lypd6	Mus musculus LY6/PLAUR domain containing 6 (Lypd6), transcript variant 1, mRNA [NM_177139]	chr2:50047988-50048047	10.62	8.39	4.67
140	A_55_P2111118	NM_146523	Olfr850	Mus musculus olfactory receptor 850 (Olfr850), mRNA [NM_146523]	chr9:19281804-19281745	7.87	5.64	4.67
141	A_30_P01027976	chr18:35046847-35047423_R		lincRNA:chr18:35046847-35047423 reverse strand	chr18:35047180-35047121	8.47	6.25	4.67
142	A_66_P116028	NM_146828	Olfr975	Mus musculus olfactory receptor 975 (Olfr975), mRNA [NM_146828]	chr9:39757481-39757422	8.75	6.53	4.67
143	A_52_P429450	NM_008694	Ngp	Mus musculus neutrophilic granule protein (Ngp), mRNA [NM_008694]	chr9:110324266-110324814	8.20	5.98	4.66
144	A_55_P2319730	AK019576	4930414F18Rik	Mus musculus adult male testis cDNA, RIKEN full-length enriched library, clone:4930414F18 product:unclassifiable, full insert sequence. [AK019576]	chr16:12629268-12629209	9.47	7.25	4.66
145	A_30_P01023385	chr15:38639259-38639546_R		lincRNA:chr15:38639259-38639546 reverse strand	chr15:38639358-38639299	10.32	8.11	4.65
146	A_30_P01032639	chr2:28151215-28151611_F		lincRNA:chr2:28151215-28151611 forward strand	chr2:28151552-28151611	8.50	6.29	4.63
147	A_30_P01026827	chr2:167404245-167414345_F		lincRNA:chr2:167404245-167414345 forward strand	chr2:167404778-167404837	9.15	6.94	4.62
148	A_51_P160083	ENSMUST00000075081		RIKEN cDNA 1500035N22 gene Gene [Source:MGI Symbol;Acc:MGI:1917508] [ENSMUST00000075081]	chr5:24503886-24503945	9.69	7.48	4.62
149	A_55_P1982004	XR_032923	LOC637146	PREDICTED: Mus musculus similar to MHC class I antigen (LOC637146), misc RNA [XR_032923]		8.12	5.92	4.59
150	A_51_P450278	NM_027237	2010003K11Rik	Mus musculus RIKEN cDNA 2010003K11 gene (2010003K11Rik), mRNA [NM_027237]	chr19:4496987-4496928	8.13	5.93	4.59
151	A_55_P1991617	XM_001473989	2600006L11Rik	PREDICTED: Mus musculus RIKEN cDNA 2600006L11 gene (2600006L11Rik), mRNA [XM_001473989]	chr9:63928583-63928642	9.32	7.12	4.59
152	A_30_P01029666	chr13:98062189-98066613_F		lincRNA:chr13:98062189-98066613 forward strand	chr13:98066473-98066532	8.72	6.53	4.58
153	A_55_P2029401	NM_001177518	Gm7849	Mus musculus predicted gene 7849 (Gm7849), mRNA [NM_001177518]	chr8:22565976-22565917	8.06	5.86	4.58
154	A_55_P2017237	NM_146780	Olfr715	Mus musculus olfactory receptor 715 (Olfr715), mRNA [NM_146780]	chr7:114272021-114271962	7.59	5.40	4.57
155	A_55_P2068247	XM_001473755	Gm2488	PREDICTED: Mus musculus similar to Ubtf protein (LOC100039907), mRNA [XM_001473755]	chr15:13245897-13245838	12.05	9.86	4.57
156	A_66_P121110	NM_011044	Pck1	Mus musculus phosphoenolpyruvate carboxykinase 1, cytosolic (Pck1), mRNA [NM_011044]	chr2:172984307-172984366	8.34	6.15	4.57
157	A_52_P313098	NM_008119	Gip	Mus musculus gastric inhibitory polypeptide (Gip), mRNA [NM_008119]	chr11:95886745-95887966	8.41	6.22	4.56
158	A_55_P2150113	ENSMUST00000098575		Putative uncharacterized protein [Source:UniProtKB/TrEMBL;Acc:Q3UUX0] [ENSMUST00000098575]	chr9:72233128-72233069	8.04	5.86	4.55

159	A_55_P2269289	NM_138650	Dgkg	Mus musculus diacylglycerol kinase, gamma (Dgkg), mRNA [NM_138650]	chr16:22469356-22469297	8.48	6.30	4.54
160	A_55_P1973833	NM_172539	Astl	Mus musculus astacin-like metalloendopeptidase (M12 family) (Astl), mRNA [NM_172539]	chr2:127183328-127183387	8.45	6.27	4.53
161	A_52_P84347	XM_001473883	4931419H13Rik	PREDICTED: Mus musculus RIKEN cDNA 4931419H13 gene (4931419H13Rik), mRNA [XM_001473883]	chr3:54887687-54887836	8.39	6.21	4.53
162	A_55_P1965649	NM_178894	AA792892	Mus musculus expressed sequence AA792892 (AA792892), mRNA [NM_178894]	chr5:94813298-94813357	8.19	6.01	4.53
163	A_30_P01019746	chr12:111617728-111665403_R		lincRNA:chr12:111617728-111665403 reverse strand	chr12:111621845-111621786	10.26	8.08	4.53
164	A_66_P136582	ENSMUST00000082150		high mobility group box 1-like Gene [Source:MGI Symbol;Acc:MGI:3054046] [ENSMUST00000082150]	chr6:131558370-131558311	11.75	9.57	4.53
165	A_30_P01022110	chr5:31862823-31871445_F		lincRNA:chr5:31862823-31871445 forward strand	chr5:31871306-31871365	8.89	6.71	4.53
166	A_55_P2046145	ENSMUST00000019416		PREDICTED: Mus musculus similar to paired-Ig-like receptor A2 (LOC675749), misc RNA [XR_032476]	chr7:3813627-3813568	7.53	5.36	4.52
167	A_55_P2138500	XM_885282	Gm6189	PREDICTED: Mus musculus predicted gene, EG620899 (EG620899), mRNA [XM_885282]	chr1:80379653-80379594	8.58	6.40	4.52
168	A_55_P1964257	ENSMUST00000066625			chr2:79250892-79250951	8.11	5.93	4.50
169	A_30_P01022367	chr11:60728207-60745369_R		lincRNA:chr11:60728207-60745369 reverse strand	chr11:60731874-60731815	8.36	6.20	4.50
170	A_30_P01017527	chr4:3406175-3443875_F		lincRNA:chr4:3406175-3443875 forward strand	chr4:3435225-3435284	9.12	6.96	4.48
171	A_52_P123655	ENSMUST00000059200		RIKEN cDNA A630023P12 gene Gene [Source:MGI Symbol;Acc:MGI:2445162] [ENSMUST00000059200]	chr5:110954673-110954732	7.56	5.40	4.47
172	A_55_P2093211	NM_026205	Rnf151	Mus musculus ring finger protein 151 (Rnf151), mRNA [NM_026205]	chr17:24853215-24853156	8.79	6.63	4.47
173	A_52_P649817	NM_031252	Il23a	Mus musculus interleukin 23, alpha subunit p19 (Il23a), mRNA [NM_031252]	chr10:127733825-127733766	8.02	5.86	4.47
174	A_51_P351015	NM_010735	Lta	Mus musculus lymphotoxin A (Lta), mRNA [NM_010735]	chr17:35340334-35340275	8.19	6.03	4.46
175	A_30_P01019538	chr14:27176652-27227602_F		lincRNA:chr14:27176652-27227602 forward strand	chr14:27202737-27202796	7.96	5.80	4.46
176	A_55_P2123616	NM_001033541	Gm5127	Mus musculus predicted gene 5127 (Gm5127), mRNA [NM_001033541]	chrX:103905812-103905871	8.57	6.42	4.45
177	A_52_P169595	NM_177669	Skint11	Mus musculus selection and upkeep of intraepithelial T cells 11 (Skint11), transcript variant 2, mRNA [NM_177669]	chr4:113917416-113917475	7.40	5.25	4.44
178	A_30_P01018971	chr2:65240793-65292293_F		lincRNA:chr2:65240793-65292293 forward strand	chr2:65252990-65253049	8.82	6.68	4.42
179	A_51_P285736	NM_008798	Pdcd1	Mus musculus programmed cell death 1 (Pdcd1), mRNA [NM_008798]	chr1:95935123-95935064	8.02	5.88	4.42
180	A_55_P1978920	XM_001476569	LOC100041553	PREDICTED: Mus musculus similar to Ubtf protein (LOC100041553), mRNA [XM_001476569]	chrY_random:44926233-44926292	9.95	7.81	4.41

181	A_55_P2156370	NM_001007591	BB014433	Mus musculus expressed sequence BB014433 (BB014433), mRNA [NM_001007591]	chr8:15041741-15041682	9.26	7.11	4.41
182	A_55_P2063618	NR_033493	Gm10818	Mus musculus predicted gene 10818 (Gm10818), non-coding RNA [NR_033493]	chr16:32666418-32666359	10.10	7.96	4.40
183	A_51_P239800	ENSMUST00000108686		myosin, heavy polypeptide 8, skeletal muscle, perinatal Gene [Source:MGI (curated);Acc:MGI:1339712] [ENSMUST00000108686]	chr11:67108324-67108383	7.38	5.25	4.39
184	A_55_P2180849	XM_001475514	AA684185	PREDICTED: Mus musculus similar to elongation factor 1 homolog (S. cerevisiae) (LOC100040632), mRNA [XM_001475514]	chr18:80210484-80210543	8.91	6.78	4.38
185	A_51_P474551	NM_007810	Cyp19a1	Mus musculus cytochrome P450, family 19, subfamily a, polypeptide 1 (Cyp19a1), mRNA [NM_007810]	chr9:54013837-54013778	9.34	7.21	4.37
186	A_55_P2048289	NM_205819	Tlr11	Mus musculus toll-like receptor 11 (Tlr11), mRNA [NM_205819]	chr14:50983110-50983169	7.97	5.85	4.37
187	A_30_P01029556	chr4:101040523-101080248_R		lincRNA:chr4:101040523-101080248 reverse strand	chr4:101077369-101077310	7.93	5.80	4.37
188	A_30_P01021905	chr4:8606650-8614031_R		lincRNA:chr4:8606650-8614031 reverse strand	chr4:8610224-8610165	9.13	7.01	4.36
189	A_55_P2154148	NR_033642	Gm3230	Mus musculus predicted gene 3230 (Gm3230), non-coding RNA [NR_033642]	chr2:19577492-19577433	8.16	6.04	4.35
190	A_30_P01017533	chr9:20313342-20323517_R		lincRNA:chr9:20313342-20323517 reverse strand	chr9:20323419-20323360	11.33	9.21	4.33
191	A_55_P1999923	NM_175521	6430598A04Rik	Mus musculus RIKEN cDNA 6430598A04 gene (6430598A04Rik), mRNA [NM_175521]	chr5:138172309-138172250	13.64	11.52	4.33
192	A_55_P1986701	XM_895013	1700055N04Rik	PREDICTED: Mus musculus RIKEN cDNA 1700055N04 gene, transcript variant 1 (1700055N04Rik), mRNA [XM_895013]	chr19:3966478-3966537	7.50	5.38	4.32
193	A_66_P119964	ENSMUST00000103546	LOC636126	Immunglobulin heavy chain Precursor; Fragment [Source:UniProtKB/TrEMBL;Acc:A0N1R4] [ENSMUST00000103546]	chr12:117107172-117107113	8.21	6.10	4.31
194	A_55_P1982857	ENSMUST00000062214		RIKEN cDNA 1500002C15 gene Gene [Source:MGI Symbol;Acc:MGI:1916196] [ENSMUST00000062214]	chr4:155108559-155108500	9.42	7.31	4.31
195	A_30_P01018899	chr2:72825297-72826470_F		lincRNA:chr2:72825297-72826470 forward strand	chr2:72825311-72825370	8.64	6.53	4.31
196	A_30_P01017707	chr3:30077338-30080281_R		lincRNA:chr3:30077338-30080281 reverse strand	chr3:30077398-30077339	7.93	5.83	4.30
197	A_51_P271865	NM_016751	Clec4f	Mus musculus C-type lectin domain family 4, member f (Clec4f), mRNA [NM_016751]	chr6:83595339-83595281	8.48	6.38	4.30
198	A_52_P71624	NM_146491	Olfr1410	Mus musculus olfactory receptor 1410 (Olfr1410), mRNA [NM_146491]	chr1:94505050-94505109	12.77	10.67	4.29
199	A_52_P321140	NM_007843	Defb1	Mus musculus defensin beta 1 (Defb1), mRNA [NM_007843]	chr8:22905041-22905100	7.80	5.70	4.29
200	A_30_P01031006	chr5:35492530-35492959_R		lincRNA:chr5:35492530-35492959 reverse strand	chr5:35492795-35492736	8.56	6.46	4.28

201	A_52_P408315	ENSMUST00000124404		von Willebrand factor A domain containing 3B Gene [Source:MGI (curated);Acc:MGI:1918103] [ENSMUST00000124404]	chr1:37120334-37120393	7.94	5.85	4.28
202	A_55_P2016114	NM_010177	Fasl	Mus musculus Fas ligand (TNF superfamily, member 6) (Fasl), mRNA [NM_010177]	chr1:163711115-163711056	9.03	6.94	4.25
203	A_30_P01021626	chr7:91558475-91733625_R		lincRNA:chr7:91558475-91733625 reverse strand	chr7:91725532-91725473	8.26	6.18	4.25
204	A_52_P355084	NM_144797	Metrnl	Mus musculus meteorin, glial cell differentiation regulator-like (Metrnl), mRNA [NM_144797]	chr11:121577358-121577417	8.60	6.51	4.25
205	A_51_P371867	NM_027512	3830417A13Rik	Mus musculus RIKEN cDNA 3830417A13 gene (3830417A13Rik), mRNA [NM_027512]	chrX:61431614-61431673	7.62	5.54	4.25
206	A_30_P01031392	chr5:22810359-22836386_F		lincRNA:chr5:22810359-22836386 forward strand	chr5:22836318-22836377	7.75	5.66	4.25
207	A_52_P354298	NM_007459	Ap2a2	Mus musculus adaptor protein complex AP-2, alpha 2 subunit (Ap2a2), mRNA [NM_007459]	chr7:148818293-148818352	11.99	9.90	4.24
208	A_51_P110381	NM_144943	Cd207	Mus musculus CD207 antigen (Cd207), mRNA [NM_144943]	chr6:83621501-83621442	8.58	6.50	4.23
209	A_66_P102110	XM_001474798	LOC100045717	PREDICTED: Mus musculus similar to ribosomal protein L10 (LOC100045717), mRNA [XM_001474798]	chr2:162619343-162619402	8.06	5.98	4.23
210	A_55_P1986868	XR_032569	Gm3267	PREDICTED: Mus musculus similar to ribosomal protein (LOC100041313), misc RNA [XR_032569]	chr15:37423179-37423238	8.25	6.18	4.22
211	A_55_P2038203	NR_027955	4931440P22Rik	Mus musculus RIKEN cDNA 4931440P22 gene (4931440P22Rik), non-coding RNA [NR_027955]	chr3:65332963-65332904	9.10	7.02	4.22
212	A_55_P2051367	ENSMUST00000100107		Putative uncharacterized protein [Source:UniProtKB/TrEMBL;Acc:Q3TTI1] [ENSMUST00000100107]	chr16:18029689-18029748	8.59	6.52	4.22
213	A_55_P2006874	ENSMUST00000103490		Mus musculus (clone 5-14) anti-fluorescein antibody IgH chain mRNA, V-region. [L39085]	chr12:115694016-115693957	8.06	5.99	4.20
214	A_55_P1988318	XM_001472135	Gm6127	PREDICTED: Mus musculus similar to dentin sialophosphoprotein precursor (LOC620104), mRNA [XM_001472135]	chr6:31652304-31652245	7.93	5.86	4.20
215	A_51_P487918	NM_177158	Rinl	Mus musculus Ras and Rab interactor-like (Rinl), mRNA [NM_177158]	chr7:29583737-29583796	9.49	7.42	4.19
216	A_52_P521564	NM_001163103	Gm70	Mus musculus predicted gene 70 (Gm70), mRNA [NM_001163103]	chr12:77539089-77539378	8.06	5.99	4.19
217	A_52_P446457	NM_001163192	Ly6g6f	Mus musculus lymphocyte antigen 6 complex, locus G6F (Ly6g6f), mRNA [NM_001163192]	chr17:35218024-35217819	7.51	5.44	4.18
218	A_55_P2032458	XM_893730	Gm6934	PREDICTED: Mus musculus predicted gene, EG628919 (EG628919), mRNA [XM_893730]	chr17:51973934-51973993	9.10	7.04	4.18
219	A_52_P399990	NM_017377	B4galt2	Mus musculus UDP-Gal:betaGlcNAc beta 1,4-galactosyltransferase, polypeptide 2 (B4galt2), mRNA [NM_017377]	chr4:117546012-117545953	10.31	8.25	4.18

220	A_55_P2101992	NM_146844	Olfr1107	Mus musculus olfactory receptor 1107 (Olfr1107), mRNA [NM_146844]	chr2:86911410-86911351	8.25	6.19	4.18
221	A_55_P2148609	XM_001473597	Gm9460	PREDICTED: Mus musculus hypothetical LOC669454 (LOC669454), mRNA [XM_001473597]	chr1:180249114-180249173	8.90	6.84	4.17
222	A_66_P125873	NM_172410	Nup93	Mus musculus nucleoporin 93 (Nup93), mRNA [NM_172410]	chr8:96838885-96838944	10.40	8.34	4.16
223	A_30_P01021190	chr16:9249688-9275013_F		lincRNA:chr16:9249688-9275013 forward strand	chr16:9259984-9260043	8.14	6.08	4.16
224	A_55_P2089472	NM_001163483	1700020A23Rik	Mus musculus RIKEN cDNA 1700020A23 gene (1700020A23Rik), transcript variant 1, mRNA [NM_001163483]	chr2:130231747-130231806	8.16	6.10	4.16
225	A_51_P105887	NM_172842	Lax1	Mus musculus lymphocyte transmembrane adaptor 1 (Lax1), transcript variant 2, mRNA [NM_172842]	chr1:135576189-135576130	8.52	6.46	4.16
226	A_30_P01023697	chr2:167388900-167402475_R		lincRNA:chr2:167388900-167402475 reverse strand	chr2:167400896-167400837	8.12	6.06	4.16
227	A_30_P01033342	chr3:121997064-122031218_F		lincRNA:chr3:121997064-122031218 forward strand	chr3:121997194-121997253	9.64	7.59	4.15
228	A_66_P104923	ENSMUST00000103548	LOC100046359	PREDICTED: Mus musculus similar to Ighg1 protein (LOC100046359), mRNA [XM_001473125]	chr12:117158672-117158613	8.47	6.42	4.15
229	A_55_P2119888	NM_010154	Erbb4	Mus musculus v-erb-a erythroblastic leukemia viral oncogene homolog 4 (avian) (Erbb4), mRNA [NM_010154]	chr1:68086745-68086686	10.05	8.00	4.15
230	A_55_P2107437	ENSMUST00000068970		RIKEN cDNA 1700045I11 gene Gene [Source:MGI Symbol;Acc:MGI:1920600] [ENSMUST00000068970]	chr4:41644771-41644712	8.38	6.33	4.14
231	A_55_P2006410	XM_001479363	LOC100048112	PREDICTED: Mus musculus hypothetical protein LOC100048112 (LOC100048112), mRNA [XM_001479363]	chr7:87391613-87391554	8.70	6.66	4.13
232	A_51_P516133	NM_015786	Hist1h1c	Mus musculus histone cluster 1, H1c (Hist1h1c), mRNA [NM_015786]	chr13:23832018-23832077	11.96	9.92	4.11
233	A_66_P105771	XM_001474242	LOC669999	PREDICTED: Mus musculus similar to ribosomal protein (LOC669999), mRNA [XM_001474242]	chr18:34369404-34369345	7.99	5.95	4.11
234	A_30_P01019581	chr7:132218864-132329172_F		lincRNA:chr7:132218864-132329172 forward strand	chr7:132326092-132326151	7.78	5.74	4.11
235	A_55_P2027668	ENSMUST00000058829	Neto1	neuropilin (NRP) and tolloid (TLL)-like 1 Gene [Source:MGI Symbol;Acc:MGI:2180216] [ENSMUST00000058829]	chr18:86672089-86672148	10.29	8.26	4.11
236	A_55_P2064506	NM_001037247	Defb36	Mus musculus defensin beta 36 (Defb36), mRNA [NM_001037247]	chr2:152438270-152438329	8.15	6.11	4.09
237	A_30_P01031520	chr10:60473030-60535340_F		lincRNA:chr10:60473030-60535340 forward strand	chr10:60528810-60528869	8.01	5.99	4.07
238	A_30_P01024981	chr1:23340097-23340552_F		lincRNA:chr1:23340097-23340552 forward strand	chr1:23340480-23340539	8.46	6.43	4.07
239	A_51_P400217	NM_016982	Vpreb1	Mus musculus pre-B lymphocyte gene 1 (Vpreb1), mRNA [NM_016982]	chr16:16868618-16868559	8.95	6.93	4.07
240	A_52_P636987	ENSMUST00000105113		PREDICTED: Mus musculus gene model 362, (NCBI) (Gm362), mRNA [XM_141720]	chrX:40945674-40945733	7.82	5.80	4.06

241	A_30_P01027392	chrX:151738814-151740969_R		lincRNA:chrX:151738814-151740969 reverse strand	chrX:151739052-151738993	8.38	6.36	4.05
242	A_55_P2122010	NM_146332	Olfr135	Mus musculus olfactory receptor 135 (Olfr135), mRNA [NM_146332]	chr17:38346071-38346130	7.38	5.36	4.05
243	A_52_P335466	NM_001081270	Dscaml1	Mus musculus Down syndrome cell adhesion molecule-like 1 (Dscaml1), mRNA [NM_001081270]	chr9:45255979-45256038	11.76	9.75	4.03
244	A_51_P223724	NM_133235	Khdrbs2	Mus musculus KH domain containing, RNA binding, signal transduction associated 2 (Khdrbs2), mRNA [NM_133235]	chr1:32714406-32714465	10.10	8.09	4.03
245	A_55_P1987379	NM_146328	Olfr110	Mus musculus olfactory receptor 110 (Olfr110), mRNA [NM_146328]	chr17:37636492-37636551	8.28	6.27	4.03
246	A_52_P1142105	ENSMUST00000103464		immunoglobulin heavy chain (X24 family) Complex/Cluster/Region [Source:MGI Symbol;Acc:MGI:96492] [ENSMUST00000103464]	chr12:115186664-115186605	7.84	5.83	4.03
247	A_52_P91274	XM_988035	1700018G05Rik	PREDICTED: Mus musculus RIKEN cDNA 1700018G05 gene (1700018G05Rik), mRNA [XM_988035]	chrX:100124337-100124396	8.19	6.19	4.03
248	A_30_P01019706	chr13:112337405-112356455_R		lincRNA:chr13:112337405-112356455 reverse strand	chr13:112350936-112350877	7.99	5.98	4.02
249	A_30_P01021440	chr17:9889983-9919633_R		lincRNA:chr17:9889983-9919633 reverse strand	chr17:9894792-9894733	7.50	5.50	4.01
250	A_52_P594584	NM_173069	Speer2	Mus musculus spermatogenesis associated glutamate (E)-rich protein 2 (Speer2), mRNA [NM_173069]	chr16:69859059-69859000	7.70	5.70	4.01
251	A_30_P01022872	chr18:75132417-75141201_R		lincRNA:chr18:75132417-75141201 reverse strand	chr18:75134377-75134318	7.63	5.62	4.01
252	A_55_P1973377	NM_145967	Vstm2a	Mus musculus V-set and transmembrane domain containing 2A (Vstm2a), mRNA [NM_145967]	chr11:16184091-16184150	8.86	6.86	4.00
253	A_55_P1975667	NM_001039698	Rhox4g	Mus musculus reproductive homeobox 4G (Rhox4g), mRNA [NM_001039698]	chrX:35061231-35061172	8.67	6.67	4.00
254	A_52_P573467	NM_008644	Prol1	Mus musculus proline rich, lacrimal 1 (Prol1), mRNA [NM_008644]	chr5:88757596-88757655	5.71	7.71	0.25
255	A_30_P01023018	chr4:146156575-146175425_R		lincRNA:chr4:146156575-146175425 reverse strand	chr4:146157658-146157599	6.45	8.45	0.25
256	A_55_P2123902	NM_178777	Nhlh2	Mus musculus nescient helix loop helix 2 (Nhlh2), mRNA [NM_178777]	chr3:101818350-101818409	8.63	10.64	0.25
257	A_55_P2159885	NM_001166584	Tead1	Mus musculus TEA domain family member 1 (Tead1), transcript variant 1, mRNA [NM_001166584]	chr7:120043445-120043504	10.95	12.95	0.25
258	A_55_P2091060	NM_153153	Svil	Mus musculus supervillin (Svil), transcript variant 1, mRNA [NM_153153]	chr18:5049365-5049424	6.99	8.99	0.25
259	A_52_P585652	NM_173182	Fndc3b	Mus musculus fibronectin type III domain containing 3B (Fndc3b), mRNA [NM_173182]	chr3:27315788-27315729	6.18	8.18	0.25
260	A_55_P2011852	NM_183417	Cdk2	Mus musculus cyclin-dependent kinase 2 (Cdk2), transcript variant 1, mRNA [NM_183417]	chr10:128135717-128135658	6.21	8.22	0.25
261	A_55_P2108837	NM_009448	Tuba1c	Mus musculus tubulin, alpha 1C (Tuba1c), mRNA [NM_009448]	chr15:98868477-98868536	11.46	13.47	0.25

262	A_55_P2074796	NM_007671	Cdkn2c	Mus musculus cyclin-dependent kinase inhibitor 2C (p18, inhibits CDK4) (Cdkn2c), mRNA [NM_007671]	chr4:109333567-109333508	5.64	7.65	0.25
263	A_55_P2067518	NM_054055	Slc13a3	Mus musculus solute carrier family 13 (sodium-dependent dicarboxylate transporter), member 3 (Slc13a3), mRNA [NM_054055]	chr2:165230855-165230796	5.74	7.75	0.25
264	A_55_P2183587	NM_010296	Gli1	Mus musculus GLI-Kruppel family member GLI1 (Gli1), mRNA [NM_010296]	chr10:126767026-126766967	7.79	9.80	0.25
265	A_51_P330213	NM_024184	Asf1b	Mus musculus ASF1 anti-silencing function 1 homolog B (S. cerevisiae) (Asf1b), mRNA [NM_024184]	chr8:86493842-86493901	6.82	8.83	0.25
266	A_55_P1967168	NM_172668	Lrp4	Mus musculus low density lipoprotein receptor-related protein 4 (Lrp4), transcript variant 1, mRNA [NM_172668]	chr2:91353566-91353625	6.71	8.73	0.25
267	A_51_P306789	NR_015543	2810055G20Rik	Mus musculus RIKEN cDNA 2810055G20 gene (2810055G20Rik), non-coding RNA [NR_015543]	chr16:77501748-77501806	8.07	10.08	0.25
268	A_52_P638798	NM_001164370	Mipol1	Mus musculus mirror-image polydactyly gene 1 homolog (human) (Mipol1), mRNA [NM_001164370]	chr12:58407026-58407085	8.50	10.52	0.25
269	A_30_P01019094	chr2:60581618-60589907_F		lincRNA:chr2:60581618-60589907 forward strand	chr2:60589810-60589869	9.29	11.31	0.25
270	A_55_P2111355	NM_178444	Egfl7	Mus musculus EGF-like domain 7 (Egfl7), transcript variant a, mRNA [NM_178444]	chr2:26448142-26448201	8.81	10.83	0.25
271	A_55_P1977855	NM_178280	Sall3	Mus musculus sal-like 3 (Drosophila) (Sall3), mRNA [NM_178280]	chr18:81163195-81163136	6.07	8.09	0.25
272	A_55_P1988766	ENSMUST00000101091		Putative uncharacterized protein [Source:UniProtKB/TrEMBL;Acc:Q3UWC7] [ENSMUST00000101091]	chr6:108421112-108421053	6.51	8.53	0.25
273	A_55_P2148370	XR_031785	Gm2862	PREDICTED: Mus musculus hypothetical protein LOC100045710 (LOC100045710), misc RNA [XR_031785]	chr15:25611930-25611989	6.39	8.41	0.25
274	A_51_P240019	NM_013755	Gyg	Mus musculus glycogenin (Gyg), mRNA [NM_013755]	chr3:20022077-20022018	8.16	10.18	0.25
275	A_55_P2027836	NM_020275	Tnfrsf10b	Mus musculus tumor necrosis factor receptor superfamily, member 10b (Tnfrsf10b), mRNA [NM_020275]	chr14:70184141-70184200	7.34	9.36	0.25
276	A_55_P2084303	NM_010917	Nid1	Mus musculus nidogen 1 (Nid1), mRNA [NM_010917]	chr13:13530138-13555992	9.16	11.19	0.25
277	A_30_P01025778	chr1:71939741-71947891_R		lincRNA:chr1:71939741-71947891 reverse strand	chr1:71940250-71940191	5.82	7.85	0.25
278	A_55_P2074942	NM_010484	Slc6a4	Mus musculus solute carrier family 6 (neurotransmitter transporter, serotonin), member 4 (Slc6a4), mRNA [NM_010484]	chr11:76845777-76845836	9.97	11.99	0.25
279	A_51_P171999	NM_009696	Apoe	Mus musculus apolipoprotein E (Apoe), mRNA [NM_009696]	chr7:20281791-20281732	9.82	11.85	0.24
280	A_55_P2019312	NM_178396	Car12	Mus musculus carbonic anyhydrase 12 (Car12), mRNA [NM_178396]	chr9:66614542-66614601	6.40	8.43	0.24
281	A_30_P01028936	chr17:33995525-34028025_F		lincRNA:chr17:33995525-34028025 forward strand	chr17:34023795-34023854	7.53	9.56	0.24

282	A_51_P293339	NM_009427	Tob1	Mus musculus transducer of ErbB-2.1 (Tob1), mRNA [NM_009427]	chr11:94075956-94076015	8.74	10.77	0.24
283	A_55_P2276105	AK157781		Mus musculus 9.5 days embryo parthenogenote cDNA, RIKEN full-length enriched library, clone:B130038C21 product:hypothetical Beta tubulin containing protein, full insert sequence. [AK157781]	chr15:13101015-13100956	6.91	8.95	0.24
284	A_51_P254805	NM_008446	Kif4	Mus musculus kinesin family member 4 (Kif4), mRNA [NM_008446]	chrX:97922085-97922145	6.17	8.21	0.24
285	A_55_P1999648	NM_007408	Plin2	Mus musculus perilipin 2 (Plin2), mRNA [NM_007408]	chr4:86302906-86302847	7.07	9.11	0.24
286	A_55_P1960916	NM_028133	Egln3	Mus musculus EGL nine homolog 3 (C. elegans) (Egln3), mRNA [NM_028133]	chr12:55281311-55281252	9.98	12.02	0.24
287	A_52_P399584	NM_181589	Ckap2l	Mus musculus cytoskeleton associated protein 2-like (Ckap2l), mRNA [NM_181589]	chr2:129094717-129094658	6.25	8.29	0.24
288	A_55_P2111513	NM_021324	Ttyh1	Mus musculus tweety homolog 1 (Drosophila) (Ttyh1), transcript variant 2, mRNA [NM_021324]	chr7:4087717-4087776	6.34	8.39	0.24
289	A_30_P01020529	chr1:64686823-64728598_F		lincRNA:chr1:64686823-64728598 forward strand	chr1:64726865-64726924	6.81	8.85	0.24
290	A_55_P2109585	NM_172743	Plekha7	Mus musculus pleckstrin homology domain containing, family A member 7 (Plekha7), mRNA [NM_172743]	chr7:123267859-123267800	6.33	8.37	0.24
291	A_55_P2071646	ENSMUST00000054395		Putative uncharacterized proteinMCG148442 ; [Source:UniProtKB/TrEMBL;Acc:Q9D2M0] [ENSMUST00000054395]	chr5:136365387-136365328	8.43	10.47	0.24
292	A_55_P2060672	NM_010452	Hoxa3	Mus musculus homeobox A3 (Hoxa3), mRNA [NM_010452]	chr6:52119146-52119087	8.23	10.28	0.24
293	A_55_P2002460	XM_001473424	Gm2399	PREDICTED: Mus musculus similar to Nid1 protein (LOC100039744), mRNA [XM_001473424]	chr13:12794813-12794872	8.23	10.28	0.24
294	A_51_P404077	NM_020510	Fzd2	Mus musculus frizzled homolog 2 (Drosophila) (Fzd2), mRNA [NM_020510]	chr11:102467613-102467672	7.58	9.63	0.24
295	A_51_P517430	NM_007639	Cd1d1	Mus musculus CD1d1 antigen (Cd1d1), mRNA [NM_007639]	chr3:86799850-86799791	7.30	9.35	0.24
296	A_30_P01031192	chr6:52046805-52072744_R		lincRNA:chr6:52046805-52072744 reverse strand	chr6:52056852-52056793	5.98	8.03	0.24
297	A_52_P679101	NM_011597	Tjp2	Mus musculus tight junction protein 2 (Tjp2), mRNA [NM_011597]	chr19:24169790-24169731	8.58	10.64	0.24
298	A_51_P255682	NM_008594	Mfge8	Mus musculus milk fat globule-EGF factor 8 protein (Mfge8), transcript variant 1, mRNA [NM_008594]	chr7:86278748-86278689	10.41	12.47	0.24
299	A_55_P2049752	NM_173749	Pamr1	Mus musculus peptidase domain containing associated with muscle regeneration 1 (Pamr1), mRNA [NM_173749]	chr2:102483126-102483185	6.35	8.40	0.24
300	A_52_P649561	NM_175256	Heg1	Mus musculus HEG homolog 1 (zebrafish) (Heg1), mRNA [NM_175256]	chr16:33767397-33767456	7.18	9.24	0.24

301	A_55_P2142451	NM_026728	Echdc2	Mus musculus enoyl Coenzyme A hydratase domain containing 2 (Echdc2), mRNA [NM_026728]	chr4:107851853-107851912	6.63	8.69	0.24
302	A_55_P2044684	NM_025290	Rsph1	Mus musculus radial spoke head 1 homolog (Chlamydomonas) (Rsph1), mRNA [NM_025290]	chr17:31392028-31391969	6.08	8.14	0.24
303	A_51_P307316	NM_027875	Syde1	Mus musculus synapse defective 1, Rho GTPase, homolog 1 (C. elegans) (Syde1), mRNA [NM_027875]	chr10:78047421-78047362	8.21	10.27	0.24
304	A_52_P233441	NM_008090	Gata2	Mus musculus GATA binding protein 2 (Gata2), mRNA [NM_008090]	chr6:88156362-88156421	9.24	11.30	0.24
305	A_52_P129428	NM_020510	Fzd2	Mus musculus frizzled homolog 2 (Drosophila) (Fzd2), mRNA [NM_020510]	chr11:102469045-102469104	6.11	8.18	0.24
306	A_51_P142515	NM_133738	Antxr2	Mus musculus anthrax toxin receptor 2 (Antxr2), mRNA [NM_133738]	chr5:98313877-98313818	6.78	8.85	0.24
307	A_52_P390944	NM_016803	Chst3	Mus musculus carbohydrate (chondroitin 6/keratan) sulfotransferase 3 (Chst3), mRNA [NM_016803]	chr10:59644565-59644506	6.19	8.25	0.24
308	A_51_P464738	NM_011400	Slc2a1	Mus musculus solute carrier family 2 (facilitated glucose transporter), member 1 (Slc2a1), mRNA [NM_011400]	chr4:118809864-118809923	9.66	11.73	0.24
309	A_52_P79385	NM_025952	Magt1	Mus musculus magnesium transporter 1 (Magt1), transcript variant 2, mRNA [NM_025952]	chrX:103169548-103166784	8.92	10.99	0.24
310	A_55_P2005672	XR_033524	Gm7996	PREDICTED: Mus musculus similar to Hmga2 protein (LOC666235), misc RNA [XR_033524]	chr1:178767319-178767378	5.94	8.01	0.24
311	A_66_P108267	NM_148935	Foxn4	Mus musculus forkhead box N4 (Foxn4), mRNA [NM_148935]	chr5:114704268-114704209	6.39	8.46	0.24
312	A_55_P1985905	NM_001077632	Nkx2-2	Mus musculus NK2 transcription factor related, locus 2 (Drosophila) (Nkx2-2), transcript variant 2, mRNA [NM_001077632]	chr2:147003366-147003307	7.86	9.93	0.24
313	A_66_P126563	NM_026865	1700113I22Rik	Mus musculus RIKEN cDNA 1700113I22 gene (1700113I22Rik), mRNA [NM_026865]	chr11:101280408-101280349	6.50	8.58	0.24
314	A_55_P1983769	NM_001012273	Birc5	Mus musculus baculoviral IAP repeat-containing 5 (Birc5), transcript variant 3, mRNA [NM_001012273]	chr11:117714057-117714116	7.97	10.04	0.24
315	A_51_P487813	NM_016753	Lxn	Mus musculus latexin (Lxn), mRNA [NM_016753]	chr3:67262048-67261989	8.34	10.42	0.24
316	A_55_P2007708	ENSMUST00000012540	Nanog	Nanog homeobox Gene [Source:MGI (curated);Acc:MGI:1919200] [ENSMUST00000012540]	chr6:122664590-122664649	6.31	8.39	0.24
317	A_55_P2176325	NM_177782	Prex1	Mus musculus phosphatidylinositol-3,4,5-trisphosphate-dependent Rac exchange factor 1 (Prex1), mRNA [NM_177782]	chr2:166392552-166392493	8.41	10.50	0.24
318	A_52_P225570	NM_020606	Parva	Mus musculus parvin, alpha (Parva), mRNA [NM_020606]	chr7:119734646-119734705	7.80	9.88	0.24

319	A_52_P381665	NM_133919	Aff1	Mus musculus AF4/FMR2 family, member 1 (Aff1), transcript variant 2, mRNA [NM_133919]	chr5:104284222-104284281	6.54	8.62	0.24
320	A_52_P385594	NM_009317	Tal2	Mus musculus T-cell acute lymphocytic leukemia 2 (Tal2), mRNA [NM_009317]	chr4:53799664-53799723	6.24	8.33	0.24
321	A_55_P2137701	NM_001177767	Gm13138	Mus musculus predicted gene 13138 (Gm13138), mRNA [NM_001177767]	chr4:145889494-145889553	7.39	9.47	0.24
322	A_55_P2012171	NM_026470	Spata6	Mus musculus spermatogenesis associated 6 (Spata6), mRNA [NM_026470]	chr4:111447424-111447483	7.00	9.08	0.24
323	A_55_P1957424	NM_172294	Sulf1	Mus musculus sulfatase 1 (Sulf1), mRNA [NM_172294]	chr1:12849507-12849566	7.14	9.23	0.24
324	A_55_P2030160	NM_001142920	Tcf7l2	Mus musculus transcription factor 7-like 2, T-cell specific, HMG-box (Tcf7l2), transcript variant 4, mRNA [NM_001142920]	chr19:55994029-55994088	7.66	9.75	0.24
325	A_51_P279437	NM_029662	Mfsd2a	Mus musculus major facilitator superfamily domain containing 2A (Mfsd2a), mRNA [NM_029662]	chr4:122624241-122624182	6.71	8.81	0.23
326	A_51_P258690	NM_009136	Scrg1	Mus musculus scrapie responsive gene 1 (Scrg1), mRNA [NM_009136]	chr8:59956257-59956316	5.89	7.98	0.23
327	A_30_P01028916	chr4:54851872-54866706_R		lincRNA:chr4:54851872-54866706 reverse strand	chr4:54865590-54865531	6.20	8.29	0.23
328	A_55_P2099665	ENSMUST00000115332		G protein-coupled receptor 37 Gene [Source:MGI Symbol;Acc:MGI:1313297] [ENSMUST00000115332]	chr6:25616953-25616894	6.11	8.20	0.23
329	A_30_P01031832	chr13:23517278-23523756_F		lincRNA:chr13:23517278-23523756 forward strand	chr13:23521765-23521824	6.29	8.38	0.23
330	A_55_P2004801	NM_001040435	Tacc3	Mus musculus transforming, acidic coiled-coil containing protein 3 (Tacc3), mRNA [NM_001040435]	chr5:34014638-34014697	8.26	10.36	0.23
331	A_55_P1992910	NM_013755	Gyg	Mus musculus glycogenin (Gyg), mRNA [NM_013755]	chr3:20022092-20022033	7.88	9.98	0.23
332	A_66_P126332	NM_001110508	Zfp703	Mus musculus zinc finger protein 703 (Zfp703), transcript variant 2, mRNA [NM_001110508]	chr8:28091863-28091922	9.73	11.83	0.23
333	A_52_P263658	NM_008236	Hes2	Mus musculus hairy and enhancer of split 2 (Drosophila) (Hes2), mRNA [NM_008236]	chr4:151536406-151536465	6.90	9.00	0.23
334	A_55_P2105100	NM_008377	Lrig1	Mus musculus leucine-rich repeats and immunoglobulin-like domains 1 (Lrig1), mRNA [NM_008377]	chr6:94554582-94554523	7.88	9.99	0.23
335	A_55_P2171508	NM_153138	Wipf1	Mus musculus WAS/WASL interacting protein family, member 1 (Wipf1), mRNA [NM_153138]	chr2:73270282-73270223	6.54	8.64	0.23
336	A_66_P119457	NM_175189	Hepacam	Mus musculus hepatocyte cell adhesion molecule (Hepacam), mRNA [NM_175189]	chr9:37193184-37193243	6.31	8.42	0.23
337	A_51_P148105	NM_011234	Rad51	Mus musculus RAD51 homolog (S. cerevisiae) (Rad51), mRNA [NM_011234]	chr2:118961264-118961323	6.15	8.26	0.23
338	A_30_P01021383	chr1:34735032-34781146_F		lincRNA:chr1:34735032-34781146 forward strand	chr1:34780946-34781005	7.01	9.12	0.23
339	A_51_P481920	NM_009828	Ccna2	Mus musculus cyclin A2 (Ccna2), mRNA [NM_009828]	chr3:36464037-36463978	6.43	8.54	0.23

340	A_55_P2016069	NM_029967	Adamtsl1	Mus musculus ADAMTS-like 1 (Adamtsl1), mRNA [NM_029967]	chr4:86074196-86074255	6.45	8.57	0.23
341	A_52_P109548	NM_008635	Mtap7	Mus musculus microtubule-associated protein 7 (Mtap7), mRNA [NM_008635]	chr10:20000837-20000896	6.91	9.03	0.23
342	A_30_P01025068	H19		lincRNA:chr7:149761435-149764019 reverse strand	chr7:149761815-149761756	10.45	12.57	0.23
343	A_52_P93467	NM_011521	Sdc4	Mus musculus syndecan 4 (Sdc4), mRNA [NM_011521]	chr2:164251270-164251211	7.47	9.59	0.23
344	A_55_P2095880	ENSMUST00000109764	Nfix	nuclear factor I/X Gene [Source:MGI (curated);Acc:MGI:97311] [ENSMUST00000109764]	chr8:87228818-87228759	6.48	8.60	0.23
345	A_51_P164203	NM_019731	Nme4	Mus musculus non-metastatic cells 4, protein expressed in (Nme4), nuclear gene encoding mitochondrial protein, mRNA [NM_019731]	chr17:26228811-26228752	6.41	8.53	0.23
346	A_55_P2201822	NM_001163564	Naf1	Mus musculus nuclear assembly factor 1 homolog (S. cerevisiae) (Naf1), mRNA [NM_001163564]	chr8:69414396-69414455	9.44	11.57	0.23
347	A_52_P552550	NM_009642	Agtrap	Mus musculus angiotensin II, type I receptor-associated protein (Agtrap), mRNA [NM_009642]	chr4:147454490-147454431	7.12	9.25	0.23
348	A_52_P679105	NM_029614	Prss23	Mus musculus protease, serine, 23 (Prss23), mRNA [NM_029614]	chr7:96657714-96657655	6.15	8.28	0.23
349	A_55_P1995007	NM_181416	Arhgap11a	Mus musculus Rho GTPase activating protein 11A (Arhgap11a), mRNA [NM_181416]	chr2:113671708-113671649	6.38	8.51	0.23
350	A_51_P298107	NM_028813	Vit	Mus musculus vitrin (Vit), mRNA [NM_028813]	chr17:79026461-79026520	6.86	9.00	0.23
351	A_51_P317640	NM_009367	Tgfb2	Mus musculus transforming growth factor, beta 2 (Tgfb2), mRNA [NM_009367]	chr1:188447593-188447534	6.99	9.12	0.23
352	A_55_P2174601	XM_001477810	LOC100047292	PREDICTED: Mus musculus similar to pleckstrin homology domain containing, family A member 7 (LOC100047292), mRNA [XM_001477810]	chr7:123267206-123267147	6.27	8.41	0.23
353	A_52_P159256	NM_178665	Lpp	Mus musculus LIM domain containing preferred translocation partner in lipoma (Lpp), transcript variant 1, mRNA [NM_178665]	chr16:24988828-24988887	7.11	9.25	0.23
354	A_66_P105422	NM_028894	Lonrf3	Mus musculus LON peptidase N-terminal domain and ring finger 3 (Lonrf3), mRNA [NM_028894]	chrX:33906756-33906815	6.70	8.84	0.23
355	A_51_P128876	NM_025378	Ifitm3	Mus musculus interferon induced transmembrane protein 3 (Ifitm3), mRNA [NM_025378]	chr7:148195650-148195591	8.90	11.05	0.23
356	A_52_P411003	NM_144553	Dlgap5	Mus musculus discs, large (Drosophila) homolog-associated protein 5 (Dlgap5), transcript variant 1, mRNA [NM_144553]	chr14:48011417-48010299	6.52	8.67	0.23
357	A_55_P2008066	NM_010585	Itpr1	Mus musculus inositol 1,4,5-triphosphate receptor 1 (Itpr1), mRNA [NM_010585]	chr6:108501025-108501084	6.94	9.09	0.23

358	A_55_P1971897	NM_010784	Mdk	Mus musculus midkine (Mdk), transcript variant 1, mRNA [NM_010784]	chr2:91770052-91769993	7.95	10.10	0.23
359	A_51_P360492	NM_008567	Mcm6	Mus musculus minichromosome maintenance deficient 6 (MIS5 homolog, S. pombe) (S. cerevisiae) (Mcm6), mRNA [NM_008567]	chr1:130228237-130228178	9.19	11.35	0.22
360	A_51_P195506	NM_007778	Csf1	Mus musculus colony stimulating factor 1 (macrophage) (Csf1), transcript variant 1, mRNA [NM_007778]	chr3:107544392-107544333	6.17	8.32	0.22
361	A_55_P2072373	NM_010833	Msn	Mus musculus moesin (Msn), mRNA [NM_010833]	chrX:93363832-93363891	9.78	11.93	0.22
362	A_55_P2089223	NM_001177602	Ak4	Mus musculus adenylate kinase 4 (Ak4), nuclear gene encoding mitochondrial protein, transcript variant 1, mRNA [NM_001177602]	chr4:101139464-101139523	9.27	11.42	0.22
363	A_51_P267239	NM_019980	Litaf	Mus musculus LPS-induced TN factor (Litaf), mRNA [NM_019980]	chr16:10959721-10959662	7.80	9.95	0.22
364	A_52_P387009	NM_028133	Egln3	Mus musculus EGL nine homolog 3 (C. elegans) (Egln3), mRNA [NM_028133]	chr12:55281012-55280953	8.39	10.55	0.22
365	A_52_P356698	NM_013665	Shox2	Mus musculus short stature homeobox 2 (Shox2), mRNA [NM_013665]	chr3:66779814-66779435	9.24	11.39	0.22
366	A_55_P1983733	NM_027406	Aldh1l1	Mus musculus aldehyde dehydrogenase 1 family, member L1 (Aldh1l1), mRNA [NM_027406]	chr6:90549091-90549150	8.15	10.30	0.22
367	A_52_P329451	NM_010777	Mbp	Mus musculus myelin basic protein (Mbp), transcript variant 7, mRNA [NM_010777]	chr18:82742266-82744570	8.33	10.49	0.22
368	A_55_P1967291	NM_144818	Ncaph	Mus musculus non-SMC condensin I complex, subunit H (Ncaph), mRNA [NM_144818]	chr2:126929684-126929625	6.89	9.05	0.22
369	A_55_P2293007	AK139019	AI662175	Mus musculus adult male aorta and vein cDNA, RIKEN full-length enriched library, clone:A530078F23 product:unclassifiable, full insert sequence. [AK139019]	chr16:19231495-19231436	5.63	7.79	0.22
370	A_55_P2397240	AK046698	B430316J06Rik	Mus musculus 4 days neonate male adipose cDNA, RIKEN full-length enriched library, clone:B430316J06 product:unclassifiable, full insert sequence. [AK046698]	chr6:93867818-93867759	6.07	8.24	0.22
371	A_30_P01031494	chr6:52046805-52072744_R		lincRNA:chr6:52046805-52072744 reverse strand	chr6:52056814-52056755	5.87	8.04	0.22
372	A_30_P01021588	H19		lincRNA:chr7:149761435-149764019 reverse strand	chr7:149761752-149761693	10.35	12.51	0.22
373	A_51_P513530	NM_017407	Spag5	Mus musculus sperm associated antigen 5 (Spag5), mRNA [NM_017407]	chr11:78135655-78135714	6.63	8.80	0.22
374	A_55_P2158314	ENSMUST00000097340		Mus musculus adult male adrenal gland cDNA, RIKEN full-length enriched library, clone:7330403C04 product:unclassifiable, full insert sequence. [AK078618]	chr17:35109039-35109098	5.93	8.10	0.22

375	A_52_P545810	NM_001111311	Lrrfip1	Mus musculus leucine rich repeat (in FLII) interacting protein 1 (Lrrfip1), transcript variant 1, mRNA [NM_001111311]	chr1:93013805-93013864	7.53	9.70	0.22
376	A_55_P2099560	NM_031159	Apobec1	Mus musculus apolipoprotein B mRNA editing enzyme, catalytic polypeptide 1 (Apobec1), transcript variant 1, mRNA [NM_031159]	chr6:122527967-122527908	6.23	8.41	0.22
377	A_66_P121459	NM_007681	Cenpa	Mus musculus centromere protein A (Cenpa), mRNA [NM_007681]	chr5:30974916-30975386	8.48	10.66	0.22
378	A_55_P2083197	NM_001013813	Maml2	Mus musculus mastermind like 2 (Drosophila) (Maml2), transcript variant 1, mRNA [NM_001013813]	chr9:13424194-13424253	7.59	9.77	0.22
379	A_55_P1983468	NM_009805	Cflar	Mus musculus CASP8 and FADD-like apoptosis regulator (Cflar), transcript variant 2, mRNA [NM_009805]	chr1:58789994-58790053	5.74	7.92	0.22
380	A_51_P496720	NM_019448	Dnmt3l	Mus musculus DNA (cytosine-5-)-methyltransferase 3-like (Dnmt3l), transcript variant 1, mRNA [NM_019448]	chr10:77526112-77526171	6.20	8.38	0.22
381	A_52_P420466	NM_175660	Hist1h2ab	Mus musculus histone cluster 1, H2ab (Hist1h2ab), mRNA [NM_175660]	chr13:23843392-23843451	9.52	11.71	0.22
382	A_51_P463765	NM_011595	Timp3	Mus musculus tissue inhibitor of metalloproteinase 3 (Timp3), mRNA [NM_011595]	chr10:85811696-85811755	7.99	10.17	0.22
383	A_51_P258493	NM_011067	Per3	Mus musculus period homolog 3 (Drosophila) (Per3), mRNA [NM_011067]	chr4:150379315-150379256	7.83	10.02	0.22
384	A_52_P593212	ENSMUST00000114435		RIKEN cDNA 5730446D14 gene Gene [Source:MGI Symbol;Acc:MGI:1913890] [ENSMUST00000114435]	chr6:52119107-52119166	8.33	10.52	0.22
385	A_52_P338605	XR_032468	Gm6639	PREDICTED: Mus musculus hypothetical protein LOC625963 (LOC625963), misc RNA [XR_032468]	chr3:35526429-35526370	5.67	7.86	0.22
386	A_55_P1961395	NM_010329	Pdpn	Mus musculus podoplanin (Pdpn), mRNA [NM_010329]	chr4:142857398-142857339	11.25	13.44	0.22
387	A_55_P2274592	NM_001163634	Wnt7b	Mus musculus wingless-related MMTV integration site 7B (Wnt7b), transcript variant 2, mRNA [NM_001163634]	chr15:85366955-85366896	6.75	8.94	0.22
388	A_51_P123405	NM_009772	Bub1	Mus musculus budding uninhibited by benzimidazoles 1 homolog (S. cerevisiae) (Bub1), transcript variant 2, mRNA [NM_009772]	chr2:127627047-127626988	6.29	8.48	0.22
389	A_65_P10913	NM_009367	Tgfb2	Mus musculus transforming growth factor, beta 2 (Tgfb2), mRNA [NM_009367]	chr1:188449392-188449333	7.40	9.59	0.22
390	A_55_P2129920	NM_001081417	Chd7	Mus musculus chromodomain helicase DNA binding protein 7 (Chd7), mRNA [NM_001081417]	chr4:8793852-8793911	11.07	13.26	0.22
391	A_55_P2429480	NM_027435	Atad2	Mus musculus ATPase family, AAA domain containing 2 (Atad2), mRNA [NM_027435]	chr15:57957644-57957367	7.03	9.23	0.22
392	A_55_P2170681	NM_026560	Cdca8	Mus musculus cell division cycle associated 8 (Cdca8), mRNA [NM_026560]	chr4:124599304-124599245	8.89	11.09	0.22

393	A_52_P53906	NM_009829	Ccnd2	Mus musculus cyclin D2 (Ccnd2), mRNA [NM_009829]	chr6:127098755-127098696	10.23	12.43	0.22
394	A_55_P2043921	NM_010441	Hmga2	Mus musculus high mobility group AT-hook 2 (Hmga2), mRNA [NM_010441]	chr10:119800680-119800621	7.01	9.21	0.22
395	A_51_P208769	NM_133719	Metrn	Mus musculus meteorin, glial cell differentiation regulator (Metrn), mRNA [NM_133719]	chr17:25931976-25931917	7.44	9.64	0.22
396	A_52_P227267	NM_178405	Atp1a2	Mus musculus ATPase, Na+/K+ transporting, alpha 2 polypeptide (Atp1a2), mRNA [NM_178405]	chr1:174208729-174208670	7.82	10.03	0.22
397	A_55_P2149983	NM_001110824	Foxp4	Mus musculus forkhead box P4 (Foxp4), transcript variant 1, mRNA [NM_001110824]	chr17:48004778-48004719	8.53	10.74	0.22
398	A_51_P440743	NM_009886	Celsr1	Mus musculus cadherin, EGF LAG seven-pass G-type receptor 1 (flamingo homolog, Drosophila) (Celsr1), mRNA [NM_009886]	chr15:85729440-85729381	7.66	9.88	0.22
399	A_55_P1958464	XR_031621	Gm9252	PREDICTED: Mus musculus similar to Phosphoglycerate dehydrogenase (LOC677380), misc RNA [XR_031621]	chr7:48922020-48921961	6.80	9.02	0.22
400	A_55_P2065991	NM_016740	S100a11	Mus musculus S100 calcium binding protein A11 (calgizzarin) (S100a11), mRNA [NM_016740]	chr3:93330022-93330081	8.75	10.97	0.21
401	A_52_P489295	NM_009621	Adamts1	Mus musculus a disintegrin-like and metallopeptidase (reprolysin type) with thrombospondin type 1 motif, 1 (Adamts1), mRNA [NM_009621]	chr16:85795103-85795044	8.15	10.37	0.21
402	A_55_P2064962	NM_010329	Pdpn	Mus musculus podoplanin (Pdpn), mRNA [NM_010329]	chr4:142859146-142858329	10.01	12.23	0.21
403	A_55_P2425938	AK079380	2810468N07Rik	Mus musculus 16 days neonate cerebellum cDNA, RIKEN full-length enriched library, clone:9630053J03 product:unclassifiable, full insert sequence. [AK079380]	chr17:25711920-25711979	7.02	9.24	0.21
404	A_55_P2002903	NM_022315	Smoc2	Mus musculus SPARC related modular calcium binding 2 (Smoc2), mRNA [NM_022315]	chr17:14541664-14541723	6.32	8.54	0.21
405	A_55_P2115235	NM_199195	Bckdhb	Mus musculus branched chain ketoacid dehydrogenase E1, beta polypeptide (Bckdhb), nuclear gene encoding mitochondrial protein, mRNA [NM_199195]	chr9:84017787-84017846	8.53	10.76	0.21
406	A_55_P2071846	XR_032130	Gm8681	PREDICTED: Mus musculus similar to high mobility group protein B2 (LOC667519), misc RNA [XR_032130]	chr10:25552875-25552816	7.52	9.74	0.21
407	A_55_P1988699	NM_001110843	Cacna2d1	Mus musculus calcium channel, voltage-dependent, alpha2/delta subunit 1 (Cacna2d1), transcript variant a, mRNA [NM_001110843]	chr5:15880152-15880211	8.57	10.79	0.21
408	A_55_P2064547	BC022182	Tuba1c	Mus musculus tubulin, alpha 1C, mRNA (cDNA clone MGC:14067 IMAGE:3661220), complete cds. [BC022182]	chr15:98868488-98868547	10.79	13.02	0.21

409	A_52_P552665	NM_008057	Fzd7	Mus musculus frizzled homolog 7 (Drosophila) (Fzd7), mRNA [NM_008057]	chr1:59543192-59543251	7.47	9.70	0.21
410	A_51_P379373	NM_145381	Lactb2	Mus musculus lactamase, beta 2 (Lactb2), mRNA [NM_145381]	chr1:13616246-13616187	7.02	9.25	0.21
411	A_55_P2165234	NM_001081961	2300005B03Rik	Mus musculus RIKEN cDNA 2300005B03 gene (2300005B03Rik), mRNA [NM_001081961]	chr15:74573328-74573269	8.25	10.48	0.21
412	A_52_P261322	NM_198294	Tanc1	Mus musculus tetratricopeptide repeat, ankyrin repeat and coiled-coil containing 1 (Tanc1), mRNA [NM_198294]	chr2:59681626-59681685	6.20	8.44	0.21
413	A_55_P1979457	NM_016764	Prdx4	Mus musculus peroxiredoxin 4 (Prdx4), mRNA [NM_016764]	chrX:151758530-151758471	7.45	9.69	0.21
414	A_55_P2143041	NM_016966	Phgdh	Mus musculus 3-phosphoglycerate dehydrogenase (Phgdh), mRNA [NM_016966]	chr3:98117361-98117303	6.78	9.02	0.21
415	A_52_P449417	NM_177545	Vangl1	Mus musculus vang-like 1 (van gogh, Drosophila) (Vangl1), mRNA [NM_177545]	chr3:101960779-101960720	7.65	9.91	0.21
416	A_55_P2038007	NM_007791	Csrp1	Mus musculus cysteine and glycine-rich protein 1 (Csrp1), mRNA [NM_007791]	chr1:137648747-137648806	7.36	9.62	0.21
417	A_51_P472829	NM_145144	Aif1l	Mus musculus allograft inflammatory factor 1-like (Aif1l), mRNA [NM_145144]	chr2:31828806-31828865	8.76	11.01	0.21
418	A_52_P433643	NM_170599	Igsf11	Mus musculus immunoglobulin superfamily, member 11 (Igsf11), mRNA [NM_170599]	chr16:39024960-39025019	6.59	8.84	0.21
419	A_51_P113182	NM_013584	Lifr	Mus musculus leukemia inhibitory factor receptor (Lifr), transcript variant 1, mRNA [NM_013584]	chr15:7141150-7141209	7.52	9.78	0.21
420	A_55_P1979082	NM_001113331	Shc1	Mus musculus src homology 2 domain-containing transforming protein C1 (Shc1), transcript variant 1, mRNA [NM_001113331]	chr3:89233868-89233927	7.96	10.22	0.21
421	A_51_P100174	NM_008613	Mns1	Mus musculus meiosis-specific nuclear structural protein 1 (Mns1), mRNA [NM_008613]	chr9:72306274-72306333	6.71	8.98	0.21
422	A_51_P337195	NM_008952	Pipox	Mus musculus pipecolic acid oxidase (Pipox), mRNA [NM_008952]	chr11:77694332-77694273	9.15	11.41	0.21
423	A_51_P159503	ENSMUST00000093902		ring finger protein 213 Gene [Source:MGI (curated);Acc:MGI:1289196] [ENSMUST00000093902]	chr11:119348605-119348664	7.31	9.58	0.21
424	A_55_P2362601	AK078562	D4Ertd681e	Mus musculus 11 days embryo gonad cDNA, RIKEN full-length enriched library, clone:7030402E17 product:DNA segment, Chr 4, ERATO Doi 681, expressed, full insert sequence. [AK078562]	chr4:97560763-97560822	6.11	8.38	0.21
425	A_55_P2137309	NM_153143	Kctd11	Mus musculus potassium channel tetramerisation domain containing 11 (Kctd11), mRNA [NM_153143]	chr11:69691826-69691767	7.66	9.93	0.21

426	A_52_P220879	NM_009373	Tgm2	Mus musculus transglutaminase 2, C polypeptide (Tgm2), mRNA [NM_009373]	chr2:157942334-157942275	6.59	8.86	0.21
427	A_55_P2097962	XM_001481064	9030622O22Rik	PREDICTED: Mus musculus hypothetical protein LOC100048732 (LOC100048732), mRNA [XM_001481064]	chr2:147798641-147798582	9.07	11.35	0.21
428	A_55_P2128734	NM_146131	Pbxip1	Mus musculus pre-B-cell leukemia transcription factor interacting protein 1 (Pbxip1), mRNA [NM_146131]	chr3:89252808-89252867	7.27	9.55	0.21
429	A_55_P2045208	NM_007866	Dll3	Mus musculus delta-like 3 (Drosophila) (Dll3), mRNA [NM_007866]	chr7:29079120-29079061	10.16	12.45	0.21
430	A_55_P1995205	NM_011623	Top2a	Mus musculus topoisomerase (DNA) II alpha (Top2a), mRNA [NM_011623]	chr11:98855581-98855522	5.97	8.25	0.20
431	A_55_P2010871	NM_001008231	Daam2	Mus musculus dishevelled associated activator of morphogenesis 2 (Daam2), mRNA [NM_001008231]	chr17:49595451-49595392	6.21	8.50	0.20
432	A_55_P1988083	NM_145150	Prc1	Mus musculus protein regulator of cytokinesis 1 (Prc1), mRNA [NM_145150]	chr7:87461048-87461107	6.24	8.53	0.20
433	A_55_P2126192	NM_010195	Lgr5	Mus musculus leucine rich repeat containing G protein coupled receptor 5 (Lgr5), mRNA [NM_010195]	chr10:114887462-114887403	6.29	8.58	0.20
434	A_55_P2064676	XR_034463	LOC100047967	PREDICTED: Mus musculus similar to growth arrest-specific 2 like 3 (LOC100047967), misc RNA [XR_034463]	chr10:88872449-88872390	6.19	8.48	0.20
435	A_30_P01027457	chr1:34734249-34786649_R		lincRNA:chr1:34734249-34786649 reverse strand	chr1:34780727-34780668	8.86	11.16	0.20
436	A_55_P2143204	NM_146131	Pbxip1	Mus musculus pre-B-cell leukemia transcription factor interacting protein 1 (Pbxip1), mRNA [NM_146131]	chr3:89254114-89254173	8.83	11.14	0.20
437	A_55_P1966432	NM_010358	Gstm1	Mus musculus glutathione S-transferase, mu 1 (Gstm1), mRNA [NM_010358]	chr3:107815654-107815595	7.74	10.05	0.20
438	A_30_P01022240	chr6:134875724-134931975_R		lincRNA:chr6:134875724-134931975 reverse strand	chr6:134899205-134899146	5.85	8.16	0.20
439	A_55_P2062836	NM_001013026	Ttf2	Mus musculus transcription termination factor, RNA polymerase II (Ttf2), mRNA [NM_001013026]	chr3:100742843-100742784	6.84	9.15	0.20
440	A_55_P2116924	NM_001037999	Dbi	Mus musculus diazepam binding inhibitor (Dbi), transcript variant 1, mRNA [NM_001037999]	chr1:122009976-122009917	11.32	13.64	0.20
441	A_51_P492893	NM_028767	Foxp4	Mus musculus forkhead box P4 (Foxp4), transcript variant 3, mRNA [NM_028767]	chr17:48004632-48004573	6.22	8.53	0.20
442	A_52_P183826	NM_178788	Dctd	Mus musculus dCMP deaminase (Dctd), transcript variant 1, mRNA [NM_178788]	chr8:49197399-49222612	8.37	10.68	0.20
443	A_55_P1953459	NM_001171147	Yap1	Mus musculus yes-associated protein 1 (Yap1), transcript variant 1, mRNA [NM_001171147]	chr9:7932084-7932025	8.02	10.34	0.20
444	A_30_P01026088	chr2:33500594-33501874_F		lincRNA:chr2:33500594-33501874 forward strand	chr2:33501484-33501543	8.00	10.31	0.20
445	A_51_P290074	NM_021272	Fabp7	Mus musculus fatty acid binding protein 7, brain (Fabp7), mRNA [NM_021272]	chr10:57508039-57508098	11.04	13.36	0.20

446	A_30_P01030984	chr9:14460976-14488051_R		lincRNA:chr9:14460976-14488051 reverse strand	chr9:14484672-14484613	7.30	9.62	0.20
447	A_55_P1957249	NM_001146268	Pdgfrb	Mus musculus platelet derived growth factor receptor, beta polypeptide (Pdgfrb), transcript variant 1, mRNA [NM_001146268]	chr18:61244606-61244665	6.39	8.72	0.20
448	A_55_P2034625	ENSMUST00000063878		sema domain, transmembrane domain (TM), and cytoplasmic domain, (semaphorin) 6A Gene [Source:MGI (curated);Acc:MGI:1203727] [ENSMUST00000063878]	chr18:47436114-47436055	7.13	9.45	0.20
449	A_30_P01027443	chr16:46384974-46391331_F		lincRNA:chr16:46384974-46391331 forward strand	chr16:46388702-46388761	7.47	9.80	0.20
450	A_51_P504354	NM_025980	Nrarp	Mus musculus Notch-regulated ankyrin repeat protein (Nrarp), mRNA [NM_025980]	chr2:25038038-25038097	6.51	8.84	0.20
451	A_30_P01029839	chr3:36280098-36347798_F		lincRNA:chr3:36280098-36347798 forward strand	chr3:36347443-36347502	6.46	8.80	0.20
452	A_66_P139785	NM_080555	Ppap2b	Mus musculus phosphatidic acid phosphatase type 2B (Ppap2b), mRNA [NM_080555]	chr4:104905037-104905096	6.49	8.82	0.20
453	A_30_P01019611	chr7:82902248-82955248_F		lincRNA:chr7:82902248-82955248 forward strand	chr7:82925548-82925607	7.05	9.39	0.20
454	A_51_P282508	NM_007484	Rhoc	Mus musculus ras homolog gene family, member C (Rhoc), mRNA [NM_007484]	chr3:104597300-104597359	10.23	12.57	0.20
455	A_55_P2046245	NM_008235	Hes1	Mus musculus hairy and enhancer of split 1 (Drosophila) (Hes1), mRNA [NM_008235]	chr16:30067820-30067879	6.54	8.88	0.20
456	A_52_P357829	NM_001081125	Gli2	Mus musculus GLI-Kruppel family member GLI2 (Gli2), mRNA [NM_001081125]	chr1:120731314-120731255	6.66	9.01	0.20
457	A_55_P2037712	NM_133786	Smc4	Mus musculus structural maintenance of chromosomes 4 (Smc4), mRNA [NM_133786]	chr3:68838437-68838496	6.89	9.24	0.20
458	A_55_P2128668	NM_172301	Ccnb1	Mus musculus cyclin B1 (Ccnb1), mRNA [NM_172301]	chr13:101553508-101553449	8.07	10.42	0.20
459	A_66_P125660	NM_001114085	Nde1	Mus musculus nuclear distribution gene E homolog 1 (A nidulans) (Nde1), transcript variant b, mRNA [NM_001114085]	chr16:14169548-14169607	7.56	9.92	0.19
460	A_66_P135185	NM_007960	Etv1	Mus musculus ets variant gene 1 (Etv1), transcript variant 1, mRNA [NM_007960]	chr12:39593054-39593113	7.33	9.70	0.19
461	A_30_P01025818	chr19:5834117-5835940_R		lincRNA:chr19:5834117-5835940 reverse strand	chr19:5834662-5834603	7.41	9.79	0.19
462	A_55_P2084308	NM_010917	Nid1	Mus musculus nidogen 1 (Nid1), mRNA [NM_010917]	chr13:13604435-13604494	7.03	9.40	0.19
463	A_52_P520466	NM_197959	Kif18b	Mus musculus kinesin family member 18B (Kif18b), mRNA [NM_197959]	chr11:102767305-102767246	6.03	8.41	0.19
464	A_55_P1954356	NM_025905	Ttc23	Mus musculus tetratricopeptide repeat domain 23 (Ttc23), transcript variant 1, mRNA [NM_025905]	chr7:74871398-74871457	6.37	8.75	0.19

465	A_51_P151902	NM_145584	Spon1	Mus musculus spondin 1, (f-spondin) extracellular matrix protein (Spon1), mRNA [NM_145584]	chr7:121184084-121184143	11.25	13.63	0.19
466	A_55_P1968370	NM_001110337	Gprc5c	Mus musculus G protein-coupled receptor, family C, group 5, member C (Gprc5c), transcript variant 1, mRNA [NM_001110337]	chr11:114733870-114733929	6.59	8.98	0.19
467	A_30_P01021563	chr16:46384974-46391331_R		lincRNA:chr16:46384974-46391331 reverse strand	chr16:46388188-46388129	7.29	9.68	0.19
468	A_55_P2004248	NM_133237	Apcdd1	Mus musculus adenomatosis polyposis coli down-regulated 1 (Apcdd1), mRNA [NM_133237]	chr18:63112729-63112788	8.20	10.60	0.19
469	A_55_P2002572	NM_007940	Ephx2	Mus musculus epoxide hydrolase 2, cytoplasmic (Ephx2), mRNA [NM_007940]	chr14:66703273-66703214	5.82	8.22	0.19
470	A_55_P2145159	NR_015386	Six3os1	Mus musculus Six3 opposite strand transcript 1 (Six3os1), non-coding RNA [NR_015386]	chr17:86010690-86010631	7.13	9.53	0.19
471	A_55_P2128646	NM_020567	Gmnn	Mus musculus geminin (Gmnn), mRNA [NM_020567]	chr13:24843774-24843715	6.45	8.85	0.19
472	A_55_P2143946	NM_172523	Slc18a2	Mus musculus solute carrier family 18 (vesicular monoamine), member 2 (Slc18a2), mRNA [NM_172523]	chr19:59370106-59370165	10.04	12.44	0.19
473	A_30_P01020075	chr9:14460976-14488051_F		lincRNA:chr9:14460976-14488051 forward strand	chr9:14484789-14484848	7.20	9.61	0.19
474	A_51_P147123	NM_008744	Ntn1	Mus musculus netrin 1 (Ntn1), mRNA [NM_008744]	chr11:68040112-68040053	7.69	10.10	0.19
475	A_52_P65494	NM_027711	Iqgap2	Mus musculus IQ motif containing GTPase activating protein 2 (Iqgap2), mRNA [NM_027711]	chr13:96398940-96398055	6.92	9.34	0.19
476	A_55_P2007703	NM_001080945	Nanogpd	Mus musculus similar to Nanog homeobox (Nanogpd), mRNA [NM_001080945]	chr6:122663514-122663573	6.39	8.81	0.19
477	A_55_P1980551	NM_146122	Dennd1a	Mus musculus DENN/MADD domain containing 1A (Dennd1a), mRNA [NM_146122]	chr2:37654571-37654512	7.80	10.22	0.19
478	A_55_P2024953	NM_001081278	Tbc1d4	Mus musculus TBC1 domain family, member 4 (Tbc1d4), mRNA [NM_001081278]	chr14:101841636-101841577	6.79	9.22	0.19
479	A_55_P2024366	NM_183024	Raver2	Mus musculus ribonucleoprotein, PTB-binding 2 (Raver2), mRNA [NM_183024]	chr4:100824894-100824953	6.09	8.52	0.19
480	A_52_P37681	NM_012056	Fkbp9	Mus musculus FK506 binding protein 9 (Fkbp9), mRNA [NM_012056]	chr6:56829199-56829258	8.23	10.66	0.19
481	A_55_P1980636	NM_011497	Aurka	Mus musculus aurora kinase A (Aurka), mRNA [NM_011497]	chr2:172181785-172181726	7.61	10.05	0.18
482	A_51_P183894	NM_015798	Fbxo15	Mus musculus F-box protein 15 (Fbxo15), mRNA [NM_015798]	chr18:85150693-85150752	6.45	8.89	0.18
483	A_51_P501844	NM_175475	Cyp26b1	Mus musculus cytochrome P450, family 26, subfamily b, polypeptide 1 (Cyp26b1), transcript variant 1, mRNA [NM_175475]	chr6:84522011-84521952	8.99	11.43	0.18
484	A_51_P442964	NM_029617	Casc5	Mus musculus cancer susceptibility candidate 5 (Casc5), mRNA [NM_029617]	chr2:118914569-118919747	6.81	9.25	0.18

485	A_55_P1988246	XR_034120	LOC638183	PREDICTED: Mus musculus similar to High mobility group box 2 (LOC638183), misc RNA [XR_034120]	chr6:81791015-81790956	7.87	10.31	0.18
486	A_51_P451151	NM_026785	Ube2c	Mus musculus ubiquitin-conjugating enzyme E2C (Ube2c), mRNA [NM_026785]	chr2:164598016-164598075	6.78	9.22	0.18
487	A_51_P518841	NM_153111	Fev	Mus musculus FEV (ETS oncogene family) (Fev), mRNA [NM_153111]	chr1:74928319-74928260	9.87	12.32	0.18
488	A_55_P2108476	NM_144888	Mavs	Mus musculus mitochondrial antiviral signaling protein (Mavs), nuclear gene encoding mitochondrial protein, mRNA [NM_144888]	chr2:131073675-131073734	6.11	8.56	0.18
489	A_55_P2397599	AK047130	B930025B16Rik	Mus musculus 10 days neonate cerebellum cDNA, RIKEN full-length enriched library, clone:B930025B16 product:unclassifiable, full insert sequence. [AK047130]	chr14:120740823-120740882	7.82	10.27	0.18
490	A_55_P2092310	XR_035004	Gm9210	PREDICTED: Mus musculus similar to 3-phosphoglycerate dehydrogenase (LOC668506), misc RNA [XR_035004]	chr17:53337968-53338027	7.90	10.35	0.18
491	A_30_P01026417	chr2:33500594-33501874_F		lincRNA:chr2:33500594-33501874 forward strand	chr2:33501799-33501858	9.95	12.40	0.18
492	A_51_P155142	NM_026560	Cdca8	Mus musculus cell division cycle associated 8 (Cdca8), mRNA [NM_026560]	chr4:124595868-124595809	7.03	9.49	0.18
493	A_55_P2067146	XM_983011	LOC675032	PREDICTED: Mus musculus similar to polymyositis scleroderma overlap syndrome (PM-SCL) antigen 1 a, transcript variant 6 (LOC675032), mRNA [XM_983011]	chr3:36465081-36465140	7.87	10.34	0.18
494	A_55_P2000439	NM_001081306	Ptprz1	Mus musculus protein tyrosine phosphatase, receptor type Z, polypeptide 1 (Ptprz1), mRNA [NM_001081306]	chr6:23002114-23002173	9.12	11.58	0.18
495	A_55_P2097964	XM_001481064	9030622O22Rik	PREDICTED: Mus musculus hypothetical protein LOC100048732 (LOC100048732), mRNA [XM_001481064]	chr2:147814595-147814536	6.56	9.02	0.18
496	A_51_P479321	NM_080575	Acss1	Mus musculus acyl-CoA synthetase short-chain family member 1 (Acss1), nuclear gene encoding mitochondrial protein, mRNA [NM_080575]	chr2:150443930-150443871	9.33	11.80	0.18
497	A_30_P01020665	chr18:69923410-69965360_R		lincRNA:chr18:69923410-69965360 reverse strand	chr18:69926747-69926688	5.55	8.02	0.18
498	A_55_P2097869	NM_172862	Frem2	Mus musculus Fras1 related extracellular matrix protein 2 (Frem2), mRNA [NM_172862]	chr3:53318064-53318005	7.38	9.86	0.18
499	A_55_P2104769	NM_009482	Utf1	Mus musculus undifferentiated embryonic cell transcription factor 1 (Utf1), mRNA [NM_009482]	chr7:147130950-147131009	6.54	9.02	0.18
500	A_55_P2015782	NM_029815	Bcas1	Mus musculus breast carcinoma amplified sequence 1 (Bcas1), transcript variant 1, mRNA [NM_029815]	chr2:170172707-170172648	8.69	11.17	0.18

501	A_55_P2025033	NM_028760	Cep55	Mus musculus centrosomal protein 55 (Cep55), transcript variant 2, mRNA [NM_028760]	chr19:38146320-38146379	6.39	8.87	0.18
502	A_55_P2087944	XM_001472867	Gm15452	PREDICTED: Mus musculus similar to Cyclin-dependent kinases regulatory subunit 2 (CKS-2) (LOC100044764), mRNA [XM_001472867]	chr1:8781796-8781737	5.80	8.28	0.18
503	A_51_P415688	NM_025278	Gng12	Mus musculus guanine nucleotide binding protein (G protein), gamma 12 (Gng12), transcript variant 2, mRNA [NM_025278]	chr6:66967776-66967835	7.01	9.50	0.18
504	A_30_P01017632	chr17:95149413-95174473_F		lincRNA:chr17:95149413-95174473 forward strand	chr17:95174346-95174405	7.87	10.35	0.18
505	A_52_P28806	NM_008021	Foxm1	Mus musculus forkhead box M1 (Foxm1), mRNA [NM_008021]	chr6:128324783-128324842	7.92	10.41	0.18
506	A_55_P2062190	NM_010358	Gstm1	Mus musculus glutathione S-transferase, mu 1 (Gstm1), mRNA [NM_010358]	chr5:117082549-117082490	6.14	8.63	0.18
507	A_55_P1953533	ENSMUST00000084696		spondin 1, (f-spondin) extracellular matrix protein Gene [Source:MGI (curated);Acc:MGI:2385287] [ENSMUST00000084696]	chr7:121073331-121073390	7.62	10.11	0.18
508	A_66_P111660	NM_013602	Mt1	Mus musculus metallothionein 1 (Mt1), mRNA [NM_013602]	chr8:96704165-96704224	10.06	12.56	0.18
509	A_55_P2031781	NM_027174	Col22a1	Mus musculus collagen, type XXII, alpha 1 (Col22a1), mRNA [NM_027174]	chr15:71628965-71628906	6.59	9.08	0.18
510	A_51_P204402	NM_011369	Shcbp1	Mus musculus Shc SH2-domain binding protein 1 (Shcbp1), mRNA [NM_011369]	chr8:4736060-4736001	6.03	8.53	0.18
511	A_55_P2089219	XM_001476512	LOC100046616	PREDICTED: Mus musculus similar to aquaporin 5 (LOC100046616), mRNA [XM_001476512]	chr15:99425214-99425273	6.38	8.87	0.18
512	A_55_P2106175	NM_008102	Gch1	Mus musculus GTP cyclohydrolase 1 (Gch1), mRNA [NM_008102]	chr14:47775407-47775348	7.02	9.52	0.18
513	A_55_P2047168	NM_008714	Notch1	Mus musculus Notch gene homolog 1 (Drosophila) (Notch1), mRNA [NM_008714]	chr2:26314689-26314630	7.77	10.27	0.18
514	A_51_P410681	NM_018826	Irx5	Mus musculus Iroquois related homeobox 5 (Drosophila) (Irx5), mRNA [NM_018826]	chr8:94884923-94884982	6.73	9.23	0.18
515	A_55_P2178044	NM_010567	Inppl1	Mus musculus inositol polyphosphate phosphatase-like 1 (Inppl1), transcript variant 1, mRNA [NM_010567]	chr7:108971253-108971194	9.83	12.33	0.18
516	A_55_P2087265	ENSMUST00000084055	Gm7676	PREDICTED: Mus musculus predicted gene, EG665536 (EG665536), mRNA [XM_977607]	chr8:13896764-13896823	6.52	9.03	0.18
517	A_30_P01020733	chr8:67542714-67561694_F		lincRNA:chr8:67542714-67561694 forward strand	chr8:67552709-67552768	9.08	11.59	0.18
518	A_52_P529570	NM_198654	Nsl1	Mus musculus NSL1, MIND kinetochore complex component, homolog (S. cerevisiae) (Nsl1), mRNA [NM_198654]	chr1:192908350-192908409	6.99	9.51	0.17
519	A_55_P2064776	NR_024257	4930412O13Rik	Mus musculus RIKEN cDNA 4930412O13 gene (4930412O13Rik), non-coding RNA [NR_024257]	chr2:9808308-9808367	8.83	11.35	0.17

520	A_66_P112164	NM_144875	Rab7l1	Mus musculus RAB7, member RAS oncogene family-like 1 (Rab7l1), mRNA [NM_144875]	chr1:133769401-133769460	6.38	8.90	0.17
521	A_66_P115116	NM_133784	Wwtr1	Mus musculus WW domain containing transcription regulator 1 (Wwtr1), transcript variant 2, mRNA [NM_133784]	chr3:57260058-57259999	5.95	8.48	0.17
522	A_55_P1974587	NM_177545	Vangl1	Mus musculus vang-like 1 (van gogh, Drosophila) (Vangl1), mRNA [NM_177545]	chr3:101962189-101962130	5.68	8.21	0.17
523	A_55_P2032147	NM_139298	Wnt9a	Mus musculus wingless-type MMTV integration site 9A (Wnt9a), mRNA [NM_139298]	chr11:59146953-59147012	6.64	9.17	0.17
524	A_55_P2011385	XM_916287	LOC637082	PREDICTED: Mus musculus similar to TIFA, transcript variant 1 (LOC637082), mRNA [XM_916287]	chr3:127501256-127501315	6.04	8.58	0.17
525	A_55_P1985768	NM_053273	Ttyh2	Mus musculus tweety homolog 2 (Drosophila) (Ttyh2), mRNA [NM_053273]	chr11:114582192-114582251	7.04	9.58	0.17
526	A_55_P1984035	NM_019980	Litaf	Mus musculus LPS-induced TN factor (Litaf), mRNA [NM_019980]	chr16:10960976-10960917	6.39	8.93	0.17
527	A_52_P532227	NM_007901	S1pr1	Mus musculus sphingosine-1-phosphate receptor 1 (S1pr1), mRNA [NM_007901]	chr3:115414079-115414020	6.64	9.19	0.17
528	A_55_P1982563	NM_010117	Rhbdf1	Mus musculus rhomboid family 1 (Drosophila) (Rhbdf1), mRNA [NM_010117]	chr11:32109646-32109587	6.99	9.53	0.17
529	A_30_P01022315	chr1:34735032-34781146_F		lincRNA:chr1:34735032-34781146 forward strand	chr1:34781027-34781086	6.24	8.79	0.17
530	A_55_P1971599	NM_007529	Bcan	Mus musculus brevican (Bcan), transcript variant 1, mRNA [NM_007529]	chr3:87791516-87791457	7.47	10.01	0.17
531	A_55_P1982075	ENSMUST00000112781			chr14:4322715-4322656	6.66	9.21	0.17
532	A_55_P1972792	NM_009185	Stil	Mus musculus Scl/Tal1 interrupting locus (Stil), mRNA [NM_009185]	chr4:114715399-114715458	6.52	9.07	0.17
533	A_55_P1991770	NM_019417	Pdlim4	Mus musculus PDZ and LIM domain 4 (Pdlim4), mRNA [NM_019417]	chr11:53868493-53868434	8.78	11.33	0.17
534	A_51_P206346	NM_178606	Reep3	Mus musculus receptor accessory protein 3 (Reep3), mRNA [NM_178606]	chr10:66474330-66474271	9.36	11.91	0.17
535	A_52_P300730	NM_010441	Hmga2	Mus musculus high mobility group AT-hook 2 (Hmga2), mRNA [NM_010441]	chr10:119798798-119798739	7.04	9.59	0.17
536	A_55_P1997141	NM_008652	Mybl2	Mus musculus myeloblastosis oncogene-like 2 (Mybl2), mRNA [NM_008652]	chr2:162909345-162909404	8.01	10.57	0.17
537	A_51_P413910	NM_008257	Hmx3	Mus musculus H6 homeobox 3 (Hmx3), mRNA [NM_008257]	chr7:138688330-138688389	6.38	8.94	0.17
538	A_30_P01026323	chr4:54851872-54866706_R		lincRNA:chr4:54851872-54866706 reverse strand	chr4:54865947-54865888	6.21	8.76	0.17
539	A_55_P2124791	NM_001109991	Col18a1	Mus musculus collagen, type XVIII, alpha 1 (Col18a1), transcript variant 1, mRNA [NM_001109991]	chr10:76515849-76515790	6.39	8.95	0.17
540	A_30_P01032694	chr4:54851872-54866706_R		lincRNA:chr4:54851872-54866706 reverse strand	chr4:54865650-54865591	6.32	8.88	0.17

541	A_52_P162099	NM_001004140	Ckap2	Mus musculus cytoskeleton associated protein 2 (Ckap2), mRNA [NM_001004140]	chr8:23279443-23279384	8.09	10.65	0.17
542	A_55_P1956448	NM_021324	Ttyh1	Mus musculus tweety homolog 1 (Drosophila) (Ttyh1), transcript variant 2, mRNA [NM_021324]	chr7:4086862-4086921	10.52	13.08	0.17
543	A_55_P2124228	NM_028283	Uaca	Mus musculus uveal autoantigen with coiled-coil domains and ankyrin repeats (Uaca), mRNA [NM_028283]	chr9:60728111-60728170	6.24	8.81	0.17
544	A_30_P01018123	chr9:14460976-14488051_R		lincRNA:chr9:14460976-14488051 reverse strand	chr9:14484488-14484429	7.19	9.76	0.17
545	A_55_P2000454	ENSMUST00000024228		Putative uncharacterized protein Fragment [Source:UniProtKB/TrEMBL;Acc:Q9CSX2] [ENSMUST00000024228]	chr2:93151479-93151420	7.65	10.21	0.17
546	A_51_P500949	NM_133919	Aff1	Mus musculus AF4/FMR2 family, member 1 (Aff1), transcript variant 2, mRNA [NM_133919]	chr5:104276766-104276825	7.18	9.76	0.17
547	A_66_P124858	NM_001024474	Diras2	Mus musculus DIRAS family, GTP-binding RAS-like 2 (Diras2), mRNA [NM_001024474]	chr13:52599802-52599744	9.87	12.45	0.17
548	A_30_P01032999	chr4:54851872-54866706_R		lincRNA:chr4:54851872-54866706 reverse strand	chr4:54866220-54866161	6.63	9.22	0.17
549	A_30_P01031011	chr1:34734249-34786649_R		lincRNA:chr1:34734249-34786649 reverse strand	chr1:34780590-34780531	7.37	9.96	0.17
550	A_51_P324287	NM_024245	Kif23	Mus musculus kinesin family member 23 (Kif23), mRNA [NM_024245]	chr9:61767732-61767492	7.87	10.46	0.17
551	A_52_P527800	NM_145158	Emilin2	Mus musculus elastin microfibril interfacer 2 (Emilin2), mRNA [NM_145158]	chr17:71601697-71601638	7.49	10.09	0.16
552	A_55_P1969276	NM_020259	Hhip	Mus musculus Hedgehog-interacting protein (Hhip), mRNA [NM_020259]	chr8:82494339-82494280	6.36	8.96	0.16
553	A_51_P253803	NM_001081117	Mki67	Mus musculus antigen identified by monoclonal antibody Ki 67 (Mki67), mRNA [NM_001081117]	chr7:142881650-142881591	7.85	10.45	0.16
554	A_66_P111562	NM_007631	Ccnd1	Mus musculus cyclin D1 (Ccnd1), mRNA [NM_007631]	chr7:152116261-152116202	9.85	12.45	0.16
555	A_55_P1953646	NM_144955	Nkx6-1	Mus musculus NK6 homeobox 1 (Nkx6-1), mRNA [NM_144955]	chr5:102088378-102088319	8.05	10.66	0.16
556	A_51_P480119	NM_029942	Prelid2	Mus musculus PRELI domain containing 2 (Prelid2), mRNA [NM_029942]	chr18:42072068-42040925	7.93	10.54	0.16
557	A_52_P214630	NM_011448	Sox9	Mus musculus SRY-box containing gene 9 (Sox9), mRNA [NM_011448]	chr11:112648809-112648868	6.28	8.90	0.16
558	A_55_P2107681	XM_001475869	Gm7321	PREDICTED: Mus musculus similar to High mobility group protein B2 (High mobility group protein 2) (HMG-2) (LOC641258), mRNA [XM_001475869]	chr14:5420918-5420977	6.61	9.24	0.16
559	A_52_P281702	NM_010518	Igfbp5	Mus musculus insulin-like growth factor binding protein 5 (Igfbp5), mRNA [NM_010518]	chr1:72904888-72904829	11.67	14.30	0.16
560	A_55_P2118173	NM_010514	Igf2	Mus musculus insulin-like growth factor 2 (Igf2), transcript variant 1, mRNA [NM_010514]	chr7:149839720-149839661	10.73	13.37	0.16
561	A_55_P1961127	NR_001592	H19	Mus musculus H19 fetal liver mRNA (H19), non-coding RNA [NR_001592]	chr7:149761551-149761492	11.88	14.52	0.16

562	A_52_P634090	NM_013822	Jag1	Mus musculus jagged 1 (Jag1), mRNA [NM_013822]	chr2:136907492-136907433	7.73	10.37	0.16
563	A_51_P300618	NM_001163566	Crb2	Mus musculus crumbs homolog 2 (Drosophila) (Crb2), mRNA [NM_001163566]	chr2:37654440-37654499	6.10	8.75	0.16
564	A_51_P159453	NM_009252	Serpina3n	Mus musculus serine (or cysteine) peptidase inhibitor, clade A, member 3N (Serpina3n), mRNA [NM_009252]	chr12:105651918-105651977	5.91	8.55	0.16
565	A_55_P2137927	NM_001136055	Cd82	Mus musculus CD82 antigen (Cd82), transcript variant 2, mRNA [NM_001136055]	chr2:93260077-93260018	8.32	10.96	0.16
566	A_51_P434670	NM_021881	Qk	Mus musculus quaking (Qk), transcript variant 3, mRNA [NM_021881]	chr17:10403223-10403164	8.53	11.18	0.16
567	A_55_P2164534	NM_029766	Dtl	Mus musculus denticleless homolog (Drosophila) (Dtl), mRNA [NM_029766]	chr1:193361318-193361259	6.21	8.86	0.16
568	A_55_P2016623	XM_204772	Gm5068	PREDICTED: Mus musculus predicted gene, EG277089 (EG277089), mRNA [XM_204772]	chr12:75062366-75062425	6.66	9.32	0.16
569	A_52_P1092823	NM_010573	Irx1	Mus musculus Iroquois related homeobox 1 (Drosophila) (Irx1), mRNA [NM_010573]	chr13:72095794-72095735	8.92	11.57	0.16
570	A_30_P01031408	chr8:67552315-67561235_R		lincRNA:chr8:67552315-67561235 reverse strand	chr8:67552431-67552372	7.38	10.03	0.16
571	A_51_P129012	NM_009735	B2m	Mus musculus beta-2 microglobulin (B2m), mRNA [NM_009735]	chr2:121978401-121978460	6.79	9.46	0.16
572	A_51_P158210	NM_008564	Mcm2	Mus musculus minichromosome maintenance deficient 2 mitotin (S. cerevisiae) (Mcm2), mRNA [NM_008564]	chr6:88833678-88833619	7.77	10.44	0.16
573	A_55_P1996946	NM_023223	Cdc20	Mus musculus cell division cycle 20 homolog (S. cerevisiae) (Cdc20), mRNA [NM_023223]	chr4:118105593-118105534	7.61	10.29	0.16
574	A_51_P210350	NM_182959	Slc17a8	Mus musculus solute carrier family 17 (sodium-dependent inorganic phosphate cotransporter), member 8 (Slc17a8), mRNA [NM_182959]	chr10:89046218-89046159	7.60	10.29	0.15
575	A_52_P175376	NM_023755	Tcfcp2l1	Mus musculus transcription factor CP2-like 1 (Tcfcp2l1), mRNA [NM_023755]	chr1:120581646-120581705	6.45	9.14	0.15
576	A_52_P192596	NM_054053	Gpr98	Mus musculus G protein-coupled receptor 98 (Gpr98), mRNA [NM_054053]	chr13:81699773-81699714	7.71	10.41	0.15
577	A_55_P1973995	ENSMUST00000077710		predicted gene 6756 Gene [Source:MGI Symbol;Acc:MGI:3647509] [ENSMUST00000077710]	chr18:37081750-37081809	7.60	10.30	0.15
578	A_55_P2014304	NM_181404	Kank1	Mus musculus KN motif and ankyrin repeat domains 1 (Kank1), mRNA [NM_181404]	chr19:25508900-25508959	7.31	10.01	0.15
579	A_66_P115098	NM_001122953	Nfia	Mus musculus nuclear factor I/A (Nfia), transcript variant 3, mRNA [NM_001122953]	chr4:97680930-97680989	8.42	11.12	0.15
580	A_55_P2134236	NM_010446	Foxa2	Mus musculus forkhead box A2 (Foxa2), mRNA [NM_010446]	chr2:147868728-147868669	7.81	10.51	0.15
581	A_51_P520849	NM_009144	Sfrp2	Mus musculus secreted frizzled-related protein 2 (Sfrp2), mRNA [NM_009144]	chr3:83577864-83577923	8.45	11.15	0.15

582	A_51_P470328	NM_001042614	Sepp1	Mus musculus selenoprotein P, plasma, 1 (Sepp1), transcript variant 3, mRNA [NM_001042614]	chr15:3230418-3230477	9.29	11.99	0.15
583	A_55_P2056496	NM_009387	Tk1	Mus musculus thymidine kinase 1 (Tk1), mRNA [NM_009387]	chr11:117676904-117676845	6.24	8.94	0.15
584	A_55_P2039324	NM_007634	Ccnf	Mus musculus cyclin F (Ccnf), mRNA [NM_007634]	chr17:24360263-24360204	8.46	11.17	0.15
585	A_30_P01019037	chr19:5834117-5835940_R		lincRNA:chr19:5834117-5835940 reverse strand	chr19:5834602-5834543	6.64	9.35	0.15
586	A_52_P1037027	ENSMUST00000098718		Putative uncharacterized protein [Source:UniProtKB/TrEMBL;Acc:Q3V438] [ENSMUST00000098718]	chr8:67552607-67552548	9.40	12.11	0.15
587	A_55_P1991164	NM_133241	Mlc1	Mus musculus megalencephalic leukoencephalopathy with subcortical cysts 1 homolog (human) (Mlc1), mRNA [NM_133241]	chr15:88786395-88786336	6.77	9.48	0.15
588	A_55_P2175880	NM_019946	Mgst1	Mus musculus microsomal glutathione S-transferase 1 (Mgst1), mRNA [NM_019946]	chr6:138104846-138104905	6.56	9.29	0.15
589	A_55_P2146254	NM_001112715	Ifitm1	Mus musculus interferon induced transmembrane protein 1 (Ifitm1), transcript variant 2, mRNA [NM_001112715]	chr7:148155659-148155718	6.37	9.09	0.15
590	A_55_P1961014	NM_009150	Selenbp1	Mus musculus selenium binding protein 1 (Selenbp1), mRNA [NM_009150]	chr3:94748621-94748680	6.41	9.13	0.15
591	A_55_P2146314	NM_033652	Lmx1a	Mus musculus LIM homeobox transcription factor 1 alpha (Lmx1a), mRNA [NM_033652]	chr1:169778804-169778863	8.92	11.65	0.15
592	A_51_P504494	NM_007866	Dll3	Mus musculus delta-like 3 (Drosophila) (Dll3), mRNA [NM_007866]	chr7:29078822-29078763	9.54	12.27	0.15
593	A_66_P105175	NM_009738	Bche	Mus musculus butyrylcholinesterase (Bche), mRNA [NM_009738]	chr3:73439794-73439735	6.22	8.97	0.15
594	A_66_P134542	NM_028390	Anln	Mus musculus anillin, actin binding protein (Anln), mRNA [NM_028390]	chr9:22136744-22136685	5.94	8.69	0.15
595	A_55_P2358679	ENSMUST00000054384		tripartite motif-containing 56 Gene [Source:MGI (curated);Acc:MGI:2685298] [ENSMUST00000054384]	chr5:137581579-137581520	5.69	8.44	0.15
596	A_55_P2008061	NM_019923	Itpr2	Mus musculus inositol 1,4,5-triphosphate receptor 2 (Itpr2), transcript variant 1, mRNA [NM_019923]	chr6:146060064-146060005	6.20	8.95	0.15
597	A_55_P2059352	NM_001109991	Col18a1	Mus musculus collagen, type XVIII, alpha 1 (Col18a1), transcript variant 1, mRNA [NM_001109991]	chr10:76514985-76514926	6.91	9.67	0.15
598	A_30_P01023432	chr1:34734249-34786649_R		lincRNA:chr1:34734249-34786649 reverse strand	chr1:34780667-34780608	8.25	11.01	0.15
599	A_55_P2168823	XR_031967	Gm7669	PREDICTED: Mus musculus similar to 3-phosphoglycerate dehydrogenase (LOC665516), misc RNA [XR_031967]	chr8:13745855-13745796	7.44	10.20	0.15
600	A_55_P2070992	NM_009657	Aldoc	Mus musculus aldolase C, fructose-bisphosphate (Aldoc), mRNA [NM_009657]	chr11:78140198-78140257	9.34	12.10	0.15

601	A_30_P01030107	chr9:14460976-14488051_R		lincRNA:chr9:14460976-14488051 reverse strand	chr9:14484548-14484489	6.81	9.58	0.15
602	A_55_P2038358	NM_012006	Acot1	Mus musculus acyl-CoA thioesterase 1 (Acot1), mRNA [NM_012006]	chr12:85358561-85358620	9.06	11.83	0.15
603	A_52_P630867	NM_001033336	Abcc4	Mus musculus ATP-binding cassette, sub-family C (CFTR/MRP), member 4 (Abcc4), transcript variant 1, mRNA [NM_001033336]	chr14:118882163-118882104	7.73	10.51	0.15
604	A_52_P628067	NM_013538	Cdca3	Mus musculus cell division cycle associated 3 (Cdca3), mRNA [NM_013538]	chr6:124783233-124783292	6.40	9.18	0.15
605	A_55_P2115713	NM_010917	Nid1	Mus musculus nidogen 1 (Nid1), mRNA [NM_010917]	chr13:13602780-13602839	6.43	9.21	0.15
606	A_30_P01027939	chr1:71939741-71947891_F		lincRNA:chr1:71939741-71947891 forward strand	chr1:71940191-71940250	5.87	8.66	0.14
607	A_55_P2076846	NM_011565	Tead2	Mus musculus TEA domain family member 2 (Tead2), mRNA [NM_011565]	chr7:52488742-52488801	9.88	12.67	0.14
608	A_51_P206405	NM_001081306	Ptprz1	Mus musculus protein tyrosine phosphatase, receptor type Z, polypeptide 1 (Ptprz1), mRNA [NM_001081306]	chr6:23002046-23002106	8.90	11.70	0.14
609	A_51_P422934	NM_146954	Olfr535	Mus musculus olfactory receptor 535 (Olfr535), mRNA [NM_146954]	chr7:147678773-147678832	6.02	8.83	0.14
610	A_52_P318073	XR_033857	Gm4870	PREDICTED: Mus musculus similar to cyclin B1 (LOC231869), misc RNA [XR_033857]	chr5:144200191-144200250	6.24	9.06	0.14
611	A_51_P393968	NM_007701	Vsx2	Mus musculus visual system homeobox 2 (Vsx2), mRNA [NM_007701]	chr12:85936221-85936280	7.95	10.78	0.14
612	A_51_P334104	NM_007833	Dcn	Mus musculus decorin (Dcn), transcript variant 2, mRNA [NM_007833]	chr10:96980233-96980292	6.18	9.00	0.14
613	A_55_P2048588	NM_007659	Cdk1	Mus musculus cyclin-dependent kinase 1 (Cdk1), mRNA [NM_007659]	chr10:68801131-68801072	5.73	8.55	0.14
614	A_52_P163021	NM_182959	Slc17a8	Mus musculus solute carrier family 17 (sodium-dependent inorganic phosphate cotransporter), member 8 (Slc17a8), mRNA [NM_182959]	chr10:89037689-89037630	7.94	10.77	0.14
615	A_52_P223626	NM_016967	Olig2	Mus musculus oligodendrocyte transcription factor 2 (Olig2), mRNA [NM_016967]	chr16:91228470-91228529	8.80	11.64	0.14
616	A_30_P01029584	chr6:90915489-90940052_R		lincRNA:chr6:90915489-90940052 reverse strand	chr6:90938148-90938089	9.95	12.78	0.14
617	A_66_P117417	ENSMUST00000023335	Pvrl3	poliovirus receptor-related 3 Gene [Source:MGI (curated);Acc:MGI:1930171] [ENSMUST00000023335]	chr16:46394512-46394453	9.14	11.97	0.14
618	A_30_P01032100	chr8:67542714-67561694_F		lincRNA:chr8:67542714-67561694 forward strand	chr8:67552826-67552885	6.51	9.36	0.14
619	A_55_P2103706	XM_485921	Gm5593	PREDICTED: Mus musculus predicted gene, EG434175, transcript variant 1 (EG434175), mRNA [XM_485921]	chr13:101549579-101549520	5.96	8.80	0.14
620	A_55_P2089530	NM_010221	Fkbp10	Mus musculus FK506 binding protein 10 (Fkbp10), transcript variant 1, mRNA [NM_010221]	chr11:100286080-100286139	6.89	9.74	0.14

621	A_55_P2058962	NM_027290	Mcm10	Mus musculus minichromosome maintenance deficient 10 (S. cerevisiae) (Mcm10), mRNA [NM_027290]	chr2:4911844-4911785	6.24	9.10	0.14
622	A_52_P603038	NM_016968	Olig1	Mus musculus oligodendrocyte transcription factor 1 (Olig1), mRNA [NM_016968]	chr16:91271799-91271858	6.29	9.15	0.14
623	A_55_P1964348	NM_023608	Gdpd2	Mus musculus glycerophosphodiester phosphodiesterase domain containing 2 (Gdpd2), mRNA [NM_023608]	chrX:97934160-97934219	8.96	11.83	0.14
624	A_51_P496309	NM_001024918	Rfx4	Mus musculus regulatory factor X, 4 (influences HLA class II expression) (Rfx4), transcript variant 1, mRNA [NM_001024918]	chr10:84368464-84368523	6.19	9.06	0.14
625	A_55_P1985143	XR_030646	Gm8284	PREDICTED: Mus musculus similar to high mobility group protein B2 (LOC666779), misc RNA [XR_030646]	chr18:90700729-90700788	9.24	12.11	0.14
626	A_51_P452779	NM_133198	Pygl	Mus musculus liver glycogen phosphorylase (Pygl), mRNA [NM_133198]	chr12:71291914-71291855	7.02	9.90	0.14
627	A_55_P1964568	NM_001001979	Megf10	Mus musculus multiple EGF-like-domains 10 (Megf10), mRNA [NM_001001979]	chr18:57457056-57457115	8.04	10.92	0.14
628	A_51_P277431	NM_028804	Ccdc3	Mus musculus coiled-coil domain containing 3 (Ccdc3), mRNA [NM_028804]	chr2:5150942-5151001	6.14	9.03	0.13
629	A_30_P01026878	chr7:82902248-82955248_F		lincRNA:chr7:82902248-82955248 forward strand	chr7:82924473-82924532	6.25	9.15	0.13
630	A_55_P2110713	NM_007585	Anxa2	Mus musculus annexin A2 (Anxa2), mRNA [NM_007585]	chr9:69339532-69339591	8.56	11.46	0.13
631	A_55_P2139878	XR_033857	Gm4870	PREDICTED: Mus musculus similar to cyclin B1 (LOC231869), misc RNA [XR_033857]	chr5:144199908-144199967	7.32	10.22	0.13
632	A_55_P2165334	NM_023317	Nde1	Mus musculus nuclear distribution gene E homolog 1 (A nidulans) (Nde1), transcript variant a, mRNA [NM_023317]	chr16:14192844-14192903	7.06	9.97	0.13
633	A_55_P2056654	NM_145588	Kif22	Mus musculus kinesin family member 22 (Kif22), mRNA [NM_145588]	chr7:134171314-134171255	6.71	9.63	0.13
634	A_55_P2150029	ENSMUST00000075226		PREDICTED: Mus musculus similar to developmental pluripotency associated 5 (LOC100046088), mRNA [XM_001475553]	chrX:108363831-108363890	6.99	9.92	0.13
635	A_55_P2123502	NM_023844	Jam2	Mus musculus junction adhesion molecule 2 (Jam2), mRNA [NM_023844]	chr16:84816558-84816617	8.68	11.61	0.13
636	A_55_P2039284	NM_013560	Hspb1	Mus musculus heat shock protein 1 (Hspb1), mRNA [NM_013560]	chr5:136365366-136365425	7.70	10.64	0.13
637	A_55_P2059931	NM_001163577	Prom1	Mus musculus prominin 1 (Prom1), transcript variant 2, mRNA [NM_001163577]	chr5:44384920-44384861	10.21	13.14	0.13
638	A_55_P2111302	NM_007752	Cp	Mus musculus ceruloplasmin (Cp), transcript variant 2, mRNA [NM_007752]	chr3:19889071-19889130	6.06	9.00	0.13

639	A_55_P2152872	NM_054071	Fgfrl1	Mus musculus fibroblast growth factor receptor-like 1 (Fgfrl1), transcript variant 1, mRNA [NM_054071]	chr5:109135855-109135914	7.81	10.74	0.13
640	A_51_P374726	NM_008987	Ptx3	Mus musculus pentraxin related gene (Ptx3), mRNA [NM_008987]	chr3:66029245-66029304	8.57	11.52	0.13
641	A_30_P01029071	chr9:14460976-14488051_F		lincRNA:chr9:14460976-14488051 forward strand	chr9:14484433-14484492	7.46	10.41	0.13
642	A_52_P233305	NM_175501	Adamts12	Mus musculus a disintegrin-like and metallopeptidase (reprolysin type) with thrombospondin type 1 motif, 12 (Adamts12), mRNA [NM_175501]	chr15:11266072-11266131	7.71	10.67	0.13
643	A_52_P187940	NM_008494	Lfng	Mus musculus LFNG O-fucosylpeptide 3-beta-N-acetylglucosaminyltransferase (Lfng), mRNA [NM_008494]	chr5:141091224-141091283	6.70	9.67	0.13
644	A_55_P2067947	XM_983385	Dppa5b	PREDICTED: Mus musculus similar to developmental pluripotency associated 5 (LOC666367), mRNA [XM_983385]	chr9:78120096-78120155	6.15	9.14	0.13
645	A_30_P01022915	chr8:67542714-67561694_F		lincRNA:chr8:67542714-67561694 forward strand	chr8:67559601-67559660	6.86	9.86	0.12
646	A_55_P1965154	NM_025565	Spc25	Mus musculus SPC25, NDC80 kinetochore complex component, homolog (S. cerevisiae) (Spc25), mRNA [NM_025565]	chr2:69032012-69031953	7.23	10.23	0.12
647	A_51_P142744	NM_172294	Sulf1	Mus musculus sulfatase 1 (Sulf1), mRNA [NM_172294]	chr1:12850302-12850361	10.20	13.21	0.12
648	A_55_P2094729	NM_011437	Sox10	Mus musculus SRY-box containing gene 10 (Sox10), mRNA [NM_011437]	chr15:78985577-78985518	6.95	9.96	0.12
649	A_51_P389539	NM_054053	Gpr98	Mus musculus G protein-coupled receptor 98 (Gpr98), mRNA [NM_054053]	chr13:81234389-81234330	6.72	9.73	0.12
650	A_55_P2053933	NM_008259	Foxa1	Mus musculus forkhead box A1 (Foxa1), mRNA [NM_008259]	chr12:58641823-58641764	7.74	10.76	0.12
651	A_55_P2004179	NM_001113515	Col2a1	Mus musculus collagen, type II, alpha 1 (Col2a1), transcript variant 2, mRNA [NM_001113515]	chr15:97806130-97806071	6.13	9.16	0.12
652	A_55_P1994939	NM_008252	Hmgb2	Mus musculus high mobility group box 2 (Hmgb2), mRNA [NM_008252]	chr8:59992929-59992988	7.87	10.90	0.12
653	A_51_P449233	NM_172444	Thsd4	Mus musculus thrombospondin, type I, domain containing 4 (Thsd4), transcript variant 2, mRNA [NM_172444]	chr9:59818131-59818072	7.68	10.72	0.12
654	A_55_P2089710	NM_007904	Ednrb	Mus musculus endothelin receptor type B (Ednrb), transcript variant 1, mRNA [NM_007904]	chr14:104220929-104220870	7.74	10.79	0.12
655	A_51_P109840	NM_011707	Vtn	Mus musculus vitronectin (Vtn), mRNA [NM_011707]	chr11:78315393-78315665	7.97	11.02	0.12
656	A_55_P2078670	NM_144855	Cbs	Mus musculus cystathionine beta-synthase (Cbs), transcript variant 1, mRNA [NM_144855]	chr17:31749652-31749593	6.84	9.89	0.12
657	A_51_P495232	NM_027127	Gpx8	Mus musculus glutathione peroxidase 8 (putative) (Gpx8), mRNA [NM_027127]	chr13:113833272-113833213	6.28	9.34	0.12
658	A_55_P2105673	NM_010712	Lhx4	Mus musculus LIM homeobox protein 4 (Lhx4), mRNA [NM_010712]	chr1:157548915-157548856	6.64	9.71	0.12

659	A_52_P549827	NM_019946	Mgst1	Mus musculus microsomal glutathione S-transferase 1 (Mgst1), mRNA [NM_019946]	chr6:138104944-138105003	5.93	9.00	0.12
660	A_51_P235801	NM_007564	Zfp36l1	Mus musculus zinc finger protein 36, C3H type-like 1 (Zfp36l1), mRNA [NM_007564]	chr12:81209081-81209022	9.33	12.41	0.12
661	A_55_P2040170	NM_008885	Pmp22	Mus musculus peripheral myelin protein 22 (Pmp22), mRNA [NM_008885]	chr11:62972989-62973048	6.19	9.27	0.12
662	A_30_P01023186	chr6:52046805-52072744_R		lincRNA:chr6:52046805-52072744 reverse strand	chr6:52053020-52052961	6.12	9.20	0.12
663	A_51_P160673	NM_021487	Kcne1l	Mus musculus potassium voltage-gated channel, Isk-related family, member 1-like, pseudogene (Kcne1l), mRNA [NM_021487]	chrX:138739423-138739364	6.08	9.17	0.12
664	A_55_P2013336	NM_010790	Melk	Mus musculus maternal embryonic leucine zipper kinase (Melk), mRNA [NM_010790]	chr4:44377041-44377100	6.04	9.14	0.12
665	A_52_P476877	NM_001024918	Rfx4	Mus musculus regulatory factor X, 4 (influences HLA class II expression) (Rfx4), transcript variant 1, mRNA [NM_001024918]	chr10:84331133-84331590	8.03	11.13	0.12
666	A_55_P2073377	NM_001081117	Mki67	Mus musculus antigen identified by monoclonal antibody Ki 67 (Mki67), mRNA [NM_001081117]	chr7:142884823-142884764	8.00	11.11	0.12
667	A_55_P2083609	NM_015814	Dkk3	Mus musculus dickkopf homolog 3 (Xenopus laevis) (Dkk3), mRNA [NM_015814]	chr7:119259615-119259556	8.23	11.35	0.12
668	A_55_P1983768	NM_009689	Birc5	Mus musculus baculoviral IAP repeat-containing 5 (Birc5), transcript variant 1, mRNA [NM_009689]	chr11:117711112-117711171	7.70	10.81	0.12
669	A_55_P1955548	NM_009510	Ezr	Mus musculus ezrin (Ezr), mRNA [NM_009510]	chr17:6942666-6942607	7.02	10.14	0.12
670	A_55_P1996941	NM_026785	Ube2c	Mus musculus ubiquitin-conjugating enzyme E2C (Ube2c), mRNA [NM_026785]	chr2:164597496-164597555	8.59	11.71	0.11
671	A_55_P2077088	NR_028271	Phxr4	Mus musculus per-hexamer repeat gene 4 (Phxr4), non-coding RNA [NR_028271]	chr9:13234932-13234991	6.47	9.62	0.11
672	A_52_P577662	NM_007904	Ednrb	Mus musculus endothelin receptor type B (Ednrb), transcript variant 1, mRNA [NM_007904]	chr14:104214543-104214484	7.87	11.02	0.11
673	A_55_P1997569	NM_183248	Nkx6-2	Mus musculus NK6 homeobox 2 (Nkx6-2), transcript variant 1, mRNA [NM_183248]	chr7:146767178-146767119	7.80	10.95	0.11
674	A_30_P01018375	chr8:67542714-67561694_F		lincRNA:chr8:67542714-67561694 forward strand	chr8:67552649-67552708	8.55	11.71	0.11
675	A_52_P354373	XM_001481164	1190002F15Rik	PREDICTED: Mus musculus RIKEN cDNA 1190002F15 gene (1190002F15Rik), mRNA [XM_001481164]	chr6:134901431-134901490	6.85	10.01	0.11
676	A_55_P1952256	NM_172301	Ccnb1	Mus musculus cyclin B1 (Ccnb1), mRNA [NM_172301]	chr13:101551283-101551224	8.51	11.67	0.11
677	A_55_P1967149	NM_008393	Irx3	Mus musculus Iroquois related homeobox 3 (Drosophila) (Irx3), mRNA [NM_008393]	chr8:94322535-94322476	8.63	11.80	0.11

678	A_55_P2173982	NM_009104	Rrm2	Mus musculus ribonucleotide reductase M2 (Rrm2), mRNA [NM_009104]	chr12:25398824-25398883	7.49	10.67	0.11
679	A_30_P01028673	chr9:14460976-14488051_F		lincRNA:chr9:14460976-14488051 forward strand	chr9:14484590-14484649	7.25	10.45	0.11
680	A_55_P2000369	NM_009700	Aqp4	Mus musculus aquaporin 4 (Aqp4), mRNA [NM_009700]	chr18:15551423-15551364	6.32	9.52	0.11
681	A_55_P2163897	XM_001477254	Gm3088	PREDICTED: Mus musculus hypothetical protein LOC100041002 (LOC100041002), mRNA [XM_001477254]	chr2:29700592-29700533	6.76	9.97	0.11
682	A_51_P264695	NM_016669	Crym	Mus musculus crystallin, mu (Crym), mRNA [NM_016669]	chr7:127330029-127329970	6.24	9.45	0.11
683	A_51_P392687	NM_011701	Vim	Mus musculus vimentin (Vim), mRNA [NM_011701]	chr2:13504232-13504291	10.30	13.52	0.11
684	A_55_P2067533	NM_010446	Foxa2	Mus musculus forkhead box A2 (Foxa2), mRNA [NM_010446]	chr2:147868673-147868614	7.03	10.29	0.10
685	A_51_P302566	NM_172778	Maob	Mus musculus monoamine oxidase B (Maob), nuclear gene encoding mitochondrial protein, mRNA [NM_172778]	chrX:16286486-16286427	8.38	11.64	0.10
686	A_30_P01033125	chr8:67550064-67560987_R		lincRNA:chr8:67550064-67560987 reverse strand	chr8:67560853-67560794	7.21	10.48	0.10
687	A_55_P2129271	NM_011565	Tead2	Mus musculus TEA domain family member 2 (Tead2), mRNA [NM_011565]	chr7:52488930-52488989	8.57	11.84	0.10
688	A_51_P246317	NM_008630	Mt2	Mus musculus metallothionein 2 (Mt2), mRNA [NM_008630]	chr8:96697371-96697430	9.87	13.14	0.10
689	A_52_P213696	NM_027402	Fndc5	Mus musculus fibronectin type III domain containing 5 (Fndc5), mRNA [NM_027402]	chr4:128821749-128821808	6.54	9.82	0.10
690	A_55_P2065671	NM_172301	Ccnb1	Mus musculus cyclin B1 (Ccnb1), mRNA [NM_172301]	chr13:101548781-101548722	6.67	9.97	0.10
691	A_55_P2055597	NM_001136077	Enpp2	Mus musculus ectonucleotide pyrophosphatase/phosphodiesterase 2 (Enpp2), transcript variant 1, mRNA [NM_001136077]	chr15:54670980-54670921	7.59	10.88	0.10
692	A_55_P2016652	NM_007659	Cdk1	Mus musculus cyclin-dependent kinase 1 (Cdk1), mRNA [NM_007659]	chr10:68801112-68801053	6.26	9.56	0.10
693	A_51_P370615	NM_009718	Neurog2	Mus musculus neurogenin 2 (Neurog2), mRNA [NM_009718]	chr3:127337805-127337864	6.46	9.77	0.10
694	A_55_P2133220	NM_001013377	E130306D19Rik	Mus musculus RIKEN cDNA E130306D19 gene (E130306D19Rik), mRNA [NM_001013377]	chr4:43509077-43509018	6.60	9.92	0.10
695	A_55_P1983773	NM_001012273	Birc5	Mus musculus baculoviral IAP repeat-containing 5 (Birc5), transcript variant 3, mRNA [NM_001012273]	chr11:117716978-117717037	9.32	12.66	0.10
696	A_55_P2015585	NM_001101433	Zcchc24	Mus musculus zinc finger, CCHC domain containing 24 (Zcchc24), mRNA [NM_001101433]	chr14:26531185-26531126	6.94	10.27	0.10
697	A_52_P64356	NM_010097	Sparcl1	Mus musculus SPARC-like 1 (Sparcl1), mRNA [NM_010097]	chr5:104508208-104508149	9.41	12.76	0.10
698	A_52_P289213	NM_010928	Notch2	Mus musculus Notch gene homolog 2 (Drosophila) (Notch2), mRNA [NM_010928]	chr3:97953914-97953973	7.51	10.87	0.10

699	A_52_P387724	NM_001079822	Tcf7l1	Mus musculus transcription factor 7-like 1 (T cell specific, HMG box) (Tcf7l1), transcript variant 1, mRNA [NM_001079822]	chr6:72576436-72576377	6.06	9.42	0.10
700	A_55_P2002884	NM_001101433	Zcchc24	Mus musculus zinc finger, CCHC domain containing 24 (Zcchc24), mRNA [NM_001101433]	chr14:26531198-26531139	7.07	10.47	0.09
701	A_51_P511015	NM_010246	Fzd9	Mus musculus frizzled homolog 9 (Drosophila) (Fzd9), mRNA [NM_010246]	chr5:135724874-135724815	6.66	10.13	0.09
702	A_55_P2084332	NM_001159620	Pigp	Mus musculus phosphatidylinositol glycan anchor biosynthesis, class P (Pigp), transcript variant 5, mRNA [NM_001159620]	chr16:94580704-94580645	7.30	10.81	0.09
703	A_55_P2030373	NM_178405	Atp1a2	Mus musculus ATPase, Na+/K+ transporting, alpha 2 polypeptide (Atp1a2), mRNA [NM_178405]	chr1:174201917-174201858	6.87	10.40	0.09
704	A_52_P590535	NM_007992	Fbln2	Mus musculus fibulin 2 (Fbln2), transcript variant 1, mRNA [NM_007992]	chr6:91222118-91222177	6.88	10.41	0.09
705	A_55_P1987499	NM_013917	Pttg1	Mus musculus pituitary tumor-transforming gene 1 (Pttg1), transcript variant 2, mRNA [NM_013917]	chr11:43233860-43233801	7.87	11.41	0.09
706	A_55_P2044385	NM_028263	Fgfbp3	Mus musculus fibroblast growth factor binding protein 3 (Fgfbp3), mRNA [NM_028263]	chr19:36992177-36992118	8.90	12.44	0.09
707	A_55_P2042297	XR_031240	Gm5250	PREDICTED: Mus musculus similar to developmental pluripotency associated 5 (LOC383490), misc RNA [XR_031240]	chr1:13052538-13052597	6.69	10.24	0.09
708	A_55_P1953087	NM_008563	Mcm3	Mus musculus minichromosome maintenance deficient 3 (S. cerevisiae) (Mcm3), mRNA [NM_008563]	chr1:20793195-20793136	7.46	11.05	0.08
709	A_51_P220162	NM_008716	Notch3	Mus musculus Notch gene homolog 3 (Drosophila) (Notch3), mRNA [NM_008716]	chr17:32257907-32257848	8.91	12.53	0.08
710	A_51_P494125	NM_007431	Alpl	Mus musculus alkaline phosphatase, liver/bone/kidney (Alpl), mRNA [NM_007431]	chr4:137297716-137297658	6.67	10.37	0.08
711	A_51_P324934	XR_031705	LOC100045677	PREDICTED: Mus musculus similar to DNA replication licensing factor MCM3 (DNA polymerase alpha holoenzyme-associated protein P1) (P1-MCM3) (LOC100045677), misc RNA [XR_031705]	chr1:20793178-20793119	7.91	11.67	0.07
712	A_30_P01030588	chr8:67542714-67561694_F		lincRNA:chr8:67542714-67561694 forward strand	chr8:67560939-67560998	8.52	12.29	0.07
713	A_55_P2168667	NM_028030	Rbpms2	Mus musculus RNA binding protein with multiple splicing 2 (Rbpms2), mRNA [NM_028030]	chr9:65508259-65508318	7.24	11.06	0.07
714	A_51_P268697	NM_148938	Slc1a3	Mus musculus solute carrier family 1 (glial high affinity glutamate transporter), member 3 (Slc1a3), mRNA [NM_148938]	chr15:8584295-8584236	7.24	11.10	0.07

715	A_52_P588881	NM_001033484	Iqgap3	Mus musculus IQ motif containing GTPase activating protein 3 (Iqgap3), mRNA [NM_001033484]	chr3:87924765-87924824	6.43	10.36	0.07
716	A_51_P187679	NM_028946	Nhedc1	Mus musculus Na+/H+ exchanger domain containing 1 (Nhedc1), mRNA [NM_028946]	chr3:135060709-135060768	6.40	10.37	0.06
717	A_55_P2235911	AK075572	0610009E02Rik	Mus musculus adult male kidney cDNA, RIKEN full-length enriched library, clone:0610009E02 product:unclassifiable, full insert sequence. [AK075572]	chr2:26314780-26314839	7.28	11.34	0.06
718	A_55_P2028171	NM_025274	Dppa5a	Mus musculus developmental pluripotency associated 5A (Dppa5a), mRNA [NM_025274]	chr9:78214925-78214866	7.46	12.05	0.04
719	A_66_P138009	NR_033456	Gm4710	Mus musculus predicted gene 4710 (Gm4710), non-coding RNA [NR_033456]	chr17:76288283-76288342	6.58	11.59	0.03
720	A_55_P2056821	ENSMUST00000064349		RIKEN cDNA A330008L17 gene Gene [Source:MGI Symbol;Acc:MGI:2443215] [ENSMUST00000064349]	chr8:101946243-101946302	6.59	11.79	0.03

Table S7
MGE Lhx6-GFP^{+} vs ES Lhx6-GFP^{+}: Absolute Fold Change >= 4

No	Probe ID	Accession	Gene Symbol	Description	Genomic Coordinates	MGE Lhx6-GFP^{+}	ES Lhx6-GFP^{+}	Fold Change
1	A_52_P300376	NR_001570	Xist	Mus musculus inactive X specific transcripts (Xist), transcript variant 2, non-coding RNA [NR_001570]	chrX:100662233-100662174	14.38	5.90	355.53
2	A_55_P1967478	NM_008221	Hbb-y	Mus musculus hemoglobin Y, beta-like embryonic chain (Hbb-y), mRNA [NM_008221]	chr7:111000327-111000268	12.35	5.62	105.78
3	A_30_P01020783	XIST		lincRNA:chrX:100655711-100678572 reverse strand	chrX:100656081-100656022	12.44	5.83	97.61
4	A_52_P113537	NR_001463	Xist	Mus musculus inactive X specific transcripts (Xist), transcript variant 1, non-coding RNA [NR_001463]	chrX:100656348-100656289	11.56	5.20	82.55
5	A_30_P01032945	XIST		lincRNA:chrX:100655711-100678572 reverse strand	chrX:100656018-100655959	12.55	6.36	72.98
6	A_55_P1967481	NM_008221	Hbb-y	Mus musculus hemoglobin Y, beta-like embryonic chain (Hbb-y), mRNA [NM_008221]	chr7:111000507-111000448	12.38	6.29	68.22
7	A_30_P01030803	XIST		lincRNA:chrX:100655711-100678572 reverse strand	chrX:100656141-100656082	11.46	6.21	38.11
8	A_30_P01022001	XIST		lincRNA:chrX:100655711-100678572 reverse strand	chrX:100655958-100655899	11.31	6.15	35.71
9	A_55_P2026420	NM_010127	Pou6f1	Mus musculus POU domain, class 6, transcription factor 1 (Pou6f1), mRNA [NM_010127]	chr15:100405950-100405891	12.00	7.24	26.96
10	A_55_P1962299	NM_001083955	Hba-a2	Mus musculus hemoglobin alpha, adult chain 2 (Hba-a2), mRNA [NM_001083955]	chr11:32197245-32197304	10.41	5.78	24.86
11	A_66_P135185	NM_007960	Etv1	Mus musculus ets variant gene 1 (Etv1), transcript variant 1, mRNA [NM_007960]	chr12:39593054-39593113	11.65	7.33	20.04
12	A_55_P1964568	NM_001001979	Megf10	Mus musculus multiple EGF-like-domains 10 (Megf10), mRNA [NM_001001979]	chr18:57457056-57457115	12.13	8.04	17.07
13	A_55_P2006615	NM_015754	Rbbp9	Mus musculus retinoblastoma binding protein 9 (Rbbp9), mRNA [NM_015754]	chr2:144368242-144368183	10.53	6.45	16.96
14	A_55_P1978905	ENSMUST00000085379	Rpl26-ps2	PREDICTED: Mus musculus predicted gene, EG217600 (EG217600), mRNA [XM_138109]	chr12:58733766-58733707	10.77	6.83	15.40
15	A_55_P1986321	NM_007960	Etv1	Mus musculus ets variant gene 1 (Etv1), transcript variant 1, mRNA [NM_007960]	chr12:39594622-39594681	10.59	6.75	14.37

16	A_30_P01026500	chr4:90877414-90917198_F		lincRNA:chr4:90877414-90917198 forward strand	chr4:90916850-90916909	9.54	5.85	12.88
17	A_55_P2112584	NM_009322	Tbr1	Mus musculus T-box brain gene 1 (Tbr1), mRNA [NM_009322]	chr2:61652093-61652152	10.70	7.10	12.07
18	A_51_P160673	NM_021487	Kcne1l	Mus musculus potassium voltage-gated channel, Isk-related family, member 1-like, pseudogene (Kcne1l), mRNA [NM_021487]	chrX:138739423-138739364	9.60	6.08	11.47
19	A_55_P1967659	NM_030717	Lactb	Mus musculus lactamase, beta (Lactb), nuclear gene encoding mitochondrial protein, mRNA [NM_030717]	chr9:66803425-66803366	9.13	5.68	10.92
20	A_51_P185757	NM_001159344	Casz1	Mus musculus castor homolog 1, zinc finger (Drosophila) (Casz1), transcript variant 1, mRNA [NM_001159344]	chr4:148327349-148327408	10.03	6.74	9.80
21	A_30_P01023652	XIST		lincRNA:chrX:100655711-100678572 reverse strand	chrX:100655898-100655839	10.34	7.06	9.75
22	A_66_P116311	NM_008448	Kif5b	Mus musculus kinesin family member 5B (Kif5b), mRNA [NM_008448]	chr18:6202574-6202515	8.92	5.63	9.73
23	A_55_P2009640	NR_033553	1700048O20Rik	Mus musculus RIKEN cDNA 1700048O20 gene (1700048O20Rik), non-coding RNA [NR_033553]	chr9:121855717-121855776	9.64	6.36	9.68
24	A_55_P1962303	NM_008218	Hba-a1	Mus musculus hemoglobin alpha, adult chain 1 (Hba-a1), mRNA [NM_008218]	chr11:32183955-32184014	12.48	9.20	9.68
25	A_55_P2061164	XM_001471589	LOC100044317	PREDICTED: Mus musculus hypothetical protein LOC100044317 (LOC100044317), mRNA [XM_001471589]	chrX:133508445-133508386	9.79	6.56	9.35
26	A_55_P2071406	ENSMUST00000043259	Pde3a	phosphodiesterase 3A, cGMP inhibited Gene [Source:MGI Symbol;Acc:MGI:1860764] [ENSMUST00000043259]	chr6:141448762-141448821	10.52	7.30	9.31
27	A_55_P2016652	NM_007659	Cdk1	Mus musculus cyclin-dependent kinase 1 (Cdk1), mRNA [NM_007659]	chr10:68801112-68801053	9.45	6.26	9.16
28	A_51_P393897	NM_025837	Mpi	Mus musculus mannose phosphate isomerase (Mpi), mRNA [NM_025837]	chr9:57392444-57392385	10.08	6.90	9.07
29	A_55_P2302973	NM_011419	Kdm5d	Mus musculus lysine (K)-specific demethylase 5D (Kdm5d), mRNA [NM_011419]	chrY:277162-277221	8.58	5.42	8.93
30	A_55_P2026734	NM_009062	Rgs4	Mus musculus regulator of G-protein signaling 4 (Rgs4), mRNA [NM_009062]	chr1:171671668-171671609	10.21	7.09	8.68
31	A_52_P95759	NM_172788	Sh3rf3	Mus musculus SH3 domain containing ring finger 3 (Sh3rf3), mRNA [NM_172788]	chr10:58601558-58601617	10.02	6.92	8.55
32	A_65_P01834	NM_001113545	Lima1	Mus musculus LIM domain and actin binding 1 (Lima1), transcript variant a, mRNA [NM_001113545]	chr15:99610370-99610311	10.47	7.39	8.41

33	A_51_P517430	NM_007639	Cd1d1	Mus musculus CD1d1 antigen (Cd1d1), mRNA [NM_007639]	chr3:86799850-86799791	10.32	7.30	8.12
34	A_30_P01028487	chr9:121845920-121858120_R		lincRNA:chr9:121845920-121858120 reverse strand	chr9:121851061-121851002	9.37	6.39	7.92
35	A_51_P448178	NM_175519	Kctd8	Mus musculus potassium channel tetramerisation domain containing 8 (Kctd8), mRNA [NM_175519]	chr5:69501318-69501259	9.74	6.82	7.59
36	A_55_P2158227	NM_009640	Angpt1	Mus musculus angiopoietin 1 (Angpt1), mRNA [NM_009640]	chr15:42256394-42256335	8.50	5.57	7.59
37	A_30_P01026237	chr9:121845920-121858120_F		lincRNA:chr9:121845920-121858120 forward strand	chr9:121851028-121851087	8.62	5.70	7.56
38	A_52_P129428	NM_020510	Fzd2	Mus musculus frizzled homolog 2 (Drosophila) (Fzd2), mRNA [NM_020510]	chr11:102469045-102469104	9.03	6.11	7.53
39	A_30_P01030682	chr5:77084398-77086144_R		lincRNA:chr5:77084398-77086144 reverse strand	chr5:77084898-77084839	9.13	6.23	7.49
40	A_51_P206346	NM_178606	Reep3	Mus musculus receptor accessory protein 3 (Reep3), mRNA [NM_178606]	chr10:66474330-66474271	12.21	9.36	7.23
41	A_51_P163106	NM_175177	Bdh1	Mus musculus 3-hydroxybutyrate dehydrogenase, type 1 (Bdh1), nuclear gene encoding mitochondrial protein, transcript variant 1, mRNA [NM_175177]	chr16:31457378-31457437	10.15	7.30	7.23
42	A_55_P1973352	XM_001476722		PREDICTED: Mus musculus hypothetical protein LOC100041156 (LOC100041156), mRNA [XM_001476722]	chr8:93415492-93415551	9.68	6.85	7.10
43	A_55_P2205650	AK162965	2610507I01Rik	Mus musculus adult male spinal cord cDNA, RIKEN full-length enriched library, clone:A330105A21 product:unclassifiable, full insert sequence. [AK162965]	chr11:59011363-59011304	9.16	6.34	7.03
44	A_55_P1953273	NR_033506	Gm3893	Mus musculus predicted gene 3893 (Gm3893), non-coding RNA [NR_033506]	chrUn_random:553693-553634	11.71	8.93	6.87
45	A_55_P2143025	NM_013657	Sema3c	Mus musculus sema domain, immunoglobulin domain (Ig), short basic domain, secreted, (semaphorin) 3C (Sema3c), mRNA [NM_013657]	chr5:17235981-17236040	11.65	8.88	6.81
46	A_51_P301603	XM_979851	AI854517	PREDICTED: Mus musculus expressed sequence AI854517 (AI854517), mRNA [XM_979851]	chr7:86677283-86677342	12.34	9.57	6.80
47	A_55_P2104924	NM_172420	Ppp1r1c	Mus musculus protein phosphatase 1, regulatory (inhibitor) subunit 1C (Ppp1r1c), mRNA [NM_172420]	chr2:79548348-79548407	8.82	6.06	6.78

48	A_30_P01027192	chr1:87343550-87362900_F		lincRNA:chr1:87343550-87362900 forward strand	chr1:87354285-87354344	9.06	6.31	6.73
49	A_55_P2048588	NM_007659	Cdk1	Mus musculus cyclin-dependent kinase 1 (Cdk1), mRNA [NM_007659]	chr10:68801131-68801072	8.45	5.73	6.59
50	A_51_P316553	NM_010612	Kdr	Mus musculus kinase insert domain protein receptor (Kdr), mRNA [NM_010612]	chr5:76329368-76329309	8.46	5.76	6.50
51	A_30_P01030971	chr9:121845920-121858120_F		lincRNA:chr9:121845920-121858120 forward strand	chr9:121855465-121855524	10.14	7.45	6.45
52	A_30_P01031788	chr9:121845920-121858120_R		lincRNA:chr9:121845920-121858120 reverse strand	chr9:121848572-121848513	9.47	6.82	6.29
53	A_30_P01022360	chr3:126969590-126972626_R		lincRNA:chr3:126969590-126972626 reverse strand	chr3:126969650-126969591	8.51	5.86	6.28
54	A_55_P2295856	AA711038	AI594671	vt54g10.r1 Barstead mouse irradiated colon MPLRB7 Mus musculus cDNA clone IMAGE:1166946 5'. [AA711038]	chr11:39849827-39849768	8.55	5.91	6.24
55	A_55_P1959541	XR_034879	Gm14026	PREDICTED: Mus musculus similar to SET domain containing 3 (LOC545461), misc RNA [XR_034879]	chr2:128996852-128996911	11.61	8.99	6.14
56	A_55_P1955289	NM_183116	1110021L09Rik	Mus musculus RIKEN cDNA 1110021L09 gene (1110021L09Rik), mRNA [NM_183116]	chr10:23547671-23547730	9.42	6.81	6.13
57	A_51_P402994	NM_012008	Ddx3y	Mus musculus DEAD (Asp-Glu-Ala-Asp) box polypeptide 3, Y-linked (Ddx3y), mRNA [NM_012008]	chrY:598274-598215	8.11	5.50	6.12
58	A_52_P455428	NM_183427	Glra2	Mus musculus glycine receptor, alpha 2 subunit (Glra2), mRNA [NM_183427]	chrX:161567556-161567497	9.89	7.29	6.06
59	A_55_P2181753	NM_001122683	Bdh1	Mus musculus 3-hydroxybutyrate dehydrogenase, type 1 (Bdh1), transcript variant 2, mRNA [NM_001122683]	chr16:31457249-31457308	8.64	6.06	5.97
60	A_55_P2014100	NM_001177666	Gm7120	Mus musculus predicted gene 7120 (Gm7120), transcript variant 2, mRNA [NM_001177666]	chr13:120277695-120277754	9.10	6.53	5.95
61	A_55_P1994939	NM_008252	Hmgb2	Mus musculus high mobility group box 2 (Hmgb2), mRNA [NM_008252]	chr8:59992929-59992988	10.40	7.87	5.80
62	A_55_P1987176	NM_029536	Gpr165	Mus musculus G protein-coupled receptor 165 (Gpr165), mRNA [NM_029536]	chrX:93914626-93914685	9.45	6.92	5.79
63	A_55_P2112642	NM_011648	Tshr	Mus musculus thyroid stimulating hormone receptor (Tshr), transcript variant 1, mRNA [NM_011648]	chr12:92778221-92778280	8.76	6.23	5.77

64	A_55_P2087414	NM_011348	Sema3e	Mus musculus sema domain, immunoglobulin domain (Ig), short basic domain, secreted, (semaphorin) 3E (Sema3e), mRNA [NM_011348]	chr5:14256598-14256656	8.68	6.15	5.76
65	A_52_P222230	XM_001476722		PREDICTED: Mus musculus hypothetical protein LOC100041156 (LOC100041156), mRNA [XM_001476722]	chr8:93412002-93412061	11.46	8.93	5.76
66	A_52_P72587	NM_008859	Prkcq	Mus musculus protein kinase C, theta (Prkcq), mRNA [NM_008859]	chr2:11222026-11222085	8.70	6.19	5.73
67	A_55_P2007919	NM_001013785	Akr1c19	Mus musculus aldo-keto reductase family 1, member C19 (Akr1c19), mRNA [NM_001013785]	chr13:4247522-4247581	7.99	5.50	5.62
68	A_30_P01027247	chr9:121845920-121858120_F		lincRNA:chr9:121845920-121858120 forward strand	chr9:121855322-121855381	8.76	6.28	5.56
69	A_55_P1983508	NM_013613	Nr4a2	Mus musculus nuclear receptor subfamily 4, group A, member 2 (Nr4a2), transcript variant 1, mRNA [NM_013613]	chr2:56959696-56959637	10.09	7.62	5.53
70	A_52_P510107	NM_172527	Nudt15	Mus musculus nudix (nucleoside diphosphate linked moiety X)-type motif 15 (Nudt15), mRNA [NM_172527]	chr14:73919744-73919685	8.96	6.50	5.49
71	A_30_P01019516	chr7:86680928-86700632_F		lincRNA:chr7:86680928-86700632 forward strand	chr7:86700167-86700226	9.99	7.54	5.47
72	A_55_P1982325	NM_022565	Ndst4	Mus musculus N-deacetylase/N-sulfotransferase (heparin glucosaminyl) 4 (Ndst4), mRNA [NM_022565]	chr3:125427787-125427846	8.63	6.18	5.47
73	A_55_P1972040	NM_015760	Nox4	Mus musculus NADPH oxidase 4 (Nox4), mRNA [NM_015760]	chr7:94547025-94547084	8.21	5.76	5.45
74	A_30_P01018292	chr2:36112819-36114110_F		lincRNA:chr2:36112819-36114110 forward strand	chr2:36113149-36113208	8.39	5.96	5.42
75	A_55_P1956918	NM_011782	Adamts5	Mus musculus a disintegrin-like and metallopeptidase (reprolysin type) with thrombospondin type 1 motif, 5 (aggrecanase-2) (Adamts5), mRNA [NM_011782]	chr16:85858461-85858402	8.69	6.27	5.35
76	A_55_P2173982	NM_009104	Rrm2	Mus musculus ribonucleotide reductase M2 (Rrm2), mRNA [NM_009104]	chr12:25398824-25398883	9.91	7.49	5.34
77	A_51_P417321	NM_177832	Zfp236	Mus musculus zinc finger protein 236 (Zfp236), mRNA [NM_177832]	chr18:82765067-82765008	9.94	7.53	5.34
78	A_30_P01025988	chr18:6144775-6202105_R		lincRNA:chr18:6144775-6202105 reverse strand	chr18:6201017-6200958	8.14	5.76	5.21

79	A_51_P194230	NM_009573	Zic1	Mus musculus zinc finger protein of the cerebellum 1 (Zic1), mRNA [NM_009573]	chr9:91255484-91255425	13.74	11.36	5.20
80	A_55_P2083919	NM_175549	Robo2	Mus musculus roundabout homolog 2 (Drosophila) (Robo2), mRNA [NM_175549]	chr16:73894712-73894653	10.98	8.60	5.20
81	A_55_P2397240	AK046698	B430316J06Rik	Mus musculus 4 days neonate male adipose cDNA, RIKEN full-length enriched library, clone:B430316J06 product:unclassifiable, full insert sequence. [AK046698]	chr6:93867818-93867759	8.44	6.07	5.16
82	A_55_P1985070	NM_019413	Robo1	Mus musculus roundabout homolog 1 (Drosophila) (Robo1), mRNA [NM_019413]	chr16:73046190-73046249	11.15	8.80	5.09
83	A_51_P476960	NM_001081391	Csmd3	Mus musculus CUB and Sushi multiple domains 3 (Csmd3), mRNA [NM_001081391]	chr15:47781534-47781475	9.59	7.25	5.07
84	A_52_P661071	NR_003270	Snhg3	Mus musculus small nucleolar RNA host gene (non-protein coding) 3 (Snhg3), non-coding RNA [NR_003270]	chr4:131908064-131908005	9.95	7.62	5.02
85	A_55_P2177351	NM_008066	Gabra2	Mus musculus gamma-aminobutyric acid (GABA) A receptor, subunit alpha 2 (Gabra2), mRNA [NM_008066]	chr5:71352365-71352306	10.13	7.80	5.01
86	A_52_P361435	NM_175513	Zfp804a	Mus musculus zinc finger protein 804A (Zfp804a), mRNA [NM_175513]	chr2:82099768-82099827	8.00	5.68	4.98
87	A_30_P01032193	chr3:4797557-4798821_F		lincRNA:chr3:4797557-4798821 forward strand	chr3:4798762-4798821	8.52	6.21	4.97
88	A_51_P425680	NM_019826	Ivd	Mus musculus isovaleryl coenzyme A dehydrogenase (Ivd), nuclear gene encoding mitochondrial protein, mRNA [NM_019826]	chr2:118706998-118707057	8.49	6.18	4.96
89	A_55_P2026738	NM_009062	Rgs4	Mus musculus regulator of G-protein signaling 4 (Rgs4), mRNA [NM_009062]	chr1:171672547-171672488	11.66	9.35	4.95
90	A_55_P1987499	NM_013917	Pttg1	Mus musculus pituitary tumor-transforming gene 1 (Pttg1), transcript variant 2, mRNA [NM_013917]	chr11:43233860-43233801	10.18	7.87	4.95
91	A_55_P2337074	NM_183151	Mid1	Mus musculus midline 1 (Mid1), transcript variant 2, mRNA [NM_183151]	chrX:166428354-166428413	8.52	6.22	4.93
92	A_55_P2144285	NM_008710	Nnt	Mus musculus nicotinamide nucleotide transhydrogenase (Nnt), nuclear gene encoding mitochondrial protein, transcript variant 1, mRNA [NM_008710]	chr13:120124394-120124335	11.14	8.85	4.88

93	A_55_P2223851	ENSMUST00000104938		Novel protein [Source:UniProtKB/TrEMBL;Acc:Q5SVU9] [ENSMUST00000104938]	chr11:90124966-90125025	8.99	6.70	4.87
94	A_52_P468343	NM_001122683	Bdh1	Mus musculus 3-hydroxybutyrate dehydrogenase, type 1 (Bdh1), transcript variant 2, mRNA [NM_001122683]	chr16:31458195-31458254	10.86	8.58	4.87
95	A_55_P1995007	NM_181416	Arhgap11a	Mus musculus Rho GTPase activating protein 11A (Arhgap11a), mRNA [NM_181416]	chr2:113671708-113671649	8.66	6.38	4.86
96	A_55_P2305010	XR_035181	A730089K16Rik	PREDICTED: Mus musculus RIKEN cDNA A730089K16 gene (A730089K16Rik), misc RNA [XR_035181]	chr5:77085013-77084954	10.07	7.80	4.83
97	A_55_P1970715	XM_001480027	LOC100048332	PREDICTED: Mus musculus similar to a disintegrin-like and metalloprotease (reprolysin type) with thrombospondin type 1 motif, 5 (aggrecanase-2) (LOC100048332), mRNA [XM_001480027]	chr16:85858232-85858173	9.13	6.88	4.78
98	A_51_P338998	NM_008922	Prim2	Mus musculus DNA primase, p58 subunit (Prim2), mRNA [NM_008922]	chr1:33510792-33510733	8.85	6.60	4.77
99	A_30_P01019231	chr5:144517700-144526071_R		lincRNA:chr5:144517700-144526071 reverse strand	chr5:144525535-144525476	7.99	5.74	4.76
100	A_55_P2054362	XM_001472585	LOC100048875	PREDICTED: Mus musculus similar to chemokine receptor CX3CR1 (LOC100048875), mRNA [XM_001472585]	chr9:119957865-119957806	7.54	5.30	4.75
101	A_55_P2165234	NM_001081961	2300005B03Rik	Mus musculus RIKEN cDNA 2300005B03 gene (2300005B03Rik), mRNA [NM_001081961]	chr15:74573328-74573269	10.49	8.25	4.74
102	A_66_P116226	NM_001004361	5730494M16Rik	Mus musculus RIKEN cDNA 5730494M16 gene (5730494M16Rik), transcript variant 1, mRNA [NM_001004361]	chr18:25296893-25296834	10.05	7.80	4.74
103	A_55_P1959576	NM_178740	Slitrk4	Mus musculus SLIT and NTRK-like family, member 4 (Slitrk4), mRNA [NM_178740]	chrX:61522807-61522748	8.70	6.46	4.74
104	A_30_P01028522	chr5:144517700-144526071_R		lincRNA:chr5:144517700-144526071 reverse strand	chr5:144525685-144525626	9.71	7.47	4.72
105	A_55_P2027900	XM_001478553	LOC100047443	PREDICTED: Mus musculus similar to Gamma-aminobutyric-acid receptor subunit alpha-2 precursor (GABA(A) receptor subunit alpha-2) (LOC100047443), mRNA [XM_001478553]	chr5:71352334-71352275	10.07	7.84	4.71

106	A_55_P1962419	NM_026201	Ccar1	Mus musculus cell division cycle and apoptosis regulator 1 (Ccar1), mRNA [NM_026201]	chr10:62207274-62207215	9.46	7.23	4.70
107	A_30_P01025600	chr14:99728359-99728958_F		lincRNA:chr14:99728359-99728958 forward strand	chr14:99728669-99728728	9.31	7.08	4.70
108	A_52_P650379	NM_011499	Strap	Mus musculus serine/threonine kinase receptor associated protein (Strap), mRNA [NM_011499]	chr6:137699817-137699876	11.33	9.10	4.68
109	A_55_P2007964	NM_009987	Cx3cr1	Mus musculus chemokine (C-X3-C) receptor 1 (Cx3cr1), mRNA [NM_009987]	chr9:119957870-119957811	7.84	5.62	4.66
110	A_55_P1953894	NM_030026	Mccc2	Mus musculus methylcrotonoyl-Coenzyme A carboxylase 2 (beta) (Mccc2), nuclear gene encoding mitochondrial protein, mRNA [NM_030026]	chr13:100718567-100718508	8.76	6.54	4.64
111	A_30_P01025517	chr1:45066850-45095650_R		lincRNA:chr1:45066850-45095650 reverse strand	chr1:45091740-45091681	10.39	8.17	4.63
112	A_52_P251623	NM_001004180	BC057022	Mus musculus cDNA sequence BC057022 (BC057022), mRNA [NM_001004180]	chr5:115063126-115063185	9.81	7.61	4.62
113	A_55_P1965694	NM_008073	Gabrg2	Mus musculus gamma-aminobutyric acid (GABA) A receptor, subunit gamma 2 (Gabrg2), transcript variant 1, mRNA [NM_008073]	chr11:41725423-41725364	11.94	9.73	4.62
114	A_55_P1965101	NM_001081643	Xlr3b	Mus musculus X-linked lymphocyte-regulated 3B (Xlr3b), mRNA [NM_001081643]	chrX:70448087-70448146	8.98	6.78	4.61
115	A_55_P2075814	NM_177066	Tnni3k	Mus musculus TNNI3 interacting kinase (Tnni3k), mRNA [NM_177066]	chr3:154449325-154449266	8.03	5.83	4.61
116	A_30_P01021129	chr5:22890322-22939119_R		lincRNA:chr5:22890322-22939119 reverse strand	chr5:22890502-22890443	10.11	7.90	4.61
117	A_55_P2115235	NM_199195	Bckdhb	Mus musculus branched chain ketoacid dehydrogenase E1, beta polypeptide (Bckdhb), nuclear gene encoding mitochondrial protein, mRNA [NM_199195]	chr9:84017787-84017846	10.74	8.53	4.60
118	A_30_P01033109	chr5:144517700-144526071_F		lincRNA:chr5:144517700-144526071 forward strand	chr5:144525633-144525692	10.17	7.97	4.60
119	A_66_P102467	AK048565	Casz1	Mus musculus 16 days embryo head cDNA, RIKEN full-length enriched library, clone:C130075M17 product:unclassifiable, full insert sequence. [AK048565]	chr4:148213791-148213850	8.33	6.13	4.59

120	A_51_P256066	NM_011878	Tiam2	Mus musculus T-cell lymphoma invasion and metastasis 2 (Tiam2), transcript variant 1, mRNA [NM_011878]	chr17:3518885-3518944	9.17	6.97	4.59
121	A_52_P28745	NM_145399	Scgn	Mus musculus secretagogin, EF-hand calcium binding protein (Scgn), mRNA [NM_145399]	chr13:24054661-24054004	8.56	6.37	4.57
122	A_30_P01018678	chr9:121845920-121858120_F		lincRNA:chr9:121845920-121858120 forward strand	chr9:121855262-121855321	8.58	6.40	4.54
123	A_51_P368496	NM_029537	Tmem98	Mus musculus transmembrane protein 98 (Tmem98), mRNA [NM_029537]	chr11:80635413-80635472	10.92	8.74	4.52
124	A_30_P01032824	chr17:36131460-36136694_F		lincRNA:chr17:36131460-36136694 forward strand	chr17:36136152-36136211	7.71	5.54	4.52
125	A_30_P01020735	chr12:53669892-53710742_R		lincRNA:chr12:53669892-53710742 reverse strand	chr12:53669955-53669896	8.98	6.81	4.51
126	A_55_P2024953	NM_001081278	Tbc1d4	Mus musculus TBC1 domain family, member 4 (Tbc1d4), mRNA [NM_001081278]	chr14:101841636-101841577	8.96	6.79	4.50
127	A_55_P2025735	NM_007402	Adam7	Mus musculus a disintegrin and metallopeptidase domain 7 (Adam7), mRNA [NM_007402]	chr14:69115452-69115393	7.57	5.40	4.50
128	A_30_P01024431	chr18:6144775-6202232_R		lincRNA:chr18:6144775-6202232 reverse strand	chr18:6201049-6200990	8.12	5.96	4.48
129	A_55_P1985559	ENSMUST00000119783		GATA zinc finger domain containing 1 Gene [Source:MGI (curated);Acc:MGI:1914460] [ENSMUST00000119783]	chr5:3643283-3643224	11.05	8.89	4.48
130	A_30_P01020363	chr7:86680928-86700632_F		lincRNA:chr7:86680928-86700632 forward strand	chr7:86700573-86700632	8.68	6.52	4.47
131	A_52_P90124	NM_011156	Prep	Mus musculus prolyl endopeptidase (Prep), mRNA [NM_011156]	chr10:44878624-44878683	11.26	9.10	4.45
132	A_55_P2071271	NM_028440	3110003A17Rik	Mus musculus RIKEN cDNA 3110003A17 gene (3110003A17Rik), mRNA [NM_028440]	chr10:17731144-17731085	12.15	10.00	4.44
133	A_30_P01032089	chr5:26333765-26338143_F		lincRNA:chr5:26333765-26338143 forward strand	chr5:26337920-26337979	7.87	5.72	4.44
134	A_30_P01021663	chr5:77084398-77086144_R		lincRNA:chr5:77084398-77086144 reverse strand	chr5:77084972-77084913	10.31	8.16	4.43
135	A_51_P333859	NM_175448	Clvs2	Mus musculus clavesin 2 (Clvs2), mRNA [NM_175448]	chr10:33232347-33232288	7.99	5.84	4.43
136	A_55_P1960698	NM_028300	Pih1d2	Mus musculus PIH1 domain containing 2 (Pih1d2), mRNA [NM_028300]	chr9:50432998-50433057	8.12	5.98	4.42
137	A_55_P1982668	NM_001159637	Nadk	Mus musculus NAD kinase (Nadk), transcript variant 1, mRNA [NM_001159637]	chr4:154965045-154965104	8.88	6.75	4.38

138	A_52_P569348	NM_010022	Dbt	Mus musculus dihydrolipoamide branched chain transacylase E2 (Dbt), nuclear gene encoding mitochondrial protein, mRNA [NM_010022]	chr3:116252015-116252074	9.35	7.23	4.37
139	A_55_P2185573	NM_020296	Rbms1	Mus musculus RNA binding motif, single stranded interacting protein 1 (Rbms1), transcript variant 3, mRNA [NM_020296]	chr2:60590872-60590813	9.26	7.14	4.36
140	A_52_P108243	NM_011054	Pde1c	Mus musculus phosphodiesterase 1C (Pde1c), transcript variant 1, mRNA [NM_011054]	chr6:56030296-56030237	8.08	5.96	4.36
141	A_55_P2034870	NM_001024147	Gm5868	Mus musculus predicted gene 5868 (Gm5868), mRNA [NM_001024147]	chr5:72973060-72973001	11.56	9.44	4.35
142	A_51_P472829	NM_145144	Aif1l	Mus musculus allograft inflammatory factor 1-like (Aif1l), mRNA [NM_145144]	chr2:31828806-31828865	10.88	8.76	4.34
143	A_30_P01033054	chr5:35893909-35899497_F		lincRNA:chr5:35893909-35899497 forward strand	chr5:35898839-35898898	8.69	6.58	4.33
144	A_30_P01017678	chr9:121845920-121858120_R		lincRNA:chr9:121845920-121858120 reverse strand	chr9:121850954-121850895	8.29	6.18	4.32
145	A_55_P2023797	NM_029606	Ccdc46	Mus musculus coiled-coil domain containing 46 (Ccdc46), transcript variant 2, mRNA [NM_029606]	chr11:108721843-108721902	10.39	8.28	4.32
146	A_52_P925277	NM_001079883	Bcl11b	Mus musculus B-cell leukemia/lymphoma 11B (Bcl11b), transcript variant 1, mRNA [NM_001079883]	chr12:109148991-109148932	12.64	10.53	4.32
147	A_66_P134171	ENSMUST00000103384		Mus musculus mRNA for immunoglobulin G2a kappa chain, V-J region from anti-HIV p17 specific hybridoma hyHIV-12. [D14730]	chr6:70167406-70167148	9.25	7.15	4.29
148	A_30_P01019915	chr5:144517700-144526071_F		lincRNA:chr5:144517700-144526071 forward strand	chr5:144525534-144525593	8.02	5.93	4.28
149	A_55_P2386095	AK036139	9630039A02Rik	Mus musculus 16 days neonate cerebellum cDNA, RIKEN full-length enriched library, clone:9630039A02 product:chromobox homolog 3 (Drosophila HP1 gamma), pseudogene3, full insert sequence. [AK036139]	chr6:45183697-45183756	8.86	6.77	4.27
150	A_66_P115757	NM_026788	Mthfd2l	Mus musculus methylenetetrahydrofolate dehydrogenase (NADP+ dependent) 2-like (Mthfd2l), mRNA [NM_026788]	chr5:91450245-91450304	11.77	9.68	4.27

151	A_55_P2082005	NM_001165999	Lingo2	Mus musculus leucine rich repeat and Ig domain containing 2 (Lingo2), transcript variant 1, mRNA [NM_001165999]	chr4:35654714-35654655	9.03	6.94	4.26
152	A_55_P2353181	AK048022	C130030K03Rik	Mus musculus 16 days embryo head cDNA, RIKEN full-length enriched library, clone:C130030K03 product:inferred: PROTEASOME SUBUNIT ALPHA TYPE 6 (EC 3.4.99.46) (PROTEASOME IOTA CHAIN) (MACROPAIN IOTA CHAIN) (MULTI, full insert sequence. [AK048022]	chr10:48813253-48813194	8.12	6.03	4.25
153	A_66_P119376	NM_177715	Kctd12	Mus musculus potassium channel tetramerisation domain containing 12 (Kctd12), mRNA [NM_177715]	chr14:103376045-103375986	11.11	9.03	4.24
154	A_30_P01028374	chr5:22890322-22939119_R		lincRNA:chr5:22890322-22939119 reverse strand	chr5:22890382-22890323	9.95	7.88	4.23
155	A_55_P2243828	AK035826	LOC552901	Mus musculus 16 days neonate cerebellum cDNA, RIKEN full-length enriched library, clone:9630008J17 product:unclassifiable, full insert sequence. [AK035826]	chr6:141250879-141250938	10.56	8.48	4.22
156	A_30_P01021822	chr10:66559716-66647841_R		lincRNA:chr10:66559716-66647841 reverse strand	chr10:66601217-66601158	8.83	6.76	4.18
157	A_52_P593337	NM_029327	Lyrm7	Mus musculus LYR motif containing 7 (Lyrm7), mRNA [NM_029327]	chr11:54653681-54653622	7.78	5.71	4.18
158	A_55_P2039514	ENSMUST00000101391		RIKEN cDNA 9130019P16 gene Gene [Source:MGI Symbol;Acc:MGI:1918824] [ENSMUST00000101391]	chr6:54351074-54351015	7.87	5.81	4.17
159	A_55_P2142251	NM_054045	Hist2h3c2-ps	Mus musculus histone cluster 2, H3c2, pseudogene (Hist2h3c2-ps), mRNA [NM_054045]	chr3:96042093-96042034	9.52	7.46	4.17
160	A_55_P2148253	NR_029457	G530011O06Rik	Mus musculus RIKEN cDNA G530011O06 gene (G530011O06Rik), non-coding RNA [NR_029457]	chrX:166413035-166412976	8.37	6.32	4.17
161	A_55_P1954693	NM_009311	Tac1	Mus musculus tachykinin 1 (Tac1), mRNA [NM_009311]	chr6:7512893-7512952	11.68	9.62	4.16
162	A_51_P506045	NM_176073	Pgcp	Mus musculus plasma glutamate carboxypeptidase (Pgcp), transcript variant 2, mRNA [NM_176073]	chr15:33524067-33524126	7.93	5.87	4.15
163	A_55_P2057681	NM_029441	Cdyl2	Mus musculus chromodomain protein, Y chromosome-like 2 (Cdyl2), mRNA [NM_029441]	chr8:119098386-119098327	8.67	6.62	4.15

164	A_55_P1973809	NM_008220	Hbb-b1	Mus musculus hemoglobin, beta adult major chain (Hbb-b1), mRNA [NM_008220]	chr7:110961164-110961105	7.31	5.26	4.14
165	A_52_P352362	BC147147	Gm7265	Mus musculus predicted gene, EG639396, mRNA (cDNA clone MGC:182286 IMAGE:9056180), complete cds. [BC147147]	chr9:72542705-72542764	10.79	8.75	4.14
166	A_30_P01026072	chr17:36131460-36136694_F		lincRNA:chr17:36131460-36136694 forward strand	chr17:36135828-36135887	10.27	8.23	4.12
167	A_30_P01018915	chr3:126969590-126972626_R		lincRNA:chr3:126969590-126972626 reverse strand	chr3:126969713-126969654	7.80	5.76	4.12
168	A_55_P1965459	NM_145492	Zfp521	Mus musculus zinc finger protein 521 (Zfp521), mRNA [NM_145492]	chr18:13845604-13845545	8.93	6.89	4.10
169	A_51_P140429	NM_026918	Zg16	Mus musculus zymogen granule protein 16 (Zg16), mRNA [NM_026918]	chr7:134193896-134193837	9.42	7.38	4.10
170	A_52_P639229	NM_139232	Fgd4	Mus musculus FYVE, RhoGEF and PH domain containing 4 (Fgd4), transcript variant alpha, mRNA [NM_139232]	chr16:16422303-16422244	10.24	8.20	4.10
171	A_55_P2058127	NM_177145	Pde4dip	Mus musculus phosphodiesterase 4D interacting protein (myomegalin) (Pde4dip), transcript variant 3, mRNA [NM_177145]	chr3:97626079-97626020	9.06	7.03	4.09
172	A_55_P2065671	NM_172301	Ccnb1	Mus musculus cyclin B1 (Ccnb1), mRNA [NM_172301]	chr13:101548781-101548722	8.71	6.67	4.09
173	A_55_P2010906	NM_153405	Rbm45	Mus musculus RNA binding motif protein 45 (Rbm45), mRNA [NM_153405]	chr2:76221763-76221822	11.25	9.22	4.08
174	A_55_P1966908	NM_009555	Zfp40	Mus musculus zinc finger protein 40 (Zfp40), mRNA [NM_009555]	chr17:23310974-23310915	8.83	6.80	4.08
175	A_55_P2375880	BG800260	2610027H17Rik	2114-65 Mouse E14.5 retina lambda ZAP II Library Mus musculus cDNA, mRNA sequence [BG800260]	chr14:79123143-79123084	8.59	6.57	4.05
176	A_51_P214985	NM_145492	Zfp521	Mus musculus zinc finger protein 521 (Zfp521), mRNA [NM_145492]	chr18:13846783-13846724	9.27	7.25	4.05
177	A_30_P01021893	chrX:135880775-135891525_R		lincRNA:chrX:135880775-135891525 reverse strand	chrX:135882495-135882436	8.08	6.06	4.05
178	A_30_P01029305	chr5:22887983-22939658_R		lincRNA:chr5:22887983-22939658 reverse strand	chr5:22890099-22890040	10.12	8.10	4.05
179	A_52_P494622	NM_013613	Nr4a2	Mus musculus nuclear receptor subfamily 4, group A, member 2 (Nr4a2), transcript variant 1, mRNA [NM_013613]	chr2:56960758-56960699	12.22	10.21	4.05
180	A_66_P111578	NM_145624	Zfp709	Mus musculus zinc finger protein 709 (Zfp709), mRNA [NM_145624]	chr8:74416272-74416331	7.92	5.91	4.04

181	A_55_P2090330	NM_021452	Kcnmb4	Mus musculus potassium large conductance calcium-activated channel, subfamily M, beta member 4 (Kcnmb4), mRNA [NM_021452]	chr10:115855036-115854977	10.22	8.21	4.04
182	A_55_P1988613	NM_001162904	Mdm1	Mus musculus transformed mouse 3T3 cell double minute 1 (Mdm1), transcript variant 3, mRNA [NM_001162904]	chr10:117605934-117605993	8.69	6.67	4.04
183	A_55_P2179020	NM_001177658	Mrpl15	Mus musculus mitochondrial ribosomal protein L15 (Mrpl15), nuclear gene encoding mitochondrial protein, transcript variant 1, mRNA [NM_001177658]	chr1:4764592-4764533	8.65	6.63	4.04
184	A_55_P2106150	NM_021790	Cenpk	Mus musculus centromere protein K (Cenpk), transcript variant 1, mRNA [NM_021790]	chr13:105039517-105039576	7.99	5.98	4.03
185	A_30_P01029162	chr7:86680928-86700632_F		lincRNA:chr7:86680928-86700632 forward strand	chr7:86700015-86700074	9.28	7.27	4.03
186	A_30_P01020660	chr11:53592946-53637922_F		lincRNA:chr11:53592946-53637922 forward strand	chr11:53630944-53631003	9.37	7.36	4.03
187	A_52_P660422	NM_008600	Mip	Mus musculus major intrinsic protein of eye lens fiber (Mip), mRNA [NM_008600]	chr10:127668747-127668806	8.14	6.14	4.01
188	A_51_P168395	NM_030188	Ttc30a1	Mus musculus tetratricopeptide repeat domain 30A1 (Ttc30a1), mRNA [NM_030188]	chr2:75817312-75817253	8.90	6.90	4.00
189	A_30_P01019777	chr1:182808654-182815659_R		lincRNA:chr1:182808654-182815659 reverse strand	chr1:182808794-182808735	5.55	7.55	0.25
190	A_55_P2159522	NM_181277	Col14a1	Mus musculus collagen, type XIV, alpha 1 (Col14a1), mRNA [NM_181277]	chr15:55347733-55347792	6.25	8.26	0.25
191	A_55_P2160810	NM_008593	Foxd2	Mus musculus forkhead box D2 (Foxd2), mRNA [NM_008593]	chr4:114579003-114578944	5.57	7.58	0.25
192	A_55_P2091359	NM_008812	Padi2	Mus musculus peptidyl arginine deiminase, type II (Padi2), mRNA [NM_008812]	chr4:140508441-140508500	6.59	8.60	0.25
193	A_30_P01029653	chr17:39960567-39992817_F		lincRNA:chr17:39960567-39992817 forward strand	chr17:39982194-39982253	14.56	16.56	0.25
194	A_55_P2391394	AY672066		Mus musculus 10 days neonate cortex cDNA, RIKEN full-length enriched library, clone:A830095L23 product:unclassifiable, full insert sequence. [AK044157]	chr2:167018192-167018251	6.07	8.08	0.25
195	A_51_P203675	NM_021407	Trem3	Mus musculus triggering receptor expressed on myeloid cells 3 (Trem3), mRNA [NM_021407]	chr17:48397990-48398049	5.40	7.41	0.25

196	A_51_P206235	NM_028051	Slc39a5	Mus musculus solute carrier family 39 (metal ion transporter), member 5 (Slc39a5), transcript variant 1, mRNA [NM_028051]	chr10:127834762-127834340	6.40	8.41	0.25
197	A_30_P01019953	chr17:46058814-46059304_F		lincRNA:chr17:46058814-46059304 forward strand	chr17:46059101-46059160	5.62	7.63	0.25
198	A_30_P01030395	chr12:33604247-33638654_R		lincRNA:chr12:33604247-33638654 reverse strand	chr12:33604466-33604407	6.66	8.66	0.25
199	A_55_P1974967	NM_033596	Hist2h4	Mus musculus histone cluster 2, H4 (Hist2h4), mRNA [NM_033596]	chr3:96067237-96067178	9.60	11.61	0.25
200	A_30_P01033342	chr3:121997064-122031218_F		lincRNA:chr3:121997064-122031218 forward strand	chr3:121997194-121997253	7.63	9.64	0.25
201	A_51_P423578	NM_011408	Slfn2	Mus musculus schlafen 2 (Slfn2), mRNA [NM_011408]	chr11:82883791-82883850	5.74	7.75	0.25
202	A_65_P16059	NM_011578	Tgfbr3	Mus musculus transforming growth factor, beta receptor III (Tgfbr3), mRNA [NM_011578]	chr5:107535877-107535818	6.63	8.65	0.25
203	A_30_P01032592	chr2:59235155-59242518_F		lincRNA:chr2:59235155-59242518 forward strand	chr2:59237815-59237874	8.47	10.48	0.25
204	A_30_P01026416	chr7:134779841-134840591_R		lincRNA:chr7:134779841-134840591 reverse strand	chr7:134803744-134803685	6.46	8.47	0.25
205	A_55_P2068228	XR_033308	Gm8483	PREDICTED: Mus musculus similar to Eno1 protein (LOC667152), misc RNA [XR_033308]	chr14:15280209-15280150	12.30	14.32	0.25
206	A_52_P497534	NM_145940	Wipi1	Mus musculus WD repeat domain, phosphoinositide interacting 1 (Wipi1), mRNA [NM_145940]	chr11:109435199-109435140	6.82	8.84	0.25
207	A_51_P230904	NM_019877	Copz2	Mus musculus coatomer protein complex, subunit zeta 2 (Copz2), mRNA [NM_019877]	chr11:96722183-96722242	6.80	8.82	0.25
208	A_52_P359819	NM_198190	Ntf5	Mus musculus neurotrophin 5 (Ntf5), mRNA [NM_198190]	chr7:52671758-52671817	6.19	8.21	0.25
209	A_66_P131137	ENSMUST00000098303		Putative uncharacterized protein [Source:UniProtKB/TrEMBL;Acc:Q8C534] [ENSMUST00000098303]	chr7:100201036-100200977	5.82	7.84	0.25
210	A_55_P2412319	AK043919	A830052D11Rik	Mus musculus 10 days neonate cortex cDNA, RIKEN full-length enriched library, clone:A830052D11 product:unclassifiable, full insert sequence. [AK043919]	chr18:32518779-32518720	6.15	8.17	0.25
211	A_55_P2186928	XR_032647	Gm13337	PREDICTED: Mus musculus similar to 3-phosphoglycerate dehydrogenase (LOC637235), misc RNA [XR_032647]	chr2:21990326-21990385	5.99	8.01	0.25

212	A_51_P161890	NM_010189	Fcgrt	Mus musculus Fc receptor, IgG, alpha chain transporter (Fcgrt), mRNA [NM_010189]	chr7:52350615-52350556	5.82	7.85	0.25
213	A_30_P01019187	chr11:22510175-22522025_F		lincRNA:chr11:22510175-22522025 forward strand	chr11:22518673-22518732	6.72	8.75	0.25
214	A_52_P36928	NM_194269	Morn2	Mus musculus MORN repeat containing 2 (Morn2), mRNA [NM_194269]	chr17:80693897-80694892	8.76	10.80	0.24
215	A_51_P230298	NM_008232	Hdgfl1	Mus musculus hepatoma derived growth factor-like 1 (Hdgfl1), mRNA [NM_008232]	chr13:26860852-26860793	6.89	8.92	0.24
216	A_52_P640922	NM_177577	Dcdc2a	Mus musculus doublecortin domain containing 2a (Dcdc2a), mRNA [NM_177577]	chr13:25297328-25297387	5.80	7.83	0.24
217	A_55_P2001489	NM_001009940	Il19	Mus musculus interleukin 19 (Il19), mRNA [NM_001009940]	chr1:132829505-132829446	5.66	7.69	0.24
218	A_55_P2143522	NM_031380	Fstl3	Mus musculus follistatin-like 3 (Fstl3), mRNA [NM_031380]	chr10:79244916-79244975	7.21	9.25	0.24
219	A_30_P01026130	chr17:32075308-32075792_R		lincRNA:chr17:32075308-32075792 reverse strand	chr17:32075532-32075473	6.53	8.57	0.24
220	A_30_P01026820	chr14:27176059-27202735_F		lincRNA:chr14:27176059-27202735 forward strand	chr14:27195591-27195650	5.85	7.88	0.24
221	A_55_P2151728	NM_146542	Olfr11	Mus musculus olfactory receptor 11 (Olfr11), mRNA [NM_146542]	chr13:21730508-21730449	5.25	7.29	0.24
222	A_55_P1977431	NM_031161	Cck	Mus musculus cholecystokinin (Cck), mRNA [NM_031161]	chr9:121399002-121398943	6.99	9.03	0.24
223	A_55_P1969111	NM_146831	Olfr133	Mus musculus olfactory receptor 133 (Olfr133), mRNA [NM_146831]	chr17:38286408-38286467	6.11	8.15	0.24
224	A_55_P2118520	NM_007742	Col1a1	Mus musculus collagen, type I, alpha 1 (Col1a1), mRNA [NM_007742]	chr11:94813115-94813174	6.34	8.38	0.24
225	A_51_P282508	NM_007484	Rhoc	Mus musculus ras homolog gene family, member C (Rhoc), mRNA [NM_007484]	chr3:104597300-104597359	8.19	10.23	0.24
226	A_55_P2081942	XM_001478032	LOC100047273	PREDICTED: Mus musculus hypothetical protein LOC100047273 (LOC100047273), mRNA [XM_001478032]	chr4:83171555-83171496	6.37	8.41	0.24
227	A_55_P2120566	XM_001474650	LOC100045635	PREDICTED: Mus musculus similar to Ubtf protein (LOC100045635), mRNA [XM_001474650]	chr18:68117608-68117549	6.03	8.07	0.24
228	A_55_P1959368	NM_146436	Olfr998	Mus musculus olfactory receptor 998 (Olfr998), mRNA [NM_146436]	chr2:85431584-85431643	6.24	8.28	0.24
229	A_30_P01030794	chr8:72076364-72077503_R		lincRNA:chr8:72076364-72077503 reverse strand	chr8:72076861-72076802	9.76	11.80	0.24
230	A_52_P84347	XM_001473883	4931419H13Rik	PREDICTED: Mus musculus RIKEN cDNA 4931419H13 gene (4931419H13Rik), mRNA [XM_001473883]	chr3:54887687-54887836	6.34	8.39	0.24

231	A_55_P2135521	XM_001475288	Gm14703	PREDICTED: Mus musculus hypothetical LOC632264 (LOC632264), mRNA [XM_001475288]	chrX:7229093-7229034	7.59	9.63	0.24
232	A_52_P636987	ENSMUST00000105113		PREDICTED: Mus musculus gene model 362, (NCBI) (Gm362), mRNA [XM_141720]	chrX:40945674-40945733	5.78	7.82	0.24
233	A_55_P2067727	NM_026280	Mxra7	Mus musculus matrix-remodelling associated 7 (Mxra7), mRNA [NM_026280]	chr11:116673310-116673251	7.97	10.02	0.24
234	A_52_P434055	ENSMUST00000115672		baculoviral IAP repeat-containing 3 Gene [Source:MGI (curated);Acc:MGI:1197007] [ENSMUST00000115672]	chr9:7856221-7856162	5.66	7.70	0.24
235	A_55_P2057040	NM_001159662	Ppp1r16b	Mus musculus protein phosphatase 1, regulatory (inhibitor) subunit 16B (Ppp1r16b), transcript variant 1, mRNA [NM_001159662]	chr2:158588550-158588609	8.59	10.64	0.24
236	A_66_P111352	NM_013833	Rax	Mus musculus retina and anterior neural fold homeobox (Rax), mRNA [NM_013833]	chr18:66094390-66094331	7.05	9.10	0.24
237	A_30_P01017707	chr3:30077338-30080281_R		lincRNA:chr3:30077338-30080281 reverse strand	chr3:30077398-30077339	5.88	7.93	0.24
238	A_55_P2074591	NM_001110218	Ppm1h	Mus musculus protein phosphatase 1H (PP2C domain containing) (Ppm1h), transcript variant 1, mRNA [NM_001110218]	chr10:122379536-122379595	8.34	10.39	0.24
239	A_51_P389004	NM_011891	Sgcd	Mus musculus sarcoglycan, delta (dystrophin-associated glycoprotein) (Sgcd), mRNA [NM_011891]	chr11:46794404-46794345	5.99	8.04	0.24
240	A_55_P1993213	NM_146860	Olfr161	Mus musculus olfactory receptor 161 (Olfr161), mRNA [NM_146860]	chr16:3593280-3593339	6.47	8.52	0.24
241	A_30_P01029854	chr1:93744621-93825321_R		lincRNA:chr1:93744621-93825321 reverse strand	chr1:93755699-93755640	6.79	8.84	0.24
242	A_55_P2142695	NM_001122668	E330014E10Rik	Mus musculus RIKEN cDNA E330014E10 gene (E330014E10Rik), mRNA [NM_001122668]	chr5:96230913-96230972	5.40	7.45	0.24
243	A_30_P01024776	chr15:99762797-99786472_R		lincRNA:chr15:99762797-99786472 reverse strand	chr15:99764010-99763951	5.86	7.91	0.24
244	A_30_P01020733	chr8:67542714-67561694_F		lincRNA:chr8:67542714-67561694 forward strand	chr8:67552709-67552768	7.03	9.08	0.24
245	A_55_P1984941	NM_146923	Olfr20	Mus musculus olfactory receptor 20 (Olfr20), mRNA [NM_146923]	chr11:73168142-73168201	5.49	7.54	0.24
246	A_55_P2106039	NM_016693	Map3k6	Mus musculus mitogen-activated protein kinase kinase kinase 6 (Map3k6), mRNA [NM_016693]	chr4:132808672-132808731	6.43	8.49	0.24

247	A_55_P1992019	NM_001083119	Ptpru	Mus musculus protein tyrosine phosphatase, receptor type, U (Ptpru), transcript variant 2, mRNA [NM_001083119]	chr4:131324432-131324373	8.58	10.63	0.24
248	A_30_P01033311	chr1:183951156-183986156_F		lincRNA:chr1:183951156-183986156 forward strand	chr1:183959051-183959110	7.25	9.31	0.24
249	A_55_P2304010	BG083149	AU015230	H3085A07-5 NIA Mouse 15K cDNA Clone Set Mus musculus cDNA clone H3085A07 5', mRNA sequence [BG083149]	chr17:62871151-62871092	6.56	8.62	0.24
250	A_30_P01031444	chr11:31647436-31647791_R		lincRNA:chr11:31647436-31647791 reverse strand	chr11:31647668-31647609	6.37	8.43	0.24
251	A_52_P662860	ENSMUST00000069682		olfactory receptor 701 Gene [Source:MGI Symbol;Acc:MGI:3030535] [ENSMUST00000069682]	chr7:113957725-113958510	5.66	7.71	0.24
252	A_52_P265578	NM_001013607	Vmo1	Mus musculus vitelline membrane outer layer 1 homolog (chicken) (Vmo1), mRNA [NM_001013607]	chr11:70327302-70327243	5.99	8.05	0.24
253	A_52_P408315	ENSMUST00000124404		von Willebrand factor A domain containing 3B Gene [Source:MGI (curated);Acc:MGI:1918103] [ENSMUST00000124404]	chr1:37120334-37120393	5.88	7.94	0.24
254	A_51_P454873	NM_023456	Npy	Mus musculus neuropeptide Y (Npy), mRNA [NM_023456]	chr6:49773743-49777505	13.32	15.39	0.24
255	A_30_P01029858	chr4:149383134-149451259_R		lincRNA:chr4:149383134-149451259 reverse strand	chr4:149433540-149433481	8.35	10.41	0.24
256	A_30_P01024097	chr12:4133875-4243375_R		lincRNA:chr12:4133875-4243375 reverse strand	chr12:4151416-4151357	5.72	7.78	0.24
257	A_66_P123155	NM_027442	Ddo	Mus musculus D-aspartate oxidase (Ddo), mRNA [NM_027442]	chr10:40369646-40369705	5.62	7.69	0.24
258	A_52_P174721	NM_176950	Defb20	Mus musculus defensin beta 20 (Defb20), mRNA [NM_176950]	chr2:152305122-152305181	5.47	7.53	0.24
259	A_30_P01024217	chr18:5162836-5165729_R		lincRNA:chr18:5162836-5165729 reverse strand	chr18:5163016-5162957	7.54	9.60	0.24
260	A_65_P08971	NM_010171	F3	Mus musculus coagulation factor III (F3), mRNA [NM_010171]	chr3:121434609-121435346	6.61	8.68	0.24
261	A_30_P01017533	chr9:20313342-20323517_R		lincRNA:chr9:20313342-20323517 reverse strand	chr9:20323419-20323360	9.26	11.33	0.24
262	A_55_P1977446	NM_001011771	Olfr1089	Mus musculus olfactory receptor 1089 (Olfr1089), mRNA [NM_001011771]	chr2:86572895-86572836	5.66	7.72	0.24
263	A_55_P1954302	NM_001159500	Esrrb	Mus musculus estrogen related receptor, beta (Esrrb), transcript variant 2, mRNA [NM_001159500]	chr12:87862509-87862568	6.19	8.26	0.24

264	A_52_P425004	NR_033609	A930017M01Rik	Mus musculus RIKEN cDNA A930017M01 gene (A930017M01Rik), non-coding RNA [NR_033609]	chr15:44715776-44715835	6.48	8.55	0.24
265	A_55_P1955851	NM_178906	AI593442	Mus musculus expressed sequence AI593442 (AI593442), transcript variant 2, mRNA [NM_178906]	chr9:52484088-52484029	7.24	9.31	0.24
266	A_51_P236380	NM_028942	Slco6c1	Mus musculus solute carrier organic anion transporter family, member 6c1 (Slco6c1), mRNA [NM_028942]	chr1:98962680-98962621	5.57	7.65	0.24
267	A_66_P121110	NM_011044	Pck1	Mus musculus phosphoenolpyruvate carboxykinase 1, cytosolic (Pck1), mRNA [NM_011044]	chr2:172984307-172984366	6.27	8.34	0.24
268	A_55_P2082880	XM_905410	LOC631287	PREDICTED: Mus musculus similar to Interferon induced transmembrane protein 2 (LOC631287), mRNA [XM_905410]	chr7:148141920-148141861	8.70	10.78	0.24
269	A_30_P01024911	chr2:173105462-173126187_R		lincRNA:chr2:173105462-173126187 reverse strand	chr2:173114998-173114939	5.49	7.56	0.24
270	A_55_P2144155	NM_146306	Olfr518	Mus musculus olfactory receptor 518 (Olfr518), mRNA [NM_146306]	chr7:116024176-116024117	6.11	8.18	0.24
271	A_30_P01025161	chr4:8865575-8899400_F		lincRNA:chr4:8865575-8899400 forward strand	chr4:8866983-8867042	6.29	8.36	0.24
272	A_30_P01024391	chr6:125330524-125410249_R		lincRNA:chr6:125330524-125410249 reverse strand	chr6:125395628-125395569	6.07	8.15	0.24
273	A_55_P1960416	NM_146069	Lrrc33	Mus musculus leucine rich repeat containing 33 (Lrrc33), mRNA [NM_146069]	chr16:32142970-32142911	6.56	8.64	0.24
274	A_30_P01022872	chr18:75132417-75141201_R		lincRNA:chr18:75132417-75141201 reverse strand	chr18:75134377-75134318	5.55	7.63	0.24
275	A_55_P2012984	NM_001136069	Ldha	Mus musculus lactate dehydrogenase A (Ldha), transcript variant 2, mRNA [NM_001136069]	chr7:54110937-54110996	11.73	13.81	0.24
276	A_52_P1142105	ENSMUST00000103464		immunoglobulin heavy chain (X24 family) Complex/Cluster/Region [Source:MGI Symbol;Acc:MGI:96492] [ENSMUST00000103464]	chr12:115186664-115186605	5.76	7.84	0.24
277	A_55_P2053551	NM_175171	Mast4	Mus musculus microtubule associated serine/threonine kinase family member 4 (Mast4), mRNA [NM_175171]	chr13:103525147-103525088	6.82	8.90	0.24
278	A_30_P01021552	chr2:129416848-129417413_R		lincRNA:chr2:129416848-129417413 reverse strand	chr2:129417115-129417056	6.31	8.39	0.24
279	A_30_P01019581	chr7:132218864-132329172_F		lincRNA:chr7:132218864-132329172 forward strand	chr7:132326092-132326151	5.70	7.78	0.24

280	A_51_P519251	NM_019738	Nupr1	Mus musculus nuclear protein 1 (Nupr1), mRNA [NM_019738]	chr7:133767037-133766978	7.75	9.83	0.24
281	A_55_P2014144	NM_146566	Olfr830	Mus musculus olfactory receptor 830 (Olfr830), mRNA [NM_146566]	chr9:18680661-18680720	5.78	7.86	0.24
282	A_30_P01028039	chr8:122712014-122732520_F		lincRNA:chr8:122712014-122732520 forward strand	chr8:122728376-122728435	7.84	9.92	0.24
283	A_30_P01027455	chr5:137392195-137426421_R		lincRNA:chr5:137392195-137426421 reverse strand	chr5:137409832-137409773	8.17	10.25	0.24
284	A_30_P01032765	chr2:130697320-130729670_F		lincRNA:chr2:130697320-130729670 forward strand	chr2:130727793-130727852	6.73	8.81	0.24
285	A_66_P116028	NM_146828	Olfr975	Mus musculus olfactory receptor 975 (Olfr975), mRNA [NM_146828]	chr9:39757481-39757422	6.67	8.75	0.24
286	A_30_P01027500	chr8:124355710-124401160_R		lincRNA:chr8:124355710-124401160 reverse strand	chr8:124397567-124397508	5.97	8.05	0.24
287	A_52_P561650	NM_147776	Vwa1	Mus musculus von Willebrand factor A domain containing 1 (Vwa1), mRNA [NM_147776]	chr4:155143759-155143700	7.12	9.21	0.24
288	A_30_P01019652	chr1:92412246-92423471_F		lincRNA:chr1:92412246-92423471 forward strand	chr1:92419303-92419362	6.90	8.99	0.24
289	A_55_P2087544	NM_020333	Slc12a5	Mus musculus solute carrier family 12, member 5 (Slc12a5), mRNA [NM_020333]	chr2:164804887-164804946	10.22	12.31	0.24
290	A_55_P2029401	NM_001177518	Gm7849	Mus musculus predicted gene 7849 (Gm7849), mRNA [NM_001177518]	chr8:22565976-22565917	5.97	8.06	0.24
291	A_30_P01022815	chr8:41730287-41746862_F		lincRNA:chr8:41730287-41746862 forward strand	chr8:41746168-41746227	5.62	7.71	0.23
292	A_51_P438805	NM_001009935	Txnip	Mus musculus thioredoxin interacting protein (Txnip), transcript variant 1, mRNA [NM_001009935]	chr3:96365147-96365206	7.48	9.57	0.23
293	A_30_P01022773	chr15:92174226-92201201_R		lincRNA:chr15:92174226-92201201 reverse strand	chr15:92196459-92196400	6.82	8.91	0.23
294	A_55_P2146837	NM_146925	Olfr481	Mus musculus olfactory receptor 481 (Olfr481), mRNA [NM_146925]	chr7:115225189-115225248	6.95	9.05	0.23
295	A_51_P385928	NM_138750	Prom2	Mus musculus prominin 2 (Prom2), transcript variant 1, mRNA [NM_138750]	chr2:127360592-127358592	6.03	8.13	0.23
296	A_55_P2118810	XR_032614	Gm5987	PREDICTED: Mus musculus similar to L-lactate dehydrogenase A chain (LDH-A) (LDH muscle subunit) (LDH-M) (LOC546882), misc RNA [XR_032614]	chr5:106645294-106645353	7.68	9.77	0.23
297	A_55_P2128153	NM_009933	Col6a1	Mus musculus collagen, type VI, alpha 1 (Col6a1), mRNA [NM_009933]	chr10:76171597-76171538	6.22	8.32	0.23
298	A_55_P2110579	XM_001474778	Gm4507	PREDICTED: Mus musculus hypothetical protein LOC100045703 (LOC100045703), mRNA [XM_001474778]	chr14:118610538-118610597	5.86	7.96	0.23

299	A_30_P01031245	chr14:33895294-33923644_R		lincRNA:chr14:33895294-33923644 reverse strand	chr14:33912478-33912419	8.15	10.24	0.23
300	A_30_P01029666	chr13:98062189-98066613_F		lincRNA:chr13:98062189-98066613 forward strand	chr13:98066473-98066532	6.62	8.72	0.23
301	A_55_P2137073	ENSMUST00000105170			chr1:85109946-85109887	5.53	7.63	0.23
302	A_55_P2063471	XR_001926	Gm11814	PREDICTED: Mus musculus similar to Ldha protein (LOC666840), misc RNA [XR_001926]	chr4:10365541-10365482	11.98	14.08	0.23
303	A_51_P172085	NM_008113	Arhgdig	Mus musculus Rho GDP dissociation inhibitor (GDI) gamma (Arhgdig), mRNA [NM_008113]	chr17:26336287-26336228	6.90	9.01	0.23
304	A_66_P136582	ENSMUST00000082150		high mobility group box 1-like Gene [Source:MGI Symbol;Acc:MGI:3054046] [ENSMUST00000082150]	chr6:131558370-131558311	9.64	11.75	0.23
305	A_55_P2207020	BY715827	4930564G21Rik	BY715827 RIKEN full-length enriched, adult male testis Mus musculus cDNA clone 4930564G21 5'. [BY715827]		5.53	7.63	0.23
306	A_55_P2172779	XM_001475665	Gm3105	PREDICTED: Mus musculus hypothetical protein LOC100041029 (LOC100041029), mRNA [XM_001475665]	chr7:66861366-66861307	11.70	13.80	0.23
307	A_55_P2165869	NM_009883	Cebpb	Mus musculus CCAAT/enhancer binding protein (C/EBP), beta (Cebpb), mRNA [NM_009883]	chr2:167515828-167515887	5.57	7.68	0.23
308	A_65_P17218	NM_001170853	Mndal	Mus musculus myeloid nuclear differentiation antigen like (Mndal), mRNA [NM_001170853]	chr1:175787609-175787550	5.55	7.66	0.23
309	A_30_P01028523	chr16:30195156-30200385_F		lincRNA:chr16:30195156-30200385 forward strand	chr16:30200266-30200325	5.86	7.96	0.23
310	A_55_P2020054	AK164572	2610035D17Rik	Mus musculus 13 days embryo lung cDNA, RIKEN full-length enriched library, clone:D430003N19 product:hypothetical protein, full insert sequence. [AK164572]	chr11:113061839-113061780	8.65	10.76	0.23
311	A_55_P2062886	XM_001475641	Gm3094	PREDICTED: Mus musculus hypothetical protein LOC100041010 (LOC100041010), mRNA [XM_001475641]	chr7:66814623-66814564	10.66	12.77	0.23
312	A_51_P109840	NM_011707	Vtn	Mus musculus vitronectin (Vtn), mRNA [NM_011707]	chr11:78315393-78315665	5.86	7.97	0.23
313	A_55_P2153545	XR_031824	Gm7108	PREDICTED: Mus musculus similar to mKIAA3013 protein (LOC633246), misc RNA [XR_031824]	chrX:87047714-87047655	5.30	7.41	0.23
314	A_30_P01020657	chr16:9129538-9137898_R		lincRNA:chr16:9129538-9137898 reverse strand	chr16:9137613-9137554	5.57	7.69	0.23
315	A_51_P143031	NM_133867	Eps8l3	Mus musculus EPS8-like 3 (Eps8l3), mRNA [NM_133867]	chr3:107695731-107695790	9.40	11.51	0.23

316	A_30_P01022367	chr11:60728207-60745369_R		lincRNA:chr11:60728207-60745369 reverse strand	chr11:60731874-60731815	6.25	8.36	0.23
317	A_55_P2019799	NM_146543	Olfr1360	Mus musculus olfactory receptor 1360 (Olfr1360), mRNA [NM_146543]	chr13:21765929-21765870	6.74	8.85	0.23
318	A_55_P2088033	NM_001164329	Gm6904	Mus musculus predicted gene 6904 (Gm6904), mRNA [NM_001164329]	chr14:59877396-59877337	5.40	7.52	0.23
319	A_30_P01024971	chr6:118250184-118258737_R		lincRNA:chr6:118250184-118258737 reverse strand	chr6:118250244-118250185	6.29	8.40	0.23
320	A_51_P457557	NM_153393	Col23a1	Mus musculus collagen, type XXIII, alpha 1 (Col23a1), mRNA [NM_153393]	chr11:51381425-51382971	6.07	8.19	0.23
321	A_55_P2096762	NM_008113	Arhgdig	Mus musculus Rho GDP dissociation inhibitor (GDI) gamma (Arhgdig), mRNA [NM_008113]	chr17:26336417-26336358	6.87	8.99	0.23
322	A_52_P175028	ENSMUST00000030257		cache domain containing 1 Gene [Source:MGI (curated);Acc:MGI:2444177] [ENSMUST00000030257]	chr4:100676879-100676938	7.94	10.06	0.23
323	A_51_P102257	ENSMUST00000050681		tensin 1 Gene [Source:MGI Symbol;Acc:MGI:104552] [ENSMUST00000050681]	chr1:74036446-74036387	5.47	7.59	0.23
324	A_55_P2180849	XM_001475514	AA684185	PREDICTED: Mus musculus similar to elongation factor 1 homolog (S. cerevisiae) (LOC100040632), mRNA [XM_001475514]	chr18:80210484-80210543	6.78	8.91	0.23
325	A_55_P2025123	ENSMUST00000108317	Proca1	protein interacting with cyclin A1 Gene [Source:MGI (curated);Acc:MGI:1918274] [ENSMUST00000108317]	chr11:78008396-78008455	6.46	8.59	0.23
326	A_30_P01023281	chr1:84947500-85045150_F		lincRNA:chr1:84947500-85045150 forward strand	chr1:85036029-85036088	5.86	7.99	0.23
327	A_55_P2052655	NM_177753	Sox21	Mus musculus SRY-box containing gene 21 (Sox21), mRNA [NM_177753]	chr14:118633410-118633351	7.69	9.83	0.23
328	A_55_P2241299	NM_001025613	Otud7b	Mus musculus OTU domain containing 7B (Otud7b), transcript variant 1, mRNA [NM_001025613]	chr3:95962836-95962895	7.23	9.37	0.23
329	A_30_P01030456	chr6:131263850-131314850_F		lincRNA:chr6:131263850-131314850 forward strand	chr6:131272426-131272485	7.52	9.65	0.23
330	A_55_P2002527	XM_979344	Gsdmcl2	PREDICTED: Mus musculus similar to Gasdermin domain containing protein RGD1359449 (LOC665769), mRNA [XM_979344]	chr15:63720569-63720628	5.99	8.12	0.23
331	A_55_P1991451	XM_001478201	Gm3845	PREDICTED: Mus musculus similar to cyclic nucleotide gated channel beta 1 (LOC100042436), mRNA [XM_001478201]	chr16:5718323-5718264	6.11	8.25	0.23

332	A_30_P01031825	chr8:122712014-122732520_F		lincRNA:chr8:122712014-122732520 forward strand	chr8:122727472-122727531	6.40	8.54	0.23
333	A_51_P491667	NM_024440	Derl3	Mus musculus Der1-like domain family, member 3 (Derl3), mRNA [NM_024440]	chr10:75358503-75358562	5.95	8.09	0.23
334	A_30_P01020124	chr15:96984422-97000322_R		lincRNA:chr15:96984422-97000322 reverse strand	chr15:96990690-96990631	6.80	8.94	0.23
335	A_52_P381484	NM_133903	Spon2	Mus musculus spondin 2, extracellular matrix protein (Spon2), mRNA [NM_133903]	chr5:33556676-33556617	5.62	7.76	0.23
336	A_30_P01031724	chr7:48794017-48815817_F		lincRNA:chr7:48794017-48815817 forward strand	chr7:48802100-48802159	5.49	7.63	0.23
337	A_52_P120132	ENSMUST00000066875		RIKEN cDNA 6030452D12 gene Gene [Source:MGI Symbol;Acc:MGI:3045356] [ENSMUST00000066875]	chr8:109032939-109032998	6.19	8.33	0.23
338	A_52_P193611	NM_181422	Pkd2l1	Mus musculus polycystic kidney disease 2-like 1 (Pkd2l1), mRNA [NM_181422]	chr19:44222665-44222606	6.94	9.08	0.23
339	A_66_P127070	NM_008109	Gdf5	Mus musculus growth differentiation factor 5 (Gdf5), mRNA [NM_008109]	chr2:155766860-155766801	8.25	10.39	0.23
340	A_30_P01025687	chr15:61984389-62102500_F		lincRNA:chr15:61984389-62102500 forward strand	chr15:62074025-62074084	6.56	8.71	0.23
341	A_52_P188678	NM_001159724	Pvrl2	Mus musculus poliovirus receptor-related 2 (Pvrl2), transcript variant 2, mRNA [NM_001159724]	chr7:20309924-20309866	9.10	11.25	0.23
342	A_55_P2131168	NM_029210	Sv2c	Mus musculus synaptic vesicle glycoprotein 2c (Sv2c), mRNA [NM_029210]	chr13:96746045-96745986	7.06	9.21	0.23
343	A_55_P2002998	NM_145921	Olah	Mus musculus oleoyl-ACP hydrolase (Olah), mRNA [NM_145921]	chr2:3259348-3259289	7.12	9.27	0.23
344	A_30_P01031520	chr10:60473030-60535340_F		lincRNA:chr10:60473030-60535340 forward strand	chr10:60528810-60528869	5.86	8.01	0.23
345	A_30_P01021626	chr7:91558475-91733625_R		lincRNA:chr7:91558475-91733625 reverse strand	chr7:91725532-91725473	6.11	8.26	0.23
346	A_52_P521564	NM_001163103	Gm70	Mus musculus predicted gene 70 (Gm70), mRNA [NM_001163103]	chr12:77539089-77539378	5.90	8.06	0.22
347	A_55_P1967885	NM_008173	Nr3c1	Mus musculus nuclear receptor subfamily 3, group C, member 1 (Nr3c1), mRNA [NM_008173]	chr18:39571700-39571641	7.11	9.27	0.22
348	A_30_P01020965	chr16:94055900-94120575_R		lincRNA:chr16:94055900-94120575 reverse strand	chr16:94085980-94085921	9.56	11.72	0.22
349	A_55_P2048270	XM_001478335	LOC100047523	PREDICTED: Mus musculus hypothetical protein LOC100047523 (LOC100047523), mRNA [XM_001478335]	chr11:88964796-88964855	6.67	8.82	0.22

350	A_30_P01033316	chr8:122712014-122732520_R		lincRNA:chr8:122712014-122732520 reverse strand	chr8:122724510-122724451	7.97	10.13	0.22
351	A_55_P2086143	NM_018887	Cyp39a1	Mus musculus cytochrome P450, family 39, subfamily a, polypeptide 1 (Cyp39a1), mRNA [NM_018887]	chr17:43887735-43887794	7.68	9.84	0.22
352	A_51_P315904	NM_011817	Gadd45g	Mus musculus growth arrest and DNA-damage-inducible 45 gamma (Gadd45g), mRNA [NM_011817]	chr13:51943733-51943792	9.36	11.52	0.22
353	A_30_P01025010	chr6:127111250-127142925_F		lincRNA:chr6:127111250-127142925 forward strand	chr6:127133442-127133501	11.80	13.97	0.22
354	A_30_P01025548	chr2:157361470-157379813_R		lincRNA:chr2:157361470-157379813 reverse strand	chr2:157371660-157371601	8.92	11.08	0.22
355	A_30_P01018366	chr4:33367670-33368135_R		lincRNA:chr4:33367670-33368135 reverse strand	chr4:33367851-33367792	6.40	8.57	0.22
356	A_55_P2180916	XM_001476487	LOC100046602	PREDICTED: Mus musculus similar to immunoglobulin gamma-2a chain (LOC100046602), mRNA [XM_001476487]		5.16	7.33	0.22
357	A_66_P128342	XM_001472313	Gm8635	PREDICTED: Mus musculus similar to TRAV3-1 (LOC546630), mRNA [XM_001472313]	chr14:53200353-53200412	6.41	8.59	0.22
358	A_52_P609334	NM_030075	Klhdc8b	Mus musculus kelch domain containing 8B (Klhdc8b), mRNA [NM_030075]	chr9:108350662-108350603	9.46	11.63	0.22
359	A_30_P01024423	chr1:63264155-63345773_R		lincRNA:chr1:63264155-63345773 reverse strand	chr1:63341602-63341543	10.06	12.23	0.22
360	A_30_P01029985	chr18:75131739-75144870_R		lincRNA:chr18:75131739-75144870 reverse strand	chr18:75140545-75140486	6.95	9.12	0.22
361	A_52_P211949	NM_011286	Rph3a	Mus musculus rabphilin 3A (Rph3a), mRNA [NM_011286]	chr5:121391071-121391012	9.29	11.46	0.22
362	A_55_P2006410	XM_001479363	LOC100048112	PREDICTED: Mus musculus hypothetical protein LOC100048112 (LOC100048112), mRNA [XM_001479363]	chr7:87391613-87391554	6.52	8.70	0.22
363	A_55_P2001324	NM_146855	Olfr985	Mus musculus olfactory receptor 985 (Olfr985), mRNA [NM_146855]	chr9:39934668-39934609	6.11	8.29	0.22
364	A_55_P2254769	AK137666	4931402G19Rik	Mus musculus adult female vagina cDNA, RIKEN full-length enriched library, clone:9930017J12 product:unclassifiable, full insert sequence. [AK137666]	chr2:120298106-120298165	7.46	9.64	0.22
365	A_55_P2029581	XM_001476111	Gm3256	PREDICTED: Mus musculus hypothetical protein LOC100041292 (LOC100041292), mRNA [XM_001476111]	chr6:54612304-54612363	5.90	8.09	0.22
366	A_55_P2059323	NR_028497	Gm13315	Mus musculus predicted gene 13315 (Gm13315), non-coding RNA [NR_028497]	chr2:14643230-14643289	7.53	9.72	0.22

367	A_55_P2000613	NM_178703	Slc6a1	Mus musculus solute carrier family 6 (neurotransmitter transporter, GABA), member 1 (Slc6a1), mRNA [NM_178703]	chr6:114254853-114254912	11.92	14.11	0.22
368	A_30_P01023395	chr4:115577276-115577566_F		lincRNA:chr4:115577276-115577566 forward strand	chr4:115577507-115577566	6.15	8.34	0.22
369	A_51_P374726	NM_008987	Ptx3	Mus musculus pentraxin related gene (Ptx3), mRNA [NM_008987]	chr3:66029245-66029304	6.37	8.57	0.22
370	A_30_P01024155	chr14:26835914-26845344_F		lincRNA:chr14:26835914-26845344 forward strand	chr14:26842178-26842237	6.31	8.51	0.22
371	A_55_P2187899	AK015708	4930505N22Rik	Mus musculus adult male testis cDNA, RIKEN full-length enriched library, clone:4930505N22 product:hypothetical protein, full insert sequence. [AK015708]	chr19:46087803-46087862	7.34	9.53	0.22
372	A_55_P2125376	NM_001083616	Cacna1d	Mus musculus calcium channel, voltage-dependent, L type, alpha 1D subunit (Cacna1d), transcript variant 2, mRNA [NM_001083616]	chr14:30855318-30855259	7.24	9.43	0.22
373	A_30_P01019785	chr8:72077827-72109034_R		lincRNA:chr8:72077827-72109034 reverse strand	chr8:72077915-72077856	8.45	10.65	0.22
374	A_55_P1986701	XM_895013	1700055N04Rik	PREDICTED: Mus musculus RIKEN cDNA 1700055N04 gene, transcript variant 1 (1700055N04Rik), mRNA [XM_895013]	chr19:3966478-3966537	5.30	7.50	0.22
375	A_52_P334562	NM_009504	Vdr	Mus musculus vitamin D receptor (Vdr), mRNA [NM_009504]	chr15:97685195-97685136	6.11	8.30	0.22
376	A_30_P01026942	chr9:30805020-30810645_R		lincRNA:chr9:30805020-30810645 reverse strand	chr9:30805314-30805255	5.78	7.97	0.22
377	A_55_P1974073	ENSMUST00000136202		fibrous sheath-interacting protein 2 Gene [Source:MGI (curated);Acc:MGI:2664111] [ENSMUST00000136202]	chr2:82805590-82805647	5.55	7.75	0.22
378	A_55_P2123616	NM_001033541	Gm5127	Mus musculus predicted gene 5127 (Gm5127), mRNA [NM_001033541]	chrX:103905812-103905871	6.37	8.57	0.22
379	A_55_P2073789	XM_001003154	LOC676974	PREDICTED: Mus musculus similar to Glucose phosphate isomerase 1, transcript variant 2 (LOC676974), mRNA [XM_001003154]	chr7:35000280-35000221	9.23	11.44	0.22
380	A_51_P157042	NM_010217	Ctgf	Mus musculus connective tissue growth factor (Ctgf), mRNA [NM_010217]	chr10:24318281-24318340	6.78	8.98	0.22
381	A_51_P160536	NM_029335	1700026D08Rik	Mus musculus RIKEN cDNA 1700026D08 gene (1700026D08Rik), mRNA [NM_029335]	chr7:90924380-90924321	5.74	7.94	0.22

382	A_30_P01022110	chr5:31862823-31871445_F		lincRNA:chr5:31862823-31871445 forward strand	chr5:31871306-31871365	6.69	8.89	0.22
383	A_55_P2136606	NM_001081200	Crnn	Mus musculus cornulin (Crnn), mRNA [NM_001081200]	chr3:92953334-92953393	5.90	8.11	0.22
384	A_55_P2121735	XM_975980	Gm7578	PREDICTED: Mus musculus predicted gene, EG665304 (EG665304), mRNA [XM_975980]	chr7:74573986-74573927	6.46	8.66	0.22
385	A_55_P2115318	NM_174993	Fmr1nb	Mus musculus fragile X mental retardation 1 neighbor (Fmr1nb), transcript variant 1, mRNA [NM_174993]	chrX:66056806-66056865	6.53	8.74	0.22
386	A_51_P386983	NM_009406	Tnni3	Mus musculus troponin I, cardiac 3 (Tnni3), mRNA [NM_009406]	chr7:4472102-4472043	6.46	8.67	0.22
387	A_30_P01018971	chr2:65240793-65292293_F		lincRNA:chr2:65240793-65292293 forward strand	chr2:65252990-65253049	6.61	8.82	0.22
388	A_51_P371867	NM_027512	3830417A13Rik	Mus musculus RIKEN cDNA 3830417A13 gene (3830417A13Rik), mRNA [NM_027512]	chrX:61431614-61431673	5.40	7.62	0.21
389	A_51_P253984	NM_008791	Pcp4	Mus musculus Purkinje cell protein 4 (Pcp4), mRNA [NM_008791]	chr16:96747241-96747300	8.39	10.61	0.21
390	A_30_P01031453	chr11:97275905-97310538_F		lincRNA:chr11:97275905-97310538 forward strand	chr11:97309780-97309839	7.33	9.55	0.21
391	A_55_P1985337	NM_031395	Sytl3	Mus musculus synaptotagmin-like 3 (Sytl3), transcript variant 1, mRNA [NM_031395]	chr17:6942330-6942389	5.90	8.13	0.21
392	A_51_P450278	NM_027237	2010003K11Rik	Mus musculus RIKEN cDNA 2010003K11 gene (2010003K11Rik), mRNA [NM_027237]	chr19:4496987-4496928	5.90	8.13	0.21
393	A_30_P01021156	chr2:157361470-157379813_F		lincRNA:chr2:157361470-157379813 forward strand	chr2:157371826-157371885	7.00	9.23	0.21
394	A_52_P254088	NM_177396	Il28b	Mus musculus interleukin 28B (Il28b), mRNA [NM_177396]	chr7:29308016-29308545	6.33	8.56	0.21
395	A_30_P01023479	chr8:122728333-122752130_R		lincRNA:chr8:122728333-122752130 reverse strand	chr8:122728393-122728334	7.17	9.40	0.21
396	A_55_P2021089	XM_001472541	Ighg	PREDICTED: Mus musculus similar to immunoglobulin gamma 2A chain (LOC100048855), mRNA [XM_001472541]		6.37	8.61	0.21
397	A_30_P01033185	chr4:88604248-88645948_R		lincRNA:chr4:88604248-88645948 reverse strand	chr4:88641776-88641717	6.61	8.85	0.21
398	A_30_P01025571	chr1:51753968-51803787_R		lincRNA:chr1:51753968-51803787 reverse strand	chr1:51754108-51754049	5.95	8.19	0.21
399	A_55_P2064506	NM_001037247	Defb36	Mus musculus defensin beta 36 (Defb36), mRNA [NM_001037247]	chr2:152438270-152438329	5.90	8.15	0.21

400	A_55_P2089223	NM_001177602	Ak4	Mus musculus adenylate kinase 4 (Ak4), nuclear gene encoding mitochondrial protein, transcript variant 1, mRNA [NM_001177602]	chr4:101139464-101139523	7.03	9.27	0.21
401	A_55_P2030433	NM_008155	Gpi1	Mus musculus glucose phosphate isomerase 1 (Gpi1), mRNA [NM_008155]	chr7:34987233-34987174	10.86	13.10	0.21
402	A_30_P01023944	chr8:122712014-122732520_R		lincRNA:chr8:122712014-122732520 reverse strand	chr8:122725354-122725295	8.17	10.42	0.21
403	A_52_P112110	NM_145987	Tmem82	Mus musculus transmembrane protein 82 (Tmem82), mRNA [NM_145987]	chr4:141170338-141170279	6.37	8.62	0.21
404	A_55_P1960894	NM_013879	Cabp1	Mus musculus calcium binding protein 1 (Cabp1), mRNA [NM_013879]	chr5:115622828-115622769	7.07	9.33	0.21
405	A_55_P1987379	NM_146328	Olfr110	Mus musculus olfactory receptor 110 (Olfr110), mRNA [NM_146328]	chr17:37636492-37636551	6.03	8.28	0.21
406	A_52_P355084	NM_144797	Metrnl	Mus musculus meteorin, glial cell differentiation regulator-like (Metrnl), mRNA [NM_144797]	chr11:121577358-121577417	6.34	8.60	0.21
407	A_55_P2154148	NR_033642	Gm3230	Mus musculus predicted gene 3230 (Gm3230), non-coding RNA [NR_033642]	chr2:19577492-19577433	5.90	8.16	0.21
408	A_55_P2022434	NM_008155	Gpi1	Mus musculus glucose phosphate isomerase 1 (Gpi1), mRNA [NM_008155]	chr7:34987237-34987178	11.82	14.07	0.21
409	A_55_P1955871	NM_175290	Nlrp4f	Mus musculus NLR family, pyrin domain containing 4F (Nlrp4f), mRNA [NM_175290]	chr13:65278503-65278444	5.85	8.10	0.21
410	A_66_P111871	XM_001477068	Gm3336	PREDICTED: Mus musculus similar to mucin 2 precursor (LOC100046923), mRNA [XM_001477068]	chr8:73246088-73246147	7.07	9.33	0.21
411	A_30_P01018545	chr17:46972160-46997919_F		lincRNA:chr17:46972160-46997919 forward strand	chr17:46994432-46994491	7.80	10.06	0.21
412	A_30_P01027485	chr13:81773237-81783062_R		lincRNA:chr13:81773237-81783062 reverse strand	chr13:81775391-81775332	5.57	7.83	0.21
413	A_30_P01026088	chr2:33500594-33501874_F		lincRNA:chr2:33500594-33501874 forward strand	chr2:33501484-33501543	5.74	8.00	0.21
414	A_55_P1964257	ENSMUST00000066625			chr2:79250892-79250951	5.85	8.11	0.21
415	A_30_P01022162	chr6:117829576-117834247_F		lincRNA:chr6:117829576-117834247 forward strand	chr6:117829691-117829750	6.11	8.37	0.21
416	A_30_P01018192	chr15:41486232-41499767_R		lincRNA:chr15:41486232-41499767 reverse strand	chr15:41486439-41486380	7.37	9.63	0.21
417	A_30_P01019146	chr2:128211990-128215223_R		lincRNA:chr2:128211990-128215223 reverse strand	chr2:128212284-128212225	6.67	8.93	0.21
418	A_30_P01024335	chr16:59557120-59602070_F		lincRNA:chr16:59557120-59602070 forward strand	chr16:59557318-59557377	5.78	8.04	0.21

419	A_55_P2144115	NM_028589	1700125H20Rik	Mus musculus RIKEN cDNA 1700125H20 gene (1700125H20Rik), mRNA [NM_028589]	chr11:84994547-84994606	6.22	8.48	0.21
420	A_51_P135732	ENSMUST00000031781		coiled-coil domain containing 136 Gene [Source:MGI (curated);Acc:MGI:1918128] [ENSMUST00000031781]	chr6:29368136-29376666	8.11	10.38	0.21
421	A_55_P2060672	NM_010452	Hoxa3	Mus musculus homeobox A3 (Hoxa3), mRNA [NM_010452]	chr6:52119146-52119087	5.97	8.23	0.21
422	A_55_P2005646	NM_030207	Sfi1	Mus musculus Sfi1 homolog, spindle assembly associated (yeast) (Sfi1), mRNA [NM_030207]	chr11:3031912-3031853	11.53	13.80	0.21
423	A_51_P194503	NM_146717	Olfr433	Mus musculus olfactory receptor 433 (Olfr433), mRNA [NM_146717]	chr1:175972960-175973019	6.72	8.99	0.21
424	A_55_P1988013	XM_001476090	Gm3249	PREDICTED: Mus musculus similar to Ubtf protein (LOC100041280), mRNA [XM_001476090]	chr4:53999887-53999946	5.40	7.68	0.21
425	A_51_P460643	NM_001079869	Hoxb3	Mus musculus homeobox B3 (Hoxb3), transcript variant 1, mRNA [NM_001079869]	chr11:96207114-96207173	6.09	8.36	0.21
426	A_55_P2099620	NM_145998	Hmx2	Mus musculus H6 homeobox 2 (Hmx2), mRNA [NM_145998]	chr7:138700012-138700071	6.99	9.27	0.21
427	A_52_P137765	NM_019390	Lmna	Mus musculus lamin A (Lmna), transcript variant 2, mRNA [NM_019390]	chr3:88287321-88287262	7.09	9.37	0.21
428	A_66_P109733	NM_001165919	1700123I01Rik	Mus musculus RIKEN cDNA 1700123I01 gene (1700123I01Rik), mRNA [NM_001165919]	chr19:6223859-6223918	6.29	8.57	0.21
429	A_66_P114042	NM_172509	Kctd7	Mus musculus potassium channel tetramerisation domain containing 7 (Kctd7), mRNA [NM_172509]	chr5:130631569-130631628	7.73	10.01	0.21
430	A_30_P01029360	chr3:9401052-9415202_F		lincRNA:chr3:9401052-9415202 forward strand	chr3:9409096-9409155	6.79	9.07	0.21
431	A_52_P312467	NM_001079869	Hoxb3	Mus musculus homeobox B3 (Hoxb3), transcript variant 1, mRNA [NM_001079869]	chr11:96209002-96209061	5.90	8.18	0.21
432	A_51_P480136	NM_021541	Cryba2	Mus musculus crystallin, beta A2 (Cryba2), mRNA [NM_021541]	chr1:74937227-74937168	5.76	8.04	0.21
433	A_55_P2067505	NM_030696	Slc16a3	Mus musculus solute carrier family 16 (monocarboxylic acid transporters), member 3 (Slc16a3), mRNA [NM_030696]	chr11:120820030-120820089	7.65	9.94	0.20
434	A_52_P429450	NM_008694	Ngp	Mus musculus neutrophilic granule protein (Ngp), mRNA [NM_008694]	chr9:110324266-110324814	5.90	8.20	0.20

435	A_52_P593212	ENSMUST00000114435		RIKEN cDNA 5730446D14 gene Gene [Source:MGI Symbol;Acc:MGI:1913890] [ENSMUST00000114435]	chr6:52119107-52119166	6.03	8.33	0.20
436	A_30_P01017450	chr1:94077221-94091396_F		lincRNA:chr1:94077221-94091396 forward strand	chr1:94091065-94091124	5.82	8.12	0.20
437	A_30_P01031530	chr9:96664617-96683617_R		lincRNA:chr9:96664617-96683617 reverse strand	chr9:96676202-96676143	6.67	8.96	0.20
438	A_30_P01030847	chr1:94655789-94657057_R		lincRNA:chr1:94655789-94657057 reverse strand	chr1:94655909-94655850	6.90	9.19	0.20
439	A_30_P01024857	chr18:5951953-5952386_F		lincRNA:chr18:5951953-5952386 forward strand	chr18:5951997-5952056	7.59	9.89	0.20
440	A_55_P2109326	NM_009599	Ache	Mus musculus acetylcholinesterase (Ache), mRNA [NM_009599]	chr5:137733122-137733181	8.58	10.88	0.20
441	A_55_P2156370	NM_001007591	BB014433	Mus musculus expressed sequence BB014433 (BB014433), mRNA [NM_001007591]	chr8:15041741-15041682	6.95	9.26	0.20
442	A_55_P2017694	NM_001110337	Gprc5c	Mus musculus G protein-coupled receptor, family C, group 5, member C (Gprc5c), transcript variant 1, mRNA [NM_001110337]	chr11:114733852-114733911	5.66	7.96	0.20
443	A_51_P480861	NM_021453	Pga5	Mus musculus pepsinogen 5, group I (Pga5), mRNA [NM_021453]	chr19:10744036-10743903	7.10	9.41	0.20
444	A_55_P1982857	ENSMUST00000062214		RIKEN cDNA 1500002C15 gene Gene [Source:MGI Symbol;Acc:MGI:1916196] [ENSMUST00000062214]	chr4:155108559-155108500	7.12	9.42	0.20
445	A_52_P540350	ENSMUST00000118300		Novel protein similar to testis derived transcript (Tes)MCG8129 ; [Source:UniProtKB/TrEMBL;Acc:A2ALC6] [ENSMUST00000118300]	chrX:23535114-23535055	5.99	8.29	0.20
446	A_55_P2174541	NM_001013371	Dtx3l	Mus musculus deltex 3-like (Drosophila) (Dtx3l), mRNA [NM_001013371]	chr16:35932944-35932885	6.09	8.40	0.20
447	A_52_P1037027	ENSMUST00000098718		Putative uncharacterized protein [Source:UniProtKB/TrEMBL;Acc:Q3V438] [ENSMUST00000098718]	chr8:67552607-67552548	7.09	9.40	0.20
448	A_55_P2168781	XR_031340	Gm7997	PREDICTED: Mus musculus similar to lactate dehydrogenase 1, A chain (LOC666237), misc RNA [XR_031340]	chr8:83295453-83295512	8.23	10.54	0.20
449	A_55_P1975667	NM_001039698	Rhox4g	Mus musculus reproductive homeobox 4G (Rhox4g), mRNA [NM_001039698]	chrX:35061231-35061172	6.36	8.67	0.20
450	A_52_P235880	NM_198635	Gm5134	Mus musculus predicted gene 5134 (Gm5134), mRNA [NM_198635]	chr10:75471327-75471386	6.37	8.68	0.20
451	A_30_P01022300	chr2:59235155-59242518_F		lincRNA:chr2:59235155-59242518 forward strand	chr2:59237280-59237339	10.74	13.06	0.20

452	A_51_P113178	NM_175398	6530418L21Rik	Mus musculus RIKEN cDNA 6530418L21 gene (6530418L21Rik), transcript variant 1, mRNA [NM_175398]	chr3:105520821-105520880	7.26	9.58	0.20
453	A_30_P01032728	chr2:10237649-10287591_R		lincRNA:chr2:10237649-10287591 reverse strand	chr2:10270283-10270224	6.13	8.45	0.20
454	A_30_P01032763	chr2:33500594-33501874_F		lincRNA:chr2:33500594-33501874 forward strand	chr2:33501619-33501678	6.22	8.54	0.20
455	A_55_P2078660	NM_178250	Pramel7	Mus musculus preferentially expressed antigen in melanoma like 7 (Pramel7), mRNA [NM_178250]	chr2:87329326-87329267	6.15	8.47	0.20
456	A_51_P329928	NM_013750	Phlda3	Mus musculus pleckstrin homology-like domain, family A, member 3 (Phlda3), mRNA [NM_013750]	chr1:137665642-137665701	7.10	9.43	0.20
457	A_51_P405606	NM_008681	Ndrg1	Mus musculus N-myc downstream regulated gene 1 (Ndrg1), mRNA [NM_008681]	chr15:66761116-66761057	5.66	7.98	0.20
458	A_55_P1986868	XR_032569	Gm3267	PREDICTED: Mus musculus similar to ribosomal protein (LOC100041313), misc RNA [XR_032569]	chr15:37423179-37423238	5.93	8.25	0.20
459	A_51_P305437	NM_009037	Rcn1	Mus musculus reticulocalbin 1 (Rcn1), mRNA [NM_009037]	chr2:105227267-105227208	8.35	10.68	0.20
460	A_52_P91274	XM_988035	1700018G05Rik	PREDICTED: Mus musculus RIKEN cDNA 1700018G05 gene (1700018G05Rik), mRNA [XM_988035]	chrX:100124337-100124396	5.86	8.19	0.20
461	A_52_P15300	NM_134032	Hoxb2	Mus musculus homeobox B2 (Hoxb2), mRNA [NM_134032]	chr11:96215122-96215181	6.29	8.62	0.20
462	A_51_P324529	NM_029755	Calcoco2	Mus musculus calcium binding and coiled-coil domain 2 (Calcoco2), transcript variant 1, mRNA [NM_029755]	chr11:95961294-95961235	6.16	8.50	0.20
463	A_55_P1992208	NM_008155	Gpi1	Mus musculus glucose phosphate isomerase 1 (Gpi1), mRNA [NM_008155]	chr7:34987232-34987173	10.86	13.20	0.20
464	A_55_P2231244	NM_008498	Lhx1	Mus musculus LIM homeobox protein 1 (Lhx1), mRNA [NM_008498]	chr11:84335594-84335442	9.36	11.70	0.20
465	A_30_P01024458	chr9:41422319-41425855_F		lincRNA:chr9:41422319-41425855 forward strand	chr9:41425599-41425658	5.95	8.29	0.20
466	A_55_P2099565	ENSMUST00000084758		olfactory receptor 471 Gene [Source:MGI Symbol;Acc:MGI:3030305] [ENSMUST00000084758]	chr7:115004629-115004570	6.15	8.50	0.20
467	A_30_P01024530	chr11:97275905-97310538_R		lincRNA:chr11:97275905-97310538 reverse strand	chr11:97309819-97309760	7.69	10.05	0.20

468	A_52_P169595	NM_177669	Skint11	Mus musculus selection and upkeep of intraepithelial T cells 11 (Skint11), transcript variant 2, mRNA [NM_177669]	chr4:113917416-113917475	5.04	7.40	0.20
469	A_51_P433824	NM_009415	Tpi1	Mus musculus triosephosphate isomerase 1 (Tpi1), mRNA [NM_009415]	chr6:124761006-124760947	10.11	12.47	0.20
470	A_55_P1961395	NM_010329	Pdpn	Mus musculus podoplanin (Pdpn), mRNA [NM_010329]	chr4:142857398-142857339	8.89	11.25	0.19
471	A_66_P104923	ENSMUST00000103548	LOC100046359	PREDICTED: Mus musculus similar to Ighg1 protein (LOC100046359), mRNA [XM_001473125]	chr12:117158672-117158613	6.11	8.47	0.19
472	A_30_P01025804	chr11:68200716-68214458_F		lincRNA:chr11:68200716-68214458 forward strand	chr11:68211545-68211604	5.47	7.83	0.19
473	A_30_P01033301	chr12:111322030-111323387_F		lincRNA:chr12:111322030-111323387 forward strand	chr12:111322843-111322902	5.62	7.99	0.19
474	A_55_P2085485	NM_022654	Lrdd	Mus musculus leucine-rich and death domain containing (Lrdd), mRNA [NM_022654]	chr7:148624475-148624416	7.41	9.79	0.19
475	A_55_P2047155	NM_001159569	Meis2	Mus musculus Meis homeobox 2 (Meis2), transcript variant 5, mRNA [NM_001159569]	chr2:115689700-115689641	9.03	11.41	0.19
476	A_30_P01025936	chr8:124355710-124401160_R		lincRNA:chr8:124355710-124401160 reverse strand	chr8:124397661-124397602	6.34	8.72	0.19
477	A_55_P2064962	NM_010329	Pdpn	Mus musculus podoplanin (Pdpn), mRNA [NM_010329]	chr4:142859146-142858329	7.63	10.01	0.19
478	A_55_P2089472	NM_001163483	1700020A23Rik	Mus musculus RIKEN cDNA 1700020A23 gene (1700020A23Rik), transcript variant 1, mRNA [NM_001163483]	chr2:130231747-130231806	5.78	8.16	0.19
479	A_30_P01019131	chr17:21752466-21793451_F		lincRNA:chr17:21752466-21793451 forward strand	chr17:21766230-21766289	6.31	8.70	0.19
480	A_52_P249424	NM_001025257	Vegfa	Mus musculus vascular endothelial growth factor A (Vegfa), transcript variant 3, mRNA [NM_001025257]	chr17:46162435-46162376	9.08	11.47	0.19
481	A_66_P130647	XM_001480325	LOC100048479	PREDICTED: Mus musculus similar to hepatocyte nuclear factor 6 beta (LOC100048479), mRNA [XM_001480325]	chr9:74737402-74737461	6.82	9.21	0.19
482	A_55_P1957363	XM_001472920	Gm2249	PREDICTED: Mus musculus similar to mucin (LOC100039458), mRNA [XM_001472920]	chr18:81886366-81886307	7.11	9.50	0.19
483	A_55_P1992218	XM_001476624	LOC100046688	PREDICTED: Mus musculus hypothetical protein LOC100046688 (LOC100046688), mRNA [XM_001476624]	chrX:75111329-75111388	6.83	9.23	0.19

484	A_30_P01031936	chr1:63319842-63349796_F		lincRNA:chr1:63319842-63349796 forward strand	chr1:63327389-63327448	8.80	11.20	0.19
485	A_30_P01029556	chr4:101040523-101080248_R		lincRNA:chr4:101040523-101080248 reverse strand	chr4:101077369-101077310	5.53	7.93	0.19
486	A_30_P01029897	chr9:43738583-43757007_F		lincRNA:chr9:43738583-43757007 forward strand	chr9:43747333-43747392	6.98	9.38	0.19
487	A_55_P2046877	NM_008239	Foxq1	Mus musculus forkhead box Q1 (Foxq1), mRNA [NM_008239]	chr13:31652206-31652265	5.78	8.18	0.19
488	A_55_P2069597	NM_028841	Tspan17	Mus musculus tetraspanin 17 (Tspan17), mRNA [NM_028841]	chr13:54897519-54897578	7.97	10.38	0.19
489	A_55_P2180504	NM_027697	4933413G19Rik	Mus musculus RIKEN cDNA 4933413G19 gene (4933413G19Rik), mRNA [NM_027697]	chr6:128334947-128335006	6.03	8.44	0.19
490	A_55_P2105858	NM_030693	Atf5	Mus musculus activating transcription factor 5 (Atf5), transcript variant 1, mRNA [NM_030693]	chr7:52067686-52067627	11.63	14.05	0.19
491	A_51_P153557	NM_175199	Hspa12a	Mus musculus heat shock protein 12A (Hspa12a), mRNA [NM_175199]	chr19:58870580-58870521	7.99	10.40	0.19
492	A_55_P2059095	XM_001000159	Fignl2	PREDICTED: Mus musculus predicted gene, EG668225 (EG668225), mRNA [XM_001000159]	chr15:100882557-100882498	8.58	11.00	0.19
493	A_51_P338600	NM_025387	Tmem14c	Mus musculus transmembrane protein 14C (Tmem14c), mRNA [NM_025387]	chr13:41117584-41117643	8.31	10.73	0.19
494	A_55_P1987284	NM_207574	Olfr1383	Mus musculus olfactory receptor 1383 (Olfr1383), mRNA [NM_207574]	chr11:49338102-49338161	6.66	9.08	0.19
495	A_52_P130787	NM_013568	Kcna6	Mus musculus potassium voltage-gated channel, shaker-related, subfamily, member 6 (Kcna6), mRNA [NM_013568]	chr6:126658483-126658424	9.09	11.52	0.19
496	A_30_P01017453	chr15:92174262-92196074_F		lincRNA:chr15:92174262-92196074 forward strand	chr15:92195960-92196019	6.61	9.04	0.19
497	A_51_P184300	NM_010087	Dtna	Mus musculus dystrobrevin alpha (Dtna), transcript variant 2, mRNA [NM_010087]	chr18:23748714-23748773	8.80	11.24	0.19
498	A_55_P1997061	XR_033957	Gm5452	PREDICTED: Mus musculus similar to lactate dehydrogenase 1, A chain (LOC432789), misc RNA [XR_033957]	chr13:83973142-83973201	10.05	12.48	0.19
499	A_52_P522379	NM_178084	B230120H23Rik	Mus musculus RIKEN cDNA B230120H23 gene (B230120H23Rik), transcript variant 2, mRNA [NM_178084]	chr2:72227533-72227592	8.00	10.44	0.19
500	A_30_P01030558	chr2:157361470-157379813_F		lincRNA:chr2:157361470-157379813 forward strand	chr2:157371645-157371704	9.20	11.64	0.19

501	A_55_P1999888	NM_011998	Chst4	Mus musculus carbohydrate (chondroitin 6/keratan) sulfotransferase 4 (Chst4), mRNA [NM_011998]	chr8:112553204-112553145	7.26	9.70	0.19
502	A_52_P387009	NM_028133	Egln3	Mus musculus EGL nine homolog 3 (C. elegans) (Egln3), mRNA [NM_028133]	chr12:55281012-55280953	5.95	8.39	0.18
503	A_30_P01031633	chr10:120058696-120076196_R		lincRNA:chr10:120058696-120076196 reverse strand	chr10:120069221-120069162	5.16	7.62	0.18
504	A_30_P01025057	chr9:75316422-75332567_F		lincRNA:chr9:75316422-75332567 forward strand	chr9:75330788-75330847	7.15	9.61	0.18
505	A_55_P2279927	AK148051	5930430L01Rik	Mus musculus B16 F10Y cells cDNA, RIKEN full-length enriched library, clone:G370004A16 product:unclassifiable, full insert sequence. [AK148051]	chr5:149801708-149801649	6.07	8.53	0.18
506	A_55_P1978920	XM_001476569	LOC100041553	PREDICTED: Mus musculus similar to Ubtf protein (LOC100041553), mRNA [XM_001476569]	chrY_random:44926233-44926292	7.49	9.95	0.18
507	A_52_P38908	NM_001190352	Tmem132b	Mus musculus transmembrane protein 132B (Tmem132b), mRNA [NM_001190352]	chr5:126269235-126269294	10.70	13.16	0.18
508	A_30_P01023385	chr15:38639259-38639546_R		lincRNA:chr15:38639259-38639546 reverse strand	chr15:38639358-38639299	7.86	10.32	0.18
509	A_51_P445841	NM_145470	Depdc6	Mus musculus DEP domain containing 6 (Depdc6), transcript variant 1, mRNA [NM_145470]	chr15:55083753-55083812	5.78	8.24	0.18
510	A_55_P2016888	ENSMUST00000105884			chr4:133614852-133614911	5.88	8.35	0.18
511	A_55_P2406802	AK015759	4930511J24Rik	Mus musculus adult male testis cDNA, RIKEN full-length enriched library, clone:4930511J24 product:unclassifiable, full insert sequence. [AK015759]	chr12:111151646-111151705	5.10	7.57	0.18
512	A_30_P01029221	chr18:38302523-38302756_R		lincRNA:chr18:38302523-38302756 reverse strand	chr18:38302583-38302524	5.57	8.05	0.18
513	A_66_P102499	ENSMUST00000103313		Mouse Ig rearranged kappa-chain mRNA V-J2-region, hybridoma A6.1, partial cds. [M33559]	chr6:67904441-67904382	5.66	8.13	0.18
514	A_55_P1958280	NM_001162485	Arrdc1	Mus musculus arrestin domain containing 1 (Arrdc1), transcript variant 1, mRNA [NM_001162485]	chr2:24780934-24780875	8.48	10.95	0.18
515	A_30_P01033603	chr2:33496712-33501183_F		lincRNA:chr2:33496712-33501183 forward strand	chr2:33500968-33501027	5.78	8.25	0.18
516	A_30_P01017527	chr4:3406175-3443875_F		lincRNA:chr4:3406175-3443875 forward strand	chr4:3435225-3435284	6.64	9.12	0.18

517	A_30_P01020585	chr2:157361470-157379813_F		lincRNA:chr2:157361470-157379813 forward strand	chr2:157371766-157371825	7.83	10.31	0.18
518	A_30_P01029070	chr18:35204950-35216650_F		lincRNA:chr18:35204950-35216650 forward strand	chr18:35213972-35214031	5.59	8.07	0.18
519	A_55_P2021011	NM_030207	Sfi1	Mus musculus Sfi1 homolog, spindle assembly associated (yeast) (Sfi1), mRNA [NM_030207]	chr11:3086127-3086068	7.60	10.09	0.18
520	A_51_P168630	NM_010635	Klf1	Mus musculus Kruppel-like factor 1 (erythroid) (Klf1), mRNA [NM_010635]	chr8:87428881-87428940	7.52	10.00	0.18
521	A_55_P2373774	AK021026	B430319H21Rik	Mus musculus 4 days neonate male adipose cDNA, RIKEN full-length enriched library, clone:B430319H21 product:hypothetical protein, full insert sequence. [AK021026]	chr11:63001848-63001907	6.82	9.31	0.18
522	A_52_P635015	NM_027677	Gpr39	Mus musculus G protein-coupled receptor 39 (Gpr39), mRNA [NM_027677]	chr1:127769282-127769341	5.86	8.36	0.18
523	A_51_P300506	NM_183405	Cox6b2	Mus musculus cytochrome c oxidase subunit VIb polypeptide 2 (Cox6b2), transcript variant 1, mRNA [NM_183405]	chr7:4703547-4703488	7.94	10.44	0.18
524	A_30_P01026827	chr2:167404245-167414345_F		lincRNA:chr2:167404245-167414345 forward strand	chr2:167404778-167404837	6.64	9.15	0.18
525	A_55_P2084303	NM_010917	Nid1	Mus musculus nidogen 1 (Nid1), mRNA [NM_010917]	chr13:13530138-13555992	6.66	9.16	0.18
526	A_30_P01033034	chr2:77807600-77836075_R		lincRNA:chr2:77807600-77836075 reverse strand	chr2:77819272-77819213	5.80	8.30	0.18
527	A_55_P2031317	NM_146945	Olfr345	Mus musculus olfactory receptor 345 (Olfr345), mRNA [NM_146945]	chr2:36496437-36496496	5.82	8.33	0.18
528	A_55_P2344753	AK044369	A930009L07Rik	Mus musculus adult retina cDNA, RIKEN full-length enriched library, clone:A930009L07 product:unclassifiable, full insert sequence. [AK044369]	chr15:72338337-72338278	6.59	9.09	0.18
529	A_55_P1993463	XR_031039	LOC676303	PREDICTED: Mus musculus similar to L-lactate dehydrogenase A chain (LDH-A) (LDH muscle subunit) (LDH-M) (LOC676303), misc RNA [XR_031039]		8.47	10.98	0.18
530	A_55_P2141876	NM_001136069	Ldha	Mus musculus lactate dehydrogenase A (Ldha), transcript variant 2, mRNA [NM_001136069]	chr7:54103010-54103069	8.66	11.17	0.18
531	A_30_P01028270	chr15:3946037-3972747_F		lincRNA:chr15:3946037-3972747 forward strand	chr15:3946069-3946128	7.35	9.87	0.18

532	A_55_P2006255	NM_001009935	Txnip	Mus musculus thioredoxin interacting protein (Txnip), transcript variant 1, mRNA [NM_001009935]	chr3:96364206-96364265	8.61	11.13	0.18
533	A_55_P1964163	NM_177785	BC049635	Mus musculus cDNA sequence BC049635 (BC049635), mRNA [NM_177785]	chr4:42881440-42881381	6.99	9.50	0.18
534	A_52_P163021	NM_182959	Slc17a8	Mus musculus solute carrier family 17 (sodium-dependent inorganic phosphate cotransporter), member 8 (Slc17a8), mRNA [NM_182959]	chr10:89037689-89037630	5.42	7.94	0.17
535	A_30_P01030849	chr2:157361470-157379813_F		lincRNA:chr2:157361470-157379813 forward strand	chr2:157371888-157371947	6.72	9.25	0.17
536	A_52_P175634	NM_001077424	A4gnt	Mus musculus alpha-1,4-N-acetylglucosaminyltransferase (A4gnt), mRNA [NM_001077424]	chr9:99521093-99521152	6.31	8.85	0.17
537	A_55_P2008434	XM_001476747	Gm3450	PREDICTED: Mus musculus hypothetical protein LOC100041646 (LOC100041646), mRNA [XM_001476747]	chr5:64439444-64439503	5.66	8.19	0.17
538	A_52_P638895	NM_001025250	Vegfa	Mus musculus vascular endothelial growth factor A (Vegfa), transcript variant 1, mRNA [NM_001025250]	chr17:46154446-46154387	9.98	12.53	0.17
539	A_52_P146865	NM_053136	Pcdhb11	Mus musculus protocadherin beta 11 (Pcdhb11), mRNA [NM_053136]	chr18:37583696-37583755	7.45	10.01	0.17
540	A_51_P498720	NM_009784	Cacna2d1	Mus musculus calcium channel, voltage-dependent, alpha2/delta subunit 1 (Cacna2d1), transcript variant e, mRNA [NM_009784]	chr5:15876550-15876609	8.77	11.33	0.17
541	A_51_P182303	NM_007743	Col1a2	Mus musculus collagen, type I, alpha 2 (Col1a2), mRNA [NM_007743]	chr6:4490619-4490678	7.44	10.02	0.17
542	A_66_P118212	NM_001011832	Olfr1490	Mus musculus olfactory receptor 1490 (Olfr1490), mRNA [NM_001011832]	chr19:13729818-13729877	5.80	8.38	0.17
543	A_55_P2151950	NM_146763	Olfr1406	Mus musculus olfactory receptor 1406 (Olfr1406), mRNA [NM_146763]	chr1:175113999-175113940	5.93	8.51	0.17
544	A_55_P1958285	NM_178408	Arrdc1	Mus musculus arrestin domain containing 1 (Arrdc1), transcript variant 2, mRNA [NM_178408]	chr2:24782493-24782434	8.51	11.10	0.17
545	A_55_P2005307	NM_010695	Lcn4	Mus musculus lipocalin 4 (Lcn4), mRNA [NM_010695]	chr2:26524927-26524868	5.36	7.96	0.16
546	A_30_P01033089	chr11:97276846-97310021_F		lincRNA:chr11:97276846-97310021 forward strand	chr11:97309778-97309837	7.20	9.81	0.16
547	A_52_P72434	NM_008439	Khk	Mus musculus ketohexokinase (Khk), mRNA [NM_008439]	chr5:31233555-31233614	7.87	10.49	0.16
548	A_55_P2013236	NM_009789	S100g	Mus musculus S100 calcium binding protein G (S100g), mRNA [NM_009789]	chrX:159399983-159399924	5.90	8.53	0.16

549	A_51_P176086	NM_146187	Ffar2	Mus musculus free fatty acid receptor 2 (Ffar2), transcript variant 1, mRNA [NM_146187]	chr7:31604208-31604149	7.75	10.38	0.16
550	A_55_P2084820	NM_013469	Anxa11	Mus musculus annexin A11 (Anxa11), mRNA [NM_013469]	chr14:26694168-26694227	5.66	8.29	0.16
551	A_55_P1953618	NM_146974	Olfr1262	Mus musculus olfactory receptor 1262 (Olfr1262), mRNA [NM_146974]	chr2:89843420-89843479	5.78	8.41	0.16
552	A_55_P2104312	NM_013618	Olfr66	Mus musculus olfactory receptor 66 (Olfr66), mRNA [NM_013618]	chr7:111029879-111029820	6.16	8.80	0.16
553	A_55_P2074631	NM_007755	Cpeb1	Mus musculus cytoplasmic polyadenylation element binding protein 1 (Cpeb1), mRNA [NM_007755]	chr7:88492396-88492337	7.59	10.23	0.16
554	A_52_P1092823	NM_010573	Irx1	Mus musculus Iroquois related homeobox 1 (Drosophila) (Irx1), mRNA [NM_010573]	chr13:72095794-72095735	6.27	8.92	0.16
555	A_66_P101600	NM_008482	Lamb1-1	Mus musculus laminin B1 subunit 1 (Lamb1-1), mRNA [NM_008482]	chr12:32014411-32014470	8.01	10.66	0.16
556	A_30_P01020592	chrX:123306246-123307974_F		lincRNA:chrX:123306246-123307974 forward strand	chrX:123307746-123307805	5.40	8.06	0.16
557	A_30_P01026521	chr17:7838192-7853103_F		lincRNA:chr17:7838192-7853103 forward strand	chr17:7853044-7853103	5.70	8.36	0.16
558	A_52_P470316	NM_033521	Laptm4b	Mus musculus lysosomal-associated protein transmembrane 4B (Laptm4b), mRNA [NM_033521]	chr15:34213600-34213659	7.21	9.87	0.16
559	A_52_P537571	NM_011635	Trap1a	Mus musculus tumor rejection antigen P1A (Trap1a), mRNA [NM_011635]	chrX:135871399-135871458	6.07	8.73	0.16
560	A_30_P01024520	chr9:114412295-114418384_F		lincRNA:chr9:114412295-114418384 forward strand	chr9:114418261-114418320	7.31	9.98	0.16
561	A_52_P350750	NM_015730	Chrna4	Mus musculus cholinergic receptor, nicotinic, alpha polypeptide 4 (Chrna4), mRNA [NM_015730]	chr2:180757368-180757309	10.02	12.69	0.16
562	A_51_P151126	NM_013706	Cd52	Mus musculus CD52 antigen (Cd52), mRNA [NM_013706]	chr4:133649564-133649505	6.07	8.75	0.16
563	A_30_P01031658	chr2:157361470-157379813_R		lincRNA:chr2:157361470-157379813 reverse strand	chr2:157371840-157371781	7.24	9.93	0.16
564	A_51_P507051	NM_021560	Bhlhe22	Mus musculus basic helix-loop-helix family, member e22 (Bhlhe22), mRNA [NM_021560]	chr3:17956925-17956984	6.81	9.49	0.16
565	A_55_P2143251	ENSMUST00000105034		Putative uncharacterized protein [Source:UniProtKB/TrEMBL;Acc:Q3TQL3] [ENSMUST00000105034]	chr4:120569114-120569173	8.27	10.96	0.16

566	A_55_P1975155	NM_001161767	Galnt6	Mus musculus UDP-N-acetyl-alpha-D-galactosamine:polypeptide N-acetylgalactosaminyltransferase 6 (Galnt6), transcript variant 1, mRNA [NM_001161767]	chr15:100522296-100522237	6.59	9.27	0.16
567	A_51_P436068	NM_007412	Gpr182	Mus musculus G protein-coupled receptor 182 (Gpr182), mRNA [NM_007412]	chr10:127186816-127186757	6.82	9.51	0.16
568	A_51_P472726	NM_145978	Pdlim2	Mus musculus PDZ and LIM domain 2 (Pdlim2), mRNA [NM_145978]	chr14:70564285-70564226	6.68	9.37	0.16
569	A_30_P01022828	chr18:82862340-82875566_F		lincRNA:chr18:82862340-82875566 forward strand	chr18:82875058-82875117	5.72	8.41	0.16
570	A_55_P2013273	XR_034534	Gm13937	PREDICTED: Mus musculus similar to lactate dehydrogenase 1, A chain (LOC623145), misc RNA [XR_034534]	chr2:110049625-110049566	8.95	11.65	0.15
571	A_51_P281333	NM_018784	St3gal6	Mus musculus ST3 beta-galactoside alpha-2,3-sialyltransferase 6 (St3gal6), mRNA [NM_018784]	chr16:58470781-58470722	6.19	8.89	0.15
572	A_51_P375754	NM_010462	Hoxc10	Mus musculus homeobox C10 (Hoxc10), mRNA [NM_010462]	chr15:102801857-102801916	6.31	9.02	0.15
573	A_30_P01021865	chr2:157361470-157379813_R		lincRNA:chr2:157361470-157379813 reverse strand	chr2:157371720-157371661	8.61	11.31	0.15
574	A_30_P01031241	chr2:59235155-59242518_F		lincRNA:chr2:59235155-59242518 forward strand	chr2:59237195-59237254	9.62	12.33	0.15
575	A_30_P01025068	H19		lincRNA:chr7:149761435-149764019 reverse strand	chr7:149761815-149761756	7.73	10.45	0.15
576	A_30_P01029054	chr8:122712014-122732520_R		lincRNA:chr8:122712014-122732520 reverse strand	chr8:122724409-122724350	6.41	9.13	0.15
577	A_55_P2075510	ENSMUST00000103508		Mus musculus partial mRNA for immunoglobulin heavy chain variable region (IGHV gene), clone 3. [FN652764]	chr12:116002805-116002746	5.62	8.34	0.15
578	A_55_P2180996	NM_182929	Rims3	Mus musculus regulating synaptic membrane exocytosis 3 (Rims3), mRNA [NM_182929]	chr4:120563927-120563986	7.62	10.34	0.15
579	A_55_P2064776	NR_024257	4930412O13Rik	Mus musculus RIKEN cDNA 4930412O13 gene (4930412O13Rik), non-coding RNA [NR_024257]	chr2:9808308-9808367	6.11	8.83	0.15
580	A_51_P486217	NM_175638	Wnk4	Mus musculus WNK lysine deficient protein kinase 4 (Wnk4), mRNA [NM_175638]	chr11:101138198-101138364	6.83	9.56	0.15
581	A_30_P01025875	chr17:41231055-41249764_R		lincRNA:chr17:41231055-41249764 reverse strand	chr17:41239083-41239024	9.20	11.93	0.15
582	A_30_P01019562	chr10:116814196-116920696_F		lincRNA:chr10:116814196-116920696 forward strand	chr10:116846961-116847020	5.53	8.26	0.15

583	A_55_P2049211	NR_003517	Pisd-ps1	Mus musculus phosphatidylserine decarboxylase, pseudogene 1 (Pisd-ps1), non-coding RNA [NR_003517]	chr11:3031786-3031845	11.51	14.25	0.15
584	A_30_P01032464	chr10:69667411-69678033_F		lincRNA:chr10:69667411-69678033 forward strand	chr10:69677914-69677973	5.97	8.72	0.15
585	A_52_P354744	NM_011401	Slc2a3	Mus musculus solute carrier family 2 (facilitated glucose transporter), member 3 (Slc2a3), mRNA [NM_011401]	chr6:122678134-122678075	8.21	10.96	0.15
586	A_52_P89683	XM_001474667	Gm14470	PREDICTED: Mus musculus similar to lactate dehydrogenase 1, A chain (LOC385319), mRNA [XM_001474667]	chrX:10258406-10258464	8.27	11.03	0.15
587	A_55_P2183735	NM_177298	Pisd	Mus musculus phosphatidylserine decarboxylase (Pisd), mRNA [NM_177298]	chr5:33079477-33079418	9.34	12.10	0.15
588	A_52_P289173	ENSMUST00000065087		Riken cDNA C130021I20 gene Gene [Source:MGI Symbol;Acc:MGI:3639863] [ENSMUST00000065087]	chr2:33501600-33501659	5.70	8.48	0.15
589	A_55_P1991617	XM_001473989	2600006L11Rik	PREDICTED: Mus musculus RIKEN cDNA 2600006L11 gene (2600006L11Rik), mRNA [XM_001473989]	chr9:63928583-63928642	6.53	9.32	0.15
590	A_55_P1977473	NM_023118	Dab2	Mus musculus disabled homolog 2 (Drosophila) (Dab2), transcript variant 1, mRNA [NM_023118]	chr15:6390616-6390675	6.31	9.10	0.15
591	A_52_P586821	NM_010317	Gng4	Mus musculus guanine nucleotide binding protein (G protein), gamma 4 (Gng4), mRNA [NM_010317]	chr13:13919304-13919363	9.21	11.99	0.15
592	A_51_P248122	NM_133234	Bbc3	Mus musculus BCL2 binding component 3 (Bbc3), mRNA [NM_133234]	chr7:16903403-16903462	8.95	11.75	0.14
593	A_30_P01018386	chr17:41231055-41249764_R		lincRNA:chr17:41231055-41249764 reverse strand	chr17:41239023-41238964	10.08	12.87	0.14
594	A_55_P2047809	NM_175692	Snhg11	Mus musculus small nucleolar RNA host gene 11 (non-protein coding) (Snhg11), mRNA [NM_175692]	chr2:158206508-158206567	7.53	10.33	0.14
595	A_55_P2120080	ENSMUST00000110633		zinc finger, MYND domain containing 11 Gene [Source:MGI (curated);Acc:MGI:1913755] [ENSMUST00000110633]	chr13:9689645-9689586	10.00	12.80	0.14
596	A_30_P01021775	chr17:39960567-39992817_F		lincRNA:chr17:39960567-39992817 forward strand	chr17:39982106-39982165	13.32	16.14	0.14
597	A_55_P2126072	XM_001472689	Gm2160	PREDICTED: Mus musculus similar to SPPL3 protein (LOC100039327), mRNA [XM_001472689]	chr6:35882916-35882975	5.10	7.92	0.14

598	A_55_P2148708	XR_031767	Gm6705	PREDICTED: Mus musculus similar to Lysosomal-associated protein transmembrane 4B (LOC626785), misc RNA [XR_031767]	chr17:16985913-16985972	9.25	12.08	0.14
599	A_30_P01033119	chr2:157361470-157379813_F		lincRNA:chr2:157361470-157379813 forward strand	chr2:157371705-157371764	8.43	11.29	0.14
600	A_52_P460734	NM_198408	Crhbp	Mus musculus corticotropin releasing hormone binding protein (Crhbp), mRNA [NM_198408]	chr13:96201962-96201903	6.94	9.83	0.14
601	A_52_P265979	NM_029702	Arfrp1	Mus musculus ADP-ribosylation factor related protein 1 (Arfrp1), transcript variant 2, mRNA [NM_029702]	chr2:181093013-181092954	7.14	10.03	0.14
602	A_55_P2184841	NM_146605	Olfr828	Mus musculus olfactory receptor 828 (Olfr828), mRNA [NM_146605]	chr9:18619857-18619798	5.95	8.85	0.13
603	A_51_P518841	NM_153111	Fev	Mus musculus FEV (ETS oncogene family) (Fev), mRNA [NM_153111]	chr1:74928319-74928260	6.97	9.87	0.13
604	A_30_P01021588	H19		lincRNA:chr7:149761435-149764019 reverse strand	chr7:149761752-149761693	7.44	10.35	0.13
605	A_55_P2141878	NM_001136069	Ldha	Mus musculus lactate dehydrogenase A (Ldha), transcript variant 2, mRNA [NM_001136069]	chr7:54107294-54107353	10.19	13.11	0.13
606	A_55_P2000224	NM_001042580	Cd63	Mus musculus CD63 antigen (Cd63), transcript variant 1, mRNA [NM_001042580]	chr10:128349813-128349872	8.68	11.60	0.13
607	A_52_P281702	NM_010518	Igfbp5	Mus musculus insulin-like growth factor binding protein 5 (Igfbp5), mRNA [NM_010518]	chr1:72904888-72904829	8.73	11.67	0.13
608	A_55_P2141256	XM_001474975	Gm1758	PREDICTED: Mus musculus gene model 1758, (NCBI) (Gm1758), mRNA [XM_001474975]	chr16:14509296-14509355	5.95	8.90	0.13
609	A_30_P01021374	chr6:47692090-47713542_F		lincRNA:chr6:47692090-47713542 forward strand	chr6:47694236-47694295	8.85	11.81	0.13
610	A_55_P2134591	NM_178776	BC049715	Mus musculus cDNA sequence BC049715 (BC049715), mRNA [NM_178776]	chr6:136788994-136789053	6.15	9.12	0.13
611	A_30_P01021905	chr4:8606650-8614031_R		lincRNA:chr4:8606650-8614031 reverse strand	chr4:8610224-8610165	6.15	9.13	0.13
612	A_55_P2132345	NM_024452	Luzp1	Mus musculus leucine zipper protein 1 (Luzp1), mRNA [NM_024452]	chr4:136099154-136099213	7.42	10.41	0.13
613	A_52_P356698	NM_013665	Shox2	Mus musculus short stature homeobox 2 (Shox2), mRNA [NM_013665]	chr3:66779814-66779435	6.24	9.24	0.13
614	A_52_P64356	NM_010097	Sparcl1	Mus musculus SPARC-like 1 (Sparcl1), mRNA [NM_010097]	chr5:104508208-104508149	6.40	9.41	0.12

615	A_55_P1979645	NM_011309	S100a1	Mus musculus S100 calcium binding protein A1 (S100a1), mRNA [NM_011309]	chr3:90315250-90315191	6.76	9.77	0.12
616	A_55_P2073965	NM_178776	BC049715	Mus musculus cDNA sequence BC049715 (BC049715), mRNA [NM_178776]	chr6:136789015-136789074	6.07	9.08	0.12
617	A_51_P491350	NM_009932	Col4a2	Mus musculus collagen, type IV, alpha 2 (Col4a2), mRNA [NM_009932]	chr8:11448840-11448899	7.88	10.90	0.12
618	A_51_P448971	NM_033610	Sncb	Mus musculus synuclein, beta (Sncb), mRNA [NM_033610]	chr13:54861250-54860865	8.32	11.34	0.12
619	A_55_P2097962	XM_001481064	9030622O22Rik	PREDICTED: Mus musculus hypothetical protein LOC100048732 (LOC100048732), mRNA [XM_001481064]	chr2:147798641-147798582	6.03	9.07	0.12
620	A_52_P238468	NM_001080815	Gipr	Mus musculus gastric inhibitory polypeptide receptor (Gipr), mRNA [NM_001080815]	chr7:19742545-19742486	7.59	10.63	0.12
621	A_30_P01022639	chr6:47692090-47713542_F		lincRNA:chr6:47692090-47713542 forward strand	chr6:47693198-47693257	8.41	11.49	0.12
622	A_55_P2101137	NM_146350	Olfr1123	Mus musculus olfactory receptor 1123 (Olfr1123), mRNA [NM_146350]	chr2:87259119-87259178	5.70	8.78	0.12
623	A_55_P1988384	NM_007515	Slc7a3	Mus musculus solute carrier family 7 (cationic amino acid transporter, y+ system), member 3 (Slc7a3), mRNA [NM_007515]	chrX:98274703-98274644	7.18	10.29	0.12
624	A_66_P104265	ENSMUST00000068158		RIKEN cDNA 4930578G10 gene Gene [Source:MGI Symbol;Acc:MGI:1923202] [ENSMUST00000068158]	chr4:42749484-42774566	7.29	10.42	0.11
625	A_55_P2072138	XM_001474110	Gm3789	PREDICTED: Mus musculus similar to phosphatidylserine decarboxylase (LOC100045344), mRNA [XM_001474110]	chr14:19875654-19875713	10.07	13.21	0.11
626	A_51_P427663	NM_007725	Cnn2	Mus musculus calponin 2 (Cnn2), mRNA [NM_007725]	chr10:79457785-79457844	6.84	9.98	0.11
627	A_55_P1981604	NM_207146	Olfr670	Mus musculus olfactory receptor 670 (Olfr670), mRNA [NM_207146]	chr7:112108365-112108306	5.95	9.09	0.11
628	A_55_P2001998	NM_145459	Zfp503	Mus musculus zinc finger protein 503 (Zfp503), mRNA [NM_145459]	chr14:22803985-22803926	7.24	10.41	0.11
629	A_55_P2040549	NM_008888	Phox2b	Mus musculus paired-like homeobox 2b (Phox2b), mRNA [NM_008888]	chr5:67485826-67485767	6.22	9.39	0.11
630	A_55_P1967733	NM_001033276	Mll2	Mus musculus myeloid/lymphoid or mixed-lineage leukemia 2 (Mll2), mRNA [NM_001033276]	chr15:98663049-98662990	7.98	11.16	0.11
631	A_55_P2150966	NM_008718	Npas1	Mus musculus neuronal PAS domain protein 1 (Npas1), mRNA [NM_008718]	chr7:17046739-17046680	7.63	10.82	0.11

632	A_51_P267278	NM_021301	Slc15a2	Mus musculus solute carrier family 15 (H+/peptide transporter), member 2 (Slc15a2), transcript variant 1, mRNA [NM_021301]	chr16:36750662-36750603	5.86	9.05	0.11
633	A_55_P1961127	NR_001592	H19	Mus musculus H19 fetal liver mRNA (H19), non-coding RNA [NR_001592]	chr7:149761551-149761492	8.68	11.88	0.11
634	A_55_P2000289	NM_011839	Mab21l2	Mus musculus mab-21-like 2 (C. elegans) (Mab21l2), mRNA [NM_011839]	chr3:86350802-86350743	9.20	12.41	0.11
635	A_55_P1985911	NM_010919	Nkx2-2	Mus musculus NK2 transcription factor related, locus 2 (Drosophila) (Nkx2-2), transcript variant 1, mRNA [NM_010919]	chr2:147009008-147008949	7.38	10.63	0.11
636	A_55_P2013357	NM_023132	Renbp	Mus musculus renin binding protein (Renbp), transcript variant 1, mRNA [NM_023132]	chrX:71167750-71167691	6.61	9.87	0.10
637	A_55_P2026950	ENSMUST00000101381		RIKEN cDNA C530030P08 gene Gene [Source:MGI Symbol;Acc:MGI:2144664] [ENSMUST00000101381]	chr11:32625904-32625963	8.29	11.56	0.10
638	A_51_P267933	NM_175692	Snhg11	Mus musculus small nucleolar RNA host gene 11 (non-protein coding) (Snhg11), mRNA [NM_175692]	chr2:158211554-158211613	10.76	14.05	0.10
639	A_51_P449638	NR_033616	Pldi	Mus musculus polymorphic derived intron containing (Pldi), non-coding RNA [NR_033616]	chr10:60391434-60391375	5.85	9.14	0.10
640	A_51_P327206	NM_011839	Mab21l2	Mus musculus mab-21-like 2 (C. elegans) (Mab21l2), mRNA [NM_011839]	chr3:86350340-86350281	6.80	10.10	0.10
641	A_55_P2162712	NM_173391	Tph2	Mus musculus tryptophan hydroxylase 2 (Tph2), mRNA [NM_173391]	chr10:114516743-114516684	6.25	9.58	0.10
642	A_30_P01031637	chr1:183951156-183986156_F		lincRNA:chr1:183951156-183986156 forward strand	chr1:183958368-183958427	5.57	8.91	0.10
643	A_52_P86693	NM_026790	Ifi27l1	Mus musculus interferon, alpha-inducible protein 27 like 1 (Ifi27l1), transcript variant 1, mRNA [NM_026790]	chr12:104678353-104678412	7.47	10.80	0.10
644	A_52_P515769	NM_017378	Pcdh12	Mus musculus protocadherin 12 (Pcdh12), mRNA [NM_017378]	chr18:38426927-38426868	5.49	8.84	0.10
645	A_52_P652336	ENSMUST00000018842	Lhx1	LIM homeobox protein 1 Gene [Source:MGI (curated);Acc:MGI:99783] [ENSMUST00000018842]	chr11:84331947-84331888	7.41	10.77	0.10
646	A_30_P01018620	chr17:39980973-39981876_R		lincRNA:chr17:39980973-39981876 reverse strand	chr17:39981446-39981387	9.74	13.15	0.10

647	A_55_P1967149	NM_008393	Irx3	Mus musculus Iroquois related homeobox 3 (Drosophila) (Irx3), mRNA [NM_008393]	chr8:94322535-94322476	5.16	8.63	0.09
648	A_51_P376445	NM_008818	Rhox5	Mus musculus reproductive homeobox 5 (Rhox5), mRNA [NM_008818]		7.05	10.52	0.09
649	A_51_P124254	NM_009931	Col4a1	Mus musculus collagen, type IV, alpha 1 (Col4a1), mRNA [NM_009931]	chr8:11199068-11199009	7.93	11.41	0.09
650	A_30_P01025132	chr17:3004525-3086000_F		lincRNA:chr17:3004525-3086000 forward strand	chr17:3081683-3081742	7.42	10.92	0.09
651	A_55_P2067453	NM_028841	Tspan17	Mus musculus tetraspanin 17 (Tspan17), mRNA [NM_028841]	chr13:54898070-54898129	9.44	12.94	0.09
652	A_51_P161323	NM_175540	Eda2r	Mus musculus ectodysplasin A2 receptor (Eda2r), transcript variant 2, mRNA [NM_175540]	chrX:94531379-94531320	6.13	9.63	0.09
653	A_55_P2094484	NM_001039223	Gm14137	Mus musculus predicted gene 14137 (Gm14137), mRNA [NM_001039223]	chr2:119002407-119002466	6.22	9.75	0.09
654	A_51_P515605	NM_009930	Col3a1	Mus musculus collagen, type III, alpha 1 (Col3a1), mRNA [NM_009930]	chr1:45405589-45405648	6.51	10.06	0.09
655	A_52_P233441	NM_008090	Gata2	Mus musculus GATA binding protein 2 (Gata2), mRNA [NM_008090]	chr6:88156362-88156421	5.66	9.24	0.08
656	A_66_P122158	NR_003518	Pisd-ps3	Mus musculus phosphatidylserine decarboxylase, pseudogene 3 (Pisd-ps3), non-coding RNA [NR_003518]	chr11:003030755-003030814	10.17	13.79	0.08
657	A_65_P19832	NM_013697	Ttr	Mus musculus transthyretin (Ttr), mRNA [NM_013697]	chr18:20828519-20828577	5.82	9.44	0.08
658	A_55_P1971398	XM_980161	Rpl17-ps2	PREDICTED: Mus musculus similar to Ribosomal protein L17 (LOC667688), mRNA [XM_980161]	chr12:96929241-96929300	6.03	9.70	0.08
659	A_55_P2111163	NM_009789	S100g	Mus musculus S100 calcium binding protein G (S100g), mRNA [NM_009789]	chrX:159399997-159399938	6.11	9.83	0.08
660	A_30_P01027977	chr2:33496712-33501183_F		lincRNA:chr2:33496712-33501183 forward strand	chr2:33501079-33501138	5.93	9.67	0.08
661	A_51_P378856	NM_019703	Pfkp	Mus musculus phosphofructokinase, platelet (Pfkp), mRNA [NM_019703]	chr13:6583872-6581718	8.70	12.45	0.07
662	A_55_P2046807	NM_009242	Sparc	Mus musculus secreted acidic cysteine rich glycoprotein (Sparc), mRNA [NM_009242]	chr11:55208061-55208003	7.33	11.10	0.07
663	A_55_P1960916	NM_028133	Egln3	Mus musculus EGL nine homolog 3 (C. elegans) (Egln3), mRNA [NM_028133]	chr12:55281311-55281252	6.19	9.98	0.07
664	A_30_P01028827	H19		lincRNA:chr7:149761435-149764019 reverse strand	chr7:149762172-149762113	8.69	12.51	0.07
665	A_51_P447545	NM_008341	Igfbp1	Mus musculus insulin-like growth factor binding protein 1 (Igfbp1), mRNA [NM_008341]	chr11:7102442-7102501	6.37	10.21	0.07

666	A_30_P01026417	chr2:33500594-33501874_F		lincRNA:chr2:33500594-33501874 forward strand	chr2:33501799-33501858	6.07	9.95	0.07
667	A_55_P2106690	NM_011527	Tal1	Mus musculus T-cell acute lymphocytic leukemia 1 (Tal1), mRNA [NM_011527]	chr4:114744219-114744278	7.31	11.24	0.07
668	A_51_P363947	NM_007669	Cdkn1a	Mus musculus cyclin-dependent kinase inhibitor 1A (P21) (Cdkn1a), transcript variant 1, mRNA [NM_007669]	chr17:29237377-29237436	7.49	11.49	0.06
669	A_30_P01026972	H19		lincRNA:chr7:149761435-149764019 reverse strand	chr7:149762232-149762173	8.22	12.26	0.06
670	A_55_P2035946	NM_001002927	Penk	Mus musculus preproenkephalin (Penk), mRNA [NM_001002927]	chr4:4060783-4060724	7.96	12.06	0.06
671	A_52_P418489	NM_023476	Tinagl1	Mus musculus tubulointerstitial nephritis antigen-like 1 (Tinagl1), transcript variant 1, mRNA [NM_023476]	chr4:129843103-129843044	6.03	10.15	0.06
672	A_30_P01028007	H19		lincRNA:chr7:149761435-149764019 reverse strand	chr7:149762016-149761957	8.36	12.49	0.06
673	A_51_P261569	NM_198674	Fbxw26	Mus musculus F-box and WD-40 domain protein 26 (Fbxw26), mRNA [NM_198674]	chr9:109624730-109624671	5.51	9.64	0.06
674	A_55_P2050173	XM_990857	LOC676534	PREDICTED: Mus musculus similar to ITGBL1 protein (LOC676534), mRNA [XM_990857]	chr4:120537534-120537593	6.82	10.97	0.06
675	A_55_P2046812	NM_009242	Sparc	Mus musculus secreted acidic cysteine rich glycoprotein (Sparc), mRNA [NM_009242]	chr11:55208998-55208939	9.12	13.29	0.06
676	A_55_P2074942	NM_010484	Slc6a4	Mus musculus solute carrier family 6 (neurotransmitter transporter, serotonin), member 4 (Slc6a4), mRNA [NM_010484]	chr11:76845777-76845836	5.80	9.97	0.06
677	A_51_P423743	NM_018777	Cldn6	Mus musculus claudin 6 (Cldn6), mRNA [NM_018777]	chr17:23819027-23819086	6.19	10.52	0.05
678	A_51_P123262	NM_029530	6330527O06Rik	Mus musculus RIKEN cDNA 6330527O06 gene (6330527O06Rik), mRNA [NM_029530]	chr2:135895179-135895238	7.49	11.83	0.05
679	A_55_P2118173	NM_010514	Igf2	Mus musculus insulin-like growth factor 2 (Igf2), transcript variant 1, mRNA [NM_010514]	chr7:149839720-149839661	5.95	10.73	0.04
680	A_52_P408025	NM_001143683	Mpped2	Mus musculus metallophosphoesterase domain containing 2 (Mpped2), transcript variant 1, mRNA [NM_001143683]	chr2:106707241-106707300	5.70	10.51	0.04
681	A_51_P250058	NM_010137	Epas1	Mus musculus endothelial PAS domain protein 1 (Epas1), mRNA [NM_010137]	chr17:87232615-87232674	6.53	11.61	0.03

682	A_66_P111011	NM_008091	Gata3	Mus musculus GATA binding protein 3 (Gata3), mRNA [NM_008091]	chr2:9779610-9779551	5.78	11.19	0.02

Table S8

MGE Lhx6-GFP^{+} vs ES Lhx6-GFP^{-}: Fold Change >= 4

No	Probe ID	Accession	Gene Symbol	Description	Genomic Coordinates	MGE Lhx6-GFP^{+}	ES Lhx6-GFP^{-}	Fold Change
1	A_52_P300376	NR_001570	Xist	Mus musculus inactive X specific transcripts (Xist), transcript variant 2, non-coding RNA [NR_001570]	chrX:100662233-100662174	14.38	6.40	251.77
2	A_55_P1967478	NM_008221	Hbb-y	Mus musculus hemoglobin Y, beta-like embryonic chain (Hbb-y), mRNA [NM_008221]	chr7:111000327-111000268	12.35	5.03	159.68
3	A_30_P01020783	XIST		lincRNA:chrX:100655711-100678572 reverse strand	chrX:100656081-100656022	12.44	6.25	72.81
4	A_52_P113537	NR_001463	Xist	Mus musculus inactive X specific transcripts (Xist), transcript variant 1, non-coding RNA [NR_001463]	chrX:100656348-100656289	11.56	5.48	67.56
5	A_55_P1967481	NM_008221	Hbb-y	Mus musculus hemoglobin Y, beta-like embryonic chain (Hbb-y), mRNA [NM_008221]	chr7:111000507-111000448	12.38	6.32	66.82
6	A_30_P01030803	XIST		lincRNA:chrX:100655711-100678572 reverse strand	chrX:100656141-100656082	11.46	5.72	53.40
7	A_51_P298615	NM_010713	Lhx8	Mus musculus LIM homeobox protein 8 (Lhx8), mRNA [NM_010713]	chr3:153969436-153969377	13.13	7.56	47.56
8	A_30_P01032945	XIST		lincRNA:chrX:100655711-100678572 reverse strand	chrX:100656018-100655959	12.55	7.01	46.27
9	A_30_P01022001	XIST		lincRNA:chrX:100655711-100678572 reverse strand	chrX:100655958-100655899	11.31	6.06	38.06
10	A_55_P2094149	NM_001083125	Lhx6	Mus musculus LIM homeobox protein 6 (Lhx6), transcript variant 2, mRNA [NM_001083125]	chr2:35958438-35958379	13.50	8.62	29.49
11	A_55_P2094158	NM_008500	Lhx6	Mus musculus LIM homeobox protein 6 (Lhx6), transcript variant 1, mRNA [NM_008500]	chr2:35937529-35937470	14.54	9.79	26.90
12	A_55_P2026420	NM_010127	Pou6f1	Mus musculus POU domain, class 6, transcription factor 1 (Pou6f1), mRNA [NM_010127]	chr15:100405950-100405891	12.00	7.28	26.18
13	A_52_P925277	NM_001079883	Bcl11b	Mus musculus B-cell leukemia/lymphoma 11B (Bcl11b), transcript variant 1, mRNA [NM_001079883]	chr12:109148991-109148932	12.64	8.09	23.46
14	A_55_P2328082	AK142074	AI606473	Mus musculus 12 days embryo eyeball cDNA, RIKEN full-length enriched library, clone:D230021C23 product:unclassifiable, full insert sequence. [AK142074]	chr3:153997592-153997651	11.67	7.13	23.33

15	A_30_P01023652	XIST		lincRNA:chrX:100655711-100678572 reverse strand	chrX:100655898-100655839	10.34	5.90	21.70
16	A_55_P1954693	NM_009311	Tac1	Mus musculus tachykinin 1 (Tac1), mRNA [NM_009311]	chr6:7512893-7512952	11.68	7.34	20.20
17	A_30_P01026500	chr4:90877414-90917198_F		lincRNA:chr4:90877414-90917198 forward strand	chr4:90916850-90916909	9.54	5.32	18.56
18	A_55_P1962299	NM_001083955	Hba-a2	Mus musculus hemoglobin alpha, adult chain 2 (Hba-a2), mRNA [NM_001083955]	chr11:32197245-32197304	10.41	6.21	18.47
19	A_51_P194230	NM_009573	Zic1	Mus musculus zinc finger protein of the cerebellum 1 (Zic1), mRNA [NM_009573]	chr9:91255484-91255425	13.74	9.76	15.82
20	A_55_P1967659	NM_030717	Lactb	Mus musculus lactamase, beta (Lactb), nuclear gene encoding mitochondrial protein, mRNA [NM_030717]	chr9:66803425-66803366	9.13	5.29	14.38
21	A_52_P455428	NM_183427	Glra2	Mus musculus glycine receptor, alpha 2 subunit (Glra2), mRNA [NM_183427]	chrX:161567556-161567497	9.89	6.05	14.28
22	A_55_P2053127	NM_001160112	Foxg1	Mus musculus forkhead box G1 (Foxg1), transcript variant 2, mRNA [NM_001160112]	chr12:50487766-50487825	14.87	11.08	13.77
23	A_55_P2071406	ENSMUST00000043259	Pde3a	phosphodiesterase 3A, cGMP inhibited Gene [Source:MGI Symbol;Acc:MGI:1860764] [ENSMUST00000043259]	chr6:141448762-141448821	10.52	6.74	13.75
24	A_55_P2006615	NM_015754	Rbbp9	Mus musculus retinoblastoma binding protein 9 (Rbbp9), mRNA [NM_015754]	chr2:144368242-144368183	10.53	6.90	12.34
25	A_55_P1962303	NM_008218	Hba-a1	Mus musculus hemoglobin alpha, adult chain 1 (Hba-a1), mRNA [NM_008218]	chr11:32183955-32184014	12.48	8.86	12.27
26	A_55_P1953273	NR_033506	Gm3893	Mus musculus predicted gene 3893 (Gm3893), non-coding RNA [NR_033506]	chrUn_random:553693-553634	11.71	8.16	11.66
27	A_51_P448178	NM_175519	Kctd8	Mus musculus potassium channel tetramerisation domain containing 8 (Kctd8), mRNA [NM_175519]	chr5:69501318-69501259	9.74	6.29	10.96
28	A_55_P2295856	AA711038	AI594671	vt54g10.r1 Barstead mouse irradiated colon MPLRB7 Mus musculus cDNA clone IMAGE:1166946 5'. [AA711038]	chr11:39849827-39849768	8.55	5.14	10.60
29	A_55_P2143025	NM_013657	Sema3c	Mus musculus sema domain, immunoglobulin domain (Ig), short basic domain, secreted, (semaphorin) 3C (Sema3c), mRNA [NM_013657]	chr5:17235981-17236040	11.65	8.28	10.36
30	A_52_P475356	NM_011445	Sox6	Mus musculus SRY-box containing gene 6 (Sox6), transcript variant 1, mRNA [NM_011445]	chr7:122615477-122615418	13.21	9.89	10.00

31	A_66_P119376	NM_177715	Kctd12	Mus musculus potassium channel tetramerisation domain containing 12 (Kctd12), mRNA [NM_177715]	chr14:103376045-103375986	11.11	7.80	9.94
32	A_55_P2009640	NR_033553	1700048O20Rik	Mus musculus RIKEN cDNA 1700048O20 gene (1700048O20Rik), non-coding RNA [NR_033553]	chr9:121855717-121855776	9.64	6.34	9.79
33	A_55_P2004442	NM_152229	Nr2e1	Mus musculus nuclear receptor subfamily 2, group E, member 1 (Nr2e1), mRNA [NM_152229]	chr10:42281885-42281826	9.65	6.38	9.70
34	A_55_P1986321	NM_007960	Etv1	Mus musculus ets variant gene 1 (Etv1), transcript variant 1, mRNA [NM_007960]	chr12:39594622-39594681	10.59	7.33	9.62
35	A_51_P145948	NM_144946	Neto1	Mus musculus neuropilin (NRP) and tolloid (TLL)-like 1 (Neto1), mRNA [NM_144946]	chr18:86670386-86670445	11.16	7.93	9.42
36	A_55_P1974845	NM_001009978	Pde1a	Mus musculus phosphodiesterase 1A, calmodulin-dependent (Pde1a), transcript variant 7, mRNA [NM_001009978]	chr2:79705260-79705201	10.69	7.52	9.01
37	A_30_P01031788	chr9:121845920-121858120_R		lincRNA:chr9:121845920-121858120 reverse strand	chr9:121848572-121848513	9.47	6.35	8.71
38	A_55_P2051787	NM_015739	Gbx1	Mus musculus gastrulation brain homeobox 1 (Gbx1), mRNA [NM_015739]	chr5:24010303-24010244	9.21	6.09	8.71
39	A_52_P95759	NM_172788	Sh3rf3	Mus musculus SH3 domain containing ring finger 3 (Sh3rf3), mRNA [NM_172788]	chr10:58601558-58601617	10.02	6.93	8.54
40	A_52_P502228	NM_009576	Zic4	Mus musculus zinc finger protein of the cerebellum 4 (Zic4), mRNA [NM_009576]	chr9:91283991-91284050	9.71	6.62	8.53
41	A_55_P2024888	NM_021281	Ctss	Mus musculus cathepsin S (Ctss), mRNA [NM_021281]	chr3:95360245-95360304	8.46	5.40	8.33
42	A_55_P2063505	XM_001479508	Gm4235	PREDICTED: Mus musculus hypothetical protein LOC100043109 (LOC100043109), mRNA [XM_001479508]	chr12:33682931-33682872	12.97	9.91	8.32
43	A_30_P01030971	chr9:121845920-121858120_F		lincRNA:chr9:121845920-121858120 forward strand	chr9:121855465-121855524	10.14	7.09	8.27
44	A_52_P468343	NM_001122683	Bdh1	Mus musculus 3-hydroxybutyrate dehydrogenase, type 1 (Bdh1), transcript variant 2, mRNA [NM_001122683]	chr16:31458195-31458254	10.86	7.82	8.27
45	A_51_P279100	NM_008969	Ptgs1	Mus musculus prostaglandin-endoperoxide synthase 1 (Ptgs1), mRNA [NM_008969]	chr2:36107644-36107703	8.85	5.83	8.13

46	A_55_P1988872	ENSMUST00000109514		B-cell CLL/lymphoma 11A (zinc finger protein) Gene [Source:MGI (curated);Acc:MGI:106190] [ENSMUST00000109514]	chr11:24068355-24068414	13.30	10.30	8.02
47	A_55_P2302973	NM_011419	Kdm5d	Mus musculus lysine (K)-specific demethylase 5D (Kdm5d), mRNA [NM_011419]	chrY:277162-277221	8.58	5.59	7.95
48	A_55_P2177351	NM_008066	Gabra2	Mus musculus gamma-aminobutyric acid (GABA) A receptor, subunit alpha 2 (Gabra2), mRNA [NM_008066]	chr5:71352365-71352306	10.13	7.14	7.94
49	A_52_P112463	NR_015388	Dlx6os1	Mus musculus Dlx6 opposite strand transcript 1 (Dlx6os1), non-coding RNA [NR_015388]	chr6:6771456-6771400	11.21	8.23	7.90
50	A_66_P126459	XM_001474797	Gm13429	PREDICTED: Mus musculus hypothetical protein LOC100040493 (LOC100040493), mRNA [XM_001474797]	chr2:36068338-36068397	9.75	6.77	7.88
51	A_55_P1978905	ENSMUST00000085379	Rpl26-ps2	PREDICTED: Mus musculus predicted gene, EG217600 (EG217600), mRNA [XM_138109]	chr12:58733766-58733707	10.77	7.80	7.87
52	A_52_P22763	NM_001039934	Mtap2	Mus musculus microtubule-associated protein 2 (Mtap2), transcript variant 1, mRNA [NM_001039934]	chr1:66488783-66488842	13.49	10.52	7.84
53	A_30_P01028487	chr9:121845920-121858120_R		lincRNA:chr9:121845920-121858120 reverse strand	chr9:121851061-121851002	9.37	6.42	7.75
54	A_52_P222230	XM_001476722		PREDICTED: Mus musculus hypothetical protein LOC100041156 (LOC100041156), mRNA [XM_001476722]	chr8:93412002-93412061	11.46	8.51	7.70
55	A_55_P1965694	NM_008073	Gabrg2	Mus musculus gamma-aminobutyric acid (GABA) A receptor, subunit gamma 2 (Gabrg2), transcript variant 1, mRNA [NM_008073]	chr11:41725423-41725364	11.94	9.00	7.66
56	A_55_P2057528	NM_025404	Arl4d	Mus musculus ADP-ribosylation factor-like 4D (Arl4d), mRNA [NM_025404]	chr11:101529032-101529091	10.45	7.53	7.56
57	A_51_P476960	NM_001081391	Csmd3	Mus musculus CUB and Sushi multiple domains 3 (Csmd3), mRNA [NM_001081391]	chr15:47781534-47781475	9.59	6.68	7.50
58	A_55_P2243828	AK035826	LOC552901	Mus musculus 16 days neonate cerebellum cDNA, RIKEN full-length enriched library, clone:9630008J17 product:unclassifiable, full insert sequence. [AK035826]	chr6:141250879-141250938	10.56	7.68	7.34
59	A_55_P2061119	XM_001003594	Gm8570	PREDICTED: Mus musculus predicted gene, EG667314 (EG667314), mRNA [XM_001003594]	chr10:92660479-92660420	14.65	11.77	7.32

60	A_55_P2020612	NM_025404	Arl4d	Mus musculus ADP-ribosylation factor-like 4D (Arl4d), mRNA [NM_025404]	chr11:101528989-101529048	11.12	8.26	7.28
61	A_55_P1973352	XM_001476722		PREDICTED: Mus musculus hypothetical protein LOC100041156 (LOC100041156), mRNA [XM_001476722]	chr8:93415492-93415551	9.68	6.83	7.20
62	A_52_P216427	NM_009385	Nkx2-1	Mus musculus NK2 homeobox 1 (Nkx2-1), transcript variant 1, mRNA [NM_009385]	chr12:57633178-57633119	12.94	10.09	7.20
63	A_55_P2096023	XM_001473759	Gm2490	PREDICTED: Mus musculus similar to 4933409K07Rik protein (LOC100039909), mRNA [XM_001473759]	chrUn_random:753332-753272	11.48	8.64	7.14
64	A_65_P01834	NM_001113545	Lima1	Mus musculus LIM domain and actin binding 1 (Lima1), transcript variant a, mRNA [NM_001113545]	chr15:99610370-99610311	10.47	7.63	7.13
65	A_55_P2120141	XM_001480410	Gm4522	PREDICTED: Mus musculus similar to reproductive homeobox on X chromosome 8 (LOC100043565), mRNA [XM_001480410]	chr6:126244172-126244231	13.95	11.12	7.11
66	A_51_P105068	NM_027990	Lypd6b	Mus musculus LY6/PLAUR domain containing 6B (Lypd6b), mRNA [NM_027990]	chr2:49804191-49804250	9.69	6.87	7.09
67	A_66_P116311	NM_008448	Kif5b	Mus musculus kinesin family member 5B (Kif5b), mRNA [NM_008448]	chr18:6202574-6202515	8.92	6.10	7.02
68	A_30_P01018292	chr2:36112819-36114110_F		lincRNA:chr2:36112819-36114110 forward strand	chr2:36113149-36113208	8.39	5.59	6.99
69	A_55_P2286493	AK016664	4933405E24Rik	Mus musculus adult male testis cDNA, RIKEN full-length enriched library, clone:4933405E24 product:unclassifiable, full insert sequence. [AK016664]	chr11:53991350-53991291	14.31	11.52	6.93
70	A_55_P1985070	NM_019413	Robo1	Mus musculus roundabout homolog 1 (Drosophila) (Robo1), mRNA [NM_019413]	chr16:73046190-73046249	11.15	8.36	6.92
71	A_52_P161630	NM_145933	St6gal1	Mus musculus beta galactoside alpha 2,6 sialyltransferase 1 (St6gal1), mRNA [NM_145933]	chr16:23360037-23360096	10.98	8.19	6.90
72	A_55_P2061164	XM_001471589	LOC100044317	PREDICTED: Mus musculus hypothetical protein LOC100044317 (LOC100044317), mRNA [XM_001471589]	chrX:133508445-133508386	9.79	7.02	6.79
73	A_55_P2130660	NM_178740	Slitrk4	Mus musculus SLIT and NTRK-like family, member 4 (Slitrk4), mRNA [NM_178740]	chrX:61523312-61523253	9.66	6.90	6.76
74	A_51_P480202	NM_010054	Dlx2	Mus musculus distal-less homeobox 2 (Dlx2), mRNA [NM_010054]	chr2:71381612-71381553	12.80	10.06	6.64

75	A_55_P2112584	NM_009322	Tbr1	Mus musculus T-box brain gene 1 (Tbr1), mRNA [NM_009322]	chr2:61652093-61652152	10.70	7.97	6.63
76	A_55_P1961152	NM_011141	Pou3f1	Mus musculus POU domain, class 3, transcription factor 1 (Pou3f1), mRNA [NM_011141]	chr4:124337692-124337751	10.94	8.22	6.61
77	A_52_P28745	NM_145399	Scgn	Mus musculus secretagogin, EF-hand calcium binding protein (Scgn), mRNA [NM_145399]	chr13:24054661-24054004	8.56	5.85	6.56
78	A_30_P01032159	chr12:50490638-50508093_F		lincRNA:chr12:50490638-50508093 forward strand	chr12:50508034-50508093	9.24	6.53	6.55
79	A_55_P2027900	XM_001478553	LOC100047443	PREDICTED: Mus musculus similar to Gamma-aminobutyric-acid receptor subunit alpha-2 precursor (GABA(A) receptor subunit alpha-2) (LOC100047443), mRNA [XM_001478553]	chr5:71352334-71352275	10.07	7.37	6.51
80	A_30_P01026237	chr9:121845920-121858120_F		lincRNA:chr9:121845920-121858120 forward strand	chr9:121851028-121851087	8.62	5.93	6.45
81	A_51_P229602	NM_008882	Plxna2	Mus musculus plexin A2 (Plxna2), mRNA [NM_008882]	chr1:196642958-196643017	13.60	10.91	6.43
82	A_52_P432969	NM_001105245	Pcdh19	Mus musculus protocadherin 19 (Pcdh19), transcript variant 1, mRNA [NM_001105245]	chrX:130122415-130122356	11.29	8.62	6.39
83	A_52_P843948	XM_914998	Gm9804	PREDICTED: Mus musculus predicted gene, ENSMUSG00000045946 (ENSMUSG00000045946), mRNA [XM_914998]	chr12:50504793-50504852	8.97	6.30	6.36
84	A_51_P507333	NM_001025581	Kcnc2	Mus musculus potassium voltage gated channel, Shaw-related subfamily, member 2 (Kcnc2), mRNA [NM_001025581]	chr10:111902778-111902837	9.19	6.54	6.30
85	A_55_P2023797	NM_029606	Ccdc46	Mus musculus coiled-coil domain containing 46 (Ccdc46), transcript variant 2, mRNA [NM_029606]	chr11:108721843-108721902	10.39	7.74	6.28
86	A_55_P2112642	NM_011648	Tshr	Mus musculus thyroid stimulating hormone receptor (Tshr), transcript variant 1, mRNA [NM_011648]	chr12:92778221-92778280	8.76	6.13	6.19
87	A_55_P2054967	ENSMUST00000053171		leucine rich repeat containing 49 Gene [Source:MGI (curated);Acc:MGI:2442689] [ENSMUST00000053171]	chr9:60417103-60417044	8.73	6.10	6.19
88	A_51_P218774	NM_026418	Rgs10	Mus musculus regulator of G-protein signalling 10 (Rgs10), mRNA [NM_026418]	chr7:135517315-135517256	12.48	9.86	6.17

89	A_55_P1958275	NM_016707	Bcl11a	Mus musculus B-cell CLL/lymphoma 11A (zinc finger protein) (Bcl11a), transcript variant 1, mRNA [NM_016707]	chr11:24073114-24073173	12.26	9.64	6.14
90	A_51_P228817	ENSMUST00000127420		staufen (RNA binding protein) homolog 2 (Drosophila) Gene [Source:MGI (curated);Acc:MGI:1352508] [ENSMUST00000127420]	chr1:16334661-16334602	10.62	8.01	6.08
91	A_55_P2223851	ENSMUST00000104938		Novel protein [Source:UniProtKB/TrEMBL;Acc:Q5SVU9] [ENSMUST00000104938]	chr11:90124966-90125025	8.99	6.39	6.05
92	A_51_P301603	XM_979851	AI854517	PREDICTED: Mus musculus expressed sequence AI854517 (AI854517), mRNA [XM_979851]	chr7:86677283-86677342	12.34	9.76	5.99
93	A_51_P163106	NM_175177	Bdh1	Mus musculus 3-hydroxybutyrate dehydrogenase, type 1 (Bdh1), nuclear gene encoding mitochondrial protein, transcript variant 1, mRNA [NM_175177]	chr16:31457378-31457437	10.15	7.57	5.98
94	A_52_P361435	NM_175513	Zfp804a	Mus musculus zinc finger protein 804A (Zfp804a), mRNA [NM_175513]	chr2:82099768-82099827	8.00	5.42	5.97
95	A_55_P2033480	NM_001085530	Gm13298	Mus musculus predicted gene 13298 (Gm13298), mRNA [NM_001085530]	chr4:41841838-41841897	10.13	7.56	5.94
96	A_55_P2157794	NM_172849	9430031J16Rik	Mus musculus RIKEN cDNA 9430031J16 gene (9430031J16Rik), mRNA [NM_172849]	chr1:81268148-81268207	8.93	6.36	5.93
97	A_55_P2009774	NM_009573	Zic1	Mus musculus zinc finger protein of the cerebellum 1 (Zic1), mRNA [NM_009573]	chr9:91257360-91257301	11.98	9.41	5.92
98	A_55_P2049373	NM_018779	Pde3a	Mus musculus phosphodiesterase 3A, cGMP inhibited (Pde3a), mRNA [NM_018779]	chr6:141447805-141447864	9.03	6.51	5.76
99	A_51_P261051	NM_010056	Dlx5	Mus musculus distal-less homeobox 5 (Dlx5), transcript variant 1, mRNA [NM_010056]	chr6:6828122-6828063	11.98	9.46	5.74
100	A_55_P2386095	AK036139	9630039A02Rik	Mus musculus 16 days neonate cerebellum cDNA, RIKEN full-length enriched library, clone:9630039A02 product:chromobox homolog 3 (Drosophila HP1 gamma), pseudogene3, full insert sequence. [AK036139]	chr6:45183697-45183756	8.86	6.35	5.71

101	A_55_P2177406	XM_001472931	Gm2813	PREDICTED: Mus musculus hypothetical protein LOC100044833 (LOC100044833), mRNA [XM_001472931]	chr5:92192410-92192351	8.37	5.86	5.69
102	A_52_P250555	NM_019682	Dynll1	Mus musculus dynein light chain LC8-type 1 (Dynll1), mRNA [NM_019682]	chr5:115747412-115747353	10.48	7.97	5.69
103	A_30_P01018678	chr9:121845920-121858120_F		lincRNA:chr9:121845920-121858120 forward strand	chr9:121855262-121855321	8.58	6.08	5.68
104	A_51_P171772	NM_021399	Bcl11b	Mus musculus B-cell leukemia/lymphoma 11B (Bcl11b), transcript variant 2, mRNA [NM_021399]	chr12:109153906-109153847	9.40	6.90	5.67
105	A_66_P118123	NM_177408	Gabrg2	Mus musculus gamma-aminobutyric acid (GABA) A receptor, subunit gamma 2 (Gabrg2), transcript variant 2, mRNA [NM_177408]	chr11:41723850-41723791	12.14	9.66	5.58
106	A_30_P01031087	chr3:4797557-4798821_F		lincRNA:chr3:4797557-4798821 forward strand	chr3:4797896-4797955	8.17	5.70	5.57
107	A_55_P1956918	NM_011782	Adamts5	Mus musculus a disintegrin-like and metallopeptidase (reprolysin type) with thrombospondin type 1 motif, 5 (aggrecanase-2) (Adamts5), mRNA [NM_011782]	chr16:85858461-85858402	8.69	6.22	5.53
108	A_55_P2007919	NM_001013785	Akr1c19	Mus musculus aldo-keto reductase family 1, member C19 (Akr1c19), mRNA [NM_001013785]	chr13:4247522-4247581	7.99	5.52	5.53
109	A_55_P2315762	AK045275		Mus musculus 9.5 days embryo parthenogenote cDNA, RIKEN full-length enriched library, clone:B130054P17 product:hypothetical protein, full insert sequence. [AK045275]	chr2:73113346-73113287	11.55	9.10	5.49
110	A_30_P01020660	chr11:53592946-53637922_F		lincRNA:chr11:53592946-53637922 forward strand	chr11:53630944-53631003	9.37	6.92	5.45
111	A_55_P2036999	NM_009575	Zic3	Mus musculus zinc finger protein of the cerebellum 3 (Zic3), mRNA [NM_009575]	chrX:55289616-55289675	10.83	8.39	5.44
112	A_52_P554703	NM_183183	Gprin3	Mus musculus GPRIN family member 3 (Gprin3), mRNA [NM_183183]	chr6:59302769-59302710	10.44	8.00	5.43
113	A_55_P2147941	NM_001025382	Fam196b	Mus musculus family with sequence similarity 196, member B (Fam196b), mRNA [NM_001025382]	chr11:34322535-34322594	9.95	7.51	5.43
114	A_55_P2057587	NM_007492	Arx	Mus musculus aristaless related homeobox (Arx), mRNA [NM_007492]	chrX:90543615-90543674	14.43	11.99	5.42

115	A_52_P114260	NM_001113356	C1rb	Mus musculus complement component 1, r subcomponent B (C1rb), mRNA [NM_001113356]	chr6:124525309-124525368	10.21	7.78	5.41
116	A_51_P179578	NM_010262	Gbx2	Mus musculus gastrulation brain homeobox 2 (Gbx2), mRNA [NM_010262]	chr1:91825072-91825013	10.70	8.27	5.40
117	A_55_P1987430	NM_001143802	Fam196a	Mus musculus family with sequence similarity 196, member A (Fam196a), mRNA [NM_001143802]	chr7:142073757-142073698	9.36	6.93	5.39
118	A_55_P2077483	NM_052977	Adarb2	Mus musculus adenosine deaminase, RNA-specific, B2 (Adarb2), mRNA [NM_052977]	chr13:8759495-8759554	11.41	8.98	5.38
119	A_66_P120555	NM_001081097	Grik3	Mus musculus glutamate receptor, ionotropic, kainate 3 (Grik3), mRNA [NM_001081097]	chr4:125391317-125391376	11.45	9.03	5.36
120	A_30_P01023611	chr9:121845920-121858120_R		lincRNA:chr9:121845920-121858120 reverse strand	chr9:121855357-121855298	8.92	6.50	5.36
121	A_52_P399990	NM_017377	B4galt2	Mus musculus UDP-Gal:betaGlcNAc beta 1,4-galactosyltransferase, polypeptide 2 (B4galt2), mRNA [NM_017377]	chr4:117546012-117545953	10.66	8.25	5.34
122	A_55_P2199737	AK020245	9030205G03Rik	Mus musculus adult male colon cDNA, RIKEN full-length enriched library, clone:9030205G03 product:unclassifiable, full insert sequence. [AK020245]	chr8:14507586-14507645	9.04	6.63	5.32
123	A_30_P01026072	chr17:36131460-36136694_F		lincRNA:chr17:36131460-36136694 forward strand	chr17:36135828-36135887	10.27	7.87	5.30
124	A_55_P1985559	ENSMUST00000119783		GATA zinc finger domain containing 1 Gene [Source:MGI (curated);Acc:MGI:1914460] [ENSMUST00000119783]	chr5:3643283-3643224	11.05	8.65	5.29
125	A_55_P2033010	NM_026422	Mrrf	Mus musculus mitochondrial ribosome recycling factor (Mrrf), nuclear gene encoding mitochondrial protein, mRNA [NM_026422]	chr2:36045366-36045425	11.25	8.87	5.22
126	A_52_P108243	NM_011054	Pde1c	Mus musculus phosphodiesterase 1C (Pde1c), transcript variant 1, mRNA [NM_011054]	chr6:56030296-56030237	8.08	5.70	5.22
127	A_55_P2392485	NM_001162537	9330159F19Rik	Mus musculus RIKEN cDNA 9330159F19 gene (9330159F19Rik), mRNA [NM_001162537]	chr10:28948163-28948222	11.50	9.12	5.21
128	A_55_P2104924	NM_172420	Ppp1r1c	Mus musculus protein phosphatase 1, regulatory (inhibitor) subunit 1C (Ppp1r1c), mRNA [NM_172420]	chr2:79548348-79548407	8.82	6.45	5.20

129	A_51_P417321	NM_177832	Zfp236	Mus musculus zinc finger protein 236 (Zfp236), mRNA [NM_177832]	chr18:82765067-82765008	9.94	7.57	5.20
130	A_51_P432950	NM_009216	Sstr1	Mus musculus somatostatin receptor 1 (Sstr1), mRNA [NM_009216]	chr12:59314287-59314346	8.94	6.56	5.20
131	A_55_P2038540	NM_016956	Hbb-b2	Mus musculus hemoglobin, beta adult minor chain (Hbb-b2), mRNA [NM_016956]		7.91	5.55	5.13
132	A_30_P01021129	chr5:22890322-22939119_R		lincRNA:chr5:22890322-22939119 reverse strand	chr5:22890502-22890443	10.11	7.75	5.12
133	A_55_P2181753	NM_001122683	Bdh1	Mus musculus 3-hydroxybutyrate dehydrogenase, type 1 (Bdh1), transcript variant 2, mRNA [NM_001122683]	chr16:31457249-31457308	8.64	6.29	5.10
134	A_52_P220810	NM_144551	Trib2	Mus musculus tribbles homolog 2 (Drosophila) (Trib2), mRNA [NM_144551]	chr12:15799068-15799009	12.33	9.99	5.05
135	A_55_P2026734	NM_009062	Rgs4	Mus musculus regulator of G-protein signaling 4 (Rgs4), mRNA [NM_009062]	chr1:171671668-171671609	10.21	7.88	5.05
136	A_30_P01027901	chr12:50490638-50508093_F		lincRNA:chr12:50490638-50508093 forward strand	chr12:50507936-50507995	9.44	7.11	5.05
137	A_55_P2261772	AK082735	C230098O21Rik	Mus musculus 0 day neonate cerebellum cDNA, RIKEN full-length enriched library, clone:C230098O21 product:unclassifiable, full insert sequence. [AK082735]	chr8:71656726-71656667	10.64	8.31	5.03
138	A_51_P183283	NM_008032	Aff2	Mus musculus AF4/FMR2 family, member 2 (Aff2), mRNA [NM_008032]	chrX:67121116-67121175	11.66	9.34	5.02
139	A_55_P1965939	ENSMUST00000113343		RIKEN cDNA 3110007F17 gene Gene [Source:MGI Symbol;Acc:MGI:1920311] [ENSMUST00000113343]	chrX:120257012-120257071	8.84	6.51	5.02
140	A_30_P01017513	chr9:121845920-121858120_R		lincRNA:chr9:121845920-121858120 reverse strand	chr9:121855293-121855234	8.26	5.93	5.02
141	A_66_P139946	XM_976924	E030019B13Rik	PREDICTED: Mus musculus RIKEN cDNA E030019B13 gene (E030019B13Rik), mRNA [XM_976924]	chr12:57591678-57591619	11.56	9.23	5.01
142	A_30_P01033327	chr13:112337405-112356455_F		lincRNA:chr13:112337405-112356455 forward strand	chr13:112351036-112351095	8.09	5.77	5.00
143	A_55_P1959576	NM_178740	Slitrk4	Mus musculus SLIT and NTRK-like family, member 4 (Slitrk4), mRNA [NM_178740]	chrX:61522807-61522748	8.70	6.38	5.00
144	A_55_P2034968	ENSMUST00000036223		SH3 domain containing ring finger 3 Gene [Source:MGI (curated);Acc:MGI:2444637] [ENSMUST00000036223]	chr10:58567568-58567627	9.35	7.03	4.99

145	A_66_P130601	NM_053242	Foxp2	Mus musculus forkhead box P2 (Foxp2), transcript variant 1, mRNA [NM_053242]	chr6:15391821-15391880	9.04	6.72	4.99
146	A_55_P2150496	NM_010063	Dync1i1	Mus musculus dynein cytoplasmic 1 intermediate chain 1 (Dync1i1), mRNA [NM_010063]	chr6:5977977-5978036	11.58	9.27	4.98
147	A_55_P2158227	NM_009640	Angpt1	Mus musculus angiopoietin 1 (Angpt1), mRNA [NM_009640]	chr15:42256394-42256335	8.50	6.19	4.97
148	A_55_P2152637	NM_009576	Zic4	Mus musculus zinc finger protein of the cerebellum 4 (Zic4), mRNA [NM_009576]	chr9:91282924-91282983	8.52	6.21	4.96
149	A_55_P2170605	ENSMUST00000068297		RIKEN cDNA 9430031J16 gene Gene [Source:MGI Symbol;Acc:MGI:2443135] [ENSMUST00000068297]	chr1:81333257-81333316	8.37	6.06	4.95
150	A_52_P244132	NM_025478	Isoc1	Mus musculus isochorismatase domain containing 1 (Isoc1), mRNA [NM_025478]	chr18:58838445-58838504	11.56	9.25	4.94
151	A_52_P39157	NM_007552	Bmi1	Mus musculus Bmi1 polycomb ring finger oncogene (Bmi1), mRNA [NM_007552]	chr2:18607987-18608046	11.21	8.93	4.87
152	A_55_P1959541	XR_034879	Gm14026	PREDICTED: Mus musculus similar to SET domain containing 3 (LOC545461), misc RNA [XR_034879]	chr2:128996852-128996911	11.61	9.33	4.87
153	A_55_P2014100	NM_001177666	Gm7120	Mus musculus predicted gene 7120 (Gm7120), transcript variant 2, mRNA [NM_001177666]	chr13:120277695-120277754	9.10	6.82	4.87
154	A_51_P204053	NM_010405	Hba-x	Mus musculus hemoglobin X, alpha-like embryonic chain in Hba complex (Hba-x), mRNA [NM_010405]	chr11:32177668-32177727	7.98	5.70	4.87
155	A_52_P282838	NM_001005370	Spin2	Mus musculus spindlin family, member 2 (Spin2), mRNA [NM_001005370]	chrX:150268691-150268750	9.82	7.54	4.86
156	A_55_P1999945	NM_172910	Dlgap2	Mus musculus discs, large (Drosophila) homolog-associated protein 2 (Dlgap2), transcript variant 1, mRNA [NM_172910]	chr8:14847595-14847654	9.91	7.63	4.85
157	A_55_P1982325	NM_022565	Ndst4	Mus musculus N-deacetylase/N-sulfotransferase (heparin glucosaminyl) 4 (Ndst4), mRNA [NM_022565]	chr3:125427787-125427846	8.63	6.35	4.84
158	A_30_P01027192	chr1:87343550-87362900_F		lincRNA:chr1:87343550-87362900 forward strand	chr1:87354285-87354344	9.06	6.78	4.84
159	A_52_P160005	NM_211138	Pcyt1b	Mus musculus phosphate cytidylyltransferase 1, choline, beta isoform (Pcyt1b), transcript variant 1, mRNA [NM_211138]	chrX:90994629-90994688	10.68	8.41	4.84

160	A_55_P2090429	NM_010063	Dync1i1	Mus musculus dynein cytoplasmic 1 intermediate chain 1 (Dync1i1), mRNA [NM_010063]	chr6:5977935-5977994	12.46	10.19	4.82
161	A_51_P402994	NM_012008	Ddx3y	Mus musculus DEAD (Asp-Glu-Ala-Asp) box polypeptide 3, Y-linked (Ddx3y), mRNA [NM_012008]	chrY:598274-598215	8.11	5.86	4.76
162	A_30_P01032193	chr3:4797557-4798821_F		lincRNA:chr3:4797557-4798821 forward strand	chr3:4798762-4798821	8.52	6.27	4.74
163	A_30_P01022360	chr3:126969590-126972626_R		lincRNA:chr3:126969590-126972626 reverse strand	chr3:126969650-126969591	8.51	6.27	4.73
164	A_55_P1961320	NM_207176	Tes	Mus musculus testis derived transcript (Tes), mRNA [NM_207176]	chr6:17055712-17055771	10.94	8.70	4.71
165	A_30_P01027542	chr3:88009045-88036270_F		lincRNA:chr3:88009045-88036270 forward strand	chr3:88033314-88033373	11.69	9.46	4.70
166	A_52_P6057	NM_018763	Chst2	Mus musculus carbohydrate sulfotransferase 2 (Chst2), mRNA [NM_018763]	chr9:95301994-95301935	9.84	7.61	4.69
167	A_30_P01031223	chr6:126766225-126771309_R		lincRNA:chr6:126766225-126771309 reverse strand	chr6:126769602-126769543	9.03	6.80	4.67
168	A_30_P01017678	chr9:121845920-121858120_R		lincRNA:chr9:121845920-121858120 reverse strand	chr9:121850954-121850895	8.29	6.06	4.67
169	A_55_P2068247	XM_001473755	Gm2488	PREDICTED: Mus musculus similar to Ubtf protein (LOC100039907), mRNA [XM_001473755]	chr15:13245897-13245838	12.08	9.86	4.67
170	A_52_P464831	NM_010053	Dlx1	Mus musculus distal-less homeobox 1 (Dlx1), mRNA [NM_010053]	chr2:71371499-71371558	14.36	12.14	4.67
171	A_52_P106789	NM_026434	Rbm18	Mus musculus RNA binding motif protein 18 (Rbm18), transcript variant 1, mRNA [NM_026434]	chr2:35971664-35971605	15.14	12.92	4.63
172	A_55_P1990200	NM_001008423	Gm1568	Mus musculus predicted gene 1568 (Gm1568), mRNA [NM_001008423]	chr12:81856850-81856791	12.65	10.44	4.62
173	A_51_P423709	NM_029007	Fam84a	Mus musculus family with sequence similarity 84, member A (Fam84a), mRNA [NM_029007]	chr12:14154930-14154871	11.39	9.19	4.61
174	A_55_P2077914	NM_001004361	5730494M16Rik	Mus musculus RIKEN cDNA 5730494M16 gene (5730494M16Rik), transcript variant 1, mRNA [NM_001004361]	chr18:25294605-25294546	9.47	7.27	4.58
175	A_55_P2090330	NM_021452	Kcnmb4	Mus musculus potassium large conductance calcium-activated channel, subfamily M, beta member 4 (Kcnmb4), mRNA [NM_021452]	chr10:115855036-115854977	10.22	8.03	4.58
176	A_30_P01029305	chr5:22887983-22939658_R		lincRNA:chr5:22887983-22939658 reverse strand	chr5:22890099-22890040	10.12	7.93	4.55

177	A_55_P2155620	NM_025282	Mef2c	Mus musculus myocyte enhancer factor 2C (Mef2c), transcript variant 2, mRNA [NM_025282]	chr13:83772788-83775025	9.27	7.09	4.55
178	A_52_P285024	NM_198247	Sertad4	Mus musculus SERTA domain containing 4 (Sertad4), transcript variant 1, mRNA [NM_198247]	chr1:194671386-194671327	10.48	8.30	4.53
179	A_55_P1973339	NM_001038663	Mapk1	Mus musculus mitogen-activated protein kinase 1 (Mapk1), transcript variant 2, mRNA [NM_001038663]	chr16:17039038-17039097	10.59	8.41	4.53
180	A_52_P612537	NM_178750	Ss18l1	Mus musculus synovial sarcoma translocation gene on chromosome 18-like 1 (Ss18l1), mRNA [NM_178750]	chr2:179804414-179804473	11.65	9.47	4.53
181	A_55_P2058137	NM_001039376	Pde4dip	Mus musculus phosphodiesterase 4D interacting protein (myomegalin) (Pde4dip), transcript variant 1, mRNA [NM_001039376]	chr3:97493807-97493748	10.27	8.09	4.53
182	A_55_P2113758	NM_001113325	Gria1	Mus musculus glutamate receptor, ionotropic, AMPA1 (alpha 1) (Gria1), transcript variant 1, mRNA [NM_001113325]	chr11:57143613-57143672	9.77	7.59	4.53
183	A_55_P2176076	NM_001081358	Lrrc7	Mus musculus leucine rich repeat containing 7 (Lrrc7), mRNA [NM_001081358]	chr3:157819812-157819753	10.42	8.24	4.52
184	A_55_P2205650	AK162965	2610507I01Rik	Mus musculus adult male spinal cord cDNA, RIKEN full-length enriched library, clone:A330105A21 product:unclassifiable, full insert sequence. [AK162965]	chr11:59011363-59011304	9.16	6.98	4.52
185	A_52_P244637	NM_001080381	Fam65b	Mus musculus family with sequence similarity 65, member B (Fam65b), transcript variant 2, mRNA [NM_001080381]	chr13:24797220-24797279	9.82	7.65	4.50
186	A_30_P01026422	chr1:162965309-162970704_F		lincRNA:chr1:162965309-162970704 forward strand	chr1:162967656-162967715	10.80	8.63	4.49
187	A_52_P481423	NM_030249	Cttnbp2nl	Mus musculus CTTNBP2 N-terminal like (Cttnbp2nl), transcript variant 1, mRNA [NM_030249]	chr3:104805139-104805080	11.41	9.24	4.48
188	A_51_P267494	NM_026514	Cdc42ep3	Mus musculus CDC42 effector protein (Rho GTPase binding) 3 (Cdc42ep3), mRNA [NM_026514]	chr17:79733468-79733409	10.98	8.82	4.48
189	A_51_P291078	NM_172710	Sel1l3	Mus musculus sel-1 suppressor of lin-12-like 3 (C. elegans) (Sel1l3), mRNA [NM_172710]	chr5:53498602-53498543	11.37	9.21	4.46

190	A_51_P223724	NM_133235	Khdrbs2	Mus musculus KH domain containing, RNA binding, signal transduction associated 2 (Khdrbs2), mRNA [NM_133235]	chr1:32714406-32714465	10.24	8.09	4.44
191	A_66_P114461	NM_177328	Grm7	Mus musculus glutamate receptor, metabotropic 7 (Grm7), mRNA [NM_177328]	chr6:111517083-111517142	10.46	8.31	4.44
192	A_52_P450934	NM_198414	Paqr9	Mus musculus progestin and adipoQ receptor family member IX (Paqr9), mRNA [NM_198414]	chr9:95462405-95462464	9.71	7.56	4.44
193	A_66_P137501	NM_001085521	Tmem90b	Mus musculus transmembrane protein 90B (Tmem90b), mRNA [NM_001085521]	chr2:149725275-149725334	9.19	7.05	4.41
194	A_51_P315042	NM_016847	Avpr1a	Mus musculus arginine vasopressin receptor 1A (Avpr1a), mRNA [NM_016847]	chr10:121890135-121890194	8.56	6.42	4.41
195	A_55_P2082005	NM_001165999	Lingo2	Mus musculus leucine rich repeat and Ig domain containing 2 (Lingo2), transcript variant 1, mRNA [NM_001165999]	chr4:35654714-35654655	9.03	6.90	4.40
196	A_30_P01025339	chr5:22890322-22939119_R		lincRNA:chr5:22890322-22939119 reverse strand	chr5:22890411-22890352	9.93	7.80	4.40
197	A_51_P338998	NM_008922	Prim2	Mus musculus DNA primase, p58 subunit (Prim2), mRNA [NM_008922]	chr1:33510792-33510733	8.85	6.72	4.39
198	A_51_P126437	NM_007930	Enc1	Mus musculus ectodermal-neural cortex 1 (Enc1), mRNA [NM_007930]	chr13:98022739-98022798	12.62	10.49	4.39
199	A_51_P357207	NM_001081144	Zfp518b	Mus musculus zinc finger protein 518B (Zfp518b), transcript variant 1, mRNA [NM_001081144]	chr5:39060195-39060136	10.65	8.52	4.37
200	A_66_P136097	NM_001164220	Trim13	Mus musculus tripartite motif-containing 13 (Trim13), transcript variant 1, mRNA [NM_001164220]	chr14:62224482-62224541	10.53	8.41	4.36
201	A_55_P2071384	NM_205769	Crh	Mus musculus corticotropin releasing hormone (Crh), mRNA [NM_205769]	chr3:19593541-19593482	8.30	6.18	4.36
202	A_30_P01029525	chr6:126766225-126771309_R		lincRNA:chr6:126766225-126771309 reverse strand	chr6:126769662-126769603	8.16	6.04	4.35
203	A_55_P1987176	NM_029536	Gpr165	Mus musculus G protein-coupled receptor 165 (Gpr165), mRNA [NM_029536]	chrX:93914626-93914685	9.45	7.33	4.35
204	A_55_P1979873	NM_028806	Phactr3	Mus musculus phosphatase and actin regulator 3 (Phactr3), transcript variant 1, mRNA [NM_028806]	chr2:178070714-178070773	9.21	7.09	4.35
205	A_52_P502267	NM_172663	Epc2	Mus musculus enhancer of polycomb homolog 2 (Drosophila) (Epc2), mRNA [NM_172663]	chr2:49405815-49405874	11.19	9.07	4.34

206	A_30_P01022232	chr13:29106280-29120376_F		lincRNA:chr13:29106280-29120376 forward strand	chr13:29120316-29120375	8.65	6.53	4.34
207	A_55_P1953753	NM_007726	Cnr1	Mus musculus cannabinoid receptor 1 (brain) (Cnr1), mRNA [NM_007726]	chr4:34035526-34035585	11.31	9.19	4.34
208	A_55_P2083919	NM_175549	Robo2	Mus musculus roundabout homolog 2 (Drosophila) (Robo2), mRNA [NM_175549]	chr16:73894712-73894653	10.98	8.87	4.33
209	A_55_P1991160	NM_026422	Mrrf	Mus musculus mitochondrial ribosome recycling factor (Mrrf), nuclear gene encoding mitochondrial protein, mRNA [NM_026422]	chr2:36045136-36045195	10.12	8.01	4.32
210	A_51_P461031	NM_172416	Ostm1	Mus musculus osteopetrosis associated transmembrane protein 1 (Ostm1), mRNA [NM_172416]	chr10:42422061-42422120	9.94	7.83	4.32
211	A_30_P01027135	chr5:22887983-22939658_R		lincRNA:chr5:22887983-22939658 reverse strand	chr5:22890128-22890069	9.90	7.79	4.31
212	A_55_P2095488	NM_019666	Syncrip	Mus musculus synaptotagmin binding, cytoplasmic RNA interacting protein (Syncrip), transcript variant 1, mRNA [NM_019666]	chr9:88350519-88350460	11.30	9.19	4.31
213	A_52_P38011	NM_178631	Ralyl	Mus musculus RALY RNA binding protein-like (Ralyl), transcript variant 1, mRNA [NM_178631]	chr3:14181384-14181443	10.00	7.90	4.30
214	A_51_P140429	NM_026918	Zg16	Mus musculus zymogen granule protein 16 (Zg16), mRNA [NM_026918]	chr7:134193896-134193837	9.42	7.31	4.30
215	A_55_P2059010	NM_011254	Rbp1	Mus musculus retinol binding protein 1, cellular (Rbp1), mRNA [NM_011254]	chr9:98346908-98346967	12.68	10.58	4.29
216	A_52_P495869	NM_010658	Mafb	Mus musculus v-maf musculoaponeurotic fibrosarcoma oncogene family, protein B (avian) (Mafb), mRNA [NM_010658]	chr2:160189605-160189546	11.78	9.68	4.29
217	A_55_P2102260	NM_008217	Has3	Mus musculus hyaluronan synthase 3 (Has3), mRNA [NM_008217]	chr8:109405354-109405413	9.87	7.77	4.28
218	A_30_P01028374	chr5:22890322-22939119_R		lincRNA:chr5:22890322-22939119 reverse strand	chr5:22890382-22890323	9.95	7.86	4.28
219	A_51_P358894	NM_028417	Ttc9b	Mus musculus tetratricopeptide repeat domain 9B (Ttc9b), mRNA [NM_028417]	chr7:28440933-28440992	10.12	8.02	4.27
220	A_51_P191726	NM_029946	Efcab6	Mus musculus EF-hand calcium binding domain 6 (Efcab6), transcript variant 1, mRNA [NM_029946]	chr15:83697552-83697493	7.97	5.88	4.27
221	A_66_P100777	NM_009216	Sstr1	Mus musculus somatostatin receptor 1 (Sstr1), mRNA [NM_009216]	chr12:59316670-59316729	9.49	7.40	4.27
222	A_65_P12152	NM_053268	Rasa2	Mus musculus RAS p21 protein activator 2 (Rasa2), mRNA [NM_053268]	chr9:96440084-96440025	9.85	7.76	4.26

223	A_51_P513661	NM_021530	Slc4a8	Mus musculus solute carrier family 4 (anion exchanger), member 8 (Slc4a8), mRNA [NM_021530]	chr15:100654207-100654266	12.26	10.17	4.26
224	A_55_P1985410	NM_178256	Reps2	Mus musculus RALBP1 associated Eps domain containing protein 2 (Reps2), mRNA [NM_178256]	chrX:158849967-158849908	8.97	6.89	4.24
225	A_51_P236738	NR_002839	Dlx6as	Mus musculus distal-less homeobox 6, antisense (Dlx6as), antisense RNA [NR_002839]	chr6:6814542-6814483	12.12	10.04	4.24
226	A_52_P352533	NM_172851	Cntnap5b	Mus musculus contactin associated protein-like 5B (Cntnap5b), mRNA [NM_172851]	chr1:102378552-102381139	8.27	6.18	4.24
227	A_55_P2052006	NM_199024	Nol4	Mus musculus nucleolar protein 4 (Nol4), transcript variant 2, mRNA [NM_199024]	chr18:22851979-22851920	12.43	10.35	4.23
228	A_51_P393897	NM_025837	Mpi	Mus musculus mannose phosphate isomerase (Mpi), mRNA [NM_025837]	chr9:57392444-57392385	10.08	8.00	4.20
229	A_51_P405167	NM_001025577	Maf	Mus musculus avian musculoaponeurotic fibrosarcoma (v-maf) AS42 oncogene homolog (Maf), mRNA [NM_001025577]	chr8:118227795-118227736	10.29	8.22	4.19
230	A_51_P265576	NM_026434	Rbm18	Mus musculus RNA binding motif protein 18 (Rbm18), transcript variant 1, mRNA [NM_026434]	chr2:35978415-35976403	14.06	11.99	4.19
231	A_55_P2148574	XM_001478611	LOC100047593	PREDICTED: Mus musculus hypothetical protein LOC100047593 (LOC100047593), mRNA [XM_001478611]	chr4:90878319-90878260	10.35	8.29	4.18
232	A_55_P1963980	NM_026825	Lrrc16a	Mus musculus leucine rich repeat containing 16A (Lrrc16a), mRNA [NM_026825]	chr13:24198238-24198179	10.83	8.77	4.17
233	A_52_P27122	NM_153177	Eif2c4	Mus musculus eukaryotic translation initiation factor 2C, 4 (Eif2c4), mRNA [NM_153177]	chr4:126167221-126167162	9.84	7.78	4.16
234	A_52_P395280	NM_001033346	Lrrc55	Mus musculus leucine rich repeat containing 55 (Lrrc55), mRNA [NM_001033346]	chr2:85028674-85028615	8.39	6.33	4.16
235	A_52_P374897	NM_009705	Arg2	Mus musculus arginase type II (Arg2), mRNA [NM_009705]	chr12:80252972-80253031	8.63	6.58	4.16
236	A_66_P120770	NM_011740	Ywhaz	Mus musculus tyrosine 3-monooxygenase/tryptophan 5-monooxygenase activation protein, zeta polypeptide (Ywhaz), mRNA [NM_011740]	chr15:36701264-36701205	14.20	12.15	4.16
237	A_55_P2119957	NM_021455	Mlxipl	Mus musculus MLX interacting protein-like (Mlxipl), mRNA [NM_021455]	chr5:135613705-135613764	13.73	11.67	4.15

238	A_55_P2112524	NM_028231	Kcnmb2	Mus musculus potassium large conductance calcium-activated channel, subfamily M, beta member 2 (Kcnmb2), mRNA [NM_028231]	chr3:32098621-32098680	8.40	6.34	4.15
239	A_52_P209944	ENSMUST00000052354		RIKEN cDNA C130071C03 gene Gene [Source:MGI Symbol;Acc:MGI:2443574] [ENSMUST00000052354]	chr13:83880591-83880650	12.49	10.44	4.14
240	A_66_P118170	NM_010252	Gabrg1	Mus musculus gamma-aminobutyric acid (GABA) A receptor, subunit gamma 1 (Gabrg1), mRNA [NM_010252]	chr5:71169383-71169324	8.40	6.35	4.14
241	A_55_P2113071	NM_153177	Eif2c4	Mus musculus eukaryotic translation initiation factor 2C, 4 (Eif2c4), mRNA [NM_153177]	chr4:126169596-126169537	9.71	7.67	4.13
242	A_51_P394847	NR_024599	Gm11346	Mus musculus predicted gene 11346 (Gm11346), non-coding RNA [NR_024599]	chr13:24690345-24690286	8.20	6.15	4.13
243	A_55_P1984178	NM_001039376	Pde4dip	Mus musculus phosphodiesterase 4D interacting protein (myomegalin) (Pde4dip), transcript variant 1, mRNA [NM_001039376]	chr3:97493815-97493756	10.10	8.06	4.13
244	A_55_P2058791	NM_026127	4833420G17Rik	Mus musculus RIKEN cDNA 4833420G17 gene (4833420G17Rik), transcript variant 1, mRNA [NM_026127]	chr13:120255051-120255110	12.45	10.40	4.12
245	A_55_P2007964	NM_009987	Cx3cr1	Mus musculus chemokine (C-X3-C) receptor 1 (Cx3cr1), mRNA [NM_009987]	chr9:119957870-119957811	7.84	5.80	4.12
246	A_55_P2081178	NM_001163833	Msl3l2	Mus musculus male-specific lethal 3-like 2 (Drosophila) (Msl3l2), mRNA [NM_001163833]	chr10:55836569-55836628	9.37	7.33	4.11
247	A_52_P164286	NM_001038619	Dnm3	Mus musculus dynamin 3 (Dnm3), transcript variant 1, mRNA [NM_001038619]	chr1:163920514-163920455	9.65	7.61	4.10
248	A_30_P01021622	chr6:54591416-54597566_R		lincRNA:chr6:54591416-54597566 reverse strand	chr6:54594142-54594083	11.00	8.98	4.08
249	A_52_P612137	NM_009822	Runx1t1	Mus musculus runt-related transcription factor 1; translocated to, 1 (cyclin D-related) (Runx1t1), transcript variant 3, mRNA [NM_009822]	chr4:13818189-13818248	14.38	12.35	4.07
250	A_55_P1962419	NM_026201	Ccar1	Mus musculus cell division cycle and apoptosis regulator 1 (Ccar1), mRNA [NM_026201]	chr10:62207274-62207215	9.46	7.44	4.07
251	A_55_P2036007	NM_198409	Rai2	Mus musculus retinoic acid induced 2 (Rai2), transcript variant 1, mRNA [NM_198409]	chrX:158217347-158217406	12.60	10.58	4.07

252	A_55_P2053374	NM_178362	Sorbs1	Mus musculus sorbin and SH3 domain containing 1 (Sorbs1), transcript variant 2, mRNA [NM_178362]	chr19:40366681-40366622	11.92	9.90	4.06
253	A_51_P303868	NM_001164493	Klhl29	Mus musculus kelch-like 29 (Drosophila) (Klhl29), mRNA [NM_001164493]	chr12:5084431-5084372	9.88	7.86	4.06
254	A_55_P2027668	ENSMUST00000058829	Neto1	neuropilin (NRP) and tolloid (TLL)-like 1 Gene [Source:MGI Symbol;Acc:MGI:2180216] [ENSMUST00000058829]	chr18:86672089-86672148	10.28	8.26	4.06
255	A_30_P01027247	chr9:121845920-121858120_F		lincRNA:chr9:121845920-121858120 forward strand	chr9:121855322-121855381	8.76	6.74	4.05
256	A_51_P295237	NM_172784	Lrp11	Mus musculus low density lipoprotein receptor-related protein 11 (Lrp11), mRNA [NM_172784]	chr10:7345019-7345078	12.31	10.30	4.05
257	A_55_P2038101	NM_001009947	Dock11	Mus musculus dedicator of cytokinesis 11 (Dock11), mRNA [NM_001009947]	chrX:33616163-33616222	10.11	8.10	4.04
258	A_55_P2016114	NM_010177	Fasl	Mus musculus Fas ligand (TNF superfamily, member 6) (Fasl), mRNA [NM_010177]	chr1:163711115-163711056	8.95	6.94	4.04
259	A_30_P01026923	chr3:121627545-121639470_R		lincRNA:chr3:121627545-121639470 reverse strand	chr3:121639036-121638977	8.29	6.28	4.04
260	A_55_P2148253	NR_029457	G530011O06Rik	Mus musculus RIKEN cDNA G530011O06 gene (G530011O06Rik), non-coding RNA [NR_029457]	chrX:166413035-166412976	8.37	6.36	4.04
261	A_30_P01025517	chr1:45066850-45095650_R		lincRNA:chr1:45066850-45095650 reverse strand	chr1:45091740-45091681	10.39	8.37	4.03
262	A_52_P589568	NM_194060	Foxo6	Mus musculus forkhead box O6 (Foxo6), mRNA [NM_194060]	chr4:119939747-119939688	11.85	9.84	4.02
263	A_55_P2176898	XM_001472766	Gm2185	PREDICTED: Mus musculus hypothetical protein LOC100039366 (LOC100039366), mRNA [XM_001472766]	chr12:50483166-50483107	8.10	6.09	4.02
264	A_30_P01019844	chr12:33808493-33829143_F		lincRNA:chr12:33808493-33829143 forward strand	chr12:33827246-33827305	6.59	8.59	0.25
265	A_30_P01029279	chr5:35975265-35982561_R		lincRNA:chr5:35975265-35982561 reverse strand	chr5:35976877-35976818	9.53	11.53	0.25
266	A_51_P299287	NM_053261	Impa2	Mus musculus inositol (myo)-1(or 4)-monophosphatase 2 (Impa2), mRNA [NM_053261]	chr18:67478382-67478441	5.82	7.82	0.25
267	A_55_P1980401	NM_144811	Cbx7	Mus musculus chromobox homolog 7 (Cbx7), mRNA [NM_144811]	chr15:79746299-79746240	7.07	9.08	0.25
268	A_55_P2230638	NM_199307	Ece1	Mus musculus endothelin converting enzyme 1 (Ece1), mRNA [NM_199307]	chr4:137519606-137519665	9.39	11.39	0.25
269	A_51_P137029	NM_019588	Plce1	Mus musculus phospholipase C, epsilon 1 (Plce1), mRNA [NM_019588]	chr19:38857137-38857196	6.88	8.88	0.25

270	A_30_P01023343	chr12:109044453-109045618_F		lincRNA:chr12:109044453-109045618 forward strand	chr12:109044893-109044952	6.51	8.52	0.25
271	A_55_P2107681	XM_001475869	Gm7321	PREDICTED: Mus musculus similar to High mobility group protein B2 (High mobility group protein 2) (HMG-2) (LOC641258), mRNA [XM_001475869]	chr14:5420918-5420977	7.24	9.24	0.25
272	A_55_P1983754	NM_025557	Pcp4l1	Mus musculus Purkinje cell protein 4-like 1 (Pcp4l1), mRNA [NM_025557]	chr1:173103482-173103423	6.73	8.73	0.25
273	A_30_P01028091	chr8:91569369-91594119_F		lincRNA:chr8:91569369-91594119 forward strand	chr8:91573566-91573625	5.86	7.87	0.25
274	A_51_P153557	NM_175199	Hspa12a	Mus musculus heat shock protein 12A (Hspa12a), mRNA [NM_175199]	chr19:58870580-58870521	7.99	9.99	0.25
275	A_55_P2038247	NM_019392	Tyro3	Mus musculus TYRO3 protein tyrosine kinase 3 (Tyro3), mRNA [NM_019392]	chr2:119643759-119643818	9.46	11.47	0.25
276	A_52_P588881	NM_001033484	Iqgap3	Mus musculus IQ motif containing GTPase activating protein 3 (Iqgap3), mRNA [NM_001033484]	chr3:87924765-87924824	8.35	10.36	0.25
277	A_55_P1959788	NM_001163731	Gm10697	Mus musculus predicted gene 10697 (Gm10697), mRNA [NM_001163731]	chr3:93764296-93764238	5.16	7.17	0.25
278	A_55_P2012804	NM_181819	Wfikkn2	Mus musculus WAP, follistatin/kazal, immunoglobulin, kunitz and netrin domain containing 2 (Wfikkn2), mRNA [NM_181819]	chr11:94098971-94098912	5.49	7.50	0.25
279	A_52_P635312	NM_010574	Irx2	Mus musculus Iroquois related homeobox 2 (Drosophila) (Irx2), mRNA [NM_010574]	chr13:72768784-72768843	6.15	8.16	0.25
280	A_55_P2058540	NM_011562	Tdgf1	Mus musculus teratocarcinoma-derived growth factor 1 (Tdgf1), mRNA [NM_011562]	chr9:110842925-110842866	5.25	7.26	0.25
281	A_55_P2146560	NM_207105	H2-Ab1	Mus musculus histocompatibility 2, class II antigen A, beta 1 (H2-Ab1), mRNA [NM_207105]	chr17:34406305-34406363	6.31	8.33	0.25
282	A_51_P503896	ENSMUST00000026133	Pycr1	pyrroline-5-carboxylate reductase 1 Gene [Source:MGI (curated);Acc:MGI:2384795] [ENSMUST00000026133]	chr11:120501238-120501179	8.85	10.87	0.25
283	A_52_P381553	NM_028055	Btbd17	Mus musculus BTB (POZ) domain containing 17 (Btbd17), mRNA [NM_028055]	chr11:114652781-114652723	6.53	8.55	0.25
284	A_51_P135732	ENSMUST00000031781		coiled-coil domain containing 136 Gene [Source:MGI (curated);Acc:MGI:1918128] [ENSMUST00000031781]	chr6:29368136-29376666	8.11	10.14	0.25
285	A_55_P1952256	NM_172301	Ccnb1	Mus musculus cyclin B1 (Ccnb1), mRNA [NM_172301]	chr13:101551283-101551224	9.65	11.67	0.25

286	A_55_P2173546	NM_175303	Sall4	Mus musculus sal-like 4 (Drosophila) (Sall4), transcript variant a, mRNA [NM_175303]	chr2:168580496-168580437	6.37	8.40	0.25
287	A_55_P2091676	NM_007987	Fas	Mus musculus Fas (TNF receptor superfamily member 6) (Fas), transcript variant 1, mRNA [NM_007987]	chr19:34402164-34402223	5.63	7.66	0.25
288	A_55_P2157033	NM_019517	Bace2	Mus musculus beta-site APP-cleaving enzyme 2 (Bace2), mRNA [NM_019517]	chr16:97658618-97658677	5.25	7.28	0.25
289	A_55_P1955771	XM_001479612	Gm9195	PREDICTED: Mus musculus similar to 3110045G13Rik protein (LOC668482), mRNA [XM_001479612]	chr14:72825879-72825820	6.41	8.44	0.25
290	A_30_P01024873	chr9:114412295-114418384_F		lincRNA:chr9:114412295-114418384 forward strand	chr9:114418097-114418156	7.55	9.58	0.25
291	A_51_P509098	NM_019734	Asah1	Mus musculus N-acylsphingosine amidohydrolase 1 (Asah1), mRNA [NM_019734]	chr8:42426740-42426681	9.71	11.73	0.25
292	A_55_P1983768	NM_009689	Birc5	Mus musculus baculoviral IAP repeat-containing 5 (Birc5), transcript variant 1, mRNA [NM_009689]	chr11:117711112-117711171	8.79	10.81	0.25
293	A_51_P152404	NM_026166	Ikbip	Mus musculus IKBKB interacting protein (Ikbip), transcript variant 1, mRNA [NM_026166]	chr10:90561290-90561349	7.38	9.41	0.25
294	A_55_P2109585	NM_172743	Plekha7	Mus musculus pleckstrin homology domain containing, family A member 7 (Plekha7), mRNA [NM_172743]	chr7:123267859-123267800	6.34	8.37	0.25
295	A_30_P01028000	chr9:114412295-114418384_F		lincRNA:chr9:114412295-114418384 forward strand	chr9:114418163-114418222	7.39	9.42	0.25
296	A_52_P354373	XM_001481164	1190002F15Rik	PREDICTED: Mus musculus RIKEN cDNA 1190002F15 gene (1190002F15Rik), mRNA [XM_001481164]	chr6:134901431-134901490	7.98	10.01	0.25
297	A_52_P381665	NM_133919	Aff1	Mus musculus AF4/FMR2 family, member 1 (Aff1), transcript variant 2, mRNA [NM_133919]	chr5:104284222-104284281	6.59	8.62	0.25
298	A_55_P1958464	XR_031621	Gm9252	PREDICTED: Mus musculus similar to Phosphoglycerate dehydrogenase (LOC677380), misc RNA [XR_031621]	chr7:48922020-48921961	6.99	9.02	0.25
299	A_52_P338605	XR_032468	Gm6639	PREDICTED: Mus musculus hypothetical protein LOC625963 (LOC625963), misc RNA [XR_032468]	chr3:35526429-35526370	5.82	7.86	0.24
300	A_55_P1977653	NM_001081390	Palld	Mus musculus palladin, cytoskeletal associated protein (Palld), mRNA [NM_001081390]	chr8:63994082-63994023	7.94	9.98	0.24
301	A_30_P01018962	chr8:4325209-4348788_F		lincRNA:chr8:4325209-4348788 forward strand	chr8:4348678-4348737	8.75	10.79	0.24

302	A_30_P01025418	chr18:5119300-5181600_F		lincRNA:chr18:5119300-5181600 forward strand	chr18:5162984-5163043	7.90	9.94	0.24
303	A_55_P2073219	NM_138646	Hps4	Mus musculus Hermansky-Pudlak syndrome 4 homolog (human) (Hps4), mRNA [NM_138646]	chr5:112807315-112807374	8.38	10.42	0.24
304	A_55_P2065866	NM_030206	Cygb	Mus musculus cytoglobin (Cygb), mRNA [NM_030206]	chr11:116507555-116507496	10.34	12.38	0.24
305	A_55_P2048833	NM_009501	Vax1	Mus musculus ventral anterior homeobox containing gene 1 (Vax1), mRNA [NM_009501]	chr19:59240804-59240745	6.22	8.26	0.24
306	A_52_P223626	NM_016967	Olig2	Mus musculus oligodendrocyte transcription factor 2 (Olig2), mRNA [NM_016967]	chr16:91228470-91228529	9.59	11.64	0.24
307	A_55_P1976589	NR_015513	E130114P18Rik	Mus musculus RIKEN cDNA E130114P18 gene (E130114P18Rik), non-coding RNA [NR_015513]	chr4:97234942-97234883	5.44	7.49	0.24
308	A_52_P124734	NM_028593	Cybrd1	Mus musculus cytochrome b reductase 1 (Cybrd1), mRNA [NM_028593]	chr2:70980706-70980765	5.53	7.57	0.24
309	A_51_P459108	NM_013754	Insl6	Mus musculus insulin-like 6 (Insl6), mRNA [NM_013754]	chr19:29395909-29395850	5.90	7.95	0.24
310	A_55_P2105858	NM_030693	Atf5	Mus musculus activating transcription factor 5 (Atf5), transcript variant 1, mRNA [NM_030693]	chr7:52067686-52067627	11.63	13.68	0.24
311	A_30_P01027696	chr2:33496712-33501183_F		lincRNA:chr2:33496712-33501183 forward strand	chr2:33500908-33500967	7.41	9.46	0.24
312	A_66_P136228	NM_030690	Rai14	Mus musculus retinoic acid induced 14 (Rai14), transcript variant 1, mRNA [NM_030690]	chr15:10498911-10498852	7.75	9.80	0.24
313	A_55_P2009752	NM_172563	Hlf	Mus musculus hepatic leukemia factor (Hlf), mRNA [NM_172563]	chr11:90200755-90200696	6.94	8.99	0.24
314	A_30_P01028433	chr1:88398401-88398644_R		lincRNA:chr1:88398401-88398644 reverse strand	chr1:88398545-88398486	7.89	9.94	0.24
315	A_55_P2068228	XR_033308	Gm8483	PREDICTED: Mus musculus similar to Eno1 protein (LOC667152), misc RNA [XR_033308]	chr14:15280209-15280150	12.30	14.35	0.24
316	A_55_P2039320	NM_178679	Zfp365	Mus musculus zinc finger protein 365 (Zfp365), mRNA [NM_178679]	chr10:67348967-67348908	7.54	9.59	0.24
317	A_55_P1970755	NM_024124	Hdac9	Mus musculus histone deacetylase 9 (Hdac9), mRNA [NM_024124]	chr12:35056464-35056405	7.12	9.17	0.24
318	A_51_P298107	NM_028813	Vit	Mus musculus vitrin (Vit), mRNA [NM_028813]	chr17:79026461-79026520	6.94	9.00	0.24
319	A_55_P1977855	NM_178280	Sall3	Mus musculus sal-like 3 (Drosophila) (Sall3), mRNA [NM_178280]	chr18:81163195-81163136	6.03	8.09	0.24

320	A_51_P382152	NM_011171	Procr	Mus musculus protein C receptor, endothelial (Procr), mRNA [NM_011171]	chr2:155580945-155581004	5.53	7.59	0.24
321	A_55_P1996578	NM_008010	Fgfr3	Mus musculus fibroblast growth factor receptor 3 (Fgfr3), transcript variant 1, mRNA [NM_008010]	chr5:34079649-34079708	10.73	12.79	0.24
322	A_51_P365516	NM_009258	Spink3	Mus musculus serine peptidase inhibitor, Kazal type 3 (Spink3), mRNA [NM_009258]	chr18:43887858-43887802	6.29	8.35	0.24
323	A_30_P01021593	chr5:35975265-35982561_F		lincRNA:chr5:35975265-35982561 forward strand	chr5:35976852-35976911	10.86	12.93	0.24
324	A_51_P318381	NM_008827	Pgf	Mus musculus placental growth factor (Pgf), mRNA [NM_008827]	chr12:86510337-86508335	8.11	10.18	0.24
325	A_66_P137775	XM_986896	A830035A12Rik	PREDICTED: Mus musculus RIKEN cDNA A830035A12 gene (A830035A12Rik), mRNA [XM_986896]	chr11:107393464-107393405	6.69	8.76	0.24
326	A_55_P2077088	NR_028271	Phxr4	Mus musculus per-hexamer repeat gene 4 (Phxr4), non-coding RNA [NR_028271]	chr9:13234932-13234991	7.55	9.62	0.24
327	A_52_P62121	NM_010338	Gpr37	Mus musculus G protein-coupled receptor 37 (Gpr37), mRNA [NM_010338]	chr6:25619040-25618981	5.62	7.69	0.24
328	A_66_P119457	NM_175189	Hepacam	Mus musculus hepatocyte cell adhesion molecule (Hepacam), mRNA [NM_175189]	chr9:37193184-37193243	6.34	8.42	0.24
329	A_55_P2444515	NM_001035533	Akap2	Mus musculus A kinase (PRKA) anchor protein 2 (Akap2), transcript variant 1, mRNA [NM_001035533]	chr4:57907544-57907603	7.54	9.61	0.24
330	A_55_P1971093	XR_031215	LOC242317	PREDICTED: Mus musculus similar to Ldha protein (LOC242317), misc RNA [XR_031215]	chr4:10366472-10366413	7.12	9.19	0.24
331	A_55_P2065074	ENSMUST00000085463		histone deacetylase 9 Gene [Source:MGI Symbol;Acc:MGI:1931221] [ENSMUST00000085463]	chr12:34792143-34792084	6.46	8.53	0.24
332	A_51_P354077	NM_153153	Svil	Mus musculus supervillin (Svil), transcript variant 1, mRNA [NM_153153]	chr18:5118987-5119046	6.29	8.36	0.24
333	A_55_P2146297	NM_010725	Lmx1b	Mus musculus LIM homeobox transcription factor 1 beta (Lmx1b), mRNA [NM_010725]	chr2:33420312-33420253	7.41	9.49	0.24
334	A_55_P2186928	XR_032647	Gm13337	PREDICTED: Mus musculus similar to 3-phosphoglycerate dehydrogenase (LOC637235), misc RNA [XR_032647]	chr2:21990326-21990385	5.99	8.07	0.24

335	A_55_P2141860	NM_026531	Aen	Mus musculus apoptosis enhancing nuclease (Aen), transcript variant 1, mRNA [NM_026531]	chr7:86053197-86053256	10.31	12.39	0.24
336	A_52_P274496	NM_183180	Tspan18	Mus musculus tetraspanin 18 (Tspan18), mRNA [NM_183180]	chr2:93042040-93041981	8.10	10.18	0.24
337	A_55_P1975837	XR_002032	Gm8659	PREDICTED: Mus musculus predicted gene, EG667483 (EG667483), misc RNA [XR_002032]	chrX:154589043-154589102	11.69	13.77	0.24
338	A_55_P2171897	NM_008714	Notch1	Mus musculus Notch gene homolog 1 (Drosophila) (Notch1), mRNA [NM_008714]	chr2:26319499-26319440	8.71	10.80	0.24
339	A_66_P115116	NM_133784	Wwtr1	Mus musculus WW domain containing transcription regulator 1 (Wwtr1), transcript variant 2, mRNA [NM_133784]	chr3:57260058-57259999	6.39	8.48	0.24
340	A_66_P105175	NM_009738	Bche	Mus musculus butyrylcholinesterase (Bche), mRNA [NM_009738]	chr3:73439794-73439735	6.88	8.97	0.24
341	A_55_P1979082	NM_001113331	Shc1	Mus musculus src homology 2 domain-containing transforming protein C1 (Shc1), transcript variant 1, mRNA [NM_001113331]	chr3:89233868-89233927	8.13	10.22	0.24
342	A_55_P1994887	NM_009569	Zfpm1	Mus musculus zinc finger protein, multitype 1 (Zfpm1), mRNA [NM_009569]	chr8:124861092-124861151	7.20	9.29	0.24
343	A_55_P2063251	NM_028207	Dusp3	Mus musculus dual specificity phosphatase 3 (vaccinia virus phosphatase VH1-related) (Dusp3), mRNA [NM_028207]	chr11:101835583-101835524	6.33	8.42	0.24
344	A_52_P285975	NM_001033253	Plekhg1	Mus musculus pleckstrin homology domain containing, family G (with RhoGef domain) member 1 (Plekhg1), transcript variant 2, mRNA [NM_001033253]	chr10:6382932-6382873	6.70	8.79	0.23
345	A_55_P2088550	NM_001162933	Rpl10l	Mus musculus ribosomal protein L10-like (Rpl10l), mRNA [NM_001162933]	chr12:67384462-67384403	5.49	7.58	0.23
346	A_55_P1969700	NM_207573	Olfr1380	Mus musculus olfactory receptor 1380 (Olfr1380), mRNA [NM_207573]	chr11:49377460-49377519	6.96	9.06	0.23
347	A_55_P1968703	NM_008115	Gfra2	Mus musculus glial cell line derived neurotrophic factor family receptor alpha 2 (Gfra2), mRNA [NM_008115]	chr14:71379586-71379645	8.33	10.42	0.23
348	A_55_P2057405	NM_013495	Cpt1a	Mus musculus carnitine palmitoyltransferase 1a, liver (Cpt1a), nuclear gene encoding mitochondrial protein, mRNA [NM_013495]	chr19:3384060-3384119	6.87	8.96	0.23

349	A_55_P2152836	XR_001688	Gm5223	PREDICTED: Mus musculus predicted gene, EG383211 (EG383211), misc RNA [XR_001688]	chr17:16919776-16919835	10.41	12.51	0.23
350	A_55_P1964163	NM_177785	BC049635	Mus musculus cDNA sequence BC049635 (BC049635), mRNA [NM_177785]	chr4:42881440-42881381	6.99	9.08	0.23
351	A_52_P570240	NM_029116	Kbtbd11	Mus musculus kelch repeat and BTB (POZ) domain containing 11 (Kbtbd11), mRNA [NM_029116]	chr8:15033192-15033251	9.48	11.58	0.23
352	A_51_P300618	NM_001163566	Crb2	Mus musculus crumbs homolog 2 (Drosophila) (Crb2), mRNA [NM_001163566]	chr2:37654440-37654499	6.64	8.75	0.23
353	A_51_P195506	NM_007778	Csf1	Mus musculus colony stimulating factor 1 (macrophage) (Csf1), transcript variant 1, mRNA [NM_007778]	chr3:107544392-107544333	6.22	8.32	0.23
354	A_66_P108796	NM_175651	Cnpy1	Mus musculus canopy 1 homolog (zebrafish) (Cnpy1), mRNA [NM_175651]	chr5:28532071-28532012	5.76	7.86	0.23
355	A_55_P2065513	NM_007897	Ebf1	Mus musculus early B-cell factor 1 (Ebf1), mRNA [NM_007897]	chr11:44818438-44818497	8.25	10.35	0.23
356	A_55_P2035613	NM_027154	Tmbim1	Mus musculus transmembrane BAX inhibitor motif containing 1 (Tmbim1), mRNA [NM_027154]	chr1:74334899-74334840	7.48	9.58	0.23
357	A_52_P79385	NM_025952	Magt1	Mus musculus magnesium transporter 1 (Magt1), transcript variant 2, mRNA [NM_025952]	chrX:103169548-103166784	8.89	10.99	0.23
358	A_55_P2010778	NM_001172054	Il11ra1	Mus musculus interleukin 11 receptor, alpha chain 1 (Il11ra1), transcript variant 3, mRNA [NM_001172054]	chr4:41716285-41716344	8.94	11.05	0.23
359	A_51_P502456	NR_004446	Gm7035	Mus musculus predicted pseudogene 7035 (Gm7035), non-coding RNA [NR_004446]	chr17:34112000-34111941	9.51	11.62	0.23
360	A_51_P393426	NM_011173	Pros1	Mus musculus protein S (alpha) (Pros1), mRNA [NM_011173]	chr16:62928674-62928733	6.85	8.96	0.23
361	A_55_P2108837	NM_009448	Tuba1c	Mus musculus tubulin, alpha 1C (Tuba1c), mRNA [NM_009448]	chr15:98868477-98868536	11.36	13.47	0.23
362	A_66_P112164	NM_144875	Rab7l1	Mus musculus RAB7, member RAS oncogene family-like 1 (Rab7l1), mRNA [NM_144875]	chr1:133769401-133769460	6.79	8.90	0.23
363	A_55_P2408848	NM_172671	Lgr4	Mus musculus leucine-rich repeat-containing G protein-coupled receptor 4 (Lgr4), mRNA [NM_172671]	chr2:109853567-109853626	7.47	9.58	0.23

364	A_55_P2174601	XM_001477810	LOC100047292	PREDICTED: Mus musculus similar to pleckstrin homology domain containing, family A member 7 (LOC100047292), mRNA [XM_001477810]	chr7:123267206-123267147	6.30	8.41	0.23
365	A_55_P1966432	NM_010358	Gstm1	Mus musculus glutathione S-transferase, mu 1 (Gstm1), mRNA [NM_010358]	chr3:107815654-107815595	7.93	10.05	0.23
366	A_55_P1958285	NM_178408	Arrdc1	Mus musculus arrestin domain containing 1 (Arrdc1), transcript variant 2, mRNA [NM_178408]	chr2:24782493-24782434	8.51	10.62	0.23
367	A_55_P1953400	NM_021477	A2bp1	Mus musculus ataxin 2 binding protein 1 (A2bp1), transcript variant 2, mRNA [NM_021477]	chr16:7409998-7410057	8.41	10.53	0.23
368	A_55_P1969002	NM_011535	Tbx3	Mus musculus T-box 3 (Tbx3), transcript variant 1, mRNA [NM_011535]	chr5:120134511-120134570	6.53	8.65	0.23
369	A_55_P2142830	NM_013610	Ninj1	Mus musculus ninjurin 1 (Ninj1), mRNA [NM_013610]	chr13:49291553-49291612	6.16	8.28	0.23
370	A_55_P2051334	NR_004446	Gm7035	Mus musculus predicted pseudogene 7035 (Gm7035), non-coding RNA [NR_004446]	chr17:034111979-034111920	9.66	11.78	0.23
371	A_52_P23225	NM_016697	Gpc3	Mus musculus glypican 3 (Gpc3), mRNA [NM_016697]	chrX:49625799-49625740	7.81	9.93	0.23
372	A_55_P2166501	NM_009851	Cd44	Mus musculus CD44 antigen (Cd44), transcript variant 1, mRNA [NM_009851]	chr2:102654337-102654278	7.20	9.32	0.23
373	A_55_P2110351	NM_144848	Eppk1	Mus musculus epiplakin 1 (Eppk1), mRNA [NM_144848]	chr15:75931995-75931936	7.27	9.39	0.23
374	A_55_P2053988	NM_010137	Epas1	Mus musculus endothelial PAS domain protein 1 (Epas1), mRNA [NM_010137]	chr17:87230587-87230646	6.25	8.37	0.23
375	A_52_P398998	NM_010279	Gfra1	Mus musculus glial cell line derived neurotrophic factor family receptor alpha 1 (Gfra1), mRNA [NM_010279]	chr19:58311663-58311604	8.45	10.57	0.23
376	A_55_P2091472	NM_009760	Bnip3	Mus musculus BCL2/adenovirus E1B interacting protein 3 (Bnip3), nuclear gene encoding mitochondrial protein, mRNA [NM_009760]	chr7:146082699-146082640	9.91	12.03	0.23
377	A_55_P2149983	NM_001110824	Foxp4	Mus musculus forkhead box P4 (Foxp4), transcript variant 1, mRNA [NM_001110824]	chr17:48004778-48004719	8.62	10.74	0.23
378	A_51_P223443	NM_133655	Cd81	Mus musculus CD81 antigen (Cd81), mRNA [NM_133655]	chr7:150253737-150253796	11.59	13.71	0.23
379	A_55_P2181655	NM_001083587	Tns3	Mus musculus tensin 3 (Tns3), mRNA [NM_001083587]	chr11:8334556-8334497	6.43	8.56	0.23

380	A_30_P01022621	chr6:127111250-127142925_F		lincRNA:chr6:127111250-127142925 forward strand	chr6:127133679-127133738	11.90	14.03	0.23
381	A_51_P371174	NM_013863	Bag3	Mus musculus BCL2-associated athanogene 3 (Bag3), mRNA [NM_013863]	chr7:135690281-135690340	7.78	9.90	0.23
382	A_51_P365008	NM_027884	Tns1	Mus musculus tensin 1 (Tns1), mRNA [NM_027884]	chr1:73956907-73956848	8.47	10.60	0.23
383	A_51_P243304	NM_016905	Galk1	Mus musculus galactokinase 1 (Galk1), mRNA [NM_016905]	chr11:115870183-115870124	8.53	10.66	0.23
384	A_51_P130727	NM_024169	Fkbp11	Mus musculus FK506 binding protein 11 (Fkbp11), mRNA [NM_024169]	chr15:98554868-98554809	7.34	9.48	0.23
385	A_30_P01022015	chr17:86560289-86565864_F		lincRNA:chr17:86560289-86565864 forward strand	chr17:86565318-86565377	6.71	8.84	0.23
386	A_55_P2047168	NM_008714	Notch1	Mus musculus Notch gene homolog 1 (Drosophila) (Notch1), mRNA [NM_008714]	chr2:26314689-26314630	8.14	10.27	0.23
387	A_55_P2108476	NM_144888	Mavs	Mus musculus mitochondrial antiviral signaling protein (Mavs), nuclear gene encoding mitochondrial protein, mRNA [NM_144888]	chr2:131073675-131073734	6.43	8.56	0.23
388	A_30_P01031936	chr1:63319842-63349796_F		lincRNA:chr1:63319842-63349796 forward strand	chr1:63327389-63327448	8.80	10.94	0.23
389	A_30_P01019651	chr3:41325168-41332366_R		lincRNA:chr3:41325168-41332366 reverse strand	chr3:41325289-41325230	5.80	7.93	0.23
390	A_51_P191782	NM_133859	Olfml3	Mus musculus olfactomedin-like 3 (Olfml3), mRNA [NM_133859]	chr3:103539649-103539590	7.08	9.22	0.23
391	A_55_P2083889	NM_011063	Pea15a	Mus musculus phosphoprotein enriched in astrocytes 15A (Pea15a), transcript variant 2, mRNA [NM_011063]	chr1:174127643-174127584	10.42	12.55	0.23
392	A_55_P1978716	NM_011769	Zim1	Mus musculus zinc finger, imprinted 1 (Zim1), mRNA [NM_011769]	chr7:6628801-6628742	6.73	8.87	0.23
393	A_55_P2028259	NM_172572	Rhbdf2	Mus musculus rhomboid 5 homolog 2 (Drosophila) (Rhbdf2), transcript variant 1, mRNA [NM_172572]	chr11:116459542-116459483	5.99	8.13	0.23
394	A_30_P01029187	chr12:33808493-33829143_F		lincRNA:chr12:33808493-33829143 forward strand	chr12:33819874-33819933	6.80	8.94	0.23
395	A_30_P01021383	chr1:34735032-34781146_F		lincRNA:chr1:34735032-34781146 forward strand	chr1:34780946-34781005	6.98	9.12	0.23
396	A_55_P2159885	NM_001166584	Tead1	Mus musculus TEA domain family member 1 (Tead1), transcript variant 1, mRNA [NM_001166584]	chr7:120043445-120043504	10.81	12.95	0.23
397	A_30_P01027648	chr3:83089315-83108396_F		lincRNA:chr3:83089315-83108396 forward strand	chr3:83104982-83105041	6.40	8.55	0.23

398	A_51_P112223	NM_010357	Gsta4	Mus musculus glutathione S-transferase, alpha 4 (Gsta4), mRNA [NM_010357]	chr9:78046172-78047344	6.95	9.10	0.23
399	A_51_P255456	NM_009994	Cyp1b1	Mus musculus cytochrome P450, family 1, subfamily b, polypeptide 1 (Cyp1b1), mRNA [NM_009994]	chr17:80106606-80106547	5.70	7.84	0.23
400	A_55_P2110910	NM_201411	Flrt1	Mus musculus fibronectin leucine rich transmembrane protein 1 (Flrt1), mRNA [NM_201411]	chr19:7169106-7169047	8.79	10.93	0.23
401	A_30_P01024606	chr5:35975265-35982561_F		lincRNA:chr5:35975265-35982561 forward strand	chr5:35976912-35976971	11.00	13.14	0.23
402	A_66_P115098	NM_001122953	Nfia	Mus musculus nuclear factor I/A (Nfia), transcript variant 3, mRNA [NM_001122953]	chr4:97680930-97680989	8.97	11.12	0.23
403	A_51_P183894	NM_015798	Fbxo15	Mus musculus F-box protein 15 (Fbxo15), mRNA [NM_015798]	chr18:85150693-85150752	6.74	8.89	0.23
404	A_30_P01020094	chr16:59557120-59602070_F		lincRNA:chr16:59557120-59602070 forward strand	chr16:59557771-59557830	6.74	8.89	0.23
405	A_55_P1967224	XR_004698	LOC674877	PREDICTED: Mus musculus hypothetical LOC674877 (LOC674877), misc RNA [XR_004698]		9.49	11.64	0.23
406	A_51_P501656	NM_212445	Kdelc2	Mus musculus KDEL (Lys-Asp-Glu-Leu) containing 2 (Kdelc2), mRNA [NM_212445]	chr9:53209495-53209554	5.82	7.97	0.23
407	A_52_P638798	NM_001164370	Mipol1	Mus musculus mirror-image polydactyly gene 1 homolog (human) (Mipol1), mRNA [NM_001164370]	chr12:58407026-58407085	8.37	10.52	0.23
408	A_65_P06147	NM_008010	Fgfr3	Mus musculus fibroblast growth factor receptor 3 (Fgfr3), transcript variant 1, mRNA [NM_008010]	chr5:34077856-34078090	9.14	11.29	0.23
409	A_51_P295442	NM_021605	Nek7	Mus musculus NIMA (never in mitosis gene a)-related expressed kinase 7 (Nek7), mRNA [NM_021605]	chr1:140381822-140381763	7.27	9.43	0.23
410	A_55_P2180481	XM_001478842	1810020O05Rik	PREDICTED: Mus musculus Riken cDNA 1810020O05 gene (1810020O05Rik), mRNA [XM_001478842]	chr6:87625838-87625779	6.15	8.31	0.22
411	A_52_P532227	NM_007901	S1pr1	Mus musculus sphingosine-1-phosphate receptor 1 (S1pr1), mRNA [NM_007901]	chr3:115414079-115414020	7.03	9.19	0.22
412	A_55_P1978136	NM_139064	Tnip2	Mus musculus TNFAIP3 interacting protein 2 (Tnip2), mRNA [NM_139064]	chr5:34838838-34838779	7.45	9.61	0.22
413	A_51_P149455	NM_007381	Acadl	Mus musculus acyl-Coenzyme A dehydrogenase, long-chain (Acadl), nuclear gene encoding mitochondrial protein, mRNA [NM_007381]	chr1:66877732-66877673	5.76	7.92	0.22

414	A_30_P01022329	chr3:83089315-83108396_F		lincRNA:chr3:83089315-83108396 forward strand	chr3:83104914-83104973	7.18	9.34	0.22
415	A_55_P2069995	XR_032158	Gm15707	PREDICTED: Mus musculus similar to L-lactate dehydrogenase A chain (LDH-A) (LDH muscle subunit) (LDH-M) (LOC546917), misc RNA [XR_032158]	chr6:145264785-145264726	7.92	10.08	0.22
416	A_51_P267239	NM_019980	Litaf	Mus musculus LPS-induced TN factor (Litaf), mRNA [NM_019980]	chr16:10959721-10959662	7.79	9.95	0.22
417	A_51_P507051	NM_021560	Bhlhe22	Mus musculus basic helix-loop-helix family, member e22 (Bhlhe22), mRNA [NM_021560]	chr3:17956925-17956984	6.81	8.97	0.22
418	A_55_P2043622	ENSMUST00000107819		glyoxylate reductase/hydroxypyruvate reductase Gene [Source:MGI (curated);Acc:MGI:1923488] [ENSMUST00000107819]	chr4:45003514-45003573	7.73	9.90	0.22
419	A_55_P2092526	NM_001164075	Tgif1	Mus musculus TGFB-induced factor homeobox 1 (Tgif1), transcript variant 1, mRNA [NM_001164075]	chr17:71193688-71193629	6.43	8.59	0.22
420	A_51_P110189	NM_053273	Ttyh2	Mus musculus tweety homolog 2 (Drosophila) (Ttyh2), mRNA [NM_053273]	chr11:114573076-114573135	5.70	7.86	0.22
421	A_51_P461191	NM_029674	Got1l1	Mus musculus glutamic-oxaloacetic transaminase 1-like 1 (Got1l1), mRNA [NM_029674]	chr8:28309887-28309828	5.57	7.74	0.22
422	A_30_P01025132	chr17:3004525-3086000_F		lincRNA:chr17:3004525-3086000 forward strand	chr17:3081683-3081742	7.42	9.58	0.22
423	A_51_P398723	NM_010228	Flt1	Mus musculus FMS-like tyrosine kinase 1 (Flt1), mRNA [NM_010228]	chr5:148373850-148373791	5.99	8.15	0.22
424	A_55_P2072661	NM_177782	Prex1	Mus musculus phosphatidylinositol-3,4,5-trisphosphate-dependent Rac exchange factor 1 (Prex1), mRNA [NM_177782]	chr2:166391941-166391882	7.27	9.44	0.22
425	A_55_P2029528	XM_001473540	LOC100044874	PREDICTED: Mus musculus similar to MHC H2-K 24 polypeptide, transcript variant 1 (LOC100044874), mRNA [XM_001473540]		8.45	10.62	0.22
426	A_55_P2062642	NM_025952	Magt1	Mus musculus magnesium transporter 1 (Magt1), transcript variant 2, mRNA [NM_025952]	chrX:103174801-103174745	9.77	11.95	0.22
427	A_55_P1987196	NM_001123367	Gm3448	Mus musculus predicted gene 3448 (Gm3448), mRNA [NM_001123367]	chr17:15132862-15132803	7.92	10.09	0.22

428	A_55_P2156274	NM_031257	Plekha2	Mus musculus pleckstrin homology domain-containing, family A (phosphoinositide binding specific) member 2 (Plekha2), mRNA [NM_031257]	chr8:26152694-26152635	6.43	8.60	0.22
429	A_52_P326354	NM_010155	Erf	Mus musculus Ets2 repressor factor (Erf), mRNA [NM_010155]	chr7:26028406-26028347	9.83	12.00	0.22
430	A_55_P2008443	NM_175418	Mybpc1	Mus musculus myosin binding protein C, slow-type (Mybpc1), mRNA [NM_175418]	chr10:87985866-87985807	5.44	7.62	0.22
431	A_55_P2293007	AK139019	AI662175	Mus musculus adult male aorta and vein cDNA, RIKEN full-length enriched library, clone:A530078F23 product:unclassifiable, full insert sequence. [AK139019]	chr16:19231495-19231436	5.62	7.79	0.22
432	A_55_P2012166	NM_026470	Spata6	Mus musculus spermatogenesis associated 6 (Spata6), mRNA [NM_026470]	chr4:111501642-111501701	6.84	9.01	0.22
433	A_51_P308844	NM_153529	Nrn1	Mus musculus neuritin 1 (Nrn1), mRNA [NM_153529]	chr13:36818000-36817941	6.69	8.86	0.22
434	A_55_P1974967	NM_033596	Hist2h4	Mus musculus histone cluster 2, H4 (Hist2h4), mRNA [NM_033596]	chr3:96067237-96067178	9.60	11.78	0.22
435	A_51_P414637	NM_015734	Col5a1	Mus musculus collagen, type V, alpha 1 (Col5a1), mRNA [NM_015734]	chr2:27892892-27892951	7.20	9.38	0.22
436	A_30_P01026489	chr15:92174226-92201201_R		lincRNA:chr15:92174226-92201201 reverse strand	chr15:92196395-92196336	6.59	8.76	0.22
437	A_55_P2023912	XR_030502	LOC630896	PREDICTED: Mus musculus similar to 3-phosphoglycerate dehydrogenase (LOC630896), misc RNA [XR_030502]	chr14:095820637-095820696	7.46	9.64	0.22
438	A_55_P2027836	NM_020275	Tnfrsf10b	Mus musculus tumor necrosis factor receptor superfamily, member 10b (Tnfrsf10b), mRNA [NM_020275]	chr14:70184141-70184200	7.18	9.36	0.22
439	A_51_P267278	NM_021301	Slc15a2	Mus musculus solute carrier family 15 (H+/peptide transporter), member 2 (Slc15a2), transcript variant 1, mRNA [NM_021301]	chr16:36750662-36750603	5.86	8.04	0.22
440	A_55_P2088571	NM_001079901	Repin1	Mus musculus replication initiator 1 (Repin1), transcript variant 1, mRNA [NM_001079901]	chr6:48549021-48549080	8.91	11.09	0.22
441	A_55_P2128153	NM_009933	Col6a1	Mus musculus collagen, type VI, alpha 1 (Col6a1), mRNA [NM_009933]	chr10:76171597-76171538	6.22	8.41	0.22
442	A_55_P1981714	NM_001177869	Rreb1	Mus musculus ras responsive element binding protein 1 (Rreb1), transcript variant 6, mRNA [NM_001177869]	chr13:38043797-38043856	7.57	9.76	0.22

443	A_51_P437240	NM_007929	Emp2	Mus musculus epithelial membrane protein 2 (Emp2), mRNA [NM_007929]	chr16:10282028-10281969	5.99	8.18	0.22
444	A_30_P01027153	chr11:76400207-76434732_F		lincRNA:chr11:76400207-76434732 forward strand	chr11:76412608-76412667	6.39	8.58	0.22
445	A_30_P01031324	chr2:115900355-115900904_F		lincRNA:chr2:115900355-115900904 forward strand	chr2:115900836-115900895	6.40	8.59	0.22
446	A_66_P119801	NM_181848	Optn	Mus musculus optineurin (Optn), mRNA [NM_181848]	chr2:4954246-4954187	6.67	8.86	0.22
447	A_55_P2141884	NM_178598	Tagln2	Mus musculus transgelin 2 (Tagln2), mRNA [NM_178598]	chr1:174436738-174436797	7.05	9.24	0.22
448	A_51_P284244	NM_025360	Tmed3	Mus musculus transmembrane emp24 domain containing 3 (Tmed3), mRNA [NM_025360]	chr9:89594110-89594051	10.42	12.61	0.22
449	A_66_P118863	NM_134094	Ncald	Mus musculus neurocalcin delta (Ncald), transcript variant 1, mRNA [NM_134094]	chr15:37296016-37295957	6.61	8.80	0.22
450	A_55_P2025730	NM_016868	Hif3a	Mus musculus hypoxia inducible factor 3, alpha subunit (Hif3a), transcript variant 2, mRNA [NM_016868]	chr7:17620870-17620811	6.67	8.86	0.22
451	A_51_P483373	NM_145367	Txndc5	Mus musculus thioredoxin domain containing 5 (Txndc5), mRNA [NM_145367]	chr13:38592739-38592680	9.31	11.50	0.22
452	A_51_P446825	NM_176952	6430573F11Rik	Mus musculus RIKEN cDNA 6430573F11 gene (6430573F11Rik), mRNA [NM_176952]	chr8:37575761-37575820	5.90	8.10	0.22
453	A_55_P1972842	NM_008453	Klf3	Mus musculus Kruppel-like factor 3 (basic) (Klf3), mRNA [NM_008453]	chr5:65221309-65221368	7.69	9.89	0.22
454	A_51_P160913	NM_008209	Mr1	Mus musculus major histocompatibility complex, class I-related (Mr1), mRNA [NM_008209]	chr1:156975282-156975223	5.30	7.50	0.22
455	A_51_P451516	NM_028027	D10Ertd610e	Mus musculus DNA segment, Chr 10, ERATO Doi 610, expressed (D10Ertd610e), transcript variant 1, mRNA [NM_028027]	chr10:126619670-126619611	9.91	12.11	0.22
456	A_55_P2071566	ENSMUST00000090371		methyltransferase like 14 Gene [Source:MGI Symbol;Acc:MGI:2442926] [ENSMUST00000090371]	chr3:123067953-123067894	5.53	7.73	0.22
457	A_52_P175376	NM_023755	Tcfcp2l1	Mus musculus transcription factor CP2-like 1 (Tcfcp2l1), mRNA [NM_023755]	chr1:120581646-120581705	6.94	9.14	0.22
458	A_55_P2022211	NM_026162	Plxdc2	Mus musculus plexin domain containing 2 (Plxdc2), mRNA [NM_026162]	chr2:16673217-16673276	8.11	10.31	0.22
459	A_66_P108267	NM_148935	Foxn4	Mus musculus forkhead box N4 (Foxn4), mRNA [NM_148935]	chr5:114704268-114704209	6.25	8.46	0.22

460	A_55_P2051414	NM_009465	Axl	Mus musculus AXL receptor tyrosine kinase (Axl), mRNA [NM_009465]	chr7:26542600-26542541	6.07	8.28	0.22
461	A_51_P440743	NM_009886	Celsr1	Mus musculus cadherin, EGF LAG seven-pass G-type receptor 1 (flamingo homolog, Drosophila) (Celsr1), mRNA [NM_009886]	chr15:85729440-85729381	7.67	9.88	0.22
462	A_52_P638895	NM_001025250	Vegfa	Mus musculus vascular endothelial growth factor A (Vegfa), transcript variant 1, mRNA [NM_001025250]	chr17:46154446-46154387	9.98	12.19	0.22
463	A_51_P405606	NM_008681	Ndrg1	Mus musculus N-myc downstream regulated gene 1 (Ndrg1), mRNA [NM_008681]	chr15:66761116-66761057	5.66	7.87	0.22
464	A_55_P2004179	NM_001113515	Col2a1	Mus musculus collagen, type II, alpha 1 (Col2a1), transcript variant 2, mRNA [NM_001113515]	chr15:97806130-97806071	6.95	9.16	0.22
465	A_52_P469306	NM_013627	Pax6	Mus musculus paired box gene 6 (Pax6), mRNA [NM_013627]	chr2:105524098-105524949	6.03	8.25	0.22
466	A_55_P1975690	NM_031397	Bicc1	Mus musculus bicaudal C homolog 1 (Drosophila) (Bicc1), mRNA [NM_031397]	chr10:70387909-70387850	5.90	8.12	0.22
467	A_30_P01025655	chr14:26842169-26845456_F		lincRNA:chr14:26842169-26845456 forward strand	chr14:26845272-26845331	5.85	8.06	0.22
468	A_30_P01022913	chr4:56480568-56658018_R		lincRNA:chr4:56480568-56658018 reverse strand	chr4:56617644-56617585	6.56	8.78	0.22
469	A_52_P261322	NM_198294	Tanc1	Mus musculus tetratricopeptide repeat, ankyrin repeat and coiled-coil containing 1 (Tanc1), mRNA [NM_198294]	chr2:59681626-59681685	6.22	8.44	0.22
470	A_51_P254262	NM_130858	Nxph3	Mus musculus neurexophilin 3 (Nxph3), mRNA [NM_130858]	chr11:95371232-95371173	7.21	9.43	0.22
471	A_55_P2025454	NM_172770	Ttc12	Mus musculus tetratricopeptide repeat domain 12 (Ttc12), mRNA [NM_172770]	chr9:49253354-49253295	5.74	7.96	0.22
472	A_55_P2116924	NM_001037999	Dbi	Mus musculus diazepam binding inhibitor (Dbi), transcript variant 1, mRNA [NM_001037999]	chr1:122009976-122009917	11.42	13.64	0.22
473	A_30_P01024645	chr6:127111250-127142925_F		lincRNA:chr6:127111250-127142925 forward strand	chr6:127133587-127133646	11.76	13.98	0.22
474	A_52_P585124	NM_009911	Cxcr4	Mus musculus chemokine (C-X-C motif) receptor 4 (Cxcr4), mRNA [NM_009911]	chr1:130485138-130485079	11.10	13.32	0.21
475	A_55_P1973941	NM_011404	Slc7a5	Mus musculus solute carrier family 7 (cationic amino acid transporter, y+ system), member 5 (Slc7a5), mRNA [NM_011404]	chr8:124405195-124405136	9.06	11.28	0.21

476	A_55_P1968295	NM_028733	Pacsin3	Mus musculus protein kinase C and casein kinase substrate in neurons 3 (Pacsin3), mRNA [NM_028733]	chr2:91104768-91104827	7.69	9.92	0.21
477	A_52_P612803	NM_009831	Ccng1	Mus musculus cyclin G1 (Ccng1), mRNA [NM_009831]	chr11:40562486-40562427	7.76	9.98	0.21
478	A_52_P634090	NM_013822	Jag1	Mus musculus jagged 1 (Jag1), mRNA [NM_013822]	chr2:136907492-136907433	8.15	10.37	0.21
479	A_51_P206405	NM_001081306	Ptprz1	Mus musculus protein tyrosine phosphatase, receptor type Z, polypeptide 1 (Ptprz1), mRNA [NM_001081306]	chr6:23002046-23002106	9.48	11.70	0.21
480	A_51_P220806	NM_008110	Gdf9	Mus musculus growth differentiation factor 9 (Gdf9), mRNA [NM_008110]	chr11:53251307-53251366	7.33	9.55	0.21
481	A_66_P121495	NM_177420	Psat1	Mus musculus phosphoserine aminotransferase 1 (Psat1), mRNA [NM_177420]	chr19:15991645-15991586	9.09	11.31	0.21
482	A_55_P2106235	NM_009304	Syngr2	Mus musculus synaptogyrin 2 (Syngr2), mRNA [NM_009304]	chr11:117675536-117675595	7.25	9.48	0.21
483	A_51_P329332	NM_054087	Slc19a2	Mus musculus solute carrier family 19 (thiamine transporter), member 2 (Slc19a2), mRNA [NM_054087]	chr1:166195274-166195333	8.23	10.46	0.21
484	A_55_P2002903	NM_022315	Smoc2	Mus musculus SPARC related modular calcium binding 2 (Smoc2), mRNA [NM_022315]	chr17:14541664-14541723	6.31	8.54	0.21
485	A_51_P452779	NM_133198	Pygl	Mus musculus liver glycogen phosphorylase (Pygl), mRNA [NM_133198]	chr12:71291914-71291855	7.67	9.90	0.21
486	A_55_P2083197	NM_001013813	Maml2	Mus musculus mastermind like 2 (Drosophila) (Maml2), transcript variant 1, mRNA [NM_001013813]	chr9:13424194-13424253	7.54	9.77	0.21
487	A_51_P304170	NM_023386	Rtp4	Mus musculus receptor transporter protein 4 (Rtp4), mRNA [NM_023386]	chr16:23613668-23613727	6.90	9.14	0.21
488	A_55_P2005655	NM_018766	Ntsr1	Mus musculus neurotensin receptor 1 (Ntsr1), mRNA [NM_018766]	chr2:180279625-180279684	7.38	9.62	0.21
489	A_52_P573467	NM_008644	Prol1	Mus musculus proline rich, lacrimal 1 (Prol1), mRNA [NM_008644]	chr5:88757596-88757655	5.47	7.71	0.21
490	A_30_P01029071	chr9:14460976-14488051_F		lincRNA:chr9:14460976-14488051 forward strand	chr9:14484433-14484492	8.17	10.41	0.21
491	A_55_P2101001	NM_016769	Smad3	Mus musculus MAD homolog 3 (Drosophila) (Smad3), mRNA [NM_016769]	chr9:63494633-63494574	8.73	10.98	0.21
492	A_55_P2010871	NM_001008231	Daam2	Mus musculus dishevelled associated activator of morphogenesis 2 (Daam2), mRNA [NM_001008231]	chr17:49595451-49595392	6.25	8.50	0.21

493	A_30_P01024116	chr17:86560289-86565864_F		lincRNA:chr17:86560289-86565864 forward strand	chr17:86565385-86565444	6.11	8.36	0.21
494	A_51_P233101	NM_008520	Ltbp3	Mus musculus latent transforming growth factor beta binding protein 3 (Ltbp3), mRNA [NM_008520]	chr19:5758289-5758346	6.46	8.71	0.21
495	A_55_P2134591	NM_178776	BC049715	Mus musculus cDNA sequence BC049715 (BC049715), mRNA [NM_178776]	chr6:136788994-136789053	6.15	8.40	0.21
496	A_51_P257885	NM_175217	Mmd2	Mus musculus monocyte to macrophage differentiation-associated 2 (Mmd2), mRNA [NM_175217]	chr5:143039987-143039928	7.24	9.49	0.21
497	A_55_P2100928	NM_008963	Ptgds	Mus musculus prostaglandin D2 synthase (brain) (Ptgds), mRNA [NM_008963]	chr2:25322291-25322232	6.37	8.64	0.21
498	A_66_P104618	ENSMUST00000069762		Putative uncharacterized protein [Source:UniProtKB/TrEMBL;Acc:Q3U4J3] [ENSMUST00000069762]	chr8:4348678-4348737	8.43	10.69	0.21
499	A_55_P1974587	NM_177545	Vangl1	Mus musculus vang-like 1 (van gogh, Drosophila) (Vangl1), mRNA [NM_177545]	chr3:101962189-101962130	5.95	8.21	0.21
500	A_55_P1959748	NM_012055	Asns	Mus musculus asparagine synthetase (Asns), mRNA [NM_012055]	chr6:7625228-7625171	8.89	11.16	0.21
501	A_55_P2116880	NM_009501	Vax1	Mus musculus ventral anterior homeobox containing gene 1 (Vax1), mRNA [NM_009501]	chr19:59240736-59240677	6.53	8.80	0.21
502	A_30_P01032379	chr4:149383134-149451259_R		lincRNA:chr4:149383134-149451259 reverse strand	chr4:149433600-149433541	8.53	10.79	0.21
503	A_30_P01022828	chr18:82862340-82875566_F		lincRNA:chr18:82862340-82875566 forward strand	chr18:82875058-82875117	5.72	7.99	0.21
504	A_55_P2034625	ENSMUST00000063878		sema domain, transmembrane domain (TM), and cytoplasmic domain, (semaphorin) 6A Gene [Source:MGI (curated);Acc:MGI:1203727] [ENSMUST00000063878]	chr18:47436114-47436055	7.18	9.45	0.21
505	A_51_P125260	NM_177470	Acaa2	Mus musculus acetyl-Coenzyme A acyltransferase 2 (mitochondrial 3-oxoacyl-Coenzyme A thiolase) (Acaa2), nuclear gene encoding mitochondrial protein, mRNA [NM_177470]	chr18:74965691-74965750	8.57	10.84	0.21
506	A_66_P134775	NM_008961	Pter	Mus musculus phosphotriesterase related (Pter), mRNA [NM_008961]	chr2:12916429-12916488	5.44	7.71	0.21
507	A_55_P2008066	NM_010585	Itpr1	Mus musculus inositol 1,4,5-triphosphate receptor 1 (Itpr1), mRNA [NM_010585]	chr6:108501025-108501084	6.82	9.09	0.21

508	A_52_P162099	NM_001004140	Ckap2	Mus musculus cytoskeleton associated protein 2 (Ckap2), mRNA [NM_001004140]	chr8:23279443-23279384	8.38	10.65	0.21
509	A_52_P545810	NM_001111311	Lrrfip1	Mus musculus leucine rich repeat (in FLII) interacting protein 1 (Lrrfip1), transcript variant 1, mRNA [NM_001111311]	chr1:93013805-93013864	7.43	9.70	0.21
510	A_52_P597371	NM_134094	Ncald	Mus musculus neurocalcin delta (Ncald), transcript variant 1, mRNA [NM_134094]	chr15:37327074-37327015	6.96	9.24	0.21
511	A_55_P2126192	NM_010195	Lgr5	Mus musculus leucine rich repeat containing G protein coupled receptor 5 (Lgr5), mRNA [NM_010195]	chr10:114887462-114887403	6.30	8.58	0.21
512	A_51_P428134	NM_177152	Lrig3	Mus musculus leucine-rich repeats and immunoglobulin-like domains 3 (Lrig3), mRNA [NM_177152]	chr10:125452006-125452065	6.33	8.61	0.21
513	A_55_P2156186	NM_153513	BC028528	Mus musculus cDNA sequence BC028528 (BC028528), mRNA [NM_153513]	chr3:95692155-95692096	6.51	8.79	0.21
514	A_55_P2064547	BC022182	Tuba1c	Mus musculus tubulin, alpha 1C, mRNA (cDNA clone MGC:14067 IMAGE:3661220), complete cds. [BC022182]	chr15:98868488-98868547	10.74	13.02	0.21
515	A_30_P01029720	NEAT1		lincRNA:chr19:5842306-5845476 reverse strand	chr19:5842713-5842654	6.70	8.98	0.21
516	A_55_P2162782	NM_009643	Ahnak	Mus musculus AHNAK nucleoprotein (desmoyokin) (Ahnak), transcript variant 1, mRNA [NM_009643]	chr19:9090559-9090618	6.09	8.37	0.21
517	A_51_P201721	NM_019922	Crtap	Mus musculus cartilage associated protein (Crtap), mRNA [NM_019922]	chr9:114284518-114284459	6.78	9.06	0.21
518	A_55_P2176325	NM_177782	Prex1	Mus musculus phosphatidylinositol-3,4,5-trisphosphate-dependent Rac exchange factor 1 (Prex1), mRNA [NM_177782]	chr2:166392552-166392493	8.21	10.50	0.21
519	A_55_P2021455	XR_032442	Gm8767	PREDICTED: Mus musculus similar to aldolase 1, A isoform (LOC667696), misc RNA [XR_032442]	chr10:29804294-29804235	8.74	11.03	0.21
520	A_51_P383270	NM_175473	Fras1	Mus musculus Fraser syndrome 1 homolog (human) (Fras1), mRNA [NM_175473]	chr5:97213218-97213277	8.39	10.67	0.21
521	A_51_P113182	NM_013584	Lifr	Mus musculus leukemia inhibitory factor receptor (Lifr), transcript variant 1, mRNA [NM_013584]	chr15:7141150-7141209	7.50	9.78	0.21

522	A_51_P268094	NM_009255	Serpine2	Mus musculus serine (or cysteine) peptidase inhibitor, clade E, member 2 (Serpine2), mRNA [NM_009255]	chr1:79796093-79793476	8.16	10.45	0.21
523	A_55_P2017929	NM_013492	Clu	Mus musculus clusterin (Clu), mRNA [NM_013492]	chr14:66600325-66600382	7.86	10.15	0.20
524	A_55_P2093874	NM_146131	Pbxip1	Mus musculus pre-B-cell leukemia transcription factor interacting protein 1 (Pbxip1), mRNA [NM_146131]	chr3:89252815-89252874	7.21	9.50	0.20
525	A_55_P2099620	NM_145998	Hmx2	Mus musculus H6 homeobox 2 (Hmx2), mRNA [NM_145998]	chr7:138700012-138700071	6.99	9.29	0.20
526	A_55_P1968808	NM_145940	Wipi1	Mus musculus WD repeat domain, phosphoinositide interacting 1 (Wipi1), mRNA [NM_145940]	chr11:109443745-109443686	6.37	8.67	0.20
527	A_30_P01025584	chr1:74084292-74138492_R		lincRNA:chr1:74084292-74138492 reverse strand	chr1:74104006-74103947	6.21	8.50	0.20
528	A_30_P01031825	chr8:122712014-122732520_F		lincRNA:chr8:122712014-122732520 forward strand	chr8:122727472-122727531	6.40	8.70	0.20
529	A_55_P2046245	NM_008235	Hes1	Mus musculus hairy and enhancer of split 1 (Drosophila) (Hes1), mRNA [NM_008235]	chr16:30067820-30067879	6.59	8.88	0.20
530	A_51_P368009	NM_177733	E2f2	Mus musculus E2F transcription factor 2 (E2f2), mRNA [NM_177733]	chr4:135750562-135750621	8.94	11.24	0.20
531	A_55_P2129498	NR_033594	Gm5712	Mus musculus predicted gene 5712 (Gm5712), non-coding RNA [NR_033594]	chr3:129137407-129137466	6.01	8.31	0.20
532	A_51_P477682	NM_008939	Prss12	Mus musculus protease, serine, 12 neurotrypsin (motopsin) (Prss12), mRNA [NM_008939]	chr3:123209409-123209468	7.92	10.22	0.20
533	A_30_P01032922	chr16:46384974-46391331_F		lincRNA:chr16:46384974-46391331 forward strand	chr16:46389805-46389864	6.31	8.62	0.20
534	A_52_P403443	NM_001083587	Tns3	Mus musculus tensin 3 (Tns3), mRNA [NM_001083587]	chr11:8332246-8332187	5.88	8.20	0.20
535	A_30_P01031970	chr2:115900355-115900904_F		lincRNA:chr2:115900355-115900904 forward strand	chr2:115900741-115900800	6.34	8.66	0.20
536	A_30_P01032597	chr14:55433382-55452757_F		lincRNA:chr14:55433382-55452757 forward strand	chr14:55445279-55445338	9.06	11.37	0.20
537	A_52_P146865	NM_053136	Pcdhb11	Mus musculus protocadherin beta 11 (Pcdhb11), mRNA [NM_053136]	chr18:37583696-37583755	7.45	9.76	0.20
538	A_55_P2095508	ENSMUST00000121245		poliovirus receptor-related 3 Gene [Source:MGI (curated);Acc:MGI:1930171] [ENSMUST00000121245]	chr16:46395161-46395102	8.21	10.53	0.20
539	A_55_P2145804	NM_026531	Aen	Mus musculus apoptosis enhancing nuclease (Aen), transcript variant 1, mRNA [NM_026531]	chr7:86053207-86053266	10.43	12.75	0.20

540	A_55_P1955457	NM_010345	Grb10	Mus musculus growth factor receptor bound protein 10 (Grb10), transcript variant 1, mRNA [NM_010345]	chr11:11833224-11833165	9.95	12.28	0.20
541	A_55_P2080151	NM_008301	Hspa2	Mus musculus heat shock protein 2 (Hspa2), transcript variant 1, mRNA [NM_008301]	chr12:77507769-77507828	7.99	10.31	0.20
542	A_66_P118093	XM_001480420	LOC100048617	PREDICTED: Mus musculus hypothetical protein LOC100048617 (LOC100048617), mRNA [XM_001480420]	chr2:119146982-119146923	6.15	8.48	0.20
543	A_52_P137765	NM_019390	Lmna	Mus musculus lamin A (Lmna), transcript variant 2, mRNA [NM_019390]	chr3:88287321-88287262	7.09	9.42	0.20
544	A_55_P2085485	NM_022654	Lrdd	Mus musculus leucine-rich and death domain containing (Lrdd), mRNA [NM_022654]	chr7:148624475-148624416	7.41	9.74	0.20
545	A_30_P01027939	chr1:71939741-71947891_F		lincRNA:chr1:71939741-71947891 forward strand	chr1:71940191-71940250	6.33	8.66	0.20
546	A_55_P1985788	NM_198092	Usp2	Mus musculus ubiquitin specific peptidase 2 (Usp2), transcript variant 3, mRNA [NM_198092]	chr9:43903649-43903708	5.82	8.15	0.20
547	A_30_P01026843	chr5:96591892-96606467_R		lincRNA:chr5:96591892-96606467 reverse strand	chr5:96603179-96603120	8.79	11.13	0.20
548	A_51_P458866	NM_172647	F11r	Mus musculus F11 receptor (F11r), mRNA [NM_172647]	chr1:173393848-173393907	7.61	9.95	0.20
549	A_55_P2182955	NM_175087	Aqp6	Mus musculus aquaporin 6 (Aqp6), mRNA [NM_175087]	chr15:99435849-99435908	6.16	8.50	0.20
550	A_52_P522379	NM_178084	B230120H23Rik	Mus musculus RIKEN cDNA B230120H23 gene (B230120H23Rik), transcript variant 2, mRNA [NM_178084]	chr2:72227533-72227592	8.00	10.35	0.20
551	A_30_P01025778	chr1:71939741-71947891_R		lincRNA:chr1:71939741-71947891 reverse strand	chr1:71940250-71940191	5.51	7.85	0.20
552	A_51_P505868	NM_175386	Lhfp	Mus musculus lipoma HMGIC fusion partner (Lhfp), mRNA [NM_175386]	chr3:53064744-53064803	8.25	10.59	0.20
553	A_52_P418814	NM_029971	Pmch	Mus musculus pro-melanin-concentrating hormone (Pmch), mRNA [NM_029971]	chr10:87555031-87555090	7.89	10.24	0.20
554	A_55_P2183735	NM_177298	Pisd	Mus musculus phosphatidylserine decarboxylase (Pisd), mRNA [NM_177298]	chr5:33079477-33079418	9.34	11.69	0.20
555	A_30_P01023479	chr8:122728333-122752130_R		lincRNA:chr8:122728333-122752130 reverse strand	chr8:122728393-122728334	7.17	9.52	0.20
556	A_55_P2089219	XM_001476512	LOC100046616	PREDICTED: Mus musculus similar to aquaporin 5 (LOC100046616), mRNA [XM_001476512]	chr15:99425214-99425273	6.52	8.87	0.20

557	A_55_P2072095	NM_001033366	Dpcr1	Mus musculus diffuse panbronchiolitis critical region 1 (human) (Dpcr1), mRNA [NM_001033366]	chr17:35772825-35772766	5.40	7.75	0.20
558	A_55_P2056729	NM_008342	Igfbp2	Mus musculus insulin-like growth factor binding protein 2 (Igfbp2), mRNA [NM_008342]	chr1:72898677-72898736	9.68	12.04	0.20
559	A_55_P2127357	XR_031313	LOC100045143	PREDICTED: Mus musculus similar to Tpd52 protein (LOC100045143), misc RNA [XR_031313]	chr8:82202919-82202860	9.20	11.56	0.20
560	A_66_P119045	ENSMUST00000071101		Putative uncharacterized protein Fragment [Source:UniProtKB/TrEMBL;Acc:Q8CA40] [ENSMUST00000071101]	chr6:128137932-128137873	6.76	9.11	0.20
561	A_55_P2027392	NM_030258	Gpr146	Mus musculus G protein-coupled receptor 146 (Gpr146), transcript variant 1, mRNA [NM_030258]	chr5:139872290-139872349	7.18	9.53	0.20
562	A_51_P496309	NM_001024918	Rfx4	Mus musculus regulatory factor X, 4 (influences HLA class II expression) (Rfx4), transcript variant 1, mRNA [NM_001024918]	chr10:84368464-84368523	6.70	9.06	0.20
563	A_30_P01028673	chr9:14460976-14488051_F		lincRNA:chr9:14460976-14488051 forward strand	chr9:14484590-14484649	8.10	10.45	0.20
564	A_55_P2067727	NM_026280	Mxra7	Mus musculus matrix-remodelling associated 7 (Mxra7), mRNA [NM_026280]	chr11:116673310-116673251	7.97	10.33	0.20
565	A_51_P445532	NM_020492	Glra1	Mus musculus glycine receptor, alpha 1 subunit (Glra1), mRNA [NM_020492]	chr11:55379742-55349979	5.42	7.78	0.20
566	A_30_P01026774	chr4:145539725-145596000_F		lincRNA:chr4:145539725-145596000 forward strand	chr4:145586971-145587030	8.08	10.44	0.19
567	A_52_P279152	NM_016794	Vamp8	Mus musculus vesicle-associated membrane protein 8 (Vamp8), mRNA [NM_016794]	chr6:72335579-72335520	7.07	9.44	0.19
568	A_55_P2040951	NM_009608	Actc1	Mus musculus actin, alpha, cardiac muscle 1 (Actc1), mRNA [NM_009608]	chr2:113873123-113873064	5.25	7.61	0.19
569	A_30_P01021374	chr6:47692090-47713542_F		lincRNA:chr6:47692090-47713542 forward strand	chr6:47694236-47694295	8.85	11.22	0.19
570	A_55_P1992910	NM_013755	Gyg	Mus musculus glycogenin (Gyg), mRNA [NM_013755]	chr3:20022092-20022033	7.61	9.98	0.19
571	A_30_P01032317	chr8:122728333-122752130_R		lincRNA:chr8:122728333-122752130 reverse strand	chr8:122728676-122728617	6.11	8.48	0.19
572	A_30_P01029858	chr4:149383134-149451259_R		lincRNA:chr4:149383134-149451259 reverse strand	chr4:149433540-149433481	8.35	10.72	0.19
573	A_55_P2234231	NR_030701	D430050G20	Mus musculus hypothetical protein D430050G20 (D430050G20), non-coding RNA [NR_030701]	chr13:72761027-72760968	5.20	7.58	0.19

574	A_52_P355169	NM_011607	Tnc	Mus musculus tenascin C (Tnc), mRNA [NM_011607]	chr4:63675011-63674248	5.74	8.11	0.19
575	A_30_P01026204	chr17:86560289-86565864_R		lincRNA:chr17:86560289-86565864 reverse strand	chr17:86565468-86565409	6.03	8.41	0.19
576	A_55_P2097869	NM_172862	Frem2	Mus musculus Fras1 related extracellular matrix protein 2 (Frem2), mRNA [NM_172862]	chr3:53318064-53318005	7.47	9.86	0.19
577	A_55_P2000489	NM_172523	Slc18a2	Mus musculus solute carrier family 18 (vesicular monoamine), member 2 (Slc18a2), mRNA [NM_172523]	chr19:59370153-59370212	8.02	10.41	0.19
578	A_51_P267933	NM_175692	Snhg11	Mus musculus small nucleolar RNA host gene 11 (non-protein coding) (Snhg11), mRNA [NM_175692]	chr2:158211554-158211613	10.76	13.15	0.19
579	A_65_P16059	NM_011578	Tgfbr3	Mus musculus transforming growth factor, beta receptor III (Tgfbr3), mRNA [NM_011578]	chr5:107535877-107535818	6.63	9.02	0.19
580	A_52_P88818	NM_029556	Clybl	Mus musculus citrate lyase beta like (Clybl), mRNA [NM_029556]	chr14:122775148-122777007	6.75	9.14	0.19
581	A_52_P635015	NM_027677	Gpr39	Mus musculus G protein-coupled receptor 39 (Gpr39), mRNA [NM_027677]	chr1:127769282-127769341	5.86	8.25	0.19
582	A_30_P01029551	chr15:61984389-62102500_F		lincRNA:chr15:61984389-62102500 forward strand	chr15:62086046-62086105	6.43	8.83	0.19
583	A_55_P1997141	NM_008652	Mybl2	Mus musculus myeloblastosis oncogene-like 2 (Mybl2), mRNA [NM_008652]	chr2:162909345-162909404	8.17	10.57	0.19
584	A_55_P1972386	ENSMUST00000089883		mannan-binding lectin serine peptidase 1 Gene [Source:MGI Symbol;Acc:MGI:88492] [ENSMUST00000089883]	chr16:23470037-23469978	7.63	10.03	0.19
585	A_30_P01023944	chr8:122712014-122732520_R		lincRNA:chr8:122712014-122732520 reverse strand	chr8:122725354-122725295	8.17	10.58	0.19
586	A_55_P2001583	NM_183180	Tspan18	Mus musculus tetraspanin 18 (Tspan18), mRNA [NM_183180]	chr2:93043472-93043413	10.45	12.86	0.19
587	A_66_P128199	NM_022721	Fzd5	Mus musculus frizzled homolog 5 (Drosophila) (Fzd5), transcript variant 1, mRNA [NM_022721]	chr1:64779538-64779479	6.41	8.82	0.19
588	A_30_P01021195	chr3:83089315-83108396_F		lincRNA:chr3:83089315-83108396 forward strand	chr3:83105042-83105101	7.01	9.42	0.19
589	A_52_P48767	NM_027760	Rassf8	Mus musculus Ras association (RalGDS/AF-6) domain family (N-terminal) member 8 (Rassf8), mRNA [NM_027760]	chr6:145765937-145765996	6.87	9.28	0.19

590	A_52_P442710	NM_172399	A930038C07Rik	Mus musculus RIKEN cDNA A930038C07 gene (A930038C07Rik), mRNA [NM_172399]	chr6:65655964-65656023	6.48	8.90	0.19
591	A_55_P2072138	XM_001474110	Gm3789	PREDICTED: Mus musculus similar to phosphatidylserine decarboxylase (LOC100045344), mRNA [XM_001474110]	chr14:19875654-19875713	10.07	12.49	0.19
592	A_52_P387724	NM_001079822	Tcf7l1	Mus musculus transcription factor 7-like 1 (T-cell specific, HMG box) (Tcf7l1), transcript variant 1, mRNA [NM_001079822]	chr6:72576436-72576377	7.00	9.42	0.19
593	A_55_P1953391	NM_021477	A2bp1	Mus musculus ataxin 2 binding protein 1 (A2bp1), transcript variant 2, mRNA [NM_021477]	chr16:7411544-7411603	8.75	11.17	0.19
594	A_52_P343856	NM_178661	Creb3l2	Mus musculus cAMP responsive element binding protein 3-like 2 (Creb3l2), mRNA [NM_178661]	chr6:37281267-37281208	6.13	8.56	0.19
595	A_51_P272553	NM_011498	Bhlhe40	Mus musculus basic helix-loop-helix family, member e40 (Bhlhe40), mRNA [NM_011498]	chr6:108616309-108616368	6.09	8.52	0.19
596	A_30_P01030414	chr15:92174226-92201201_R		lincRNA:chr15:92174226-92201201 reverse strand	chr15:92196519-92196460	6.43	8.86	0.19
597	A_55_P2055257	NM_173740	Maoa	Mus musculus monoamine oxidase A (Maoa), nuclear gene encoding mitochondrial protein, mRNA [NM_173740]	chrX:16264785-16264844	8.01	10.44	0.19
598	A_52_P559545	NM_207298	Cercam	Mus musculus cerebral endothelial cell adhesion molecule (Cercam), mRNA [NM_207298]	chr2:29737777-29737836	6.34	8.78	0.19
599	A_51_P324814	NM_010664	Krt18	Mus musculus keratin 18 (Krt18), mRNA [NM_010664]	chr15:101862341-101862400	7.55	9.98	0.19
600	A_30_P01026878	chr7:82902248-82955248_F		lincRNA:chr7:82902248-82955248 forward strand	chr7:82924473-82924532	6.72	9.15	0.19
601	A_55_P2021011	NM_030207	Sfi1	Mus musculus Sfi1 homolog, spindle assembly associated (yeast) (Sfi1), mRNA [NM_030207]	chr11:3086127-3086068	7.60	10.04	0.19
602	A_52_P353417	NM_010299	Gm2a	Mus musculus GM2 ganglioside activator protein (Gm2a), mRNA [NM_010299]	chr11:54923856-54923915	7.70	10.14	0.19
603	A_51_P320304	NM_011122	Plod1	Mus musculus procollagen-lysine, 2-oxoglutarate 5-dioxygenase 1 (Plod1), mRNA [NM_011122]	chr4:147291457-147290037	8.18	10.63	0.18

604	A_51_P234692	NR_003513	Neat1	Mus musculus nuclear paraspeckle assembly transcript 1 (non-protein coding) (Neat1), non-coding RNA [NR_003513]	chr19:5842623-5842564	7.45	9.89	0.18
605	A_55_P2055217	XR_031611	Gm7556	PREDICTED: Mus musculus similar to Aldolase 1, A (LOC665254), misc RNA [XR_031611]	chr6:39793692-39793633	9.28	11.72	0.18
606	A_55_P1992019	NM_001083119	Ptpru	Mus musculus protein tyrosine phosphatase, receptor type, U (Ptpru), transcript variant 2, mRNA [NM_001083119]	chr4:131324432-131324373	8.58	11.02	0.18
607	A_51_P287069	NM_009825	Serpinh1	Mus musculus serine (or cysteine) peptidase inhibitor, clade H, member 1 (Serpinh1), transcript variant 1, mRNA [NM_009825]	chr7:106494030-106493971	9.44	11.89	0.18
608	A_55_P2037689	XR_031802	Gm13549	PREDICTED: Mus musculus similar to lactate dehydrogenase 1, A chain (LOC625354), misc RNA [XR_031802]	chr2:59237555-59237496	6.40	8.86	0.18
609	A_55_P1969276	NM_020259	Hhip	Mus musculus Hedgehog-interacting protein (Hhip), mRNA [NM_020259]	chr8:82494339-82494280	6.50	8.96	0.18
610	A_51_P434670	NM_021881	Qk	Mus musculus quaking (Qk), transcript variant 3, mRNA [NM_021881]	chr17:10403223-10403164	8.72	11.18	0.18
611	A_55_P1957249	NM_001146268	Pdgfrb	Mus musculus platelet derived growth factor receptor, beta polypeptide (Pdgfrb), transcript variant 1, mRNA [NM_001146268]	chr18:61244606-61244665	6.25	8.72	0.18
612	A_51_P334104	NM_007833	Dcn	Mus musculus decorin (Dcn), transcript variant 2, mRNA [NM_007833]	chr10:96980233-96980292	6.53	9.00	0.18
613	A_55_P2167112	NM_145611	Kank2	Mus musculus KN motif and ankyrin repeat domains 2 (Kank2), mRNA [NM_145611]	chr9:21571329-21571270	6.48	8.95	0.18
614	A_30_P01031222	chr6:127111250-127142925_F		lincRNA:chr6:127111250-127142925 forward strand	chr6:127133211-127133270	9.92	12.38	0.18
615	A_52_P183826	NM_178788	Dctd	Mus musculus dCMP deaminase (Dctd), transcript variant 1, mRNA [NM_178788]	chr8:49197399-49222612	8.21	10.68	0.18
616	A_52_P570543	NM_172800	Sdk2	Mus musculus sidekick homolog 2 (chicken) (Sdk2), mRNA [NM_172800]	chr11:113655119-113655060	8.63	11.10	0.18
617	A_55_P2083559	NM_007545	Hrk	Mus musculus harakiri, BCL2 interacting protein (contains only BH3 domain) (Hrk), mRNA [NM_007545]	chr5:118639423-118639482	8.35	10.82	0.18
618	A_55_P2141093	NM_010165	Eya2	Mus musculus eyes absent 2 homolog (Drosophila) (Eya2), mRNA [NM_010165]	chr2:165597072-165597131	7.96	10.43	0.18

619	A_52_P285470	NM_001081088	Lrp2	Mus musculus low density lipoprotein receptor-related protein 2 (Lrp2), mRNA [NM_001081088]	chr2:69262625-69262566	6.68	9.15	0.18
620	A_55_P2012171	NM_026470	Spata6	Mus musculus spermatogenesis associated 6 (Spata6), mRNA [NM_026470]	chr4:111447424-111447483	6.61	9.08	0.18
621	A_55_P2269700	NM_026714	Ccdc163	Mus musculus coiled-coil domain containing 163 (Ccdc163), mRNA [NM_026714]	chr4:116381639-116381698	7.00	9.48	0.18
622	A_52_P306305	NM_001035533	Akap2	Mus musculus A kinase (PRKA) anchor protein 2 (Akap2), transcript variant 1, mRNA [NM_001035533]	chr4:57905932-57905991	7.89	10.37	0.18
623	A_51_P373901	NM_025290	Rsph1	Mus musculus radial spoke head 1 homolog (Chlamydomonas) (Rsph1), mRNA [NM_025290]	chr17:31410515-31410354	6.40	8.88	0.18
624	A_30_P01022773	chr15:92174226-92201201_R		lincRNA:chr15:92174226-92201201 reverse strand	chr15:92196459-92196400	6.82	9.31	0.18
625	A_30_P01018205	chr5:96591892-96606467_R		lincRNA:chr5:96591892-96606467 reverse strand	chr5:96603511-96603452	6.68	9.16	0.18
626	A_51_P290074	NM_021272	Fabp7	Mus musculus fatty acid binding protein 7, brain (Fabp7), mRNA [NM_021272]	chr10:57508039-57508098	10.87	13.36	0.18
627	A_55_P2046262	NM_016966	Phgdh	Mus musculus 3-phosphoglycerate dehydrogenase (Phgdh), mRNA [NM_016966]	chr3:98117274-98117215	7.47	9.96	0.18
628	A_55_P1954221	NM_010128	Emp1	Mus musculus epithelial membrane protein 1 (Emp1), mRNA [NM_010128]	chr6:135333061-135333120	6.46	8.96	0.18
629	A_55_P2154387	NM_007554	Bmp4	Mus musculus bone morphogenetic protein 4 (Bmp4), mRNA [NM_007554]	chr14:47003450-47003391	8.00	10.50	0.18
630	A_66_P133328	NM_001025261	Tpd52	Mus musculus tumor protein D52 (Tpd52), transcript variant 1, mRNA [NM_001025261]	chr3:8929759-8929700	9.22	11.72	0.18
631	A_55_P2219982	NM_008265	Hoxa4	Mus musculus homeobox A4 (Hoxa4), mRNA [NM_008265]	chr6:52140346-52140287	5.99	8.49	0.18
632	A_55_P1979650	NM_011309	S100a1	Mus musculus S100 calcium binding protein A1 (S100a1), mRNA [NM_011309]	chr3:90315018-90314959	5.49	7.99	0.18
633	A_66_P111562	NM_007631	Ccnd1	Mus musculus cyclin D1 (Ccnd1), mRNA [NM_007631]	chr7:152116261-152116202	9.95	12.45	0.18
634	A_55_P2024669	NM_001039546	Myo6	Mus musculus myosin VI (Myo6), mRNA [NM_001039546]	chr9:80155567-80155626	6.72	9.23	0.18
635	A_30_P01020665	chr18:69923410-69965360_R		lincRNA:chr18:69923410-69965360 reverse strand	chr18:69926747-69926688	5.51	8.02	0.18

636	A_55_P1968370	NM_001110337	Gprc5c	Mus musculus G protein-coupled receptor, family C, group 5, member C (Gprc5c), transcript variant 1, mRNA [NM_001110337]	chr11:114733870-114733929	6.46	8.98	0.17
637	A_30_P01032108	chr14:55433382-55452757_F		lincRNA:chr14:55433382-55452757 forward strand	chr14:55445400-55445459	8.39	10.91	0.17
638	A_55_P2294074	CB196702	AA536887	AGENCOURT_11258496 NIH_MGC_135 Mus musculus cDNA clone IMAGE:30135500 5', mRNA sequence [CB196702]	chr7:144478538-144478479	5.82	8.34	0.17
639	A_30_P01022940	chr6:127111250-127142925_F		lincRNA:chr6:127111250-127142925 forward strand	chr6:127133527-127133586	11.48	14.00	0.17
640	A_52_P99411	NM_008410	Itm2b	Mus musculus integral membrane protein 2B (Itm2b), mRNA [NM_008410]	chr14:73762892-73762833	9.13	11.66	0.17
641	A_55_P2145696	NM_176830	1110036O03Rik	Mus musculus RIKEN cDNA 1110036O03 gene (1110036O03Rik), mRNA [NM_176830]	chr11:100270122-100270063	8.61	11.15	0.17
642	A_51_P497395	NM_145100	Lypd1	Mus musculus Ly6/Plaur domain containing 1 (Lypd1), mRNA [NM_145100]	chr1:127768698-127768639	7.96	10.49	0.17
643	A_55_P2109326	NM_009599	Ache	Mus musculus acetylcholinesterase (Ache), mRNA [NM_009599]	chr5:137733122-137733181	8.58	11.12	0.17
644	A_55_P2123902	NM_178777	Nhlh2	Mus musculus nescient helix loop helix 2 (Nhlh2), mRNA [NM_178777]	chr3:101818350-101818409	8.10	10.64	0.17
645	A_30_P01032863	chr16:46384974-46391331_F		lincRNA:chr16:46384974-46391331 forward strand	chr16:46388941-46389000	6.92	9.47	0.17
646	A_55_P2143251	ENSMUST00000105034		Putative uncharacterized protein [Source:UniProtKB/TrEMBL;Acc:Q3TQL3] [ENSMUST00000105034]	chr4:120569114-120569173	8.27	10.82	0.17
647	A_51_P246224	NM_177409	Tram2	Mus musculus translocating chain-associating membrane protein 2 (Tram2), transcript variant 2, mRNA [NM_177409]	chr1:20991548-20991489	5.76	8.31	0.17
648	A_30_P01022300	chr2:59235155-59242518_F		lincRNA:chr2:59235155-59242518 forward strand	chr2:59237280-59237339	10.74	13.30	0.17
649	A_55_P2027421	NM_177838	Fam163a	Mus musculus family with sequence similarity 163, member A (Fam163a), mRNA [NM_177838]	chr1:157923165-157923106	5.63	8.19	0.17
650	A_55_P1991770	NM_019417	Pdlim4	Mus musculus PDZ and LIM domain 4 (Pdlim4), mRNA [NM_019417]	chr11:53868493-53868434	8.77	11.33	0.17
651	A_55_P2120080	ENSMUST00000110633		zinc finger, MYND domain containing 11 Gene [Source:MGI (curated);Acc:MGI:1913755] [ENSMUST00000110633]	chr13:9689645-9689586	10.00	12.56	0.17

652	A_55_P2328445	AK044281	A930005G22Rik	Mus musculus adult retina cDNA, RIKEN full-length enriched library, clone:A930005G22 product:unclassifiable, full insert sequence. [AK044281]	chr5:115870695-115870636	7.78	10.35	0.17
653	A_55_P2011385	XM_916287	LOC637082	PREDICTED: Mus musculus similar to TIFA, transcript variant 1 (LOC637082), mRNA [XM_916287]	chr3:127501256-127501315	6.01	8.58	0.17
654	A_30_P01027457	chr1:34734249-34786649_R		lincRNA:chr1:34734249-34786649 reverse strand	chr1:34780727-34780668	8.59	11.16	0.17
655	A_66_P135391	NM_008342	Igfbp2	Mus musculus insulin-like growth factor binding protein 2 (Igfbp2), mRNA [NM_008342]	chr1:72898813-72898872	10.37	12.95	0.17
656	A_51_P147123	NM_008744	Ntn1	Mus musculus netrin 1 (Ntn1), mRNA [NM_008744]	chr11:68040112-68040053	7.52	10.10	0.17
657	A_55_P2019312	NM_178396	Car12	Mus musculus carbonic anyhydrase 12 (Car12), mRNA [NM_178396]	chr9:66614542-66614601	5.86	8.43	0.17
658	A_30_P01021631	NEAT1		lincRNA:chr19:5842306-5845476 reverse strand	chr19:5842593-5842534	6.76	9.34	0.17
659	A_55_P2429225	NM_001190161	Psrc1	Mus musculus proline/serine-rich coiled-coil 1 (Psrc1), transcript variant 1, mRNA [NM_001190161]	chr3:108190744-108190803	7.03	9.61	0.17
660	A_30_P01029584	chr6:90915489-90940052_R		lincRNA:chr6:90915489-90940052 reverse strand	chr6:90938148-90938089	10.19	12.78	0.17
661	A_55_P2047809	NM_175692	Snhg11	Mus musculus small nucleolar RNA host gene 11 (non-protein coding) (Snhg11), mRNA [NM_175692]	chr2:158206508-158206567	7.53	10.12	0.17
662	A_52_P409833	NM_008872	Plat	Mus musculus plasminogen activator, tissue (Plat), mRNA [NM_008872]	chr8:23892531-23892590	8.86	11.45	0.17
663	A_51_P413910	NM_008257	Hmx3	Mus musculus H6 homeobox 3 (Hmx3), mRNA [NM_008257]	chr7:138688330-138688389	6.34	8.94	0.17
664	A_30_P01020135	NEAT1		lincRNA:chr19:5842306-5845476 reverse strand	chr19:5842653-5842594	7.41	10.01	0.17
665	A_55_P2105371	NM_010052	Dlk1	Mus musculus delta-like 1 homolog (Drosophila) (Dlk1), transcript variant 1, mRNA [NM_010052]	chr12:110698502-110698561	8.79	11.38	0.17
666	A_30_P01032788	chr8:94841965-94881130_R		lincRNA:chr8:94841965-94881130 reverse strand	chr8:94850317-94850258	5.57	8.17	0.17
667	A_51_P479408	NM_008317	Hyal1	Mus musculus hyaluronoglucosaminidase 1 (Hyal1), mRNA [NM_008317]	chr9:107481893-107481952	6.24	8.83	0.17

668	A_55_P2235911	AK075572	0610009E02Rik	Mus musculus adult male kidney cDNA, RIKEN full-length enriched library, clone:0610009E02 product:unclassifiable, full insert sequence. [AK075572]	chr2:26314780-26314839	8.74	11.34	0.17
669	A_30_P01017453	chr15:92174262-92196074_F		lincRNA:chr15:92174262-92196074 forward strand	chr15:92195960-92196019	6.61	9.21	0.17
670	A_66_P112573	NM_020583	Isg20	Mus musculus interferon-stimulated protein (Isg20), transcript variant 1, mRNA [NM_020583]	chr7:86065136-86065195	6.64	9.25	0.17
671	A_51_P444645	NM_010701	Lect1	Mus musculus leukocyte cell derived chemotaxin 1 (Lect1), mRNA [NM_010701]	chr14:80050133-80045279	5.59	8.20	0.16
672	A_55_P2069597	NM_028841	Tspan17	Mus musculus tetraspanin 17 (Tspan17), mRNA [NM_028841]	chr13:54897519-54897578	7.97	10.57	0.16
673	A_51_P307316	NM_027875	Syde1	Mus musculus synapse defective 1, Rho GTPase, homolog 1 (C. elegans) (Syde1), mRNA [NM_027875]	chr10:78047421-78047362	7.66	10.27	0.16
674	A_55_P1999972	XM_001475932	Gm3187	PREDICTED: Mus musculus similar to 1700001E04Rik protein (LOC100041184), mRNA [XM_001475932]	chr14:6772278-6772337	10.90	13.51	0.16
675	A_55_P1979341	NM_007806	Cyba	Mus musculus cytochrome b-245, alpha polypeptide (Cyba), mRNA [NM_007806]	chr8:124948785-124948726	7.17	9.78	0.16
676	A_55_P2104487	NM_001164056	Pld1	Mus musculus phospholipase D1 (Pld1), transcript variant 1, mRNA [NM_001164056]	chr3:28032066-28032125	5.51	8.12	0.16
677	A_55_P2030160	NM_001142920	Tcf7l2	Mus musculus transcription factor 7-like 2, T-cell specific, HMG-box (Tcf7l2), transcript variant 4, mRNA [NM_001142920]	chr19:55994029-55994088	7.14	9.75	0.16
678	A_52_P53906	NM_009829	Ccnd2	Mus musculus cyclin D2 (Ccnd2), mRNA [NM_009829]	chr6:127098755-127098696	9.82	12.43	0.16
679	A_51_P240019	NM_013755	Gyg	Mus musculus glycogenin (Gyg), mRNA [NM_013755]	chr3:20022077-20022018	7.57	10.18	0.16
680	A_55_P1967443	XR_034924	Gm5207	PREDICTED: Mus musculus similar to Phosphoglycerate dehydrogenase (LOC382931), misc RNA [XR_034924]	chr14:95820521-95820580	7.09	9.71	0.16
681	A_51_P433824	NM_009415	Tpi1	Mus musculus triosephosphate isomerase 1 (Tpi1), mRNA [NM_009415]	chr6:124761006-124760947	10.11	12.73	0.16
682	A_51_P315904	NM_011817	Gadd45g	Mus musculus growth arrest and DNA-damage-inducible 45 gamma (Gadd45g), mRNA [NM_011817]	chr13:51943733-51943792	9.36	11.98	0.16

683	A_55_P1953533	ENSMUST00000084696		spondin 1, (f-spondin) extracellular matrix protein Gene [Source:MGI (curated);Acc:MGI:2385287] [ENSMUST00000084696]	chr7:121073331-121073390	7.48	10.11	0.16
684	A_55_P2052290	NM_177420	Psat1	Mus musculus phosphoserine aminotransferase 1 (Psat1), mRNA [NM_177420]	chr19:15979681-15979622	9.97	12.60	0.16
685	A_55_P1967885	NM_008173	Nr3c1	Mus musculus nuclear receptor subfamily 3, group C, member 1 (Nr3c1), mRNA [NM_008173]	chr18:39571700-39571641	7.11	9.74	0.16
686	A_55_P2115318	NM_174993	Fmr1nb	Mus musculus fragile X mental retardation 1 neighbor (Fmr1nb), transcript variant 1, mRNA [NM_174993]	chrX:66056806-66056865	6.53	9.17	0.16
687	A_52_P515094	NM_025326	Tmem176a	Mus musculus transmembrane protein 176A (Tmem176a), transcript variant 1, mRNA [NM_025326]	chr6:48794048-48794107	8.35	10.98	0.16
688	A_52_P53019	NM_139228	Rhbdl3	Mus musculus rhomboid, veinlet-like 3 (Drosophila) (Rhbdl3), mRNA [NM_139228]	chr11:80169190-80169249	7.03	9.67	0.16
689	A_30_P01023186	chr6:52046805-52072744_R		lincRNA:chr6:52046805-52072744 reverse strand	chr6:52053020-52052961	6.56	9.20	0.16
690	A_51_P184300	NM_010087	Dtna	Mus musculus dystrobrevin alpha (Dtna), transcript variant 2, mRNA [NM_010087]	chr18:23748714-23748773	8.80	11.45	0.16
691	A_30_P01023928	lincP21		lincRNA:chr17:29194418-29215906 reverse strand	chr17:29194800-29194741	6.11	8.75	0.16
692	A_30_P01025936	chr8:124355710-124401160_R		lincRNA:chr8:124355710-124401160 reverse strand	chr8:124397661-124397602	6.34	8.99	0.16
693	A_52_P130787	NM_013568	Kcna6	Mus musculus potassium voltage-gated channel, shaker-related, subfamily, member 6 (Kcna6), mRNA [NM_013568]	chr6:126658483-126658424	9.09	11.73	0.16
694	A_55_P1998601	NM_183161	Slc17a9	Mus musculus solute carrier family 17, member 9 (Slc17a9), mRNA [NM_183161]	chr2:180476906-180476965	6.85	9.50	0.16
695	A_55_P1981381	NM_010683	Lamc1	Mus musculus laminin, gamma 1 (Lamc1), mRNA [NM_010683]	chr1:155066215-155066156	7.01	9.66	0.16
696	A_55_P2168667	NM_028030	Rbpms2	Mus musculus RNA binding protein with multiple splicing 2 (Rbpms2), mRNA [NM_028030]	chr9:65508259-65508318	8.41	11.06	0.16
697	A_55_P2129972	XR_035510	Gm5276	PREDICTED: Mus musculus similar to fructose-1,6-bisphosphate aldolase A (LOC383862), misc RNA [XR_035510]	chr3:57167204-57167263	10.18	12.83	0.16

698	A_66_P108723	ENSMUST00000094634		Testis protein TEX17 [Source:UniProtKB/TrEMBL;Acc:Q99MW2] [ENSMUST00000094634]	chr4:133560238-133560297	7.25	9.91	0.16
699	A_51_P420859	NM_011773	Slc30a3	Mus musculus solute carrier family 30 (zinc transporter), member 3 (Slc30a3), mRNA [NM_011773]	chr5:31390447-31390388	6.22	8.88	0.16
700	A_55_P1996716	NM_198037	Cachd1	Mus musculus cache domain containing 1 (Cachd1), mRNA [NM_198037]	chr4:100676283-100676342	8.79	11.45	0.16
701	A_30_P01025010	chr6:127111250-127142925_F		lincRNA:chr6:127111250-127142925 forward strand	chr6:127133442-127133501	11.80	14.46	0.16
702	A_51_P248122	NM_133234	Bbc3	Mus musculus BCL2 binding component 3 (Bbc3), mRNA [NM_133234]	chr7:16903403-16903462	8.95	11.62	0.16
703	A_51_P378856	NM_019703	Pfkp	Mus musculus phosphofructokinase, platelet (Pfkp), mRNA [NM_019703]	chr13:6583872-6581718	8.70	11.36	0.16
704	A_51_P403564	NM_008499	Lhx5	Mus musculus LIM homeobox protein 5 (Lhx5), mRNA [NM_008499]	chr5:120891144-120891203	6.01	8.68	0.16
705	A_51_P204080	NM_013820	Hk2	Mus musculus hexokinase 2 (Hk2), mRNA [NM_013820]	chr6:82675115-82675056	6.30	8.98	0.16
706	A_55_P2369191	NM_010283	Ggta1	Mus musculus glycoprotein galactosyltransferase alpha 1, 3 (Ggta1), transcript variant 1, mRNA [NM_010283]	chr2:35257682-35257623	6.15	8.83	0.16
707	A_30_P01025511	chr4:149445201-149445902_F		lincRNA:chr4:149445201-149445902 forward strand	chr4:149445831-149445890	6.11	8.79	0.16
708	A_30_P01022871	chr16:46384974-46391331_F		lincRNA:chr16:46384974-46391331 forward strand	chr16:46389526-46389585	6.97	9.66	0.16
709	A_51_P463765	NM_011595	Timp3	Mus musculus tissue inhibitor of metalloproteinase 3 (Timp3), mRNA [NM_011595]	chr10:85811696-85811755	7.48	10.17	0.16
710	A_51_P258493	NM_011067	Per3	Mus musculus period homolog 3 (Drosophila) (Per3), mRNA [NM_011067]	chr4:150379315-150379256	7.33	10.02	0.16
711	A_51_P113906	NM_025467	Gkn2	Mus musculus gastrokine 2 (Gkn2), mRNA [NM_025467]	chr6:87329285-87329344	5.68	8.37	0.15
712	A_30_P01024217	chr18:5162836-5165729_R		lincRNA:chr18:5162836-5165729 reverse strand	chr18:5163016-5162957	7.54	10.23	0.15
713	A_66_P128079	XR_004919	Gm5604	PREDICTED: Mus musculus similar to Keratin 8 (LOC675884), misc RNA [XR_004919]	chr7:24143775-24143832	6.33	9.03	0.15
714	A_55_P2006255	NM_001009935	Txnip	Mus musculus thioredoxin interacting protein (Txnip), transcript variant 1, mRNA [NM_001009935]	chr3:96364206-96364265	8.61	11.32	0.15

715	A_55_P2143517	NM_008047	Fstl1	Mus musculus follistatin-like 1 (Fstl1), mRNA [NM_008047]	chr16:37836492-37836551	8.37	11.08	0.15
716	A_52_P552550	NM_009642	Agtrap	Mus musculus angiotensin II, type I receptor-associated protein (Agtrap), mRNA [NM_009642]	chr4:147454490-147454431	6.55	9.25	0.15
717	A_30_P01030598	chr16:46384974-46391331_R		lincRNA:chr16:46384974-46391331 reverse strand	chr16:46388308-46388249	6.15	8.86	0.15
718	A_55_P1988424	NM_009415	Tpi1	Mus musculus triosephosphate isomerase 1 (Tpi1), mRNA [NM_009415]	chr6:124761982-124761923	11.21	13.92	0.15
719	A_51_P129012	NM_009735	B2m	Mus musculus beta-2 microglobulin (B2m), mRNA [NM_009735]	chr2:121978401-121978460	6.75	9.46	0.15
720	A_55_P2074631	NM_007755	Cpeb1	Mus musculus cytoplasmic polyadenylation element binding protein 1 (Cpeb1), mRNA [NM_007755]	chr7:88492396-88492337	7.59	10.30	0.15
721	A_52_P298002	NM_008102	Gch1	Mus musculus GTP cyclohydrolase 1 (Gch1), mRNA [NM_008102]	chr14:47773856-47773797	5.90	8.62	0.15
722	A_52_P470316	NM_033521	Laptm4b	Mus musculus lysosomal-associated protein transmembrane 4B (Laptm4b), mRNA [NM_033521]	chr15:34213600-34213659	7.21	9.92	0.15
723	A_51_P253984	NM_008791	Pcp4	Mus musculus Purkinje cell protein 4 (Pcp4), mRNA [NM_008791]	chr16:96747241-96747300	8.39	11.10	0.15
724	A_30_P01019037	chr19:5834117-5835940_R		lincRNA:chr19:5834117-5835940 reverse strand	chr19:5834602-5834543	6.63	9.35	0.15
725	A_52_P72434	NM_008439	Khk	Mus musculus ketohexokinase (Khk), mRNA [NM_008439]	chr5:31233555-31233614	7.87	10.58	0.15
726	A_51_P420400	NM_010703	Lef1	Mus musculus lymphoid enhancer binding factor 1 (Lef1), mRNA [NM_010703]	chr3:130907851-130926012	5.76	8.48	0.15
727	A_55_P2074656	NM_008812	Padi2	Mus musculus peptidyl arginine deiminase, type II (Padi2), mRNA [NM_008812]	chr4:140508436-140508495	6.84	9.56	0.15
728	A_55_P2344753	AK044369	A930009L07Rik	Mus musculus adult retina cDNA, RIKEN full-length enriched library, clone:A930009L07 product:unclassifiable, full insert sequence. [AK044369]	chr15:72338337-72338278	6.59	9.31	0.15
729	A_66_P136714	BC021399	Scpep1	Mus musculus serine carboxypeptidase 1, mRNA (cDNA clone MGC:29291 IMAGE:4193674), complete cds. [BC021399]	chr11:88785441-88785382	8.17	10.90	0.15
730	A_55_P1988481	XR_033805	Gm8865	PREDICTED: Mus musculus similar to interferon-inducible protein 16 (LOC667893), misc RNA [XR_033805]	chr14:55445131-55445072	7.25	9.97	0.15

731	A_55_P1955548	NM_009510	Ezr	Mus musculus ezrin (Ezr), mRNA [NM_009510]	chr17:6942666-6942607	7.41	10.14	0.15
732	A_55_P2003034	NM_177161	P4ha3	Mus musculus procollagen-proline, 2-oxoglutarate 4-dioxygenase (proline 4-hydroxylase), alpha polypeptide III (P4ha3), mRNA [NM_177161]	chr7:107467431-107467490	7.34	10.07	0.15
733	A_55_P2137527	NM_029283	Fam183b	Mus musculus family with sequence similarity 183, member B (Fam183b), transcript variant 1, mRNA [NM_029283]	chr11:58606416-58606357	7.05	9.78	0.15
734	A_51_P438200	NM_030749	Sil1	Mus musculus endoplasmic reticulum chaperone SIL1 homolog (S. cerevisiae) (Sil1), mRNA [NM_030749]	chr18:35426149-35426090	8.70	11.43	0.15
735	A_51_P426754	NM_009673	Anxa5	Mus musculus annexin A5 (Anxa5), mRNA [NM_009673]	chr3:36348124-36348065	7.27	10.01	0.15
736	A_52_P641132	NM_011440	Sox14	Mus musculus SRY-box containing gene 14 (Sox14), mRNA [NM_011440]	chr9:99775688-99775629	6.66	9.40	0.15
737	A_55_P2006118	NM_001159487	Rbp4	Mus musculus retinol binding protein 4, plasma (Rbp4), transcript variant 1, mRNA [NM_001159487]	chr19:38191179-38191120	6.01	8.75	0.15
738	A_66_P126323	ENSMUST00000114915		RIKEN cDNA 9230102O04 gene Gene [Source:MGI Symbol;Acc:MGI:1924932] [ENSMUST00000114915]	chr2:9804803-9804744	7.07	9.82	0.15
739	A_66_P118600	NM_008480	Lama1	Mus musculus laminin, alpha 1 (Lama1), mRNA [NM_008480]	chr17:68171924-68171983	7.92	10.67	0.15
740	A_52_P630867	NM_001033336	Abcc4	Mus musculus ATP-binding cassette, sub-family C (CFTR/MRP), member 4 (Abcc4), transcript variant 1, mRNA [NM_001033336]	chr14:118882163-118882104	7.76	10.51	0.15
741	A_55_P1985768	NM_053273	Ttyh2	Mus musculus tweety homolog 2 (Drosophila) (Ttyh2), mRNA [NM_053273]	chr11:114582192-114582251	6.84	9.58	0.15
742	A_30_P01032267	chr16:46384974-46391331_R		lincRNA:chr16:46384974-46391331 reverse strand	chr16:46388117-46388058	6.80	9.55	0.15
743	A_55_P1965456	NM_008262	Onecut1	Mus musculus one cut domain, family member 1 (Onecut1), mRNA [NM_008262]	chr9:74710921-74710980	6.15	8.90	0.15
744	A_55_P2030433	NM_008155	Gpi1	Mus musculus glucose phosphate isomerase 1 (Gpi1), mRNA [NM_008155]	chr7:34987233-34987174	10.86	13.61	0.15
745	A_30_P01020604	chr19:5771425-5848475_F		lincRNA:chr19:5771425-5848475 forward strand	chr19:5842428-5842487	7.71	10.47	0.15
746	A_30_P01027299	chr8:94849929-94870274_R		lincRNA:chr8:94849929-94870274 reverse strand	chr8:94850306-94850247	5.36	8.12	0.15

747	A_66_P130887	NM_130448	Pcdh18	Mus musculus protocadherin 18 (Pcdh18), mRNA [NM_130448]	chr3:49547364-49547305	7.87	10.64	0.15
748	A_55_P1982563	NM_010117	Rhbdf1	Mus musculus rhomboid family 1 (Drosophila) (Rhbdf1), mRNA [NM_010117]	chr11:32109646-32109587	6.76	9.53	0.15
749	A_55_P1958379	NM_015748	Slit1	Mus musculus slit homolog 1 (Drosophila) (Slit1), mRNA [NM_015748]	chr19:41674808-41674749	7.76	10.54	0.15
750	A_66_P126332	NM_001110508	Zfp703	Mus musculus zinc finger protein 703 (Zfp703), transcript variant 2, mRNA [NM_001110508]	chr8:28091863-28091922	9.05	11.83	0.15
751	A_51_P135268	NM_016869	Corin	Mus musculus corin (Corin), transcript variant 1, mRNA [NM_016869]	chr5:72692347-72692288	5.78	8.56	0.15
752	A_55_P2425938	AK079380	2810468N07Rik	Mus musculus 16 days neonate cerebellum cDNA, RIKEN full-length enriched library, clone:9630053J03 product:unclassifiable, full insert sequence. [AK079380]	chr17:25711920-25711979	6.46	9.24	0.15
753	A_55_P2013356	NM_023132	Renbp	Mus musculus renin binding protein (Renbp), transcript variant 1, mRNA [NM_023132]	chrX:71175594-71175535	6.39	9.18	0.15
754	A_30_P01027443	chr16:46384974-46391331_F		lincRNA:chr16:46384974-46391331 forward strand	chr16:46388702-46388761	7.01	9.80	0.15
755	A_52_P249424	NM_001025257	Vegfa	Mus musculus vascular endothelial growth factor A (Vegfa), transcript variant 3, mRNA [NM_001025257]	chr17:46162435-46162376	9.08	11.87	0.14
756	A_52_P489295	NM_009621	Adamts1	Mus musculus a disintegrin-like and metallopeptidase (reprolysin type) with thrombospondin type 1 motif, 1 (Adamts1), mRNA [NM_009621]	chr16:85795103-85795044	7.57	10.37	0.14
757	A_55_P1973254	NM_133978	Cmtm7	Mus musculus CKLF-like MARVEL transmembrane domain containing 7 (Cmtm7), mRNA [NM_133978]	chr9:114666105-114666046	6.31	9.12	0.14
758	A_55_P2097964	XM_001481064	9030622O22Rik	PREDICTED: Mus musculus hypothetical protein LOC100048732 (LOC100048732), mRNA [XM_001481064]	chr2:147814595-147814536	6.22	9.02	0.14
759	A_52_P357829	NM_001081125	Gli2	Mus musculus GLI-Kruppel family member GLI2 (Gli2), mRNA [NM_001081125]	chr1:120731314-120731255	6.21	9.01	0.14
760	A_55_P2046393	NM_026555	Rcn3	Mus musculus reticulocalbin 3, EF-hand calcium binding domain (Rcn3), mRNA [NM_026555]	chr7:52338358-52338299	7.02	9.83	0.14
761	A_51_P131800	NM_007806	Cyba	Mus musculus cytochrome b-245, alpha polypeptide (Cyba), mRNA [NM_007806]	chr8:124951161-124950893	6.94	9.74	0.14

762	A_55_P1979645	NM_011309	S100a1	Mus musculus S100 calcium binding protein A1 (S100a1), mRNA [NM_011309]	chr3:90315250-90315191	6.76	9.57	0.14
763	A_30_P01031724	chr7:48794017-48815817_F		lincRNA:chr7:48794017-48815817 forward strand	chr7:48802100-48802159	5.49	8.30	0.14
764	A_55_P2022434	NM_008155	Gpi1	Mus musculus glucose phosphate isomerase 1 (Gpi1), mRNA [NM_008155]	chr7:34987237-34987178	11.82	14.64	0.14
765	A_30_P01028912	chr16:46384974-46391331_R		lincRNA:chr16:46384974-46391331 reverse strand	chr16:46387920-46387861	7.20	10.03	0.14
766	A_30_P01029054	chr8:122712014-122732520_R		lincRNA:chr8:122712014-122732520 reverse strand	chr8:122724409-122724350	6.41	9.24	0.14
767	A_30_P01025818	chr19:5834117-5835940_R		lincRNA:chr19:5834117-5835940 reverse strand	chr19:5834662-5834603	6.96	9.79	0.14
768	A_30_P01029839	chr3:36280098-36347798_F		lincRNA:chr3:36280098-36347798 forward strand	chr3:36347443-36347502	5.97	8.80	0.14
769	A_55_P1961014	NM_009150	Selenbp1	Mus musculus selenium binding protein 1 (Selenbp1), mRNA [NM_009150]	chr3:94748621-94748680	6.30	9.13	0.14
770	A_52_P31543	NM_007570	Btg2	Mus musculus B-cell translocation gene 2, anti-proliferative (Btg2), mRNA [NM_007570]	chr1:135972153-135972094	8.05	10.88	0.14
771	A_51_P159453	NM_009252	Serpina3n	Mus musculus serine (or cysteine) peptidase inhibitor, clade A, member 3N (Serpina3n), mRNA [NM_009252]	chr12:105651918-105651977	5.72	8.55	0.14
772	A_55_P2038358	NM_012006	Acot1	Mus musculus acyl-CoA thioesterase 1 (Acot1), mRNA [NM_012006]	chr12:85358561-85358620	9.00	11.83	0.14
773	A_52_P354744	NM_011401	Slc2a3	Mus musculus solute carrier family 2 (facilitated glucose transporter), member 3 (Slc2a3), mRNA [NM_011401]	chr6:122678134-122678075	8.21	11.05	0.14
774	A_51_P415688	NM_025278	Gng12	Mus musculus guanine nucleotide binding protein (G protein), gamma 12 (Gng12), transcript variant 2, mRNA [NM_025278]	chr6:66967776-66967835	6.66	9.50	0.14
775	A_55_P2199717	AK033854	9330102E08Rik	Mus musculus adult male diencephalon cDNA, RIKEN full-length enriched library, clone:9330102E08 product:unclassifiable, full insert sequence. [AK033854]	chr6:128119782-128119723	6.70	9.54	0.14
776	A_55_P2020054	AK164572	2610035D17Rik	Mus musculus 13 days embryo lung cDNA, RIKEN full-length enriched library, clone:D430003N19 product:hypothetical protein, full insert sequence. [AK164572]	chr11:113061839-113061780	8.65	11.49	0.14

777	A_55_P2111508	NM_021324	Ttyh1	Mus musculus tweety homolog 1 (Drosophila) (Ttyh1), transcript variant 2, mRNA [NM_021324]	chr7:4086343-4086402	9.41	12.25	0.14
778	A_30_P01029977	chr5:96591892-96606467_R		lincRNA:chr5:96591892-96606467 reverse strand	chr5:96603112-96603053	8.21	11.05	0.14
779	A_30_P01026296	chr14:55433382-55452757_R		lincRNA:chr14:55433382-55452757 reverse strand	chr14:55445008-55444949	7.46	10.30	0.14
780	A_30_P01021563	chr16:46384974-46391331_R		lincRNA:chr16:46384974-46391331 reverse strand	chr16:46388188-46388129	6.84	9.68	0.14
781	A_30_P01018401	chr15:96984422-97000322_F		lincRNA:chr15:96984422-97000322 forward strand	chr15:96991770-96991829	6.25	9.10	0.14
782	A_55_P2111302	NM_007752	Cp	Mus musculus ceruloplasmin (Cp), transcript variant 2, mRNA [NM_007752]	chr3:19889071-19889130	6.15	9.00	0.14
783	A_52_P549827	NM_019946	Mgst1	Mus musculus microsomal glutathione S-transferase 1 (Mgst1), mRNA [NM_019946]	chr6:138104944-138105003	6.15	9.00	0.14
784	A_55_P1993744	NM_019472	Myo10	Mus musculus myosin X (Myo10), mRNA [NM_019472]	chr15:25740245-25740304	7.21	10.07	0.14
785	A_55_P1973995	ENSMUST00000077710		predicted gene 6756 Gene [Source:MGI Symbol;Acc:MGI:3647509] [ENSMUST00000077710]	chr18:37081750-37081809	7.44	10.30	0.14
786	A_55_P2137309	NM_153143	Kctd11	Mus musculus potassium channel tetramerisation domain containing 11 (Kctd11), mRNA [NM_153143]	chr11:69691826-69691767	7.07	9.93	0.14
787	A_55_P1979833	NM_007709	Cited1	Mus musculus Cbp/p300-interacting transactivator with Glu/Asp-rich carboxy-terminal domain 1 (Cited1), mRNA [NM_007709]	chrX:99442835-99442776	8.80	11.65	0.14
788	A_55_P2038007	NM_007791	Csrp1	Mus musculus cysteine and glycine-rich protein 1 (Csrp1), mRNA [NM_007791]	chr1:137648747-137648806	6.76	9.62	0.14
789	A_55_P2131168	NM_029210	Sv2c	Mus musculus synaptic vesicle glycoprotein 2c (Sv2c), mRNA [NM_029210]	chr13:96746045-96745986	7.06	9.93	0.14
790	A_55_P1977473	NM_023118	Dab2	Mus musculus disabled homolog 2 (Drosophila) (Dab2), transcript variant 1, mRNA [NM_023118]	chr15:6390616-6390675	6.31	9.18	0.14
791	A_51_P396570	NM_011961	Plod2	Mus musculus procollagen lysine, 2-oxoglutarate 5-dioxygenase 2 (Plod2), transcript variant 2, mRNA [NM_011961]	chr9:92502483-92502542	7.30	10.17	0.14
792	A_51_P171999	NM_009696	Apoe	Mus musculus apolipoprotein E (Apoe), mRNA [NM_009696]	chr7:20281791-20281732	8.98	11.85	0.14

793	A_51_P307741	NM_009827	Cckar	Mus musculus cholecystokinin A receptor (Cckar), mRNA [NM_009827]	chr5:54090104-54090045	6.61	9.48	0.14
794	A_55_P2065577	ENSMUST00000066038		RIKEN cDNA 6430503K07 gene Gene [Source:MGI Symbol;Acc:MGI:1925318] [ENSMUST00000066038]	chr2:147014084-147014143	6.22	9.10	0.14
795	A_52_P65494	NM_027711	Iqgap2	Mus musculus IQ motif containing GTPase activating protein 2 (Iqgap2), mRNA [NM_027711]	chr13:96398940-96398055	6.46	9.34	0.14
796	A_55_P1953459	NM_001171147	Yap1	Mus musculus yes-associated protein 1 (Yap1), transcript variant 1, mRNA [NM_001171147]	chr9:7932084-7932025	7.46	10.34	0.14
797	A_51_P495232	NM_027127	Gpx8	Mus musculus glutathione peroxidase 8 (putative) (Gpx8), mRNA [NM_027127]	chr13:113833272-113833213	6.46	9.34	0.14
798	A_55_P1984416	XR_001935	Gm8341	PREDICTED: Mus musculus predicted gene, EG666875 (EG666875), misc RNA [XR_001935]	chr15:19384445-19384386	7.38	10.27	0.14
799	A_51_P113178	NM_175398	6530418L21Rik	Mus musculus RIKEN cDNA 6530418L21 gene (6530418L21Rik), transcript variant 1, mRNA [NM_175398]	chr3:105520821-105520880	7.26	10.15	0.14
800	A_55_P2128734	NM_146131	Pbxip1	Mus musculus pre-B-cell leukemia transcription factor interacting protein 1 (Pbxip1), mRNA [NM_146131]	chr3:89252808-89252867	6.66	9.55	0.14
801	A_55_P2178044	NM_010567	Inppl1	Mus musculus inositol polyphosphate phosphatase-like 1 (Inppl1), transcript variant 1, mRNA [NM_010567]	chr7:108971253-108971194	9.44	12.33	0.13
802	A_55_P2031781	NM_027174	Col22a1	Mus musculus collagen, type XXII, alpha 1 (Col22a1), mRNA [NM_027174]	chr15:71628965-71628906	6.19	9.08	0.13
803	A_51_P484500	NM_019938	Pmfbp1	Mus musculus polyamine modulated factor 1 binding protein 1 (Pmfbp1), mRNA [NM_019938]	chr8:112066429-112066488	5.74	8.64	0.13
804	A_55_P2231244	NM_008498	Lhx1	Mus musculus LIM homeobox protein 1 (Lhx1), mRNA [NM_008498]	chr11:84335594-84335442	9.36	12.26	0.13
805	A_55_P1992208	NM_008155	Gpi1	Mus musculus glucose phosphate isomerase 1 (Gpi1), mRNA [NM_008155]	chr7:34987232-34987173	10.86	13.76	0.13
806	A_55_P2067947	XM_983385	Dppa5b	PREDICTED: Mus musculus similar to developmental pluripotency associated 5 (LOC666367), mRNA [XM_983385]	chr9:78120096-78120155	6.24	9.14	0.13
807	A_55_P2069602	NM_001024702	C1ql4	Mus musculus complement component 1, q subcomponent-like 4 (C1ql4), mRNA [NM_001024702]	chr15:98915244-98915185	6.81	9.71	0.13

808	A_55_P2152872	NM_054071	Fgfrl1	Mus musculus fibroblast growth factor receptor-like 1 (Fgfrl1), transcript variant 1, mRNA [NM_054071]	chr5:109135855-109135914	7.84	10.74	0.13
809	A_55_P1997061	XR_033957	Gm5452	PREDICTED: Mus musculus similar to lactate dehydrogenase 1, A chain (LOC432789), misc RNA [XR_033957]	chr13:83973142-83973201	10.05	12.95	0.13
810	A_55_P1954302	NM_001159500	Esrrb	Mus musculus estrogen related receptor, beta (Esrrb), transcript variant 2, mRNA [NM_001159500]	chr12:87862509-87862568	6.19	9.09	0.13
811	A_55_P2106175	NM_008102	Gch1	Mus musculus GTP cyclohydrolase 1 (Gch1), mRNA [NM_008102]	chr14:47775407-47775348	6.61	9.52	0.13
812	A_55_P2168781	XR_031340	Gm7997	PREDICTED: Mus musculus similar to lactate dehydrogenase 1, A chain (LOC666237), misc RNA [XR_031340]	chr8:83295453-83295512	8.23	11.15	0.13
813	A_55_P2124791	NM_001109991	Col18a1	Mus musculus collagen, type XVIII, alpha 1 (Col18a1), transcript variant 1, mRNA [NM_001109991]	chr10:76515849-76515790	6.03	8.95	0.13
814	A_51_P246317	NM_008630	Mt2	Mus musculus metallothionein 2 (Mt2), mRNA [NM_008630]	chr8:96697371-96697430	10.21	13.14	0.13
815	A_55_P1988384	NM_007515	Slc7a3	Mus musculus solute carrier family 7 (cationic amino acid transporter, y+ system), member 3 (Slc7a3), mRNA [NM_007515]	chrX:98274703-98274644	7.18	10.11	0.13
816	A_52_P278354	NM_007557	Bmp7	Mus musculus bone morphogenetic protein 7 (Bmp7), mRNA [NM_007557]	chr2:172695262-172695203	6.48	9.42	0.13
817	A_55_P2059323	NR_028497	Gm13315	Mus musculus predicted gene 13315 (Gm13315), non-coding RNA [NR_028497]	chr2:14643230-14643289	7.53	10.47	0.13
818	A_52_P502577	NM_010101	S1pr3	Mus musculus sphingosine-1-phosphate receptor 3 (S1pr3), mRNA [NM_010101]	chr13:51517079-51517138	6.95	9.90	0.13
819	A_52_P220879	NM_009373	Tgm2	Mus musculus transglutaminase 2, C polypeptide (Tgm2), mRNA [NM_009373]	chr2:157942334-157942275	5.90	8.86	0.13
820	A_55_P2032147	NM_139298	Wnt9a	Mus musculus wingless-type MMTV integration site 9A (Wnt9a), mRNA [NM_139298]	chr11:59146953-59147012	6.22	9.17	0.13
821	A_55_P2165869	NM_009883	Cebpb	Mus musculus CCAAT/enhancer binding protein (C/EBP), beta (Cebpb), mRNA [NM_009883]	chr2:167515828-167515887	5.57	8.53	0.13
822	A_52_P350750	NM_015730	Chrna4	Mus musculus cholinergic receptor, nicotinic, alpha polypeptide 4 (Chrna4), mRNA [NM_015730]	chr2:180757368-180757309	10.02	12.98	0.13

823	A_51_P269084	NM_175329	Chchd10	Mus musculus coiled-coil-helix-coiled-coil-helix domain containing 10 (Chchd10), mRNA [NM_175329]	chr10:75400362-75400421	8.04	11.00	0.13
824	A_51_P389539	NM_054053	Gpr98	Mus musculus G protein-coupled receptor 98 (Gpr98), mRNA [NM_054053]	chr13:81234389-81234330	6.77	9.73	0.13
825	A_55_P2008061	NM_019923	Itpr2	Mus musculus inositol 1,4,5-triphosphate receptor 2 (Itpr2), transcript variant 1, mRNA [NM_019923]	chr6:146060064-146060005	5.99	8.95	0.13
826	A_55_P1962747	NM_207105	H2-Ab1	Mus musculus histocompatibility 2, class II antigen A, beta 1 (H2-Ab1), mRNA [NM_207105]	chr17:34406296-34406355	6.40	9.37	0.13
827	A_66_P119155	ENSMUST00000107172	Dusp3	dual specificity phosphatase 3 (vaccinia virus phosphatase VH1-related) Gene [Source:MGI (curated);Acc:MGI:1919599] [ENSMUST00000107172]	chr11:101832519-101832460	7.52	10.48	0.13
828	A_30_P01027015	chr14:55433382-55452757_F		lincRNA:chr14:55433382-55452757 forward strand	chr14:55445340-55445399	7.87	10.84	0.13
829	A_55_P2026950	ENSMUST00000101381		RIKEN cDNA C530030P08 gene Gene [Source:MGI Symbol;Acc:MGI:2144664] [ENSMUST00000101381]	chr11:32625904-32625963	8.29	11.27	0.13
830	A_51_P157042	NM_010217	Ctgf	Mus musculus connective tissue growth factor (Ctgf), mRNA [NM_010217]	chr10:24318281-24318340	6.78	9.75	0.13
831	A_55_P2123502	NM_023844	Jam2	Mus musculus junction adhesion molecule 2 (Jam2), mRNA [NM_023844]	chr16:84816558-84816617	8.64	11.61	0.13
832	A_55_P2187076	NM_011430	Sncg	Mus musculus synuclein, gamma (Sncg), mRNA [NM_011430]	chr14:35183556-35183497	6.84	9.82	0.13
833	A_51_P320852	NM_007657	Cd9	Mus musculus CD9 antigen (Cd9), mRNA [NM_007657]	chr6:125410446-125410387	7.15	10.14	0.13
834	A_52_P20906	NM_011658	Twist1	Mus musculus twist homolog 1 (Drosophila) (Twist1), mRNA [NM_011658]	chr12:34644411-34644470	5.86	8.84	0.13
835	A_55_P2063471	XR_001926	Gm11814	PREDICTED: Mus musculus similar to Ldha protein (LOC666840), misc RNA [XR_001926]	chr4:10365541-10365482	11.98	14.98	0.13
836	A_52_P150683	XM_972673	LOC674050	PREDICTED: Mus musculus similar to adaptor protein Gads (LOC674050), mRNA [XM_972673]	chr15:80482962-80483021	5.82	8.82	0.13
837	A_52_P527800	NM_145158	Emilin2	Mus musculus elastin microfibril interfacer 2 (Emilin2), mRNA [NM_145158]	chr17:71601697-71601638	7.09	10.09	0.13

838	A_52_P474814	NM_001037743	4921506M07Rik	Mus musculus RIKEN cDNA 4921506M07 gene (4921506M07Rik), mRNA [NM_001037743]	chr12:58718507-58718566	6.19	9.19	0.13
839	A_52_P151853	NM_001025262	Tpd52	Mus musculus tumor protein D52 (Tpd52), transcript variant 2, mRNA [NM_001025262]	chr3:8947530-8944703	8.31	11.31	0.13
840	A_30_P01031757	chr5:96591892-96606467_F		lincRNA:chr5:96591892-96606467 forward strand	chr5:96603273-96603332	8.07	11.08	0.12
841	A_55_P2118810	XR_032614	Gm5987	PREDICTED: Mus musculus similar to L-lactate dehydrogenase A chain (LDH-A) (LDH muscle subunit) (LDH-M) (LOC546882), misc RNA [XR_032614]	chr5:106645294-106645353	7.68	10.69	0.12
842	A_55_P2012984	NM_001136069	Ldha	Mus musculus lactate dehydrogenase A (Ldha), transcript variant 2, mRNA [NM_001136069]	chr7:54110937-54110996	11.73	14.75	0.12
843	A_55_P1988804	NM_013515	Stom	Mus musculus stomatin (Stom), mRNA [NM_013515]	chr2:35177093-35177034	7.31	10.33	0.12
844	A_55_P2132345	NM_024452	Luzp1	Mus musculus leucine zipper protein 1 (Luzp1), mRNA [NM_024452]	chr4:136099154-136099213	7.42	10.44	0.12
845	A_55_P2048800	NM_001136055	Cd82	Mus musculus CD82 antigen (Cd82), transcript variant 2, mRNA [NM_001136055]	chr2:93274078-93274019	7.36	10.39	0.12
846	A_30_P01025234	chr5:96591892-96606467_F		lincRNA:chr5:96591892-96606467 forward strand	chr5:96603301-96603360	7.82	10.84	0.12
847	A_66_P130647	XM_001480325	LOC100048479	PREDICTED: Mus musculus similar to hepatocyte nuclear factor 6 beta (LOC100048479), mRNA [XM_001480325]	chr9:74737402-74737461	6.82	9.85	0.12
848	A_30_P01024356	chr16:46384974-46391331_R		lincRNA:chr16:46384974-46391331 reverse strand	chr16:46388248-46388189	6.94	9.97	0.12
849	A_55_P2091359	NM_008812	Padi2	Mus musculus peptidyl arginine deiminase, type II (Padi2), mRNA [NM_008812]	chr4:140508441-140508500	6.59	9.63	0.12
850	A_51_P449233	NM_172444	Thsd4	Mus musculus thrombospondin, type I, domain containing 4 (Thsd4), transcript variant 2, mRNA [NM_172444]	chr9:59818131-59818072	7.69	10.72	0.12
851	A_55_P2078670	NM_144855	Cbs	Mus musculus cystathionine beta-synthase (Cbs), transcript variant 1, mRNA [NM_144855]	chr17:31749652-31749593	6.84	9.89	0.12
852	A_55_P2168823	XR_031967	Gm7669	PREDICTED: Mus musculus similar to 3-phosphoglycerate dehydrogenase (LOC665516), misc RNA [XR_031967]	chr8:13745855-13745796	7.14	10.20	0.12
853	A_55_P2059352	NM_001109991	Col18a1	Mus musculus collagen, type XVIII, alpha 1 (Col18a1), transcript variant 1, mRNA [NM_001109991]	chr10:76514985-76514926	6.61	9.67	0.12

854	A_55_P2016623	XM_204772	Gm5068	PREDICTED: Mus musculus predicted gene, EG277089 (EG277089), mRNA [XM_204772]	chr12:75062366-75062425	6.25	9.32	0.12
855	A_30_P01027478	chr5:96591892-96606467_F		lincRNA:chr5:96591892-96606467 forward strand	chr5:96603471-96603530	7.65	10.72	0.12
856	A_51_P182303	NM_007743	Col1a2	Mus musculus collagen, type I, alpha 2 (Col1a2), mRNA [NM_007743]	chr6:4490619-4490678	7.44	10.51	0.12
857	A_55_P2088425	NM_015749	Tcn2	Mus musculus transcobalamin 2 (Tcn2), transcript variant 1, mRNA [NM_015749]	chr11:3817256-3817197	7.23	10.31	0.12
858	A_51_P464738	NM_011400	Slc2a1	Mus musculus solute carrier family 2 (facilitated glucose transporter), member 1 (Slc2a1), mRNA [NM_011400]	chr4:118809864-118809923	8.65	11.73	0.12
859	A_55_P2183587	NM_010296	Gli1	Mus musculus GLI-Kruppel family member GLI1 (Gli1), mRNA [NM_010296]	chr10:126767026-126766967	6.72	9.80	0.12
860	A_55_P2013236	NM_009789	S100g	Mus musculus S100 calcium binding protein G (S100g), mRNA [NM_009789]	chrX:159399983-159399924	5.90	8.98	0.12
861	A_51_P478458	NM_133239	Crb1	Mus musculus crumbs homolog 1 (Drosophila) (Crb1), mRNA [NM_133239]	chr1:141094983-141094924	7.20	10.29	0.12
862	A_51_P500949	NM_133919	Aff1	Mus musculus AF4/FMR2 family, member 1 (Aff1), transcript variant 2, mRNA [NM_133919]	chr5:104276766-104276825	6.68	9.76	0.12
863	A_52_P187940	NM_008494	Lfng	Mus musculus LFNG O-fucosylpeptide 3-beta-N-acetylglucosaminyltransferase (Lfng), mRNA [NM_008494]	chr5:141091224-141091283	6.59	9.67	0.12
864	A_55_P2051254	NR_003368	Pvt1	Mus musculus plasmacytoma variant translocation 1 (Pvt1), non-coding RNA [NR_003368]	chr15:62081020-62081079	6.53	9.62	0.12
865	A_55_P2046877	NM_008239	Foxq1	Mus musculus forkhead box Q1 (Foxq1), mRNA [NM_008239]	chr13:31652206-31652265	5.78	8.87	0.12
866	A_55_P2065991	NM_016740	S100a11	Mus musculus S100 calcium binding protein A11 (calgizzarin) (S100a11), mRNA [NM_016740]	chr3:93330022-93330081	7.87	10.97	0.12
867	A_55_P1954186	NM_011795	C1ql1	Mus musculus complement component 1, q subcomponent-like 1 (C1ql1), mRNA [NM_011795]	chr11:102801166-102801107	6.89	9.99	0.12
868	A_55_P2099665	ENSMUST00000115332		G protein-coupled receptor 37 Gene [Source:MGI Symbol;Acc:MGI:1313297] [ENSMUST00000115332]	chr6:25616953-25616894	5.10	8.20	0.12
869	A_30_P01024520	chr9:114412295-114418384_F		lincRNA:chr9:114412295-114418384 forward strand	chr9:114418261-114418320	7.31	10.41	0.12

870	A_30_P01025687	chr15:61984389-62102500_F		lincRNA:chr15:61984389-62102500 forward strand	chr15:62074025-62074084	6.56	9.67	0.12
871	A_55_P2084308	NM_010917	Nid1	Mus musculus nidogen 1 (Nid1), mRNA [NM_010917]	chr13:13604435-13604494	6.29	9.40	0.12
872	A_55_P2167501	NM_153807	Acsf2	Mus musculus acyl-CoA synthetase family member 2 (Acsf2), mRNA [NM_153807]	chr11:94419664-94419605	6.96	10.08	0.12
873	A_51_P410681	NM_018826	Irx5	Mus musculus Iroquois related homeobox 5 (Drosophila) (Irx5), mRNA [NM_018826]	chr8:94884923-94884982	6.11	9.23	0.12
874	A_66_P104265	ENSMUST00000068158		RIKEN cDNA 4930578G10 gene Gene [Source:MGI Symbol;Acc:MGI:1923202] [ENSMUST00000068158]	chr4:42749484-42774566	7.29	10.42	0.11
875	A_30_P01022315	chr1:34735032-34781146_F		lincRNA:chr1:34735032-34781146 forward strand	chr1:34781027-34781086	5.66	8.79	0.11
876	A_51_P357341	NM_033444	Clic1	Mus musculus chloride intracellular channel 1 (Clic1), mRNA [NM_033444]	chr17:35195587-35195646	10.58	13.71	0.11
877	A_52_P460734	NM_198408	Crhbp	Mus musculus corticotropin releasing hormone binding protein (Crhbp), mRNA [NM_198408]	chr13:96201962-96201903	6.94	10.07	0.11
878	A_52_P408025	NM_001143683	Mpped2	Mus musculus metallophosphoesterase domain containing 2 (Mpped2), transcript variant 1, mRNA [NM_001143683]	chr2:106707241-106707300	5.70	8.83	0.11
879	A_30_P01025875	chr17:41231055-41249764_R		lincRNA:chr17:41231055-41249764 reverse strand	chr17:41239083-41239024	9.20	12.34	0.11
880	A_30_P01018386	chr17:41231055-41249764_R		lincRNA:chr17:41231055-41249764 reverse strand	chr17:41239023-41238964	10.08	13.22	0.11
881	A_51_P519251	NM_019738	Nupr1	Mus musculus nuclear protein 1 (Nupr1), mRNA [NM_019738]	chr7:133767037-133766978	7.75	10.89	0.11
882	A_51_P511015	NM_010246	Fzd9	Mus musculus frizzled homolog 9 (Drosophila) (Fzd9), mRNA [NM_010246]	chr5:135724874-135724815	6.99	10.13	0.11
883	A_30_P01031241	chr2:59235155-59242518_F		lincRNA:chr2:59235155-59242518 forward strand	chr2:59237195-59237254	9.62	12.77	0.11
884	A_52_P663413	NM_011031	P4ha2	Mus musculus procollagen-proline, 2-oxoglutarate 4-dioxygenase (proline 4-hydroxylase), alpha II polypeptide (P4ha2), transcript variant 2, mRNA [NM_011031]	chr11:53945032-53945091	7.49	10.64	0.11
885	A_30_P01026835	chr14:55433382-55452757_R		lincRNA:chr14:55433382-55452757 reverse strand	chr14:55445167-55445108	8.04	11.20	0.11
886	A_51_P158678	NM_021427	Fam181b	Mus musculus family with sequence similarity 181, member B (Fam181b), mRNA [NM_021427]	chr7:100230092-100230151	9.38	12.54	0.11

887	A_52_P603038	NM_016968	Olig1	Mus musculus oligodendrocyte transcription factor 1 (Olig1), mRNA [NM_016968]	chr16:91271799-91271858	5.99	9.15	0.11
888	A_66_P122158	NR_003518	Pisd-ps3	Mus musculus phosphatidylserine decarboxylase, pseudogene 3 (Pisd-ps3), non-coding RNA [NR_003518]	chr11:003030755-003030814	10.17	13.34	0.11
889	A_51_P245533	NM_028602	Tex19.1	Mus musculus testis expressed gene 19.1 (Tex19.1), mRNA [NM_028602]	chr11:121009485-121009544	5.95	9.13	0.11
890	A_51_P515605	NM_009930	Col3a1	Mus musculus collagen, type III, alpha 1 (Col3a1), mRNA [NM_009930]	chr1:45405589-45405648	6.51	9.69	0.11
891	A_55_P2089530	NM_010221	Fkbp10	Mus musculus FK506 binding protein 10 (Fkbp10), transcript variant 1, mRNA [NM_010221]	chr11:100286080-100286139	6.56	9.74	0.11
892	A_52_P263095	NM_009643	Ahnak	Mus musculus AHNAK nucleoprotein (desmoyokin) (Ahnak), transcript variant 1, mRNA [NM_009643]	chr19:9093549-9093608	7.54	10.73	0.11
893	A_55_P2073789	XM_001003154	LOC676974	PREDICTED: Mus musculus similar to Glucose phosphate isomerase 1, transcript variant 2 (LOC676974), mRNA [XM_001003154]	chr7:35000280-35000221	9.23	12.43	0.11
894	A_51_P223404	NM_025836	Plin3	Mus musculus perilipin 3 (Plin3), mRNA [NM_025836]	chr17:56418488-56418429	7.78	10.98	0.11
895	A_55_P2175880	NM_019946	Mgst1	Mus musculus microsomal glutathione S-transferase 1 (Mgst1), mRNA [NM_019946]	chr6:138104846-138104905	6.09	9.29	0.11
896	A_30_P01032100	chr8:67542714-67561694_F		lincRNA:chr8:67542714-67561694 forward strand	chr8:67552826-67552885	6.16	9.36	0.11
897	A_30_P01032592	chr2:59235155-59242518_F		lincRNA:chr2:59235155-59242518 forward strand	chr2:59237815-59237874	8.47	11.67	0.11
898	A_55_P2000289	NM_011839	Mab21l2	Mus musculus mab-21-like 2 (C. elegans) (Mab21l2), mRNA [NM_011839]	chr3:86350802-86350743	9.20	12.40	0.11
899	A_55_P2042297	XR_031240	Gm5250	PREDICTED: Mus musculus similar to developmental pluripotency associated 5 (LOC383490), misc RNA [XR_031240]	chr1:13052538-13052597	7.03	10.24	0.11
900	A_55_P1971599	NM_007529	Bcan	Mus musculus brevican (Bcan), transcript variant 1, mRNA [NM_007529]	chr3:87791516-87791457	6.80	10.01	0.11
901	A_55_P1957424	NM_172294	Sulf1	Mus musculus sulfatase 1 (Sulf1), mRNA [NM_172294]	chr1:12849507-12849566	6.01	9.23	0.11
902	A_55_P1991164	NM_133241	Mlc1	Mus musculus megalencephalic leukoencephalopathy with subcortical cysts 1 homolog (human) (Mlc1), mRNA [NM_133241]	chr15:88786395-88786336	6.25	9.48	0.11

903	A_55_P2013273	XR_034534	Gm13937	PREDICTED: Mus musculus similar to lactate dehydrogenase 1, A chain (LOC623145), misc RNA [XR_034534]	chr2:110049625-110049566	8.95	12.19	0.11
904	A_51_P411728	NM_172884	2900026A02Rik	Mus musculus RIKEN cDNA 2900026A02 gene (2900026A02Rik), mRNA [NM_172884]	chr5:113519094-113519035	6.93	10.17	0.11
905	A_51_P161086	NM_008908	Ppic	Mus musculus peptidylprolyl isomerase C (Ppic), mRNA [NM_008908]	chr18:53566155-53566096	6.57	9.82	0.11
906	A_52_P449417	NM_177545	Vangl1	Mus musculus vang-like 1 (van gogh, Drosophila) (Vangl1), mRNA [NM_177545]	chr3:101960779-101960720	6.66	9.91	0.11
907	A_55_P1990210	NM_029023	Scpep1	Mus musculus serine carboxypeptidase 1 (Scpep1), mRNA [NM_029023]	chr11:88785395-88785336	8.37	11.63	0.10
908	A_55_P2094729	NM_011437	Sox10	Mus musculus SRY-box containing gene 10 (Sox10), mRNA [NM_011437]	chr15:78985577-78985518	6.69	9.96	0.10
909	A_51_P191669	NM_007694	Chgb	Mus musculus chromogranin B (Chgb), mRNA [NM_007694]	chr2:132620703-132620762	10.61	13.88	0.10
910	A_55_P2040170	NM_008885	Pmp22	Mus musculus peripheral myelin protein 22 (Pmp22), mRNA [NM_008885]	chr11:62972989-62973048	5.99	9.27	0.10
911	A_51_P520849	NM_009144	Sfrp2	Mus musculus secreted frizzled-related protein 2 (Sfrp2), mRNA [NM_009144]	chr3:83577864-83577923	7.87	11.15	0.10
912	A_55_P1988699	NM_001110843	Cacna2d1	Mus musculus calcium channel, voltage-dependent, alpha2/delta subunit 1 (Cacna2d1), transcript variant a, mRNA [NM_001110843]	chr5:15880152-15880211	7.50	10.79	0.10
913	A_55_P2015541	NM_016868	Hif3a	Mus musculus hypoxia inducible factor 3, alpha subunit (Hif3a), transcript variant 2, mRNA [NM_016868]	chr7:17616914-17616855	7.20	10.49	0.10
914	A_51_P255682	NM_008594	Mfge8	Mus musculus milk fat globule-EGF factor 8 protein (Mfge8), transcript variant 1, mRNA [NM_008594]	chr7:86278748-86278689	9.17	12.47	0.10
915	A_55_P2125557	NM_010574	Irx2	Mus musculus Iroquois related homeobox 2 (Drosophila) (Irx2), mRNA [NM_010574]	chr13:72771499-72771558	5.25	8.55	0.10
916	A_51_P392687	NM_011701	Vim	Mus musculus vimentin (Vim), mRNA [NM_011701]	chr2:13504232-13504291	10.20	13.52	0.10
917	A_55_P2031272	NM_001039653	Lhx3	Mus musculus LIM homeobox protein 3 (Lhx3), mRNA [NM_001039653]	chr2:26055805-26055746	5.70	9.01	0.10
918	A_55_P2072373	NM_010833	Msn	Mus musculus moesin (Msn), mRNA [NM_010833]	chrX:93363832-93363891	8.61	11.93	0.10

919	A_55_P2055597	NM_001136077	Enpp2	Mus musculus ectonucleotide pyrophosphatase/phosphodiesterase 2 (Enpp2), transcript variant 1, mRNA [NM_001136077]	chr15:54670980-54670921	7.55	10.88	0.10
920	A_51_P422934	NM_146954	Olfr535	Mus musculus olfactory receptor 535 (Olfr535), mRNA [NM_146954]	chr7:147678773-147678832	5.49	8.83	0.10
921	A_55_P1973033	NM_019919	Ltbp1	Mus musculus latent transforming growth factor beta binding protein 1 (Ltbp1), transcript variant 1, mRNA [NM_019919]	chr17:75790530-75790589	6.78	10.12	0.10
922	A_66_P111660	NM_013602	Mt1	Mus musculus metallothionein 1 (Mt1), mRNA [NM_013602]	chr8:96704165-96704224	9.21	12.56	0.10
923	A_55_P2092310	XR_035004	Gm9210	PREDICTED: Mus musculus similar to 3-phosphoglycerate dehydrogenase (LOC668506), misc RNA [XR_035004]	chr17:53337968-53338027	6.99	10.35	0.10
924	A_55_P2071858	NM_008598	Mgmt	Mus musculus O-6-methylguanine-DNA methyltransferase (Mgmt), mRNA [NM_008598]	chr7:144319790-144319849	6.19	9.55	0.10
925	A_55_P2004248	NM_133237	Apcdd1	Mus musculus adenomatosis polyposis coli down-regulated 1 (Apcdd1), mRNA [NM_133237]	chr18:63112729-63112788	7.23	10.60	0.10
926	A_30_P01031011	chr1:34734249-34786649_R		lincRNA:chr1:34734249-34786649 reverse strand	chr1:34780590-34780531	6.59	9.96	0.10
927	A_55_P1959425	NM_030696	Slc16a3	Mus musculus solute carrier family 16 (monocarboxylic acid transporters), member 3 (Slc16a3), mRNA [NM_030696]	chr11:120820023-120820082	8.02	11.40	0.10
928	A_55_P1986296	NM_178598	Tagln2	Mus musculus transgelin 2 (Tagln2), mRNA [NM_178598]	chr1:174437450-174437506	6.19	9.57	0.10
929	A_55_P2104612	ENSMUST00000113886			chr17:35517074-35517015	7.75	11.14	0.10
930	A_55_P2115713	NM_010917	Nid1	Mus musculus nidogen 1 (Nid1), mRNA [NM_010917]	chr13:13602780-13602839	5.82	9.21	0.10
931	A_55_P2017694	NM_001110337	Gprc5c	Mus musculus G protein-coupled receptor, family C, group 5, member C (Gprc5c), transcript variant 1, mRNA [NM_001110337]	chr11:114733852-114733911	5.66	9.05	0.10
932	A_55_P2087265	ENSMUST00000084055	Gm7676	PREDICTED: Mus musculus predicted gene, EG665536 (EG665536), mRNA [XM_977607]	chr8:13896764-13896823	5.63	9.03	0.10
933	A_51_P246543	NM_010096	Ebf3	Mus musculus early B-cell factor 3 (Ebf3), transcript variant 3, mRNA [NM_010096]	chr7:144385549-144385490	6.68	10.08	0.10
934	A_52_P213696	NM_027402	Fndc5	Mus musculus fibronectin type III domain containing 5 (Fndc5), mRNA [NM_027402]	chr4:128821749-128821808	6.41	9.82	0.09

935	A_51_P292008	NM_008161	Gpx3	Mus musculus glutathione peroxidase 3 (Gpx3), transcript variant 2, mRNA [NM_008161]	chr11:54723436-54723495	8.16	11.58	0.09
936	A_55_P1975843	XR_030977	Gm8144	PREDICTED: Mus musculus similar to interferon-inducible protein 16 (LOC666517), misc RNA [XR_030977]	chrX:57679316-57679257	7.82	11.24	0.09
937	A_30_P01018527	chr1:34735032-34781146_F		lincRNA:chr1:34735032-34781146 forward strand	chr1:34781087-34781146	5.86	9.29	0.09
938	A_55_P2141878	NM_001136069	Ldha	Mus musculus lactate dehydrogenase A (Ldha), transcript variant 2, mRNA [NM_001136069]	chr7:54107294-54107353	10.19	13.64	0.09
939	A_55_P2000369	NM_009700	Aqp4	Mus musculus aquaporin 4 (Aqp4), mRNA [NM_009700]	chr18:15551423-15551364	6.07	9.52	0.09
940	A_30_P01029804	chr14:55433382-55452757_F		lincRNA:chr14:55433382-55452757 forward strand	chr14:55445153-55445212	8.29	11.75	0.09
941	A_55_P1983733	NM_027406	Aldh1l1	Mus musculus aldehyde dehydrogenase 1 family, member L1 (Aldh1l1), mRNA [NM_027406]	chr6:90549091-90549150	6.84	10.30	0.09
942	A_55_P2059095	XM_001000159	Fignl2	PREDICTED: Mus musculus predicted gene, EG668225 (EG668225), mRNA [XM_001000159]	chr15:100882557-100882498	8.58	12.06	0.09
943	A_30_P01020380	chr5:96591892-96606467_R		lincRNA:chr5:96591892-96606467 reverse strand	chr5:96603313-96603254	8.10	11.58	0.09
944	A_55_P2111355	NM_178444	Egfl7	Mus musculus EGF-like domain 7 (Egfl7), transcript variant a, mRNA [NM_178444]	chr2:26448142-26448201	7.35	10.83	0.09
945	A_51_P126626	NM_145459	Zfp503	Mus musculus zinc finger protein 503 (Zfp503), mRNA [NM_145459]	chr14:22803318-22803259	7.04	10.53	0.09
946	A_51_P329928	NM_013750	Phlda3	Mus musculus pleckstrin homology-like domain, family A, member 3 (Phlda3), mRNA [NM_013750]	chr1:137665642-137665701	7.10	10.59	0.09
947	A_55_P1953646	NM_144955	Nkx6-1	Mus musculus NK6 homeobox 1 (Nkx6-1), mRNA [NM_144955]	chr5:102088378-102088319	7.17	10.66	0.09
948	A_55_P2002884	NM_001101433	Zcchc24	Mus musculus zinc finger, CCHC domain containing 24 (Zcchc24), mRNA [NM_001101433]	chr14:26531198-26531139	6.97	10.47	0.09
949	A_52_P93467	NM_011521	Sdc4	Mus musculus syndecan 4 (Sdc4), mRNA [NM_011521]	chr2:164251270-164251211	6.09	9.59	0.09
950	A_52_P192596	NM_054053	Gpr98	Mus musculus G protein-coupled receptor 98 (Gpr98), mRNA [NM_054053]	chr13:81699773-81699714	6.90	10.41	0.09
951	A_55_P2015585	NM_001101433	Zcchc24	Mus musculus zinc finger, CCHC domain containing 24 (Zcchc24), mRNA [NM_001101433]	chr14:26531185-26531126	6.77	10.27	0.09

952	A_52_P476877	NM_001024918	Rfx4	Mus musculus regulatory factor X, 4 (influences HLA class II expression) (Rfx4), transcript variant 1, mRNA [NM_001024918]	chr10:84331133-84331590	7.62	11.13	0.09
953	A_30_P01031408	chr8:67552315-67561235_R		lincRNA:chr8:67552315-67561235 reverse strand	chr8:67552431-67552372	6.52	10.03	0.09
954	A_55_P1956448	NM_021324	Ttyh1	Mus musculus tweety homolog 1 (Drosophila) (Ttyh1), transcript variant 2, mRNA [NM_021324]	chr7:4086862-4086921	9.56	13.08	0.09
955	A_51_P264695	NM_016669	Crym	Mus musculus crystallin, mu (Crym), mRNA [NM_016669]	chr7:127330029-127329970	5.93	9.45	0.09
956	A_55_P1998578	NM_030694	Ifitm2	Mus musculus interferon induced transmembrane protein 2 (Ifitm2), mRNA [NM_030694]	chr7:148140940-148140881	8.31	11.85	0.09
957	A_65_P19832	NM_013697	Ttr	Mus musculus transthyretin (Ttr), mRNA [NM_013697]	chr18:20828519-20828577	5.82	9.37	0.09
958	A_55_P1993463	XR_031039	LOC676303	PREDICTED: Mus musculus similar to L-lactate dehydrogenase A chain (LDH-A) (LDH muscle subunit) (LDH-M) (LOC676303), misc RNA [XR_031039]		8.47	12.02	0.09
959	A_51_P370615	NM_009718	Neurog2	Mus musculus neurogenin 2 (Neurog2), mRNA [NM_009718]	chr3:127337805-127337864	6.22	9.77	0.09
960	A_51_P460643	NM_001079869	Hoxb3	Mus musculus homeobox B3 (Hoxb3), transcript variant 1, mRNA [NM_001079869]	chr11:96207114-96207173	6.09	9.65	0.09
961	A_52_P15300	NM_134032	Hoxb2	Mus musculus homeobox B2 (Hoxb2), mRNA [NM_134032]	chr11:96215122-96215181	6.29	9.85	0.09
962	A_55_P1957148	XM_925521	6430503K07Rik	PREDICTED: Mus musculus RIKEN cDNA 6430503K07 gene (6430503K07Rik), mRNA [XM_925521]	chr2:147014131-147014190	5.93	9.49	0.08
963	A_55_P2146254	NM_001112715	Ifitm1	Mus musculus interferon induced transmembrane protein 1 (Ifitm1), transcript variant 2, mRNA [NM_001112715]	chr7:148155659-148155718	5.53	9.09	0.08
964	A_51_P248044	NM_008483	Lamb2	Mus musculus laminin, beta 2 (Lamb2), mRNA [NM_008483]	chr9:108392189-108392248	7.10	10.67	0.08
965	A_51_P337195	NM_008952	Pipox	Mus musculus pipecolic acid oxidase (Pipox), mRNA [NM_008952]	chr11:77694332-77694273	7.84	11.41	0.08
966	A_55_P2000454	ENSMUST00000024228		Putative uncharacterized protein Fragment [Source:UniProtKB/TrEMBL;Acc:Q9CSX2] [ENSMUST00000024228]	chr2:93151479-93151420	6.63	10.21	0.08
967	A_55_P2141876	NM_001136069	Ldha	Mus musculus lactate dehydrogenase A (Ldha), transcript variant 2, mRNA [NM_001136069]	chr7:54103010-54103069	8.66	12.25	0.08

968	A_66_P101600	NM_008482	Lamb1-1	Mus musculus laminin B1 subunit 1 (Lamb1-1), mRNA [NM_008482]	chr12:32014411-32014470	8.01	11.61	0.08
969	A_52_P89683	XM_001474667	Gm14470	PREDICTED: Mus musculus similar to lactate dehydrogenase 1, A chain (LOC385319), mRNA [XM_001474667]	chrX:10258406-10258464	8.27	11.87	0.08
970	A_55_P2052655	NM_177753	Sox21	Mus musculus SRY-box containing gene 21 (Sox21), mRNA [NM_177753]	chr14:118633410-118633351	7.69	11.30	0.08
971	A_51_P501803	NM_010451	Hoxa2	Mus musculus homeobox A2 (Hoxa2), mRNA [NM_010451]	chr6:52112654-52112595	5.95	9.56	0.08
972	A_55_P2071646	ENSMUST00000054395		Putative uncharacterized proteinMCG148442 ; [Source:UniProtKB/TrEMBL;Acc:Q9D2M0] [ENSMUST00000054395]	chr5:136365387-136365328	6.85	10.47	0.08
973	A_55_P2150029	ENSMUST00000075226		PREDICTED: Mus musculus similar to developmental pluripotency associated 5 (LOC100046088), mRNA [XM_001475553]	chrX:108363831-108363890	6.29	9.92	0.08
974	A_55_P2137828	NM_022420	Gprc5b	Mus musculus G protein-coupled receptor, family C, group 5, member B (Gprc5b), mRNA [NM_022420]	chr7:126117227-126117168	8.00	11.63	0.08
975	A_52_P552665	NM_008057	Fzd7	Mus musculus frizzled homolog 7 (Drosophila) (Fzd7), mRNA [NM_008057]	chr1:59543192-59543251	6.03	9.70	0.08
976	A_55_P2002460	XM_001473424	Gm2399	PREDICTED: Mus musculus similar to Nid1 protein (LOC100039744), mRNA [XM_001473424]	chr13:12794813-12794872	6.61	10.28	0.08
977	A_55_P2047155	NM_001159569	Meis2	Mus musculus Meis homeobox 2 (Meis2), transcript variant 5, mRNA [NM_001159569]	chr2:115689700-115689641	9.03	12.71	0.08
978	A_52_P227267	NM_178405	Atp1a2	Mus musculus ATPase, Na+/K+ transporting, alpha 2 polypeptide (Atp1a2), mRNA [NM_178405]	chr1:174208729-174208670	6.34	10.03	0.08
979	A_55_P2067453	NM_028841	Tspan17	Mus musculus tetraspanin 17 (Tspan17), mRNA [NM_028841]	chr13:54898070-54898129	9.44	13.13	0.08
980	A_55_P2256943	AK082264	C230030N03Rik	Mus musculus 0 day neonate cerebellum cDNA, RIKEN full-length enriched library, clone:C230030N03 product:hypothetical protein, full insert sequence. [AK082264]	chr1:34781083-34781142	6.24	9.94	0.08
981	A_55_P2143946	NM_172523	Slc18a2	Mus musculus solute carrier family 18 (vesicular monoamine), member 2 (Slc18a2), mRNA [NM_172523]	chr19:59370106-59370165	8.73	12.44	0.08

982	A_55_P2148708	XR_031767	Gm6705	PREDICTED: Mus musculus similar to Lysosomal-associated protein transmembrane 4B (LOC626785), misc RNA [XR_031767]	chr17:16985913-16985972	9.25	12.97	0.08
983	A_51_P210350	NM_182959	Slc17a8	Mus musculus solute carrier family 17 (sodium-dependent inorganic phosphate cotransporter), member 8 (Slc17a8), mRNA [NM_182959]	chr10:89046218-89046159	6.56	10.29	0.08
984	A_55_P2015782	NM_029815	Bcas1	Mus musculus breast carcinoma amplified sequence 1 (Bcas1), transcript variant 1, mRNA [NM_029815]	chr2:170172707-170172648	7.44	11.17	0.08
985	A_52_P312467	NM_001079869	Hoxb3	Mus musculus homeobox B3 (Hoxb3), transcript variant 1, mRNA [NM_001079869]	chr11:96209002-96209061	5.90	9.67	0.07
986	A_30_P01022915	chr8:67542714-67561694_F		lincRNA:chr8:67542714-67561694 forward strand	chr8:67559601-67559660	6.09	9.86	0.07
987	A_51_P491350	NM_009932	Col4a2	Mus musculus collagen, type IV, alpha 2 (Col4a2), mRNA [NM_009932]	chr8:11448840-11448899	7.88	11.65	0.07
988	A_51_P483159	NM_177157	Gchfr	Mus musculus GTP cyclohydrolase I feedback regulator (Gchfr), mRNA [NM_177157]	chr2:118998049-118998108	6.90	10.68	0.07
989	A_51_P480119	NM_029942	Prelid2	Mus musculus PRELI domain containing 2 (Prelid2), mRNA [NM_029942]	chr18:42072068-42040925	6.76	10.54	0.07
990	A_51_P128876	NM_025378	Ifitm3	Mus musculus interferon induced transmembrane protein 3 (Ifitm3), mRNA [NM_025378]	chr7:148195650-148195591	7.26	11.05	0.07
991	A_52_P37681	NM_012056	Fkbp9	Mus musculus FK506 binding protein 9 (Fkbp9), mRNA [NM_012056]	chr6:56829199-56829258	6.87	10.66	0.07
992	A_51_P230904	NM_019877	Copz2	Mus musculus coatomer protein complex, subunit zeta 2 (Copz2), mRNA [NM_019877]	chr11:96722183-96722242	6.80	10.59	0.07
993	A_52_P193611	NM_181422	Pkd2l1	Mus musculus polycystic kidney disease 2-like 1 (Pkd2l1), mRNA [NM_181422]	chr19:44222665-44222606	6.94	10.74	0.07
994	A_52_P537571	NM_011635	Trap1a	Mus musculus tumor rejection antigen P1A (Trap1a), mRNA [NM_011635]	chrX:135871399-135871458	6.07	9.87	0.07
995	A_51_P494125	NM_007431	Alpl	Mus musculus alkaline phosphatase, liver/bone/kidney (Alpl), mRNA [NM_007431]	chr4:137297716-137297658	6.56	10.37	0.07
996	A_55_P2067505	NM_030696	Slc16a3	Mus musculus solute carrier family 16 (monocarboxylic acid transporters), member 3 (Slc16a3), mRNA [NM_030696]	chr11:120820030-120820089	7.65	11.47	0.07

997	A_66_P117417	ENSMUST00000023335	Pvrl3	poliovirus receptor-related 3 Gene [Source:MGI (curated);Acc:MGI:1930171] [ENSMUST00000023335]	chr16:46394512-46394453	8.14	11.97	0.07
998	A_51_P277431	NM_028804	Ccdc3	Mus musculus coiled-coil domain containing 3 (Ccdc3), mRNA [NM_028804]	chr2:5150942-5151001	5.20	9.03	0.07
999	A_52_P289213	NM_010928	Notch2	Mus musculus Notch gene homolog 2 (Drosophila) (Notch2), mRNA [NM_010928]	chr3:97953914-97953973	7.03	10.87	0.07
1000	A_55_P2105673	NM_010712	Lhx4	Mus musculus LIM homeobox protein 4 (Lhx4), mRNA [NM_010712]	chr1:157548915-157548856	5.86	9.71	0.07
1001	A_51_P235801	NM_007564	Zfp36l1	Mus musculus zinc finger protein 36, C3H type-like 1 (Zfp36l1), mRNA [NM_007564]	chr12:81209081-81209022	8.56	12.41	0.07
1002	A_30_P01032763	chr2:33500594-33501874_F		lincRNA:chr2:33500594-33501874 forward strand	chr2:33501619-33501678	6.22	10.08	0.07
1003	A_55_P2111163	NM_009789	S100g	Mus musculus S100 calcium binding protein G (S100g), mRNA [NM_009789]	chrX:159399997-159399938	6.11	9.97	0.07
1004	A_52_P590535	NM_007992	Fbln2	Mus musculus fibulin 2 (Fbln2), transcript variant 1, mRNA [NM_007992]	chr6:91222118-91222177	6.53	10.41	0.07
1005	A_52_P329451	NM_010777	Mbp	Mus musculus myelin basic protein (Mbp), transcript variant 7, mRNA [NM_010777]	chr18:82742266-82744570	6.59	10.49	0.07
1006	A_55_P1985911	NM_010919	Nkx2-2	Mus musculus NK2 transcription factor related, locus 2 (Drosophila) (Nkx2-2), transcript variant 1, mRNA [NM_010919]	chr2:147009008-147008949	7.38	11.28	0.07
1007	A_52_P86693	NM_026790	Ifi27l1	Mus musculus interferon, alpha-inducible protein 27 like 1 (Ifi27l1), transcript variant 1, mRNA [NM_026790]	chr12:104678353-104678412	7.47	11.37	0.07
1008	A_55_P2089710	NM_007904	Ednrb	Mus musculus endothelin receptor type B (Ednrb), transcript variant 1, mRNA [NM_007904]	chr14:104220929-104220870	6.87	10.79	0.07
1009	A_55_P2163897	XM_001477254	Gm3088	PREDICTED: Mus musculus hypothetical protein LOC100041002 (LOC100041002), mRNA [XM_001477254]	chr2:29700592-29700533	6.03	9.97	0.07
1010	A_55_P1985905	NM_001077632	Nkx2-2	Mus musculus NK2 transcription factor related, locus 2 (Drosophila) (Nkx2-2), transcript variant 2, mRNA [NM_001077632]	chr2:147003366-147003307	5.99	9.93	0.07

1011	A_52_P175028	ENSMUST00000030257		cache domain containing 1 Gene [Source:MGI (curated);Acc:MGI:2444177] [ENSMUST00000030257]	chr4:100676879-100676938	7.94	11.89	0.07
1012	A_51_P447545	NM_008341	Igfbp1	Mus musculus insulin-like growth factor binding protein 1 (Igfbp1), mRNA [NM_008341]	chr11:7102442-7102501	6.37	10.34	0.06
1013	A_52_P577662	NM_007904	Ednrb	Mus musculus endothelin receptor type B (Ednrb), transcript variant 1, mRNA [NM_007904]	chr14:104214543-104214484	7.03	11.02	0.06
1014	A_51_P151902	NM_145584	Spon1	Mus musculus spondin 1, (f-spondin) extracellular matrix protein (Spon1), mRNA [NM_145584]	chr7:121184084-121184143	9.62	13.63	0.06
1015	A_30_P01033125	chr8:67550064-67560987_R		lincRNA:chr8:67550064-67560987 reverse strand	chr8:67560853-67560794	6.46	10.48	0.06
1016	A_51_P427663	NM_007725	Cnn2	Mus musculus calponin 2 (Cnn2), mRNA [NM_007725]	chr10:79457785-79457844	6.84	10.88	0.06
1017	A_51_P423743	NM_018777	Cldn6	Mus musculus claudin 6 (Cldn6), mRNA [NM_018777]	chr17:23819027-23819086	6.19	10.22	0.06
1018	A_55_P2070992	NM_009657	Aldoc	Mus musculus aldolase C, fructose-bisphosphate (Aldoc), mRNA [NM_009657]	chr11:78140198-78140257	8.06	12.10	0.06
1019	A_51_P470328	NM_001042614	Sepp1	Mus musculus selenoprotein P, plasma, 1 (Sepp1), transcript variant 3, mRNA [NM_001042614]	chr15:3230418-3230477	7.94	11.99	0.06
1020	A_51_P124254	NM_009931	Col4a1	Mus musculus collagen, type IV, alpha 1 (Col4a1), mRNA [NM_009931]	chr8:11199068-11199009	7.93	11.98	0.06
1021	A_66_P124858	NM_001024474	Diras2	Mus musculus DIRAS family, GTP-binding RAS-like 2 (Diras2), mRNA [NM_001024474]	chr13:52599802-52599744	8.38	12.45	0.06
1022	A_52_P418489	NM_023476	Tinagl1	Mus musculus tubulointerstitial nephritis antigen-like 1 (Tinagl1), transcript variant 1, mRNA [NM_023476]	chr4:129843103-129843044	6.03	10.11	0.06
1023	A_55_P2137927	NM_001136055	Cd82	Mus musculus CD82 antigen (Cd82), transcript variant 2, mRNA [NM_001136055]	chr2:93260077-93260018	6.82	10.96	0.06
1024	A_51_P501844	NM_175475	Cyp26b1	Mus musculus cytochrome P450, family 26, subfamily b, polypeptide 1 (Cyp26b1), transcript variant 1, mRNA [NM_175475]	chr6:84522011-84521952	7.28	11.43	0.06
1025	A_55_P2143204	NM_146131	Pbxip1	Mus musculus pre-B-cell leukemia transcription factor interacting protein 1 (Pbxip1), mRNA [NM_146131]	chr3:89254114-89254173	6.99	11.14	0.06

1026	A_55_P2134236	NM_010446	Foxa2	Mus musculus forkhead box A2 (Foxa2), mRNA [NM_010446]	chr2:147868728-147868669	6.34	10.51	0.06
1027	A_55_P2044385	NM_028263	Fgfbp3	Mus musculus fibroblast growth factor binding protein 3 (Fgfbp3), mRNA [NM_028263]	chr19:36992177-36992118	8.28	12.44	0.06
1028	A_51_P161323	NM_175540	Eda2r	Mus musculus ectodysplasin A2 receptor (Eda2r), transcript variant 2, mRNA [NM_175540]	chrX:94531379-94531320	6.13	10.30	0.06
1029	A_51_P498720	NM_009784	Cacna2d1	Mus musculus calcium channel, voltage-dependent, alpha2/delta subunit 1 (Cacna2d1), transcript variant e, mRNA [NM_009784]	chr5:15876550-15876609	8.77	12.95	0.06
1030	A_55_P2001998	NM_145459	Zfp503	Mus musculus zinc finger protein 503 (Zfp503), mRNA [NM_145459]	chr14:22803985-22803926	7.24	11.44	0.06
1031	A_51_P327206	NM_011839	Mab21l2	Mus musculus mab-21-like 2 (C. elegans) (Mab21l2), mRNA [NM_011839]	chr3:86350340-86350281	6.80	11.01	0.05
1032	A_51_P305437	NM_009037	Rcn1	Mus musculus reticulocalbin 1 (Rcn1), mRNA [NM_009037]	chr2:105227267-105227208	8.35	12.59	0.05
1033	A_55_P2162712	NM_173391	Tph2	Mus musculus tryptophan hydroxylase 2 (Tph2), mRNA [NM_173391]	chr10:114516743-114516684	6.25	10.49	0.05
1034	A_51_P300506	NM_183405	Cox6b2	Mus musculus cytochrome c oxidase subunit VIb polypeptide 2 (Cox6b2), transcript variant 1, mRNA [NM_183405]	chr7:4703547-4703488	7.94	12.22	0.05
1035	A_55_P2013357	NM_023132	Renbp	Mus musculus renin binding protein (Renbp), transcript variant 1, mRNA [NM_023132]	chrX:71167750-71167691	6.61	10.89	0.05
1036	A_51_P268697	NM_148938	Slc1a3	Mus musculus solute carrier family 1 (glial high affinity glutamate transporter), member 3 (Slc1a3), mRNA [NM_148938]	chr15:8584295-8584236	6.82	11.10	0.05
1037	A_30_P01033603	chr2:33496712-33501183_F		lincRNA:chr2:33496712-33501183 forward strand	chr2:33500968-33501027	5.78	10.08	0.05
1038	A_55_P2060672	NM_010452	Hoxa3	Mus musculus homeobox A3 (Hoxa3), mRNA [NM_010452]	chr6:52119146-52119087	5.97	10.28	0.05
1039	A_55_P2000224	NM_001042580	Cd63	Mus musculus CD63 antigen (Cd63), transcript variant 1, mRNA [NM_001042580]	chr10:128349813-128349872	8.68	13.00	0.05
1040	A_52_P652336	ENSMUST00000018842	Lhx1	LIM homeobox protein 1 Gene [Source:MGI (curated);Acc:MGI:99783] [ENSMUST00000018842]	chr11:84331947-84331888	7.41	11.76	0.05
1041	A_55_P1997569	NM_183248	Nkx6-2	Mus musculus NK6 homeobox 2 (Nkx6-2), transcript variant 1, mRNA [NM_183248]	chr7:146767178-146767119	6.59	10.95	0.05

1042	A_55_P2083609	NM_015814	Dkk3	Mus musculus dickkopf homolog 3 (Xenopus laevis) (Dkk3), mRNA [NM_015814]	chr7:119259615-119259556	6.97	11.35	0.05
1043	A_51_P282508	NM_007484	Rhoc	Mus musculus ras homolog gene family, member C (Rhoc), mRNA [NM_007484]	chr3:104597300-104597359	8.19	12.57	0.05
1044	A_55_P2089223	NM_001177602	Ak4	Mus musculus adenylate kinase 4 (Ak4), nuclear gene encoding mitochondrial protein, transcript variant 1, mRNA [NM_001177602]	chr4:101139464-101139523	7.03	11.42	0.05
1045	A_55_P2039284	NM_013560	Hspb1	Mus musculus heat shock protein 1 (Hspb1), mRNA [NM_013560]	chr5:136365366-136365425	6.24	10.64	0.05
1046	A_55_P2056821	ENSMUST00000064349		RIKEN cDNA A330008L17 gene Gene [Source:MGI Symbol;Acc:MGI:2443215] [ENSMUST00000064349]	chr8:101946243-101946302	7.35	11.79	0.05
1047	A_51_P220162	NM_008716	Notch3	Mus musculus Notch gene homolog 3 (Drosophila) (Notch3), mRNA [NM_008716]	chr17:32257907-32257848	8.09	12.53	0.05
1048	A_55_P2146314	NM_033652	Lmx1a	Mus musculus LIM homeobox transcription factor 1 alpha (Lmx1a), mRNA [NM_033652]	chr1:169778804-169778863	7.18	11.65	0.05
1049	A_55_P2040549	NM_008888	Phox2b	Mus musculus paired-like homeobox 2b (Phox2b), mRNA [NM_008888]	chr5:67485826-67485767	6.22	10.70	0.05
1050	A_52_P593212	ENSMUST00000114435		RIKEN cDNA 5730446D14 gene Gene [Source:MGI Symbol;Acc:MGI:1913890] [ENSMUST00000114435]	chr6:52119107-52119166	6.03	10.52	0.05
1051	A_55_P2084303	NM_010917	Nid1	Mus musculus nidogen 1 (Nid1), mRNA [NM_010917]	chr13:13530138-13555992	6.66	11.19	0.04
1052	A_51_P376445	NM_008818	Rhox5	Mus musculus reproductive homeobox 5 (Rhox5), mRNA [NM_008818]		7.05	11.60	0.04
1053	A_55_P1961395	NM_010329	Pdpn	Mus musculus podoplanin (Pdpn), mRNA [NM_010329]	chr4:142857398-142857339	8.89	13.44	0.04
1054	A_30_P01020733	chr8:67542714-67561694_F		lincRNA:chr8:67542714-67561694 forward strand	chr8:67552709-67552768	7.03	11.59	0.04
1055	A_51_P393968	NM_007701	Vsx2	Mus musculus visual system homeobox 2 (Vsx2), mRNA [NM_007701]	chr12:85936221-85936280	6.21	10.78	0.04
1056	A_30_P01026088	chr2:33500594-33501874_F		lincRNA:chr2:33500594-33501874 forward strand	chr2:33501484-33501543	5.74	10.31	0.04
1057	A_55_P2084332	NM_001159620	Pigp	Mus musculus phosphatidylinositol glycan anchor biosynthesis, class P (Pigp), transcript variant 5, mRNA [NM_001159620]	chr16:94580704-94580645	6.22	10.81	0.04
1058	A_52_P387009	NM_028133	Egln3	Mus musculus EGL nine homolog 3 (C. elegans) (Egln3), mRNA [NM_028133]	chr12:55281012-55280953	5.95	10.55	0.04

1059	A_55_P2064962	NM_010329	Pdpn	Mus musculus podoplanin (Pdpn), mRNA [NM_010329]	chr4:142859146-142858329	7.63	12.23	0.04
1060	A_51_P123262	NM_029530	6330527O06Rik	Mus musculus RIKEN cDNA 6330527O06 gene (6330527O06Rik), mRNA [NM_029530]	chr2:135895179-135895238	7.49	12.13	0.04
1061	A_52_P289173	ENSMUST00000065087		Riken cDNA C130021I20 gene Gene [Source:MGI Symbol;Acc:MGI:3639863] [ENSMUST00000065087]	chr2:33501600-33501659	5.70	10.34	0.04
1062	A_55_P2067533	NM_010446	Foxa2	Mus musculus forkhead box A2 (Foxa2), mRNA [NM_010446]	chr2:147868673-147868614	5.63	10.29	0.04
1063	A_51_P187679	NM_028946	Nhedc1	Mus musculus Na+/H+ exchanger domain containing 1 (Nhedc1), mRNA [NM_028946]	chr3:135060709-135060768	5.72	10.37	0.04
1064	A_30_P01023432	chr1:34734249-34786649_R		lincRNA:chr1:34734249-34786649 reverse strand	chr1:34780667-34780608	6.29	11.01	0.04
1065	A_55_P1964348	NM_023608	Gdpd2	Mus musculus glycerophosphodiester phosphodiesterase domain containing 2 (Gdpd2), mRNA [NM_023608]	chrX:97934160-97934219	7.07	11.83	0.04
1066	A_55_P2030373	NM_178405	Atp1a2	Mus musculus ATPase, Na+/K+ transporting, alpha 2 polypeptide (Atp1a2), mRNA [NM_178405]	chr1:174201917-174201858	5.63	10.40	0.04
1067	A_51_P142744	NM_172294	Sulf1	Mus musculus sulfatase 1 (Sulf1), mRNA [NM_172294]	chr1:12850302-12850361	8.41	13.21	0.04
1068	A_51_P363947	NM_007669	Cdkn1a	Mus musculus cyclin-dependent kinase inhibitor 1A (P21) (Cdkn1a), transcript variant 1, mRNA [NM_007669]	chr17:29237377-29237436	7.49	12.31	0.04
1069	A_66_P138009	NR_033456	Gm4710	Mus musculus predicted gene 4710 (Gm4710), non-coding RNA [NR_033456]	chr17:76288283-76288342	6.76	11.59	0.04
1070	A_30_P01025068	H19		lincRNA:chr7:149761435-149764019 reverse strand	chr7:149761815-149761756	7.73	12.57	0.04
1071	A_55_P2106690	NM_011527	Tal1	Mus musculus T-cell acute lymphocytic leukemia 1 (Tal1), mRNA [NM_011527]	chr4:114744219-114744278	7.31	12.14	0.04
1072	A_55_P2110713	NM_007585	Anxa2	Mus musculus annexin A2 (Anxa2), mRNA [NM_007585]	chr9:69339532-69339591	6.59	11.46	0.03
1073	A_55_P2053933	NM_008259	Foxa1	Mus musculus forkhead box A1 (Foxa1), mRNA [NM_008259]	chr12:58641823-58641764	5.86	10.76	0.03
1074	A_52_P233305	NM_175501	Adamts12	Mus musculus a disintegrin-like and metallopeptidase (reprolysin type) with thrombospondin type 1 motif, 12 (Adamts12), mRNA [NM_175501]	chr15:11266072-11266131	5.74	10.67	0.03
1075	A_55_P2046807	NM_009242	Sparc	Mus musculus secreted acidic cysteine rich glycoprotein (Sparc), mRNA [NM_009242]	chr11:55208061-55208003	7.33	12.29	0.03

1076	A_30_P01018375	chr8:67542714-67561694_F		lincRNA:chr8:67542714-67561694 forward strand	chr8:67552649-67552708	6.74	11.71	0.03
1077	A_30_P01030588	chr8:67542714-67561694_F		lincRNA:chr8:67542714-67561694 forward strand	chr8:67560939-67560998	7.29	12.29	0.03
1078	A_51_P302566	NM_172778	Maob	Mus musculus monoamine oxidase B (Maob), nuclear gene encoding mitochondrial protein, mRNA [NM_172778]	chrX:16286486-16286427	6.62	11.64	0.03
1079	A_52_P1037027	ENSMUST00000098718		Putative uncharacterized protein [Source:UniProtKB/TrEMBL;Acc:Q3V438] [ENSMUST00000098718]	chr8:67552607-67552548	7.09	12.11	0.03
1080	A_55_P2028171	NM_025274	Dppa5a	Mus musculus developmental pluripotency associated 5A (Dppa5a), mRNA [NM_025274]	chr9:78214925-78214866	7.03	12.05	0.03
1081	A_30_P01021588	H19		lincRNA:chr7:149761435-149764019 reverse strand	chr7:149761752-149761693	7.44	12.51	0.03
1082	A_51_P374726	NM_008987	Ptx3	Mus musculus pentraxin related gene (Ptx3), mRNA [NM_008987]	chr3:66029245-66029304	6.37	11.52	0.03
1083	A_52_P356698	NM_013665	Shox2	Mus musculus short stature homeobox 2 (Shox2), mRNA [NM_013665]	chr3:66779814-66779435	6.24	11.39	0.03
1084	A_51_P109840	NM_011707	Vtn	Mus musculus vitronectin (Vtn), mRNA [NM_011707]	chr11:78315393-78315665	5.86	11.02	0.03
1085	A_55_P2064776	NR_024257	4930412O13Rik	Mus musculus RIKEN cDNA 4930412O13 gene (4930412O13Rik), non-coding RNA [NR_024257]	chr2:9808308-9808367	6.11	11.35	0.03
1086	A_52_P1092823	NM_010573	Irx1	Mus musculus Iroquois related homeobox 1 (Drosophila) (Irx1), mRNA [NM_010573]	chr13:72095794-72095735	6.27	11.57	0.03
1087	A_55_P2097962	XM_001481064	9030622O22Rik	PREDICTED: Mus musculus hypothetical protein LOC100048732 (LOC100048732), mRNA [XM_001481064]	chr2:147798641-147798582	6.03	11.35	0.03
1088	A_52_P163021	NM_182959	Slc17a8	Mus musculus solute carrier family 17 (sodium-dependent inorganic phosphate cotransporter), member 8 (Slc17a8), mRNA [NM_182959]	chr10:89037689-89037630	5.42	10.77	0.03
1089	A_51_P518841	NM_153111	Fev	Mus musculus FEV (ETS oncogene family) (Fev), mRNA [NM_153111]	chr1:74928319-74928260	6.97	12.32	0.03
1090	A_55_P2035946	NM_001002927	Penk	Mus musculus preproenkephalin (Penk), mRNA [NM_001002927]	chr4:4060783-4060724	7.96	13.37	0.02
1091	A_30_P01027977	chr2:33496712-33501183_F		lincRNA:chr2:33496712-33501183 forward strand	chr2:33501079-33501138	5.93	11.37	0.02
1092	A_30_P01028827	H19		lincRNA:chr7:149761435-149764019 reverse strand	chr7:149762172-149762113	8.69	14.21	0.02
1093	A_51_P250058	NM_010137	Epas1	Mus musculus endothelial PAS domain protein 1 (Epas1), mRNA [NM_010137]	chr17:87232615-87232674	6.53	12.08	0.02

1094	A_52_P281702	NM_010518	Igfbp5	Mus musculus insulin-like growth factor binding protein 5 (Igfbp5), mRNA [NM_010518]	chr1:72904888-72904829	8.73	14.30	0.02
1095	A_55_P2046812	NM_009242	Sparc	Mus musculus secreted acidic cysteine rich glycoprotein (Sparc), mRNA [NM_009242]	chr11:55208998-55208939	9.12	14.77	0.02
1096	A_52_P233441	NM_008090	Gata2	Mus musculus GATA binding protein 2 (Gata2), mRNA [NM_008090]	chr6:88156362-88156421	5.66	11.30	0.02
1097	A_30_P01026972	H19		lincRNA:chr7:149761435-149764019 reverse strand	chr7:149762232-149762173	8.22	14.00	0.02
1098	A_55_P1960916	NM_028133	Egln3	Mus musculus EGL nine homolog 3 (C. elegans) (Egln3), mRNA [NM_028133]	chr12:55281311-55281252	6.19	12.02	0.02
1099	A_55_P1961127	NR_001592	H19	Mus musculus H19 fetal liver mRNA (H19), non-coding RNA [NR_001592]	chr7:149761551-149761492	8.68	14.52	0.02
1100	A_30_P01028007	H19		lincRNA:chr7:149761435-149764019 reverse strand	chr7:149762016-149761957	8.36	14.36	0.02
1101	A_55_P2074942	NM_010484	Slc6a4	Mus musculus solute carrier family 6 (neurotransmitter transporter, serotonin), member 4 (Slc6a4), mRNA [NM_010484]	chr11:76845777-76845836	5.80	11.99	0.01
1102	A_30_P01026417	chr2:33500594-33501874_F		lincRNA:chr2:33500594-33501874 forward strand	chr2:33501799-33501858	6.07	12.40	0.01
1103	A_52_P64356	NM_010097	Sparcl1	Mus musculus SPARC-like 1 (Sparcl1), mRNA [NM_010097]	chr5:104508208-104508149	6.40	12.76	0.01
1104	A_55_P1967149	NM_008393	Irx3	Mus musculus Iroquois related homeobox 3 (Drosophila) (Irx3), mRNA [NM_008393]	chr8:94322535-94322476	5.16	11.80	0.01
1105	A_66_P111011	NM_008091	Gata3	Mus musculus GATA binding protein 3 (Gata3), mRNA [NM_008091]	chr2:9779610-9779551	5.78	12.85	0.01
1106	A_55_P2118173	NM_010514	Igf2	Mus musculus insulin-like growth factor 2 (Igf2), transcript variant 1, mRNA [NM_010514]	chr7:149839720-149839661	5.95	13.37	0.01

Table S9 Supervised Heatmap Genes

ProbeID	Accession	GeneSymbol	Description	GenomicCoordinates	aveA	Lhx6_GFPvsnon_GFP.M	Lhx6_GFPvsnon_GFP.Fold	GFPvsnon_GFP.M	GFPvsnon_GFP.Fold	Lhx6_GFPvsGFP.M	Lhx6_GFPvsGFP.Fold	Lhx6_GFP	non_GFP	GFP
A_55_P21535	XR_031824	Gm7108	PREDICTED: M	chrX:8704771	6.21162411	-0.3807747	0.76802508	1.81829667	3.52664577	-2.1990713	0.21777778	5.35167544	5.73245011	7.55074679
A_55_P19999	XM_0014759	Gm3187	PREDICTED: M	chr14:677227	12.5024991	-2.6001298	0.16492365	-0.8175687	0.56739734	-1.7825611	0.29066694	11.0416021	13.6417319	12.8241632
A_52_P28921	NM_010928	Notch2	Mus musculu	chr3:9795391	8.61016941	-3.8420011	0.06973366	-3.3632689	0.09717514	-0.4787322	0.71760797	7.169925	11.0119261	7.64865718
A_30_P01026	chr5:96591892-96606467_	lincRNA:chr5:	chr5:9660317	10.220056	-2.3431374	0.19708128	-0.8020281	0.57354233	-1.5411092	0.34362116	8.92530717	11.2684445	10.4664164
A_55_P20861	NM_018887	Cyp39a1	Mus musculu	chr17:438877	8.87383384	-1.0047868	0.49834376	1.15777929	2.23113728	-2.1625661	0.22335863	7.81804952	8.82283635	9.98061564
A_30_P01024	chr16:59557120-59602070	lincRNA:chr16	chr16:595573	6.92062705	-0.8676693	0.5480315	1.45259959	2.73700787	-2.3202689	0.20023015	5.857981	6.72565028	8.17824987
A_52_P22223	XM_001476722		PREDICTED: M	chr8:9341200	9.7753629	2.94535428	7.70264682	0.41682633	1.33498759	2.52852794	5.76982652	11.5999903	8.65463603	9.07146236
A_51_P19550	NM_007778	Csf1	Mus musculu	chr3:1075443	7.02498388	-2.1299975	0.22845825	-2.186413	0.21969698	0.05641551	1.03987889	6.33378987	8.4637874	6.27737436
A_55_P20062	NM_0010099	Txnip	Mus musculu	chr3:9636420	10.4958492	-2.7030063	0.1535727	-0.1831274	0.88079156	-2.5198789	0.17435759	8.7548875	11.4578938	11.2747664
A_55_P20876	NM_0010426	Cp	Mus musculu	chr3:1990751	6.30352725	-2.0127673	0.24779736	-0.8014089	0.57378855	-1.2113584	0.4318618	5.22881869	7.24158599	6.44017706
A_51_P12425	NM_009931	Col4a1	Mus musculu	chr8:1119906	10.5822054	-4.0487807	0.06042207	-0.5693277	0.67393075	-3.4794529	0.08965619	8.07279417	12.1215748	11.5522471
A_51_P11390	NM_025467	Gkn2	Mus musculu	chr6:8732928	7.27798082	-2.7623169	0.1473872	-0.9414563	0.520707	-1.8208606	0.28305207	5.75025498	8.51257188	7.57111559
A_66_P11575	NM_026788	Mthfd2l	Mus musculu	chr5:9145024	10.6956392	1.55469808	2.93772242	-0.5382366	0.6886121	2.09293465	4.26614987	11.9115168	10.3568187	9.81858218
A_55_P21366	NM_0010812	Crnn	Mus musculu	chr3:9295333	6.90160386	-0.4604586	0.72675522	1.79518021	3.47058824	-2.2556388	0.20940405	5.99623808	6.45669665	8.25187686
A_55_P20650	ENSMUST00000085463		histone deace	chr12:347921	7.93873182	-2.0935014	0.23431133	-0.1181743	0.92135289	-1.9753271	0.25431225	6.58245565	8.67595703	8.55778278
A_51_P51925	NM_019738	Nupr1	Mus musculu	chr7:1337670	9.62903653	-3.1432261	0.11318651	-1.0625276	0.47879246	-2.0806985	0.23639993	7.88772834	11.0309545	9.9684268
A_55_P20471	NM_008714	Notch1	Mus musculu	chr2:2631468	8.86857052	-2.1317316	0.22818382	-2.5023828	0.17648497	0.37065119	1.29293629	8.28154371	10.4132753	7.91089253
A_51_P47696	NM_0010813	Csmd3	Mus musculu	chr15:477815	7.97861305	2.9145532	7.53994083	0.57353515	1.48816568	2.34101805	5.0666004	9.73047014	6.81591694	7.38945209
A_30_P01025	chr4:8865575-8899400_F		lincRNA:chr4:	chr4:8866983	7.17118428	-0.2036081	0.86837607	1.89517003	3.71965812	-2.0987782	0.23345588	6.40372219	6.60733031	8.50250034
A_55_P19756	NM_0010396	Rhox4g	Mus musculu	chrX:3506123	7.3602523	-0.3181479	0.80209895	2.00916342	4.02548726	-2.3273113	0.19925512	6.47843258	6.79658045	8.80574387
A_66_P11404	NM_172509	Kctd7	Mus musculu	chr5:1306315	8.78011962	-0.4376575	0.73833246	1.84163649	3.58416361	-2.279294	0.20599854	7.87446912	8.31212663	10.1537631
A_55_P19867	XM_895013	1700055N04I	PREDICTED: M	chr19:396647	6.14190179	-0.0857299	0.94230769	2.19921931	4.59230769	-2.2849492	0.20519263	5.35167544	5.43740531	7.63662462
A_55_P19917	NM_019417	Pdlim4	Mus musculu	chr11:538684	9.76743222	-2.5701776	0.16838347	-2.5542195	0.17025635	-0.0159581	0.98899966	8.90538701	11.4755646	8.92134508
A_55_P20517	NM_015739	Gbx1	Mus musculu	chr5:2401030	7.87288843	3.15306833	8.89545455	1.87640532	3.67159091	1.27666301	2.42277933	9.34946554	6.19639721	8.07280253
A_55_P19674	NM_008221	Hbb-y	Mus musculu	chr7:1110003	7.75238501	7.40524628	169.512315	0.61078849	1.5270936	6.79445779	111.003226	12.4856197	5.08037342	5.6911619
A_55_P21234	ENSMUST00000107082		MCG21374, is	chr11:102437	6.31476273	-2.0577155	0.24019608	-1.2261691	0.42745098	-0.8315464	0.56192661	5.35167544	7.40939094	6.18322182
A_55_P20514	NM_009465	Axl	Mus musculu	chr7:2654260	7.10202055	-2.2452947	0.21091086	-1.7043099	0.306868	-0.5409848	0.68730159	6.17326071	8.41855541	6.71424552
A_55_P19796	NM_011309	S100a1	Mus musculu	chr3:9031501	6.72260688	-2.5823931	0.16696376	-1.6453571	0.31966726	-0.937036	0.52230483	5.54946382	8.13185696	6.48649986
A_51_P26156	NM_198674	Fbxw26	Mus musculu	chr9:1096247	7.11759328	-0.4213292	0.74673629	3.78539484	13.7885117	-4.2067241	0.05415641	5.57490884	5.99623808	9.78163293
A_30_P01023	chr3:41143423-41359623_	lincRNA:chr3:	chr3:4121501	6.57095286	-0.9072234	0.53321033	1.1285223	2.18634686	-2.0357457	0.24388186	5.58996318	6.49718654	7.62570884
A_52_P16959	NM_177669	Skint11	Mus musculu	chr4:1139174	5.97711899	-0.2031629	0.86864407	2.24147826	4.72881356	-2.4446412	0.18369176	5.0945176	5.29768055	7.53915881
A_55_P19713	XM_980161	Rpl17-ps2	PREDICTED: M	chr12:969292	7.37171313	-0.0068374	0.99527187	3.70332275	13.0260047	-3.7101602	0.07640653	6.13271392	6.13955135	9.8428741
A_30_P01019	chr13:112337405-1123564	lincRNA:chr13	chr13:112350	6.8050101	0.14268842	1.1039604	2.05259495	4.14851485	-1.9099065	0.26610979	6.2159374	6.07324898	8.12584393

A_55_P2143(	NM_013657	Sema3c	Mus musculu	chr5:172359	9.74342202	3.3712499	10.3477837	0.60334993	1.51924014	2.76789998	6.81115742	11.7898053	8.41855541	9.02190534
A_55_P2092	XR_035004	Gm9210	PREDICTED: I	chr17:53337	8.55624289	-3.3616417	0.09728481	-2.4526965	0.18266898	-0.9089452	0.53257432	7.13271392	10.4943556	8.04165915
A_55_P2444	NM_001035	Akap2	Mus musculu	chr4:579075	8.66737249	-2.0735746	0.23757013	-1.1764288	0.44244535	-0.8971458	0.53694797	7.67713234	9.75070699	8.57427815
A_55_P2113	NM_001113	Gria1	Mus musculu	chr11:57143	9.54795169	2.18067185	4.53364632	3.25905436	9.57355243	-1.0783825	0.47355946	9.91538147	7.73470962	10.993764
A_55_P2142	NM_001122	E330014E10F	Mus musculu	chr5:962309	6.46333747	-0.8774468	0.5443299	1.25682391	2.38969072	-2.1342707	0.22778257	5.45943162	6.33687844	7.59370235
A_55_P2156	NM_001007	BB014433	Mus musculu	chr8:150417	7.91252036	-0.1599083	0.89508197	2.13947307	4.40601093	-2.2993814	0.20315019	7.09275714	7.25266543	9.39213851
A_30_P0102	chr11:68200716-68214458	lincRNA:chr1	chr11:68211	6.51643785	-0.519253	0.697733	1.9244685	3.79596977	-2.4437215	0.18380889	5.52877967	6.0480327	7.9725012	
A_30_P0102	chr8:124355710-12440116	lincRNA:chr8	chr8:124397	7.38549245	-1.8383709	0.27963738	0.29224782	1.22454672	-2.1306187	0.22835991	6.06249593	7.90086681	8.19311463	
A_55_P2120	ENSMUST00000110633	zinc finger, M	chr13:96896	11.9252924	-2.5618066	0.16936332	0.23744866	1.17890598	-2.7992553	0.14366143	10.1382718	12.7000784	12.9375271	
A_55_P1965	NM_008073	Gabrg2	Mus musculu	chr11:41725	10.3627294	2.94067706	7.6777153	0.73525675	1.6646937	2.20542032	4.61208889	12.0780952	9.13741813	9.87267488
A_55_P2165	NM_023317	Nde1	Mus musculu	chr16:14192	8.61315178	-1.5789958	0.33471479	-2.9146298	0.13262	1.33563396	2.52386364	8.53203118	10.111027	7.19639721
A_30_P0102	chr6:117829576-11783424	lincRNA:chr6	chr6:117829	7.21242727	-0.6969519	0.61687414	1.59556573	3.02213001	-2.2925177	0.20411899	6.2159374	6.91288934	8.50845506	
A_55_P2094	NM_001039	Gm14137	Mus musculu	chr2:119002	7.54397848	-0.0698214	0.95275591	3.49059034	11.2401575	-3.5604118	0.08476357	6.33390074	6.40372219	9.89431253
A_55_P2008	NM_175418	Mybpc1	Mus musculu	chr10:87985	6.62414078	-2.2551675	0.20947247	-1.1454136	0.45206007	-1.1097539	0.46337308	5.50250034	7.7576678	6.61225419
A_52_P6409	NM_177577	Dcdc2a	Mus musculu	chr13:25297	6.87528338	-0.8962252	0.53729072	1.19771414	2.29375951	-2.0939393	0.23424021	5.87856187	6.77478706	7.9725012
A_51_P4801	NM_021541	Cryba2	Mus musculu	chr1:749372	7.07820202	-1.3788351	0.38452915	0.96562901	1.9529148	-2.3444641	0.19690011	5.83710227	7.2159374	8.18156641
A_66_P1258	NM_172410	Nup93	Mus musculu	chr8:968388	9.66212727	1.48362413	2.7965035	2.05735938	4.16223776	-0.5737352	0.671875	9.96542357	8.48179943	10.5391588
A_52_P6350	NM_027677	Gpr39	Mus musculu	chr1:127769	7.61248369	-2.4487581	0.18316832	0.10067795	1.07227723	-2.5494361	0.17082179	5.94641896	8.39517708	8.49585503
A_30_P0102	chr18:38302523-38302756	lincRNA:chr1	chr18:38302	6.90572073	-1.2517318	0.41994382	1.29658114	2.45646067	-2.5483129	0.17095483	5.63903917	6.89077093	8.18735207	
A_52_P3855	NM_009317	Tal2	Mus musculu	chr4:537996	7.10610691	-1.9717242	0.25494816	-2.1086944	0.23185674	0.13697028	1.0995935	6.49452228	8.46624644	6.357552
A_51_P2774	NM_028804	Ccdc3	Mus musculu	chr2:515094	6.88914962	-3.9236663	0.06589595	-2.9236663	0.13179191	-1	0.5	5.24792751	9.17159382	6.24792751
A_55_P2144	NM_028589	1700125H20	Mus musculu	chr11:84994	7.10665581	-0.029504	0.97975709	2.25925724	4.78744939	-2.2887612	0.20465116	6.33390074	6.36340473	8.62266197
A_51_P4845	NM_019938	Pmfbp1	Mus musculu	chr8:112066	7.45990632	-2.9621602	0.12832194	-0.9923522	0.50265756	-1.969808	0.25528701	5.81591694	8.77807713	7.78572491
A_55_P2016	ENSMUST00000105884			chr4:133614	6.8823989	-0.2097636	0.8646789	2.30729482	4.94954128	-2.5170584	0.1746988	5.97345821	6.18322182	8.49051665
A_52_P2146	NM_011448	Sox9	Mus musculu	chr11:11264	7.69215896	-1.3890274	0.38182212	-2.6402581	0.16039954	1.2512307	2.38044401	7.64622673	9.03525412	6.39499603
A_30_P0101	chr10:116814196-1169206	lincRNA:chr1	chr10:11684	6.57443091	-0.1424869	0.90595611	2.66842932	6.35736677	-2.8109163	0.14250493	5.58996318	5.73245011	8.40087944	
A_52_P5157	NM_017378	Pcdh12	Mus musculu	chr18:38426	6.7154508	-0.0701448	0.95254237	3.35767128	10.2508475	-3.4278161	0.09292328	5.54946382	5.61960864	8.97727992
A_30_P0102	chr2:33496712-33501183_	lincRNA:chr2	chr2:335010	9.11305687	-5.494488	0.02218167	-1.7057969	0.30655186	-3.7886911	0.07235863	6.01866384	11.5131519	9.80735492	
A_51_P1266	NM_145459	Zfp503	Mus musculu	chr14:22803	8.94569034	-3.4863527	0.08922843	-1.6801939	0.31204069	-1.8061588	0.28595126	7.18151982	10.6678726	8.98767864
A_52_P1307	NM_013568	Kcna6	Mus musculu	chr6:126658	10.919777	-2.64886	0.15944602	-0.2152165	0.8614169	-2.4336435	0.18509739	9.22560913	11.8744691	11.6592527
A_55_P2353	AK048022	C130030K03	Mus musculu	chr10:48813	6.94402544	1.82668318	3.54720617	-0.2984915	0.81310212	2.12517472	4.36255924	8.26131141	6.43462823	6.13613669
A_51_P2103	NM_182959	Slc17a8	Mus musculu	chr10:89046	8.28437051	-3.7384797	0.07492133	-2.6903472	0.15492617	-1.0481325	0.48359375	6.6888331	10.4273128	7.73696559
A_55_P2030	NM_001142	Tcf7l2	Mus musculu	chr19:55994	8.32241863	-2.6172114	0.16298246	-2.0908838	0.23473684	-0.5263276	0.69431988	7.27457229	9.8917837	7.8008999
A_52_P2494	NM_001025	Vegfa	Mus musculu	chr17:46162	10.9452197	-2.7968005	0.14390608	-0.4045409	0.75547666	-2.3922596	0.19048382	9.215533	12.0123335	11.6077926
A_30_P0102	chr16:46384974-46391331	lincRNA:chr1	chr16:46387	8.71596012	-2.8250208	0.14111852	-1.5306076	0.34613156	-1.2944132	0.40770197	7.34281546	10.1678363	8.63722864	
A_55_P2406	AK015759	4930511J24R	Mus musculu	chr12:11115	6.02256988	-0.0597062	0.95945946	2.49905578	5.65315315	-2.558762	0.16972112	5.14974712	5.20945337	7.70850915
A_55_P2101	NM_146350	Olfr1123	Mus musculu	chr2:872591	6.86426262	-0.1262638	0.91620112	3.0224918	8.12569832	-3.1487556	0.11275352	5.7725895	5.89885328	8.92134508
A_55_P2037	XR_031802	Gm13549	PREDICTED: I	chr2:592375	7.94398996	-2.4787843	0.17939552	-0.6962845	0.61715957	-1.7824997	0.2906793	6.52356196	9.00234623	8.30606169
A_55_P1954	NM_009311	Tac1	Mus musculu	chr6:751289	9.68496268	4.33385625	20.1660448	2.27765173	4.8488806	2.05620452	4.15890727	11.8149829	7.48112669	9.75877842
A_55_P2141	NM_001136	Ldha	Mus musculu	chr7:541030	10.8338072	-3.594391	0.0827905	-1.0787237	0.47344748	-2.5156673	0.17486733	8.79712109	12.3915121	11.3127884
A_55_P2091	NM_007987	Fas	Mus musculu	chr19:34402	6.47343313	-2.0941449	0.23420683	-1.8820769	0.27129289	-0.212068	0.86329884	5.70469547	7.7988404	5.91676351
A_51_P4987	NM_009784	Cacna2d1	Mus musculu	chr5:158765	11.1533966	-4.1801131	0.05516461	-1.6161975	0.32619408	-2.5639156	0.16911592	8.90538701	13.0855001	11.4693026
A_51_P1876	NM_028946	Nhedc1	Mus musculu	chr3:135060	7.60969649	-4.7193117	0.0379617	-3.9927816	0.0628135	-0.7265301	0.60435572	5.79441587	10.5137276	6.52094601
A_51_P3588	NM_028417	Ttc9b	Mus musculu	chr7:284409	9.3223318	2.09414974	4.26974448	1.37812489	2.59930314	0.71602484	1.64264969	10.2590567	8.16490693	9.54303182

A_55_P2141(	NM_010165	Eya2	Mus musculu	chr2:165597(	9.29331552	-2.4723336	0.18019943	-1.3601331	0.38954635	-1.1122006	0.4625879	8.09847079	10.5708044	9.21067134
A_30_P0101¦	chr2:128211990-12821522	lincRNA:chr2:	chr2:1282122	7.49578356	0.16965466	1.12478921	2.43691866	5.4148398	-2.267264	0.20772345	6.79658045	6.62692579	9.06384445
A_55_P2035(	NM_027154	Tmbim1	Mus musculu	chr1:7433489	8.70371394	-2.1017403	0.23297704	-0.9401471	0.52117973	-1.1615932	0.44701861	7.6159361	9.71767642	8.77752931
A_66_P1380(	NR_033456	Gm4710	Mus musculu	chr17:762882	8.4423003	-4.8379999	0.03496366	-5.0214117	0.03078963	0.1834118	1.13556619	6.89077093	11.7287708	6.70735913
A_51_P4486(	NM_011539	Tbxas1	Mus musculu	chr6:3903435	6.46514752	-0.5495882	0.68321513	1.52637668	2.88061466	-2.0759649	0.23717686	5.58996318	6.13955135	7.66592804
A_55_P2152(	NM_009576	Zic4	Mus musculu	chr9:9128292	7.35032495	2.33988719	5.06263048	0.75432964	1.6868476	1.58555755	3.00123762	8.65880653	6.31891935	7.07324898
A_55_P2082:	XM_905410	LOC631287	PREDICTED: M	chr7:1481419	9.97575369	-1.3260069	0.39887071	0.74662495	1.67786304	-2.0726319	0.23772543	8.8428741	10.168881	10.915506
A_55_P2088!	NM_0011629	Rpl10l	Mus musculu	chr12:673844	6.75535185	-2.1727745	0.22178374	-0.7278849	0.60378848	-1.4448896	0.36732026	5.54946382	7.72223831	6.99435344
A_51_P2791(	NM_008969	Ptgs1	Mus musculu	chr2:3610764	7.50928541	3.07238063	8.41160221	1.71082546	3.27348066	1.36155517	2.56962025	8.98726401	5.91488339	7.62570884
A_55_P2279!	AK148051	5930430L01R	Mus musculu	chr5:1498017	6.99165636	0.0439777	1.03095238	2.54314233	5.82857143	-2.4991646	0.17687908	6.17326071	6.12928302	8.67242534
A_30_P0102!	chr18:35204950-35216650	lincRNA:chr1:	chr18:352139	6.50331656	0.01917231	1.01337793	2.57365985	5.95317726	-2.5544875	0.17022472	5.65821148	5.63903917	8.21269903
A_30_P0102(	chr17:36131460-36136694	lincRNA:chr1'	chr17:361358	8.92775735	2.406116	5.30045425	0.3630901	1.28617781	2.0430259	4.12108981	10.4108047	8.00468865	8.36777875
A_55_P2046:	NM_008239	Foxq1	Mus musculu	chr13:316522	7.72839969	-3.1488126	0.11274907	-0.6863691	0.62141584	-2.4624435	0.181439	5.857981	9.00679358	8.3204245
A_55_P2053:	NM_0011601	Foxg1	Mus musculu	chr12:504877	13.3626396	3.75503637	13.5013931	2.65967139	6.31889106	1.09536498	2.1366713	14.9794401	11.2244037	13.8840751
A_51_P3245:	NM_029755	Calcoco2	Mus musculu	chr11:959612	7.20937048	-0.441227	0.73650794	1.92660192	3.8015873	-2.3678289	0.19373695	6.27301849	6.71424552	8.64084744
A_55_P1965:	NM_146939	Olfr354	Mus musculu	chr2:3676336	6.44290925	-0.2599184	0.83513514	1.74938931	3.36216216	-2.0093077	0.24839228	5.68650053	5.94641896	7.69580827
A_51_P1264:	NM_007930	Enc1	Mus musculu	chr13:980227	11.9785801	2.12751777	4.36965012	1.90931652	3.75631101	0.21820125	1.1632823	12.7604864	10.6329687	12.5422852
A_30_P0103:	chr1:183951156-18398615	lincRNA:chr1:	chr1:1839583	6.95615852	-0.5441827	0.68577982	2.86299273	7.27522936	-3.4071754	0.0942623	5.63903917	6.18322182	9.04621455
A_55_P2121:	XM_975980	Gm7578	PREDICTED: M	chr7:7457398	7.28382424	0.1175696	1.08490566	2.33924497	5.06037736	-2.2216754	0.21439224	6.58245565	6.46488605	8.80413102
A_51_P1050(	NM_027990	Lypd6b	Mus musculu	chr2:4980419	8.82989747	2.83120661	7.11669108	2.6561536	6.30350216	0.17505302	1.12900589	9.83198401	7.0007774	9.656931
A_55_P2126(	XM_0014726	Gm2160	PREDICTED: M	chr6:3588291	6.15188879	-0.0981804	0.93421053	2.81006421	7.01315789	-2.9082446	0.13320826	5.14974712	5.24792751	8.05799172
A_51_P2462:	NM_177409	Tram2	Mus musculu	chr1:2099154	7.19158191	-2.618225	0.162868	-1.173011	0.44349478	-1.445214	0.36723769	5.83710227	8.45532722	7.28231624
A_55_P2123(	NM_0010335	Gm5127	Mus musculu	chrX:1039058	7.25122023	-0.0497982	0.96607143	2.17049739	4.50178571	-2.2202956	0.21459738	6.49452228	6.54432052	8.7148179
A_66_P1231!	NM_027442	Ddo	Mus musculu	chr10:403696	6.56175003	-0.4867602	0.71362587	1.65222814	3.14318707	-2.1389883	0.22703894	5.68650053	6.17326071	7.82548885
A_30_P0102(	chr1:162965309-16297070	lincRNA:chr1:	chr1:1629676	9.66257131	2.16583086	4.48724781	0.49916911	1.41339931	1.66666175	3.17479127	10.9400688	8.77423798	9.2734071
A_55_P2083(	NM_015814	Dkk3	Mus musculu	chr7:1192596	8.98961296	-4.3796504	0.04803899	-3.1160353	0.11533999	-1.2636151	0.41649899	7.10852446	11.4881749	8.37213954
A_30_P0103:	chr14:55433382-55452757	lincRNA:chr1·	chr14:554454	9.74724788	-2.5210394	0.1742174	-1.3847206	0.38296366	-1.1363188	0.45491887	8.52812848	11.0491679	9.66444728
A_55_P1973:	NM_001038(	Mapk1	Mus musculu	chr16:170390	9.56263258	2.18108697	4.53495102	0.86227164	1.81789849	1.31881533	2.49461181	10.7292667	8.54817971	9.41045135
A_55_P2067:	XM_983011	LOC675032	PREDICTED: M	chr3:3646508	9.19031363	-1.4006188	0.37876664	-2.4647491	0.18114926	1.06413034	2.09090909	9.07815081	10.4787696	8.01402047
A_51_P2679:	NM_175692	Snhg11	Mus musculu	chr2:1582115	12.7893646	-2.3834991	0.19164402	0.88982663	1.85295344	-3.2733257	0.10342625	10.9037563	13.2872555	14.1770821
A_66_P1057:	XM_0014742	LOC669999	PREDICTED: M	chr18:343694	6.77738731	0.10509706	1.07556675	2.08296679	4.23677582	-1.9778697	0.25386445	6.15312976	6.0480327	8.13099949
A_30_P0102·	chr2:173105462-17312618	lincRNA:chr2:	chr2:1731149	6.38903236	-0.3654196	0.77624309	1.78786649	3.45303867	-2.1532861	0.2248	5.54946382	5.91488339	7.70274988
A_52_P1747:	NM_176950	Defb20	Mus musculu	chr2:1523051	6.43269268	-0.5657379	0.67560976	1.58026316	2.9902439	-2.1460011	0.22593801	5.52877967	6.0945176	7.67478076
A_30_P0102:	chr5:31862823-31871445_	lincRNA:chr5:	chr5:3187130	7.56521545	-0.0253111	0.9826087	2.1844873	4.54565217	-2.2097984	0.21616451	6.82017896	6.84549005	9.02997735
A_51_P3038(	NM_0011644	Klhl29	Mus musculu	chr12:508443	9.05358565	2.02404742	4.06723238	1.14799528	2.21605744	0.87605214	1.8353461	10.0202855	7.99623808	9.14423336
A_30_P0101:	chr17:39980973-39981876	lincRNA:chr1'	chr17:399814	11.2459779	-0.6869462	0.62116732	2.70962202	6.54150241	-3.3965682	0.0949579	9.88480649	10.5717526	13.2813747
A_52_P3007:	NM_010441	Hmga2	Mus musculu	chr10:119798	8.38684947	-1.4848621	0.35728269	-2.5553304	0.17012529	1.07046838	2.10011507	8.24871825	9.73358031	7.17824987
A_30_P0102!	chr14:99728359-99728958	lincRNA:chr1·	chr14:997286	8.07006715	1.91368908	3.767713	-0.3170841	0.80269058	2.2307732	4.69385475	9.45155457	7.53786549	7.22078137
A_55_P2064!	BC022182	Tuba1c	Mus musculu	chr15:988684	11.652871	-2.276377	0.20641547	-2.221389	0.2144348	-0.054988	0.96260246	10.8757494	13.1521264	10.9307373
A_51_P2624:	NM_009215	Sst	Mus musculu	chr16:238898	13.2054172	1.42051763	2.67681537	2.41081828	5.31775859	-0.9903007	0.50337286	13.3488229	11.9283052	14.3391235
A_55_P2040!	NM_009608	Actc1	Mus musculu	chr2:1138731	6.24716728	-2.4549789	0.18238022	-2.0614975	0.23956723	-0.3934814	0.76129032	5.29768055	7.7526594	5.6911619
A_30_P0102(	H19		lincRNA:chr7:	chr7:1497622	11.6317579	-5.7717701	0.01830308	-1.7384809	0.29968507	-4.0332892	0.06107436	8.36340473	14.1351749	12.396694
A_52_P3294!	NM_010777	Mbp	Mus musculu	chr18:827422	8.60894464	-3.9038581	0.06680694	-2.1578332	0.22409258	-1.7460249	0.29812207	6.72565028	10.6295084	8.47167521
A_30_P0102(	chr12:53669892-53710742	lincRNA:chr1:	chr12:536699	8.03912309	1.07125322	2.10125786	-1.1034296	0.46540881	2.17468281	4.51486486	9.12110177	8.04984855	6.94641896

A_65_P06147	NM_008010	Fgfr3	Mus musculu	chr5:3407785	10.2977291	-2.1577685	0.22410263	-1.2518864	0.41989882	-0.9058822	0.53370625	9.27651217	11.4342807	10.1823944
A_52_P16428	NM_0010386	Dnm3	Mus musculu	chr1:1639205	8.88045948	2.03570561	4.1002322	1.35438927	2.55688854	0.68131634	1.60360224	9.78613347	7.75042785	9.10481713
A_55_P2207(	BY715827	4930564G21I	BY715827 RIKEN full-length		6.42507839	-0.3249202	0.79834254	1.85550521	3.61878453	-2.1804254	0.22061069	5.58996318	5.91488339	7.7703886
A_51_P44584	NM_145470	Depdc6	Mus musculu	chr15:550837	7.17367184	-1.4227898	0.37299035	1.10149299	2.14576635	-2.5242828	0.17382617	5.857981	7.28077077	8.38226376
A_55_P20056	NM_030207	Sfi1	Mus musculu	chr11:303191	12.8192497	-1.1796463	0.44145973	1.08361233	2.11933599	-2.2632586	0.20830096	11.6716148	12.851261	13.9348734
A_55_P20282	NM_172572	Rhbdf2	Mus musculu	chr11:116459	6.91032756	-2.1852042	0.21988115	-1.8911932	0.26958401	-0.294011	0.81563126	6.08392248	8.26912668	6.3779335
A_52_P62806	NM_013538	Cdca3	Mus musculu	chr6:1247832	7.99008149	-1.1787349	0.44173868	-2.7930707	0.14427861	1.61433575	3.06170599	8.13528176	9.3140167	6.52094601
A_30_P01031	chr14:33895294-33923644	lincRNA:chr14	chr14:339124		9.05737672	-0.215001	0.86154554	1.88361003	3.6899724	-2.0986111	0.23348292	8.28617268	8.50117372	10.3847838
A_55_P1988(	XM_0014760	Gm3249	PREDICTED: M	chr4:5399988	6.3965661	-0.4554518	0.72928177	1.90049991	3.73342541	-2.3559517	0.19533851	5.45943162	5.91488339	7.8153833
A_51_P22960	NM_008882	Plxna2	Mus musculu	chr1:1966429	12.209932	2.67674581	6.39411995	0.79161193	1.73100745	1.88513388	3.69387201	13.7305586	11.0538128	11.8454247
A_30_P01024	chr15:99762797-99786472	lincRNA:chr15	chr15:997640		6.82669443	-0.5373968	0.68901304	1.56603277	2.96089385	-2.1034296	0.2327044	5.94641896	6.48381578	8.04984855
A_52_P2333(	NM_175501	Adamts12	Mus musculu	chr15:112660	8.15936791	-4.9945208	0.03136891	-2.9586887	0.12863109	-2.0358321	0.24386724	5.81591694	10.8104377	7.85174904
A_51_P12887	NM_025378	Ifitm3	Mus musculu	chr7:1481956	9.21030402	-3.7866602	0.07246056	-2.1508015	0.22518747	-1.6358586	0.32177885	7.40279777	11.1894579	9.03865638
A_55_P19671	NM_008393	Irx3	Mus musculu	chr8:9432253	8.63856185	-6.7291461	0.00942595	-3.1709667	0.11103091	-3.5581794	0.08489484	5.20945337	11.9385995	8.76763273
A_55_P21848	NM_146605	Olfr828	Mus musculu	chr9:1861985	7.45649461	-1.2924843	0.40824742	1.65133285	3.14123711	-2.9438172	0.1299639	6.04439412	7.33687844	8.98821128
A_30_P01032	chr10:69667411-69678033	lincRNA:chr10	chr10:696779		7.07150848	-0.2351846	0.84957627	2.55666841	5.88347458	-2.791853	0.14440043	6.06249593	6.29768055	8.85434896
A_55_P19914	XM_0014782	Gm3845	PREDICTED: M	chr16:571832	7.04942507	-0.3283831	0.79642857	1.84369677	3.58928571	-2.1720799	0.22189055	6.2159374	6.54432052	8.38801729
A_55_P20312	NM_0010396	Lhx3	Mus musculu	chr2:2605580	7.37149812	-3.3729181	0.09652737	-1.9491104	0.25897587	-1.4238077	0.37272727	5.7725895	9.14550764	7.19639721
A_55_P19758	XR_030977	Gm8144	PREDICTED: M	chrX:5767931	9.74973718	-3.4273002	0.09295652	-1.4737912	0.36003494	-1.953509	0.25818749	7.95613411	11.3834343	9.90964311
A_55_P19998	NM_011998	Chst4	Mus musculu	chr8:1125532	8.19991437	0.05057536	1.03567788	2.48972728	5.61671764	-2.4391519	0.18439201	7.40372219	7.35314683	9.8428741
A_55_P19849	NM_146923	Olfr20	Mus musculu	chr11:731681	6.43840345	-0.5344587	0.69041769	1.59790156	3.02702703	-2.1323602	0.22808442	5.54946382	6.08392248	7.68182404
A_55_P19763	NM_020496	Tbx20	Mus musculu	chr9:2454477	6.36447049	-0.0742386	0.94984326	1.97029977	3.9184953	-2.0445384	0.2424	5.65821148	5.73245011	7.70274988
A_55_P20157	NM_029815	Bcas1	Mus musculu	chr2:1701727	9.24296145	-3.7318106	0.07526847	-2.4802217	0.17921687	-1.251589	0.41998539	7.58182825	11.3136389	8.83341722
A_55_P20566	NM_145588	Kif22	Mus musculu	chr7:1341713	8.34614058	-1.3404022	0.39491055	-2.9211769	0.13201952	1.5807747	2.99130435	8.42626475	9.76666693	6.84549005
A_55_P20501	XM_990857	LOC676534	PREDICTED: M	chr4:1205375	8.86879212	-1.5901311	0.33214126	2.56934637	5.93540456	-4.1594775	0.05595933	6.9522559	8.54238704	11.1117334
A_66_P13609	NM_0011642	Trim13	Mus musculu	chr14:622244	9.6518893	2.12676315	4.36736514	1.19015053	2.28176549	0.93661263	1.91402892	10.6730146	8.5462514	9.73640193
A_55_P2113(	NM_153177	Eif2c4	Mus musculu	chr4:1261695	8.52882707	2.04910653	4.1384959	0.11853141	1.08562919	1.93057511	3.81207133	9.85538762	7.80628109	7.9248125
A_55_P19796	NM_011309	S100a1	Mus musculu	chr3:9031525	8.83473596	-2.8151494	0.14208741	0.20159631	1.14997007	-3.0167457	0.12355748	6.89077093	9.70592032	9.90751663
A_30_P01033	chr2:77807600-77836075_	lincRNA:chr2:	chr2:7781927		6.94556954	-0.6331908	0.64474886	1.93464144	3.82283105	-2.5678322	0.16865743	5.87856187	6.51175265	8.4463941
A_55_P20996	ENSMUST00000115332		G protein-cou	chr6:2561695	6.5697468	-3.1938088	0.10928681	-2.1276185	0.2288353	-1.0661903	0.47757848	5.14974712	8.34355587	6.2159374
A_55_P2088(	NM_0011643	Gm6904	Mus musculu	chr14:598773	6.24495636	-0.160177	0.89491525	2.03622019	4.10169492	-2.1963972	0.21818182	5.45943162	5.61960864	7.65582883
A_55_P19863	NM_007960	Etv1	Mus musculu	chr12:395946	8.35995522	3.26676888	9.62488219	-0.5856425	0.6663525	3.85241142	14.4441301	10.7330153	7.46624644	6.8806039
A_51_P31504	NM_016847	Avpr1a	Mus musculu	chr10:121890	7.51554988	2.15727474	4.46071429	0.75641336	1.68928571	1.40086139	2.64059197	8.70159526	6.54432052	7.30073387
A_66_P13477	NM_008961	Pter	Mus musculu	chr2:1291642	6.50574669	-2.352368	0.19582433	-1.694997	0.30885529	-0.657371	0.63403263	5.50250034	7.85486838	6.15987134
A_55_P22342	NR_030701	D430050G20	Mus musculu	chr13:727610	6.67022525	-2.4686062	0.18066561	-0.6703191	0.62836767	-1.798287	0.28751576	5.24792751	7.71653369	7.04621455
A_51_P10588	NM_172842	Lax1	Mus musculu	chr1:1355761	7.380323	0.31762638	1.2462784	2.07161437	4.20356789	-1.753988	0.29648109	6.9015358	6.58390942	8.65552379
A_51_P31655	NM_010612	Kdr	Mus musculu	chr5:7632936	7.06889419	1.8403178	3.580889	-0.9214576	0.52797532	2.76177541	6.78230381	8.60292526	6.76260746	5.84114984
A_51_P25788	NM_175217	Mmd2	Mus musculu	chr5:1430399	8.33268688	-2.2544559	0.20957581	-1.6446517	0.3198236	-0.6098042	0.65528562	7.3779335	9.6323894	7.98773773
A_55_P20894	NM_0011634	1700020A23F	Mus musculu	chr2:1302317	6.79015133	-0.3514724	0.78378378	2.09356627	4.26801802	-2.4450386	0.18364116	5.857981	6.20945337	8.30301963
A_55_P21652	NM_0010819	2300005B03F	Mus musculu	chr15:745733	9.88010784	0.01186659	1.00825921	-2.2322025	0.21283355	2.24406913	4.73731343	10.6320864	10.6202198	8.38801729
A_30_P01031	chr10:120058696-1200761	lincRNA:chr10	chr10:120069		6.11497851	-0.1655861	0.89156627	2.38540331	5.2248996	-2.5509894	0.17063797	5.20945337	5.37503943	7.76044275
A_51_P46928	NM_008737	Nrp1	Mus musculu	chr8:1310266	9.24878535	1.53303471	2.89393939	2.2643406	4.80434783	-0.7313059	0.60235843	9.51602828	7.98299357	10.2473342
A_55_P21517	NM_146542	Olfr11	Mus musculu	chr13:217305	5.93523867	0.21730713	1.16256158	2.34728862	5.08866995	-2.1299815	0.22846079	5.29768055	5.08037342	7.42766204
A_55_P20128	NM_181819	Wfikkn2	Mus musculu	chr11:940989	6.56221318	-2.0931896	0.23436197	-1.1481312	0.45120934	-0.9450585	0.5194085	5.54946382	7.64265344	6.49452228

A_51_P3016(	XM_979851	AI854517	PREDICTED: M	chr7:866772	10.695849	2.57860765	5.97362906	-0.1861091	0.87897311	2.76471673	6.79614544	12.4769571	9.89834945	9.71224038
A_52_P4814	NM_030249	Cttnbp2nl	Mus musculu	chr3:1048051	10.5734264	2.16570782	4.48686515	1.41535329	2.66725044	0.75035453	1.68220617	11.5454472	9.37973936	10.7950926
A_30_P0102	NEAT1		lincRNA:chr1	chr19:58425	8.05523824	-2.583273	0.16686196	-1.673144	0.31356925	-0.910129	0.53213752	6.89077093	9.47404389	7.8008999
A_52_P4250(	NR_033609	A930017M01	Mus musculu	chr15:44715	7.45952055	-0.4634546	0.72524752	1.62226933	3.07858911	-2.085724	0.23557789	6.60979435	7.07324898	8.69551831
A_52_P9252	NM_001079	Bcl11b	Mus musculu	chr12:10914	10.5565633	4.54724217	23.3806344	2.44321108	5.43850863	2.10403109	4.29908933	12.7736544	8.22641219	10.6696233
A_30_P0103	chr8:67552315-67561235_		lincRNA:chr8	chr8:675524	8.11403105	-3.5242899	0.08691265	-2.6596418	0.15825886	-0.8646481	0.54918033	6.65105169	10.1753416	7.51569984
A_51_P2672	NM_019980	Litaf	Mus musculu	chr16:10959	8.65403287	-2.1610347	0.22359585	-2.1551381	0.2245116	-0.0058966	0.99592114	7.93172245	10.0927571	7.93761903
A_55_P2072	NM_010833	Msn	Mus musculu	chrX:933638	10.2460652	-3.327799	0.09959389	-2.1535849	0.22475343	-1.174214	0.44312511	8.74539422	12.0731932	9.91960824
A_55_P2180	XM_0014764	LOC1000466(	PREDICTED: Mus musculus		6.03905954	-0.2279519	0.85384615	2.03291462	4.09230769	-2.2608666	0.20864662	5.20945337	5.43740531	7.47031993
A_51_P1101	NM_053273	Ttyh2	Mus musculu	chr11:11457	6.85453513	-2.2283494	0.21340273	-1.210862	0.43201041	-1.0174874	0.4939759	5.7725895	8.00093895	6.79007693
A_30_P0103(	chr12:33604247-33638654		lincRNA:chr1	chr12:33604	7.73669192	-0.8344949	0.56077933	1.1839112	2.27191868	-2.0184061	0.24683072	6.78572491	7.62021983	8.80413102
A_66_P1180	XM_0014804	LOC1000486	PREDICTED: M	chr2:119146	7.2742942	-2.3554086	0.19541206	-1.6695171	0.31435854	-0.6858914	0.62162162	6.26052755	8.6159361	6.94641896
A_51_P3510	NM_010735	Lta	Mus musculu	chr17:35340	7.03788484	0.5153975	1.42938792	2.19500545	4.57891393	-1.6796079	0.31216746	6.64981469	6.13441718	8.32942263
A_55_P2397	AK047130	B930025B16	Mus musculu	chr14:12074(	9.17188566	-1.2508552	0.42019904	-2.4505078	0.18294631	1.19965257	2.29684352	9.15481811	10.4056733	7.95516554
A_52_P8439	XM_914998	Gm9804	PREDICTED: M	chr12:50504	7.75085133	2.6889285	6.44834308	1.31006794	2.47953216	1.37886056	2.60062893	9.10678102	6.41785251	7.72792045
A_55_P2128	NM_020567	Gmnn	Mus musculu	chr13:24843	7.95448519	-0.6998499	0.61563624	-2.4155095	0.18743867	1.71565958	3.28446771	8.29308841	8.99293834	6.57742883
A_55_P2002	NM_145921	Olah	Mus musculu	chr2:325934	7.90139454	0.21506908	1.16075949	2.3668755	5.15822785	-2.1518064	0.22503067	7.25581542	7.04074634	9.40762184
A_51_P3747	NM_008987	Ptx3	Mus musculu	chr3:660292	8.95313578	-5.1647304	0.02787798	-2.9536203	0.12908379	-2.2111101	0.21596806	6.49452228	11.6592527	8.70563239
A_52_P2164	NM_009385	Nkx2-1	Mus musculu	chr12:57633	11.7039479	2.84492818	7.18470132	1.57521267	2.97979414	1.26971551	2.41114016	13.0754958	10.2305676	11.8057802
A_55_P1995	NM_011623	Top2a	Mus musculu	chr11:98855	7.20021032	-1.2522191	0.41980198	-2.3326812	0.19851485	1.08046203	2.11471322	7.14295795	8.39517708	6.06249593
A_55_P2058	NM_011562	Tdgf1	Mus musculu	chr9:110842	6.16237588	-2.1060416	0.23228346	-1.6179973	0.3257874	-0.4880444	0.71299094	5.29768055	7.40372219	5.78572491
A_30_P0102	chr16:46384974-46391331		lincRNA:chr1(	chr16:46388	8.23687244	-2.7930286	0.14428281	-2.3297582	0.19891746	-0.4632704	0.72534014	7.15143943	9.94446805	7.61470984
A_55_P2080	NM_008301	Hspa2	Mus musculu	chr12:77507	9.05355555	-2.3231284	0.19983367	-1.8785887	0.27194963	-0.4445398	0.7348187	8.13099949	10.4541279	8.57553925
A_55_P1953	ENSMUST00000084696		spondin 1, (f-	chr7:121073	8.5422754	-2.624722	0.16213619	-2.48511	0.17861065	-0.139612	0.90776326	7.62083075	10.2455527	7.76044275
A_66_P1311	ENSMUST00000098303		Putative unch	chr7:100201(	6.90015194	-0.903681	0.53452116	1.17242194	2.25389755	-2.076103	0.23715415	5.9068906	6.81057163	7.98299357
A_51_P2655	NM_026434	Rbm18	Mus musculu	chr2:3597841	12.924952	2.05337309	4.15075302	0.321093	1.24927665	1.73228009	3.32252509	14.1868364	12.1334633	12.4545563
A_55_P1955	NM_010345	Grb10	Mus musculu	chr11:11833	11.0764329	-2.3211364	0.20010978	-1.6978463	0.30824591	-0.6232901	0.64918876	10.0949574	12.4160938	10.7182474
A_52_P1092	NM_173379	Leprel1	Mus musculu	chr16:25968	7.30526559	-1.3877089	0.38217123	0.62059676	1.53751103	-2.0083057	0.24856487	6.17326071	7.56096965	8.18156641
A_52_P6057	NM_018763	Chst2	Mus musculu	chr9:953019	9.04381457	2.22899734	4.6880805	1.6511628	3.14086687	0.57783455	1.4926072	9.9794252	7.75042785	9.40159065
A_51_P4647	NM_011400	Slc2a1	Mus musculu	chr4:118809	10.1503009	-3.0785325	0.11837756	-2.0666049	0.23872063	-1.0119276	0.49588323	8.78681414	11.8653466	9.79874179
A_55_P2160	NM_008593	Foxd2	Mus musculu	chr4:114579(	6.92014593	-1.764683	0.29429134	0.31395424	1.24311024	-2.0786372	0.23673793	5.63903917	7.40372219	7.71767642
A_55_P2145	NR_015386	Six3os1	Mus musculu	chr17:86010	8.20928279	-1.9710075	0.25507484	-2.4015914	0.18925569	0.43058393	1.34777898	7.69580827	9.66681576	7.26522434
A_55_P2254	AK137666	4931402G19	Mus musculu	chr2:1202981	8.28762175	0.10567311	1.07599629	2.28564219	4.87581094	-2.1799691	0.22068048	7.59618976	7.49051665	9.77615883
A_55_P2067	NM_054055	Slc13a3	Mus musculu	chr2:165230	6.59619422	-1.8048208	0.2862166	-2.0728263	0.23769339	0.26800555	1.20414201	6.08392248	7.88874325	5.81591694
A_55_P1957	NM_172294	Sulf1	Mus musculu	chr1:128495(	7.58271436	-3.2534193	0.10486322	-2.0842689	0.2358156	-1.1691504	0.44468314	6.10852446	9.36194377	7.27767486
A_51_P1494	NM_007381	Acadl	Mus musculu	chr1:668777	6.72270416	-2.2208895	0.21450907	-1.7849732	0.29018136	-0.4359162	0.73922414	5.83710227	8.05799172	6.27301849
A_30_P0103	chr12:111322030-1113233		lincRNA:chr1	chr12:11132	6.46576899	0.10153803	1.07291667	2.54088143	5.81944444	-2.4393434	0.18436754	5.68650053	5.5849625	8.12584393
A_30_P0102	chr1:135956616-13595748		lincRNA:chr1	chr1:135956	6.87296989	-1.7169477	0.30419162	-2.0261523	0.24550898	0.30920459	1.23902439	6.40372219	8.12066989	6.0945176
A_30_P0103	chr2:157361470-15737981		lincRNA:chr2	chr2:157371	8.71491935	-1.3103633	0.40321932	1.37291847	2.58993964	-2.6832818	0.15568676	7.38370429	8.69406764	10.0669861
A_55_P2065	ENSMUST00000066038		RIKEN cDNA (	chr2:147014(	7.80457043	-2.9025122	0.1337386	-1.3930152	0.38076817	-1.5094969	0.35123367	6.33390074	9.23641289	7.84339767
A_55_P2106(	NM_016693	Map3k6	Mus musculu	chr4:132808	7.61224504	-1.10124	0.4661157	0.97048862	1.95950413	-2.0717286	0.23787431	6.55458885	7.65582883	8.62631745
A_55_P2059	NM_001163	Prom1	Mus musculu	chr5:443849	11.9204067	-1.1449983	0.45219023	-2.9333386	0.13091128	1.78834037	3.45417307	12.1348541	13.2798524	10.3465137
A_30_P0102	XIST		lincRNA:chrX	chrX:100655	7.95989657	5.28956857	39.1127907	0.10043009	1.07209302	5.18913848	36.4826464	11.4527989	6.16323035	6.26366044
A_30_P0103	chr2:130697320-13072967		lincRNA:chr2	chr2:130727	7.58563564	-0.0842982	0.94324324	2.00194827	4.00540541	-2.0862465	0.23549258	6.86212073	6.94641896	8.94836723

A_55_P1954	NM_0011595	Esrrb	Mus musculu	chr12:87862	7.97543548	-2.9343406	0.1308204	-0.8354163	0.56042129	-2.0989242	0.23343225	6.29768055	9.23202112	8.39660478
A_55_P2147	NM_0010253	Fam196b	Mus musculu	chr11:34322	9.08361468	2.44013106	5.4269103	1.86474145	3.64202658	0.57538961	1.49007982	10.0887882	7.64865718	9.51339863
A_55_P2053	ENSMUST00000110746		forkhead box	chr12:50495	7.27175325	2.0195528	4.0545809	0.54214942	1.45614035	1.47740338	2.78447122	8.43740531	6.41785251	6.96000193
A_55_P1977	NM_0010117	Olfr1089	Mus musculu	chr2:865728	6.47499887	-0.1049696	0.92982456	2.03129613	4.0877193	-2.1362657	0.22746781	5.72792045	5.83289001	7.86418614
A_55_P2027	NM_030258	Gpr146	Mus musculu	chr5:139872	8.26898946	-2.3571103	0.19518171	-1.854965	0.27643936	-0.5021452	0.70605613	7.31590431	9.67301456	7.81804952
A_30_P0101	chr1:182808654-18281565		lincRNA:chr1	chr1:182808	6.46528534	-0.4643138	0.72481572	1.60840242	3.04914005	-2.0727163	0.23771152	5.61960864	6.08392248	7.6923249
A_52_P4958	NM_010658	Mafb	Mus musculu	chr2:160189	11.1721273	2.09741113	4.27940771	1.94847091	3.85965235	0.14894023	1.10875471	11.9209111	9.82349993	11.7719708
A_52_P3736	NM_177049	Jph4	Mus musculu	chr14:55726	11.1439332	0.76873339	1.7037733	2.31923626	4.99067949	-1.5505029	0.34139105	10.8833433	10.11461	12.4338462
A_30_P0102	chr2:157361470-15737981		lincRNA:chr2	chr2:157371	10.1127062	-1.3919172	0.38105808	1.31810146	2.49337773	-2.7100186	0.15282806	8.74539422	10.1373114	11.4554128
A_52_P4204	NM_175660	Hist1h2ab	Mus musculu	chr13:23843	10.5524251	-1.7030812	0.30712947	-2.1879565	0.21946207	0.48487531	1.39946491	10.1463565	11.8494377	9.66148122
A_55_P2072	NM_177782	Prex1	Mus musculu	chr2:166391	8.4987067	-2.1655351	0.22289943	-1.0800627	0.47300827	-1.0854725	0.47123792	7.4150375	9.58057264	8.50050996
A_55_P2013	NM_023132	Renbp	Mus musculu	chrX:711677	9.25782431	-4.2911131	0.05107945	-1.0196501	0.49323596	-3.271463	0.10355987	6.73696559	11.0280787	10.0084286
A_51_P1917	NM_029946	Efcab6	Mus musculu	chr15:83697	6.93253801	2.14411582	4.42021277	0.74461917	1.67553191	1.39949665	2.63809524	8.11374217	5.96962635	6.71424552
A_30_P0103	chr2:157361470-15737981		lincRNA:chr2	chr2:157371	8.20686462	-1.5305147	0.34615385	1.0043173	2.00599401	-2.534832	0.17255976	6.85174904	8.38226376	9.38658105
A_55_P2032	XM_893730	Gm6934	PREDICTED: I	chr17:51973	8.48211977	1.84827358	3.60069045	2.06333612	4.17951669	-0.2150625	0.86150881	9.02652344	7.17824987	9.24158599
A_55_P2166	NM_009851	Cd44	Mus musculu	chr2:102654	8.58569034	-2.1162746	0.23064172	-0.5039245	0.70518589	-1.6123501	0.32706514	7.34281546	9.45909003	8.95516554
A_30_P0102	chr12:4133875-4243375_R		lincRNA:chr1	chr12:41514	6.67938257	-0.5245035	0.69519833	1.60589316	3.04384134	-2.1303966	0.22839506	5.79441587	6.31891935	7.9248125
A_51_P3739	NM_025290	Rsph1	Mus musculu	chr17:31410	7.92981154	-2.4983434	0.1769798	-0.777938	0.58319974	-1.7204054	0.30346344	6.52356196	9.02190534	8.24396733
A_30_P0102	chr18:75132417-75141201		lincRNA:chr1	chr18:75134	6.35965162	-0.0715533	0.9516129	2.07702242	4.21935484	-2.1485757	0.22553517	5.61960864	5.6911619	7.76818432
A_55_P1996	NM_008010	Fgfr3	Mus musculu	chr5:340796	11.7094443	-2.0580114	0.24014681	-1.6072531	0.32822269	-0.4507583	0.73165816	10.8731877	12.9311992	11.3239461
A_51_P1609	NM_008209	Mr1	Mus musculu	chr1:156975	6.24074978	-2.290978	0.20433695	-1.914733	0.26522102	-0.376245	0.77044025	5.35167544	7.64265344	5.72792045
A_55_P2046	NM_016966	Phgdh	Mus musculu	chr3:981172	8.63707335	-2.4977052	0.17705811	-1.9061812	0.26679782	-0.5915239	0.66364152	7.60733031	10.1050355	8.19885426
A_30_P0103	chr4:88604248-88645948_		lincRNA:chr4	chr4:886417	7.39118144	0.28575448	1.21904762	2.53415649	5.79238095	-2.248402	0.21045709	6.73696559	6.45121111	8.9853676
A_55_P2040	NM_177901	4933402J07R	Mus musculu	chr8:901100	6.54345279	-0.5294369	0.69282511	1.51198306	2.85201794	-2.0414199	0.24292453	5.68650053	6.2159374	7.72792045
A_55_P1986	XR_032569	Gm3267	PREDICTED: I	chr15:37423	6.89950959	-0.2667384	0.83119658	2.10906048	4.31410256	-2.3757989	0.19266964	6.01866384	6.28540222	8.39446269
A_55_P2144	NM_146306	Olfr518	Mus musculu	chr7:116024	6.94947961	-0.0908838	0.93894737	2.01885903	4.05263158	-2.1097428	0.23168831	6.2159374	6.3068212	8.32568023
A_30_P0102	chr13:31736054-31781679		lincRNA:chr1	chr13:31762	6.14483671	-0.7867847	0.57963446	1.23258061	2.34986945	-2.0193653	0.24666667	5.20945337	5.99623808	7.22881869
A_51_P4208	NM_011773	Slc30a3	Mus musculu	chr5:313904	7.75788786	-2.6810496	0.15592784	-1.0901378	0.46971649	-1.5909118	0.33196159	6.33390074	9.01495034	7.9248125
A_30_P0102	chr14:26835914-26845344		lincRNA:chr1	chr14:26842	7.43066775	-0.7808532	0.58202247	1.4347594	2.70337079	-2.2156126	0.2152951	6.43184579	7.21269903	8.64745843
A_52_P2630	NM_009643	Ahnak	Mus musculu	chr19:90935	9.36750231	-3.1895027	0.1096135	-1.3078954	0.40390967	-1.8816072	0.27138122	7.67713234	10.866635	9.55873958
A_55_P1976	NR_015513	E130114P18	Mus musculu	chr4:972349	6.37856791	-2.1232085	0.22953586	-1.6182143	0.3257384	-0.5049942	0.70466321	5.50250034	7.62570884	6.00749454
A_30_P0102	chr4:54851872-54866706_		lincRNA:chr4	chr4:548659	7.5522298	-1.4659745	0.36199095	-2.5874755	0.16637661	1.12150101	2.17573222	7.43740531	8.90337978	6.31590431
A_55_P2005	NM_010695	Lcn4	Mus musculu	chr2:265249	6.40345377	-0.2761244	0.82580645	2.413	5.32580645	-2.6891244	0.15505754	5.4150375	5.6911619	8.1041619
A_55_P1982	ENSMUST00000062214		RIKEN cDNA	chr4:155108	8.09059797	-0.1995118	0.8708452	2.10532405	4.30294397	-2.3048359	0.20238358	7.25581542	7.45532722	9.56065127
A_30_P0103	chr2:10237649-10287591_		lincRNA:chr2	chr2:102702	7.1883626	-0.5008133	0.70670827	1.84824696	3.60062402	-2.3490603	0.19627383	6.23840474	6.73921805	8.58746501
A_55_P2373	AK021026	B430319H21	Mus musculu	chr11:63001	7.75150963	0.10050686	1.07215007	2.59877491	6.05772006	-2.4982681	0.17698904	6.9522559	6.85174904	9.45052395
A_30_P0102	chr15:5969454-5994540_R		lincRNA:chr1	chr15:59943	6.87357793	-1.9657426	0.25600739	0.10290957	1.07393715	-2.0686522	0.2383821	5.52877967	7.49452228	7.59743185
A_55_P1982	ENSMUST00000112781			chr14:43227	8.12602237	-1.1174444	0.46090956	-2.5528851	0.1704139	1.43544067	2.70464768	8.23202112	9.34946554	6.79658045
A_30_P0102	XIST		lincRNA:chrX	chrX:100655	7.89226229	4.48791348	22.4386423	1.20015913	2.29765013	3.28775435	9.76590909	10.4841516	5.99623808	7.19639721
A_52_P1377	NM_019390	Lmna	Mus musculu	chr3:882873	8.76356531	-2.3260899	0.19942389	-0.0479399	0.96731664	-2.27815	0.20616195	7.22881869	9.55490856	9.50696869
A_55_P2061	NM_0010117	Olfr222	Mus musculu	chr11:59384	6.18934692	-0.6719458	0.62765957	1.33110752	2.51595745	-2.0030533	0.24947146	5.29768055	5.96962635	7.30073387
A_55_P2081	XM_0014780	LOC1000472	PREDICTED: I	chr4:831715	7.47471686	-0.8805171	0.54317269	1.17954942	2.26506024	-2.0600666	0.23980496	6.49452228	7.37503943	8.55458885
A_55_P2010	NM_001172	Il11ra1	Mus musculu	chr4:417162	10.2994061	-2.109251	0.23176731	-0.5653997	0.67576816	-1.5438513	0.34296867	9.08170534	11.1909564	10.6255567
A_30_P0102	chr18:5119300-5181600_F		lincRNA:chr1	chr18:51629	9.32468906	-2.0408226	0.24302513	-0.2325554	0.85112597	-1.8082672	0.28553368	8.04165915	10.0824817	9.84992631

A_51_P1605	NM_029335	1700026D08I	Mus musculu	chr7:909243	6.80356854	-0.6958357	0.6173516	1.57128338	2.9716895	-2.2671191	0.20774431	5.81591694	6.51175265	8.08303603
A_55_P1968	NM_145940	Wipi1	Mus musculu	chr11:10944	7.73172625	-2.3128326	0.20126488	-0.9140534	0.53069196	-1.3987792	0.37924991	6.49452228	8.80735492	7.89330153
A_66_P1049	ENSMUST000	LOC1000463	PREDICTED: M	chr12:11715	7.12458015	-0.3283831	0.79642857	2.06916202	4.19642857	-2.3975451	0.18978723	6.2159374	6.54432052	8.61348254
A_55_P2155	NM_025282	Mef2c	Mus musculu	chr13:83772	8.58030286	2.1837818	4.54342984	1.88029937	3.68151448	0.30348243	1.23411978	9.40939094	7.22560913	9.10590851
A_55_P2048	NM_205819	Tlr11	Mus musculu	chr14:509831	6.70946733	0.15318515	1.11202186	2.18300483	4.54098361	-2.0298197	0.24488568	6.08392248	5.93073734	8.11374217
A_55_P2157	NM_019517	Bace2	Mus musculu	chr16:97658	6.26954795	-2.1187651	0.2302439	-1.3219281	0.4	-0.7968371	0.57560976	5.29768055	7.41644569	6.0945176
A_51_P4059	NM_144799	Lmcd1	Mus musculu	chr6:112280	6.41354651	-0.66073	0.63255814	1.41167849	2.66046512	-2.0724085	0.23776224	5.50250034	6.16323035	7.57490884
A_30_P0101	chr15:92174262-92196074	lincRNA:chr1	chr15:92195	8.41914488	-2.6073303	0.16410256	-0.1681228	0.89	-2.4392076	0.1843849	6.73696559	9.34429591	9.17617315
A_51_P1232	NM_029530	6330527O06I	Mus musculu	chr2:1358951	10.6247615	-4.6368866	0.04019371	-0.2948141	0.81517735	-4.3420724	0.0493067	7.63177521	12.2686618	11.9738476
A_30_P0102	chr1:63264155-63345773_	lincRNA:chr1	chr1:633416	11.4408525	-1.5508057	0.34131939	0.62438329	1.54155172	-2.175189	0.22141287	10.1988543	11.74966	12.3740433
A_30_P0102	chr4:115577276-11557756	lincRNA:chr4	chr4:1155775	7.1268978	-0.3785116	0.76923077	1.84208752	3.58528428	-2.2205991	0.21455224	6.26052755	6.63903917	8.48112669
A_55_P2035	NM_001002	Penk	Mus musculu	chr4:406078	11.2657603	-5.4035322	0.02362516	-1.305196	0.40466613	-4.0983362	0.05838185	8.09847079	13.502003	12.196807
A_30_P0102	chr3:83089315-83108396_	lincRNA:chr3	chr3:831049	7.34043749	-2.1646884	0.2230303	-1.8787501	0.27191919	-0.2859382	0.82020802	6.52356196	8.68825031	6.80950019
A_66_P1308	NM_130448	Pcdh18	Mus musculu	chr3:495473	9.28541413	-2.7669891	0.14691065	-1.7044222	0.30684411	-1.0625669	0.47877943	8.00889544	10.7758846	9.07146236
A_52_P2334	NM_008090	Gata2	Mus musculu	chr6:881563	8.84874699	-5.7164474	0.01901857	-2.0704152	0.23809097	-3.6460322	0.07987943	5.72792045	11.4443679	9.37395266
A_30_P0102	chr9:121845920-12185812	lincRNA:chr9	chr9:121855	7.95847467	2.43422035	5.40472175	1.56042628	2.94940978	0.87379407	1.8324757	9.06114614	6.62692579	8.18735207
A_55_P2362	AK078562	D4Ertd681e	Mus musculu	chr4:975607	7.38366092	-1.121146	0.45972851	-2.3024757	0.20271493	1.18132976	2.26785714	7.40372219	8.52486815	6.22239242
A_30_P0102	chr6:52046805-52072744_	lincRNA:chr6	chr6:520530	7.41710689	-2.6480453	0.15953608	-3.1112693	0.11572165	0.46322396	1.37861915	6.6888331	9.33687844	6.22560913
A_52_P7243	NM_008439	Khk	Mus musculu	chr5:312335	9.78674129	-2.7171808	0.15207124	-0.100824	0.93250025	-2.6163568	0.16307903	8.00889544	10.7260762	10.6252522
A_51_P4496	NR_033616	Pldi	Mus musculu	chr10:60391	7.21171462	-0.4983206	0.70793037	2.84629058	7.19148936	-3.3446112	0.09844002	5.93073734	6.42905797	9.27534855
A_30_P0102	chr5:22890322-22939119_	lincRNA:chr5	chr5:228905	8.72790327	2.35574828	5.11859649	0.15261042	1.11157895	2.20313786	4.60479798	10.247532	7.8917837	8.04439412
A_55_P2063	NM_028207	Dusp3	Mus musculu	chr11:10183	7.58905961	-2.1131457	0.23114248	-0.804494	0.57256286	-1.3086517	0.403698	6.4484605	8.56160618	7.75711217
A_51_P2560	NM_011878	Tiam2	Mus musculu	chr17:35188	8.0259924	1.64115082	3.11914544	-0.5556265	0.68036154	2.19677732	4.58454106	9.30530178	7.66415095	7.10852446
A_30_P0102	chr16:30195156-30200385	lincRNA:chr1	chr16:30200	6.81525265	-0.4487581	0.73267327	1.70898483	3.26930693	-2.1577429	0.2241066	5.94641896	6.39517708	8.1041619
A_55_P2143	NM_031380	Fstl3	Mus musculu	chr10:79244	8.09810558	-0.201442	0.86968085	1.8319922	3.56028369	-2.0334342	0.24427291	7.35314683	7.55458885	9.38658105
A_30_P0102	chr4:145539725-14559600	lincRNA:chr4	chr4:145586	9.19167587	-2.3623906	0.19446864	-1.8024151	0.28669425	-0.5599754	0.67831371	8.21755386	10.5799444	8.77752931
A_51_P1001	NM_008613	Mns1	Mus musculu	chr9:723062	7.78545089	-1.7176531	0.30404293	-2.2687678	0.20750704	0.55111473	1.46521739	7.39660478	9.11425784	6.84549005
A_51_P1582	NM_008564	Mcm2	Mus musculu	chr6:888336	9.45694397	-0.7023048	0.61458957	-2.6702296	0.15710167	1.96792476	3.91204986	9.87881728	10.5811221	7.91089253
A_55_P2089	NM_001177	Ak4	Mus musculu	chr4:101139	9.37853957	-4.3995999	0.04737928	-2.1583018	0.22401981	-2.2412981	0.21149595	7.16490693	11.5645068	9.40620501
A_55_P2009	NM_172563	Hlf	Mus musculu	chr11:90200	7.82529331	-2.0512409	0.24127647	-1.851702	0.27706531	-0.1995389	0.87082885	7.07503339	9.12627427	7.27457229
A_55_P2053	NM_175171	Mast4	Mus musculu	chr13:10352	7.79502865	-0.4457752	0.73418972	1.63676789	3.10968379	-2.0825431	0.23609787	6.9522559	7.39803107	9.03479896
A_66_P1042	ENSMUST00000068158		RIKEN cDNA	chr4:427494	9.51361125	-3.1291633	0.1142952	-0.000479	0.99966807	-3.1286843	0.11433315	7.42766204	10.5568253	10.5563464
A_30_P0102	chr16:9129538-9137898_R	lincRNA:chr1	chr16:91376	6.58331008	-0.646363	0.63888889	1.54008663	2.90811966	-2.1864497	0.2196914	5.63903917	6.28540222	7.82548885
A_55_P1956	NM_011782	Adamts5	Mus musculu	chr16:85858	7.18368895	2.49072891	5.62061856	0.04970262	1.03505155	2.44102629	5.43027888	8.82760735	6.33687844	6.38658105
A_55_P2120	XM_001480	Gm4522	PREDICTED: M	chr6:126244	13.0288848	2.8191146	7.05729149	2.49022253	5.61864608	0.32889207	1.25604841	14.0782203	11.2591057	13.7493282
A_51_P4913	NM_009932	Col4a2	Mus musculu	chr8:114488	10.2825674	-3.7729522	0.07315234	-0.7430531	0.5974736	-3.0298991	0.1224361	8.01495034	11.7879026	11.0448494
A_30_P0102	chr7:134779841-13484059	lincRNA:chr7	chr7:134803	7.37424278	-0.3443345	0.78767123	1.68669239	3.21917808	-2.0310269	0.24468085	6.58245565	6.92679015	8.61348254
A_55_P2177	NM_008066	Gabra2	Mus musculu	chr5:713523	8.49488152	2.98988124	7.94408602	0.6663901	1.58709677	2.32349114	5.00542005	10.2660057	7.27612441	7.94251451
A_55_P2043	NM_010441	Hmga2	Mus musculu	chr10:11980	8.09348564	-1.5648307	0.33801737	-2.203109	0.21716914	0.63827832	1.55647059	7.78463485	9.34946554	7.14635653
A_55_P2182	ENSMUST00000099245			chrUn_rando	6.39739288	-0.8917837	0.53894737	1.16349873	2.24	-2.0552824	0.2406015	5.4150375	6.3068212	7.47031993
A_55_P2071	NM_028440	3110003A17I	Mus musculu	chr10:17731	11.1035256	1.41249868	2.66197806	-0.7406715	0.59846072	2.15317021	4.44804139	12.2920819	10.8795832	10.1389117
A_51_P1719	NM_009696	Apoe	Mus musculu	chr7:202817	10.3542042	-2.8751515	0.13629915	-2.0296943	0.24490697	-0.8454572	0.5565344	9.11400131	11.9891528	9.95945854
A_55_P2031	NM_008718	Npas1	Mus musculu	chr7:170411	9.24068088	1.23125992	2.3477193	2.81543161	7.03929825	-1.5841717	0.3335161	9.12304363	7.8917837	10.7072153
A_30_P0103	chr17:36131460-36136694	lincRNA:chr1	chr17:361361	6.52468617	1.72935241	3.31578947	-0.5224838	0.69617225	2.2518362	4.7628866	7.85174904	6.12239663	5.59991284

A_55_P1981:	NM_010683	Lamc1	Mus musculu	chr1:1550662	8.45534561	-2.653767	0.15890462	-1.3958155	0.38002981	-1.2579515	0.41813725	7.15143943	9.80520646	8.40939094
A_51_P5016!	NM_212445	Kdelc2	Mus musculu	chr9:532094!	7.10040395	-2.2051144	0.21686747	-0.8296888	0.5626506	-1.3754256	0.38543897	5.9068906	8.11200503	7.28231624
A_55_P2048	NM_0011360	Cd82	Mus musculu	chr2:9327407	9.04713729	-3.0250581	0.12284763	-1.4162054	0.37469655	-1.6088527	0.32785897	7.50250034	10.5275585	9.11135306
A_52_P6523:	ENSMUST000	Lhx1	LIM homeob	chr11:843319	10.1229735	-4.3482259	0.04909684	-0.9912979	0.50302503	-3.356928	0.09760318	7.55458885	11.9028147	10.9115168
A_55_P1967:	XR_004698	LOC674877	PREDICTED: Mus musculus		10.8762	-2.1522305	0.22496454	-0.5693921	0.67390071	-1.5828384	0.33382446	9.63117706	11.7834075	11.2140155
A_55_P1971	XM_0014804	Gm4538	PREDICTED: M	chr17:662656	6.74010058	0.03588979	1.02518892	2.04031388	4.11335013	-2.0044241	0.24923454	6.08392248	6.0480327	8.08834657
A_30_P0103:	chr4:54851872-54866706_		lincRNA:chr4:	chr4:5486565	7.73670295	-1.264613	0.416211	-2.5770953	0.167578	1.31248234	2.48368522	7.7526594	9.0172724	6.44017706
A_51_P1098	NM_011707	Vtn	Mus musculu	chr11:78315:	8.40936358	-5.2125938	0.02696826	-3.0517899	0.12059234	-2.1608039	0.22363161	5.95156434	11.1641581	8.11236828
A_30_P0102!	chr18:75131739-75144870		lincRNA:chr1	chr18:75140!	7.90649649	-0.2911169	0.81726908	1.88548812	3.69477912	-2.1766051	0.22119565	7.08392248	7.37503943	9.26052755
A_55_P2078	NM_144855	Cbs	Mus musculu	chr17:317496	7.99188963	-3.0591236	0.11998088	-3.0572089	0.12014022	-0.0019147	0.99867374	6.97154355	10.0306671	6.97345821
A_55_P1967	XR_034924	Gm5207	PREDICTED: M	chr14:958205	8.31788338	-2.6246208	0.16214755	-1.9820476	0.25313035	-0.6425732	0.6405694	7.22881869	9.85343952	7.87139191
A_30_P0102(	chrX:123306246-12330797		lincRNA:chrX:	chrX:1233077	6.47695846	-0.3131579	0.80487805	2.42626475	5.375	-2.7394226	0.14974475	5.45943162	5.7725895	8.19885426
A_30_P0101	chr4:3406175-3443875_F		lincRNA:chr4:	chr4:3435225	7.71079146	-0.3214889	0.80024361	2.1650355	4.48477467	-2.4865244	0.17843563	6.77478706	7.09627591	9.26131141
A_30_P0102!	chr15:61984389-62102500		lincRNA:chr1!	chr15:62074(	8.44784266	-3.1193267	0.11507715	-0.9616247	0.51347834	-2.157702	0.22411296	6.6888331	9.80815977	8.8465351
A_55_P2014:	NM_0011776	Gm7120	Mus musculu	chr13:12027	7.61445056	2.28689708	4.88005391	-0.294484	0.81536388	2.58138113	5.98512397	9.23720996	6.95031288	6.65582883
A_51_P4455:	NM_020492	Glra1	Mus musculu	chr11:553797	6.80135315	-2.4436858	0.18381344	-0.9266922	0.5260631	-1.5169936	0.3494133	5.48112669	7.9248125	6.99812027
A_52_P6498:	NM_031252	Il23a	Mus musculu	chr10:12773:	6.75285969	0.2067108	1.15405405	2.2126114	4.63513514	-2.0059006	0.24897959	6.15312976	5.94641896	8.15903036
A_30_P0102:	chr17:9889983-9919633_R		lincRNA:chr1	chr17:98947!	6.28046279	0.07934343	1.0565371	2.0829577	4.23674912	-2.0036143	0.24937448	5.63903917	5.55969574	7.64265344
A_30_P0102!	chr12:33808493-33829143		lincRNA:chr1:	chr12:33819{	8.19623013	-2.1489985	0.22546909	-0.5074272	0.70347585	-1.6415713	0.32050721	6.93270689	9.08170534	8.57427815
A_55_P2030:	NM_178405	Atp1a2	Mus musculu	chr1:174201!	7.75074771	-4.8383783	0.03495449	-3.5396825	0.08599028	-1.2986958	0.40649351	5.70505635	10.5434347	7.00375213
A_55_P2199	AK020245	9030205G03	Mus musculu	chr8:1450758	8.07852826	2.4193939	5.34946237	1.53153718	2.89093702	0.88785672	1.85042508	9.18094514	6.76155123	8.29308841
A_51_P1535!	NM_175199	Hspa12a	Mus musculu	chr19:58870!	9.60011385	-2.0090102	0.24844352	0.4047894	1.32389564	-2.4137996	0.18766096	8.12584393	10.1348541	10.5396435
A_55_P2132:	NM_024452	Luzp1	Mus musculu	chr4:1360991	9.56394224	-3.0222699	0.12308528	-0.035622	0.97561108	-2.9866479	0.12616224	7.56096965	10.5832395	10.5476176
A_51_P5058	NM_175386	Lhfp	Mus musculu	chr3:5306474	9.63195227	-2.3444006	0.19690879	-0.960214	0.51398066	-1.3841865	0.38310546	8.38908991	10.7334905	9.77327644
A_55_P1978!	ENSMUST000	Rpl26-ps2	PREDICTED: M	chr12:587337	8.60656765	2.97671976	7.87194293	-0.9728152	0.50951087	3.94953493	15.45	10.9153192	7.93859946	6.96578428
A_55_P2315	AK045275		Mus musculu	chr2:7311334	10.7037069	2.45725133	5.49169435	1.95301046	3.87181617	0.50424088	1.41837683	11.690871	9.23361968	11.1866301
A_51_P4941:	NM_007431	Alpl	Mus musculu	chr4:1372977	8.0006114	-3.8189615	0.07085623	-3.7025882	0.07680861	-0.1163734	0.92250373	6.6888331	10.5077946	6.80520646
A_30_P0101	chr1:34735032-34781146_		lincRNA:chr1:	chr1:3478108	7.64125532	-3.4840336	0.08937198	-1.8835581	0.27101449	-1.6004755	0.32976827	5.94641896	9.43045255	7.54689446
A_55_P2110!	XM_0014747	Gm4507	PREDICTED: M	chr14:11861(	6.7683262	-0.3141086	0.80434783	1.83750453	3.57391304	-2.1516131	0.22506083	5.94641896	6.26052755	8.09803208
A_51_P2036	NM_021407	Trem3	Mus musculu	chr17:48397!	6.07321059	0.24997825	1.18918919	2.34129342	5.06756757	-2.0913152	0.23466667	5.45943162	5.20945337	7.55074679
A_55_P2047:	NM_0011595	Meis2	Mus musculu	chr2:1156897	11.1867377	-3.6801227	0.07801402	-1.301032	0.40583578	-2.3790907	0.19223052	9.16699991	12.8471226	11.5460906
A_55_P2026!	ENSMUST00000101381		RIKEN cDNA (	chr11:32625!	10.5139034	-2.9727015	0.12738775	0.29659426	1.22824151	-3.2692958	0.10371556	8.43323768	11.4059392	11.7025335
A_55_P2180!	NM_182929	Rims3	Mus musculu	chr4:1205639	9.30908538	-1.9253281	0.26328038	0.79527175	1.73540423	-2.7205998	0.15171127	7.76044275	9.68577082	10.4810426
A_30_P0103:	chr10:60473030-60535340		lincRNA:chr1(	chr10:605288	6.7275421	-0.1375035	0.90909091	2.06836236	4.19410319	-2.2058659	0.21675454	5.94641896	6.08392248	8.15228484
A_30_P0102(	chr7:82902248-82955248_		lincRNA:chr7:	chr7:8292447	7.50347514	-2.4376935	0.18457851	-2.9202088	0.13210814	0.48251523	1.39717742	6.85174904	9.28944258	6.36923381
A_55_P2003(	NM_177161	P4ha3	Mus musculu	chr7:1074674	8.74494451	-2.7276157	0.15097528	-1.663778	0.31561158	-1.0638377	0.47835788	7.48112669	10.2087424	8.54496443
A_55_P2026	NM_010127	Pou6f1	Mus musculu	chr15:10040!	8.98120458	4.71161774	26.2022308	-0.0426024	0.97090204	4.7542201	26.9875125	12.1364839	7.42486612	7.38226376
A_51_P2674!	NM_026514	Cdc42ep3	Mus musculu	chr17:797334	10.0717452	2.16734502	4.4919598	1.17223233	2.25360134	0.99511269	1.99323621	11.1258977	8.95855272	10.130785
A_55_P2004:	NM_133237	Apcdd1	Mus musculu	chr18:631127	8.81732727	-3.3743018	0.09643483	-2.3955885	0.19004481	-0.9787133	0.50743209	7.36632221	10.740624	8.34503556
A_55_P2142:	NM_054045	Hist2h3c2-ps	Mus musculu	chr3:960420!	8.31973479	1.96287975	3.89839357	-0.094578	0.93654618	2.0574577	4.16252144	9.65984728	7.69696753	7.60238957
A_51_P2062:	NM_028051	Slc39a5	Mus musculu	chr10:12783	7.36924391	-0.509861	0.70229008	1.51732378	2.86259542	-2.0271848	0.24533333	6.52356196	7.033423	8.55074679
A_30_P0102	chr1:88398401-88398644_		lincRNA:chr1:	chr1:8839854	8.97072944	-2.0522453	0.24110855	-1.2760203	0.41293303	-0.776225	0.58389262	8.027906	10.0801513	8.80413102
A_51_P2063	NM_178606	Reep3	Mus musculu	chr10:66474:	11.3015652	0.30282591	1.23355831	-2.5551551	0.17014597	2.857981	7.25	12.3551675	12.0523416	9.49718654
A_55_P2088	NM_015749	Tcn2	Mus musculu	chr11:38172!	8.91169116	-3.0786064	0.1183715	-1.5211059	0.34841872	-1.5575004	0.33973919	7.36632221	10.4449286	8.92382266

A_52_P38148	NM_133903	Spon2	Mus musculu	chr5:3355667	6.67584813	-0.7536765	0.59309021	1.46068975	2.75239923	-2.2143663	0.21548117	5.68650053	6.44017706	7.90086681
A_55_P22697	NM_026714	Ccdc163	Mus musculu	chr4:1163816	8.33855519	-2.4778728	0.17950889	-1.3689539	0.38717189	-1.1089189	0.46364133	7.14295795	9.62083075	8.25187686
A_30_P01020	chr3:60288326-60288925_		lincRNA:chr3:	chr3:6028868	6.22746613	-0.2730185	0.82758621	1.75806654	3.38244514	-2.031085	0.24467099	5.45943162	5.73245011	7.49051665
A_52_P14431	NM_008077	Gad1	Mus musculu	chr2:7043969	13.0545046	1.68073707	3.20591699	2.29264018	4.89951918	-0.6119031	0.65433298	13.4107826	11.7300455	14.0226857
A_30_P01022	chr6:127111250-12714292		lincRNA:chr6:	chr6:1271335	13.0821256	-2.5163571	0.17478375	-0.6387399	0.64227371	-1.8776172	0.2721328	11.6174675	14.1338245	13.4950847
A_52_P46483	NM_010053	Dlx1	Mus musculu	chr2:7137149	13.6739558	2.20326687	4.60520975	1.98303422	3.95323638	0.22023264	1.16492142	14.4817889	12.2785221	14.2615563
A_52_P12473	NM_028593	Cybrd1	Mus musculu	chr2:7098070	6.46547816	-2.1219905	0.22972973	-1.6174361	0.32591415	-0.5045544	0.70487805	5.58996318	7.71195371	6.0945176
A_52_P16302	NM_182959	Slc17a8	Mus musculu	chr10:890376	8.15818521	-5.4292662	0.02320748	-2.8273569	0.1408902	-2.6019093	0.16472034	5.48112669	10.9103929	8.08303603
A_51_P22372	NM_133235	Khdrbs2	Mus musculu	chr1:3271440	9.6158347	2.15146198	4.44277778	2.00958605	4.02666667	0.14187593	1.10333885	10.3802807	8.22881869	10.2384047
A_55_P19948	NM_0011635	2010001E11F	Mus musculu	chr10:396402	7.44608383	0.51061516	1.42465753	2.29237837	4.89863014	-1.7817632	0.29082774	7.02236781	6.51175265	8.80413102
A_66_P10973	NM_0011659	1700123I01R	Mus musculu	chr19:622385	7.61563199	-1.3332434	0.396875	0.9692426	1.9578125	-2.302486	0.20271349	6.40372219	7.73696559	8.7062082
A_55_P19536	NM_146974	Olfr1262	Mus musculu	chr2:8984342	6.88426766	-0.3899465	0.76315789	2.29896695	4.92105263	-2.6889135	0.15508021	5.857981	6.24792751	8.54689446
A_52_P57024	NM_029116	Kbtbd11	Mus musculu	chr8:1503319	10.5833065	-2.1009537	0.2331041	-1.3030125	0.40527904	-0.7979412	0.57516938	9.61700823	11.717962	10.4149494
A_55_P20028	NM_0011014	Zcchc24	Mus musculu	chr14:265311	8.30734781	-3.5004234	0.08836241	-3.4043767	0.09444533	-0.0960467	0.93559322	7.10852446	10.6089478	7.20457114
A_55_P23040	BG083149	AU015230	H3085A07-5 I	chr17:628711	7.25129251	0.3820119	1.30315789	2.45140201	5.46947368	-2.0693901	0.2382602	6.6888331	6.3068212	8.75822321
A_55_P21247	NM_028266	Col16a1	Mus musculu	chr4:1297764	6.32852011	-2.073669	0.23755459	-1.6692788	0.31441048	-0.4043903	0.75555556	5.50250034	7.57616938	5.9068906
A_55_P21355	XM_0014752	Gm14703	PREDICTED: M	chrX:7229093	8.73834345	-0.9863251	0.5047619	1.05861886	2.08293651	-2.0449439	0.24233187	7.72792045	8.71424552	9.77286438
A_55_P21088	NM_009448	Tuba1c	Mus musculu	chr15:988684	12.2369286	-2.0988567	0.23344318	-1.9991499	0.25014735	-0.0997068	0.93322266	11.5040741	13.6029308	11.6037809
A_66_P12707	NM_008109	Gdf5	Mus musculu	chr2:1557668	9.22310763	-0.3673543	0.77520278	1.777027	3.42719197	-2.1443814	0.22619182	8.3858624	8.75321675	10.5302438
A_55_P20176	NM_0011103	Gprc5c	Mus musculu	chr11:114733	7.6719257	-3.4619041	0.09075342	-1.0917925	0.46917808	-2.3701116	0.19343066	5.72792045	9.18982456	8.09803208
A_55_P20488	NM_009501	Vax1	Mus musculu	chr19:592408	7.43403417	-2.0669787	0.23865878	-0.8335571	0.56114398	-1.2334216	0.42530756	6.33390074	8.40087944	7.56732234
A_51_P32493	XR_031705	LOC1000456	PREDICTED: M	chr1:2079317	9.90499652	-1.9577422	0.25743102	-3.761593	0.07373058	1.80385087	3.49150943	9.85369942	11.8114416	8.04984855
A_55_P20715	ENSMUST00000090371		methyltransfe	chr3:1230679	6.70081549	-2.2834809	0.20540156	-1.2344049	0.42501777	-1.049076	0.48327759	5.58996318	7.87344411	6.63903917
A_51_P51613	NM_015786	Hist1h1c	Mus musculu	chr13:238320	10.8604543	0.35846297	1.28205928	2.03946145	4.11092044	-1.6809985	0.31186672	10.4196091	10.0611461	12.1006076
A_51_P10225	ENSMUST00000050681		tensin 1 Gene	chr1:7403644	6.6515504	-1.1623822	0.44677419	1.04354772	2.06129032	-2.20593	0.21674491	5.52877967	6.6911619	7.73470962
A_55_P21780	NM_010567	Inppl1	Mus musculu	chr7:1089712	10.6711359	-2.896724	0.13427624	-2.504767	0.17619355	-0.3919571	0.7620951	9.57490884	12.4716329	9.9668659
A_52_P12013	ENSMUST00000066875		RIKEN cDNA 6	chr8:1090329	6.87232936	0.44801482	1.36416185	2.61997609	6.14739884	-2.1719613	0.22190879	6.29768055	5.84966573	8.46964182
A_30_P01026	chr14:27176059-27202735		lincRNA:chr14	chr14:271955	7.06383663	-1.3076674	0.40397351	0.78396307	1.7218543	-2.0916305	0.23461538	5.93073734	7.23840474	8.02236781
A_55_P19752	NR_002845	Copg2as2	Mus musculus coatomer pr		12.8142116	1.00939499	2.01306672	2.28106739	4.86037418	-1.2716724	0.41417937	12.7267858	11.7173908	13.9984582
A_51_P14810	NM_011234	Rad51	Mus musculu	chr2:1189612	7.47135546	-0.64822	0.63806706	-2.1403519	0.22682446	1.49213185	2.81304348	7.7526594	8.40087944	6.26052755
A_66_P11166	NM_013602	Mt1	Mus musculu	chr8:9670416	10.7474157	-3.3472999	0.09825674	-2.4918939	0.17777275	-0.855406	0.55270978	9.34651373	12.6938136	10.2019197
A_30_P01020	chr15:96984422-97000322		lincRNA:chr15	chr15:969906	8.02159197	-1.1207665	0.45984944	1.02512219	2.03513174	-2.1458887	0.22595561	6.93270689	8.05347341	9.0785956
A_66_P11886	NM_134094	Ncald	Mus musculu	chr15:372960	7.80181479	-2.2035927	0.21709634	-1.2126379	0.43147897	-0.9909549	0.50314465	6.73696559	8.94055831	7.72792045
A_51_P36394	NM_007669	Cdkn1a	Mus musculu	chr17:292373	10.5728831	-4.8212576	0.03537177	-0.8241462	0.56481637	-3.9971114	0.06262526	7.63342681	12.4546844	11.6305382
A_66_P11860	NM_008480	Lama1	Mus musculu	chr17:681719	9.53005954	-2.7457979	0.14908449	-1.0889051	0.47011804	-1.6568929	0.31712139	8.06249593	10.8082939	9.71938882
A_51_P12526	NM_177470	Acaa2	Mus musculu	chr18:749656	9.9050526	-2.2706599	0.20723507	-0.9440491	0.51977203	-1.3266108	0.39870378	8.70596235	10.9766223	10.0325732
A_55_P21541	NR_033642	Gm3230	Mus musculu	chr2:1957749	6.81166582	-0.1433133	0.90543735	2.15965667	4.46808511	-2.3029699	0.2026455	5.99623808	6.13955135	8.29920802
A_30_P01029	chr3:9401052-9415202_F		lincRNA:chr3:	chr3:9409096	8.02167051	-1.0167585	0.49422554	1.26597169	2.4048913	-2.2827302	0.20550847	6.92184094	7.93859946	9.20457114
A_55_P20549	ENSMUST00000053171		leucine rich re	chr9:6041710	7.9189341	2.66193855	6.32882883	2.46650367	5.52702703	0.19543488	1.14506927	8.87139191	6.20945337	8.67595703
A_55_P19837	NM_009689	Birc5	Mus musculu	chr11:117711	9.23886879	-2.0307476	0.24472822	-3.1148713	0.11543308	1.0841237	2.12008734	8.92332749	10.9540751	7.83920379
A_55_P19798	NM_007709	Cited1	Mus musculu	chrX:9944283	10.3118598	-2.8585741	0.13787434	-1.5841156	0.33352907	-1.2744585	0.41338028	8.93418229	11.7927564	10.2086408
A_55_P19645	NM_0010019	Megf10	Mus musculu	chr18:574570	10.5076206	1.20978985	2.31303942	-2.8841863	0.13544825	4.09397615	17.0769231	12.2755426	11.0657527	8.18156641
A_55_P19902	NM_0010084	Gm1568	Mus musculu	chr12:818568	11.6082568	2.20260317	4.60309166	0.8813914	1.8421511	1.32121177	2.49875901	12.7828617	10.5802586	11.46165
A_52_P39899	NM_010279	Gfra1	Mus musculu	chr19:583116	9.82500611	-2.1239152	0.22942346	-0.535207	0.69005964	-1.5887081	0.33246903	8.58746501	10.7113802	10.1761731

A_55_P2137(	ENSMUST00000105170			chr1:8510994	6.51378978	-0.5932586	0.66284404	1.5849625	3	-2.1782211	0.22094801	5.58996318	6.18322182	7.76818432
A_30_P0103:	chr5:22810359-22836386_		lincRNA:chr5:	chr5:2283631	6.53880551	0.26378797	1.20062696	2.15527823	4.45454545	-1.8914903	0.2695285	5.99623808	5.73245011	7.88772834
A_55_P20348	NM_0010241	Gm5868	Mus musculu	chr5:7297306	10.6415441	1.05611556	2.07932542	-1.065756	0.47772226	2.12187156	4.35258226	11.7008732	10.6447576	9.57900159
A_55_P2295	AA711038	AI594671	vt54g10.r1 Ba	chr11:398498	6.62751009	3.49630102	11.284738	0.80688537	1.74943052	2.68941565	6.45052083	8.68941565	5.19311463	6
A_55_P2128	NM_146131	Pbxip1	Mus musculu	chr3:8925280	7.96092771	-2.9019424	0.13379143	-2.2782763	0.2061439	-0.623666	0.64901961	6.78572491	9.68766728	7.40939094
A_55_P2294(	CB196702	AA536887	AGENCOURT_	chr7:1444785	6.9845401	-2.5775967	0.16751978	-1.9222448	0.26384365	-0.6553518	0.63492063	5.9068906	8.48448727	6.56224242
A_51_P1823(	NM_007743	Col1a2	Mus musculu	chr6:4490619	9.4634585	-3.0769181	0.1185101	-0.4995222	0.70734101	-2.5773959	0.16754309	7.57868718	10.6556053	10.1560831
A_51_P4235	NM_011408	Slfn2	Mus musculu	chr11:828837	6.65296716	-0.4383244	0.73799127	1.63450196	3.10480349	-2.0728263	0.23769339	5.81591694	6.25424129	7.88874325
A_55_P2152	NM_054071	Fgfrl1	Mus musculu	chr5:1091358	8.93537692	-2.9061271	0.13340392	-2.9379632	0.13049232	0.03183609	1.02231237	7.97727992	10.883407	7.94544384
A_30_P0101	chr9:20313342-20323517_		lincRNA:chr9:	chr9:2032341	10.0692525	0.04759164	1.03353815	2.12062477	4.34882232	-2.0730331	0.23765932	9.39410537	9.34651373	11.4671385
A_52_P9575	NM_172788	Sh3rf3	Mus musculu	chr10:586015	8.09526882	3.09652317	8.55354919	-0.0035977	0.99750934	3.10012092	8.57490637	10.1608168	7.06429368	7.06069593
A_55_P2036	NM_011611	Cd40	Mus musculu	chr2:1648970	6.53453188	-2.0462937	0.24210526	-1.4061092	0.37732794	-0.6401845	0.6416309	5.63903917	7.68533283	6.27922364
A_55_P2012	NM_0011360	Ldha	Mus musculu	chr7:5411093	13.5633643	-2.9962322	0.12532688	-0.9252026	0.52660658	-2.0710297	0.23798958	11.874277	14.8705092	13.9453067
A_55_P2123	NM_178777	Nhlh2	Mus musculu	chr3:1018183	9.26162369	-2.5397586	0.1719715	-2.0050806	0.24912114	-0.534678	0.69031274	8.23681148	10.7765701	8.77148947
A_52_P5025	NM_010101	S1pr3	Mus musculu	chr13:515170	8.61778013	-2.9524801	0.12918584	-1.3033873	0.40517378	-1.6490928	0.31884058	7.08392248	10.0364026	8.73301532
A_51_P3939	NM_007701	Vsx2	Mus musculu	chr12:859362	8.44155509	-4.6000995	0.04123178	-2.8232467	0.14129216	-1.7768528	0.29181929	6.31590431	10.9160038	8.09275714
A_55_P2078	NM_178250	Pramel7	Mus musculu	chr2:8732932	7.19988679	-0.4651227	0.72440945	1.88783226	3.7007874	-2.352955	0.19574468	6.26052755	6.72565028	8.61348254
A_51_P1122	NM_010357	Gsta4	Mus musculu	chr9:7804617	8.29363526	-2.1466184	0.22584136	-0.6876678	0.6208567	-1.4589506	0.36375762	7.09177892	9.23839732	8.55072954
A_55_P1984	XR_001935	Gm8341	PREDICTED: N	chr15:193844	8.86845261	-2.8831962	0.13554125	-1.735639	0.30027599	-1.1475572	0.45138889	7.52486815	10.4080643	8.67242534
A_55_P1964	ENSMUST00000066625			chr2:7925089	6.73438146	-0.095325	0.93606138	2.22028245	4.65984655	-2.3156074	0.20087816	5.93073734	6.0260623	8.24634474
A_55_P2073	NM_0010811	Mki67	Mus musculu	chr7:1428848	9.58533798	-1.8847959	0.27078206	-3.1136374	0.11553186	1.22884145	2.34378698	9.36668649	11.2514824	8.13784503
A_55_P2083	NM_175549	Robo2	Mus musculu	chr16:738947	9.62602851	2.11910416	4.34424105	-0.2613994	0.83427831	2.38050352	5.20718447	11.1258977	9.00679358	8.74539422
A_51_P3067	NR_015543	2810055G20I	Mus musculu	chr16:775017	9.11590897	-1.3093193	0.40351122	-2.0155619	0.24731782	0.70624258	1.6315493	8.91488339	10.2242027	8.20864081
A_30_P0102	chr6:118250184-11825873		lincRNA:chr6:	chr6:1182502	7.05573707	0.18132976	1.13392857	2.31870419	4.98883929	-2.1373744	0.22729306	6.40372219	6.22239242	8.54109662
A_55_P1965	NM_025565	Spc25	Mus musculu	chr2:6903201	8.940606	-1.285879	0.41012085	-3.0043652	0.12462236	1.71848617	3.29090909	9.08480839	10.3706874	7.36632221
A_55_P1957	NM_0011462	Pdgfrb	Mus musculu	chr18:612446	7.24832866	-2.4908185	0.17790531	-2.3443525	0.19691535	-0.146466	0.90346084	6.36923381	8.86005235	6.51569984
A_51_P3183	NM_008827	Pgf	Mus musculu	chr12:865103	9.39076254	-2.0686001	0.2383907	-0.7110674	0.610868	-1.3575327	0.39024912	8.24871825	10.3173184	9.60625097
A_30_P0102	chr11:60728207-60745369		lincRNA:chr1	chr11:607318	7.06040164	0.06241261	1.04421053	2.19832872	4.58947368	-2.1359161	0.22752294	6.36923381	6.3068212	8.50514992
A_30_P0102	chr8:94849929-94870274_		lincRNA:chr8:	chr8:9485030	6.84417576	-2.8486229	0.13882863	-1.4098311	0.37635575	-1.4387919	0.36887608	5.4150375	8.26366044	6.85382935
A_55_P1992	XM_0014766	LOC1000466	PREDICTED: N	chrX:7511132	7.68996033	0.22014698	1.16485226	2.61859673	6.14152411	-2.3984497	0.18966827	6.96385941	6.74371243	9.36230915
A_30_P0101	chr16:31607863-31608726		lincRNA:chr1	chr16:316086	6.42774677	-0.1631652	0.89306358	1.89740832	3.72543353	-2.0605735	0.23972071	5.68650053	5.84966573	7.74707405
A_51_P3890(	NM_011891	Sgcd	Mus musculu	chr11:467944	6.99990975	-0.6552956	0.6349454	1.43737069	2.70826833	-2.0926662	0.234447	6.08392248	6.73921805	8.17658873
A_55_P1987	NM_146328	Olfr110	Mus musculu	chr17:376364	6.98092037	-0.2538671	0.83864542	2.03688507	4.10358566	-2.2907522	0.20436893	6.13271392	6.38658105	8.42346612
A_55_P2062	NM_0010130	Ttf2	Mus musculu	chr3:1007428	8.27784129	-0.7115094	0.61068091	-2.3123293	0.20133511	1.60081994	3.0331565	8.57427815	9.2857875	6.97345821
A_55_P1987	NM_025896	Prl3a1	Mus musculu	chr13:273684	8.5289077	0.64835121	1.56737589	2.27460533	4.83865248	-1.6262541	0.32392818	8.20294006	7.55458885	9.82919418
A_55_P2039	NM_178679	Zfp365	Mus musculu	chr10:673489	8.65633184	-2.0524884	0.24106792	-1.1673783	0.44522968	-0.8851101	0.54144621	7.67713234	9.72962074	8.56224242
A_51_P3005(	NM_183405	Cox6b2	Mus musculu	chr7:4703547	10.3410283	-4.2714831	0.05177922	-1.7743064	0.29233483	-2.4971767	0.17712297	8.08480839	12.3562915	10.5819851
A_30_P0102	chr6:125330524-12541024		lincRNA:chr6:	chr6:1253956	6.92637427	-0.1456586	0.9039666	1.96802339	3.91231733	-2.113682	0.23105656	6.17326071	6.31891935	8.28694274
A_30_P0102(	chr10:3231337-3231802_F		lincRNA:chr1	chr10:323167	6.57288993	-2.0227389	0.24609053	-1.2439256	0.42222222	-0.7788133	0.582846	5.63903917	7.6617781	6.41785251
A_55_P2074	NM_0011102	Ppm1h	Mus musculu	chr10:122379	9.82678913	-1.9773332	0.25395887	0.07223919	1.0513472	-2.0495724	0.24155567	8.48448727	10.4618205	10.5340597
A_30_P0102	chr3:88009045-88036270_		lincRNA:chr3:	chr3:8803331	10.9657668	2.23347767	4.70266209	1.86222544	3.63568055	0.37125223	1.29347505	11.8340101	9.60053242	11.4627579
A_30_P0102	chr4:56480568-56658018_		lincRNA:chr4:	chr4:5661764	8.04155416	-2.2260503	0.21374309	-0.3939374	0.76104972	-1.8321129	0.28085299	6.6888331	8.91488339	8.52094601
A_30_P0102	chr19:5834117-5835940_R		lincRNA:chr1	chr19:583466	8.19446225	-2.8292257	0.14070781	-2.3744229	0.19285348	-0.4548027	0.72960993	7.09978612	9.92901177	7.55458885
A_51_P1511	NM_013706	Cd52	Mus musculu	chr4:1336495	7.06112856	0.05086408	1.03588517	2.76533171	6.79904306	-2.7144676	0.15235749	6.17326071	6.12239663	8.88772834

A_30_P01028	H19		lincRNA:chr7:	chr7:1497620	11.8708588	-5.9833031	0.01580689	-1.857551	0.27594431	-4.1257521	0.05728288	8.50117372	14.4844768	12.6269258
A_51_P47272	NM_145978	Pdlim2	Mus musculu	chr14:705642	7.97818114	-0.8128649	0.56925032	1.88674886	3.69800932	-2.6996138	0.15393426	6.80735492	7.62021983	9.50696869
A_55_P20006	NM_178703	Slc6a1	Mus musculu	chr6:1142548	13.1959059	-1.2242532	0.428019	0.94919442	1.93079423	-2.1734477	0.22168028	12.0633389	13.2875921	14.2367866
A_51_P42293	NM_146954	Olfr535	Mus musculu	chr7:1476787	6.87889421	-3.4153583	0.09372915	-2.8424255	0.13942628	-0.5729328	0.6722488	5.54946382	8.96482217	6.12239663
A_51_P24654	NM_010096	Ebf3	Mus musculu	chr7:1443855	8.51232276	-3.4091889	0.09413083	-1.7034742	0.3070458	-1.7057147	0.30656934	6.80735492	10.2165438	8.51306958
A_30_P01022	chr8:67542714-67561694_		lincRNA:chr8:	chr8:6755960	7.73103229	-3.805949	0.07149821	-3.0079928	0.12430939	-0.7979562	0.5751634	6.19639721	10.0023462	6.99435344
A_65_P10913	NM_009367	Tgfb2	Mus musculu	chr1:1884493	8.61802152	-1.1499945	0.45062696	-2.1932913	0.21865204	1.04329683	2.0609319	8.58245565	9.73245011	7.53915881
A_52_P12787	ENSMUST00000050758		filaggrin Gene	chr3:9308369	6.5470575	-0.0569024	0.96132597	1.95342475	3.87292818	-2.0103271	0.24821683	5.857981	5.91488339	7.86830813
A_52_P40344	NM_0010835	Tns3	Mus musculu	chr11:833224	7.22262199	-2.3619321	0.19453044	-0.9763729	0.50825593	-1.3855592	0.38274112	5.97345821	8.33539035	7.35901741
A_30_P01022	chr18:82862340-82875566		lincRNA:chr18	chr18:828750	7.49054921	-2.3314281	0.19868735	0.42554391	1.34307876	-2.756972	0.14793425	5.79441587	8.12584393	8.55138784
A_55_P19805	NM_146122	Dennd1a	Mus musculu	chr2:3765457	8.8989242	-1.9566834	0.25762002	-2.4202995	0.18681737	0.46361606	1.37899388	8.40123509	10.3579185	7.93761903
A_55_P20699	XR_032158	Gm15707	PREDICTED: M	chr6:1452647	9.28524998	-2.1609025	0.22361634	-0.6535428	0.63571727	-1.5073597	0.35175439	8.06249593	10.2233984	9.56985561
A_55_P21058	NM_030693	Atf5	Mus musculu	chr7:5206768	13.2534814	-2.0381321	0.24347876	0.36249577	1.28564807	-2.4006279	0.18938213	11.7738947	13.8120268	14.1745226
A_55_P21198	NM_010154	Erbb4	Mus musculu	chr1:6808674	9.36692711	1.64002215	3.11670616	2.05234913	4.14780806	-0.412327	0.75141041	9.77615883	8.13613669	10.1884858
A_55_P20383	NM_012006	Acot1	Mus musculu	chr12:853585	10.1024264	-2.8378431	0.13986985	-2.7748936	0.14610793	-0.0629495	0.95730496	9.13549554	11.9733386	9.19844504
A_51_P23638	NM_028942	Slco6c1	Mus musculu	chr1:9896268	6.72877177	-1.1225121	0.45929339	1.02417367	2.03379416	-2.1466857	0.22583082	5.63903917	6.76155123	7.78572491
A_55_P19624	NM_026201	Ccar1	Mus musculu	chr10:622072	8.18434047	2.02244515	4.06271777	-0.209257	0.86498258	2.23170217	4.69687815	9.60238957	7.57994443	7.37068741
A_55_P23619	AK015599	4930480G23R	Mus musculu	chr4:1990665	6.87371345	-0.0872668	0.94130435	1.92682452	3.80217391	-2.0140914	0.24757004	6.17326071	6.26052755	8.18735207
A_30_P01019	chr17:46058814-46059304		lincRNA:chr17	chr17:460591	6.72526135	-1.0345987	0.48815166	1.04708514	2.06635071	-2.0816838	0.23623853	5.68650053	6.72109919	7.76818432
A_52_P79385	NM_025952	Magt1	Mus musculu	chrX:1031695	9.7411338	-2.1087583	0.23184647	-2.0736855	0.23755187	-0.0350728	0.97598253	9.02652344	11.1352818	9.06159621
A_30_P01024	chr9:41422319-41425855_		lincRNA:chr9:	chr9:4142559	7.23072577	-1.1715433	0.44394619	1.21590839	2.32286996	-2.3874517	0.19111969	6.04439412	7.2159374	8.43184579
A_55_P21462	NM_0011127	Ifitm1	Mus musculu	chr7:1481556	7.10242803	-3.6408579	0.08016644	-2.7443212	0.14923717	-0.8965367	0.53717472	5.58996318	9.23082104	6.48649986
A_55_P19597	NM_0011637	Gm10697	Mus musculu	chr3:9376429	6.04024537	-2.1019165	0.23294858	-1.7114571	0.30535152	-0.3904595	0.7628866	5.20945337	7.3113699	5.59991284
A_55_P21595	NM_181277	Col14a1	Mus musculu	chr15:553477	7.06867188	-0.0709433	0.95201536	1.95642772	3.88099808	-2.027371	0.24530168	6.36923381	6.44017706	8.39660478
A_51_P18328	NM_008032	Aff2	Mus musculu	chrX:6712111	10.607141	2.32975638	5.0272045	1.07257884	2.10318949	1.25717754	2.39027654	11.8027856	9.47302922	10.5456081
A_51_P44923	NM_172444	Thsd4	Mus musculu	chr9:5981813	8.83624954	-3.0344091	0.12205395	-3.0397151	0.12160588	0.00530598	1.0036846	7.82654849	10.8609576	7.8212425
A_52_P52156	NM_0011631	Gm70	Mus musculu	chr12:775390	6.76374855	-0.0982795	0.93414634	2.10597237	4.30487805	-2.2042519	0.21699717	5.99623808	6.0945176	8.20048997
A_55_P23860	AK036139	9630039A02R	Mus musculu	chr6:4518369	7.45933951	2.52908255	5.77204503	0.42984831	1.34709193	2.09923424	4.28481894	9.00211178	6.47302922	6.90287753
A_55_P20369	NM_009575	Zic3	Mus musculu	chrX:5528961	9.56854736	2.44253971	5.43597836	0.67285891	1.59422904	1.7696808	3.40978507	10.9726209	8.53008115	9.20294006
A_30_P01029	chr3:36280098-36347798_		lincRNA:chr3:	chr3:3634744	7.19587472	-2.8726691	0.13653388	-2.3452019	0.19679946	-0.5274673	0.69377163	6.06249593	8.93516505	6.58996318
A_66_P10826	NM_148935	Foxn4	Mus musculu	chr5:1147042	7.15986039	-2.230679	0.21305842	-2.0894783	0.23496564	-0.1412007	0.90676417	6.36923381	8.59991284	6.51043452
A_30_P01021	chr4:8606650-8614031_R		lincRNA:chr4:	chr4:8610224	7.55740747	-0.8824304	0.54245283	2.12577897	4.36438679	-3.0082094	0.12429073	6.26052755	7.14295795	9.26873692
A_55_P19573	XM_0014729	Gm2249	PREDICTED: M	chr18:818863	8.04872093	-0.0094603	0.99346405	2.3834596	5.21786492	-2.3929199	0.19039666	7.24792751	7.25738784	9.64084744
A_30_P01027	chr14:55433382-55452757		lincRNA:chr14	chr14:554453	9.54082237	-2.9704702	0.12758493	-1.3451596	0.39361043	-1.6253106	0.3241401	8.00889544	10.9793656	9.63420602
A_51_P20405	NM_010405	Hba-x	Mus musculu	chr11:321776	6.80734635	2.34808038	5.09146341	0.75619016	1.68902439	1.59189022	3.01444043	8.12066989	5.7725895	6.52877967
A_51_P15514	NM_026560	Cdca8	Mus musculu	chr4:1245958	8.36055269	-1.3547145	0.39101218	-2.4607956	0.18164637	1.10608104	2.15260116	8.27767486	9.6323894	7.17159382
A_55_P19872	NM_207574	Olfr1383	Mus musculu	chr11:493381	7.84785519	-0.7521406	0.59372197	1.68210968	3.20896861	-2.4342503	0.18501956	6.78572491	7.53786549	9.21997517
A_51_P50733	NM_0010255	Kcnc2	Mus musculu	chr10:111902	7.96817646	2.66296501	6.33333333	1.24555661	2.37110016	1.4174084	2.67105263	9.32830093	6.66533592	7.91089253
A_30_P01030	chr2:157361470-15737981		lincRNA:chr2:	chr2:1573716	10.4306374	-0.842305	0.55775174	1.59666688	3.02443758	-2.4389719	0.18441503	9.33687844	10.1791834	11.7758503
A_51_P51560	NM_009930	Col3a1	Mus musculu	chr1:4540558	8.89211047	-3.1949051	0.1092038	0.36940377	1.29181885	-3.5643088	0.08453492	6.63903917	9.83394423	10.203348
A_55_P21435	NM_008047	Fstl1	Mus musculu	chr16:378364	9.74989278	-2.70488	0.15337338	-1.7071812	0.30625786	-0.9976988	0.50079818	8.51569984	11.2205799	9.51339863
A_55_P19538	NM_030026	Mccc2	Mus musculu	chr13:100718	7.76572758	1.16891648	2.24842767	-1.0554951	0.48113208	2.22441159	4.67320261	8.89683693	7.72792045	6.67242534
A_51_P13526	NM_016869	Corin	Mus musculu	chr5:7269234	7.25954132	-2.8418806	0.13947896	-1.4790802	0.35871743	-1.3628004	0.38882682	5.857981	8.6998616	7.22078137
A_52_P66286	ENSMUST00000069682		olfactory rece	chr7:1139577	6.60989244	-0.5200071	0.69736842	1.60590184	3.04385965	-2.1259089	0.22910663	5.72792045	6.24792751	7.85382935

A_66_P11509	NM_0011229	Nfia	Mus musculu	chr4:976809	9.64465182	-2.1533576	0.22478885	-2.7002929	0.15386181	0.54693528	1.46097884	9.1091777	11.2625353	8.56224242
A_30_P01022	chr3:126969590-12697262	lincRNA:chr3:	chr3:1269696		6.99847113	2.26805498	4.81673307	-0.4323847	0.74103586	2.70043972	6.5	8.65463603	6.38658105	5.95419631
A_55_P21658	NM_009883	Cebpb	Mus musculu	chr2:1675158	7.37650468	-3.0333862	0.12214052	-0.8543758	0.55310458	-2.1790103	0.22082718	5.63903917	8.67242534	7.81804952
A_30_P01033	chr2:157361470-15737981	lincRNA:chr2:	chr2:1573717		10.014206	-1.4701869	0.36093552	1.38509065	2.61188369	-2.8552776	0.13818974	8.57238443	10.0425714	11.427662
A_30_P01021	chr7:91558475-91733625_	lincRNA:chr7:	chr7:9172553		6.96859054	-0.0694648	0.95299145	2.11902978	4.34401709	-2.1884946	0.21938023	6.2159374	6.28540222	8.404432
A_30_P01030	chr8:72076364-72077503_	lincRNA:chr8:	chr8:7207686		11.2082363	-1.8883894	0.27010844	0.15061483	1.11004243	-2.0390042	0.24333163	9.89910512	11.7874945	11.9381093
A_65_P17218	NM_0011708	Mndal	Mus musculu	chr1:1757876	6.4877133	-0.4284241	0.74307305	1.74746587	3.35768262	-2.1758899	0.22130533	5.61960864	6.0480327	7.79549856
A_51_P12340	NM_009772	Bub1	Mus musculu	chr2:1276270	7.65012703	-0.6920677	0.61896608	-2.2154483	0.21531962	1.52338055	2.87463851	7.9272313	8.61929904	6.40385076
A_52_P60969	NM_020278	Lgi1	Mus musculu	chr19:383823	8.68669083	1.36161663	2.56972973	2.89341469	7.43027027	-1.5317981	0.34584606	8.62996369	7.26834705	10.1617617
A_55_P20739	NM_178776	BC049715	Mus musculu	chr6:1367890	7.8409689	-1.962876	0.25651659	1.07737261	2.11018957	-3.0402486	0.12156092	6.17326071	8.13613669	9.2135093
A_30_P01027	chr13:81773237-81783062	lincRNA:chr13	chr13:817753		6.53214449	-0.3458539	0.78684211	1.98760809	3.96578947	-2.333462	0.19840743	5.63903917	5.98489311	7.9725012
A_51_P19178	NM_133859	Olfml3	Mus musculu	chr3:1035396	7.99302322	-2.1323655	0.22808359	-1.9480054	0.25917431	-0.1843601	0.88003933	7.22078137	9.35314683	7.40514146
A_66_P13777	XM_986896	A830035A12I	PREDICTED: M	chr11:107393	7.76330185	-2.0746388	0.23739496	-1.3199089	0.40056022	-0.7547299	0.59265734	6.82017896	8.89481776	7.57490884
A_55_P21468	NM_146925	Olfr481	Mus musculu	chr7:1152251	7.6877858	0.30703223	1.23716012	2.39915044	5.27492447	-2.0921182	0.23453608	7.09275714	6.78572491	9.18487534
A_52_P22763	NM_0010399	Mtap2	Mus musculu	chr1:6648878	12.0918495	2.9614415	7.78901823	1.33456509	2.52199444	1.6268764	3.08843593	13.6212888	10.6598473	11.9944124
A_55_P21311	NM_029210	Sv2c	Mus musculu	chr13:967460	8.87166078	-2.8701828	0.13676939	-0.7194903	0.60731196	-2.1506924	0.2252045	7.19803571	10.0682185	9.34872815
A_66_P11817	NM_010252	Gabrg1	Mus musculu	chr5:7116938	7.28233862	2.06806739	4.19324578	0.35986079	1.28330206	1.7082066	3.26754386	8.54109662	6.47302922	6.83289001
A_52_P43405	ENSMUST00000115672		baculoviral IA	chr9:7856221	6.68504463	-0.7558953	0.59217877	1.35958189	2.56610801	-2.1154772	0.23076923	5.72792045	6.48381578	7.84339767
A_55_P20843	NM_0011596	Pigp	Mus musculu	chr16:945807	8.24114686	-4.6182338	0.04071675	-3.5147292	0.08749054	-1.1035046	0.46538462	6.33390074	10.9521345	7.43740531
A_55_P19888	NM_013515	Stom	Mus musculu	chr2:3517709	8.97307228	-3.0204835	0.12323778	-1.4712595	0.36066729	-1.549224	0.34169381	7.44983646	10.4703199	8.99906044
A_55_P19886	NM_0011108	Cacna2d1	Mus musculu	chr5:1588015	9.09371264	-3.2896228	0.10226449	-2.2260681	0.21374046	-1.0635548	0.47845172	7.64265344	10.9322763	8.7062082
A_52_P45542	NM_183427	Glra2	Mus musculu	chrX:1615675	7.86922033	3.87373945	14.6592506	1.27453228	2.41920375	2.59920717	6.05953533	10.0268692	6.15312976	7.42766204
A_30_P01026	chr9:121845920-12185812	lincRNA:chr9:	chr9:1218510		6.85560307	2.73770336	6.67007673	-0.249081	0.84143223	2.98678438	7.92705167	8.76376565	6.0260623	5.77698127
A_55_P19948	NM_009569	Zfpm1	Mus musculu	chr8:1248610	8.33828467	-2.0876371	0.2352657	-1.1888666	0.43864734	-0.8987705	0.53634361	7.34281546	9.43045255	8.24158599
A_65_P12152	NM_053268	Rasa2	Mus musculu	chr9:9644008	8.66602834	2.09218951	4.263947	0.2032951	1.15132497	1.88889441	3.70351302	9.99305631	7.90086681	8.1041619
A_51_P41172	NM_172884	2900026A02R	Mus musculu	chr5:1135190	8.67909154	-3.2432982	0.10560147	-1.6529692	0.31798505	-1.590329	0.33209571	7.06788247	10.3111807	8.65821148
A_51_P20640	NM_0010813	Ptprz1	Mus musculu	chr6:2300204	10.1630644	-2.225588	0.2138116	-2.8051921	0.14307147	0.57960415	1.49443915	9.61440312	11.8399911	9.03479896
A_30_P01031	chr6:52046805-52072744_	lincRNA:chr6:	chr6:5205681		6.79230848	-1.938184	0.2609447	-2.2146568	0.21543779	0.27647278	1.21122995	6.23840474	8.17658873	5.96193196
A_51_P27255	NM_011498	Bhlhe40	Mus musculu	chr6:1086163	7.48957442	-2.461219	0.18159307	-1.0429063	0.48534874	-1.4183126	0.37414966	6.19639721	8.65761619	7.61470984
A_66_P12149	NM_177420	Psat1	Mus musculu	chr19:159916	10.3587885	-2.2316758	0.21291127	-1.0553708	0.48117354	-1.176305	0.44248334	9.2227949	11.4544707	10.3990999
A_55_P21817	NM_0011226	Bdh1	Mus musculu	chr16:314572	7.11911074	2.37196878	5.17647059	-0.2428094	0.84509804	2.61477816	6.12529002	8.78135971	6.40939094	6.16658156
A_30_P01033	chr8:67550064-67560987_	lincRNA:chr8:	chr8:6756085		8.18433114	-4.0349353	0.06100472	-3.2642441	0.10407936	-0.7706912	0.5861366	6.58245565	10.6173909	7.35314683
A_52_P10928	NM_010573	Irx1	Mus musculu	chr13:720957	9.05105907	-5.3260892	0.024928	-2.6587444	0.15835733	-2.6673448	0.15741612	6.38658105	11.7126703	9.05392588
A_51_P36849	NM_029537	Tmem98	Mus musculu	chr11:806354	10.0882147	0.73978171	1.66992315	-1.4392309	0.36876384	2.17901261	4.52843518	11.0611461	10.3213644	8.88213353
A_55_P20574	NM_013495	Cpt1a	Mus musculu	chr19:338406	7.9136797	-2.0969102	0.23375835	-1.4640377	0.36247723	-0.6328725	0.64489112	7.00375213	9.10066234	7.63662462
A_30_P01032	chr3:4797557-4798821_F	lincRNA:chr3:	chr3:4798762		7.12003385	2.27103514	4.82669323	-0.0706767	0.95219124	2.34171188	5.06903766	8.65761619	6.38658105	6.31590431
A_30_P01018	chr15:41486232-41499767	lincRNA:chr15	chr15:414864		8.48494341	-0.648369	0.63800116	1.61099264	3.05461941	-2.2593617	0.20886437	7.51569984	8.16406888	9.77506152
A_55_P21486	XM_0014735	Gm9460	PREDICTED: M	chr1:1802491	7.71426789	0.15925571	1.11671088	2.06317333	4.17904509	-1.9039176	0.26721676	7.13271392	6.97345821	9.03663154
A_30_P01031	chr7:48794017-48815817_	lincRNA:chr7:	chr7:4880210		7.25518568	-2.8962408	0.13432122	-0.675316	0.62619503	-2.2209248	0.21450382	5.54946382	8.44570464	7.7703886
A_51_P39484	NR_024599	Gm11346	Mus musculu	chr13:246903	7.59083555	2.0748628	4.21304348	1.91606118	3.77391304	0.15880162	1.11635945	8.33539035	6.26052755	8.17658873
A_30_P01021	chr6:47692090-47713542_	lincRNA:chr6:	chr6:4769423		10.7702504	-2.3726877	0.19308558	0.58939326	1.50461383	-2.9620809	0.12832899	8.99199416	11.3646819	11.9540751
A_52_P11421	ENSMUST00000103464		immunoglobu	chr12:115186	6.5761037	-0.0777811	0.94751381	2.06144206	4.17403315	-2.1392232	0.22700199	5.83710227	5.91488339	7.97632544
A_55_P19558	NM_175290	Nlrp4f	Mus musculu	chr13:652785	6.91405964	-0.6391183	0.64210526	1.67173038	3.18596491	-2.3108486	0.20154185	5.93073734	6.56985561	8.24158599
A_55_P21199	NM_021455	Mlxipl	Mus musculu	chr5:1356137	13.26336	2.04896031	4.13807648	2.30478851	4.94095017	-0.2558282	0.83750621	13.8610707	11.8121104	14.1168989

A_55_P2293(	AK139019	AI662175	Mus musculu	chr16:192314	6.44174918	-2.2471901	0.21063395	-2.2286343	0.2133606	-0.0185558	0.98722045	5.68650053	7.93369065	5.70505635
A_51_P3718(	NM_027512	3830417A13F	Mus musculu	chrX:6143161	6.27400111	-0.1404812	0.90721649	2.16274603	4.47766323	-2.3032272	0.20260936	5.45943162	5.59991284	7.76265887
A_51_P1131	NM_175398	6530418L21F	Mus musculu	chr3:1055208	9.13656265	-2.8845672	0.1354125	-0.5706129	0.67333067	-2.3139542	0.20110847	7.40372219	10.2882893	9.71767642
A_52_P2659	NM_029702	Arfrp1	Mus musculu	chr2:181093(	8.3645935	-0.3740849	0.77159468	2.52189386	5.74335548	-2.8959787	0.13434563	7.27457229	7.64865718	10.170551
A_52_P2540	NM_177396	Il28b	Mus musculu	chr7:2930801	7.33127072	-0.3970296	0.75942029	1.85437155	3.61594203	-2.2514011	0.21002004	6.4484605	6.84549005	8.6998616
A_52_P5390(	NM_009829	Ccnd2	Mus musculu	chr6:1270987	10.9679726	-2.6128016	0.1634814	-2.2009669	0.21749183	-0.4118347	0.75166688	9.9597605	12.5725621	10.3715952
A_30_P0102	chr11:97275905-97310538	lincRNA:chr1	chr11:973098	9.19680375	-1.7515097	0.29699084	0.60774648	1.52387702	-2.3592561	0.19489161	7.82654849	9.57805814	10.1858046
A_55_P2014	NM_181404	Kank1	Mus musculu	chr19:255089	8.83041253	-1.2566047	0.41852777	-2.6950445	0.15442257	1.43843974	2.71027593	8.89102419	10.1476289	7.45258445
A_52_P4294	NM_008694	Ngp	Mus musculu	chr9:1103242	6.80286492	-0.0770109	0.9480198	2.2658587	4.80940594	-2.3428696	0.19711786	5.99623808	6.07324898	8.33910768
A_30_P0102	chr2:129416848-12941741	lincRNA:chr2	chr2:1294171	7.21834629	-0.2593161	0.83548387	1.84086927	3.58225806	-2.1001854	0.23322828	6.43184579	6.6911619	8.53203118
A_51_P3680(	NM_177733	E2f2	Mus musculu	chr4:1357505	9.96557055	-2.3071724	0.20205607	-1.9520855	0.25844237	-0.3550869	0.78182257	9.07815081	11.3853232	9.43323768
A_55_P1969	NM_146831	Olfr133	Mus musculu	chr17:382864	7.02332415	-0.3488472	0.78521127	1.72446582	3.30457746	-2.073313	0.23761321	6.2159374	6.56478462	8.28925043
A_52_P5734(	NM_008644	Prol1	Mus musculu	chr5:8875759	6.3887512	-2.3229694	0.1998557	-2.0660241	0.23881674	-0.2569452	0.83685801	5.52877967	7.85174904	5.78572491
A_30_P0103(	XIST		lincRNA:chrX	chrX:1006561	7.90279089	5.80363663	55.8558559	0.52148844	1.43543544	5.28214819	38.9121339	11.5980525	5.79441587	6.31590431
A_52_P2817(	NM_010518	Igfbp5	Mus musculu	chr1:7290488	11.6980709	-5.5506014	0.02133548	-2.6150006	0.16323241	-2.9356009	0.13070617	8.86933679	14.4199382	11.8049377
A_55_P2186	XR_032647	Gm13337	PREDICTED: I	chr2:2199032	7.48134504	-2.1239053	0.22942503	-0.055543	0.96223224	-2.0683624	0.23842999	6.08392248	8.20782779	8.15228484
A_51_P3706	NM_009718	Neurog2	Mus musculu	chr3:1273378	7.61041326	-3.5809826	0.08356354	-3.3324277	0.09927486	-0.2485549	0.84173913	6.33390074	9.91488339	6.58245565
A_52_P2136	NM_027402	Fndc5	Mus musculu	chr4:1288217	7.72262417	-3.424219	0.09315526	-3.2980419	0.10166945	-0.1261771	0.91625616	6.53915881	9.96337779	6.66533592
A_30_P0102	chr5:77084398-77086144_	lincRNA:chr5	chr5:7708497	9.32792284	1.20615907	2.30722559	-0.9399927	0.52123552	2.14615176	4.42645503	10.4453598	9.23920071	8.29920802
A_30_P0103	chr5:35492530-35492959_	lincRNA:chr5	chr5:3549279	7.39186501	0.30580843	1.23611111	2.1148991	4.33159722	-1.8090907	0.28537074	6.89077093	6.5849625	8.6998616
A_55_P1975(	NM_031397	Bicc1	Mus musculu	chr10:703879	7.13632964	-2.2650733	0.20803911	-1.109872	0.46333514	-1.1552013	0.44900352	5.99623808	8.26131141	7.15143943
A_66_P1399	XM_976924	E030019B13F	PREDICTED: I	chr12:575916	10.3034506	2.32814529	5.02159364	0.48378626	1.39840889	1.84435902	3.59093372	11.6942853	9.36614004	9.84992631
A_55_P1971	NM_010784	Mdk	Mus musculu	chr2:9177005	9.17004775	-1.0668024	0.47737588	-2.151981	0.22500344	1.08517862	2.12163814	9.17617315	10.2429756	8.09099453
A_66_P1174	ENSMUST00(	Pvrl3	poliovirus rec	chr16:463945	9.89090576	-3.8315901	0.0702387	-2.842043	0.13946326	-0.989547	0.50363587	8.28386005	12.1154501	9.2734071
A_51_P4204(	NM_010703	Lef1	Mus musculu	chr3:1309078	7.33800267	-2.7788338	0.14570943	-1.0549665	0.48130841	-1.7238674	0.3027361	5.83710227	8.6159361	7.56096965
A_55_P2060(	NM_010452	Hoxa3	Mus musculu	chr6:5211914	8.28504069	-4.3579907	0.04876566	-2.0483471	0.24176091	-2.3096436	0.20171026	6.06249593	10.4204866	8.37213954
A_55_P2059(	XM_0010001	Fignl2	PREDICTED: I	chr15:100882	10.6890385	-3.4711567	0.09017325	-1.0521488	0.48224936	-2.419008	0.18698469	8.72565028	12.196807	11.1446582
A_55_P2173	NM_175303	Sall4	Mus musculu	chr2:1685804	7.3515852	-2.0433432	0.2426009	-1.5154977	0.34977578	-0.5278455	0.69358974	6.49452228	8.53786549	7.02236781
A_55_P2148	NR_029457	G530011O06	Mus musculu	chrX:166413(	7.14471461	2.03188406	4.08938547	-0.0491875	0.96648045	2.08107161	4.23121387	8.51569984	6.48381578	6.43462823
A_52_P4336	NM_170599	Igsf11	Mus musculu	chr16:390249	7.82499498	-1.2023951	0.43455325	-2.2673218	0.20771513	1.06492664	2.09206349	7.77917215	8.98156728	6.71424552
A_51_P2433(	NM_016905	Galk1	Mus musculu	chr11:11587(	9.95147004	-2.1308959	0.22831603	-0.4317241	0.7413753	-1.6991719	0.30796282	8.67478076	10.8056767	10.3739527
A_55_P1972	ENSMUST00000089883		mannan-bind	chr16:23470(	9.01045791	-2.4044515	0.18888087	-1.0820823	0.47234657	-1.3223692	0.39987771	7.76818432	10.1726359	9.09055354
A_55_P2032	ENSMUST00000122450		RIKEN cDNA I	chr16:98231(	7.04254259	-1.3299326	0.39778684	-2.0245751	0.24577752	0.69464258	1.61848341	6.83077927	8.16071182	6.13613669
A_55_P2020(	NM_025404	Arl4d	Mus musculu	chr11:101528	10.1109013	2.86605347	7.29068047	2.26401223	4.80325444	0.60204124	1.51786264	11.2669329	8.40087944	10.6648917
A_51_P4819	NM_009828	Ccna2	Mus musculu	chr3:3646403	7.60326657	-1.1180105	0.46072874	-2.1281883	0.22874494	1.01017779	2.01415929	7.56732234	8.68533283	6.55714456
A_30_P0102(	chr18:69923410-69965360	lincRNA:chr1	chr18:699267	6.45314147	-2.5899981	0.16608595	-2.5452983	0.17131243	-0.0446998	0.96949153	5.57490884	8.16490693	5.61960864
A_55_P2072(	NM_0010333	Dpcr1	Mus musculu	chr17:357728	6.661202	-2.4353861	0.18487395	-1.2654611	0.41596639	-1.169925	0.44444444	5.45943162	7.89481776	6.62935662
A_30_P0102	chr14:55433382-55452757	lincRNA:chr1	chr14:554451	10.2096206	-3.4560729	0.09112098	-1.5788213	0.33475527	-1.8772516	0.27220179	8.43184579	11.8879187	10.3090973
A_55_P2000	ENSMUST00000024228		Putative unch	chr2:9315147	8.30045	-3.5880938	0.08315266	-2.5661345	0.16885601	-1.0219593	0.49244713	6.76376565	10.3518594	7.78572491
A_55_P2148	XR_031785	Gm2862	PREDICTED: I	chr15:256119	7.73964976	-0.3929788	0.76155556	-2.0403123	0.24311111	1.64733345	3.13254113	8.15776798	8.55074679	6.51043452
A_55_P1978	XM_0014765	LOC1000415	PREDICTED: I	chrY_random	8.55513515	-0.3214414	0.80026991	2.14174519	4.41295547	-2.4631866	0.18134557	7.62692579	7.94836723	10.0901124
A_52_P6128(	NM_009831	Ccng1	Mus musculu	chr11:405624	8.86807693	-2.224439	0.21398194	-1.5472477	0.3421622	-0.6771913	0.6253816	7.90086681	10.1253058	8.57805814
A_30_P0102	chr8:41730287-41746862_	lincRNA:chr8	chr8:4174616	6.62504267	-0.6503779	0.6371134	1.51487061	2.85773196	-2.1652485	0.22294372	5.68650053	6.33687844	7.85174904
A_55_P1977	NM_031161	Cck	Mus musculu	chr9:121399(	8.43008826	-1.8796308	0.27175325	0.15863919	1.11623377	-2.03827	0.2434555	7.12412131	9.00375213	9.16239133

A_55_P2177(	NM_175678	Npsr1	Mus musculu	chr9:2412074	6.18925562	-0.0418202	0.97142857	1.97662549	3.93571429	-2.0184457	0.24682396	5.50250034	5.54432052	7.52094601
A_52_P5295.	NM_198654	Nsl1	Mus musculu	chr1:1929083	8.62963361	-0.5426249	0.6865207	-2.516692	0.17474318	1.9740671	3.92874109	9.10678102	9.64940589	7.13271392
A_55_P2090.	NM_021452	Kcnmb4	Mus musculu	chr10:115855	8.96255576	2.19410456	4.57605552	0.18128377	1.13389242	2.01282078	4.03570518	10.3648642	8.17075965	8.35204343
A_30_P0102!	chr4:101040523-10108024	lincRNA:chr4:	chr4:1010773	6.51349623	-0.2926799	0.81638418	2.18523942	4.5480226	-2.4779193	0.17950311	5.58996318	5.88264305	8.06788247
A_51_P4703.	NM_001042(	Sepp1	Mus musculu	chr15:323041	9.88142428	-4.0473432	0.0604823	-2.7048388	0.15337776	-1.3425045	0.39433551	8.08480839	12.1321516	9.42731284
A_30_P0102.	chr1:34735032-34781146_	lincRNA:chr1:	chr1:3478102	7.00097446	-3.195407	0.10916581	-2.571652	0.16821147	-0.623755	0.64897959	5.72792045	8.92332749	6.35167544
A_30_P0102(	chr8:67542714-67561694_	lincRNA:chr8:	chr8:675527(	9.37099061	-4.5664121	0.04220588	-2.5145732	0.175	-2.0518389	0.24117647	7.16490693	11.731319	9.21674586
A_55_P1996.	NM_198037	Cachd1	Mus musculu	chr4:1006762	10.3565158	-2.6632501	0.15786353	-1.0506427	0.48275305	-1.6126074	0.32700681	8.93122998	11.5944801	10.5438374
A_30_P0101{	chr2:72825297-72826470_	lincRNA:chr2:	chr2:7282531	7.42665945	0.1983068	1.14735099	2.12134482	4.35099338	-1.923038	0.26369863	6.85174904	6.65344224	8.77478706
A_30_P0102.	chr11:76400207-76434732	lincRNA:chr1:	chr11:764126	7.50108475	-2.2078129	0.21646221	-1.4436752	0.3676296	-0.7641378	0.58880517	6.51043452	8.71824745	7.27457229
A_55_P1977(	NM_0010813	Palld	Mus musculu	chr8:6399408	9.19643203	-2.0376796	0.24355516	-0.7298476	0.60296763	-1.307832	0.40392742	8.0812615	10.1189411	9.38909352
A_30_P0101.	chr17:95149413-95174473	lincRNA:chr1	chr17:951743	9.41538202	-0.7433844	0.59733642	-2.4835234	0.17880718	1.74013902	3.34067358	9.74763356	10.491018	8.00749454
A_55_P2000.	NM_011839	Mab21l2	Mus musculu	chr3:863508(	11.4729747	-3.2061777	0.10835385	0.00374878	1.00260184	-3.2099265	0.10807266	9.33427329	12.540451	12.5441997
A_55_P1982(	XR_032923	LOC637146	PREDICTED: Mus musculus		6.85418764	0.29018601	1.22279793	2.24989331	4.75647668	-1.9597073	0.25708061	6.29768055	6.00749454	8.25738784
A_52_P5107{	NM_007801	Ctsh	Mus musculu	chr9:8997082	7.17049849	-2.0090793	0.24843162	-0.6398455	0.64178168	-1.3692338	0.38709677	6.04439412	8.05347341	7.41362793
A_55_P2051.	ENSMUST00000100107		Putative unch	chr16:180296	7.43789173	0.28644821	1.21963394	2.08845094	4.25291181	-1.8020027	0.28677621	6.93270689	6.64625868	8.73470962
A_55_P1965!	ENSMUST00000113343		RIKEN cDNA :	chrX:120257(	7.59818965	2.33824075	5.05685619	0.53921069	1.45317726	1.79903006	3.47986191	8.97727992	6.63903917	7.17824987
A_55_P2125.	NM_0010836	Cacna1d	Mus musculu	chr14:308553	8.08465914	0.0711123	1.05052632	2.26240151	4.79789474	-2.1912892	0.21895568	7.3779335	7.3068212	9.56922271
A_51_P4793.	NM_080575	Acss1	Mus musculu	chr2:1504439	10.8643977	-0.7591378	0.59084934	-2.4691606	0.18059619	1.71002283	3.27166001	11.1813593	11.9404971	9.47133651
A_51_P2920(	NM_008161	Gpx3	Mus musculu	chr11:547234	10.0979944	-3.4152275	0.09373765	-1.4523772	0.36541881	-1.9628503	0.25652115	8.30530178	11.7205293	10.2681521
A_55_P2025(	NM_028760	Cep55	Mus musculu	chr19:381463	7.62746043	-1.6393023	0.32101167	-2.49519	0.17736706	0.85588769	1.80987203	7.36632221	9.00562455	6.51043452
A_30_P0102.	chr3:83089315-83108396_	lincRNA:chr3:	chr3:8310504	8.41773917	-2.4066623	0.18859164	-1.0144255	0.49502543	-1.3922369	0.38097365	7.15143943	9.55810178	8.54367631
A_30_P0103.	chr14:28231577-28242027	lincRNA:chr1	chr14:282388	8.79288448	1.11318722	2.16323024	2.46572768	5.52405498	-1.3525405	0.39160187	8.71310007	7.59991284	10.0656405
A_55_P2001!	NM_145459	Zfp503	Mus musculu	chr14:228039	9.83383113	-4.197527	0.05450276	-1.027361	0.49060674	-3.1701659	0.11109256	7.3779335	11.5754605	10.5480994
A_55_P1967{	NM_008173	Nr3c1	Mus musculu	chr18:395717	8.84423929	-2.634206	0.16107383	-0.4794767	0.71723773	-2.1547293	0.22457523	7.24792751	9.88213353	9.40265681
A_51_P2718(	NM_016751	Clec4f	Mus musculu	chr6:8359533	7.30063622	0.28853837	1.22140221	2.12181066	4.35239852	-1.8332723	0.28062738	6.78572491	6.49718654	8.6189972
A_55_P2005(	NM_018766	Ntsr1	Mus musculu	chr2:1802796	8.84185572	-2.2386209	0.21188878	-0.5262791	0.69434324	-1.7123418	0.30516432	7.52486815	9.76348904	9.23720996
A_55_P1960!	NM_028133	Egln3	Mus musculu	chr12:552813	9.52428566	-5.8607188	0.01720869	-2.0416222	0.24289048	-3.8190966	0.07084959	6.29768055	12.1583993	10.1167771
A_55_P2157.	NM_172849	9430031J16R	Mus musculu	chr1:8126814	7.91523623	2.5849625	6	1.70929885	3.27001862	0.87566365	1.83485194	9.06877828	6.48381578	8.19311463
A_55_P2059(	NM_011254	Rbp1	Mus musculu	chr9:9834690	11.4735682	2.09515875	4.27273176	0.15947159	1.11687799	1.93568715	3.82560297	12.8171835	10.7220248	10.8814964
A_55_P2087.	ENSMUST000	Gm7676	PREDICTED: N	chr8:1389676	7.17520518	-3.4644511	0.09059334	-2.5184558	0.17452967	-0.9459953	0.51907131	5.70505635	9.16950749	6.65105169
A_55_P1981(	NM_207146	Olfr670	Mus musculu	chr7:1121083	7.1190451	-0.0395284	0.97297297	3.14489621	8.84520885	-3.1844246	0.11	6.04439412	6.08392248	9.22881869
A_30_P0103.	chr16:46384974-46391331	lincRNA:chr1(	chr16:463881	8.12922784	-2.7540854	0.14823054	-1.918608	0.26450961	-0.8354774	0.56039755	6.93270689	9.6867923	7.76818432
A_30_P0102.	chr6:127111250-12714292	lincRNA:chr6:	chr6:1271336	13.2784504	-2.1193847	0.23014505	-0.5168311	0.69890531	-1.6025536	0.3292936	12.0378043	14.157189	13.6403579
A_51_P3293.	NM_054087	Slc19a2	Mus musculu	chr1:1661952	9.57433876	-2.2286642	0.21335618	-0.8594304	0.55117014	-1.3692338	0.38709677	8.37503943	10.6037036	9.74427324
A_30_P0102.	chr1:34735032-34781146_	lincRNA:chr1:	chr1:3478094	7.84261206	-2.1454423	0.22602554	-2.1094801	0.23173051	-0.0359622	0.97538101	7.11547722	9.26091953	7.15143943
A_51_P4051(	NM_0010255	Maf	Mus musculu	chr8:1182277	9.62121737	2.06693101	4.18994413	1.72186641	3.29862875	0.34506459	1.27020785	10.4252159	8.3582849	10.0801513
A_51_P5188.	NM_153111	Fev	Mus musculu	chr1:7492831	9.86129601	-5.3518888	0.02448617	-2.4454629	0.18358717	-2.9064259	0.13337629	7.10852446	12.4604132	10.0149503
A_55_P1973(	NM_019919	Ltbp1	Mus musculu	chr17:757905	8.67917021	-3.3511622	0.09799404	-1.4034819	0.37801572	-1.9476804	0.2592327	6.91288934	10.2640516	8.86056972
A_30_P0103(	chr8:67542714-67561694_	lincRNA:chr8:	chr8:6756093	9.50820491	-4.9997389	0.03125566	-3.7704004	0.07328184	-1.2293385	0.42651297	7.43184579	12.4315847	8.66118427
A_51_P5018.	NM_175475	Cyp26b1	Mus musculu	chr6:8452201	9.3716176	-4.1472544	0.05643545	-2.4416422	0.184074	-1.7056122	0.30659111	7.42066205	11.5679165	9.12627427
A_52_P4768.	NM_0010249	Rfx4	Mus musculu	chr10:843311	9.06828939	-3.5115552	0.08768324	-3.0995704	0.11666386	-0.4119848	0.75158868	7.76044275	11.2719979	8.17242751
A_51_P1600{	ENSMUST00000075081		RIKEN cDNA :	chr5:2450388	8.72341628	1.10087936	2.14485388	2.20871	4.62261753	-1.1078306	0.4639912	8.72109919	7.62021983	9.82892983
A_52_P9127.	XM_988035	1700018G05I	PREDICTED: N	chrX:1001243	6.85970587	-0.3512616	0.78389831	2.03733755	4.10487288	-2.3885991	0.19096774	5.94641896	6.29768055	8.33501809

A_30_P0101	chr2:65240793-65292293_	lincRNA:chr2:	chr2:652529	7.50267397	-0.073606	0.95025984	2.14991305	4.43801039	-2.2235191	0.21411843	6.73696559	6.81057163	8.96048468
A_51_P1916	NM_007694	Chgb	Mus musculu	chr2:132620	12.4152996	-3.2664504	0.10392031	-1.5308769	0.34606696	-1.7355735	0.30028962	10.7479583	14.0144087	12.4835318
A_30_P0102	chr1:74084292-74138492_	lincRNA:chr1:	chr1:741040	7.22257354	-2.3285529	0.19908372	-1.9370981	0.26114119	-0.3914548	0.76236045	6.31590431	8.64445719	6.70735913
A_30_P0101	chr7:132218864-13232917	lincRNA:chr7:	chr7:132326	6.50146194	-0.0433274	0.9704142	2.09996244	4.28698225	-2.1432899	0.22636301	5.7725895	5.81591694	7.91587938
A_55_P1956	ENSMUST00000107605	solute carrier	chr7:572190	6.49035414	-2.053466	0.24090462	-0.6908959	0.61946903	-1.3625701	0.38888889	5.35167544	7.40514146	6.71424552
A_52_P6212	NM_010338	Gpr37	Mus musculu	chr6:256190	6.63618023	-2.1432222	0.22637363	-1.4374053	0.36923077	-0.7058169	0.61309524	5.68650053	7.82972274	6.39231742
A_52_P3748	NM_009705	Arg2	Mus musculu	chr12:80252	7.61960319	2.06643312	4.1884984	0.67720741	1.59904153	1.38922571	2.61938062	8.77148947	6.70505635	7.38226376
A_52_P2655	NM_001013	Vmo1	Mus musculu	chr11:70327	6.90011084	-0.3451355	0.78723404	1.7582941	3.38297872	-2.1034296	0.2327044	6.08392248	6.42905797	8.18735207
A_55_P2021	XR_032442	Gm8767	PREDICTED:	chr10:29804	10.0980948	-2.287466	0.20483497	-0.9255192	0.52649102	-1.3619469	0.38905692	8.88162384	11.1690899	10.2435707
A_55_P2100	NM_008963	Ptgds	Mus musculu	chr2:253222	7.39091191	-2.2808136	0.20578167	-1.8724584	0.27310765	-0.4083552	0.75348189	6.49452228	8.77533593	6.90287753
A_55_P2046	NM_026555	Rcn3	Mus musculu	chr7:523383	8.52967293	-2.8071147	0.14288093	-1.5048246	0.35237302	-1.3022901	0.40548204	7.15987134	9.96698603	8.46216141
A_66_P1264	XM_0014747	Gm13429	PREDICTED:	chr2:360683	8.54754048	2.98459697	7.91504178	1.94939187	3.86211699	1.0352051	2.04940498	9.8874745	6.90287753	8.8522694
A_30_P0103	chr2:59235155-59242518_	lincRNA:chr2:	chr2:592378	10.3452771	-3.1954337	0.10916379	-1.1807247	0.44112986	-2.014709	0.24746407	8.60856286	11.8039965	10.6232719
A_52_P2859	NM_001033	Plekhg1	Mus musculu	chr10:63829	7.81411829	-2.0989723	0.23342447	-1.2479275	0.42105263	-0.8510448	0.55438312	6.83077927	8.92975155	7.68182404
A_55_P1991	NM_133241	Mlc1	Mus musculu	chr15:88786	7.6309807	-3.2515969	0.10499577	-2.7179532	0.15198984	-0.5336437	0.6908078	6.36923381	9.62083075	6.90287753
A_30_P0102	chr8:122712014-12273252	lincRNA:chr8:	chr8:122725	9.86672776	-2.4053373	0.18876493	-0.1582043	0.8961398	-2.247133	0.21064228	8.31590431	10.7212416	10.5630373
A_55_P1992	NM_001083	Ptpru	Mus musculu	chr4:131324	10.2180454	-2.4466493	0.18343625	-0.3904779	0.76287686	-2.0561714	0.24045329	8.71710517	11.1637545	10.7732766
A_30_P0102	chr2:157361470-15737981	lincRNA:chr2:	chr2:157371	8.23546558	-1.0501567	0.48291572	1.17720952	2.26138952	-2.2273662	0.21354822	7.14295795	8.19311463	9.37032414
A_55_P2084	NM_013469	Anxa11	Mus musculu	chr14:26694	6.93587582	-0.9255218	0.52649007	1.77282252	3.41721854	-2.6983443	0.15406977	5.72792045	6.65344224	8.42626475
A_55_P1970	XM_0014800	LOC1000483	PREDICTED:	chr16:85858	7.86720803	1.95672548	3.88179916	-0.3028143	0.81066946	2.25953979	4.7883871	9.27262978	7.31590431	7.01309
A_30_P0102	chr1:71939741-71947891_	lincRNA:chr1:	chr1:719402	6.49244107	-2.416613	0.18729535	-2.0806293	0.23641126	-0.3359837	0.79224377	5.57490884	7.99152185	5.91089253
A_55_P2012	NM_026470	Spata6	Mus musculu	chr4:111501	7.85464037	-2.1765093	0.22121034	-1.7037281	0.30699177	-0.4727812	0.72057416	6.97154355	9.14805282	7.44432473
A_52_P2441	NM_025478	Isoc1	Mus musculu	chr18:58838	10.4822616	2.30608008	4.94537554	0.96858564	1.95692117	1.33749444	2.52712047	11.6967865	9.39070637	10.359292
A_55_P2067	NM_026280	Mxra7	Mus musculu	chr11:11667	9.58274891	-2.3604708	0.19472759	-0.3139213	0.80445226	-2.0465495	0.24206234	8.11374217	10.4742129	10.1602916
A_30_P0102	chr14:26842169-26845456	lincRNA:chr1	chr14:26845	6.96332444	-2.2713865	0.20713073	-1.4450117	0.36728919	-0.8263748	0.56394453	5.93073734	8.20212382	6.75711217
A_51_P3434	NM_026331	Slc25a37	Mus musculu	chr14:69860	8.64770758	-2.0014684	0.24974568	-1.8785517	0.2719566	-0.1229167	0.91832918	7.93957921	9.94104761	8.06249593
A_55_P2002	XM_979344	Gsdmcl2	PREDICTED:	chr15:63720	6.95311186	-0.4278302	0.743379	1.75190779	3.36803653	-2.179738	0.22071584	6.08392248	6.51175265	8.26366044
A_52_P4607	NM_198408	Crhbp	Mus musculu	chr13:96201	9.08516567	-3.1360437	0.11375141	-0.2416906	0.84575366	-2.8943531	0.13449709	7.07503339	10.2110771	9.96938652
A_55_P2027	NM_020275	Tnfrsf10b	Mus musculu	chr14:70184	8.10179306	-2.1787387	0.22086877	-2.0223885	0.24615031	-0.1563501	0.89729225	7.32343012	9.5021688	7.47978026
A_55_P2025	NM_007402	Adam7	Mus musculu	chr14:69115	6.33996793	1.86114071	3.63294798	-0.3902341	0.76300578	2.25137482	4.76136364	7.71080643	5.84966573	5.45943162
A_51_P2539	NM_008791	Pcp4	Mus musculu	chr16:96747	10.1761854	-2.7144809	0.15235609	-0.4906489	0.7117049	-2.223832	0.214072	8.53008115	11.244562	10.7539131
A_55_P2008	NM_010585	Itpr1	Mus musculu	chr6:108501	7.75388279	-2.2767632	0.20636023	-2.1486456	0.22552423	-0.1281175	0.91502463	6.9522559	9.22901905	7.08037342
A_55_P2025	NM_172770	Ttc12	Mus musculu	chr9:492533	6.75716701	-2.2821151	0.20559611	-1.7404801	0.29927007	-0.5416351	0.68699187	5.81591694	8.09803208	6.357552
A_55_P2125	NM_010574	Irx2	Mus musculu	chr13:72771	6.90478938	-3.38882	0.09546926	-1.9563135	0.25768608	-1.4325065	0.37048666	5.29768055	8.68650053	6.73018706
A_55_P2429	NM_027435	Atad2	Mus musculu	chr15:57957	8.18806056	-1.3367759	0.39590444	-2.193088	0.21868285	0.85631217	1.81040462	8.027906	9.36468186	7.17159382
A_51_P4056	NM_008681	Ndrg1	Mus musculu	chr15:66761	7.28413828	-2.2767682	0.20635951	0.11511709	1.08306295	-2.3918853	0.19053325	5.72792045	8.00468865	8.11980574
A_30_P0101	chr9:121845920-12185812	lincRNA:chr9:	chr9:121850	6.95829911	2.26303441	4.8	0.12217187	1.08837209	2.14086254	4.41025641	8.42626475	6.16323035	6.28540222
A_66_P1024	ENSMUST00000103313	Mouse Ig rea	chr6:679044	6.8378229	-0.7838322	0.58082192	1.76204295	3.39178082	-2.5458751	0.17124394	5.72792045	6.51175265	8.2737956
A_51_P2201	NM_008716	Notch3	Mus musculu	chr17:32257	9.98039678	-4.437578	0.04614832	-3.6180166	0.08144576	-0.8195614	0.56661417	8.22801697	12.665595	9.04757838
A_30_P0103	chr9:121845920-12185812	lincRNA:chr9:	chr9:121848	7.67992897	3.13953216	8.81238274	0.48116709	1.39587242	2.65836507	6.31317204	9.61256138	6.47302922	6.95419631
A_55_P2116	NM_009501	Vax1	Mus musculu	chr19:59240	7.93060472	-2.2775191	0.20625212	-0.7449947	0.59667006	-1.5325244	0.34567198	6.66059021	8.93810933	8.19311463
A_52_P4748	NM_001037	4921506M07	Mus musculu	chr12:58718	7.88516366	-3.0276249	0.12262925	-1.2928005	0.40815796	-1.7348244	0.30044558	6.29768055	9.32530546	8.03250496
A_55_P1984	NM_019980	Litaf	Mus musculu	chr16:10960	7.67634975	-1.6189418	0.32557418	-2.5583438	0.16977033	0.93940194	1.91773309	7.44983646	9.06877828	6.51043452
A_55_P2015	NM_016868	Hif3a	Mus musculu	chr7:176169	8.93207148	-3.2909366	0.1021714	-1.8141052	0.28438057	-1.4768314	0.35927702	7.34281546	10.6337521	8.8196469

A_55_P1985	NM_178256	Reps2	Mus musculu	chrX:158849	8.09801859	2.08441321	4.24102564	1.14253911	2.20769231	0.94187409	1.92102207	9.10678102	7.02236781	8.16490693
A_52_P4427	NM_172399	A930038C07	Mus musculu	chr6:656559	7.77335386	-2.4256921	0.18612037	-1.3607057	0.38939177	-1.0649864	0.47797716	6.60979435	9.03548645	7.67478076
A_51_P2986	NM_010713	Lhx8	Mus musculu	chr3:153969	10.8639361	5.56821886	47.4461415	3.93616451	15.3074759	1.63205436	3.0995405	13.2640271	7.69580827	11.6319728
A_51_P3573	NM_033444	Clic1	Mus musculu	chr17:35195	12.3363536	-3.1229011	0.11479239	-1.3983293	0.37936821	-1.7245718	0.30258832	10.7205293	13.8434304	12.4451011
A_55_P2052	NM_177753	Sox21	Mus musculu	chr14:11863	9.7478171	-3.6086969	0.08197359	-1.4757753	0.35954013	-2.1329217	0.22799567	7.83394423	11.4426412	9.9668659
A_55_P2142	NM_013610	Ninj1	Mus musculu	chr13:49291	7.31369248	-2.1486957	0.2255164	-1.1753695	0.44277035	-0.9733262	0.50933041	6.27301849	8.42171421	7.24634474
A_55_P1966	NM_009555	Zfp40	Mus musculu	chr17:23310	7.81610466	1.4198513	2.67557932	-0.6122207	0.65418895	2.03207201	4.08991826	8.96674576	7.54689446	6.93467375
A_51_P4611	NM_029674	Got1l1	Mus musculu	chr8:283098	6.81082814	-2.2395227	0.21175637	-0.9636785	0.51274788	-1.2758442	0.41298343	5.63903917	7.87856187	6.91488339
A_52_P4683	NM_001122	Bdh1	Mus musculu	chr16:31458	9.227596	3.04805463	8.27095909	0.76342725	1.69751844	2.28462738	4.87238246	11.0051567	7.95710204	8.72052929
A_30_P0102	chr18:5951953-5952386_F	lincRNA:chr1	chr18:59519	8.33750773	0.47053261	1.38562092	2.76982704	6.82026144	-2.2992944	0.20316243	7.72792045	7.25738784	10.0272149
A_55_P2025	ENSMUST000	Proca1	protein intera	chr11:78008	7.66075911	-1.0911479	0.46938776	1.05261463	2.07428571	-2.1437625	0.22628886	6.58245565	7.67360353	8.72621816
A_51_P3832	NM_175473	Fras1	Mus musculu	chr5:972132	9.52339773	-2.2843163	0.20528267	-1.5828248	0.33382762	-0.7014915	0.61493615	8.52812848	10.8124447	9.22961997
A_55_P1984	NM_001039	Pde4dip	Mus musculu	chr3:974938	9.03543566	2.04417067	4.12436116	0.47048624	1.38557638	1.57368443	2.97663934	10.2413874	8.19721669	8.66770293
A_55_P2337	NM_183151	Mid1	Mus musculu	chrX:166428	7.62823031	0.78048941	1.71771348	-1.5467831	0.34227241	2.32727252	5.0185567	8.66415095	7.88366154	6.33687844
A_51_P4035	NM_008499	Lhx5	Mus musculu	chr5:120891	7.48093549	-2.7100577	0.15282392	-1.3028823	0.40531561	-1.4071754	0.37704918	6.10852446	8.81858218	7.51569984
A_55_P1998	NM_183161	Slc17a9	Mus musculu	chr2:180476	8.40469569	-2.6530902	0.15897919	-1.0467727	0.48404979	-1.6063175	0.32843561	6.98489311	9.6379833	8.59121065
A_51_P2646	NM_016669	Crym	Mus musculu	chr7:127330	7.32088019	-3.5736375	0.08399007	-3.2406259	0.10579726	-0.3330116	0.79387755	6.01866384	9.5923013	6.35167544
A_30_P0101	chr15:96984422-97000322	lincRNA:chr1	chr15:96991	7.829008	-2.8643859	0.13732004	-1.3494492	0.39244186	-1.5149367	0.34991182	6.36923381	9.23361968	7.88417052
A_51_P1618	NM_010189	Fcgrt	Mus musculu	chr7:523506	6.71628987	-0.3473507	0.7860262	1.73349644	3.32532751	-2.0808471	0.23637557	5.9068906	6.25424129	7.98773773
A_51_P3869	NM_009406	Tnni3	Mus musculu	chr7:447210	7.7176448	-1.1779871	0.44196772	1.04959327	2.0699462	-2.2275804	0.21351652	6.58245565	7.76044275	8.81003601
A_52_P9346	NM_011521	Sdc4	Mus musculu	chr2:164251	7.84501552	-3.5349218	0.08627451	-2.1239887	0.22941176	-1.4109331	0.37606838	6.19639721	9.73131903	7.60733031
A_30_P0102	chr2:33500594-33501874_	lincRNA:chr2	chr2:335014	8.13490514	-4.6350365	0.04024528	-2.3131084	0.20122641	-2.3219281	0.2	5.81591694	10.4509535	8.13784503
A_30_P0102	chr2:167404245-16741434	lincRNA:chr2	chr2:167404	7.71050343	-0.2984619	0.81311881	2.21022527	4.62747525	-2.5086872	0.17571543	6.77478706	7.07324898	9.28347426
A_55_P2092	NM_001164	Tgif1	Mus musculu	chr17:71193	7.51029482	-2.1801208	0.22065728	-1.4931236	0.35524257	-0.6869971	0.62114537	6.55458885	8.73470962	7.24158599
A_55_P2037	NM_133786	Smc4	Mus musculu	chr3:688384	8.44806696	-0.4260722	0.74428536	-2.3515848	0.19593067	1.9255126	3.79871795	8.94788041	9.37395266	7.02236781
A_30_P0101	chr4:33367670-33368135_	lincRNA:chr4	chr4:333678	7.71289329	-1.3813235	0.38386648	0.805347	1.74756606	-2.1866705	0.21965778	6.52356196	7.90488546	8.71023246
A_55_P2011	XM_916287	LOC637082	PREDICTED: M	chr3:127501	6.99105042	-2.6131444	0.16344256	-2.5787109	0.16739045	-0.0344335	0.97641509	6.10852446	8.72166886	6.14295795
A_55_P2031	NM_146945	Olfr345	Mus musculu	chr2:364964	6.8008649	-0.1191717	0.92071611	2.44357952	5.4398977	-2.5627512	0.16925247	5.9068906	6.0260623	8.46964182
A_52_P5851	NM_009911	Cxcr4	Mus musculu	chr1:130485	12.1941052	-2.2122838	0.21579244	-1.5726666	0.33618644	-0.6396172	0.64188325	11.2434715	13.4557553	11.8830887
A_66_P1283	XM_0014723	Gm8635	PREDICTED: M	chr14:53200	7.40728085	-0.4150375	0.75	1.77429113	3.42069892	-2.1893286	0.21925344	6.53915881	6.95419631	8.72848744
A_51_P2910	NM_172710	Sel1l3	Mus musculu	chr5:534986	10.4836688	2.15912163	4.46642839	1.24017206	2.36226704	0.91894957	1.89073814	11.5096925	9.35057091	10.590743
A_30_P0102	chr4:90877414-90917198_	lincRNA:chr4	chr4:909168	6.99483132	4.29974133	19.6947791	0.55963432	1.47389558	3.74010701	13.3623978	9.67478076	5.37503943	5.93467375
A_30_P0101	chr5:144517700-14452607	lincRNA:chr5	chr5:144525	6.81886309	1.89188843	3.7112069	-0.2543547	0.83836207	2.14624308	4.42673522	8.16490693	6.27301849	6.01866384
A_51_P4511	NM_026785	Ube2c	Mus musculu	chr2:164598	8.22694982	-0.9542649	0.51610449	-2.4452321	0.18361654	1.49096719	2.81077348	8.40585058	9.36011549	6.91488339
A_55_P2128	NM_009933	Col6a1	Mus musculu	chr10:76171	7.78027627	-2.214279	0.21549421	-0.0894313	0.93989314	-2.1248476	0.22927523	6.33390074	8.54817971	8.45874836
A_51_P4382	NM_030749	Sil1	Mus musculu	chr18:35426	10.1581329	-2.7344706	0.15025963	-1.5137285	0.35020497	-1.2207421	0.42906196	8.83972869	11.5741993	10.0604708
A_55_P2044	NM_028263	Fgfbp3	Mus musculu	chr19:36992	10.0138945	-4.1628125	0.05583012	-3.5459276	0.08561886	-0.6168849	0.65207739	8.42066205	12.5834746	9.03754695
A_52_P6369	ENSMUST00000105113		PREDICTED: M	chrX:409456	6.56751867	-0.0246621	0.98305085	2.07928891	4.2259887	-2.103951	0.23262032	5.857981	5.88264305	7.96193196
A_52_P1082	NM_011054	Pde1c	Mus musculu	chr6:560302	6.68234884	2.4502054	5.46493902	0.27907262	1.21341463	2.17113278	4.50376884	8.2227949	5.7725895	6.05166212
A_55_P1987	NM_001123	Gm3448	Mus musculu	chr17:15132	9.33148442	-2.1723813	0.22184419	-0.5202225	0.69726427	-1.6521588	0.31816371	8.05663772	10.2290191	9.70879651
A_51_P4591	NM_013754	Insl6	Mus musculu	chr19:29395	6.82973001	-2.0929917	0.23439412	-1.6855076	0.31089351	-0.4074841	0.75393701	5.99623808	8.08922977	6.40372219
A_55_P1965	NM_008262	Onecut1	Mus musculu	chr9:747109	7.73915779	-2.7765618	0.14593909	-1.1172328	0.46097716	-1.6593289	0.31658637	6.26052755	9.03708932	7.91985649
A_55_P1960	NM_146069	Lrrc33	Mus musculu	chr16:32142	7.89031118	-1.515738	0.34971751	0.57295816	1.48757062	-2.0886962	0.23509305	6.6888331	8.20457114	8.77752931
A_55_P2105	NM_008377	Lrig1	Mus musculu	chr6:945545	8.90624169	-1.5579118	0.33964232	-2.1047667	0.23248882	0.5468549	1.46089744	8.56922271	10.1271346	8.02236781

A_55_P2180!	NM_027697	4933413G19I	Mus musculu	chr6:1283349	6.96592133	-0.0505079	0.96559633	2.39860643	5.27293578	-2.4491143	0.1831231	6.13271392	6.18322182	8.58182825
A_55_P1952!	XR_032445	LOC1000411	PREDICTED: M	chrY_random	5.86557408	0.15062186	1.11004785	2.0789105	4.22488038	-1.9282886	0.26274066	5.27301849	5.12239663	7.20130713
A_30_P0102(	chr2:157361470-15737981		lincRNA:chr2:	chr2:1573717	9.21154325	-1.2441386	0.42215989	1.23459677	2.35315568	-2.4787354	0.1794016	7.97058527	9.21472386	10.4493206
A_30_P0103	chr4:149383134-14945125		lincRNA:chr4:	chr4:1494336	10.0651331	-2.2666022	0.20781875	-0.340914	0.78954092	-1.9256882	0.26321467	8.66770293	10.9343052	10.5933911
A_55_P2124	NM_0011099	Col18a1	Mus musculu	chr10:765158	7.24588118	-2.9565158	0.12882497	-2.5735299	0.16799266	-0.3829859	0.76684882	6.13271392	9.08922977	6.51569984
A_52_P5403!	ENSMUST00000118300		Novel proteir	chrX:2353511	6.82332153	0.12972617	1.09408602	2.47764948	5.56989247	-2.3479233	0.19642857	6.08392248	5.95419631	8.43184579
A_52_P5705	NM_172800	Sdk2	Mus musculu	chr11:113655	10.0271652	-2.4727266	0.18015036	-1.1618877	0.44692737	-1.3108389	0.40308642	8.76597668	11.2387033	10.0768156
A_30_P0102!	chr13:98062189-98066613		lincRNA:chr1	chr13:980664	7.42222379	0.09921716	1.07119205	2.20712748	4.61754967	-2.1079103	0.23198279	6.7526594	6.65344224	8.86056972
A_52_P3263!	NM_010155	Erf	Mus musculu	chr7:260284(	11.1980161	-2.1741632	0.22157036	-0.6552361	0.63497159	-1.5189271	0.34894532	9.96698603	12.1411492	11.4859131
A_52_P6495(	NM_175256	Heg1	Mus musculu	chr16:337673	8.20883163	-1.4373147	0.36925396	-2.0580483	0.24014067	0.62073363	1.5376569	7.93663794	9.37395266	7.31590431
A_51_P4776	NM_008939	Prss12	Mus musculu	chr3:1232094	9.30796732	-2.307132	0.20206173	-0.8602752	0.55084746	-1.4468568	0.36681975	8.05663772	10.3637697	9.5034945
A_55_P2015	NM_0011014	Zcchc24	Mus musculu	chr14:265311	8.12913037	-3.5124085	0.08763139	-3.3400263	0.09875336	-0.1723822	0.88737624	6.90086681	10.4132753	7.07324898
A_55_P1983	NM_027406	Aldh1l1	Mus musculu	chr6:9054909	8.56737352	-3.4720907	0.09011489	-2.1566915	0.22426999	-1.3153992	0.4018143	6.97154355	10.4436343	8.28694274
A_55_P2111	NM_007752	Cp	Mus musculu	chr3:1988907	7.18444232	-2.8724005	0.1365593	-2.9730567	0.12735639	0.10065621	1.07226107	6.26052755	9.13292808	6.15987134
A_55_P2129	NM_011565	Tead2	Mus musculu	chr7:5248893	10.3382544	-1.646807	0.31934616	-3.2654364	0.10399338	1.61862943	3.07083168	10.3288619	11.9756689	8.71023246
A_51_P2367	NR_002839	Dlx6as	Mus musculu	chr6:6814542	11.4258138	2.08390155	4.23952182	1.66439697	3.16981132	0.41950458	1.33746819	12.2602825	10.176381	11.8407779
A_52_P2090(	NM_011658	Twist1	Mus musculu	chr12:346444	7.43175316	-3.0360993	0.12191104	-1.6161961	0.3261944	-1.4199033	0.37373737	5.94641896	8.9825183	7.36632221
A_51_P4146	NM_015734	Col5a1	Mus musculu	chr2:2789289	8.22783202	-2.1768492	0.22115823	-1.6808376	0.3119015	-0.4960116	0.70906433	7.33687844	9.5137276	7.83289001
A_30_P0103	chr8:94841965-94881130_		lincRNA:chr8:	chr8:9485031	6.88746068	-2.6700582	0.15712034	-1.5948518	0.33105623	-1.0752063	0.47460317	5.63903917	8.30909735	6.71424552
A_55_P2046	NM_009242	Sparc	Mus musculu	chr11:55208(	10.3816399	-4.9671217	0.03197035	-1.188063	0.43889173	-3.7790587	0.07284336	7.46624644	12.4333681	11.2453051
A_30_P0102(	chr2:33500594-33501874_		lincRNA:chr2:	chr2:3350179	9.59900479	-6.3634722	0.01214518	-2.4497121	0.18304723	-3.9137601	0.06634998	6.17326071	12.5367329	10.0870208
A_55_P1987	NM_0011438	Fam196a	Mus musculu	chr7:1420737	8.32954377	2.42889463	5.38480697	1.36685566	2.57907846	1.06203897	2.08788025	9.49318831	7.06429368	8.43114934
A_55_P2068	XM_0014737	Gm2488	PREDICTED: M	chr15:132458	11.4690907	2.22338327	4.66987284	2.1909389	4.56602543	0.03244437	1.0227435	12.2210332	9.99764995	12.1885888
A_52_P3909	NM_016803	Chst3	Mus musculu	chr10:596445	7.06150675	-1.8977951	0.26835317	-2.0946369	0.23412698	0.19684173	1.14618644	6.49452228	8.39231742	6.29768055
A_51_P2538(	NM_0010811	Mki67	Mus musculu	chr7:1428816	9.40420557	-0.9672433	0.51148248	-2.6026472	0.16463612	1.63540395	3.10674525	9.62692579	10.5941691	7.99152185
A_55_P2141	XM_0014749	Gm1758	PREDICTED: M	chr16:145092	7.18858562	-0.4394217	0.73743017	2.55373118	5.87150838	-2.9931528	0.12559467	6.04439412	6.48381578	9.03754695
A_55_P2174!	NM_0010133	Dtx3l	Mus musculu	chr16:359329	7.20812885	-0.6943737	0.61797753	1.64644747	3.13061798	-2.3408212	0.19739794	6.19639721	6.89077093	8.5372184
A_51_P2690	NM_175329	Chchd10	Mus musculu	chr10:754003	9.53865115	-2.9670071	0.12789156	-1.8478269	0.27781052	-1.1191802	0.46035534	8.17658873	11.1435958	9.29576893
A_30_P0103	chr14:55433382-55452757		lincRNA:chr1	chr14:554452	10.3675659	-2.3172401	0.20065095	-1.1135906	0.46214241	-1.2036495	0.43417557	9.19393598	11.5111761	10.3975855
A_55_P2049	NM_173749	Pamr1	Mus musculu	chr2:1024831	7.38496134	-1.4013626	0.37857143	-2.076715	0.23705357	0.6753524	1.59698682	7.14295795	8.54432052	6.46760555
A_55_P1961	NM_207176	Tes	Mus musculu	chr6:1705571	9.95177718	2.23780097	4.7167756	1.09047955	2.12944808	1.14732141	2.21502259	11.0801513	8.84235034	9.9328299
A_51_P2842	NM_025360	Tmed3	Mus musculu	chr9:8959411	11.7329955	-2.1867752	0.21964184	-0.8603368	0.55082396	-1.3264384	0.39875143	10.5619243	12.7486995	11.8883627
A_55_P2111	NM_178444	Egfl7	Mus musculu	chr2:2644814	9.13886427	-3.4803895	0.08959801	-2.0237511	0.24591794	-1.4566384	0.36434109	7.49318831	10.9735778	8.94982671
A_55_P2096	NM_008113	Arhgdig	Mus musculu	chr17:263364	8.14229665	-1.2901026	0.40892193	0.83542823	1.78438662	-2.1255309	0.22916667	7.00375213	8.29385478	9.12928302
A_51_P4952	NM_027127	Gpx8	Mus musculu	chr13:113833	7.48669376	-2.8942906	0.13450292	-3.0758668	0.11859649	0.18157621	1.13412229	6.58245565	9.4767462	6.40087944
A_66_P1182	NM_0010118	Olfr1490	Mus musculu	chr19:137298	6.7255574	0.10597237	1.07621951	2.75293131	6.74085366	-2.6469589	0.15965626	5.87856187	5.7725895	8.52552081
A_30_P0102!	chr1:51753968-51803787_		lincRNA:chr1:	chr1:517541(	7.03163865	-0.6767051	0.62559242	1.60832344	3.04897314	-2.2850285	0.20518135	6.04439412	6.72109919	8.32942263
A_55_P2120!	XM_0014746	LOC1000456	PREDICTED: M	chr18:681176	6.74268533	0.25007087	1.18926554	2.33005598	5.02824859	-2.0799851	0.23651685	6.13271392	5.88264305	8.21269903
A_52_P5375	NM_011635	Trap1a	Mus musculu	chrX:1358713	8.35324912	-3.8402946	0.06981619	-1.140624	0.45356337	-2.6996706	0.15392819	6.17326071	10.0135553	8.87293134
A_51_P3540	NM_153153	Svil	Mus musculu	chr18:511898	7.1808054	-2.1007658	0.23313447	-1.870282	0.27351996	-0.2304838	0.85234899	6.40372219	8.50448798	6.63420602
A_55_P2006	NM_0011594	Rbp4	Mus musculu	chr19:381911	7.53817057	-2.7827529	0.14531415	-1.2765676	0.41277641	-1.5061854	0.35204082	6.10852446	8.89127741	7.61470984
A_30_P0102(	chr17:86560289-86565864		lincRNA:chr1	chr17:865654	7.22907095	-2.4180329	0.18711111	-1.5469947	0.34222222	-0.8710382	0.54675325	6.13271392	8.55074679	7.00375213
A_55_P2021(	XM_0014725	Ighg	PREDICTED: Mus musculus		7.03153862	0.64485656	1.56358382	2.90076213	7.46820809	-2.2559056	0.20936533	6.49452228	5.84966573	8.75042785
A_66_P1207	NM_011740	Ywhaz	Mus musculu	chr15:367012	13.1908045	2.03287208	4.09218703	0.66329554	1.58369612	1.36957655	2.58394712	14.324954	12.2920819	12.9553775

A_55_P2058	NM_0010393	Pde4dip	Mus musculu	chr3:9749380	9.15814529	2.17926114	4.52921536	0.61593815	1.53255426	1.56332299	2.95533769	10.4056733	8.22641219	8.84235034
A_51_P1838	NM_015798	Fbxo15	Mus musculu	chr18:851506	7.49128528	-2.1556976	0.22442455	-2.4486335	0.18318414	0.29293589	1.22513089	6.87036472	9.0260623	6.57742883
A_52_P1592	NM_178665	Lpp	Mus musculu	chr16:249888	8.04203803	-1.8933927	0.26917331	-2.1402363	0.22684263	0.24684356	1.18660812	7.49318831	9.38658105	7.24634474
A_55_P2019	NM_178396	Car12	Mus musculu	chr9:6661454	7.01562343	-2.6278592	0.161784	-2.048105	0.24180149	-0.5797542	0.66907776	5.94641896	8.57427815	6.52617317
A_30_P0102	chr1:84947500-85045150_	lincRNA:chr1	chr1:8503602		6.75354676	-0.2368029	0.84862385	1.94777767	3.85779817	-2.1845805	0.21997622	5.94641896	6.18322182	8.13099949
A_55_P1988	NM_007515	Slc7a3	Mus musculu	chrX:982747	9.33170308	-2.9345915	0.13079765	0.1782133	1.13148173	-3.1128048	0.11559855	7.31590431	10.2504958	10.4287091
A_55_P2182	NM_175087	Aqp6	Mus musculu	chr15:994358	7.3767249	-2.3714387	0.19325281	-1.4317582	0.37067888	-0.9396805	0.52134831	6.27301849	8.64445719	7.21269903
A_52_P2874	NM_145399	Scgn	Mus musculu	chr13:240546	7.03884015	2.76854591	6.81420765	0.55576252	1.46994536	2.21278339	4.63568773	8.69928325	5.93073734	6.48649986
A_55_P2057	NM_001159	Ppp1r16b	Mus musculu	chr2:1585885	9.80919013	-1.1951594	0.4367382	0.85349028	1.80686695	-2.0486497	0.24171021	8.72792045	9.92307983	10.7765701
A_30_P0102	chr8:122712014-12273252	lincRNA:chr8	chr8:1227244		8.39455307	-2.8366047	0.13998996	-0.1070266	0.92849975	-2.7295781	0.15077006	6.53915881	9.37576349	9.26873692
A_55_P1991	XM_0014739	2600006L11R	PREDICTED: M	chr9:6392858	7.78989209	-0.5952252	0.66194111	2.19745521	4.58669575	-2.7926804	0.14431764	6.66059021	7.25581542	9.45327063
A_55_P2074	NM_010484	Slc6a4	Mus musculu	chr11:768457	9.37305752	-6.255704	0.01308716	-2.027921	0.24520817	-4.227783	0.05337164	5.87856187	12.1342659	10.1063448
A_65_P0897	NM_010171	F3	Mus musculu	chr3:1214346	8.07234449	-1.9271854	0.26294166	0.15176598	1.11092851	-2.0789513	0.23668639	6.73696559	8.66415095	8.81591694
A_51_P4548	NM_023456	Npy	Mus musculu	chr6:4977374	13.9671869	0.51554978	1.42953881	2.56305144	5.90956297	-2.0475017	0.24190263	13.4565362	12.9409865	15.5040379
A_55_P1979	NM_007806	Cyba	Mus musculu	chr8:1249487	8.43924447	-2.6119576	0.16357707	-1.8402915	0.27926536	-0.7716661	0.58574063	7.3113699	9.92332749	8.08303603
A_52_P5223	NM_178084	B230120H23I	Mus musculu	chr2:7222753	9.73747397	-2.3428469	0.19712097	0.09275346	1.06640353	-2.4356003	0.18484651	8.14465824	10.4875051	10.5802586
A_55_P1974	ENSMUST00000136202		fibrous sheat	chr2:8280559	6.36443813	0.03464614	1.02430556	2.30378075	4.9375	-2.2691346	0.20745429	5.61960864	5.5849625	7.88874325
A_30_P0101	chr17:46972160-46997919	lincRNA:chr1	chr17:469944		8.81816955	-0.3656057	0.77614293	1.89282235	3.71361009	-2.2584281	0.20899958	7.94349161	8.30909735	10.2019197
A_30_P0102	chr6:134875724-13493197	lincRNA:chr6	chr6:1348992		7.01387277	-1.4922347	0.35546152	-2.3649159	0.19412854	0.87268117	1.83106267	6.80735492	8.29958963	5.93467375
A_55_P2034	ENSMUST00000036223		SH3 domain c	chr10:585675	8.12811511	2.31657486	4.98148148	0.55799545	1.47222222	1.75857941	3.3836478	9.48649986	7.169925	7.72792045
A_30_P0103	chr6:52046805-52072744_	lincRNA:chr6	chr6:5205685		6.82053796	-1.9564901	0.25765453	-2.0991785	0.2333911	0.14268842	1.1039604	6.2159374	8.17242751	6.07324898
A_55_P2016	NM_010177	Fasl	Mus musculu	chr1:1637111	8.44162336	2.01598079	4.04455446	2.08914235	4.2549505	-0.0731616	0.95055265	9.08922977	7.07324898	9.16239133
A_51_P2556	NM_008594	Mfge8	Mus musculu	chr7:8627874	10.8236447	-3.2992285	0.10158586	-2.0536776	0.24086929	-1.2455508	0.42174684	9.30871824	12.6079467	10.5542691
A_52_P4753	NM_011445	Sox6	Mus musculu	chr7:1226154	11.792077	3.31610051	9.9596877	1.96812894	3.91260357	1.34797156	2.5455397	13.3467676	10.0306671	11.9987961
A_30_P0103	chr16:46384974-46391331	lincRNA:chr1	chr16:463883		7.46013867	-2.7352396	0.15017956	-1.8716458	0.27326151	-0.8635938	0.54958184	6.26052755	8.99576715	7.12412131
A_55_P2081	NM_0011638	Msl3l2	Mus musculu	chr10:558365	8.37927983	2.0354587	4.09953052	0.68735515	1.61032864	1.34810355	2.54577259	9.50713391	7.47167521	8.15903036
A_52_P3915	NM_007552	Bmi1	Mus musculu	chr2:1860798	10.1601047	2.29012937	4.89099969	0.99865148	1.99813142	1.2914779	2.44778678	11.3539738	9.06384445	10.0624959
A_55_P2144	NM_008710	Nnt	Mus musculu	chr13:120124	10.1668142	1.06004305	2.08499373	-1.2322085	0.42566532	2.29225159	4.89819967	11.2842457	10.2242027	8.99199416
A_55_P2052	NM_199024	Nol4	Mus musculu	chr18:228519	11.690755	2.07980718	4.2275071	1.52166014	2.87121256	0.55814704	1.47237692	12.5700731	10.4902659	12.0119261
A_30_P0103	chr2:83540813-83541273_	lincRNA:chr2	chr2:8354093		6.68895356	-0.2223924	0.85714286	1.82986382	3.55503513	-2.0522562	0.24110672	5.93073734	6.15312976	7.98299357
A_52_P3815	NM_028055	Btbd17	Mus musculu	chr11:114652	7.49579059	-2.0334774	0.24426559	-1.5613537	0.338833	-0.4721237	0.72090261	6.66059021	8.69406764	7.13271392
A_51_P3041	NM_023386	Rtp4	Mus musculu	chr16:236136	8.06698694	-2.2388642	0.21185304	-1.3990067	0.37919013	-0.8398576	0.55869873	7.04074634	9.27961058	7.8806039
A_52_P5022	NM_172663	Epc2	Mus musculu	chr2:4940581	10.1667663	2.12429075	4.35988701	0.76229476	1.69618644	1.36199599	2.57040553	11.3288619	9.20457114	9.9668659
A_55_P2008	XM_0014767	Gm3450	PREDICTED: M	chr5:6443944	6.69633362	-0.2981418	0.81329923	2.3089558	4.95524297	-2.6070976	0.16412903	5.72792045	6.0260623	8.33501809
A_55_P2033	NM_0010855	Gm13298	Mus musculu	chr4:4184183	9.04599569	2.57019738	5.93890675	1.48036488	2.79019293	1.0898325	2.12849323	10.2660057	7.69580827	9.17617315
A_30_P0102	chr16:46384974-46391331	lincRNA:chr1	chr16:463882		8.55412761	-3.0352324	0.12198432	-1.6331822	0.32237636	-1.4020502	0.37839102	7.07503339	10.1102658	8.47708364
A_51_P1471	NM_008744	Ntn1	Mus musculu	chr11:680401	8.57429411	-2.5738373	0.16795687	-2.4101265	0.18813934	-0.1637108	0.89272594	7.6617781	10.2356154	7.82548885
A_52_P9012	NM_011156	Prep	Mus musculu	chr10:448786	10.1222427	1.67412569	3.19125898	-0.4839223	0.71503101	2.15804798	4.46310573	11.399634	9.72550828	9.24158599
A_51_P4446	NM_010701	Lect1	Mus musculu	chr14:800501	6.99911799	-2.6771789	0.15634675	-1.3316382	0.39731682	-1.3455407	0.39350649	5.65821148	8.33539035	7.00375213
A_52_P1506	XM_972673	LOC674050	PREDICTED: M	chr15:804829	7.30157966	-3.0502114	0.12072435	-1.9163557	0.26492287	-1.1338557	0.4556962	5.9068906	8.95710204	7.04074634
A_51_P1595	ENSMUST00000093902		ring finger pr	chr11:119348	8.30916072	-1.9695789	0.25532755	-2.2696539	0.20737964	0.30007495	1.23120837	7.7526594	9.72223831	7.45258445
A_55_P2064	NM_0010372	Defb36	Mus musculu	chr2:1524382	6.83519108	-0.2261543	0.85491071	2.06455032	4.18303571	-2.2907047	0.20437567	5.99623808	6.22239242	8.28694274
A_51_P2481	NM_133234	Bbc3	Mus musculu	chr7:1690340	10.9108679	-2.6658638	0.1575778	0.1292155	1.09369881	-2.7950793	0.14407788	9.09055354	11.7564173	11.8856328
A_55_P2065	NM_016740	S100a11	Mus musculu	chr3:9333002	9.33639127	-3.0971276	0.11686156	-2.2215582	0.21440966	-0.8755694	0.54503871	8.01215893	11.1092865	8.88772834

A_55_P20512	NR_003368	Pvt1	Mus musculu	chr15:62081(	8.24564575	-3.0998525	0.11664105	-1.4445384	0.36740968	-1.6553141	0.31746862	6.66059021	9.76044275	8.31590431
A_30_P01022	chr13:29106280-29120376	lincRNA:chr1	chr13:291203	7.64051005	2.13002981	4.37726524	0.80972973	1.75288303	1.32030008	2.49718045	8.79062001	6.66059021	7.47031993
A_55_P21049	NM_172420	Ppp1r1c	Mus musculu	chr2:7954834	7.23311106	2.3930404	5.25263158	-0.403274	0.75614035	2.79631445	6.94663573	8.96289601	6.56985561	6.16658156
A_55_P19751	NM_0011617	Galnt6	Mus musculu	chr15:100522	7.86059657	-0.7410817	0.5982906	1.95688976	3.88224122	-2.6979715	0.15410959	6.71424552	7.45532722	9.41221698
A_51_P39872	NM_010228	Flt1	Mus musculu	chr5:1483738	7.3664164	-2.2091659	0.2162593	-0.5708501	0.67321998	-1.6383158	0.32123125	6.08392248	8.29308841	7.72223831
A_52_P35437	XM_0014811	1190002F15R	PREDICTED: M	chr6:1349014	8.41928261	-2.031615	0.24458114	-3.1673917	0.11130639	1.13577678	2.19736842	8.12066989	10.1522848	6.98489311
A_55_P22569	AK082264	C230030N03R	Mus musculu	chr1:3478108	8.24623288	-3.7308063	0.07532088	-1.7779403	0.29159942	-1.952866	0.25830258	6.35167544	10.0824817	8.30454146
A_55_P20014	NM_0010099	Il19	Mus musculu	chr1:1328295	6.62952002	-0.6029964	0.65838509	1.49880586	2.82608696	-2.1018023	0.23296703	5.72792045	6.33091688	7.82972274
A_30_P01031	chr15:61870759-61938796	lincRNA:chr15	chr15:618708	7.42650053	-1.6180068	0.32578524	0.39567573	1.3155588	-2.0136826	0.2476402	6.2159374	7.83394423	8.22961997
A_30_P01023	chr12:109044453-1090456	lincRNA:chr12	chr12:109044	7.73883728	-2.018577	0.24680149	-0.7377597	0.59966983	-1.2808173	0.41156228	6.63903917	8.65761619	7.91985649
A_55_P19697	NM_207573	Olfr1380	Mus musculu	chr11:493774	8.16008427	-2.0933285	0.23433941	-1.0057626	0.49800683	-1.087566	0.4705546	7.09978612	9.19311463	8.18735207
A_52_P55954	NM_207298	Cercam	Mus musculu	chr2:2973777	7.46084163	-2.4490007	0.18313753	-1.9101346	0.26606773	-0.5388661	0.68831169	6.46488605	8.91388671	7.00375213
A_51_P17208	NM_008113	Arhgdig	Mus musculu	chr17:263362	8.31109375	-1.7096815	0.30572755	0.3916792	1.3119195	-2.1013607	0.23303835	7.04074634	8.75042785	9.14210706
A_30_P01019	chr17:21752466-21793451	lincRNA:chr17	chr17:217662	7.40634756	-0.5145732	0.7	1.89435896	3.71756757	-2.4089321	0.18829517	6.43184579	6.94641896	8.84077792
A_30_P01026	chr3:121627545-12163947	lincRNA:chr3:	chr3:1216390	7.39824566	2.0380606	4.10693069	0.97114514	1.96039604	1.06691546	2.09494949	8.43323768	6.39517708	7.36632221
A_30_P01032	chr8:122728333-12275213	lincRNA:chr8:	chr8:1227286	7.62492355	-2.4039769	0.18894302	-0.5809953	0.66850244	-1.8229816	0.28263625	6.2159374	8.61991427	8.03891899
A_51_P32035	NM_008171	Grin2b	Mus musculu	chr6:1356801	9.54261409	1.94965678	3.86282623	2.58067059	5.98217696	-0.6310138	0.64572249	9.98216174	8.03250496	10.6131756
A_55_P19739	NM_011404	Slc7a5	Mus musculu	chr8:1244051	10.5374741	-2.2273113	0.21355634	-0.4363074	0.73902373	-1.7910039	0.28897089	9.19803571	11.4253471	10.9890396
A_55_P21706	ENSMUST00000068297	RIKEN cDNA 9	chr1:8133325	7.28331176	2.34522471	5.08139535	1.01501952	2.02093023	1.3302052	2.51438435	8.50845506	6.16323035	7.17824987
A_55_P19857	NM_198092	Usp2	Mus musculu	chr9:4390364	7.21553388	-2.3884957	0.19098143	-0.8510616	0.55437666	-1.5374341	0.34449761	5.9068906	8.29538631	7.44432473
A_55_P21169	NM_0010379	Dbi	Mus musculu	chr1:1220099	12.2629881	-2.2095461	0.21620231	-2.2998856	0.20307921	0.09033944	1.06462064	11.5565859	13.766132	11.4662464
A_55_P19853	NM_031395	Sytl3	Mus musculu	chr17:694233	7.18333912	-1.2860782	0.41006424	0.98914679	1.98501071	-2.2752249	0.20658037	5.99623808	7.28231624	8.27146303
A_55_P19536	NM_144955	Nkx6-1	Mus musculu	chr5:1020883	8.76348105	-3.4938808	0.08876404	-2.6086607	0.16395131	-0.8852201	0.54140491	7.30378075	10.7976615	8.18900087
A_51_P31590	NM_011817	Gadd45g	Mus musculu	chr13:519437	11.0949133	-2.6290339	0.16165231	-0.4648877	0.72452746	-2.1641462	0.22311413	9.49718654	12.1262205	11.6613328
A_52_P54581	NM_0011113	Lrrfip1	Mus musculu	chr1:9301380	8.35844626	-2.2747661	0.20664609	-2.1761604	0.22126385	-0.0986057	0.93393517	7.56732234	9.84208839	7.66592804
A_55_P19823	NM_022565	Ndst4	Mus musculu	chr3:1254277	7.17535472	2.29460351	4.90619137	-0.187627	0.87804878	2.48223051	5.58760684	8.76763273	6.47302922	6.28540222
A_55_P21627	NM_173391	Tph2	Mus musculu	chr10:114516	8.90717002	-4.2619432	0.05212274	-0.9100779	0.53215637	-3.3518654	0.09794629	6.36923381	10.6311771	9.72109919
A_51_P38592	NM_138750	Prom2	Mus musculu	chr2:1273605	6.89496756	-0.1526883	0.89957265	1.98138432	3.94871795	-2.1340726	0.22781385	6.13271392	6.28540222	8.26678654
A_55_P21019	NM_146844	Olfr1107	Mus musculu	chr2:8691141	7.08009556	0.2569083	1.19491525	2.09033674	4.25847458	-1.8334284	0.28059701	6.55458885	6.29768055	8.38801729
A_51_P19423	NM_009573	Zic1	Mus musculu	chr9:9125548	11.7605847	3.97678071	15.744551	1.60312767	3.03801221	2.37365304	5.18251736	13.877396	9.90061527	11.5037429
A_66_P11101	NM_008091	Gata3	Mus musculu	chr2:9779610	10.0585587	-7.128424	0.00714711	-1.6551148	0.31751248	-5.4733092	0.0225097	5.857981	12.986405	11.3312902
A_52_P36928	NM_194269	Morn2	Mus musculu	chr17:806938	10.2382665	-1.969808	0.25528701	0.06504413	1.04611694	-2.0348521	0.24403296	8.90337978	10.8731877	10.9382319
A_55_P21044	NM_0011640	Pld1	Mus musculu	chr3:2803206	6.83253904	-2.6848344	0.1555193	-1.5967783	0.33061446	-1.0880562	0.47039474	5.57490884	8.25974326	6.66296501
A_52_P40831	ENSMUST00000124404	von Willebrar	chr1:3712033	6.66300131	0.04272087	1.03005464	2.15407105	4.45081967	-2.1113502	0.23143033	5.97345821	5.93073734	8.08480839
A_52_P38908	NM_0011903	Tmem132b	Mus musculu	chr5:1262692	11.9644117	-0.9238592	0.52709715	1.53618196	2.90025944	-2.4600412	0.18174138	10.8364449	11.7603041	13.2964861
A_66_P11980	NM_181848	Optn	Mus musculu	chr2:4954246	7.8323807	-2.197773	0.21797386	-1.2881452	0.40947712	-0.9096277	0.53232243	6.79658045	8.99435344	7.7062082
A_30_P01025	chr5:22890322-22939119_	lincRNA:chr5:	chr5:2289041	8.73062757	2.13643394	4.39673913	0.23965041	1.18070652	1.89678353	3.72382048	10.0750334	7.93859946	8.17824987
A_55_P21870	NM_011430	Sncg	Mus musculu	chr14:351835	8.48184847	-2.9820215	0.12656746	-1.4560586	0.36448754	-1.5259629	0.34724771	6.97918699	9.9612085	8.50514992
A_51_P24804	NM_008483	Lamb2	Mus musculu	chr9:1083921	8.9793244	-3.5743192	0.08395039	-1.9306539	0.26231026	-1.6436653	0.32004234	7.23999624	10.8143154	8.88366154
A_55_P20913	NM_008812	Padi2	Mus musculu	chr4:1405084	8.41120135	-3.0430854	0.12132212	-1.0295177	0.4898739	-2.0135678	0.24765991	6.72565028	9.76873571	8.73921805
A_55_P21010	NM_016769	Smad3	Mus musculu	chr9:6349463	9.93872446	-2.2517285	0.20997238	-1.3060777	0.4044189	-0.9456508	0.51919528	8.87293134	11.1246599	9.81858218
A_30_P01022	chr16:46384974-46391331	lincRNA:chr16	chr16:463895	8.33921686	-2.6885966	0.15511428	-1.6851161	0.31097789	-1.0034806	0.49879518	7.10852446	9.79712109	8.11200503
A_52_P55266	NM_008057	Fzd7	Mus musculu	chr1:5954319	7.86447881	-3.7132987	0.07624049	-2.2313028	0.21296632	-1.4819959	0.3579932	6.13271392	9.84601267	7.61470984
A_55_P19541	NM_011795	C1ql1	Mus musculu	chr11:102801	8.55826938	-3.1045517	0.11626174	-1.6013987	0.32955731	-1.503153	0.35278155	7.02236781	10.1269195	8.52552081

A_52_P11211	NM_145987	Tmem82	Mus musculu	chr4:1411703	7.38832215	-0.4143707	0.75034674	1.85265828	3.61165049	-2.2670289	0.2077573	6.49452228	6.90889295	8.76155123
A_55_P20452	NM_007866	Dll3	Mus musculu	chr7:2907912	11.1664859	-1.9747256	0.2544183	-2.2839902	0.20532907	0.30926457	1.23907591	10.6113322	12.5860579	10.3020677
A_55_P20674	NM_028841	Tspan17	Mus musculu	chr13:548980	11.9739568	-3.6830802	0.07785426	-0.1841233	0.88018378	-3.4989569	0.08845228	9.57994443	13.2630246	13.0789013
A_55_P21076	XM_0014758	Gm7321	PREDICTED: M	chr14:542091	7.8337951	-2.0018059	0.24968727	-2.6360269	0.16087065	0.63422108	1.55209953	7.3779335	9.37973936	6.74371243
A_55_P19882	XR_034120	LOC638183	PREDICTED: M	chr6:8179101	9.23573319	-1.1991082	0.43554443	-2.4419121	0.18403957	1.24280388	2.36658031	9.25029842	10.4494066	8.00749454
A_30_P01019	chr5:144517700-14452607	lincRNA:chr5:	chr5:1445255	6.81927037	1.61838885	3.07031963	-0.6958357	0.6173516	2.31422457	4.97337278	8.13014151	6.51175265	5.81591694
A_55_P21373	NM_153143	Kctd11	Mus musculu	chr11:696918	8.36228264	-2.86055	0.13768564	-2.2723491	0.20699257	-0.5882009	0.6651719	7.21269903	10.073249	7.8008999
A_30_P01030	chr15:92174226-92201201	lincRNA:chr15	chr15:921965	7.91437334	-2.4449414	0.18365353	-0.8105294	0.57017258	-1.634412	0.32210166	6.55458885	8.9995303	8.18900087
A_66_P10879	NM_175651	Cnpy1	Mus musculu	chr5:2853207	6.85076224	-2.1638367	0.223162	-1.2866934	0.40988939	-0.8771433	0.54444444	5.83710227	8.00093895	6.71424552
A_55_P19558	NM_178906	AI593442	Mus musculu	chr9:5248408	8.50053333	-1.3003732	0.40602116	0.76705308	1.70179007	-2.0674263	0.23858475	7.3779335	8.6783067	9.44535978
A_55_P21878	AK015708	4930505N22I	Mus musculu	chr19:460878	8.13560223	0.22846126	1.1715847	2.42034913	5.35300546	-2.1918879	0.21886484	7.48112669	7.25266543	9.67301456
A_51_P23980	ENSMUST00000108686		myosin, heav	chr11:671083	6.11667406	0.23109912	1.17372881	2.22588141	4.6779661	-1.9947823	0.2509058	5.52877967	5.29768055	7.52356196
A_55_P21153	NM_174993	Fmr1nb	Mus musculu	chrX:6605680	8.2819779	-2.6426197	0.16013719	-0.4210764	0.74686717	-2.2215433	0.21441187	6.66059021	9.30320995	8.88213353
A_52_P86693	NM_026790	Ifi27l1	Mus musculu	chr12:104678	10.0210005	-3.906315	0.06669327	-0.5716197	0.67286097	-3.3346954	0.09911894	7.60733031	11.5136454	10.9420257
A_55_P21152	NM_199195	Bckdhb	Mus musculu	chr9:8401778	10.151203	-0.0225723	0.98447584	-2.224155	0.21402407	2.20158274	4.599837	10.8775398	10.9001121	8.67595703
A_51_P16108	NM_008908	Ppic	Mus musculu	chr18:535661	8.30743692	-3.2594232	0.10442774	-1.7047852	0.30676692	-1.554638	0.34041394	6.70274988	9.96217303	8.25738784
A_55_P23197	AK019576	4930414F18R	Mus musculu	chr16:126292	8.21665146	0.26279537	1.19980119	2.21880273	4.65506958	-1.9560074	0.25774076	7.65224746	7.38945209	9.60825482
A_52_P24463	NM_0010803	Fam65b	Mus musculu	chr13:247972	9.101582	2.16968357	4.49924699	1.76483163	3.39834337	0.40485193	1.32395302	9.9597605	7.79007693	9.55490856
A_30_P01032	chr5:26333765-26338143_	lincRNA:chr5:	chr5:2633792	6.77996077	1.47232442	2.77468582	-0.7421552	0.5978456	2.21447957	4.64114114	8.00889544	6.53657102	5.79441587
A_51_P41732	NM_177832	Zfp236	Mus musculu	chr18:827650	8.48642431	2.3791536	5.20231445	-0.0385053	0.97366321	2.41765887	5.34303279	10.0853618	7.7062082	7.66770293
A_51_P38215	NM_011171	Procr	Mus musculu	chr2:1555809	6.82310769	-2.1379573	0.22720126	-0.576481	0.67059748	-1.5614762	0.33880422	5.58996318	7.72792045	7.15143943
A_30_P01025	chr2:157361470-15737981	lincRNA:chr2:	chr2:1573716	10.1723552	-1.1779802	0.44196984	0.99119207	1.98782681	-2.1691722	0.2223382	9.05663772	10.2346179	11.2258099
A_51_P49166	NM_024440	Derl3	Mus musculu	chr10:753585	7.11036776	-1.0126951	0.49561952	1.17253077	2.25406758	-2.1852258	0.21987785	6.04439412	7.05708919	8.22961997
A_55_P20013	NM_146855	Olfr985	Mus musculu	chr9:3993466	6.90584885	0.14268842	1.1039604	2.35511119	5.11633663	-2.2124228	0.21577165	6.2159374	6.07324898	8.42836017
A_30_P01027	chr5:96591892-96606467_	lincRNA:chr5:	chr5:9660347	9.18530389	-3.0742182	0.11873209	-1.9562323	0.25770057	-1.1179858	0.46073662	7.78790256	10.8621207	8.90588838
A_30_P01029	chr1:93744621-93825321_	lincRNA:chr1:	chr1:9375569	7.70899502	-0.3069778	0.80833333	1.74750675	3.35777778	-2.0544845	0.24073461	6.92184094	7.22881869	8.97632544
A_55_P20995	NM_031159	Apobec1	Mus musculu	chr6:1225279	7.20554848	-1.8225294	0.28272484	-2.2053643	0.21682992	0.38283482	1.30390144	6.72565028	8.54817971	6.34281546
A_55_P21125	NM_028231	Kcnmb2	Mus musculu	chr3:3209862	7.756297	2.07233235	4.20566038	1.80190049	3.48679245	0.27043186	1.20616883	8.5372184	6.46488605	8.26678654
A_55_P21345	NM_178776	BC049715	Mus musculu	chr6:1367889	8.01943388	-2.283793	0.20535714	0.70913306	1.63482143	-2.992926	0.12561442	6.26052755	8.54432052	9.25345358
A_55_P20718	NM_008598	Mgmt	Mus musculu	chr7:1443197	7.93393101	-3.3947897	0.09507503	-1.8808279	0.27152785	-1.5139617	0.35014837	6.29768055	9.69247021	7.81164228
A_55_P19761	NM_007900	Ect2	Mus musculu	chr3:2699633	7.65170103	0.02865876	1.02006336	-1.98033	0.25343189	2.00898878	4.025	8.33091688	8.30225811	6.32192809
A_55_P19851	XR_030646	Gm8284	PREDICTED: M	chr18:907007	10.8363883	-1.3786739	0.38457213	-2.87946	0.13589272	1.50078607	2.82996865	10.8770924	12.2557663	9.3763063
A_55_P20321	NM_139298	Wnt9a	Mus musculu	chr11:591469	7.47297396	-2.9763333	0.12706747	-2.535447	0.17248622	-0.4408863	0.73668189	6.33390074	9.31023407	6.77478706
A_55_P19969	NM_023223	Cdc20	Mus musculu	chr4:1181055	9.22509897	-0.9291954	0.52515115	-2.6816351	0.15586457	1.75243969	3.36927851	9.49951374	10.4287091	7.74707405
A_55_P21306	NM_178740	Slitrk4	Mus musculu	chrX:6152331	8.28827417	2.76096202	6.77848101	0.98162147	1.97468354	1.77934055	3.43269231	9.80170836	7.04074634	8.02236781
A_55_P21671	NM_145611	Kank2	Mus musculu	chr9:2157132	7.66861511	-2.4794354	0.17931457	-1.7824086	0.29069767	-0.6970268	0.61684211	6.60979435	9.08922977	7.3068212
A_55_P20892	XM_0014765	LOC1000466	PREDICTED: M	chr15:994252	7.38679905	-2.3611072	0.1946417	-2.5149724	0.17495158	0.15386515	1.11254613	6.65105169	9.01215893	6.49718654
A_52_P53222	NM_007901	S1pr1	Mus musculu	chr3:1154140	7.75667251	-2.1553805	0.22447389	-2.5505184	0.17069369	0.39513794	1.31506849	7.169925	9.32530546	6.77478706
A_51_P33899	NM_008922	Prim2	Mus musculu	chr1:3351079	7.52464342	2.14024512	4.40836941	-0.121562	0.91919192	2.2618071	4.79591837	8.99199416	6.85174904	6.73018706
A_55_P20904	NM_010063	Dync1i1	Mus musculu	chr6:5977935	11.3675469	2.26638308	4.81115435	0.85359961	1.80700389	1.41278347	2.66250359	12.5939357	10.3275526	11.1811523
A_30_P01030	chr1:94655789-94657057_	lincRNA:chr1:	chr1:9465590	7.79408503	0.00921853	1.00641026	2.30593312	4.94487179	-2.2967146	0.20352606	7.03158634	7.02236781	9.32830093
A_30_P01025	chr9:75316422-75332567_	lincRNA:chr9:	chr9:7533078	8.37319368	-0.7843703	0.58060531	1.67617707	3.19579988	-2.4605474	0.18167762	7.29155445	8.07592477	9.75210184
A_66_P11257	NM_020583	Isg20	Mus musculu	chr7:8606513	8.14691826	-2.609637	0.1638404	-1.1028803	0.46558603	-1.5067567	0.35190145	6.77478706	9.38442402	8.28154371
A_30_P01026	chr17:7838192-7853103_F	lincRNA:chr17	chr17:785304	6.63001666	0.15298086	1.11186441	2.87824319	7.35254237	-2.7252623	0.15122176	5.7725895	5.61960864	8.49785184

A_55_P2047{	NM_175692	Snhg11	Mus musculu	chr2:1582065	9.46830866	-2.5853553	0.16662129	0.21693927	1.16226518	-2.8022946	0.1433591	7.67242534	10.2577807	10.4747199
A_52_P2384(	NM_0010808	Gipr	Mus musculu	chr7:1974254	9.3006568	-1.6718915	0.3138416	1.37442603	2.59264742	-3.0463175	0.12105063	7.72792045	9.39981196	10.774238
A_55_P2083{	NM_011063	Pea15a	Mus musculu	chr1:1741276	11.6194792	-2.1319307	0.22815234	-1.0754208	0.47453262	-1.0565098	0.48079379	10.5566657	12.6885964	11.6131756
A_55_P2014:	NM_146566	Olfr830	Mus musculu	chr9:1868066	6.61303739	-0.1269121	0.91578947	2.01134497	4.03157895	-2.1382571	0.22715405	5.857981	5.98489311	7.99623808
A_55_P1987:	NM_029536	Gpr165	Mus musculu	chrX:9391462	8.04181179	2.11687933	4.33754682	-0.4186442	0.74812734	2.53552357	5.79787234	9.59261276	7.47573343	7.05708919
A_55_P2176{	XM_0014727	Gm2185	PREDICTED: M	chr12:504831	7.385149	2.04041427	4.11363636	1.5258411	2.87954545	0.51457317	1.42857143	8.23681148	6.19639721	7.72223831
A_55_P2328⁄	AK044281	A930005G22I	Mus musculu	chr5:1158706	9.03872359	-2.5687094	0.16855491	-1.7956856	0.28803468	-0.7730239	0.58518964	7.9248125	10.4935219	8.69783636
A_55_P2097!	XM_0014810	9030622O22I	PREDICTED: M	chr2:1477986	8.94386898	-5.3585546	0.0243733	-2.2836441	0.20537834	-3.0749105	0.11867512	6.13271392	11.4912686	9.20762447
A_55_P2055:	XR_031611	Gm7556	PREDICTED: M	chr6:3979369	10.8195355	-2.4445411	0.1837045	-0.6882496	0.62060639	-1.7562916	0.29600807	9.41925797	11.8637991	11.1755496
A_55_P2106:	NM_008102	Gch1	Mus musculu	chr14:477754	7.85326731	-2.9259994	0.13157895	-2.5030937	0.17639803	-0.4229057	0.74592075	6.73696559	9.66296501	7.15987134
A_55_P2059:	NR_028497	Gm13315	Mus musculu	chr2:1464323	9.37769209	-2.9412449	0.13019583	-0.7525223	0.59356491	-2.1887226	0.21934556	7.66770293	10.6089478	9.85642553
A_66_P1194!	NM_175189	Hepacam	Mus musculu	chr9:3719318	7.15171752	-2.0935347	0.23430592	-2.1265749	0.22900088	0.03304026	1.02316602	6.46488605	8.55842071	6.43184579
A_52_P6353:	NM_010574	Irx2	Mus musculu	chr13:727687	7.56363765	-2.0440139	0.24248814	-0.1786975	0.88350026	-1.8653164	0.27446301	6.26052755	8.30454146	8.12584393
A_66_P1306⁄	XM_0014803	LOC1000484:	PREDICTED: M	chr9:7473740	8.76280022	-3.0373751	0.12180328	-0.6431173	0.64032787	-2.3942578	0.19022017	6.9522559	9.98963103	9.34651373
A_55_P1985:	NM_053273	Ttyh2	Mus musculu	chr11:114582	7.95791291	-2.7524017	0.14840363	-2.5456954	0.17126527	-0.2067063	0.86651323	6.97154355	9.7239453	7.17824987
A_52_P2099⁄	ENSMUST00000052354		RIKEN cDNA (	chr13:838805	11.4919958	2.04740462	4.1336167	0.69275719	1.61636968	1.35464742	2.5573461	12.6260132	10.5786086	11.2713658
A_30_P0103:	chr5:96591892-96606467_		lincRNA:chr5:	chr5:9660327	9.6880509	-3.0078808	0.12431904	-1.5897061	0.33223914	-1.4181748	0.37418541	8.21269903	11.2205799	9.63087381
A_52_P1756:	NM_0010774	A4gnt	Mus musculu	chr9:9952109	7.32019492	-0.1124747	0.925	2.44009794	5.42678571	-2.5525727	0.17045081	6.43184579	6.54432052	8.98441846
A_51_P3248:	NM_010664	Krt18	Mus musculu	chr15:101862	9.13076331	-2.4324626	0.18524897	-0.5356371	0.68985399	-1.8968255	0.26853359	7.68766728	10.1201299	9.5844928
A_52_P8434:	XM_0014738	4931419H13I	PREDICTED: M	chr3:5488768	7.10397796	0.1459667	1.10647182	2.20920914	4.62421712	-2.0632424	0.23927765	6.46488605	6.31891935	8.52812848
A_55_P1988:	XM_0014721	Gm6127	PREDICTED: M	chr6:3165230	6.70760863	0.1621055	1.11891892	2.12146351	4.35135135	-1.959358	0.25714286	6.10852446	5.94641896	8.06788247
A_55_P1986:	NM_178598	Tagln2	Mus musculu	chr1:1744374	8.09379465	-3.4145598	0.09378105	-1.4407774	0.36836877	-1.9737825	0.25458468	6.29768055	9.71224038	8.27146303
A_55_P2091⁄	NM_009760	Bnip3	Mus musculu	chr7:1460826	11.1960851	-2.1233713	0.22950997	-0.8066716	0.57169931	-1.3166997	0.40145225	10.0493948	12.1727661	11.3660945
A_66_P1191!	ENSMUST000	Dusp3	dual specificit	chr11:101832	9.07545261	-2.9682812	0.12777866	-1.6776911	0.3125825	-1.2905901	0.40878378	7.65582883	10.62411	8.94641896
A_51_P3302:	NM_024184	Asf1b	Mus musculu	chr8:8649384	7.81654991	-1.4457171	0.36710963	-2.016389	0.24717608	0.57067184	1.48521505	7.52486815	8.97058527	6.95419631
A_51_P4338:	NM_009415	Tpi1	Mus musculu	chr6:1247610	11.9080439	-2.6205277	0.16260824	-0.2604096	0.83485083	-2.3601181	0.1947752	10.2478286	12.8683564	12.6079467
A_55_P2097{	NM_172862	Frem2	Mus musculu	chr3:5331806	8.37756901	-2.3870955	0.19116688	-2.4745583	0.17992177	0.08746284	1.0625	7.6110248	9.99812027	7.52356196
A_55_P1975{	XR_002032	Gm8659	PREDICTED: M	chrX:1545890	13.0602148	-2.0787471	0.2367199	-0.461656	0.72615224	-1.617091	0.32599211	11.8282687	13.9070158	13.4453598
A_52_P1620!	NM_0010041	Ckap2	Mus musculu	chr8:2327944	9.18264462	-2.2745307	0.2066798	-2.5620014	0.16934046	0.28747066	1.22049861	8.52029128	10.794822	8.23282062
A_52_P1879⁄	NM_008494	Lfng	Mus musculu	chr5:1410912	7.78599163	-3.0965939	0.11690481	-2.9779494	0.12692522	-0.1186445	0.92105263	6.71424552	9.81083937	6.83289001
A_52_P5204(	NM_197959	Kif18b	Mus musculu	chr11:102767	7.17450673	-1.7089759	0.30587711	-2.412043	0.18788958	0.7030671	1.62796209	6.83920379	8.54817971	6.13613669
A_55_P1997⁄	NM_0011774	Gm13698	Mus musculu	chr2:8379886	6.13690872	2.07815081	4.22265625	0.08746284	1.0625	1.99068797	3.97426471	7.49318831	5.4150375	5.50250034
A_52_P5905:	NM_007992	Fbln2	Mus musculu	chr6:9122211	8.07600974	-3.8937588	0.06727625	-3.541259	0.08589637	-0.3524998	0.78322581	6.66059021	10.554349	7.01309
A_30_P0102{	H19		lincRNA:chr7:	chr7:1497621	11.9358472	-5.4977459	0.02213164	-1.6787034	0.31236325	-3.8190425	0.07085225	8.8302511	14.327997	12.6492936
A_55_P2150!	NM_008718	Npas1	Mus musculu	chr7:1704673	8.94600345	-0.3394687	0.79033233	2.85452003	7.2326284	-3.1939887	0.10927318	7.76818432	8.107653	10.962173
A_51_P2234(	NM_025836	Plin3	Mus musculu	chr17:564184	9.45831188	-3.1940204	0.10927078	-1.7875428	0.28966499	-1.4064777	0.37723157	7.9248125	11.118833	9.3312902
A_66_P1115(	NM_007631	Ccnd1	Mus musculu	chr7:1521162	10.8901269	-2.5035525	0.17634194	-2.6011014	0.16481261	0.09754895	1.06995413	10.0881257	12.5916782	9.99057675
A_55_P1959!	XR_034879	Gm14026	PREDICTED: M	chr2:1289968	10.1168996	2.28813785	4.88425271	-0.3341381	0.7932579	2.62227596	6.15720654	11.7537043	9.4655664	9.13142829
A_52_P2322!	NM_016697	Gpc3	Mus musculu	chrX:4962579	9.24179407	-2.1198512	0.23007064	-0.359422	0.77947683	-1.7604293	0.29516033	7.94836723	10.0682185	9.70879651
A_55_P2241:	NM_0010256	Otud7b	Mus musculu	chr3:9596283	7.9860348	0.28676492	1.21990172	2.41957202	5.35012285	-2.1328071	0.22801378	7.37068741	7.08392248	9.5034945
A_55_P2102:	NM_008217	Has3	Mus musculu	chr8:1094053	9.01130521	2.09865226	4.28309078	1.20558413	2.30630631	0.89306812	1.85712139	10.0085453	7.90989308	9.11547722
A_55_P2030⁄	NM_008155	Gpi1	Mus musculu	chr7:3498723	12.6586971	-2.7429136	0.14938285	-0.4998623	0.70717428	-2.2430513	0.21123909	10.9967089	13.7396224	13.2397601
A_52_P3354(	NM_0010812	Dscaml1	Mus musculu	chr9:4525597	11.1689154	1.82647708	3.54669944	2.00795641	4.02212079	-0.1814793	0.88179834	11.717248	9.89077093	11.8987273
A_51_P4388(	NM_0010099	Txnip	Mus musculu	chr3:9636514	8.75166504	-1.3167708	0.40143247	0.77364523	1.7095839	-2.090416	0.23481297	7.6159361	8.93270689	9.70635212

A_55_P2243	AK035826	LOC552901	Mus musculu	chr6:141250	9.04692658	2.87504805	7.33627667	0.79562996	1.73583517	2.07941809	4.2263671	10.6984153	7.82336724	8.6189972
A_51_P3088	NM_153529	Nrn1	Mus musculu	chr13:36818	8.10834184	-2.1821673	0.22034449	-0.4998459	0.70718232	-1.6823214	0.31158088	6.82017896	9.00234623	8.50250034
A_51_P4156	NM_025278	Gng12	Mus musculu	chr6:669677	7.85687124	-2.8477245	0.13891512	-2.4820099	0.17899486	-0.3657145	0.77608441	6.78572491	9.63344937	7.15143943
A_55_P2231	NM_008498	Lhx1	Mus musculu	chr11:84335	11.2417528	-2.9037717	0.13362189	-0.5598477	0.67837378	-2.3439241	0.19697384	9.49252086	12.3962926	11.8364449
A_55_P2038	NM_007791	Csrp1	Mus musculu	chr1:137648	8.05017412	-2.8664802	0.13712085	-2.2547508	0.20953298	-0.6117294	0.65441176	6.89077093	9.7572511	7.50250034
A_51_P2933	NM_009427	Tob1	Mus musculu	chr11:94075	9.6680537	-1.7029342	0.30716075	-2.0349387	0.24401831	0.33200446	1.25876106	9.21107711	10.9140113	8.87907265
A_66_P1375	NM_0010855	Tmem90b	Mus musculu	chr2:149725	8.47244864	2.13840361	4.402746	1.71936141	3.29290618	0.41904221	1.33703961	9.32493058	7.18652697	8.90588838
A_66_P1024	AK048565	Casz1	Mus musculu	chr4:148213	7.36909469	1.06178469	2.08751229	-1.169925	0.44444444	2.23170969	4.69690265	8.46692616	7.40514146	6.23521646
A_55_P2108	NM_144888	Mavs	Mus musculu	chr2:131073	7.1601027	-2.148738	0.2255098	-2.4809344	0.17912835	0.33219643	1.25892857	6.55458885	8.70332684	6.22239242
A_55_P2150	NM_010063	Dync1i1	Mus musculu	chr6:597797	10.3902675	2.31883672	4.98929758	0.64026673	1.5586173	1.67856999	3.20110497	11.7227364	9.40389967	10.0441664
A_55_P2286	AK016664	4933405E24F	Mus musculu	chr11:53991	13.5210891	2.77580262	6.84856922	2.81216067	7.02335654	-0.0363581	0.97511342	14.4342373	11.6584347	14.4705953
A_55_P2095	ENSMUST00000121245		poliovirus rec	chr16:463951	9.2711458	-2.3170668	0.20067505	-1.8801367	0.27165797	-0.4369301	0.73870482	8.35314683	10.6702136	8.79007693
A_55_P1972	NM_009185	Stil	Mus musculu	chr4:114715	7.83278255	-1.5740467	0.33586498	-2.5596197	0.16962025	0.98557293	1.9800995	7.63662462	9.21067134	6.65105169
A_51_P5018	NM_010451	Hoxa2	Mus musculu	chr6:521126	7.82685154	-3.6570567	0.07927134	-1.9667412	0.25583025	-1.6903155	0.30985915	6.04439412	9.70145087	7.73470962
A_55_P2038	NM_019392	Tyro3	Mus musculu	chr2:119643	10.3276442	-2.0118403	0.24795662	-1.8269869	0.28185266	-0.1848534	0.87973844	9.5954129	11.6072532	9.78026635
A_55_P2046	NM_008235	Hes1	Mus musculu	chr16:30067	7.46967956	-2.3081223	0.20192308	-2.3499425	0.19615385	0.04182018	1.02941176	6.71424552	9.02236781	6.67242534
A_55_P2075	ENSMUST00000103508		Mus musculu	chr12:11600	6.6710833	-0.1631652	0.89306358	2.62741791	6.17919075	-2.7905831	0.1445276	5.68650053	5.84966573	8.47708364
A_55_P2183	NM_177298	Pisd	Mus musculu	chr5:330794	11.1844126	-2.3539886	0.1956045	0.40895193	1.32772092	-2.7629405	0.1473235	9.47876962	11.8327582	12.2417101
A_30_P0101	chr8:67542714-67561694_		lincRNA:chr8	chr8:675526	9.13518061	-4.9765621	0.03176183	-3.1586765	0.11198082	-1.8178856	0.28363636	6.87036472	11.8469268	8.68825031
A_55_P2328	AK142074	AI606473	Mus musculu	chr3:153997	10.0232994	4.54416454	23.3308108	3.72069258	13.1837838	0.82347196	1.7696597	11.8125116	7.26834705	10.9890396
A_51_P2500	NM_010137	Epas1	Mus musculu	chr17:87232	10.2107735	-5.5634366	0.02114651	-0.4763233	0.71880716	-5.0871133	0.02941889	6.66059021	12.2240268	11.7477035
A_55_P2180	XM_0014788	1810020O05I	PREDICTED: M	chr6:876258	7.18297101	-2.1865557	0.21967526	-1.605781	0.32855778	-0.5807747	0.66860465	6.26052755	8.44708323	6.84130225
A_30_P0101	chr1:94077221-94091396_		lincRNA:chr1	chr1:940910	6.67131472	0.05722487	1.04046243	2.40772212	5.30635838	-2.3504972	0.19607843	5.9068906	5.84966573	8.25738784
A_55_P2175	NM_019946	Mgst1	Mus musculu	chr6:138104	7.43654129	-3.2256676	0.1068999	-2.7309029	0.15063168	-0.4947647	0.70967742	6.19639721	9.42206477	6.6911619
A_55_P2104	NM_013618	Olfr66	Mus musculu	chr7:111029	7.13445073	0.07662128	1.05454545	2.73753928	6.66931818	-2.660918	0.15811893	6.27301849	6.19639721	8.93393649
A_30_P0102	chr17:86560289-86565864		lincRNA:chr1	chr17:86565	7.13906803	-2.2825794	0.20552995	-1.7957669	0.28801843	-0.4868125	0.7136	6.2159374	8.49851683	6.70274988
A_52_P2505	NM_019682	Dynll1	Mus musculu	chr5:115747	9.25808439	2.50693588	5.68411552	0.92609079	1.90012034	1.58084509	2.99145028	10.620678	8.11374217	9.03983296
A_55_P1967	NM_0010332	Mll2	Mus musculu	chr15:98663	9.34293775	-0.4891245	0.71245734	2.68855466	6.44667235	-3.1776791	0.11051552	8.12066989	8.60979435	11.298349
A_55_P2146	NM_010725	Lmx1b	Mus musculu	chr2:334203	8.42657847	-2.0778006	0.23687526	-1.5396323	0.34397312	-0.5381683	0.68864469	7.55458885	9.6323894	8.09275714
A_30_P0102	chr6:90915489-90940052_		lincRNA:chr6	chr6:909381	11.1127163	-2.5814532	0.16707257	-2.8309286	0.14054182	0.24947535	1.18877473	10.3353904	12.9168436	10.085915
A_55_P1973	NM_172539	Astl	Mus musculu	chr2:127183	7.4721478	1.05082426	2.07171315	2.20587598	4.61354582	-1.1550517	0.44905009	7.43740531	6.38658105	8.59245704
A_55_P1953	NM_021477	A2bp1	Mus musculu	chr16:74115	10.0335216	-2.4244929	0.18627515	-1.4116059	0.37589303	-1.0128869	0.49555362	8.88772834	11.3122212	9.90061527
A_30_P0102	chr6:126766225-12677130		lincRNA:chr6	chr6:126769	7.27152064	2.16041979	4.47044917	1.23548808	2.35460993	0.92493172	1.89859438	8.29997115	6.13955135	7.37503943
A_66_P1118	XM_0014770	Gm3336	PREDICTED: M	chr8:732460	7.94951016	0.04277402	1.03009259	2.29598146	4.91087963	-2.2532074	0.20975725	7.21269903	7.169925	9.46590646
A_52_P2980	NM_008102	Gch1	Mus musculu	chr14:47773	7.23074679	-2.7592059	0.14770536	-1.8148857	0.28422676	-0.9443202	0.51967436	5.99623808	8.75544399	6.94055831
A_30_P0103	chr3:121997064-12203121		lincRNA:chr3	chr3:121997	8.42415312	0.04253404	1.02992126	2.05297446	4.1496063	-2.0104404	0.24819734	7.76818432	7.72565028	9.77862475
A_30_P0102	chr5:96591892-96606467_		lincRNA:chr5	chr5:966033	10.0013848	-3.4786478	0.08970625	-1.6802816	0.31202172	-1.7983661	0.2875	8.2423802	11.721028	10.0407463
A_55_P2412	AK043919	A830052D11	Mus musculu	chr18:32518	7.5762389	-1.8917573	0.26947862	0.16361946	1.12009373	-2.0553768	0.24058577	6.26052755	8.15228484	8.31590431
A_55_P2099	NM_145998	Hmx2	Mus musculu	chr7:138700	8.65577359	-2.292502	0.20412121	-0.015825	0.98909091	-2.276677	0.20637255	7.13271392	9.4252159	9.40939094
A_55_P2023	XR_030502	LOC630896	PREDICTED: M	chr14:09582	8.63867497	-2.1799691	0.22068048	-1.2324825	0.42558449	-0.9474866	0.51853506	7.59618976	9.77615883	8.54367631
A_55_P1977	NM_023118	Dab2	Mus musculu	chr15:639061	8.32862999	-2.8885787	0.1350365	-0.0868048	0.94160584	-2.8017739	0.14341085	6.43184579	9.3204245	9.23361968
A_55_P1981	NM_0011778	Rreb1	Mus musculu	chr13:38043	8.73337878	-2.1898088	0.21918047	-1.3119006	0.40278989	-0.8779082	0.54415584	7.71080643	9.90061527	8.58871464
A_52_P1124	NR_015388	Dlx6os1	Mus musculu	chr6:677145	9.92528565	2.98444075	7.91418476	1.69026421	3.227158	1.29417653	2.45236978	11.3514914	8.36705066	10.0573149
A_55_P1958	NM_016707	Bcl11a	Mus musculu	chr11:24073	11.1546031	2.61752549	6.13696557	1.49728926	2.82311767	1.12023623	2.17382564	12.4005237	9.78299821	11.2802875

A_30_P0102(	chr16:59557120-59602070	lincRNA:chr1(		chr16:59557	7.75181481	-2.1556976	0.22442455	-1.6670449	0.3148977	-0.4886527	0.71269036	6.87036472	9.0260623	7.35901741
A_55_P2000;	NM_0010425	Cd63	Mus musculu	chr10:128349	11.2343464	-4.3132234	0.05030259	-1.3935094	0.38063776	-2.919714	0.13215345	8.82336724	13.1365907	11.7430812
A_66_P12566	NM_001114(	Nde1	Mus musculu	chr16:141695	8.61485414	-1.9756624	0.25425316	-2.3611875	0.19463087	0.38552514	1.3063352	8.08480839	10.0604708	7.69928325
A_30_P0103:	chr2:115900355-11590090	lincRNA:chr2:		chr2:1159007	7.69441655	-2.332235	0.19857625	-0.9758786	0.50843012	-1.3563565	0.39056743	6.46488605	8.79712109	7.8212425
A_52_P4693(	NM_013627	Pax6	Mus musculu	chr2:105524(	7.0641462	-2.2545854	0.20955699	-1.714874	0.30462917	-0.5397114	0.6879085	6.13271392	8.38729935	6.67242534
A_51_P2584!	NM_011067	Per3	Mus musculu	chr4:1503793	8.53528059	-2.6927007	0.15467364	-2.1905194	0.21907254	-0.5021813	0.70603849	7.47031993	10.1630206	7.9725012
A_55_P2073;	XM_0010031	LOC676974	PREDICTED: I	chr7:3500028	11.1704684	-3.1926621	0.10937371	-0.9870709	0.50450103	-2.2055912	0.21679581	9.37105058	12.5637127	11.5766418
A_52_P3542!	NM_007459	Ap2a2	Mus musculu	chr7:1488182	10.7730269	0.09760867	1.06999842	2.08351056	4.23837301	-1.9859019	0.25245499	10.1435958	10.0459871	12.1294977
A_52_P3534:	NM_010299	Gm2a	Mus musculu	chr11:549238	9.09548771	-2.4372765	0.18463188	-1.1182828	0.46064179	-1.3189937	0.40081443	7.84339767	10.2806741	9.16239133
A_30_P0103:	chr5:35893909-35899497_	lincRNA:chr5:		chr5:3589883	7.7764237	1.0417581	2.05873494	-1.0827178	0.47213855	2.12447589	4.36044657	8.83183503	7.79007693	6.70735913
A_51_P3757!	NM_010462	Hoxc10	Mus musculu	chr15:102801	7.34652046	-0.0193653	0.98666667	2.70529337	6.52190476	-2.7246587	0.15128505	6.43184579	6.45121111	9.15650449
A_51_P1606;	NM_021487	Kcne1l	Mus musculu	chrX:1387394	8.40872101	0.42929881	1.34657895	-3.1235994	0.11473684	3.55289819	11.7362385	9.73612002	9.3068212	6.18322182
A_30_P0103:	chr5:144517700-14452607	lincRNA:chr5:		chr5:1445256	8.987679	1.77530098	3.42309417	-0.4258605	0.74439462	2.20116145	4.59849398	10.3131665	8.53786549	8.11200503
A_55_P2074(	NM_007755	Cpeb1	Mus musculu	chr7:8849239	9.51187328	-2.7081838	0.15302256	-0.0645091	0.95627068	-2.6436747	0.16002013	7.72792045	10.4361042	10.3715952
A_51_P4802(	NM_010054	Dlx2	Mus musculu	chr2:7138161	11.5630643	2.72853976	6.62784452	1.34999737	2.54911661	1.37854239	2.60005545	12.9320917	10.2035519	11.5535493
A_30_P0102{	chr14:25924039-25924372	lincRNA:chr1·		chr14:25924(	7.18154588	0.40312418	1.32236842	2.39773092	5.26973684	-1.9946067	0.25093633	6.65105169	6.24792751	8.64565843
A_66_P1205!	NM_001081(	Grik3	Mus musculu	chr4:1253913	10.346711	2.42742257	5.37931535	1.11422106	2.16478097	1.3132015	2.48492361	11.5935856	9.16616308	10.2803841
A_30_P0102!	chr4:149383134-14945125	lincRNA:chr4:		chr4:1494335	9.96820861	-2.3756365	0.19269133	-0.3101672	0.80654828	-2.0654693	0.23890861	8.48784003	10.8634765	10.5533093
A_55_P1999!	NM_172910	Dlgap2	Mus musculu	chr8:1484759	8.95520346	2.27893959	4.85321101	1.28211783	2.43195719	0.99682176	1.99559887	10.0471239	7.76818432	9.05030216
A_30_P0102·	chr18:6144775-6202232_R	lincRNA:chr1!		chr18:620104	6.95243135	1.71699089	3.2875	-0.4926584	0.71071429	2.20964929	4.62562814	8.26131141	6.54432052	6.05166212
A_51_P4237(	NM_029007	Fam84a	Mus musculu	chr12:141549	10.462439	2.20950415	4.6251628	1.21315582	2.3184423	0.99634832	1.9949441	11.5310565	9.32155234	10.5347082
A_55_P1973;	NM_133978	Cmtm7	Mus musculu	chr9:1146661	7.65587187	-2.8216078	0.14145276	-1.9711373	0.25505188	-0.8504705	0.55460385	6.43184579	9.25345358	7.28231624
A_52_P6791(	NM_011597	Tjp2	Mus musculu	chr19:241697	9.46833069	-1.8836509	0.27099706	-2.055846	0.24050753	0.17219518	1.12677165	8.89784546	10.7814963	8.72565028
A_51_P3655:	NM_009258	Spink3	Mus musculu	chr18:438878	7.67418724	-2.0841178	0.2358403	-0.3568405	0.78087279	-1.7272773	0.3020214	6.40372219	8.48784003	8.13099949
A_51_P5090!	NM_019734	Asah1	Mus musculu	chr8:4242674	10.8953324	-2.0263773	0.24547069	-0.9094955	0.53237123	-1.1168818	0.46108933	9.8475794	11.8739567	10.9644612
A_55_P2172;	XM_0014756	Gm3105	PREDICTED: I	chr7:6686136	12.8395855	-0.901024	0.53550649	1.19653833	2.29189084	-2.0975624	0.2336527	11.8400567	12.7410807	13.937619
A_55_P2143!	NM_172523	Slc18a2	Mus musculu	chr19:593701	10.5419232	-3.7140003	0.07620343	-2.4040672	0.18893119	-1.3099331	0.40333957	8.86727874	12.5812791	10.1772119
A_51_P4515:	NM_028027	D10Ertd610e	Mus musculu	chr10:126619	11.3979725	-2.2014461	0.2174196	-0.3503465	0.78439566	-1.8510996	0.27718103	10.0471239	12.24857	11.8982235
A_51_P2825(	NM_007484	Rhoc	Mus musculu	chr3:1045973	10.4689604	-4.3738153	0.04823368	-2.3335001	0.1984022	-2.0403153	0.24311061	8.33091688	12.7047322	10.3712321
A_55_P2071{	XR_032130	Gm8681	PREDICTED: I	chr10:255528	9.01050069	-0.3906315	0.76279562	-2.2273236	0.21355454	1.83669202	3.57190083	9.49252086	9.88315239	7.65582883
A_55_P2118!	NM_007742	Col1a1	Mus musculu	chr11:948131	7.7278405	-1.7282286	0.30182232	0.33240618	1.25911162	-2.0606348	0.23971054	6.46488605	8.19311463	8.52552081
A_55_P2163{	XM_0014772	Gm3088	PREDICTED: I	chr2:2970059	7.71141731	-3.9760283	0.06354717	-3.2159465	0.10762264	-0.7600818	0.59046283	6.13271392	10.1087422	6.89279577
A_55_P2067!	NM_030696	Slc16a3	Mus musculu	chr11:12082(	9.82601889	-3.8196589	0.07082198	-1.5249689	0.34748705	-2.29469	0.20381187	7.78790256	11.6075615	10.0825926
A_30_P0102;	chr6:47692090-47713542_	lincRNA:chr6:		chr6:4769319	10.2314915	-1.9584994	0.25729594	1.11179101	2.16113771	-3.0702904	0.11905578	8.5552282	10.5137276	11.6255186
A_30_P0101!	chr3:41325168-41332366_	lincRNA:chr3:		chr3:4132528	6.86302114	-2.1929005	0.21871128	-1.4324232	0.37050805	-0.7604773	0.590301	5.87856187	8.07146236	6.63903917
A_51_P3228:	NM_026096	1700054O13I	Mus musculu	chrX:9424484	6.59274346	0.19472839	1.14450867	2.03450479	4.09682081	-1.8397764	0.27936508	6.04439412	5.84966573	7.88417052
A_55_P2138!	XM_885282	Gm6189	PREDICTED: I	chr1:8037965	7.40319824	0.45059755	1.36660617	2.19615916	4.58257713	-1.7455616	0.29821782	6.97154355	6.52094601	8.71710517
A_55_P2017;	NM_146780	Olfr715	Mus musculu	chr7:114272(	6.41284512	0.5849625	1.5	2.275278	4.84090909	-1.6903155	0.30985915	6.04439412	5.45943162	7.73470962
A_55_P1988;	ENSMUST00000101091		Putative uncl	chr6:1084211	7.45976849	-1.5950327	0.33101473	-2.03586	0.24386252	0.44082737	1.35738255	7.07503339	8.67006607	6.63420602
A_51_P2017;	NM_019922	Crtap	Mus musculu	chr9:1142845	8.13967741	-2.2871919	0.2048739	-0.8940195	0.53811278	-1.3931724	0.3807267	6.91288934	9.20008121	8.30606169
A_30_P0102·	chr9:114412295-11441838	lincRNA:chr9:		chr9:1144182	9.37445985	-3.1037928	0.11632291	-0.4337155	0.74035263	-2.6700773	0.15711825	7.44983646	10.5536293	10.1199138
A_66_P1362;	NM_030690	Rai14	Mus musculu	chr15:104989	8.79016469	-2.0513611	0.24125637	-1.3954131	0.38013582	-0.655948	0.63465833	7.88772834	9.93908942	8.54367631
A_55_P2141{	NM_178598	Tagln2	Mus musculu	chr1:1744367	8.53813538	-2.1883314	0.21940505	-0.3218376	0.80005021	-1.8664938	0.2742391	7.18652697	9.37485836	9.0530208
A_51_P3242{	NM_024245	Kif23	Mus musculu	chr9:6176773	9.22095342	-1.5520415	0.34102715	-2.5962091	0.16537246	1.04416758	2.06217617	9.05166212	10.6037036	8.00749454

A_51_P4973	NM_145100	Lypd1	Mus musculu	chr1:127768	9.42378416	-2.5320239	0.17289597	-1.0881076	0.47037796	-1.4439162	0.36756817	8.09847079	10.6304947	9.54238704
A_52_P6387	NM_0011643	Mipol1	Mus musculu	chr12:58407	9.269078	-2.1521187	0.22498196	-2.0184052	0.24683088	-0.1337135	0.91148225	8.50713391	10.6592527	8.64084744
A_30_P0103	chr13:23517278-23523756		lincRNA:chr1	chr13:235217	7.67437966	-0.4224752	0.74614338	-2.1172238	0.23049002	1.69474861	3.23720472	8.09847079	8.52094601	6.40372219
A_55_P1980	NM_144811	Cbx7	Mus musculu	chr15:797462	8.03594328	-2.0006077	0.24989471	-1.5314827	0.34592166	-0.469125	0.7224026	7.21269903	9.21330677	7.68182404
A_51_P2187	NM_026418	Rgs10	Mus musculu	chr7:135517	11.1318655	2.62114193	6.15236855	0.77515913	1.71137881	1.8459828	3.59497765	12.6209071	9.99976517	10.7749243
A_52_P6125	NM_178750	Ss18l1	Mus musculu	chr2:179804	10.9009656	2.18060852	4.53344732	1.7012222	3.2517632	0.47938632	1.39415051	11.7876305	9.60702201	11.3082442
A_52_P2783	NM_007557	Bmp7	Mus musculu	chr2:172695	8.128214	-2.9483074	0.12956003	-1.3413559	0.39464957	-1.6069515	0.32829132	6.60979435	9.55810178	8.21674586
A_55_P1988	NM_145150	Prc1	Mus musculu	chr7:874610	7.59203306	-0.9333669	0.52363488	-2.318405	0.200489	1.38503814	2.61178862	7.74259014	8.67595703	6.357552
A_51_P3788	NM_019703	Pfkp	Mus musculu	chr13:658387	10.975741	-2.6696445	0.1571654	1.08452106	2.12067135	-3.7541655	0.07411115	8.83447105	11.5041155	12.5886366
A_55_P2045	NM_0010117	Olfr106	Mus musculu	chr17:375323	6.81130425	0.63199603	1.5497076	2.30324667	4.93567251	-1.6712506	0.31398104	6.46488605	5.83289001	8.13613669
A_30_P0103	chr9:121845920-12185812		lincRNA:chr9	chr9:121855	8.36558566	3.05040495	8.28444444	0.35989595	1.28333333	2.69050901	6.45541126	10.2792236	7.22881869	7.58871464
A_55_P1983	NM_013613	Nr4a2	Mus musculu	chr2:569596	8.8478539	1.67010683	3.18238158	-0.7960633	0.57591855	2.46617014	5.52574942	10.2266129	8.55650605	7.76044275
A_55_P2002	NM_007940	Ephx2	Mus musculu	chr14:667032	6.94271861	-1.8080856	0.28556962	-2.4517819	0.18278481	0.64369633	1.56232687	6.55458885	8.36267444	5.91089253
A_51_P5070	NM_021560	Bhlhe22	Mus musculu	chr3:179569	8.56075749	-2.1647026	0.22302811	0.5253238	1.43925657	-2.6900264	0.15496063	6.94251451	9.10721708	9.63254088
A_51_P1103	NM_144943	Cd207	Mus musculu	chr6:836215	7.37135599	0.13683986	1.0994941	2.09645074	4.27655987	-1.9596109	0.25709779	6.76376565	6.62692579	8.72337653
A_30_P0101	chr9:121845920-12185812		lincRNA:chr9	chr9:121855	6.9905393	2.37517279	5.18797954	0.51825822	1.43222506	1.85691457	3.62232143	8.40123509	6.0260623	6.54432052
A_52_P5616	NM_147776	Vwa1	Mus musculu	chr4:155143	8.08880151	-0.4130696	0.75102375	1.67281914	3.18837019	-2.0858887	0.23555099	7.25581542	7.66888498	9.34170413
A_51_P1290	NM_009735	B2m	Mus musculu	chr2:121978	7.80449174	-2.7144229	0.15236221	-2.6712976	0.15698541	-0.0431253	0.97055019	6.88530901	9.59973194	6.92843429
A_55_P2001	NM_183180	Tspan18	Mus musculu	chr2:930434	11.7090947	-2.4026573	0.18911591	-1.4451113	0.36726384	-0.9575461	0.51493203	10.5890269	12.9916842	11.546573
A_55_P1967	NM_030717	Lactb	Mus musculu	chr9:668034	6.78969643	3.93005588	15.2427984	0.4194834	1.33744856	3.51057248	11.3969231	9.26990588	5.33985	5.75933341
A_55_P2022	NM_026162	Plxdc2	Mus musculu	chr2:166732	9.31295193	-2.2042093	0.21700357	-1.2157176	0.43055886	-0.9884917	0.50400442	8.24871825	10.4529276	9.23720996
A_51_P4082	NM_030218	9130017N09	Mus musculu	chr10:792907	8.12821035	0.4534028	1.36926606	2.38156279	5.21100917	-1.92816	0.26276408	7.63662462	7.18322182	9.56478462
A_55_P2274	NM_0011636	Wnt7b	Mus musculu	chr15:853669	7.68044733	-1.9928931	0.25123457	-2.1962117	0.21820988	0.20331858	1.15134371	7.08392248	9.0768156	6.8806039
A_52_P1753	NM_023755	Tcfcp2l1	Mus musculu	chr1:120581	7.64466864	-2.2065103	0.21665774	-2.7041149	0.15345474	0.49760456	1.41186736	7.07503339	9.28154371	6.57742883
A_55_P2112	NM_009322	Tbr1	Mus musculu	chr2:616520	8.72966781	2.72862667	6.62824381	-0.8678094	0.54797827	3.59643607	12.095815	10.8380221	8.10939539	7.24158599
A_52_P2636	NM_008236	Hes2	Mus musculu	chr4:151536	7.99857847	-1.3047036	0.40480427	-2.10284	0.23279953	0.79813639	1.7388535	7.82972274	9.13442632	7.03158634
A_52_P4184	NM_023476	Tinagl1	Mus musculu	chr4:129843	8.89022619	-4.1196558	0.05752545	0.03322511	1.02329712	-4.152881	0.05621578	6.13271392	10.2523698	10.2855949
A_30_P0102	lincP21		lincRNA:chr1	chr17:291948	7.46667822	-2.6766054	0.15640891	-1.6009884	0.32965106	-1.075617	0.47446809	6.2159374	8.89254282	7.29155445
A_51_P4281	NM_177152	Lrig3	Mus musculu	chr10:125452	7.41591873	-2.2991731	0.20317953	-1.6959714	0.30864676	-0.6032016	0.65829146	6.4484605	8.74763356	7.05166212
A_52_P3507	NM_015730	Chrna4	Mus musculu	chr2:180757	12.0319675	-2.9580188	0.12869083	-0.29186	0.81684823	-2.6661588	0.15754559	10.1572417	13.1152605	12.8234004
A_55_P2034	ENSMUST00000063878		sema domain	chr18:474361	8.05995504	-2.2677805	0.20764909	-2.3259863	0.1994382	0.05820578	1.0411701	7.32343012	9.59121065	7.26522434
A_51_P2813	NM_018784	St3gal6	Mus musculu	chr16:584707	7.73109623	-1.5737114	0.33594306	1.15282432	2.22348754	-2.7265357	0.15108835	6.29768055	7.87139191	9.02421624
A_55_P2111	NM_021324	Ttyh1	Mus musculu	chr7:408634	10.8237317	-2.8447611	0.13920075	-1.8474227	0.27788835	-0.9973384	0.50092328	9.54303182	12.387793	10.5403702
A_55_P2095	NM_019666	Syncrip	Mus musculu	chr9:883505	10.1835835	2.11255988	4.32457956	0.44432008	1.3606727	1.6682398	3.17826583	11.4438501	9.3312902	9.77561028
A_30_P0102	chr9:121845920-12185812		lincRNA:chr9	chr9:121855	7.39038109	2.02542708	4.07112376	-0.4715389	0.72119488	2.49696602	5.64497041	8.89784546	6.87241838	6.40087944
A_55_P1962	NM_207105	H2-Ab1	Mus musculu	chr17:344062	7.86344772	-2.9822496	0.12654746	-1.9448419	0.25974324	-1.0374077	0.48720212	6.52356196	9.50581155	7.56096965
A_30_P0102	chr9:14460976-14488051_		lincRNA:chr9	chr9:144845	8.74014421	-2.3573576	0.19514825	-3.204717	0.10846361	0.84735939	1.79920477	8.23681148	10.5941691	7.38945209
A_66_P1341	ENSMUST00000103384		Mus musculu	chr6:701674	8.3018495	1.1692092	2.24888393	-0.930838	0.52455357	2.10004718	4.28723404	9.39160162	8.22239242	7.29155445
A_66_P1034	XM_0014795	4930568B11	PREDICTED: M	chr5:145305	7.18834076	0.25420487	1.19267823	2.00693271	4.01926782	-1.7527278	0.29674017	6.6888331	6.43462823	8.44156094
A_30_P0102	chrX:151738814-15174096		lincRNA:chrX	chrX:151739	7.27851368	0.34696349	1.27188082	2.03713023	4.10428305	-1.6901667	0.30989111	6.83077927	6.48381578	8.52094601
A_55_P2094	NM_0010831	Lhx6	Mus musculu	chr2:359584	11.6740909	4.87462038	29.3364091	3.86798764	14.6009227	1.00663274	2.0092161	13.6345086	8.75988818	12.6278758
A_55_P2022	NM_008155	Gpi1	Mus musculu	chr7:349872	13.6371279	-2.7970038	0.1438858	-0.5544744	0.68090508	-2.2425295	0.21131551	11.9572835	14.7542873	14.1998129
A_55_P2110	NM_144848	Eppk1	Mus musculu	chr15:759319	8.51980467	-2.1158565	0.23070857	-0.9174114	0.52945815	-1.198445	0.43574468	7.4150375	9.53089398	8.61348254
A_55_P2129	NM_0010814	Chd7	Mus musculu	chr4:879385	12.3654308	-0.9083126	0.53280791	-2.1856107	0.21981919	1.27729815	2.42384618	12.488426	13.3967386	11.2111278

A_51_P4502	NM_027237	2010003K11F	Mus musculu	chr19:44969	6.76536533	-0.0298242	0.97953964	2.2477333	4.74936061	-2.2775575	0.20624663	5.99623808	6.0260623	8.2737956
A_51_P4429	NM_029617	Casc5	Mus musculu	chr2:118914	8.26002775	-0.9394978	0.52141434	-2.4401621	0.18426295	1.50066427	2.82972973	8.44708323	9.38658105	6.94641896
A_51_P4139	NM_008257	Hmx3	Mus musculu	chr7:138688	7.34510719	-2.6083629	0.16398515	-2.5760624	0.16769802	-0.0323005	0.97785978	6.46488605	9.07324898	6.49718654
A_55_P2071	NM_205769	Crh	Mus musculu	chr3:195935	7.12759733	2.15615872	4.45726496	0.37042661	1.29273504	1.78573211	3.44793388	8.44156094	6.28540222	6.65582883
A_51_P4360	NM_007412	Gpr182	Mus musculu	chr10:12718	7.76457388	0.261094	1.1983871	2.95914193	7.7766129	-2.6980479	0.15410142	6.9522559	6.6911619	9.65030383
A_30_P0103	chr2:115900355-11590090	lincRNA:chr2	chr2:115900	7.55352937	-2.2083227	0.21638573	-1.3267432	0.39866719	-0.8815795	0.54277286	6.52356196	8.73188468	7.40514146
A_52_P3761	NM_177139	Lypd6	Mus musculu	chr2:500479	9.70854082	1.29786736	2.45865169	2.22387372	4.67146067	-0.9260064	0.52631326	9.83249448	8.53462712	10.7585008
A_30_P0102	chr8:124355710-12440116	lincRNA:chr8	chr8:124397	8.15100676	-2.6626785	0.1579261	-0.2669948	0.83104887	-2.3956837	0.19003227	6.46488605	9.1275645	8.86056972
A_55_P2111	NM_146523	Olfr850	Mus musculu	chr9:192818	6.63319117	0.45444247	1.37025316	2.28867629	4.88607595	-1.8342338	0.28044041	6.17326071	5.71881825	8.00749454
A_30_P0102	chr14:55433382-55452757	lincRNA:chr1	chr14:554451	9.69602703	-3.1614342	0.11176697	-1.7794865	0.29128705	-1.3819477	0.38370044	8.18156641	11.3430006	9.56351408
A_55_P2027	ENSMUST000	Neto1	neuropilin (N	chr18:86672	9.74867508	2.01966496	4.05489614	2.03654594	4.10262117	-0.016881	0.98836719	10.4162697	8.39660478	10.4331507
A_52_P1468	NM_053136	Pcdhb11	Mus musculu	chr18:37583	9.21332018	-2.318674	0.20045162	0.24021753	1.18117075	-2.5588915	0.16970588	7.58746501	9.906139	10.1463565
A_55_P2008	NM_019923	Itpr2	Mus musculu	chr6:146060	7.15999115	-3.0053073	0.124541	-2.7824086	0.14534884	-0.2228987	0.85684211	6.08392248	9.08922977	6.3068212
A_51_P2794	NM_029662	Mfsd2a	Mus musculu	chr4:122624	7.74266262	-1.5055978	0.35218422	-2.0954237	0.23399932	0.58982591	1.50506512	7.43740531	8.94300314	6.8475794
A_55_P1954	NM_010128	Emp1	Mus musculu	chr6:135333	7.81842181	-2.511182	0.17541184	-1.3144655	0.40207444	-1.1967165	0.43626707	6.58245565	9.09363764	7.77917215
A_30_P0102	chr10:66559716-66647841	lincRNA:chr1	chr10:66601	7.88599952	1.17232989	2.25375375	-0.8975789	0.53678679	2.06990883	4.1986014	8.96674576	7.79441587	6.89683693
A_30_P0101	chr12:82500406-82501102	lincRNA:chr1	chr12:82500	6.30226708	1.75140541	3.36686391	-0.292355	0.81656805	2.04376039	4.12318841	7.56732234	5.81591694	5.52356196
A_51_P4833	NM_145367	Txndc5	Mus musculu	chr13:38592	10.6146835	-2.1987218	0.21783055	-0.8894724	0.53981149	-1.3092494	0.40353077	9.44535978	11.6440816	10.7546092
A_55_P1967	NM_008221	Hbb-y	Mus musculu	chr7:111000	8.4513364	6.08103055	67.6974952	-0.030906	0.97880539	6.11193659	69.1633858	12.5156588	6.43462823	6.40372219
A_30_P0102	chrX:135880775-13589152	lincRNA:chrX	chrX:135882	6.9949185	1.61022355	3.05299145	-0.447459	0.73333333	2.05768253	4.16317016	8.21755386	6.60733031	6.15987134
A_55_P2053	NM_010137	Epas1	Mus musculu	chr17:87230	7.37125078	-2.146466	0.22586521	-1.2868811	0.40983607	-0.8595849	0.55111111	6.36923381	8.51569984	7.22881869
A_55_P1979	NM_016764	Prdx4	Mus musculu	chrX:151758	8.60181648	-1.4522073	0.36546185	-2.2457564	0.21084337	0.79354912	1.73333333	8.38226376	9.83447105	7.58871464
A_66_P1214	NM_007681	Cenpa	Mus musculu	chr5:309749	9.60876717	-1.3959961	0.37998224	-2.1789867	0.2208308	0.78299059	1.72069403	9.404432	10.8004281	8.62144141
A_55_P2027	NM_177838	Fam163a	Mus musculu	chr1:157923	6.85330103	-2.6288444	0.16167355	-1.8129547	0.28460744	-0.8158897	0.56805808	5.70505635	8.33390074	6.52094601
A_55_P1997	NM_008652	Mybl2	Mus musculu	chr2:162909	9.05680019	-2.3968902	0.18987342	-2.5552181	0.17013854	0.15832794	1.11599297	8.31061278	10.7075029	8.15228484
A_30_P0102	chr18:35046847-35047423	lincRNA:chr1	chr18:35047	7.28118793	0.50695999	1.42105263	2.24638962	4.74493927	-1.7394296	0.29948805	6.87036472	6.36340473	8.60979435
A_55_P2170	NM_026560	Cdca8	Mus musculu	chr4:124599	9.91667747	-1.7298277	0.30148797	-2.2011825	0.21745932	0.47135487	1.38641087	9.49718654	11.2270142	9.02583167
A_55_P1988	XR_033805	Gm8865	PREDICTED: I	chr14:554451	8.68542107	-2.7227702	0.15148321	-1.5576334	0.33970788	-1.1651368	0.44592199	7.38945209	10.1122223	8.55458885
A_55_P2344	AK044369	A930009L07F	Mus musculu	chr15:72338	8.46330031	-2.7293888	0.15078985	-0.2116132	0.86357109	-2.5177756	0.17461197	6.71424552	9.44363428	9.23202112
A_55_P2162	NM_009643	Ahnak	Mus musculu	chr19:90905	7.23460674	-2.3166724	0.20072993	-1.5187161	0.34899635	-0.7979562	0.5751634	6.19639721	8.51306958	6.99435344
A_52_P2208	NM_009373	Tgm2	Mus musculu	chr2:157942	7.23633662	-3	0.125	-2.2797044	0.20593995	-0.7202956	0.60697306	5.99623808	8.99623808	6.71653369
A_52_P1518	NM_0010252	Tpd52	Mus musculu	chr3:894753	9.90491364	-3.0041589	0.12464018	-1.6472103	0.31925691	-1.3569487	0.39040714	8.45121111	11.45537	9.80815977
A_55_P2031	NM_027174	Col22a1	Mus musculu	chr15:71628	7.41063375	-2.9222946	0.13191727	-2.5057297	0.17607602	-0.416565	0.74920635	6.29768055	9.21997517	6.71424552
A_55_P1968	NM_008115	Gfra2	Mus musculu	chr14:71379	9.44453181	-2.0944414	0.23415872	-1.2560657	0.41868417	-0.8383756	0.55927292	8.46692616	10.5613675	9.30530178
A_51_P1131	NM_013584	Lifr	Mus musculu	chr15:71411	8.4072434	-2.2867029	0.20494336	-2.2615494	0.20854789	-0.0251535	0.98271605	7.63662462	9.92332749	7.6617781
A_52_P3877	NM_0010798	Tcf7l1	Mus musculu	chr6:725764	7.62126631	-2.4180117	0.18711386	-3.4010983	0.09466019	0.98308662	1.97668998	7.14295795	9.56096965	6.15987134
A_55_P1983	NM_009805	Cflar	Mus musculu	chr1:587899	6.88654452	-1.2722668	0.41400876	-2.2420748	0.21138211	0.96980797	1.95857988	6.78572491	8.05799172	5.81591694
A_55_P2010	NM_0010082	Daam2	Mus musculu	chr17:49595	7.11087053	-2.2722159	0.20701169	-2.3195216	0.20033389	0.04730571	1.03333333	6.36923381	8.64144969	6.32192809
A_55_P2358	ENSMUST00000054384		tripartite mo	chr5:137581	7.02225535	-1.8700907	0.27355623	-2.8161519	0.14198871	0.94606119	1.9266055	6.71424552	8.5843362	5.76818432
A_55_P1958	NM_178408	Arrdc1	Mus musculu	chr2:247824	10.2173808	-2.1146467	0.23090211	0.48047353	1.39520154	-2.5951203	0.16549732	8.64745843	10.7621052	11.2425787
A_30_P0102	chr15:61984389-62102500	lincRNA:chr1	chr15:62086	7.53326148	-2.4102333	0.18812542	-1.8844488	0.27084723	-0.5257846	0.69458128	6.55458885	8.96482217	7.08037342
A_55_P2181	NM_0010835	Tns3	Mus musculu	chr11:83345	7.35536424	-2.1452727	0.2260521	-1.8882193	0.27014028	-0.2570534	0.83679525	6.55458885	8.6998616	6.81164228
A_52_P5284	NM_018867	Cpxm2	Mus musculu	chr7:139234	7.80593922	1.02204402	2.03079417	2.34328111	5.07455429	-1.3212371	0.40019163	7.7062082	6.68416418	9.02744529
A_52_P5498	NM_019946	Mgst1	Mus musculu	chr6:138104	7.14000706	-2.8803022	0.1358134	-3.1221659	0.1148509	0.24186371	1.18251928	6.26052755	9.14082977	6.01866384

A_55_P1996	NM_026785	Ube2c	Mus musculu	chr2:1645974	10.1894076	-1.8614196	0.27520534	-3.1220901	0.11485694	1.26067049	2.39607073	9.98915793	11.8505775	8.72848744
A_55_P2053	NM_008259	Foxa1	Mus musculu	chr12:586418	8.2411101	-4.9519305	0.03230877	-3.0197876	0.12329724	-1.9321429	0.26203966	5.94641896	10.8983495	7.87856187
A_52_P5932	ENSMUST00000114435		RIKEN cDNA 5	chr6:5211910	8.41856105	-4.5240089	0.04346479	-2.1904763	0.21907908	-2.3335325	0.19839774	6.13271392	10.6567228	8.46624644
A_65_P1605	NM_011578	Tgfbr3	Mus musculu	chr5:1075358	8.23653672	-2.3952647	0.19008746	-0.3722162	0.77259475	-2.0230485	0.24603774	6.76376565	9.15903036	8.78681414
A_51_P2288	ENSMUST00000127420		staufen (RNA	chr1:1633466	9.64045351	2.60556729	6.08630778	1.85387071	3.61468695	0.75169658	1.68377175	10.7595415	8.15397418	10.0078449
A_55_P1978	NM_139064	Tnip2	Mus musculu	chr5:3483883	8.89153557	-2.1564229	0.22431175	-0.411871	0.75164793	-1.7445519	0.29842662	7.59121065	9.74763356	9.33576252
A_51_P2686	NM_148938	Slc1a3	Mus musculu	chr15:858429	8.52256306	-4.2881379	0.0511849	-3.8653544	0.06861394	-0.4227835	0.74598394	6.9522559	11.2403938	7.37503943
A_51_P2542	NM_130858	Nxph3	Mus musculu	chr11:953712	8.42380472	-2.2163924	0.21517877	-1.220811	0.42904146	-0.9955813	0.50153374	7.35314683	9.56953919	8.34872815
A_55_P1961	NM_009150	Selenbp1	Mus musculu	chr3:9474862	7.4056425	-2.8524428	0.13846154	-2.7415157	0.14952767	-0.1109272	0.92599278	6.41785251	9.27029533	6.52877967
A_30_P0103	chr1:63319842-63349796_		lincRNA:chr1:	chr1:6332738	10.4537546	-2.1365912	0.22741649	0.26640636	1.20280799	-2.4029976	0.18907132	8.94055831	11.0771495	11.3435559
A_55_P2057	NM_029441	Cdyl2	Mus musculu	chr8:1190983	7.74027221	1.15396582	2.22524752	-0.9077836	0.5330033	2.06174945	4.1749226	8.81217731	7.65821148	6.75042785
A_66_P1113	NM_013833	Rax	Mus musculu	chr18:660943	7.92295827	-0.1622012	0.89366053	1.88489152	3.69325153	-2.0470927	0.24197121	7.18652697	7.34872815	9.23361968
A_51_P5135	NM_017407	Spag5	Mus musculu	chr11:781356	7.69600942	-1.556854	0.33989145	-2.1767927	0.22116689	0.61993864	1.53680982	7.38370429	8.94055831	6.76376565
A_51_P3299	NM_013750	Phlda3	Mus musculu	chr1:1376656	9.17860637	-3.4923125	0.08886059	-1.1687947	0.44479279	-2.3235178	0.19977974	7.23999624	10.7323088	9.56351408
A_55_P2026	NM_022378	Foxb1	Mus musculu	chr9:6960561	6.56922082	-2.053959	0.24082232	-1.718024	0.30396476	-0.335935	0.79227053	5.7725895	7.82654849	6.10852446
A_30_P0102	chr5:137392195-13742642		lincRNA:chr5:	chr5:1374098	9.39801322	-1.1896062	0.43842251	0.89208628	1.85585793	-2.0816925	0.23623711	8.30758032	9.49718654	10.3892728
A_30_P0101	chr8:4325209-4348788_F		lincRNA:chr8:	chr8:4348678	10.0685271	-2.0408517	0.24302022	-0.5393072	0.68810124	-1.5015445	0.3531751	8.88772834	10.9285801	10.3892728
A_51_P1318	NM_007806	Cyba	Mus musculu	chr8:1249511	8.65580666	-2.8093915	0.14265562	-0.8764633	0.54470111	-1.9329283	0.26189705	7.07503339	9.88442494	9.00796165
A_30_P0101	chr8:72077827-72109034_		lincRNA:chr8:	chr8:7207791	9.82998257	-1.5175614	0.3492758	0.67932295	1.60138805	-2.1968844	0.21810816	8.59183398	10.1093954	10.7887183
A_55_P2106	NM_009304	Syngr2	Mus musculu	chr11:117675	8.29845222	-2.2277092	0.21349745	-1.7284181	0.30178268	-0.4992912	0.70745429	7.38945209	9.61716132	7.88874325
A_51_P1357	ENSMUST00000031781		coiled-coil do	chr6:2936813	9.68233033	-2.0214952	0.24630277	0.24127664	1.18203818	-2.2627719	0.20837125	8.25424129	10.2757365	10.5170132
A_51_P4928	NM_028767	Foxp4	Mus musculu	chr17:480046	7.45267731	-1.3236972	0.3995098	-2.3355469	0.19812092	1.01184972	2.01649485	7.34872815	8.67242534	6.33687844
A_51_P2672	NM_021301	Slc15a2	Mus musculu	chr16:367506	7.77177631	-2.2334901	0.21264368	1.00909178	2.01264368	-3.2425819	0.10565391	5.94641896	8.17990909	9.18900087
A_30_P0103	chr12:50490638-50508093		lincRNA:chr1	chr12:505080	7.88151874	2.72051042	6.5910596	0.96371908	1.95033113	1.75679133	3.37945671	9.37395266	6.65344224	7.61716132
A_55_P2026	NM_009062	Rgs4	Mus musculu	chr1:1716716	8.53433791	2.33763482	5.05473278	-0.7822599	0.58145525	3.11989476	8.69324474	10.3535144	8.01587961	7.23361968
A_52_P4494	NM_177545	Vangl1	Mus musculu	chr3:1019607	8.20895427	-3.2620806	0.10423555	-2.2544732	0.2095733	-1.0076074	0.4973704	6.78572491	10.0478056	7.79333236
A_51_P1430	NM_133867	Eps8l3	Mus musculu	chr3:1076957	10.7279852	-1.4563262	0.36441993	0.65722153	1.57704248	-2.1135477	0.23107807	9.53802722	10.9943534	11.651575
A_55_P1956	NM_021324	Ttyh1	Mus musculu	chr7:4086862	11.190448	-3.5192798	0.087215	-2.5585528	0.16974574	-0.9607271	0.51379791	9.69711237	13.2163922	10.6578395
A_51_P1686	NM_010635	Klf1	Mus musculu	chr8:8742888	8.94486042	-1.378053	0.38473768	1.11098884	2.15993641	-2.4890418	0.17812454	7.65582883	9.0338818	10.1448706
A_55_P2129	XR_035510	Gm5276	PREDICTED: M	chr3:5716720	11.8329024	-2.6499073	0.15933032	-0.7561708	0.59206572	-1.8937365	0.26910918	10.3183545	12.9682618	12.212091
A_52_P1838	NM_178788	Dctd	Mus musculu	chr8:4919739	9.22528239	-2.4697822	0.1805184	-2.3121138	0.20136519	-0.1576684	0.89647274	8.34946554	10.8192477	8.50713391
A_52_P1600	NM_211138	Pcyt1b	Mus musculu	chrX:9099462	9.61837925	2.27487456	4.83955556	0.92802283	1.90266667	1.34685173	2.54356459	10.8256213	8.55074679	9.47876962
A_52_P6549	NM_027711	Iqgap2	Mus musculu	chr13:963989	7.70573182	-2.8915882	0.1347551	-2.413348	0.18771971	-0.4782403	0.71785268	6.58245565	9.47404389	7.06069593
A_51_P2400	NM_013755	Gyg	Mus musculu	chr3:2002207	8.77559355	-2.6151562	0.1632148	-2.0221564	0.24618992	-0.5929998	0.66296296	7.7062082	10.3213644	8.29920802
A_55_P2152	NM_010544	Ihh	Mus musculu	chr1:7499196	7.3830396	0.96656577	1.95418327	2.02280988	4.06374502	-1.0562441	0.48088235	7.35314683	6.38658105	8.40939094
A_55_P2057	NM_007492	Arx	Mus musculu	chrX:9054361	13.3766751	2.41793505	5.34405574	1.30752789	2.47517047	1.11040716	2.15906573	14.5527892	12.1348541	13.442382
A_55_P2429	NM_0011901	Psrc1	Mus musculu	chr3:1081907	8.51760947	-2.5821671	0.16698991	-1.1062266	0.46450737	-1.4759405	0.35949896	7.16490693	9.74707405	8.64084744
A_55_P2103	XM_485921	Gm5593	PREDICTED: M	chr13:101549	7.54294835	-1.3039337	0.40502035	-2.8888962	0.13500678	1.5849625	3	7.63662462	8.94055831	6.05166212
A_30_P0102	chr1:64686823-64728598_		lincRNA:chr1:	chr1:6472686	7.9651638	-1.0368042	0.48740595	-2.0465194	0.24206739	1.00971515	2.01351351	7.95613411	8.99293834	6.94641896
A_55_P1962	NM_008218	Hba-a1	Mus musculu	chr11:321839	10.3182424	3.61655902	12.2657115	0.33957739	1.26538587	3.27698163	9.69325785	12.6160893	8.9995303	9.33910768
A_55_P2058	NM_177145	Pde4dip	Mus musculu	chr3:9762607	7.8987209	1.87637654	3.67151767	-0.1550056	0.8981289	2.03138213	4.08796296	9.20130713	7.32493058	7.169925
A_55_P1983	NM_0010122	Birc5	Mus musculu	chr11:117714	8.95020485	-1.6226986	0.32472748	-2.0738699	0.23752151	0.45117129	1.36714976	8.55969574	10.1823944	8.10852446
A_51_P4267	NM_009673	Anxa5	Mus musculu	chr3:3634812	8.61031869	-2.7395697	0.14972949	-1.8932958	0.2691914	-0.8462739	0.55621945	7.4150375	10.1546072	8.26131141
A_66_P1160	NM_146828	Olfr975	Mus musculu	chr9:3975748	7.44574777	0.14313821	1.10430464	2.23377838	4.70364238	-2.0906402	0.23477649	6.79658045	6.65344224	8.88722062

A_30_P01022	chr17:86560289-86565864		lincRNA:chr17	chr17:865653	7.61859937	-2.1366442	0.22740814	-1.9351016	0.26150281	-0.2015426	0.86962025	6.83920379	8.97584797	7.04074634
A_55_P19937	NM_019472	Myo10	Mus musculu	chr15:257402	8.66271799	-2.8555956	0.13815928	-1.7824776	0.29068375	-1.0731179	0.4752907	7.35314683	10.2087424	8.42626475
A_30_P01024	chr1:23340097-23340552_		lincRNA:chr1:	chr1:2334048	7.35243479	0.34372225	1.26902655	2.04214844	4.11858407	-1.6984262	0.30812205	6.90086681	6.55714456	8.59929299
A_55_P19826	NM_0011596	Nadk	Mus musculu	chr4:154965	7.99345421	0.93014195	1.90546347	-1.2042045	0.43400859	2.13434644	4.3903819	9.01495034	8.08480839	6.8806039
A_51_P47940	NM_008317	Hyal1	Mus musculu	chr9:1074818	7.50621931	-2.6189098	0.1627907	-1.7741881	0.2923588	-0.8447218	0.55681818	6.35167544	8.97058527	7.19639721
A_55_P21432	NM_146131	Pbxip1	Mus musculu	chr3:8925411	9.12345677	-4.1539409	0.0561745	-2.3098754	0.20167785	-1.8440655	0.27853577	7.12412131	11.2780622	8.96818677
A_55_P20747	NM_007671	Cdkn2c	Mus musculu	chr4:1093335	6.7340224	-1.1055828	0.46471471	-2.0755976	0.23723724	0.97001485	1.95886076	6.6888331	7.79441587	5.71881825
A_30_P01023	chr1:34734249-34786649_		lincRNA:chr1:	chr1:3478066	8.64858445	-4.74814	0.03721067	-2.7616932	0.14745092	-1.9864468	0.25235966	6.40372219	11.1518622	8.39016896
A_51_P46376	NM_011595	Timp3	Mus musculu	chr10:858116	8.68639317	-2.6916741	0.15478375	-2.1866609	0.21965924	-0.5050132	0.70465394	7.62083075	10.3125048	8.12584393
A_30_P01032	chr16:46384974-46391331		lincRNA:chr16	chr16:463898	7.54109116	-2.3319199	0.19861963	-1.3361036	0.39608896	-0.9958163	0.50145208	6.43184579	8.76376565	7.42766204
A_51_P33719	NM_008952	Pipox	Mus musculu	chr11:776943	9.60635135	-3.5741223	0.08396185	-2.2667515	0.20779725	-1.3073708	0.40405658	7.97918699	11.5533093	9.28655776
A_55_P20243	NM_183024	Raver2	Mus musculu	chr4:1008248	7.38267986	-1.3775628	0.38486842	-2.4632927	0.18133224	1.08572987	2.12244898	7.28540222	8.66296501	6.19967234
A_51_P43724	NM_007929	Emp2	Mus musculu	chr16:102820	7.18656604	-2.2319818	0.21286611	-1.156033	0.44874477	-1.0759489	0.47435897	6.08392248	8.31590431	7.15987134
A_30_P01027	chr1:87343550-87362900_		lincRNA:chr1:	chr1:8735428	7.50968641	2.27981444	4.85615491	-0.4894232	0.71230982	2.76923766	6.81747573	9.19270378	6.91288934	6.42346612
A_52_P58888	NM_0010334	Iqgap3	Mus musculu	chr3:8792476	8.51444658	-2.0091759	0.24841499	-3.940541	0.06512968	1.9313651	3.81415929	8.48850965	10.4976855	6.55714456
A_55_P19790	NM_0011133	Shc1	Mus musculu	chr3:8923386	8.90993118	-2.0888354	0.23507037	-2.2622663	0.20844428	0.17343094	1.12773723	8.27146303	10.3602984	8.09803208
A_55_P20879	XM_0014728	Gm15452	PREDICTED: M	chr1:8781796	7.28480121	-0.8779717	0.54413191	-2.5422231	0.17167798	1.66425141	3.16949153	7.54689446	8.42486612	5.88264305
A_30_P01021	H19		lincRNA:chr7:	chr7:1497617	10.2391921	-5.0660798	0.02985094	-2.160068	0.22374572	-2.9060118	0.13341458	7.58182825	12.6479081	10.48784
A_55_P20649	NM_010329	Pdpn	Mus musculu	chr4:1428591	10.0977707	-4.6052926	0.04108363	-2.2218261	0.21436985	-2.3834665	0.19164835	7.76818432	12.3734769	10.1516508
A_30_P01023	chr8:122728333-12275213		lincRNA:chr8:	chr8:1227283	8.83108792	-2.3520481	0.19586777	-0.1221746	0.91880165	-2.2298734	0.21317742	7.30378075	9.65582883	9.53365419
A_52_P27122	NM_153177	Eif2c4	Mus musculu	chr4:1261672	9.07791899	2.0589333	4.16678106	1.40335569	2.64516129	0.6555776	1.5752465	9.98275596	7.92382266	9.32717836
A_51_P31764	NM_009367	Tgfb2	Mus musculu	chr1:1884475	8.48839739	-0.1807682	0.88223309	-2.1367982	0.22738386	1.95603	3.87992832	9.08015131	9.26091953	7.12412131
A_52_P35516	NM_011607	Tnc	Mus musculu	chr4:6367501	7.19581073	-2.4398985	0.18429662	-0.7401156	0.59869138	-1.6997829	0.30783242	5.81591694	8.25581542	7.51569984
A_52_P29079	NM_022320	Gpr35	Mus musculu	chr1:9488191	7.35084996	0.41090961	1.32952381	2.28800693	4.88380952	-1.8770973	0.27223089	6.86212073	6.45121111	8.73921805
A_55_P20041	NM_0011135	Col2a1	Mus musculu	chr15:978061	7.53804751	-2.2110811	0.21597241	-3.0597871	0.11992571	0.84870602	1.80088496	7.08392248	9.29500358	6.23521646
A_55_P20611	XM_0014715	LOC1000443	PREDICTED: M	chrX:1335084	7.92549413	2.76790663	6.81118881	-0.4710382	0.72144522	3.23894486	9.44103393	9.92777796	7.15987134	6.6888331
A_51_P41068	NM_018826	Irx5	Mus musculu	chr8:9488492	7.48303623	-3.1551132	0.11225774	-2.5089299	0.17568588	-0.6461833	0.63896848	6.2159374	9.37105058	6.86212073
A_30_P01020	chr11:53592946-53637922		lincRNA:chr11	chr11:536309	8.02003133	2.44739879	5.4543179	0.44142763	1.35794743	2.00597115	4.01658986	9.50448798	7.05708919	7.49851683
A_30_P01033	chr1:183951156-18398615		lincRNA:chr1:	chr1:1839590	8.00244042	0.21452641	1.16032295	2.26801781	4.816609	-2.0534914	0.24090038	7.38945209	7.17492568	9.4429435
A_55_P21379	NM_0011360	Cd82	Mus musculu	chr2:9326007	8.83911636	-4.1540889	0.05616873	-2.6475965	0.15958573	-1.5064925	0.35196589	6.9522559	11.1063448	8.45874836
A_52_P33860	XR_032468	Gm6639	PREDICTED: M	chr3:3552642	6.54669809	-2.0893475	0.23498695	-2.2592725	0.20887728	0.169925	1.125	5.9068906	7.99623808	5.73696559
A_52_P58565	NM_173182	Fndc3b	Mus musculu	chr3:2731578	7.04488147	-1.8021183	0.28675325	-2.040278	0.24311688	0.23815974	1.17948718	6.52356196	8.32568023	6.28540222
A_51_P20440	NM_011369	Shcbp1	Mus musculu	chr8:4736060	7.55046882	-0.8144647	0.56861942	-2.5390111	0.17206063	1.72454633	3.3047619	7.85382935	8.66829408	6.12928302
A_55_P19728	NM_008453	Klf3	Mus musculu	chr5:6522130	8.76863876	-2.2009693	0.21749146	-1.5978551	0.33036778	-0.6031142	0.65833133	7.83394423	10.0349136	8.43705847
A_51_P45755	NM_153393	Col23a1	Mus musculu	chr11:513814	7.42728517	-1.6059114	0.32852807	0.55025048	1.46433991	-2.1561619	0.22435233	6.17326071	7.77917215	8.32942263
A_66_P13671	BC021399	Scpep1	Mus musculu	chr11:887854	9.59452191	-2.7209561	0.15167381	-1.6060595	0.32849437	-1.1148967	0.46172422	8.31590431	11.0368604	9.43080099
A_55_P19611	NM_011141	Pou3f1	Mus musculu	chr4:1243376	9.55411837	2.72611013	6.61669203	0.8569963	1.81126332	1.86911383	3.65308123	11.0858597	8.35974956	9.21674586
A_55_P19534	NM_021477	A2bp1	Mus musculu	chr16:740999	9.71759469	-2.1146892	0.23089531	-0.7365223	0.60018439	-1.3781669	0.38470729	8.5533093	10.6679985	9.93147623
A_55_P20589	NM_027290	Mcm10	Mus musculu	chr2:4911844	7.89578311	-1.1478082	0.45131034	-2.8812508	0.13572414	1.73344253	3.32520325	8.09099453	9.23880278	6.357552
A_55_P20522	NM_177420	Psat1	Mus musculu	chr19:159796	11.2275748	-2.6244673	0.16216481	-1.8943965	0.2689861	-0.7300709	0.6028743	10.1093954	12.7338627	10.8394663
A_55_P20200	AK164572	2610035D17R	Mus musculu	chr11:113061	10.4402682	-2.8418451	0.13948239	-0.7347457	0.60092393	-2.1070994	0.23211322	8.79062001	11.6324651	10.8977194
A_52_P38166	NM_133919	Aff1	Mus musculu	chr5:1042842	7.37810757	-2.0428666	0.24268105	-2.0941472	0.23420647	0.0512805	1.03618421	6.71424552	8.75711217	6.66296501
A_51_P30774	NM_009827	Cckar	Mus musculu	chr5:5409010	8.05499778	-2.8847811	0.13539243	-1.8154656	0.28411254	-1.0693155	0.47654505	6.73696559	9.62174665	7.80628109
A_55_P20820	NM_0011659	Lingo2	Mus musculu	chr4:3565471	7.76104552	2.13959045	4.40636943	0.04878707	1.0343949	2.09080338	4.25985222	9.1711768	7.03158634	7.08037342

A_55_P2029	NM_0011775	Gm7849	Mus musculu	chr8:2256597	6.7385155	0.10829962	1.07795699	2.24465795	4.73924731	-2.1363583	0.2274532	6.06249593	5.95419631	8.19885426
A_55_P2369	NM_010283	Ggta1	Mus musculu	chr2:3525768	7.73325817	-2.7062182	0.15323118	-0.9942446	0.50199867	-1.7119737	0.3052422	6.26052755	8.96674576	7.9725012
A_55_P1987	NM_013917	Pttg1	Mus musculu	chr11:432338	9.95924301	-1.2350496	0.42482789	-3.5423462	0.08583167	2.30729655	4.94954722	10.3166587	11.5517083	8.0093621
A_55_P1965	NM_145492	Zfp521	Mus musculu	chr18:13845	8.03415327	1.05582961	2.07891332	-0.9814561	0.50646831	2.03728572	4.10472542	9.06519171	8.0093621	7.027906
A_55_P2046	ENSMUST00000019416		PREDICTED: N	chr7:3813627	6.28746926	0.357552	1.28125	2.25974326	4.7890625	-1.9021913	0.2675367	5.7725895	5.4150375	7.67478076
A_51_P4610	NM_172416	Ostm1	Mus musculu	chr10:42422	9.13356856	2.11017237	4.31742876	1.36728954	2.57985421	0.74288283	1.67351657	10.084587	7.97441459	9.34170413
A_66_P1280	XR_004919	Gm5604	PREDICTED: N	chr7:2414377	7.95342136	-2.7193758	0.15184005	-0.9238689	0.5270936	-1.7955068	0.28807037	6.4484605	9.16783626	8.24396733
A_55_P2391	AY672066		Mus musculu	chr2:1670181	6.98914513	-0.4041681	0.7556719	1.63931703	3.11518325	-2.0434851	0.24257703	6.17326071	6.57742883	8.21674586
A_55_P1985	NM_0010776	Nkx2-2	Mus musculu	chr2:1470033	8.04979551	-3.9853035	0.06313993	-2.0729879	0.23766677	-1.9123156	0.2656658	6.08392248	10.069226	7.99623808
A_52_P3550	NM_144797	Metrnl	Mus musculu	chr11:121577	7.278498	-0.16932	0.88926174	2.10219591	4.29362416	-2.2715159	0.20711215	6.46488605	6.63420602	8.73640193
A_55_P2110	NM_201411	Flrt1	Mus musculu	chr19:71691	10.094038	-2.150729	0.2251988	-0.7952654	0.57623717	-1.3554636	0.39080922	8.92530717	11.0760362	10.2807708
A_51_P5038	ENSMUST000	Pycr1	pyrroline-5-c	chr11:120501	10.1036642	-2.022448	0.24614017	-0.6956953	0.61741169	-1.3267527	0.39866457	8.98726401	11.009712	10.3140167
A_30_P0102	XIST		lincRNA:chrX	chrX:100656	8.28737867	6.20878501	73.9657258	-0.4543504	0.72983871	6.66313543	101.345304	12.5780188	6.36923381	5.91488339
A_55_P1997	XR_033957	Gm5452	PREDICTED: N	chr13:839731	11.9645374	-2.9034875	0.13364822	-0.4710863	0.7214212	-2.4324012	0.18525685	10.1859078	13.0893953	12.618309
A_52_P3438	NM_178661	Creb3l2	Mus musculu	chr6:3728126	7.50070503	-2.4579833	0.1820008	-1.1290657	0.45721173	-1.3289176	0.39806678	6.23840474	8.69638801	7.56732234
A_55_P2105	NM_010712	Lhx4	Mus musculu	chr1:1575489	7.52311235	-3.9061105	0.06670272	-3.0821409	0.11808185	-0.8239696	0.5648855	5.94641896	9.85252951	6.7703886
A_55_P2143	ENSMUST00000105034		Putative unch	chr4:1205691	10.1576773	-2.550662	0.17067669	0.13717478	1.09974937	-2.6878368	0.15519599	8.41151099	10.962173	11.0993478
A_51_P4276	NM_007725	Cnn2	Mus musculu	chr10:79457	9.37477987	-4.0409827	0.06074954	-0.8951868	0.53767755	-3.1457959	0.11298507	6.97918699	11.0201697	10.1249829
A_51_P3338	NM_175448	Clvs2	Mus musculu	chr10:33232	6.89722442	1.48680476	2.80267559	-0.712249	0.61036789	2.19905378	4.59178082	8.12584393	6.63903917	5.92679015
A_51_P3604	NM_008567	Mcm6	Mus musculu	chr1:1302282	10.4746131	-0.8777966	0.54419793	-2.1583665	0.22400976	1.2805699	2.42934924	10.6088708	11.4866675	9.32830093
A_30_P0102	chr5:22890322-22939119_		lincRNA:chr5	chr5:2289038	8.7020486	2.0987193	4.28328982	0.01871226	1.01305483	2.08000704	4.22809278	10.0949574	7.99623808	8.01495034
A_55_P2002	XM_0014734	Gm2399	PREDICTED: N	chr13:127948	8.5116406	-3.6866756	0.07766048	-2.0493262	0.24159689	-1.6373494	0.32144651	6.73696559	10.4236412	8.374315
A_51_P3025	NM_172778	Maob	Mus musculu	chrX:1628648	9.01838716	-5.0243219	0.03072758	-3.2514605	0.1050057	-1.7728614	0.29262777	6.7526594	11.7769813	8.52552081
A_55_P2084	NM_010917	Nid1	Mus musculu	chr13:13604	7.70480701	-3.1370516	0.11367196	-2.3708488	0.19333184	-0.7662028	0.58796296	6.40372219	9.54077383	7.169925
A_52_P2850	NM_198247	Sertad4	Mus musculu	chr1:1946713	9.58109931	2.18067424	4.53365385	1.25040775	2.37908654	0.93026649	1.90562797	10.6180796	8.43740531	9.68781306
A_55_P2137	NM_022420	Gprc5b	Mus musculu	chr7:1261172	9.96960013	-3.6338792	0.08055516	-1.7699314	0.29322268	-1.8639478	0.27472349	8.13699111	11.7708703	10.0009389
A_66_P1211	NM_011044	Pck1	Mus musculu	chr2:1729843	7.0427451	0.1260535	1.09130435	2.22059914	4.66086957	-2.0945456	0.23414179	6.38658105	6.26052755	8.48112669
A_52_P3999	NM_017377	B4galt2	Mus musculu	chr4:117546	9.87921193	2.41719238	5.34130543	2.06285619	4.17812656	0.35433619	1.27839723	10.8030548	8.3858624	10.4487186
A_55_P2007	NM_001080	Nanogpd	Mus musculu	chr6:1226635	7.59218672	-1.6290517	0.32330064	-2.4292562	0.18566114	0.80020447	1.74134791	7.31590431	8.94495603	6.51569984
A_55_P1970	NM_024124	Hdac9	Mus musculu	chr12:35056	8.37033798	-2.0544186	0.2407456	-0.7652696	0.5883434	-1.289149	0.40919232	7.25581542	9.31023407	8.54496443
A_52_P1294	NM_020510	Fzd2	Mus musculu	chr11:10246	7.90187594	0.8423864	1.79301356	-2.0980321	0.23357664	2.94041848	7.67633929	9.1628109	8.3204245	6.22239242
A_65_P0183	NM_0011135	Lima1	Mus musculu	chr15:996103	8.63715071	2.83539955	7.13740458	-0.2351132	0.84961832	3.07051277	8.40071878	10.6057881	7.7703886	7.53527538
A_51_P5110	NM_010246	Fzd9	Mus musculu	chr5:1357248	8.0651884	-3.1507421	0.11259837	-3.478283	0.08972893	0.32754086	1.25487256	7.12412131	10.2748634	6.79658045
A_51_P5174	NM_007639	Cd1d1	Mus musculu	chr3:8679985	9.13027177	0.97027355	1.95921205	-2.0500053	0.2414832	3.02027883	8.11324376	10.4604559	9.49018234	7.44017706
A_30_P0103	chr16:46384974-46391331		lincRNA:chr1	chr16:46388	8.31638475	-2.5498232	0.17077596	-1.3271731	0.3985484	-1.22265	0.42849491	7.05889369	9.60871685	8.28154371
A_55_P1973	NM_145967	Vstm2a	Mus musculu	chr11:16184	8.32404145	1.98435702	3.95686275	2.004707	4.0130719	-0.02035	0.98599349	8.97871046	6.99435344	8.99906044
A_51_P1857	NM_0011593	Casz1	Mus musculu	chr4:1483273	8.6002008	1.41729603	2.67084458	-1.8830256	0.27111454	3.30032164	9.85135135	10.17274	8.75544399	6.87241838
A_55_P2415	NM_026668	Lrriq4	Mus musculu	chr3:3057116	7.61158121	0.48610351	1.40065681	2.35263237	5.10755337	-1.8665289	0.27423244	7.15143943	6.66533592	9.01796829
A_51_P2680	NM_009255	Serpine2	Mus musculu	chr1:7979609	9.23431062	-2.2849734	0.20518919	-1.7829202	0.29059459	-0.5020531	0.70610119	8.30530178	10.5902751	8.80735492
A_55_P2000	NM_0010813	Ptprz1	Mus musculu	chr6:2300211	10.2901636	-1.8330812	0.28066456	-2.4657042	0.18102938	0.63262304	1.55038126	9.89001088	11.7230921	9.25738784
A_52_P6093	NM_030075	Klhdc8b	Mus musculu	chr9:1083506	10.921117	-1.8034845	0.28648181	0.3724749	1.29457173	-2.1759594	0.22129466	9.59463563	11.3981202	11.7705951
A_55_P2071	ENSMUST00000054395		Putative unch	chr5:1363653	8.72126504	-3.6285894	0.08085106	-2.0480631	0.24180851	-1.5805264	0.33435988	6.98489311	10.6134825	8.56541947
A_66_P1193	NM_177715	Kctd12	Mus musculu	chr14:103376	9.45238523	3.3158879	9.95822011	1.22546943	2.33831522	2.09041848	4.25871586	11.2544874	7.93859946	9.16406888
A_51_P3203	NM_011122	Plod1	Mus musculu	chr4:1472914	9.48633813	-2.4413317	0.18411362	-1.4006897	0.37874803	-1.040642	0.48611111	8.32568023	10.7670119	9.36632221

A_55_P1955	NM_183116	1110021L09R	Mus musculu	chr10:23547	8.15684086	1.58687716	3.00398406	-1.0309852	0.48937583	2.6178624	6.13839891	9.55842071	7.97154355	6.94055831
A_55_P2016	NM_029967	Adamtsl1	Mus musculu	chr4:860741	7.47211698	-1.5734943	0.33599362	-2.1287794	0.22865124	0.55528509	1.46945899	7.13271392	8.7062082	6.57742883
A_55_P2149	NM_001110	Foxp4	Mus musculu	chr17:48004	9.43490925	-2.1245754	0.22931849	-2.2157595	0.21527318	0.09118413	1.06524415	8.75711217	10.8816876	8.66592804
A_52_P5547	NM_183183	Gprin3	Mus musculu	chr6:593027	9.36388546	2.44144949	5.43187204	1.24179682	2.36492891	1.19965268	2.29684369	10.5775862	8.13613669	9.3779335
A_51_P4329	NM_009216	Sstr1	Mus musculu	chr12:59314	7.66067058	2.3869889	5.23064516	0.52153712	1.43548387	1.86545178	3.64382022	9.07815081	6.6911619	7.21269903
A_55_P2099	ENSMUST00000084758		olfactory rec	chr7:115004	6.99194274	0.18727857	1.13861386	2.56880271	5.93316832	-2.3815241	0.19190655	6.26052755	6.07324898	8.64205169
A_51_P2455	NM_028602	Tex19.1	Mus musculu	chr11:12100	7.61236515	-3.2208302	0.10725894	-1.7377473	0.29983749	-1.4830829	0.35772358	6.04439412	9.26522434	7.52747701
A_52_P2272	NM_178405	Atp1a2	Mus musculu	chr1:174208	8.19530024	-3.7009631	0.07689518	-2.2106836	0.21603192	-1.4902795	0.35594359	6.46488605	10.1658491	7.95516554
A_55_P2176	NM_001081	Lrrc7	Mus musculu	chr3:157819	9.61274126	2.17559878	4.51773227	1.51583372	2.85964036	0.65976506	1.57982533	10.5578625	8.38226376	9.89809748
A_66_P1199	ENSMUST00	LOC636126	Immunglobul	chr12:11710	6.98877413	0.19426882	1.14414414	2.14369346	4.41891892	-1.9494246	0.25891947	6.40372219	6.20945337	8.35314683
A_55_P2077	NM_001004	5730494M16	Mus musculu	chr18:25294	8.24804374	2.19516229	4.57941176	0.32079613	1.24901961	1.87436617	3.66640502	9.60455323	7.40939094	7.73018706
A_55_P2058	NM_026127	4833420G17	Mus musculu	chr13:12025	11.2711186	2.04141404	4.11648806	0.1399465	1.10186426	1.90146754	3.7359303	12.5854125	10.5439985	10.683945
A_55_P1963	NM_026825	Lrrc16a	Mus musculu	chr13:24198	9.83225904	2.06380366	4.18087138	0.71155034	1.63756292	1.35225332	2.55310579	10.9709447	8.90714104	9.61869138
A_55_P2054	XM_0014725	LOC1000488	PREDICTED:	chr9:119957	6.29242153	1.83215831	3.56069364	-0.5038909	0.70520231	2.3360492	5.04918033	7.68182404	5.84966573	5.34577484
A_30_P0102	chr13:34821764-34822230		lincRNA:chr1	chr13:34822	6.47735469	0.32750194	1.25483871	2.0310764	4.08709677	-1.7035745	0.30702447	6.01866384	5.6911619	7.72223831
A_55_P1992	NM_008155	Gpi1	Mus musculu	chr7:349872	12.7384782	-2.8939132	0.13453811	-0.5603998	0.67811423	-2.3335134	0.19840036	10.9960026	13.8899158	13.3295161
A_51_P3006	NM_0011635	Crb2	Mus musculu	chr2:376544	7.28963991	-2.1098923	0.23166432	-2.675226	0.15655853	0.56533369	1.47972973	6.77478706	8.88467932	6.20945337
A_55_P2064	XR_034463	LOC1000479	PREDICTED:	chr10:88872	7.29943632	-1.6422544	0.32035548	-2.3237609	0.19974609	0.68150644	1.60381356	6.97918699	8.62144141	6.29768055
A_30_P0102	chr17:3346621-3347209_F		lincRNA:chr1	chr17:33470	7.09614403	-2.0019091	0.2496694	-1.6084277	0.32795557	-0.3934814	0.76129032	6.29768055	8.29958963	6.6911619
A_55_P2093	NM_146131	Pbxip1	Mus musculu	chr3:892528	8.23772528	-2.2892058	0.20458811	-1.9246762	0.26339937	-0.3645296	0.77672209	7.35314683	9.6423526	7.71767642
A_30_P0102	chr5:144517700-14452607		lincRNA:chr5	chr5:144525	8.50235921	1.7928005	3.46486826	-0.4461431	0.73400251	2.2389436	4.72051282	9.84627391	8.05347341	7.60733031
A_51_P1586	NM_021427	Fam181b	Mus musculu	chr7:100230	11.173535	-3.1618381	0.11173568	-1.3511561	0.39197782	-1.8106821	0.28505613	9.51602828	12.6778664	11.3267104
A_52_P5895	NM_194060	Foxo6	Mus musculu	chr4:119939	10.9350891	2.00509682	4.01415638	0.84905199	1.80131687	1.15604483	2.22845655	11.988803	9.98370619	10.8327582
A_51_P1795	NM_010262	Gbx2	Mus musculu	chr1:918250	9.39084748	2.4321484	5.39696525	0.50586109	1.41997063	1.92628731	3.80075836	10.8436594	8.41151099	8.91737208
A_55_P2375	BG800260	2610027H17	2114-65 Mou	chr14:79123	7.89614354	0.48034989	1.39508197	-1.5499156	0.34153005	2.03026544	4.0848	8.73301532	8.25266543	6.70274988
A_55_P2109	NM_172743	Plekha7	Mus musculu	chr7:123267	7.14301546	-2.0508138	0.24134791	-2.0672393	0.23861566	0.01642555	1.01145038	6.46488605	8.51569984	6.4484605
A_55_P2039	NM_013560	Hspb1	Mus musculu	chr5:136365	8.32568249	-4.4302989	0.04638175	-2.9385767	0.13043684	-1.4917222	0.35558781	6.35167544	10.7819744	7.84339767
A_55_P1959	NM_146436	Olfr998	Mus musculu	chr2:854315	7.06269445	-0.0661771	0.95516569	2.0007029	4.00194932	-2.06688	0.23867511	6.35167544	6.41785251	8.41855541
A_30_P0102	chr6:127111250-12714292		lincRNA:chr6	chr6:127133	13.5424608	-2.6378015	0.1606729	-0.4799772	0.71698895	-2.1578243	0.22409396	11.9439189	14.5817204	14.1017432
A_66_P1181	NM_177408	Gabrg2	Mus musculu	chr11:41723	10.8168164	2.48056062	5.58114302	0.57244848	1.48704518	1.90811214	3.7531765	12.2797073	9.79914668	10.3715952
A_55_P2000	NM_172523	Slc18a2	Mus musculu	chr19:59370	9.31219783	-2.3874379	0.19112152	-1.3115862	0.4028777	-1.0758517	0.47439092	8.15776798	10.5452058	9.23361968
A_66_P1353	NM_008342	Igfbp2	Mus musculu	chr1:728988	11.7450821	-2.5710531	0.16828131	-1.4480428	0.36651831	-1.1230103	0.4591348	10.5137276	13.0847807	11.6367379
A_51_P1427	NM_172294	Sulf1	Mus musculu	chr1:128503	10.7480853	-4.7940814	0.03604439	-3.0038348	0.12466818	-1.7902466	0.28912263	8.5533093	13.3473907	10.3435559
A_51_P1370	NM_019588	Plce1	Mus musculu	chr19:38857	7.97598724	-2.0069639	0.24879615	-1.1252362	0.45842697	-0.8817278	0.54271709	7.01309	9.02005395	7.89481776
A_55_P2013	NM_010790	Melk	Mus musculu	chr4:443770	7.81842801	-1.2344881	0.42499327	-3.1304491	0.11419337	1.89596104	3.72169811	8.03891899	9.2734071	6.14295795
A_30_P0103	chr9:14460976-14488051_		lincRNA:chr9	chr9:144846	8.49248329	-1.4769571	0.35924572	-2.3210955	0.20011545	0.8441384	1.79519231	8.28154371	9.75850084	7.43740531
A_30_P0102	chr8:122712014-12273252		lincRNA:chr8	chr8:122728	9.26504947	-1.7635207	0.29452852	0.3191262	1.2475747	-2.0826469	0.23608087	7.98299357	9.74651432	10.0656405
A_51_P4481	NM_175519	Kctd8	Mus musculu	chr5:695013	7.74976121	3.47630544	11.1294118	0.54480537	1.45882353	2.93150006	7.62903226	9.88569637	6.40939094	6.95419631
A_30_P0102	chr9:114412295-11441838		lincRNA:chr9	chr9:114418	8.72818846	-2.0263308	0.24547861	-0.4739223	0.72000441	-1.5524084	0.34094042	7.53527538	9.56160618	9.08768383
A_55_P1973	XM_001476722		PREDICTED:	chr8:934154	7.92591653	2.85459867	7.23302264	0.02003113	1.01398136	2.83456754	7.13328956	9.82230526	6.9677066	6.98773773
A_51_P2331	NM_008520	Ltbp3	Mus musculu	chr19:57582	7.96826145	-2.2661673	0.20788142	-0.3749172	0.77114967	-1.8912501	0.26957337	6.58245565	8.84862294	8.47370575
A_55_P2127	XR_031313	LOC1000451	PREDICTED:	chr8:822029	10.3619432	-2.3587124	0.19496508	-1.6422305	0.32036079	-0.7164818	0.60857972	9.33687844	11.6955908	10.0533603
A_55_P1974	NM_001009	Pde1a	Mus musculu	chr2:797052	9.13196594	3.17085206	9.00578512	1.25755928	2.39090909	1.91329278	3.76667819	10.8266809	7.65582883	8.91338811
A_55_P2089	NM_010221	Fkbp10	Mus musculu	chr11:10028	7.86520862	-3.1955918	0.10915183	-2.8620571	0.13754188	-0.3335347	0.79358974	6.6888331	9.88442494	7.02236781

A_55_P2009(	NR_033553	1700048O20I	Mus musculu	chr9:121855	7.57405719	3.31127279	9.92641509	0.01624064	1.01132075	3.29503214	9.81529851	9.77615883	6.46488605	6.48112669
A_30_P0103	chr8:94841965-94881130_		lincRNA:chr8	chr8:9485037	6.79659808	-2.0375247	0.2435813	-1.524512	0.3475971	-0.5130127	0.70075758	5.94641896	7.98394365	6.45943162
A_51_P1631(	NM_175177	Bdh1	Mus musculu	chr16:314573	8.48043237	2.57998438	5.97933227	-0.2745484	0.82670906	2.85453278	7.23269231	10.2919381	7.71195371	7.43740531
A_55_P1958	NM_015748	Slit1	Mus musculu	chr19:416748	9.17827194	-2.7732745	0.146272	-1.7263896	0.3022073	-1.0468849	0.48401212	7.90488546	10.67816	8.95177039
A_55_P2051	NR_004446	Gm7035	Mus musculu	chr17:034111	10.8111183	-2.1169904	0.23052731	-1.2025167	0.43451663	-0.9144737	0.53053738	9.80063031	11.9176207	10.715104
A_30_P0103	chr2:33500594-33501874_		lincRNA:chr2	chr2:3350161	8.41266723	-3.8913067	0.0673907	-1.5463139	0.34238374	-2.3449928	0.19682798	6.33390074	10.2252074	8.67889352
A_55_P2067	XM_983385	Dppa5b	PREDICTED: M	chr9:7812009	7.29401371	-2.924449	0.13172043	-3.0218831	0.12311828	0.09743415	1.069869	6.35167544	9.27612441	6.25424129
A_51_P4029	NM_012008	Ddx3y	Mus musculu	chrY:598274-	6.58573419	2.30466889	4.94054054	-0.3867232	0.76486486	2.69139211	6.45936396	8.25108785	5.94641896	5.55969574
A_30_P0103	chr11:97275905-97310538		lincRNA:chr1	chr11:973097	8.80524104	-1.7839214	0.29039301	0.43692062	1.35371179	-2.220842	0.21451613	7.47031993	9.25424129	9.6911619
A_52_P5022	NM_009576	Zic4	Mus musculu	chr9:9128399	8.25651795	3.10379122	8.59674923	1.41447907	2.66563467	1.68931215	3.22502904	9.85421908	6.75042785	8.16490693
A_55_P2167	NM_153807	Acsf2	Mus musculu	chr11:944196	8.65618305	-3.1254213	0.11459204	-1.5816518	0.33409914	-1.5437695	0.34298812	7.09978612	10.2252074	8.6435556
A_55_P2026	NM_009062	Rgs4	Mus musculu	chr1:1716725	10.5830435	1.33597613	2.5244623	-0.972307	0.50969038	2.3082831	4.95293299	11.7977966	10.4618205	9.4895135
A_30_P0103	chr13:112337405-1123564		lincRNA:chr1	chr13:112351	6.94774603	2.3823554	5.21387283	0.91188551	1.88150289	1.47046989	2.77112135	8.23202112	5.84966573	6.76155123
A_66_P1263	NM_001110	Zfp703	Mus musculu	chr8:2809186	10.339064	-2.7795939	0.14563269	-2.097861	0.23360434	-0.6817328	0.62341603	9.18528843	11.9648823	9.86702128
A_55_P2124	NM_028283	Uaca	Mus musculu	chr9:6072811	7.49985588	-1.7459442	0.29813875	-2.5864279	0.16649746	0.8404837	1.79065041	7.19803571	8.94397991	6.357552
A_52_P3801	NM_178631	Ralyl	Mus musculu	chr3:1418138	9.6596018	2.10557245	4.30368488	2.76746097	6.80908513	-0.6618885	0.63205038	10.1408298	8.03525732	10.8027183
A_55_P2301(	AK036921	9930024M15	Mus musculu	chr10:415488	7.23749053	1.41551896	2.66755674	-0.5946256	0.66221629	2.01014456	4.02822581	8.37937837	6.96385941	6.36923381
A_66_P1051	NM_009738	Bche	Mus musculu	chr3:7343979	7.48371755	-2.0910719	0.23470624	-2.7702612	0.14657783	0.67918926	1.60123967	7.01309	9.1041619	6.33390074
A_55_P2029	XM_0014735	LOC1000448	PREDICTED: Mus musculus		9.78033069	-2.1727698	0.22178446	-0.7669426	0.58766156	-1.4058272	0.37740168	8.58746501	10.7602348	9.99329224
A_65_P1983	NM_013697	Ttr	Mus musculu	chr18:208285	8.33171926	-3.598921	0.08253095	0.07664409	1.05456213	-3.675565	0.07826087	5.9068906	9.50581155	9.58245565
A_55_P2180	XM_0014755	AA684185	PREDICTED: M	chr18:802104	7.6220588	0.00399639	1.00277393	2.13550117	4.39389736	-2.1315048	0.2282197	6.91288934	6.90889295	9.04439412
A_51_P2981(	NM_028813	Vit	Mus musculu	chr17:790264	7.73410497	-2.0578947	0.24016625	-2.1385746	0.22710405	0.08067995	1.05751634	7.07503339	9.13292808	6.99435344
A_55_P2028	NM_025274	Dppa5a	Mus musculu	chr9:7821492	8.98349657	-5.023244	0.03075055	-4.5907191	0.04150074	-0.4325249	0.74096386	7.16490693	12.1881509	7.59743185
A_66_P1016(	NM_008482	Lamb1-1	Mus musculu	chr12:320144	10.2345926	-3.5989884	0.08252709	-0.9485175	0.51816467	-2.650471	0.15926808	8.15143943	11.7504279	10.8019104
A_51_P3073	NM_027875	Syde1	Mus musculu	chr10:780474	8.85374853	-2.6085619	0.16396253	-2.0618115	0.23951509	-0.5467504	0.68456033	7.80197774	10.4105397	8.34872815
A_55_P1983	NM_0010122	Birc5	Mus musculu	chr11:117716	11.0727214	-1.8330211	0.28067625	-3.334397	0.09913945	1.50137587	2.83112583	10.962173	12.7951941	9.46079716
A_30_P0101	chr11:22510175-22522025		lincRNA:chr1	chr11:225186	7.41707668	0.33999639	1.26575342	2.37597568	5.19086758	-2.0359793	0.24384236	6.85174904	6.51175265	8.88772834
A_51_P2992	NM_053261	Impa2	Mus musculu	chr18:674783	6.63530396	-2.0550414	0.24064171	-1.9248426	0.26336898	-0.1301987	0.91370558	5.9068906	7.96193196	6.03708932
A_55_P2039	NM_007634	Ccnf	Mus musculu	chr17:243602	9.92275032	-1.4544676	0.36488971	-2.7068472	0.15316439	1.25237953	2.38234033	9.85538762	11.3098553	8.60300809
A_55_P2027	XM_0014785	LOC1000474	PREDICTED: M	chr5:7135233	8.56570872	2.70257036	6.50960659	0.46721026	1.38243367	2.2353601	4.70880212	10.2116855	7.50911518	7.97632544
A_55_P2040	NM_008885	Pmp22	Mus musculu	chr11:629729	7.26183423	-3.3199772	0.10013532	-3.1062191	0.11612744	-0.2137581	0.86228814	6.08392248	9.40389967	6.29768055
A_55_P2143(	NM_016966	Phgdh	Mus musculu	chr3:9811736	7.76083795	-1.9482228	0.25913525	-2.2520289	0.20992867	0.30380608	1.23439667	7.21269903	9.16092187	6.90889295
A_52_P6388	NM_0010252	Vegfa	Mus musculu	chr17:461544	11.71012	-2.2104456	0.21606757	0.33451829	1.26095631	-2.5449639	0.17135214	10.1249835	12.3354291	12.6699474
A_55_P2006(	NM_015754	Rbbp9	Mus musculu	chr2:1443682	8.09390023	3.63035238	12.3835443	-0.4708907	0.72151899	4.10124312	17.1631579	10.6710987	7.04074634	6.56985561
A_55_P1966	NM_010358	Gstm1	Mus musculu	chr3:1078156	8.71249288	-2.1133117	0.23111588	-2.3075524	0.20200286	0.19424066	1.14412181	8.07280253	10.1861142	7.87856187
A_52_P2516	NM_0010041	BC057022	Mus musculu	chr5:1150631	8.59004069	1.88728307	3.69937888	-0.3208084	0.80062112	2.20809149	4.62063615	9.95516554	8.06788247	7.74707405
A_55_P2183	NM_010296	Gli1	Mus musculu	chr10:126767	8.24019154	-3.0892986	0.11749746	-2.0132696	0.24771109	-1.0760289	0.47433265	6.85174904	9.94104761	7.92777796
A_55_P2408	NM_172671	Lgr4	Mus musculu	chr2:1098535	8.47124495	-2.1086492	0.231864	-1.636638	0.32160506	-0.4720112	0.72095882	7.6110248	9.71967402	8.08303603
A_52_P5693	NM_010022	Dbt	Mus musculu	chr3:1162520	8.40332856	1.13736982	2.19979613	-0.9868246	0.50458716	2.12419443	4.35959596	9.49051665	8.35314683	7.36632221
A_55_P2077	NM_052977	Adarb2	Mus musculu	chr13:875949	10.4460385	2.43020596	5.38970368	1.54460425	2.91724035	0.88560171	1.84753501	11.5513077	9.12110177	10.665706
A_55_P2068	XR_033308	Gm8483	PREDICTED: M	chr14:152802	13.7862433	-2.0342965	0.24412696	-0.0362314	0.97519906	-1.9980651	0.25033552	12.4421228	14.4764192	14.4401879
A_55_P1955	NM_009510	Ezr	Mus musculu	chr17:694266	8.3319691	-2.7268583	0.15105457	-3.1215758	0.11489789	0.39471751	1.31468531	7.55458885	10.2814471	7.15987134
A_55_P2056	NM_008342	Igfbp2	Mus musculu	chr1:7289867	10.8958844	-2.3551264	0.19545029	-1.4879216	0.35652581	-0.8672048	0.54820798	9.82177398	12.1769003	10.6889788
A_55_P1960	NM_013879	Cabp1	Mus musculu	chr5:1156228	8.30797318	-1.0360192	0.48767123	1.21378402	2.31945205	-2.2498032	0.21025278	7.21269903	8.24871825	9.46250227

A_55_P2043	ENSMUST00000107819		glyoxylate re	chr4:4500351	8.95825776	-2.163421	0.22322632	-1.075476	0.47451447	-1.0879449	0.47043101	7.87446912	10.0378901	8.96241406
A_30_P0102	chr6:54591416-54597566_		lincRNA:chr6:	chr6:5459414	10.324356	2.0346594	4.09725986	1.60109021	3.03372478	0.43356919	1.35057072	11.1470989	9.11243951	10.7135297
A_30_P0103	chr9:96664617-96683617_		lincRNA:chr9:	chr9:9667620	7.44337268	0.36195222	1.28516378	2.66428114	6.33911368	-2.3023289	0.20273556	6.79658045	6.43462823	9.09890937
A_52_P2983	NM_020278	Lgi1	Mus musculu	chr19:383807	10.1642182	1.32046862	2.49747219	2.32270588	5.00269633	-1.0022373	0.49922522	10.2702953	8.94982671	11.2725326
A_55_P2150	ENSMUST00000098575		Putative unch	chr9:7223312	6.78213506	0.26951844	1.20540541	2.23762986	4.71621622	-1.9681114	0.25558739	6.2159374	5.94641896	8.18404882
A_55_P2044	NM_025290	Rsph1	Mus musculu	chr17:313920	7.00645146	-1.7269549	0.30208891	-2.0983219	0.23352973	0.37136703	1.29357798	6.55458885	8.28154371	6.18322182
A_55_P2025	NM_016868	Hif3a	Mus musculu	chr7:1762087	7.62311991	-2.2029498	0.2171931	-1.9262813	0.26310648	-0.2766685	0.82549505	6.79658045	8.9995303	7.07324898
A_52_P4464	NM_0011631	Ly6g6f	Mus musculu	chr17:352180	6.29302599	0.22542011	1.16911765	2.14615684	4.42647059	-1.9207367	0.2641196	5.72792045	5.50250034	7.64865718
A_55_P2151	NM_146763	Olfr1406	Mus musculu	chr1:1751139	6.72526674	0.5161635	1.43014706	3.15213569	8.88970588	-2.6359722	0.16087676	6.01866384	5.50250034	8.65463603
A_55_P2076	NM_144944	Prokr2	Mus musculu	chr2:1321970	7.09771951	2.02280988	4.06374502	0.11060549	1.07968127	1.9122044	3.76383764	8.40939094	6.38658105	6.49718654
A_55_P2070	NM_009657	Aldoc	Mus musculu	chr11:781401	9.97137898	-4.0456597	0.06055292	-2.7688325	0.14672306	-1.2768272	0.41270213	8.19721669	12.2428764	9.47404389
A_52_P3614	NM_175513	Zfp804a	Mus musculu	chr2:8209976	6.46028795	2.65927707	6.31716418	0.27820672	1.21268657	2.38107035	5.20923077	8.14040376	5.48112669	5.75933341
A_66_P1054	NM_028894	Lonrf3	Mus musculu	chrX:3390675	7.66699475	-1.7950403	0.28816353	-2.1486773	0.22551929	0.35363695	1.27777778	7.18652697	8.98156728	6.83289001
A_51_P2954	NM_021605	Nek7	Mus musculu	chr1:1403818	8.24770528	-2.150382	0.22525297	-1.8027606	0.2866256	-0.3476214	0.78587874	7.4150375	9.56541947	7.76265887
A_30_P0101	chr7:82902248-82955248_		lincRNA:chr7:	chr7:8292554	8.18471328	-1.6808175	0.31190584	-2.3376882	0.19782707	0.6568707	1.57665904	7.84339767	9.5242152	7.18652697
A_30_P0102	chr4:54851872-54866706_		lincRNA:chr4:	chr4:5486559	7.31572742	-1.214963	0.43078412	-2.1208408	0.22991288	0.90587782	1.87368421	7.21269903	8.42766204	6.3068212
A_52_P3124	NM_0010798	Hoxb3	Mus musculu	chr11:962090	8.04380617	-3.8132621	0.0711367	-1.48382	0.35754086	-2.3294421	0.19896104	5.99623808	9.80950019	8.32568023
A_55_P2087	NM_011348	Sema3e	Mus musculu	chr5:1425659	7.40929464	1.6644775	3.16998828	-0.8909119	0.53927315	2.55538939	5.87826087	8.81591694	7.15143943	6.26052755
A_52_P3598	NM_198190	Ntf5	Mus musculu	chr7:5267175	7.04519589	-0.1914984	0.87569573	1.85954919	3.62894249	-2.0510476	0.24130879	6.29768055	6.48917896	8.34872815
A_55_P2223	ENSMUST00000104938		Novel proteir	chr11:901249	7.48922877	2.61129097	6.11050228	0.32113736	1.24931507	2.29015361	4.89108187	9.12304363	6.51175265	6.83289001
A_55_P2182	NM_013524	Fut7	Mus musculu	chr2:2528152	6.5434906	0.44288728	1.35932203	2.32875859	5.02372881	-1.8858713	0.2705803	6.06249593	5.61960864	7.94836723
A_55_P2085	NM_022654	Lrdd	Mus musculu	chr7:1486244	9.12226392	-2.3298361	0.19890672	0.04335302	1.03050608	-2.3731891	0.19301848	7.55458885	9.88442494	9.92777796
A_55_P2104	NM_009482	Utf1	Mus musculu	chr7:1471309	7.75219729	-1.7288424	0.30169393	-2.4840791	0.17873832	0.7552367	1.6879085	7.42766204	9.15650449	6.67242534
A_30_P0101	chr3:30077338-30080281_		lincRNA:chr3:	chr3:3007739	6.65371471	0.05857483	1.04143646	2.15791915	4.46270718	-2.0993443	0.23336428	5.97345821	5.91488339	8.07280253
A_55_P2007	ENSMUST000	Nanog	Nanog homeo	chr6:1226645	7.29476716	-1.5954216	0.33092551	-2.1046624	0.23250564	0.50924077	1.42330097	6.93270689	8.52812848	6.42346612
A_52_P2744	NM_183180	Tspan18	Mus musculu	chr2:9304204	9.09468592	-2.0810499	0.23634235	-1.6051827	0.32869407	-0.4758672	0.71903443	8.2423802	10.3234301	8.71824745
A_51_P4784	NM_133239	Crb1	Mus musculu	chr1:1410949	8.83824018	-3.0825753	0.1180463	-1.6788764	0.31232578	-1.4036989	0.37795887	7.34281546	10.4253908	8.74651432
A_66_P1365	ENSMUST00000082150		high mobility	chr6:1315583	10.4586231	0.08012363	1.05710863	2.18057648	4.53334665	-2.1004528	0.23318504	9.78517998	9.70505635	11.8856328
A_66_P1306	NM_053242	Foxp2	Mus musculu	chr6:1539182	8.07683082	2.3223438	5.00144092	1.34666062	2.54322767	0.97568318	1.96657224	9.17617315	6.85382935	8.20048997
A_51_P1524	NM_026166	Ikbip	Mus musculu	chr10:905612	8.3391627	-2.0252373	0.24566474	-1.6075909	0.32814584	-0.4176464	0.74864499	7.52486815	9.55010545	7.94251451
A_30_P0103	XIST		lincRNA:chrX:	chrX:1006560	8.76931176	5.52932298	46.1840563	-0.675706	0.62602579	6.20502898	73.7734082	12.6807624	7.15143943	6.47573343
A_55_P2219	NM_008265	Hoxa4	Mus musculu	chr6:5214034	7.110248	-2.5457377	0.17126026	-2.0124989	0.24784347	-0.5332388	0.6910017	6.08392248	8.62966019	6.61716132
A_55_P1992	NM_013755	Gyg	Mus musculu	chr3:2002209	8.63269028	-2.3703842	0.19339411	-2.1006758	0.23314901	-0.2697084	0.82948718	7.7526594	10.1230436	8.02236781
A_52_P8881	NM_029556	Clybl	Mus musculu	chr14:122775	7.87205747	-2.3928032	0.19041207	-1.8112457	0.28494479	-0.5815575	0.66824197	6.8806039	9.2734071	7.46216141
A_55_P2057	NM_025404	Arl4d	Mus musculu	chr11:101529	9.21168393	2.91882414	7.56229508	1.71311885	3.27868852	1.20570529	2.3065	10.5865271	7.66770293	9.38082178
A_55_P2038	NM_016956	Hbb-b2	Mus musculus hemoglobin,		6.67762064	2.43023991	5.38983051	0.74379609	1.67457627	1.68644382	3.21862348	8.04984855	5.61960864	6.36340473
A_52_P3003	NR_001570	Xist	Mus musculu	chrX:1006622	9.00315503	7.97510674	251.62069	-0.5284797	0.69328494	8.50358642	362.939791	14.4960528	6.52094601	5.99246633
A_55_P1960	NM_028300	Pih1d2	Mus musculu	chr9:5043299	7.23942156	0.87760712	1.83732535	-1.3104553	0.40319361	2.18806243	4.55693069	8.26131141	7.38370429	6.07324898
A_55_P1990	NM_029023	Scpep1	Mus musculu	chr11:887853	10.0849278	-3.2656113	0.10398077	-1.8018045	0.28681562	-1.4638068	0.36253526	8.50845506	11.7740664	9.97226185
A_55_P1999	NM_010031	Defa1	Mus musculus defensin, alp		6.8277105	0.55369104	1.46783626	2.43077043	5.39181287	-1.8770794	0.27223427	6.38658105	5.83289001	8.26366044
A_52_P3870	NM_028133	Egln3	Mus musculu	chr12:552810	8.42290936	-4.6453127	0.03995964	-2.1550797	0.22452069	-2.490233	0.17797753	6.04439412	10.6897068	8.53462712
A_30_P0101	chr3:126969590-12697262		lincRNA:chr3:	chr3:1269697	6.56147045	2.02175455	4.06077348	-0.0819934	0.94475138	2.10374792	4.29824561	7.93663794	5.91488339	5.83289001
A_55_P2013	XR_034534	Gm13937	PREDICTED: M	chr2:1100496	11.0669968	-3.2417009	0.10571845	-0.5434736	0.68611696	-2.6982273	0.15408226	9.08702077	12.3287217	11.7852481
A_55_P2146	NM_033652	Lmx1a	Mus musculu	chr1:1697788	9.38581879	-4.4703653	0.04511136	-2.7309872	0.15062288	-1.7393781	0.29949875	7.31590431	11.7862696	9.05528244

A_51_P1843(	NM_010087	Dtna	Mus musculu	chr18:237487	10.6353989	-2.6442865	0.15995229	-0.2069865	0.86634495	-2.4373	0.18462886	8.94153674	11.5858232	11.3788367
A_51_P4801	NM_029942	Prelid2	Mus musculu	chr18:42072(	8.54711848	-3.7919311	0.07219631	-2.6148196	0.16325289	-1.1771115	0.44223602	6.89077093	10.682702	8.06788247
A_52_P3578	NM_0010811	Gli2	Mus musculu	chr1:1207313	7.41716296	-2.8296033	0.14067098	-2.3554307	0.19540906	-0.4741726	0.71987952	6.31590431	9.14550764	6.79007693
A_51_P1594	NM_009252	Serpina3n	Mus musculu	chr12:105651	6.8294945	-2.8996518	0.13400402	-2.6940676	0.15452716	-0.2055841	0.8671875	5.79441587	8.69406764	6
A_55_P2075	NM_177066	Tnni3k	Mus musculu	chr3:1544493	6.83547101	1.75582462	3.37719298	-0.5029691	0.70565302	2.25879375	4.7859116	8.17367714	6.41785251	5.91488339
A_52_P8968	XM_0014746	Gm14470	PREDICTED: N	chrX:102584(	10.5316859	-3.597302	0.08262361	-0.8425451	0.55765893	-2.754757	0.14816155	8.41433289	12.0116349	11.1690899
A_30_P0102(	chr9:14460976-14488051_		lincRNA:chr9:	chr9:1448478	8.49636097	-1.3452792	0.3935778	-2.4051808	0.18878541	1.05990159	2.08478931	8.40123509	9.74651432	7.34133349
A_55_P2023	NM_029606	Ccdc46	Mus musculu	chr11:108721	8.9438451	2.64931462	6.27369165	0.54040895	1.45438472	2.10890567	4.31363968	10.5299185	7.8806039	8.42101286
A_52_P5868	NM_010317	Gng4	Mus musculu	chr13:139193	10.8685869	-1.781353	0.29091045	1.00683889	2.00950322	-2.7881919	0.14476734	9.34540525	11.1267582	12.1335971
A_52_P4188	NM_029971	Pmch	Mus musculu	chr10:87555(	8.97727066	-2.3504792	0.19608089	-1.8528644	0.27684217	-0.4976148	0.7082768	8.027906	10.3783852	8.52552081
A_51_P2149	NM_145492	Zfp521	Mus musculu	chr18:138467	8.4030765	0.9841014	1.97808086	-1.0305381	0.48952752	2.01463953	4.04079602	9.40265681	8.41855541	7.38801729
A_30_P0101	chr7:86680928-86700632_		lincRNA:chr7:	chr7:8670016	8.88916428	1.28425327	2.43555957	-1.1688837	0.44476534	2.45313699	5.47605519	10.134961	8.85070776	7.68182404
A_55_P2093	NM_026205	Rnf151	Mus musculu	chr17:248532	7.62850199	0.44745898	1.36363636	2.16671049	4.48998459	-1.7192515	0.30370625	7.20457114	6.75711217	8.92382266
A_55_P2110	NM_007585	Anxa2	Mus musculu	chr9:6933953	9.00449254	-4.885125	0.03384004	-2.8995089	0.1340173	-1.9856161	0.25250501	6.71424552	11.5993705	8.6998616
A_52_P3566	NM_013665	Shox2	Mus musculu	chr3:6677981	9.08612995	-5.1810863	0.02756371	-2.1588091	0.22394105	-3.0222772	0.12308465	6.35167544	11.5327618	9.37395266
A_55_P1964	NM_177785	BC049635	Mus musculu	chr4:4288144	8.66282125	-2.0978685	0.23360313	0.42036277	1.33826402	-2.5182313	0.17455683	7.12412131	9.22198983	9.6423526
A_55_P2105	NM_010052	Dlk1	Mus musculu	chr12:110698	10.3744569	-2.5962115	0.16537219	-0.8449738	0.5567209	-1.7512376	0.29704684	8.92530717	11.5215187	10.6765448
A_66_P1345	NM_028390	Anln	Mus musculu	chr9:2213674	7.34555421	-1.6504156	0.31854839	-2.7989181	0.14369501	1.14850252	2.21683673	7.17824987	8.82866543	6.02974734
A_55_P2024	NM_0010395	Myo6	Mus musculu	chr9:8015556	8.31809701	-2.514391	0.1750221	-0.6297381	0.64629372	-1.8846529	0.27080891	6.85174904	9.36614004	8.73640193
A_52_P1095	NM_008635	Mtap7	Mus musculu	chr10:200008	7.87259147	-1.7729027	0.29261939	-2.1178454	0.23039074	0.34494266	1.2701005	7.39660478	9.16950749	7.05166212
A_51_P3711	NM_013863	Bag3	Mus musculu	chr7:1356902	9.24164047	-2.1304212	0.22839117	-0.2805712	0.82326498	-1.84985	0.27742121	7.91488339	10.0453046	9.76473339
A_66_P1007	NM_009216	Sstr1	Mus musculu	chr12:593166	8.52833944	2.09073193	4.25964126	0.88068992	1.84125561	1.21004202	2.31344374	9.62859743	7.53786549	8.41855541
A_52_P3995	NM_181589	Ckap2l	Mus musculu	chr2:1290947	7.52114495	-0.6636615	0.63127413	-2.0684411	0.23841699	1.40477959	2.64777328	7.76818432	8.43184579	6.36340473
A_55_P1952	NM_172301	Ccnb1	Mus musculu	chr13:101551	10.0818017	-2.0210537	0.24637816	-3.1620418	0.11171991	1.14098803	2.20532003	9.78844646	11.8095002	8.64745843
A_55_P2118	NM_010514	Igf2	Mus musculu	chr7:1498397	10.1374045	-7.4516482	0.00571255	-2.6242654	0.1621875	-4.8273828	0.03522192	6.04439412	13.4960423	10.8717769
A_52_P2037	NM_0011050	Vmn2r61	Mus musculu	chr7:4955565	6.46021321	0.62119865	1.53815261	2.63432267	6.20883534	-2.013124	0.24773609	5.99623808	5.37503943	8.0093621
A_55_P2148	XM_0014786	LOC1000475	PREDICTED: N	chr4:9087831	9.47391576	2.06195999	4.17553191	1.07261338	2.10323985	0.98934661	1.98528567	10.491018	8.42905797	9.50167135
A_55_P2230	NM_199307	Ece1	Mus musculu	chr4:1375196	10.7638982	-2.0043997	0.24923875	-0.2917093	0.81693357	-1.7126904	0.3050906	9.52486815	11.5292679	11.2375585
A_55_P2006	XM_0014793	LOC1000481	PREDICTED: N	chr7:8739161	7.4253268	-0.1346732	0.91087613	2.05347888	4.1510574	-2.1881521	0.21943231	6.65105169	6.78572491	8.83920379
A_52_P1936	NM_181422	Pkd2l1	Mus musculu	chr19:442226	9.05840994	-3.8051879	0.07153594	-1.6602461	0.31638518	-2.1449418	0.22610397	7.07503339	10.8802212	9.21997517
A_30_P0102	chr18:6144775-6202105_R		lincRNA:chr1	chr18:620101	6.83724779	1.897279	3.7250996	-0.5452788	0.68525896	2.4425578	5.43604651	8.28386005	6.38658105	5.84130225
A_55_P2159	NM_0011665	Tead1	Mus musculu	chr7:1200434	11.709904	-2.1423897	0.2265043	-2.0007167	0.24987584	-0.141673	0.9064674	10.9485497	13.0909394	11.0902227
A_55_P2091	NM_146011	Arhgap9	Mus musculu	chr10:126766	6.78127406	-1.4601669	0.36345109	-2.0118093	0.24796196	0.55164243	1.46575342	6.47843258	7.93859946	5.92679015
A_30_P0101	chr17:41231055-41249764		lincRNA:chr1	chr17:41239(	12.1960395	-3.131156	0.11413744	-0.342578	0.78863083	-2.788578	0.14472861	10.2227949	13.3539509	13.0113729
A_55_P2235	AK075572	0610009E02F	Mus musculu	chr2:2631478	9.26180995	-2.6015201	0.16476479	-4.0624819	0.05985095	1.46096179	2.75291829	8.88162384	11.483144	7.42066205
A_52_P6503	NM_011499	Strap	Mus musculu	chr6:1376998	10.0689874	1.979667	3.94402036	-0.2512686	0.8401573	2.23093563	4.69438326	11.4725216	9.49285462	9.24158599
A_55_P1988	NM_0011629	Mdm1	Mus musculu	chr10:117605	7.8508901	0.90775086	1.87611838	-1.11465	0.46180317	2.02240089	4.06259314	8.82760735	7.91985649	6.80520646
A_55_P2055	NM_0011360	Enpp2	Mus musculu	chr15:546709	8.81284448	-3.3352784	0.09907889	-3.2950252	0.10188226	-0.0402532	0.97248428	7.68766728	11.0229457	7.72792045
A_55_P2056	NM_009387	Tk1	Mus musculu	chr11:117676	7.69597151	-1.4330482	0.37034759	-2.7241533	0.15133805	1.29110517	2.44715447	7.64865718	9.08170534	6.357552
A_52_P4329	NM_0011052	Pcdh19	Mus musculu	chrX:1301224	10.4391286	2.67699476	6.39522342	2.36905453	5.16602465	0.30794023	1.23793901	11.4341069	8.75711217	11.1261667
A_51_P2870	NM_009825	Serpinh1	Mus musculu	chr7:106494(	10.9111211	-2.4520006	0.18275711	-0.9019801	0.53515173	-1.5500205	0.34150522	9.57711407	12.0291146	11.1271346
A_55_P2042	XR_031240	Gm5250	PREDICTED: N	chr1:1305253	8.12370202	-3.2110771	0.1079865	-3.5608231	0.08473941	0.34974604	1.27433628	7.169925	10.3810021	6.82017896
A_55_P1961	NM_010329	Pdpn	Mus musculu	chr4:1428573	11.3332906	-4.548972	0.04271919	-2.1817902	0.2204021	-2.3671818	0.19382388	9.027906	13.576878	11.3950878
A_66_P1190	ENSMUST00000071101		Putative uncl	chr6:1281379	7.81012637	-2.3618945	0.19453552	-1.9657227	0.25601093	-0.3961718	0.75987193	6.89077093	9.25266543	7.28694274

A_55_P20831	NM_0010138	Maml2	Mus musculu	chr9:1342419	8.44257378	-2.238747	0.21187026	-2.1811698	0.22049689	-0.0575773	0.96087637	7.67713234	9.91587938	7.73470962
A_51_P40407	NM_020510	Fzd2	Mus musculu	chr11:102467	8.89898388	-0.5704657	0.67339939	-2.0503512	0.2414253	1.47988552	2.78926598	9.20212382	9.7725895	7.72223831
A_52_P21194	NM_011286	Rph3a	Mus musculu	chr5:1213910	10.5293881	-1.1324073	0.45615393	1.04770193	2.06723432	-2.1801093	0.22065903	9.4252159	10.5576232	11.6053252
A_30_P01029	chr9:14460976-14488051_	lincRNA:chr9:	chr9:1448443	8.81944049	-2.245729	0.21084738	-2.9558774	0.12888199	0.71014846	1.63597246	8.30758032	10.5533093	7.59743185
A_66_P13332	NM_0010252	Tpd52	Mus musculu	chr3:8929759	10.4666222	-2.5057358	0.17607527	-1.6776583	0.31258961	-0.8280775	0.56327935	9.3553511	11.8610869	10.1834286
A_52_P66042	NM_008600	Mip	Mus musculu	chr10:127668	7.37057733	0.70391563	1.62891986	-1.3320169	0.39721254	2.03593254	4.10087719	8.28386005	7.57994443	6.24792751
A_55_P19865	NM_023061	Mcam	Mus musculu	chr9:4394938	6.80351307	-2.0365746	0.24374177	-1.5018669	0.35309618	-0.5347077	0.69029851	5.94641896	7.98299357	6.48112669
A_30_P01027	chr12:50490638-50508093	lincRNA:chr12	chr12:505079	8.35543022	2.33518419	5.04615385	0.99682575	1.9956044	1.33835844	2.52863436	9.57994443	7.24476023	8.24158599
A_51_P48791	NM_177158	Rinl	Mus musculu	chr7:2958373	8.34030902	0.27297459	1.20829656	2.06504353	4.18446602	-1.7920689	0.28875765	7.83394423	7.56096965	9.62601318
A_30_P01020	chr16:94055900-94120575	lincRNA:chr16	chr16:940859	10.7927913	-1.1105986	0.46310185	1.04762886	2.06712963	-2.1582274	0.22403135	9.70318262	10.8137812	11.8614101
A_51_P23580	NM_007564	Zfp36l1	Mus musculu	chr12:812090	10.2401444	-3.8522248	0.06924123	-3.0818662	0.11810433	-0.7703586	0.58627174	8.69928325	12.551508	9.46964182
A_52_P48929	NM_009621	Adamts1	Mus musculu	chr16:857951	8.83331812	-2.8005952	0.14352806	-2.2198607	0.21466208	-0.5807345	0.66862327	7.7062082	10.5068034	8.28694274
A_55_P21061	NM_021790	Cenpk	Mus musculu	chr13:105039	7.40362834	0.12264697	1.08873057	-1.9342456	0.26165803	2.05689253	4.16089109	8.13014151	8.00749454	6.07324898
A_30_P01028	chr9:121845920-12185812	lincRNA:chr9:	chr9:1218510	7.52227901	2.96776148	7.82321429	-0.033886	0.97678571	3.00164748	8.00914077	9.512082	6.54432052	6.51043452
A_51_P37937	NM_145381	Lactb2	Mus musculu	chr1:1361624	8.12505156	-1.5621864	0.33863749	-2.2288635	0.2133267	0.66667715	1.58741259	7.82654849	9.38873487	7.15987134
A_30_P01026	chr17:32075308-32075792	lincRNA:chr17	chr17:320755	7.13597066	0.62350089	1.54060914	2.67314314	6.37817259	-2.0496422	0.24154397	6.66059021	6.03708932	8.71023246
A_51_P36500	NM_027884	Tns1	Mus musculu	chr1:7395690	9.3678354	-2.1309365	0.22830961	-1.9840554	0.25277832	-0.1468811	0.90320093	8.60856286	10.7394994	8.75544399
A_55_P20179	NM_013492	Clu	Mus musculu	chr14:666003	9.2268342	-2.2911052	0.20431894	-0.9059323	0.53368771	-1.3851729	0.38284363	8.00140819	10.2925134	9.38658105
A_30_P01031	chr6:127111250-12714292	lincRNA:chr6:	chr6:1271332	11.4947911	-2.4653744	0.18107078	-0.6142562	0.65326662	-1.8511182	0.27717745	10.0559602	12.5213346	11.9070784
A_51_P38953	NM_054053	Gpr98	Mus musculu	chr13:812343	7.87716624	-2.9738584	0.12728564	-3.0188186	0.12338008	0.04496014	1.03165468	6.90086681	9.87472526	6.85590667
A_55_P20675	NM_010446	Foxa2	Mus musculu	chr2:1478686	7.7672304	-4.7199847	0.03794399	-3.2534472	0.1048612	-1.4665375	0.36184971	5.70505635	10.425041	7.17159382
A_30_P01021	chr17:39960567-39992817	lincRNA:chr17	chr17:399821	14.4509476	-0.2038868	0.86820832	2.57546026	5.96061117	-2.7793471	0.1456576	13.4565362	13.6604231	16.2358833
A_55_P20875	NM_020333	Slc12a5	Mus musculu	chr2:1648048	11.2675896	-0.6380841	0.64256569	1.45284499	2.73747348	-2.0909291	0.23472947	10.3579185	10.9960026	12.4488476
A_30_P01022	chr7:28592214-28605984_	lincRNA:chr7:	chr7:2860592	6.98389109	1.25540061	2.38733432	-0.7706431	0.58615611	2.02604375	4.07286432	8.07770587	6.82230526	6.05166212
A_52_P22557	NM_020606	Parva	Mus musculu	chr7:1197346	8.71625296	-1.8431116	0.27872	-2.0821635	0.23616	0.2390519	1.1802168	8.18156641	10.024678	7.94251451
A_52_P71624	NM_146491	Olfr1410	Mus musculu	chr1:9450505	12.017587	1.51267575	2.85338763	2.09713898	4.27860052	-0.5844632	0.66689742	12.3269912	10.8143154	12.9114544
A_51_P25869	NM_009136	Scrg1	Mus musculu	chr8:5995625	6.82632678	-1.73236	0.30095923	-2.1454829	0.22601918	0.41312284	1.33156499	6.38658105	8.11894107	5.97345821
A_30_P01029	chr18:75132417-75141201	lincRNA:chr18	chr18:751397	7.01477268	0.6627708	1.5831202	2.30336033	4.93606138	-1.6405895	0.32072539	6.6888331	6.0260623	8.32942263
A_51_P29523	NM_172784	Lrp11	Mus musculu	chr10:734501	11.4711815	2.01709395	4.04767638	1.09152358	2.13098965	0.92557036	1.89943503	12.4520696	10.4349757	11.5264992
A_51_P15704	NM_010217	Ctgf	Mus musculu	chr10:243182	8.64250428	-2.9819284	0.12657563	-0.775012	0.58438375	-2.2069164	0.21659676	6.91288934	9.89481776	9.11980574
A_51_P48315	NM_177157	Gchfr	Mus musculu	chr2:1189980	8.93958489	-3.7873952	0.07242366	-1.8507948	0.2772396	-1.9366004	0.26123128	7.03158634	10.8189815	8.96818677
A_55_P22018	NM_0011635	Naf1	Mus musculu	chr8:6941439	10.4338055	-1.7064686	0.30640917	-2.1312002	0.22826788	0.4247316	1.34232275	10.0065598	11.7130284	9.58182825
A_55_P21715	NM_153138	Wipf1	Mus musculu	chr2:7327028	7.50923354	-1.6968907	0.30845017	-2.1178481	0.2303903	0.42095747	1.33881579	7.08392248	8.78081313	6.66296501
A_55_P23924	NM_0011625	9330159F19R	Mus musculu	chr10:289481	10.4173777	2.38157177	5.21104161	1.09250848	2.13244493	1.28906329	2.44369341	11.6409227	9.25935096	10.3518594
A_55_P20003	NM_009700	Aqp4	Mus musculu	chr18:155514	7.4254676	-3.4897043	0.08902138	-3.222788	0.10711349	-0.2669163	0.83109405	6.17326071	9.66296501	6.44017706
A_55_P20647	NR_024257	4930412O13R	Mus musculu	chr2:9808308	8.89198418	-5.2734925	0.02585357	-2.5188446	0.17448264	-2.7546479	0.14817276	6.2159374	11.4894299	8.97058527
A_66_P12485	NM_0010244	Diras2	Mus musculu	chr13:525998	10.3748003	-4.0716595	0.05947143	-2.5797919	0.16726507	-1.4918676	0.35555197	8.52029128	12.5919508	10.0121589
A_55_P20132	NM_009789	S100g	Mus musculu	chrX:1593999	7.93020952	-3.1257271	0.11456775	-0.4495398	0.7322764	-2.6761873	0.15645425	5.99623808	9.12196514	8.67242534
A_30_P01019	chr1:92412246-92423471_	lincRNA:chr1:	chr1:9241930	7.57643973	0.48360179	1.39823009	2.57428373	5.95575221	-2.0906819	0.23476969	7.04074634	6.55714456	9.13142829
A_55_P20626	NM_025952	Magt1	Mus musculu	chrX:1031748	10.7697677	-2.1700356	0.22220519	-1.7791529	0.29135443	-0.3908827	0.76266281	9.91612827	12.0861639	10.307011
A_55_P20121	NM_026470	Spata6	Mus musculu	chr4:1114474	7.69853067	-2.4838158	0.17877095	-2.0829363	0.23603352	-0.4008794	0.75739645	6.73696559	9.22078137	7.13784503
A_55_P20248	NM_021281	Ctss	Mus musculu	chr3:9536024	7.15765746	3.14048122	8.81818182	1.95419631	3.875	1.18628491	2.27565982	8.59991284	5.45943162	7.41362793
A_55_P20947	NM_011437	Sox10	Mus musculu	chr15:789855	8.0002356	-3.2764264	0.1032042	-3.0126829	0.12390593	-0.2637435	0.83292383	6.82017896	10.0966054	7.08392248
A_55_P21294	NR_033594	Gm5712	Mus musculu	chr3:1291374	6.9994933	-2.3468028	0.1965812	-2.020699	0.24643875	-0.3261038	0.79768786	6.10852446	8.45532722	6.43462823

A_55_P2118	XR_032614	Gm5987	PREDICTED: M	chr5:1066452	9.51997635	-3.0086976	0.12424868	-0.9116146	0.53158981	-2.0970829	0.23373036	7.81804952	10.8267471	9.91513245
A_51_P1642	NM_019731	Nme4	Mus musculu	chr17:26228	7.45537064	-1.5075184	0.35171569	-2.1436457	0.22630719	0.63612726	1.55415162	7.16490693	8.67242534	6.52877967
A_55_P2171	NM_008714	Notch1	Mus musculu	chr2:263194	9.72510979	-2.0881666	0.23517937	-1.5578119	0.33966585	-0.5303546	0.69238452	8.8522694	10.940436	9.38262403
A_51_P4407	NM_009886	Celsr1	Mus musculu	chr15:857294	8.5415618	-2.2112233	0.21595112	-2.2166045	0.21514713	0.00538119	1.00373692	7.80628109	10.0175044	7.8008999
A_55_P2016	XM_204772	Gm5068	PREDICTED: M	chr12:75062	7.54072289	-3.0871206	0.11767497	-2.659774	0.15824437	-0.4273466	0.74362819	6.36923381	9.45635442	6.79658045
A_52_P2236	NM_016967	Olig2	Mus musculu	chr16:912284	10.1496929	-2.0402302	0.24312494	-2.833817	0.14026073	0.79358677	1.73337856	9.73414508	11.7743753	8.94055831
A_52_P4110	NM_144553	Dlgap5	Mus musculu	chr14:48011	7.66545436	-1.2747606	0.41329372	-2.1589843	0.22391385	0.88422369	1.84577114	7.53527538	8.81003601	6.65105169
A_30_P0102	chr15:38639259-38639546	lincRNA:chr1		chr15:38639	8.90173163	-0.2508983	0.84037301	2.21468412	4.64179923	-2.4655824	0.18104467	7.99623808	8.24713635	10.4618205
A_52_P3130	NM_008119	Gip	Mus musculu	chr11:958867	7.19215277	0.35195466	1.27628866	2.21386835	4.63917526	-1.8619137	0.27511111	6.6888331	6.33687844	8.55074679
A_55_P2055	NM_173740	Maoa	Mus musculu	chrX:1626478	9.34171195	-2.4307026	0.1854751	-1.2905875	0.40878452	-1.140115	0.4537234	8.15143943	10.582142	9.29155445
A_55_P2004	NM_152229	Nr2e1	Mus musculu	chr10:422818	8.10895324	3.29722933	9.8302583	1.53807078	2.90405904	1.75915854	3.38500635	9.79441587	6.49718654	8.03525732
A_51_P3783	NR_033641	4933436C20R	Mus musculu	chr8:9486876	7.01477724	-2.0066484	0.24885057	-1.4887472	0.35632184	-0.5179012	0.6983871	6.17326071	8.17990909	6.6911619
A_55_P2145	NM_026531	Aen	Mus musculu	chr7:860532	11.8942611	-2.3144004	0.20104629	-0.6498856	0.63733086	-1.6645148	0.31545042	10.5679561	12.8823565	12.2324709
A_55_P1968	NM_028733	Pacsin3	Mus musculu	chr2:911047	8.66302184	-2.223145	0.21417397	-1.9590571	0.2571965	-0.2640879	0.83272506	7.83394423	10.0570892	8.09803208
A_55_P2077	NM_172471	Itih5	Mus musculu	chr2:101779	6.47643743	-1.9407608	0.26047904	-2.0261523	0.24550898	0.08539149	1.06097561	5.857981	7.79874179	5.7725895
A_30_P0103	chr2:59235155-59242518_	lincRNA:chr2		chr2:592371	11.715891	-3.1485188	0.11277203	-0.4381715	0.73806948	-2.7103473	0.15279325	9.76293564	12.9114544	12.473283
A_51_P4728	NM_145144	Aif1l	Mus musculu	chr2:318288	10.3549961	-0.1372502	0.90925052	-2.2588926	0.20893229	2.12164234	4.35189076	11.0164601	11.1537104	8.89481776
A_55_P2062	NM_010358	Gstm1	Mus musculu	chr5:117082	7.39125851	-1.6061053	0.32848392	-2.5180492	0.17457887	0.91194382	1.88157895	7.15987134	8.76597668	6.24792751
A_30_P0102	chr4:149445201-14944590	lincRNA:chr4		chr4:149445	7.40212829	-2.7138142	0.15242652	-1.8690556	0.27375256	-0.8447585	0.556804	6.2159374	8.92975155	7.06069593
A_55_P1965	NM_001081	Xlr3b	Mus musculu	chrX:704480	7.82317363	1.6733594	3.18956438	-0.5347413	0.69028243	2.20810073	4.62066574	9.11699368	7.44363428	6.90889295
A_55_P2048	NM_007659	Cdk1	Mus musculu	chr10:688011	7.69668274	-0.1077633	0.92802573	-2.8878734	0.13510253	2.78011009	6.86904762	8.58746501	8.69522829	5.80735492
A_51_P4967	NM_019448	Dnmt3l	Mus musculu	chr10:77526	7.20362381	-1.7326004	0.30090909	-2.2115041	0.21590909	0.4789037	1.39368421	6.78572491	8.51832531	6.3068212
A_30_P0103	chr8:67542714-67561694_	lincRNA:chr8		chr8:675528	7.46891554	-3.2216704	0.10719649	-2.8556498	0.13815409	-0.3660207	0.77591973	6.27301849	9.49468894	6.63903917
A_55_P2158	ENSMUST00000097340		Mus musculu	chr17:35109	6.95096906	-1.6393796	0.32099448	-2.2181476	0.21491713	0.57876801	1.49357326	6.59743185	8.23681148	6.01866384
A_55_P2007	NM_001013	Akr1c19	Mus musculu	chr13:42475	6.42350073	2.54088143	5.81944444	-0.0252668	0.98263889	2.56614819	5.92226148	8.12584393	5.5849625	5.55969574
A_30_P0103	chr2:33496712-33501183_	lincRNA:chr2		chr2:335009	8.15888213	-4.3662217	0.04848823	-1.82974	0.28131531	-2.5364817	0.17236256	5.857981	10.2242027	8.39446269
A_51_P4808	NM_021453	Pga5	Mus musculu	chr19:10744	8.64853788	-1.9232341	0.26366279	0.37915669	1.3005814	-2.3023908	0.20272687	7.23999624	9.16323035	9.54238704
A_55_P1988	NM_009415	Tpi1	Mus musculu	chr6:124761	12.8984743	-2.6983833	0.15406561	-0.76602	0.58803749	-1.9323633	0.26199963	11.3548922	14.0532754	13.2872555
A_51_P5136	NM_021530	Slc4a8	Mus musculu	chr15:10065	11.4737781	2.09125013	4.26117154	1.39200229	2.62442668	0.69924784	1.62365806	12.403944	10.3126939	11.7046962
A_55_P2205	AK162965	2610507I01R	Mus musculu	chr11:59011	7.62685317	2.17433369	4.51377246	-0.6557838	0.63473054	2.83011753	7.11132075	9.29500358	7.12066989	6.46488605
A_55_P2004	NM_001040	Tacc3	Mus musculu	chr5:340146	9.22157572	-1.7352497	0.30035702	-2.0970692	0.23373258	0.36181953	1.28504558	8.76376565	10.4990154	8.40194612
A_51_P4489	NM_033610	Sncb	Mus musculu	chr13:54861	10.0535378	-1.7624106	0.29475524	1.26672889	2.40615385	-3.0291395	0.12250058	8.45635442	10.218765	11.4854939
A_55_P1959	NM_012055	Asns	Mus musculu	chr6:762522	10.2507724	-2.2708684	0.20720512	-0.8807313	0.54309206	-1.3901371	0.38152855	9.03043724	11.3013057	10.4205743
A_55_P2006	ENSMUST00000103490		Mus musculu	chr12:11569	6.89522327	0.29206345	1.22439024	2.11005355	4.31707317	-1.8179901	0.28361582	6.38658105	6.0945176	8.20457114
A_55_P2083	NM_007545	Hrk	Mus musculu	chr5:118639	9.6951037	-2.4705918	0.18041712	-1.3193927	0.40070358	-1.1511991	0.45025084	8.48784003	10.9584319	9.63903917
A_55_P1971	XR_031215	LOC242317	PREDICTED: M	chr4:103664	8.44185898	-2.074728	0.23738027	-0.5913254	0.66373285	-1.4834026	0.35764431	7.25581542	9.33054346	8.73921805
A_55_P2112	NM_011648	Tshr	Mus musculu	chr12:92778	7.15761656	2.6596013	6.31858407	0.107599	1.07743363	2.5520023	5.86447639	8.89481776	6.23521646	6.34281546
A_55_P2276	AK157781		Mus musculu	chr15:13101	7.79237694	-1.8454762	0.27826354	-2.0338103	0.24420923	0.18833412	1.13944724	7.23999624	9.08547246	7.05166212
A_51_P1519	NM_145584	Spon1	Mus musculu	chr7:121184	11.6393203	-4.0057778	0.0622502	-2.3732591	0.19300912	-1.6325187	0.32252465	9.75988818	13.765666	11.3924069
A_55_P2173	NM_009104	Rrm2	Mus musculu	chr12:25398	9.49662475	-0.7593013	0.59078238	-3.1769127	0.11057424	2.41761144	5.34285714	10.0493948	10.8086961	7.63178336
A_30_P0103	chr2:28151215-28151611_	lincRNA:chr2		chr2:281515	7.3068677	0.46097378	1.37647059	2.2314565	4.69607843	-1.7704827	0.29311065	6.87036472	6.40939094	8.64084744
A_30_P0102	chr17:33995525-34028025	lincRNA:chr1		chr17:34023	8.47018479	-1.6522422	0.31814532	-2.0298439	0.24488157	0.37760169	1.29918033	8.04530462	9.69754681	7.66770293
A_55_P1983	NM_025557	Pcp4l1	Mus musculu	chr1:173103	7.69459341	-2.0108106	0.24813367	-1.5242032	0.34767153	-0.4866074	0.71370143	6.86212073	8.87293134	7.34872815
A_55_P2168	NM_028030	Rbpms2	Mus musculu	chr9:655082	9.0459115	-2.6539772	0.15888147	-3.8224602	0.0706846	1.16848303	2.24775225	8.55074679	11.204724	7.38226376

A_51_P39657	NM_011961	Plod2	Mus musculu	chr9:9250248	8.88939193	-2.8710285	0.13668923	-1.3860972	0.38259841	-1.4849313	0.35726554	7.43740531	10.3084338	8.92233662
A_52_P64356	NM_010097	Sparcl1	Mus musculu	chr5:1045082	9.6545375	-6.3715241	0.01207759	-3.3501217	0.09806474	-3.0214025	0.1231593	6.52356196	12.8950861	9.54496443
A_52_P99411	NM_008410	Itm2b	Mus musculu	chr14:737628	10.4817874	-2.5294153	0.17320887	-1.4313577	0.3707818	-1.0980576	0.46714502	9.27262978	11.8020451	10.3706874
A_55_P20249	NM_0010812	Tbc1d4	Mus musculu	chr14:101841	8.4597616	-0.253799	0.83868502	-2.4263567	0.18603466	2.17255766	4.50821918	9.09934781	9.35314683	6.92679015
A_52_P30630	NM_0010355	Akap2	Mus musculu	chr4:5790593	9.03245891	-2.481869	0.17901235	-1.9500793	0.25880201	-0.5317897	0.69169611	8.027906	10.509775	8.55969574
A_55_P20655	NM_007897	Ebf1	Mus musculu	chr11:448184	9.23212521	-2.1017048	0.23298278	-1.6839862	0.31122154	-0.4177186	0.7486075	8.39231742	10.4940222	8.81003601
A_55_P23972	AK046698	B430316J06R	Mus musculu	chr6:9386781	7.71270941	0.20669215	1.15403914	-2.1958544	0.21826392	2.40254656	5.28735632	8.58245565	8.37576349	6.17990909
A_55_P21465	NM_207105	H2-Ab1	Mus musculu	chr17:344063	7.25453611	-2.037796	0.2435355	-1.6075211	0.32816173	-0.4302749	0.74212034	6.43184579	8.46964182	6.86212073
A_55_P20133	NM_023132	Renbp	Mus musculu	chrX:7117559	8.10832039	-2.8035822	0.14323121	-0.8135067	0.56899712	-1.9900754	0.25172572	6.51043452	9.3140167	8.50050996
A_51_P42374	NM_018777	Cldn6	Mus musculu	chr17:238190	9.10823729	-4.0660892	0.05970149	0.29949185	1.23071085	-4.365581	0.04850976	6.29768055	10.3637697	10.6632616
A_30_P01028	chr15:3946037-3972747_F		lincRNA:chr15	chr15:394606	8.19564647	0.40926582	1.32800983	2.92590613	7.5995086	-2.5166403	0.17474943	7.49318831	7.08392248	10.0098286
A_52_P15300	NM_134032	Hoxb2	Mus musculu	chr11:962151	8.3858371	-3.5868546	0.08322412	-1.2273644	0.42709699	-2.3594902	0.19485999	6.40372219	9.99057675	8.76321237
A_30_P01026	chr14:55433382-55452757		lincRNA:chr14	chr14:554450	8.85175188	-2.8420214	0.13946534	-1.9359559	0.26134801	-0.9060655	0.53363844	7.60238957	10.444411	8.50845506
A_55_P19855	ENSMUST00000119783		GATA zinc fin	chr5:3643283	9.6706229	2.40426954	5.2936747	0.23736836	1.17884036	2.16690118	4.49057809	11.1943465	8.79007693	9.02744529
A_51_P44754	NM_008341	Igfbp1	Mus musculu	chr11:710244	9.10747413	-3.9818864	0.06328966	-0.1249173	0.91705662	-3.8569691	0.0690139	6.49452228	10.4764087	10.3514914
A_55_P19582	NM_0011624	Arrdc1	Mus musculu	chr2:2478093	9.87934491	-1.3078925	0.40391047	1.17260311	2.2541806	-2.4804957	0.17918283	8.61654884	9.92444139	11.0970445
A_52_P59458	NM_173069	Speer2	Mus musculu	chr16:698590	6.59641596	0.40067121	1.32012195	2.07080817	4.20121951	-1.670137	0.31422351	6.17326071	5.7725895	7.84339767
A_51_P48781	NM_016753	Lxn	Mus musculu	chr3:6726204	9.42117519	-1.3288046	0.39809798	-2.0805396	0.23642596	0.75173505	1.68381665	9.22881869	10.5576232	8.47708364
A_52_P11426	NM_0011133	C1rb	Mus musculu	chr6:1245253	9.01299108	2.43549461	5.40949759	0.84390917	1.79490709	1.59158544	3.01380368	10.3553511	7.91985649	8.76376565
A_51_P43467	NM_021881	Qk	Mus musculu	chr17:104032	9.61888971	-2.4697662	0.18052041	-2.6536958	0.15891247	0.18392965	1.13597387	8.8569442	11.3267104	8.67301456
A_55_P21418	NM_026531	Aen	Mus musculu	chr7:8605319	11.7105485	-2.0794559	0.23660362	-0.3845048	0.76604188	-1.6949511	0.30886513	10.4524129	12.5318688	12.1473639
A_30_P01033	chr8:122712014-12273252		lincRNA:chr8:	chr8:1227245	9.43843593	-1.8370946	0.27988486	0.32339301	1.2512699	-2.1604876	0.22368065	8.10590851	9.94300314	10.2663961
A_51_P39268	NM_011701	Vim	Mus musculu	chr2:1350423	11.4768268	-3.3023104	0.10136908	-3.2092472	0.10812355	-0.0930632	0.93753003	10.3450356	13.647346	10.4380987
A_52_P47031	NM_033521	Laptm4b	Mus musculu	chr15:342136	9.14251022	-2.7148916	0.15231272	-0.0484371	0.96698334	-2.6664546	0.15751329	7.34872815	10.0636198	10.0151827
A_55_P20210	NM_030207	Sfi1	Mus musculu	chr11:308612	9.38266168	-2.4365933	0.18471933	0.04702806	1.03313448	-2.4836213	0.17879505	7.74259014	10.1791834	10.2262115
A_30_P01021	chr5:35975265-35982561_		lincRNA:chr5:	chr5:3597685	12.102496	-2.0615606	0.23955676	-0.8202097	0.56635962	-1.2413509	0.42297641	11.0015255	13.0630861	12.2428764
A_55_P19778	NM_178280	Sall3	Mus musculu	chr18:811631	6.84573194	-2.0985073	0.23349972	-2.0579605	0.2401553	-0.0405468	0.97228637	6.13271392	8.23122118	6.17326071
A_30_P01021	chr16:9249688-9275013_F		lincRNA:chr16	chr16:925998	6.90688307	0.07730573	1.05504587	2.09367802	4.26834862	-2.0163723	0.24717894	6.26052755	6.18322182	8.27689984
A_52_P31807	XR_033857	Gm4870	PREDICTED: M	chr5:1442001	7.81437045	-1.3101027	0.40329218	-2.840279	0.13963389	1.53017633	2.88821138	7.88772834	9.197831	6.357552
A_55_P19683	NM_0011103	Gprc5c	Mus musculu	chr11:114733	7.47423863	-2.5321543	0.17288034	-2.3889597	0.19092002	-0.1431946	0.90551181	6.58245565	9.11460995	6.72565028
A_30_P01031	chr17:84470764-84486339		lincRNA:chr17	chr17:844783	7.07511939	0.40919493	1.32794457	2.2963811	4.91224018	-1.8871862	0.2703338	6.58245565	6.17326071	8.46964182
A_52_P10168	ENSMUST00000061000		AV256778 RII	chr12:588387	6.70158146	-2.0390192	0.2433291	-1.9620083	0.2566709	-0.0770109	0.9480198	5.99623808	8.03525732	6.07324898
A_55_P20658	NM_030206	Cygb	Mus musculu	chr11:116507	11.7448306	-2.0380213	0.24349748	-0.2940055	0.81563439	-1.7440158	0.29853753	10.4841516	12.5221728	12.2281673
A_55_P19594	NM_030696	Slc16a3	Mus musculu	chr11:120820	9.90404846	-3.3786411	0.09614521	-1.5184408	0.34906296	-1.8602003	0.27543803	8.15776798	11.5364091	10.0179683
A_55_P19999	NM_175521	6430598A04R	Mus musculu	chr5:1381723	12.678567	0.94072771	1.91949621	2.10685693	4.30751831	-1.1661292	0.44561533	12.6034332	11.6627055	13.7695624
A_66_P12656	NM_026865	1700113I22R	Mus musculu	chr11:101280	7.49930041	-1.5656657	0.33782178	-2.0877486	0.23524752	0.52208281	1.43602694	7.15143943	8.71710517	6.62935662
A_30_P01022	chr3:83089315-83108396_		lincRNA:chr3:	chr3:8310491	8.17964331	-2.1573601	0.22416608	-1.7460806	0.29811057	-0.4112795	0.75195618	7.32343012	9.4807902	7.73470962
A_52_P28806	NM_008021	Foxm1	Mus musculu	chr6:1283247	9.13699817	-1.7479569	0.2977231	-2.4843831	0.17870066	0.73642617	1.66604361	8.79982125	10.5477782	8.06339508
A_55_P19749	NM_033596	Hist2h4	Mus musculu	chr3:9606723	11.1399548	-2.1771958	0.2211051	-0.1673471	0.89047865	-2.0098487	0.24829916	9.74427324	11.9214691	11.754122
A_51_P16132	NM_175540	Eda2r	Mus musculu	chrX:9453137	8.81578125	-4.1965709	0.05453889	-0.6610123	0.63243438	-3.5355586	0.08623644	6.23840474	10.4349757	9.77396337
A_52_P57766	NM_007904	Ednrb	Mus musculu	chr14:104214	8.78003465	-3.9908918	0.06289583	-3.1514547	0.11254277	-0.8394371	0.55886158	7.169925	11.1608168	8.0093621
A_30_P01031	chr11:31647436-31647791		lincRNA:chr11	chr11:316476	7.22144619	-0.1029096	0.93115318	1.97495258	3.93115318	-2.0778622	0.23686515	6.49452228	6.59743185	8.57238443
A_51_P44682	NM_176952	6430573F11R	Mus musculu	chr8:3757576	6.95410498	-2.2453479	0.21090308	-1.6170951	0.32599119	-0.6282528	0.64695946	5.99623808	8.24158599	6.62449086
A_51_P50449	NM_007866	Dll3	Mus musculu	chr7:2907882	11.1826813	-0.9533756	0.51642274	-2.7385386	0.14983654	1.78516309	3.44657423	11.4599438	12.4133194	9.67478076

A_51_P1945(	NM_146717	Olfr433	Mus musculu	chr1:175972(	7.59720123	0.04117741	1.02895323	2.31871138	4.98886414	-2.277534	0.20625	6.85174904	6.81057163	9.12928302
A_55_P2123!	NM_023844	Jam2	Mus musculu	chr16:84816!	9.78201288	-2.9757199	0.12712151	-2.9314089	0.1310865	-0.044311	0.96975286	8.77533593	11.7510558	8.8196469
A_30_P0102:	chr5:22887983-22939658_	lincRNA:chr5:	chr5:2289012	8.70336184	2.1087956	4.31331058	0.20612258	1.15358362	1.90267302	3.73905325	10.040518	7.93172245	8.13784503
A_52_P2828:	NM_0010053	Spin2	Mus musculu	chrX:1502686	8.53381572	2.2829018	4.86655818	0.28362526	1.2172498	1.99927654	3.99799465	9.9612085	7.6783067	7.96193196
A_52_P1886(	NM_0011597	Pvrl2	Mus musculu	chr7:2030992	10.4513463	-1.4816605	0.35807645	0.66834413	1.58924784	-2.1500046	0.2253119	9.24079133	10.7224518	11.3907959
A_55_P2199:	AK033854	9330102E08R	Mus musculu	chr6:1281197	8.25772885	-2.8479676	0.13889171	-1.4150864	0.37498729	-1.4328812	0.37039046	6.83077927	9.67874684	8.26366044
A_52_P2358(	NM_198635	Gm5134	Mus musculu	chr10:754713	7.24053724	0.0908001	1.06496063	2.41964505	5.3503937	-2.328845	0.19904341	6.49452228	6.40372219	8.82336724
A_55_P1953(	NM_0011711	Yap1	Mus musculu	chr9:7932084	8.74577886	-2.8800502	0.13583714	-2.311333	0.2014742	-0.5687172	0.67421603	7.59618976	10.4762399	8.16490693
A_51_P2087(	NM_133719	Metrn	Mus musculu	chr17:259319	8.4522856	-1.7791316	0.29135873	-2.2056309	0.21678984	0.42649936	1.34396853	8.00140819	9.78053977	7.57490884
A_55_P2019:	NM_146543	Olfr1360	Mus musculu	chr13:21765!	7.4060069	0.51281271	1.42682927	2.63255196	6.20121951	-2.1197392	0.2300885	6.87036472	6.357552	8.99010396
A_30_P0103:	chr8:122712014-12273252	lincRNA:chr8:	chr8:1227274	8.01495075	-2.3159043	0.20083682	-0.1576422	0.89648899	-2.1582621	0.22402597	6.52356196	8.83946626	8.68182404
A_55_P2072:	XM_0014741	Gm3789	PREDICTED: M	chr14:198756	12.0619037	-2.4119977	0.18789549	0.71966927	1.64680447	-3.131667	0.11409702	10.2140155	12.6260132	13.3456824
A_52_P2791!	NM_016794	Vamp8	Mus musculu	chr6:7233557	8.60394137	-2.3622098	0.19449301	-0.5506926	0.68269231	-1.8115172	0.28489117	7.21269903	9.57490884	9.02421624
A_55_P2074(	NM_008812	Padi2	Mus musculu	chr4:1405084	8.37822411	-2.7219434	0.15157005	-1.2238451	0.4281401	-1.4980983	0.35401975	6.97154355	9.69348696	8.46964182
A_52_P1455:	NM_011648	Tshr	Mus musculu	chr12:927437	7.03008109	1.78133576	3.43744292	-0.2263504	0.85479452	2.00768619	4.02136752	8.29308841	6.51175265	6.28540222
A_30_P0101(	chr9:14460976-14488051_	lincRNA:chr9:	chr9:1448448	8.39220846	-1.9489741	0.25900035	-2.5664244	0.16882209	0.61745035	1.53416149	7.94836723	9.89734128	7.33091688
A_55_P2069!	NM_028841	Tspan17	Mus musculu	chr13:548975	9.77848533	-2.6076928	0.16406134	-0.1976551	0.87196665	-2.4100377	0.18815093	8.10590851	10.7136013	10.5159462
A_51_P1404:	NM_026918	Zg16	Mus musculu	chr7:1341938	8.17793258	2.09958134	4.28584995	0.06823474	1.04843305	2.03134661	4.08786232	9.55490856	7.45532722	7.52356196
A_55_P2139(	XR_033857	Gm4870	PREDICTED: M	chr5:1441999	8.81087571	-1.7377913	0.29982834	-2.898112	0.13414712	1.16032072	2.23507109	8.6183855	10.3561768	7.45806478
A_55_P2147(	NM_009398	Tnfaip6	Mus musculu	chr2:5191207	6.57839946	-1.2604542	0.41741253	-2.0392675	0.24328723	0.77881334	1.71571906	6.41785251	7.6783067	5.63903917
A_66_P1351(	NM_007960	Etv1	Mus musculu	chr12:39593(	9.70039295	1.95350154	3.87313433	-2.3683588	0.19366582	4.3218603	19.9990602	11.7921802	9.83867869	7.47031993
A_52_P6610:	NR_003270	Snhg3	Mus musculu	chr4:131908(	8.82374347	1.46546372	2.7615222	-0.8622192	0.55010571	2.32768295	5.01998463	10.0881257	8.62266197	7.76044275
A_55_P2164!	NM_029766	Dtl	Mus musculu	chr1:1933613	7.6343205	-1.4236591	0.37276568	-2.6804181	0.1559961	1.25675908	2.38958333	7.57868718	9.00234623	6.32192809
A_55_P1955:	XM_0014796	Gm9195	PREDICTED: M	chr14:728258	7.77307122	-2.0420418	0.24281984	-0.3823463	0.76718886	-1.6596954	0.31650596	6.53915881	8.58120058	8.19885426
A_55_P2059:	NM_0011099	Col18a1	Mus musculu	chr10:76514!	7.86746964	-3.0768156	0.11851852	-2.7621191	0.14740741	-0.3146965	0.8040201	6.73696559	9.81378119	7.05166212
A_30_P0102:	chr2:167388900-16740247	lincRNA:chr2:	chr2:1674008	7.04875616	0.56241993	1.47674419	2.09415749	4.26976744	-1.5317376	0.34586057	6.72565028	6.16323035	8.25738784
A_55_P2088!	NM_0010799	Repin1	Mus musculu	chr6:4854902	10.1851472	-2.1897996	0.21918187	-0.9628014	0.5130597	-1.2269983	0.42720539	9.04621455	11.2360142	10.2732128
A_30_P0103(	chr5:77084398-77086144_	lincRNA:chr5:	chr5:770848!	7.71647461	1.73865121	3.33723022	-1.1911631	0.43794964	2.92981432	7.6201232	9.27262978	7.53397857	6.34281546
A_52_P4975:	NM_145940	Wipi1	Mus musculu	chr11:109435	7.97773622	-1.0552386	0.48121762	0.96596368	1.95336788	-2.0212023	0.24635279	6.9522559	8.00749454	8.97345821
A_55_P2167:	ENSMUST00000025618		proprotein cc	chr19:175079	6.68653995	0.55331687	1.46745562	2.05855218	4.16568047	-1.5052353	0.35227273	6.36923381	5.81591694	7.87446912
A_55_P1973(	NM_008220	Hbb-b1	Mus musculu	chr7:1109611	6.05824307	2.03479896	4.09765625	-0.1051822	0.9296875	2.1399812	4.40756303	7.44983646	5.4150375	5.30985526
A_51_P4862:	NM_175638	Wnk4	Mus musculu	chr11:101138	7.95726224	-0.2488396	0.84157303	2.48252927	5.58876404	-2.7313689	0.15058303	6.96385941	7.21269903	9.69522829
A_51_P2346(	NR_003513	Neat1	Mus musculu	chr19:584262	8.79168717	-2.4422124	0.18400127	-1.2829951	0.41094148	-1.1592172	0.44775542	7.59121065	10.033423	8.75042785
A_52_P7258:	NM_008859	Prkcq	Mus musculu	chr2:1122202	7.53678361	1.37203041	2.58834586	-1.1726394	0.44360902	2.54466979	5.83474576	8.84235034	7.47031993	6.29768055
A_51_P2900:	NM_021272	Fabp7	Mus musculu	chr10:57508(	11.896366	-2.4848789	0.17863926	-2.3087461	0.20183579	-0.1761328	0.88507228	11.0093621	13.494241	11.1854949
A_52_P4098:	NM_008872	Plat	Mus musculu	chr8:2389253	10.1326653	-2.5905073	0.16602734	-1.7844272	0.29029121	-0.8060801	0.57193374	9.00046955	11.5909768	9.80654962
A_55_P2269:	NM_138650	Dgkg	Mus musculu	chr16:22469:	7.33051661	0.53440338	1.44834308	2.2035889	4.60623782	-1.6691855	0.31443081	6.9522559	6.41785251	8.62144141
A_55_P2168:	XR_031340	Gm7997	PREDICTED: M	chr8:8329545	10.1172307	-2.9189111	0.13222702	-0.6112485	0.65462994	-2.3076626	0.20198743	8.37503943	11.2939506	10.682702
A_30_P0102(	chr5:22887983-22939658_	lincRNA:chr5:	chr5:228900(	8.85674658	2.18572902	4.54956629	0.17012362	1.12515489	2.0156054	4.0435022	10.2571914	8.07146236	8.24158599
A_30_P0102:	chr1:34734249-34786649_	lincRNA:chr1:	chr1:3478072	9.67683321	-2.575491	0.16776446	-2.3093446	0.20175207	-0.2661464	0.83153771	8.72962074	11.3051117	8.99576715
A_30_P0103:	chr4:54851872-54866706_	lincRNA:chr4:	chr4:5486622	8.03549902	-1.3569087	0.39041794	-2.5960347	0.16539246	1.23912591	2.3605547	7.99623808	9.35314683	6.75711217
A_55_P2076(	NM_011565	Tead2	Mus musculu	chr7:5248874	11.2964367	-1.7373467	0.29992076	-2.7853327	0.14505454	1.04798599	2.06764139	11.0666498	12.8039965	10.0186638
A_51_P4256(	NM_019826	Ivd	Mus musculu	chr2:1187069	7.57266692	0.82003636	1.76545048	-1.5208789	0.34847357	2.34091523	5.06623932	8.62631745	7.80628109	6.28540222
A_30_P0103:	chr6:126766225-12677130	lincRNA:chr6:	chr6:1267696	8.01333305	2.22813715	4.6852861	1.00784075	2.01089918	1.22029639	2.3299458	9.1628109	6.93467375	7.94251451

A_51_P2040(	NM_013820	Hk2	Mus musculu	chr6:826751	7.5817205	-2.6967574	0.15423933	-1.9019109	0.2675887	-0.7948465	0.57640449	6.41785251	9.11460995	7.21269903
A_30_P0103(	chr17:22335458-22466521		lincRNA:chr1	chr17:223355	7.6115442	2.00274225	4.00761035	0.50752918	1.42161339	1.49521307	2.81905782	8.77752931	6.77478706	7.28231624
A_55_P2177	XM_0014729	Gm2813	PREDICTED: M	chr5:9219241	7.5621245	2.56071495	5.9	2.28640166	4.87837838	0.27431329	1.20941828	8.50713391	5.94641896	8.23282062
A_51_P1683	NM_030188	Ttc30a1	Mus musculu	chr2:758173	7.96294513	1.21051456	2.31420162	-0.7917809	0.57763061	2.00229546	4.00636943	9.0338818	7.82336724	7.03158634
A_30_P0101	chr9:121845920-12185812		lincRNA:chr9:	chr9:1218552	7.14251479	2.54015471	5.81651376	0.33772418	1.26376147	2.20243052	4.60254083	8.72337653	6.18322182	6.52094601
A_30_P0102	chr2:59235155-59242518_		lincRNA:chr2:	chr2:5923728	12.5029584	-2.5507704	0.17066387	-0.2381107	0.84785492	-2.3126597	0.201289	10.881815	13.4325854	13.1944747
A_30_P0103	chr3:4797557-4798821_F		lincRNA:chr3:	chr3:479789	6.8911521	2.5433148	5.82926829	0.812373	1.75609756	1.73094181	3.31944444	8.31590431	5.7725895	6.5849625
A_30_P0102	chr9:43738583-43757007_		lincRNA:chr9:	chr9:4374733	7.84802213	0.20258788	1.15076072	2.6028105	6.0746888	-2.4002226	0.18943534	7.11547722	6.91288934	9.51569984
A_30_P0102	chr9:30805020-30810645_		lincRNA:chr9:	chr9:3080531	6.47647255	0.39854938	1.31818182	2.65257341	6.28787879	-2.254024	0.20963855	5.857981	5.45943162	8.11200503
A_30_P0102(	NEAT1		lincRNA:chr1	chr19:584265	9.03670805	-2.5915555	0.16590675	-0.7367534	0.60008825	-1.8548021	0.27647059	7.55458885	10.1461444	9.40939094
A_55_P2179(	NM_0011776	Mrpl15	Mus musculu	chr1:476459	7.90264224	0.62946721	1.54699358	-1.3935813	0.3806188	2.02304849	4.06441718	8.78681414	8.15734694	6.76376565
A_30_P0103(	chr9:14460976-14488051_		lincRNA:chr9:	chr9:1448454	8.17916163	-1.8482821	0.27772287	-2.7732551	0.14627397	0.92497295	1.89864865	7.87139191	9.71967402	6.94641896
A_55_P1982	NM_010117	Rhbdf1	Mus musculu	chr11:321096	7.8947893	-2.7787047	0.14572247	-2.5453543	0.17130577	-0.2333504	0.85065711	6.89077093	9.66947565	7.12412131
A_55_P1974	NM_177545	Vangl1	Mus musculu	chr3:101962	6.71895812	-2.3087527	0.20183486	-2.5938134	0.1656473	0.28506071	1.21846154	6.04439412	8.35314683	5.75933341
A_52_P6340	NM_013822	Jag1	Mus musculu	chr2:136907	8.88786533	-2.2229425	0.21420403	-2.6408071	0.16033852	0.41786455	1.33594864	8.28617268	10.5091152	7.86830813
A_55_P2261	AK082735	C230098O21	Mus musculu	chr8:716567	9.37879626	2.33057482	5.03005725	0.46043245	1.3759542	1.87014237	3.65568655	10.7790353	8.4484605	8.90889295
A_66_P1144	NM_177328	Grm7	Mus musculu	chr6:111517(	9.32494573	2.15042622	4.4395893	0.4831613	1.39780325	1.66726492	3.17611889	10.5975094	8.44708323	8.93024453
A_30_P0102	chr16:46384974-46391331		lincRNA:chr1(	chr16:463881	8.07312818	-2.8486354	0.13882743	-2.3925169	0.19044985	-0.4561185	0.72894482	6.97154355	9.82017896	7.42766204
A_55_P2126	NM_010195	Lgr5	Mus musculu	chr10:11488	7.17955996	-2.2992527	0.20316832	-2.313383	0.20118812	0.01413033	1.00984252	6.41785251	8.71710517	6.40372219
A_55_P2176	NM_177782	Prex1	Mus musculu	chr2:166392	9.18174206	-2.2837043	0.20536976	-2.081623	0.2362485	-0.2020814	0.86929553	8.35314683	10.6368512	8.5552282
A_66_P1281	NM_022721	Fzd5	Mus musculu	chr1:647795	7.67895678	-2.4193939	0.18693467	-1.4193939	0.37386935	-1	0.5	6.53915881	8.95855272	7.53915881
A_30_P0102(	chr19:5771425-5848475_F		lincRNA:chr1	chr19:584242	9.15911863	-2.7558895	0.14804529	-1.5896703	0.33224738	-1.1662192	0.44558753	7.85174904	10.6076385	9.01796829
A_55_P2094	NM_008500	Lhx6	Mus musculu	chr2:359375	12.847563	4.72430883	26.4337432	4.02616815	16.292862	0.69814068	1.62241251	14.6550462	9.93073734	13.9569055
A_66_P1397	NM_080555	Ppap2b	Mus musculu	chr4:104905(	7.67004806	-1.5230794	0.34794246	-2.3482305	0.19638675	0.82515112	1.77172061	7.43740531	8.96048468	6.61225419
A_51_P4527	NM_133198	Pygl	Mus musculu	chr12:71291	8.33848409	-2.2322964	0.2128197	-2.8840673	0.13545943	0.65177094	1.57109557	7.81164228	10.0439387	7.15987134
A_55_P2005	XR_033524	Gm7996	PREDICTED: M	chr1:178767	7.07560027	-1.0973564	0.46737213	-2.1174576	0.23045267	1.02010121	2.02806122	7.04984855	8.14720492	6.02974734
A_51_P1717	NM_021399	Bcl11b	Mus musculu	chr12:10915	8.40409922	2.50460422	5.67493639	1.60742444	3.04707379	0.89717978	1.86242171	9.53802722	7.033423	8.64084744
A_55_P2154	NM_007554	Bmp4	Mus musculu	chr14:47003	9.31263705	-2.49986	0.17679385	-1.4727823	0.36028681	-1.0270778	0.49070308	8.13699111	10.6368512	9.16406888
A_52_P3547	NM_011401	Slc2a3	Mus musculu	chr6:122678	10.2170037	-2.8401732	0.13964413	-0.0887759	0.94032028	-2.7513973	0.14850698	8.35314683	11.19332	11.1045442
A_55_P1969	NM_020259	Hhip	Mus musculu	chr8:824943	7.40150897	-2.4744185	0.17993921	-2.6177827	0.16291793	0.14336418	1.10447761	6.62449086	9.09890937	6.48112669
A_51_P4002	NM_016982	Vpreb1	Mus musculu	chr16:16868	8.10113898	1.08714575	2.124533	2.02339015	4.06537983	-0.9362444	0.52259151	8.15143943	7.06429368	9.08768383
A_55_P2053	NM_178362	Sorbs1	Mus musculu	chr19:40366	11.1068777	2.02145438	4.05992866	1.18550833	2.2744352	0.83594605	1.78502719	12.0593445	10.0378901	11.2233984
A_30_P0101	chr12:16003706-16022581		lincRNA:chr1	chr12:16008	10.2750422	1.5228319	2.87354552	-0.4810393	0.71646133	2.00387116	4.01074755	11.4506099	9.92777796	9.4467387
A_55_P2141	NM_001136(	Ldha	Mus musculu	chr7:541072	12.4507376	-3.434172	0.0925148	-0.5223024	0.69625979	-2.9118696	0.13287397	10.3353904	13.7695624	13.24726
A_55_P1972(	NM_015760	Nox4	Mus musculu	chr7:945470	6.94807001	1.6960233	3.24006623	-0.81214	0.56953642	2.50816329	5.68895349	8.34946554	6.65344224	5.84130225
A_55_P2305(	XR_035181	A730089K16	PREDICTED: M	chr5:770850	9.14538019	0.93440491	1.91110217	-1.3370961	0.39581657	2.27150099	4.82825203	10.2140155	9.27961058	7.94251451
A_55_P2062	XM_0014756	Gm3094	PREDICTED: M	chr7:668146	11.7618137	-0.7708892	0.58605617	1.33207679	2.51764834	-2.1029659	0.2327792	10.803862	11.5747512	12.906828
A_51_P2234	NM_133655	Cd81	Mus musculu	chr7:150253	13.0504108	-2.1185055	0.23028534	-0.2699653	0.82933949	-1.8485402	0.27767319	11.7280622	13.8465677	13.5766024
A_30_P0103(	chr6:131263850-13131485		lincRNA:chr6:	chr6:131272	8.28393302	0.25210665	1.19094488	2.38852584	5.23622047	-2.1364192	0.22744361	7.65582883	7.40372219	9.79224803
A_55_P2302	NM_011419	Kdm5d	Mus musculu	chrY:277162-	6.62014579	3.06288771	8.35643564	-0.1770848	0.88448845	3.2399725	9.44776119	8.72109919	5.65821148	5.48112669
A_51_P2463	NM_008630	Mt2	Mus musculu	chr8:966973	11.2119458	-2.9275617	0.13143654	-3.2638105	0.10411065	0.3362488	1.26246973	10.3481749	13.2757365	10.0119261
A_55_P2048	XM_0014783	LOC1000475	PREDICTED: M	chr11:88964	7.45214212	0.1991486	1.14802065	2.36498221	5.15146299	-2.1658336	0.22285332	6.79658045	6.59743185	8.96241406
A_51_P5060	NM_176073	Pgcp	Mus musculu	chr15:33524(	7.13783957	0.68417818	1.60678643	-1.4217723	0.37325349	2.10595051	4.30481283	8.06788247	7.38370429	5.96193196
A_66_P1031	ENSMUST00000103482		predicted ger	chr12:11553	7.86441088	0.37895605	1.30040053	2.32269835	5.00267023	-1.9437423	0.25994129	7.34281546	6.96385941	9.28655776

A_51_P32085	NM_007657	Cd9	Mus musculu	chr6:1254104	8.71679125	-2.9841821	0.12637806	-1.6926538	0.30935735	-1.2915283	0.40851804	7.29155445	10.2757365	8.58308277
A_51_P37644	NM_008818	Rhox5	Mus musculus reproductive		9.8630231	-4.5529021	0.04260297	-1.0763157	0.47423836	-3.4765863	0.08983452	7.18652697	11.739429	10.6631133
A_52_P10370	ENSMUST00000098718		Putative uncl	chr8:6755260	9.67064402	-5.0206405	0.03080609	-2.715805	0.15221633	-2.3048355	0.20238363	7.22881869	12.2494592	9.53365419
A_55_P20770	NR_028271	Phxr4	Mus musculu	chr9:1323493	8.01411674	-2.0695838	0.23822821	-3.1598192	0.11189215	1.09023543	2.12908778	7.68766728	9.7572511	6.59743185
A_55_P21220	NM_146332	Olfr135	Mus musculu	chr17:383460	6.27920931	0.4918531	1.40625	2.10066234	4.2890625	-1.6088092	0.32786885	5.9068906	5.4150375	7.51569984
A_52_P52780	NM_145158	Emilin2	Mus musculu	chr17:716016	8.36222683	-3.0009014	0.12492192	-2.6015784	0.16475814	-0.399323	0.75821398	7.22881869	10.2297201	7.62814172
A_55_P20097	NM_009573	Zic1	Mus musculu	chr9:9125736	10.686408	2.57081542	5.94145147	0.84386933	1.79485752	1.72694609	3.31026357	12.1189951	9.54817971	10.392049
A_52_P39528	NM_0010333	Lrrc55	Mus musculu	chr2:8502867	7.35736558	2.07691737	4.21904762	0.64154603	1.56	1.43537134	2.7045177	8.52812848	6.45121111	7.09275714
A_30_P01024	chr6:127111250-12714292		lincRNA:chr6:	chr6:1271335	13.2087679	-2.2132562	0.21564704	-0.4947845	0.7096677	-1.7184717	0.30387045	11.898192	14.1114482	13.6166637
A_52_P59333	NM_029327	Lyrm7	Mus musculu	chr11:546536	6.55674488	1.94525704	3.85106383	-0.1839014	0.88031915	2.12915848	4.37462236	7.91488339	5.96962635	5.78572491
A_51_P45886	NM_172647	F11r	Mus musculu	chr1:1733938	9.13429712	-2.340428	0.19745174	-0.5359428	0.68970779	-1.8044852	0.28628319	7.7526594	10.0930874	9.55714456
A_51_P17608	NM_146187	Ffar2	Mus musculu	chr7:3160420	8.70188119	0.1904742	1.14113873	2.81731963	7.04851644	-2.6268454	0.16189772	7.88975745	7.69928325	10.5166029
A_55_P20979	XM_0014810	9030622O22I	PREDICTED: N	chr2:1478145	7.39236509	-2.8251296	0.14110787	-2.4748662	0.17988338	-0.3502634	0.78444084	6.33390074	9.15903036	6.68416418
A_51_P50435	NM_025980	Nrarp	Mus musculu	chr2:2503803	7.834756	-1.0979057	0.4671942	-2.3425281	0.19716452	1.24462237	2.36956522	7.88366154	8.98156728	6.63903917
A_52_P55255	NM_009642	Agtrap	Mus musculu	chr4:1474544	7.77468106	-2.7139541	0.15241174	-2.1282073	0.22874192	-0.5857468	0.66630435	6.67478076	9.38873487	7.26052755
A_55_P19787	NM_011769	Zim1	Mus musculu	chr7:6628801	8.06311294	-2.1446728	0.22614612	-0.6863691	0.62141584	-1.4583038	0.36392075	6.86212073	9.00679358	8.3204245
A_51_P22080	NM_008110	Gdf9	Mus musculu	chr11:532513	8.57352431	-2.2221503	0.21432168	-1.1346874	0.45543358	-1.0874628	0.47058824	7.47031993	9.69247021	8.55778278
A_52_P12365	ENSMUST00000059200		RIKEN cDNA /	chr5:1109546	6.38613986	0.53680646	1.45075758	2.24331826	4.73484848	-1.7065118	0.3064	5.99623808	5.45943162	7.70274988
A_55_P19530	NM_008563	Mcm3	Mus musculu	chr1:2079319	9.39604553	-1.8010649	0.2869627	-3.5984529	0.08255773	1.79738808	3.47590361	9.39481993	11.1958848	7.59743185
A_66_P10872	ENSMUST00000094634		Testis proteir	chr4:1335602	8.80763071	-2.6576718	0.15847511	-1.0608078	0.47936358	-1.596864	0.33059481	7.38945209	10.0471239	8.98631612
A_30_P01029	NEAT1		lincRNA:chr19	chr19:584271	7.79513159	-2.2864309	0.20498199	-1.6798049	0.31212485	-0.606626	0.65673077	6.83077927	9.11721018	7.43740531
A_55_P21746	XM_0014778	LOC1000472	PREDICTED: N	chr7:1232672	7.11839345	-2.1328943	0.228	-2.1641657	0.22311111	0.03127146	1.02191235	6.41785251	8.55074679	6.38658105
A_51_P14251	NM_133738	Antxr2	Mus musculu	chr5:9831387	7.88916206	-1.2162341	0.43040474	-2.0695351	0.23823626	0.85330094	1.80662983	7.76818432	8.98441846	6.91488339
A_55_P19656	NM_178894	AA792892	Mus musculu	chr5:9481329	7.06723758	0.6552412	1.57487923	2.22089817	4.66183575	-1.565657	0.33782383	6.76376565	6.10852446	8.32942263
A_51_P35720	NM_0010811	Zfp518b	Mus musculu	chr5:3906019	9.65625954	2.12644626	4.36640593	0.85877953	1.8135035	1.26766673	2.4077185	10.7876305	8.66118427	9.5199638
A_55_P20696	NM_0010247	C1ql4	Mus musculu	chr15:989152	8.52238566	-2.908714	0.13316492	-1.0778145	0.47374594	-1.8308995	0.28108932	6.94251451	9.85122849	8.77341398
A_52_P60303	NM_016968	Olig1	Mus musculu	chr16:912717	7.25883691	-3.2049436	0.10844658	-2.8851439	0.13535838	-0.3197997	0.8011811	6.08392248	9.28886607	6.40372219
A_55_P20360	NM_198409	Rai2	Mus musculu	chrX:1582173	11.5852171	2.0170903	4.04766614	0.58210624	1.49703323	1.43498405	2.70379178	12.7359085	10.7188182	11.3009245
A_52_P35253	NM_172851	Cntnap5b	Mus musculu	chr1:1023785	7.08159728	2.11122983	4.32059448	0.24969977	1.18895966	1.86153006	3.63392857	8.40585058	6.29462075	6.54432052
A_51_P25545	NM_009994	Cyp1b1	Mus musculu	chr17:801066	6.78049855	-2.2113541	0.21593153	-1.3989812	0.37919684	-0.812373	0.56944444	5.7725895	7.98394365	6.5849625
A_66_P10461	ENSMUST00000069762		Putative uncl	chr8:4348678	9.83795286	-2.2627449	0.20837515	-0.7287845	0.6034121	-1.5339604	0.34532809	8.57238443	10.8351293	10.1063448
A_52_P26132	NM_198294	Tanc1	Mus musculu	chr2:5968162	7.07292669	-2.2441574	0.21107719	-2.2712369	0.2071522	0.02707953	1.01894737	6.33390074	8.57805814	6.3068212
A_55_P21424	NM_026728	Echdc2	Mus musculu	chr4:1078518	7.92559176	-0.6334339	0.64464023	-2.0694363	0.23825257	1.43600246	2.70570108	8.19311463	8.82654849	6.75711217
A_55_P21500	ENSMUST00000075226		PREDICTED: N	chrX:1083638	7.86450843	-3.653367	0.07947434	-2.9243753	0.13172716	-0.7289917	0.60332542	6.40372219	10.0570892	7.13271392
A_30_P01018	chr5:96591892-96606467_		lincRNA:chr5:	chr5:9660351	7.87434197	-2.4926162	0.17768377	-1.7842713	0.29032258	-0.7083449	0.61202186	6.80735492	9.29997115	7.51569984
A_55_P20897	NM_007904	Ednrb	Mus musculu	chr14:104220	8.60580922	-3.9257529	0.06580072	-3.0453345	0.12113314	-0.8804184	0.54320988	7.00375213	10.929505	7.88417052
A_55_P20405	NM_008888	Phox2b	Mus musculu	chr5:6748582	8.9011949	-4.5063527	0.044	-1.3108228	0.40309091	-3.1955298	0.10915652	6.33390074	10.8402534	9.52943055
A_52_P10678	NM_026434	Rbm18	Mus musculu	chr2:3597166	13.9430785	2.18665259	4.55247973	0.45779433	1.37344042	1.72885826	3.31465395	15.2482488	13.0615962	13.5193905
A_55_P19622	NM_0010839	Hba-a2	Mus musculu	chr11:321972	7.57851633	4.23558957	18.8382046	-0.4567986	0.72860125	4.69238819	25.8553009	10.5545089	6.31891935	5.86212073
A_55_P20910	NM_153153	Svil	Mus musculu	chr18:504936	7.95680449	-1.5144199	0.35003717	-2.0062347	0.24892193	0.49181479	1.40621266	7.6159361	9.13035605	7.12412131
A_55_P19950	NM_181416	Arhgap11a	Mus musculu	chr2:1136717	7.98619176	0.14680289	1.10711332	-2.1521357	0.22497932	2.29893858	4.92095588	8.80143892	8.65463603	6.50250034
A_30_P01027	chr1:71939741-71947891_		lincRNA:chr1:	chr1:7194019	7.07007124	-2.3513607	0.19596111	-2.8378893	0.13986537	0.48652854	1.40106952	6.4484605	8.79982125	5.96193196
A_30_P01033	chr11:97276846-97310021		lincRNA:chr1:	chr11:973097	8.83604513	-1.8704913	0.27348028	0.73870639	1.66867893	-2.6091977	0.16389029	7.34281546	9.21330677	9.95201316
A_51_P23090	NM_019877	Copz2	Mus musculu	chr11:967221	8.87531978	-3.8032017	0.0716345	-1.7785646	0.29147325	-2.024637	0.24576697	6.93270689	10.7359085	8.95734392

A_55_P1961	NR_001592	H19	Mus musculu	chr7:1497615	11.8287154	-5.8145029	0.01776889	-2.6129613	0.1634633	-3.2015415	0.10870261	8.82336724	14.6378701	12.0249088
A_52_P1925	NM_054053	Gpr98	Mus musculu	chr13:816997	8.47968945	-3.5058266	0.08803209	-2.6948239	0.15444618	-0.8110027	0.56998557	7.04074634	10.546573	7.85174904
A_55_P2148	XR_031767	Gm6705	PREDICTED: M	chr17:169859	11.5713147	-3.7164877	0.07607216	-0.8863118	0.54099539	-2.8301759	0.14061517	9.38909352	13.1055812	12.2192694
A_55_P2056	ENSMUST00000064349		RIKEN cDNA A	chr8:1019462	8.7119468	-4.4298848	0.04639506	-5.2114943	0.02698882	0.78160951	1.71904762	7.49585503	11.9257399	6.71424552
A_55_P1985	NM_010919	Nkx2-2	Mus musculu	chr2:1470090	9.90793649	-3.8995606	0.06700625	-0.6499162	0.63731732	-3.2496444	0.10513796	7.52486815	11.4244288	10.7745125
A_30_P0102	chr1:45066850-45095650_		lincRNA:chr1:	chr1:4509174	9.1187969	2.00908671	4.02527322	-0.1997955	0.87067395	2.20888224	4.62316946	10.5247866	8.51569984	8.31590431
A_30_P0102	chr5:35975265-35982561_		lincRNA:chr5:	chr5:3597691	12.3688178	-2.1413303	0.22667069	-0.5951809	0.66196145	-1.5461494	0.34242279	11.1396579	13.2809882	12.6858073
A_52_P4876	NM_027760	Rassf8	Mus musculu	chr6:1457659	8.06663257	-2.4132213	0.18773619	-1.6378013	0.32134585	-0.77542	0.58421851	7.00375213	9.41697341	7.77917215
A_55_P2063	XR_001926	Gm11814	PREDICTED: M	chr4:1036554	13.8069463	-2.9588397	0.12861763	-0.8729194	0.54604076	-2.0859203	0.23554583	12.1253597	15.0841994	14.2112799
A_55_P2039	ENSMUST00000101391		RIKEN cDNA 9	chr6:5435107	6.7780982	1.57426721	2.977842	-0.5438573	0.68593449	2.11812451	4.34129213	8.00889544	6.43462823	5.89077093
A_55_P2374	AK038155	A130071D04	Mus musculu	chr1:1400585	7.30291796	0.42077387	1.33864542	2.32823685	5.02191235	-1.907463	0.26656089	6.80735492	6.38658105	8.7148179
A_52_P1750	ENSMUST00000030257		cache domain	chr4:1006768	10.1035881	-3.9526164	0.06458682	-1.8275862	0.2817356	-2.1250302	0.22924622	8.07770587	12.0303223	10.202736
A_55_P1959	NM_178740	Slitrk4	Mus musculu	chrX:6152280	7.31055411	2.33904836	5.05968779	0.08113676	1.05785124	2.2579116	4.78298611	8.8428741	6.50382574	6.5849625
A_51_P2610	NM_010056	Dlx5	Mus musculu	chr6:6828122	10.885736	2.52437284	5.75323276	1.34799548	2.5455819	1.17637736	2.26008551	12.1193194	9.59494659	10.9429421
A_52_P2647	NM_027924	Pdgfd	Mus musculu	chr9:6376926	6.45968262	0.25525706	1.19354839	2.05030508	4.14193548	-1.795048	0.28816199	5.94641896	5.6911619	7.74146699
A_30_P0102	chr7:86680928-86700632_		lincRNA:chr7:	chr7:8670001	8.40874747	1.02941123	2.04119107	-0.9779737	0.50769231	2.00738493	4.02052786	9.42101286	8.39160162	7.41362793
A_55_P2115	NM_010917	Nid1	Mus musculu	chr13:136027	7.26921579	-3.4418376	0.09202454	-2.7966995	0.14391616	-0.645138	0.63943162	5.9068906	9.34872815	6.55202861
A_51_P1307	NM_024169	Fkbp11	Mus musculu	chr15:985548	8.47990129	-2.1275965	0.22883879	-1.2749886	0.41322841	-0.8526078	0.55378281	7.48649986	9.61409632	8.33910768
A_55_P2156	NM_153513	BC028528	Mus musculu	chr3:9569215	7.55234468	-2.2897259	0.20451436	-1.8395353	0.27941176	-0.4501906	0.73194614	6.63903917	8.9287651	7.08922977
A_66_P1115	NM_145624	Zfp709	Mus musculu	chr8:7441627	6.84246066	1.59760988	3.02641509	-0.464886	0.7245283	2.06249593	4.17708333	8.06249593	6.46488605	6
A_51_P1459	NM_144946	Neto1	Mus musculu	chr18:866703	10.1220385	3.23817922	9.43602484	2.92428877	7.59099379	0.31389045	1.24305527	11.3060617	8.06788247	10.9921712
A_51_P3934	NM_011173	Pros1	Mus musculu	chr16:629286	8.06409097	-2.1087445	0.23184869	-0.9798955	0.50701647	-1.1288491	0.45728039	6.98489311	9.09363764	8.11374217
A_30_P0102	chr17:3004525-3086000_F		lincRNA:chr1	chr17:308168	9.44742875	-2.1629757	0.22329523	1.33342599	2.52000394	-3.4964016	0.08860908	7.56096965	9.7239453	11.0573713
A_55_P2128	NM_172301	Ccnb1	Mus musculu	chr13:101553	9.26230344	-1.5540877	0.34054381	-2.3514505	0.19594892	0.79736279	1.73792135	9.01006182	10.5641495	8.21269903
A_30_P0102	chr5:96591892-96606467_		lincRNA:chr5:	chr5:9660330	9.37696354	-3.0267407	0.12270444	-1.790993	0.28897307	-1.2357476	0.4246224	7.95613411	10.9828748	9.19188173
A_55_P2104	ENSMUST00000113886			chr17:355170	9.5092431	-3.3910599	0.09532115	-1.9175755	0.26469898	-1.4734844	0.36011151	7.88772834	11.2787882	9.36121274
A_55_P1991	NM_026422	Mrrf	Mus musculu	chr2:3604513	9.04500244	2.1104596	4.31828839	0.57022943	1.48475967	1.54023017	2.908409	10.261899	8.15143943	8.72166886
A_30_P0101	chr2:60581618-60589907_		lincRNA:chr2:	chr2:6058981	10.4185116	-1.0771631	0.47395988	-2.021193	0.24635438	0.94402981	1.92389466	10.3741338	11.451297	9.43010403
A_55_P1985	NM_019413	Robo1	Mus musculu	chr16:730461	9.57481212	2.79426591	6.93677896	0.44060763	1.35717582	2.35365828	5.11118667	11.2907869	8.49652094	8.93712857
A_55_P1999	NM_007408	Plin2	Mus musculu	chr4:8630290	8.55407238	-0.0534467	0.96363139	-2.0387834	0.24336888	1.98533668	3.95955056	9.19803571	9.25148241	7.21269903
A_55_P2168	XR_031967	Gm7669	PREDICTED: M	chr8:1374585	8.40184241	-3.0627851	0.11967676	-2.7623553	0.14738327	-0.3004298	0.81201044	7.28077077	10.3435559	7.58120058
A_55_P2156	NM_031257	Plekha2	Mus musculu	chr8:2615269	7.81911055	-2.1891236	0.2192846	-0.5846821	0.66679627	-1.6044415	0.32886297	6.55458885	8.74371243	8.15903036
A_55_P2134	NM_010446	Foxa2	Mus musculu	chr2:1478687	8.35372045	-4.183022	0.0550535	-2.6995408	0.15394204	-1.4834812	0.35762483	6.46488605	10.6479081	7.94836723
A_52_P6634	NM_011031	P4ha2	Mus musculu	chr11:539450	9.22883308	-3.1524425	0.11246574	-1.5137359	0.35020319	-1.6387067	0.32114425	7.63178336	10.7842259	9.27049001
A_52_P6121	NM_009822	Runx1t1	Mus musculu	chr4:1381818	13.3515804	2.00727577	4.02022368	0.57767086	1.49243785	1.42960492	2.69372937	14.4972073	12.4899316	13.0676024
A_55_P2111	NM_021324	Ttyh1	Mus musculu	chr7:4087717	7.28779308	-1.6577638	0.31693002	-2.0632424	0.23927765	0.40547867	1.3245283	6.87036472	8.52812848	6.46488605
A_55_P2061	XM_0010035	Gm8570	PREDICTED: M	chr10:926604	13.8520668	2.84630109	7.19154175	2.96880001	7.8288479	-0.1224989	0.91859516	14.7600008	11.9136998	14.8824998
A_52_P1616	NM_145933	St6gal1	Mus musculu	chr16:233600	9.74665179	2.7880644	6.90702479	1.45018877	2.73243802	1.33787563	2.52778828	11.1219651	8.33390074	9.78408951
A_30_P0102	chr5:35975265-35982561_		lincRNA:chr5:	chr5:3597687	10.7155856	-2.0016616	0.24971222	-0.860562	0.55073798	-1.1410997	0.45341384	9.66799854	11.6696602	10.8090982
A_30_P0102	chr9:114412295-11441838		lincRNA:chr9:	chr9:1144180	8.8255001	-2.0265782	0.24543651	-0.639658	0.64186508	-1.3869202	0.38238022	7.68766728	9.71424552	9.0745875
A_51_P4745	NM_007810	Cyp19a1	Mus musculu	chr9:5401383	8.52164392	1.3798685	2.60244648	2.12562279	4.36391437	-0.7457543	0.59635599	8.73301532	7.35314683	9.47876962
A_55_P1993	XR_031039	LOC676303	PREDICTED: Mus musculus		10.6329659	-3.5510664	0.08531443	-1.0381539	0.4869502	-2.5129125	0.17520155	8.61163963	12.162706	11.1245522
A_55_P1998	NM_030694	Ifitm2	Mus musculu	chr7:1481409	10.2740664	-3.5432897	0.08577555	-1.6180136	0.32578372	-1.9252761	0.26328987	8.45121111	11.9945008	10.3764872
A_52_P4034	NM_0011271	Ptpn2	Mus musculu	chr18:678317	6.03824637	1.49388078	2.8164557	-0.5355964	0.68987342	2.0294772	4.08256881	7.21269903	5.71881825	5.18322182

A_55_P20109	NM_153405	Rbm45	Mus musculu	chr2:7622176	10.0595918	1.95420752	3.8750301	-0.0803589	0.9458223	2.03456646	4.09699593	11.3891832	9.43497566	9.35461671
A_55_P2096(	XM_0014737	Gm2490	PREDICTED: M	chrUn_rando	10.0707514	2.83738116	7.14721485	1.03243363	2.04547177	1.80494753	3.49416451	11.6181943	8.78081313	9.81324676
A_55_P20295	XM_0014761	Gm3256	PREDICTED: M	chr6:5461230	6.86060235	-0.3613139	0.77845528	1.87046497	3.65650407	-2.2317789	0.21289605	5.99623808	6.357552	8.22801697
A_52_P12813	NM_008242	Foxd1	Mus musculu	chr13:991264	6.74978877	-2.0469045	0.24200278	-1.4183856	0.37413074	-0.6285189	0.64684015	5.857981	7.90488546	6.48649986
A_30_P0102(	chr7:86680928-86700632_	lincRNA:chr7:	chr7:8670057		7.46969756	1.88869349	3.70299728	-0.2836221	0.82152589	2.17231555	4.50746269	8.82336724	6.93467375	6.65105169
A_55_P20382	NR_027955	4931440P22R	Mus musculu	chr3:6533296	7.93737293	0.25516616	1.19347319	2.07733862	4.22027972	-1.8221725	0.28279481	7.4150375	7.15987134	9.23720996
A_55_P20493	NM_018779	Pde3a	Mus musculu	chr6:1414478	7.84518996	2.53697078	5.80369128	1.09598104	2.13758389	1.44098974	2.71507064	9.1711768	6.63420602	7.73018706
A_55_P21855	NM_020296	Rbms1	Mus musculu	chr2:6059087	8.62371566	0.20091269	1.14942529	-1.9232587	0.26365829	2.12417141	4.35952637	9.39874369	9.197831	7.27457229
A_30_P01022	chr15:92174226-92201201	lincRNA:chr15	chr15:921964		8.48171868	-2.4903421	0.17796407	-0.3922958	0.76191617	-2.0980463	0.23357435	6.9522559	9.44259798	9.05030216
A_55_P21375	NM_029283	Fam183b	Mus musculu	chr11:586064	8.37616767	-2.7330813	0.15040441	-1.8972404	0.26845638	-0.8358408	0.56025641	7.18652697	9.91960824	8.02236781
A_55_P19543	NM_025905	Ttc23	Mus musculu	chr7:7487139	7.52025172	-1.7012014	0.30752991	-2.4012285	0.18930331	0.70002711	1.62453532	7.18652697	8.88772834	6.48649986
A_55_P20118	NM_183417	Cdk2	Mus musculu	chr10:128135	7.3244107	-1.0681951	0.47691527	-2.0378215	0.2435312	0.96962635	1.95833333	7.29155445	8.35974956	6.32192809
A_55_P21066	NM_011527	Tal1	Mus musculu	chr4:1147442	10.373848	-4.8368176	0.03499233	-0.9016005	0.53529255	-3.935217	0.06537047	7.44983646	12.286654	11.3850535
A_52_P53019	NM_139228	Rhbdl3	Mus musculu	chr11:801691	8.26926917	-2.6382348	0.16062465	-1.978437	0.25376464	-0.6597977	0.63296703	7.169925	9.80815977	7.82972274
A_30_P01019	chr19:5834117-5835940_R	lincRNA:chr19	chr19:583460		7.67288051	-2.7207216	0.15169846	-2.7140987	0.15239646	-0.0066229	0.99541985	6.76376565	9.48448727	6.7703886
A_55_P20809	NM_177717	4732456N10R	Mus musculu	chr15:101382	6.7699203	-2.0469045	0.24200278	-1.357991	0.39012517	-0.6889135	0.62032086	5.857981	7.90488546	6.54689446
A_51_P28573	NM_008798	Pdcd1	Mus musculu	chr1:9593512	6.85079526	0.44822616	1.3643617	2.19528058	4.57978723	-1.7470544	0.29790941	6.41785251	5.96962635	8.16490693
A_51_P23029	NM_008232	Hdgfl1	Mus musculu	chr13:268608	7.55941415	0.42493596	1.34251291	2.46101092	5.5060241	-2.036075	0.2438262	7.02236781	6.59743185	9.05844278
A_55_P20732	NM_138646	Hps4	Mus musculu	chr5:1128073	9.59802441	-2.0392451	0.243291	-0.8452909	0.55659856	-1.1939542	0.43710317	8.52029128	10.5595364	9.71424552
A_66_P11216	NM_144875	Rab7l1	Mus musculu	chr1:1337694	7.48430309	-2.1120409	0.23131955	-2.5366953	0.17233704	0.4246544	1.34225092	6.92184094	9.0338818	6.49718654
A_52_P59737	NM_134094	Ncald	Mus musculu	chr15:373270	8.2487025	-2.2750722	0.20660223	-1.1033953	0.46541986	-1.1716769	0.44390507	7.09978612	9.37485836	8.27146303
A_55_P24086	AK051100	4933439C10R	Mus musculu	chr11:593229	8.26895265	0.65726298	1.57708779	2.30264626	4.93361884	-1.6453833	0.31966146	7.93957921	7.28231624	9.5849625
A_52_P5101(	NM_172527	Nudt15	Mus musculu	chr14:739197	7.67458062	1.7959692	3.47248677	-0.6698514	0.62857143	2.4658206	5.52441077	9.09517722	7.29920802	6.62935662
A_51_P50245	NR_004446	Gm7035	Mus musculu	chr17:341120	10.7019318	-2.1080072	0.23196721	-1.0516947	0.48240116	-1.0563125	0.48085957	9.64715858	11.7551658	10.703471
A_30_P01024	chr18:5162836-5165729_R	lincRNA:chr18	chr18:516301		9.26511941	-2.6968203	0.1542326	-0.6296794	0.64632002	-2.0671409	0.23863195	7.67713234	10.3739527	9.74427324
A_52_P37681	NM_012056	Fkbp9	Mus musculu	chr6:5682919	8.72448891	-3.7960017	0.07199289	-2.429793	0.18559207	-1.3662087	0.38790932	7.00375213	10.7997538	8.36996079
A_51_P4963(	NM_0010249	Rfx4	Mus musculu	chr10:843684	7.44052482	-2.3623354	0.19447608	-2.8954341	0.13439636	0.53309872	1.4470339	6.83077927	9.19311463	6.29768055
A_51_P52084	NM_009144	Sfrp2	Mus musculu	chr3:8357786	9.29776344	-3.2842888	0.10264329	-2.7019736	0.15368267	-0.5823152	0.6678911	8.00889544	11.2931842	8.59121065
A_51_P2548(	NM_008446	Kif4	Mus musculu	chrX:9792208	7.24159487	-1.2459887	0.42161885	-2.0665512	0.23872951	0.82056248	1.76609442	7.09978612	8.34577484	6.27922364
A_55_P19798	NM_028806	Phactr3	Mus musculu	chr2:1780707	8.22445484	2.11769504	4.34	0.86921339	1.82666667	1.24848165	2.37591241	9.34651373	7.22881869	8.09803208
A_55_P19671	NM_172668	Lrp4	Mus musculu	chr2:9135356	7.68054156	-1.5343009	0.3452466	-2.0197277	0.24660472	0.48542683	1.4	7.33091688	8.86521775	6.84549005
A_55_P19932	NM_146860	Olfr161	Mus musculu	chr16:359328	7.1106217	0.52418287	1.43811881	2.58793529	6.01237624	-2.0637524	0.23919308	6.59743185	6.07324898	8.66118427
A_55_P19532	NR_033506	Gm3893	Mus musculu	chrUn_rando	9.73832877	3.54205893	11.6483922	0.75930299	1.69267264	2.78275595	6.8816568	11.8466004	8.30454146	9.06384445
A_55_P20635	XM_0014795	Gm4235	PREDICTED: M	chr12:336829	11.9700325	3.05165414	8.29162081	2.70821726	6.53513598	0.34343689	1.26877556	13.1017295	10.0500754	12.7582926
A_51_P3386(	NM_025387	Tmem14c	Mus musculu	chr13:411175	9.87536107	-1.8584086	0.27578032	0.5680164	1.48248387	-2.426425	0.18602585	8.44708323	10.3054918	10.8735082
A_55_P19739	ENSMUST00000077710		predicted ger	chr18:370817	8.58504327	-2.8562885	0.13809294	-2.6935087	0.15458705	-0.1627798	0.89330218	7.57868718	10.4349757	7.74146699
A_30_P01031	chr1:34734249-34786649_	lincRNA:chr1:	chr1:3478059		8.1075695	-3.3851023	0.09571559	-2.5902326	0.16605895	-0.7948697	0.57639524	6.71424552	10.0993478	7.50911518
A_52_P32114	NM_007843	Defb1	Mus musculu	chr8:2290504	6.66929636	0.52509104	1.43902439	2.16502953	4.4847561	-1.6399385	0.32087016	6.29768055	5.7725895	7.93761903
A_52_P63922	NM_139232	Fgd4	Mus musculu	chr16:164223	9.09300093	1.82478952	3.54255319	-0.2095533	0.86480496	2.03434281	4.09636084	10.3793784	8.55458885	8.34503556
A_66_P11631	NM_008448	Kif5b	Mus musculu	chr18:620257	6.98917684	2.84356744	7.17792793	-0.504397	0.70495495	3.34796446	10.1821086	9.0530208	6.20945337	5.70505635
A_52_P35236	BC147147	Gm7265	Mus musculu	chr9:7254270	9.68441188	1.70080836	3.25083056	-0.3484317	0.78543743	2.04924011	4.1388791	10.934428	9.23361968	8.88518793
A_52_P6791(	NM_029614	Prss23	Mus musculu	chr7:9665771	7.11427732	-1.761124	0.29501823	-2.1611867	0.2235723	0.40006266	1.31956522	6.66059021	8.42171421	6.26052755
A_30_P01018	chr2:36112819-36114110_	lincRNA:chr2:	chr2:3611314		6.74805883	2.8760914	7.34158416	0.39345064	1.31353135	2.48264076	5.58919598	8.53430288	5.65821148	6.05166212
A_55_P23408	BY714998	4930452L12R	BY714998 RIK	chr10:561560	8.33638404	0.76298839	1.69700214	2.39921502	5.2751606	-1.6362266	0.32169677	8.04530462	7.28231624	9.68153126

A_55_P21456	NM_176830	1110036O03I	Mus musculu	chr11:100270	10.1908198	-2.533498	0.1727194	-0.759199	0.59082428	-1.774299	0.29233632	8.7548875	11.2883855	10.5291865
A_52_P31543	NM_007570	Btg2	Mus musculu	chr1:1359721	9.45158226	-2.834638	0.14018093	-1.8815318	0.2713954	-0.9531062	0.51651917	8.18900087	11.0236389	9.14210706
A_51_P40244	NM_146564	Olfr836	Mus musculu	chr9:1892628	6.82612356	1.07972719	2.11363636	3.02034865	8.11363636	-1.9406215	0.2605042	6.53915881	5.45943162	8.47978026
A_55_P19715	NM_007529	Bcan	Mus musculu	chr3:8779151	8.23175903	-3.222533	0.10713242	-2.5479096	0.17100263	-0.6746234	0.62649573	6.93270689	10.1552399	7.60733031
A_55_P21377	NM_0011777	Gm13138	Mus musculu	chr4:1458894	8.47233738	-1.3414055	0.39463602	-2.0801879	0.23648361	0.73878243	1.66876688	8.27146303	9.6128685	7.5326806
A_52_P45093	NM_198414	Paqr9	Mus musculu	chr9:9546240	8.63150188	2.15090359	4.44105854	0.64575231	1.56455493	1.50515128	2.83854434	9.85018684	7.69928325	8.34503556
A_52_P49462	NM_013613	Nr4a2	Mus musculu	chr2:5696075	11.355886	1.011624	2.01617937	-1.0042335	0.49853494	2.01585748	4.04420878	12.3650465	11.3534225	10.3491891
A_55_P20029	NM_022315	Smoc2	Mus musculu	chr17:145416	7.18578522	-2.253487	0.2097166	-2.2451558	0.21093117	-0.0083313	0.99424184	6.43184579	8.68533283	6.44017706
A_51_P50094	NM_133919	Aff1	Mus musculu	chr5:1042767	8.01088585	-3.0945176	0.11707317	-2.5784424	0.1674216	-0.5160752	0.69927159	6.80735492	9.90187252	7.32343012
A_55_P19806	NM_011497	Aurka	Mus musculu	chr2:1721817	8.96804808	-1.2256928	0.42759212	-2.4359294	0.18480434	1.2102366	2.3137558	8.96289601	10.1885888	7.7526594
A_51_P39389	NM_025837	Mpi	Mus musculu	chr9:5739244	8.46412805	2.07148131	4.20318021	-1.1130719	0.4623086	3.18455321	9.09171975	10.2161396	8.14465824	7.03158634
A_30_P01028	chr8:91569369-91594119_	lincRNA:chr8:	chr8:9157356		6.6957481	-2.0582697	0.24010383	-1.868552	0.27384815	-0.1897177	0.87677725	5.94641896	8.00468865	6.13613669
A_55_P19571	XM_925521	6430503K07I	PREDICTED: I	chr2:1470141	7.82113459	-3.6106928	0.08186027	-1.8139733	0.28440657	-1.7967195	0.28782834	6.01866384	9.62935662	7.8153833
A_30_P01025	H19		lincRNA:chr7:	chr7:1497618	10.3867373	-4.8302755	0.03515136	-2.114515	0.23092319	-2.7157605	0.15222102	7.87139191	12.7016675	10.5871524
A_55_P19888	ENSMUST00000109514		B-cell CLL/lyn	chr11:240683	11.9082087	2.99687465	7.98268815	1.41657598	2.6695119	1.58029867	2.9903175	13.4339331	10.4370585	11.8536345
A_51_P30543	NM_009037	Rcn1	Mus musculu	chr2:1052272	10.6805135	-4.2295965	0.05330459	-1.9002191	0.26790268	-2.3293774	0.19896997	8.4941889	12.7237854	10.8235663
A_52_P40802	NM_0011436	Mpped2	Mus musculu	chr2:1067072	8.46531502	-3.1979958	0.1089701	1.68218503	3.20913621	-4.8801808	0.03395621	5.7725895	8.97058527	10.6527703
A_55_P20468	NM_009242	Sparc	Mus musculu	chr11:552089	12.5250513	-5.6280946	0.0202197	-1.4684943	0.36135925	-4.1596003	0.05595457	9.2624864	14.890581	13.4220867
A_66_P12632	ENSMUST00000114915		RIKEN cDNA 9	chr2:9804803	8.71250871	-2.7485095	0.14880455	-0.9975899	0.50083598	-1.7509196	0.29711234	7.21269903	9.9612085	8.96361862
A_55_P20714	ENSMUST000	Pde3a	phosphodiest	chr6:1414487	8.32639632	3.79002748	13.8328592	0.57190635	1.48648649	3.21812113	9.30574163	10.6624459	6.87241838	7.44432473
A_55_P20166	NM_007659	Cdk1	Mus musculu	chr10:688011	8.55230243	-0.1085878	0.92749548	-3.3245383	0.09981924	3.21595042	9.2917505	9.58808996	9.6966778	6.37213954
A_55_P20381	NM_0010099	Dock11	Mus musculu	chrX:3361616	9.50602466	2.01447366	4.04033149	1.79316587	3.46574586	0.2213078	1.16578989	10.2512851	8.23681148	10.0299773
A_30_P01023	chr4:146156575-14617542	lincRNA:chr4:	chr4:1461576		7.52893031	-1.1792871	0.44156964	-2.0168958	0.24708926	0.83760867	1.78708551	7.4150375	8.5943246	6.57742883
A_66_P10211	XM_0014747	LOC1000457	PREDICTED: I	chr2:1626193	7.04748932	0.79711574	1.73762376	2.12560528	4.36386139	-1.3284895	0.39818491	6.87036472	6.07324898	8.19885426
A_30_P01028	chr3:93159107-93159613_	lincRNA:chr3:	chr3:9315943		7.6792782	0.39432726	1.31432974	2.37217085	5.17719569	-1.9778436	0.25386905	7.15143943	6.75711217	9.12928302
A_55_P19975	NM_183248	Nkx6-2	Mus musculu	chr7:1467671	8.5822923	-4.3807668	0.04800183	-3.1573933	0.11208046	-1.2233735	0.42828008	6.71424552	11.0950123	7.93761903
A_55_P20330	NM_026422	Mrrf	Mus musculu	chr2:3604536	9.9918927	2.38794467	5.23411154	0.5708598	1.48540856	1.81708487	3.52368478	11.3935692	9.00562455	9.57648435
A_55_P21788	ENSMUST00000103456		PREDICTED: I	chr12:115045	7.22206419	0.86359376	1.81956522	2.02101616	4.05869565	-1.1574224	0.4483128	7.12412131	6.26052755	8.28154371
A_52_P33456	NM_009504	Vdr	Mus musculu	chr15:976851	6.83733241	0.36627167	1.28901734	2.59672837	6.04913295	-2.2304567	0.21309126	6.2159374	5.84966573	8.4463941
A_55_P21528	XR_001688	Gm5223	PREDICTED: I	chr17:169197	11.830479	-2.0934279	0.23432326	-0.3414031	0.78927333	-1.7520248	0.29688481	10.5486614	12.6420893	12.3006862
A_55_P24259	AK079380	2810468N07I	Mus musculu	chr17:257119	7.70783646	-2.7987268	0.14371407	-2.2213111	0.21444639	-0.5774157	0.67016317	6.58245565	9.38118241	7.15987134
A_55_P19690	NM_011535	Tbx3	Mus musculu	chr5:1201345	7.59136575	-2.1294867	0.22853916	-1.4666468	0.36182229	-0.6628399	0.63163371	6.66059021	8.79007693	7.32343012
A_52_P22081	NM_144551	Trib2	Mus musculu	chr12:157990	11.3432942	2.33593288	5.04877323	1.30288163	2.4672119	1.03305125	2.04634764	12.4662889	10.1303561	11.4332377
A_55_P22462	NM_022314	Tpm3	Mus musculu	chr3:8987766	7.62392458	0.62621853	1.54351396	2.24954747	4.75533662	-1.6233289	0.32458564	7.29155445	6.66533592	8.91488339
A_55_P21093	NM_009599	Ache	Mus musculu	chr5:1377331	10.338191	-2.5373254	0.17226178	-0.2370287	0.84849101	-2.3002967	0.20302134	8.72565028	11.2629757	11.025947
A_55_P20492	NR_003517	Pisd-ps1	Mus musculu	chr11:303178	13.2194914	-1.9853735	0.25254748	0.73771367	1.66753111	-2.7230871	0.15144994	11.6500046	13.635378	14.3730917
A_55_P19672	NM_144818	Ncaph	Mus musculu	chr2:1269296	8.27485603	-0.5617606	0.67747489	-2.1596126	0.22381636	1.59785201	3.02692308	8.62021983	9.18198044	7.02236781
A_30_P01025	chr2:136327645-13634125	lincRNA:chr2:	chr2:1363406		6.19176792	0.37538842	1.29718876	2.07479703	4.21285141	-1.6994086	0.3079123	5.75042785	5.37503943	7.44983646
A_30_P01027	chr15:62046023-62082530	lincRNA:chr15	chr15:620824		7.52510599	-2.016431	0.24716888	-0.6172871	0.65189562	-1.3991439	0.37915408	6.38658105	8.40301202	7.78572491
A_52_P28917	ENSMUST00000065087		Riken cDNA C	chr2:3350160	8.28868443	-4.7043254	0.03835809	-1.8603661	0.27540639	-2.8439593	0.13927813	5.7725895	10.4769149	8.61654884
A_55_P20843	NM_010917	Nid1	Mus musculu	chr13:135301	9.13706559	-4.543978	0.04286732	-2.033934	0.24418831	-2.510044	0.17555025	6.78572491	11.3297029	9.29576893
A_55_P20636	NR_033493	Gm10818	Mus musculu	chr16:326664	8.88866301	0.22976556	1.17264438	2.13949537	4.40607903	-1.9097298	0.26614238	8.32867493	8.09890937	10.2384047
A_55_P20079	NM_009987	Cx3cr1	Mus musculu	chr9:1199578	6.51893284	2.10035053	4.28813559	-0.1914811	0.87570621	2.29183167	4.89677419	7.98299357	5.88264305	5.6911619
A_30_P01019	chr12:111617728-1116654	lincRNA:chr1:	chr12:111621		9.06294395	0.34924754	1.27389603	2.1796588	4.53046395	-1.8304113	0.28118445	8.56922271	8.21997517	10.399634

A_66_P11622	NM_0010043	5730494M16	Mus musculu	chr18:252968	8.77343522	1.99485009	3.98574686	-0.248956	0.84150513	2.24380611	4.73644986	10.1863206	8.19147053	7.94251451
A_52_P28547	NM_001081(	Lrp2	Mus musculu	chr2:6926262	7.8121336	-2.4803575	0.1792	-1.9463789	0.25946667	-0.5339786	0.69064748	6.80735492	9.28771238	7.34133349
A_52_P51509	NM_025326	Tmem176a	Mus musculu	chr6:4879404	10.0091216	-2.6353962	0.160941	-0.7089564	0.61176251	-1.9264398	0.26307758	8.48850965	11.1239058	10.4149494
A_55_P21074	ENSMUST00000068970		RIKEN cDNA 1	chr4:4164477	7.27809262	0.41090961	1.32952381	2.0697349	4.19809524	-1.6588253	0.31669691	6.86212073	6.45121111	8.52094601
A_55_P21332	NM_0010133	E130306D19I	Mus musculu	chr4:4350907	8.32732331	-1.8655536	0.27441788	-3.3284812	0.0995468	1.46292757	2.7566719	8.19311463	10.0586683	6.73018706
A_30_P01019	chr14:27176652-27227602		lincRNA:chr1	chr14:272027	6.77152529	0.45125769	1.36723164	2.21538903	4.6440678	-1.7641313	0.29440389	6.33390074	5.88264305	8.09803208
A_30_P01026	chr15:92174226-92201201		lincRNA:chr1!	chr15:921963	8.01764943	-2.1891343	0.21928298	-0.4680568	0.7229377	-1.7210775	0.3033221	6.71424552	8.90337978	8.435323
A_52_P63086	NM_0010333	Abcc4	Mus musculu	chr14:118882	8.80672227	-2.7470413	0.14895606	-2.7765162	0.1459437	0.02947489	1.02064057	7.90086681	10.6479081	7.87139191
A_51_P32720	NM_011839	Mab21l2	Mus musculu	chr3:8635034	9.44076548	-4.2212673	0.05361322	-0.9183588	0.52911059	-3.3029085	0.10132707	6.93270689	11.1539742	10.2356154
A_30_P01019	chr12:33808493-33829143		lincRNA:chr1	chr12:338272	7.65752547	-2.0114048	0.2480315	-1.1929697	0.43740157	-0.8184351	0.56705671	6.71424552	8.72565028	7.5326806
A_55_P19643	NM_023608	Gdpd2	Mus musculu	chrX:9793416	9.4286739	-4.7597423	0.03691261	-2.8715601	0.13663888	-1.8881823	0.27014722	7.21269903	11.9724414	9.10088131
A_55_P21582	NM_009640	Angpt1	Mus musculu	chr15:422563	6.85818404	2.34015185	5.06355932	-0.6586414	0.63347458	2.99879323	7.99331104	8.6378324	6.29768055	5.63903917
A_30_P01029	chr5:96591892-96606467_		lincRNA:chr5	chr5:9660311	9.79724197	-2.8433108	0.13934075	-1.3344369	0.3965468	-1.5088739	0.35138539	8.34651373	11.1898246	9.85538762
A_66_P12215	NR_003518	Pisd-ps3	Mus musculu	chr11:00303(	12.5698881	-3.1627998	0.11166122	0.45052275	1.36653532	-3.6133226	0.08171119	10.3111807	13.4739805	13.9245033
A_52_P64113	NM_011440	Sox14	Mus musculu	chr9:9977568	8.14132978	-2.746631	0.14899842	-1.4264474	0.37204591	-1.3201836	0.40048397	6.78572491	9.53235593	8.10590851
A_55_P19584	XR_031621	Gm9252	PREDICTED: I	chr7:4892202	7.73787106	-2.0306968	0.24473684	-2.2201444	0.21461988	0.18944756	1.14032698	7.12412131	9.15481811	6.93467375
A_30_P01027	chr2:33496712-33501183_		lincRNA:chr2	chr2:3350090	8.59369776	-2.0437739	0.24252849	-0.970221	0.51042786	-1.0735529	0.47514743	7.55458885	9.59836272	8.62814172
A_66_P11511	NM_133784	Wwtr1	Mus musculu	chr3:5726005	7.05692158	-2.1055016	0.23237043	-2.571542	0.1682243	0.4660404	1.38131313	6.51043452	8.6159361	6.04439412
A_30_P01025	chr17:41231055-41249764		lincRNA:chr1	chr17:41239(	11.2972246	-3.1376638	0.11362374	-0.4015337	0.75705306	-2.7361301	0.15008689	9.3392933	12.4769571	12.0754234
A_55_P21111	NM_009789	S100g	Mus musculu	chrX:1593999	8.76594898	-3.8991063	0.06702735	-0.1481777	0.90238954	-3.7509285	0.07427763	6.2159374	10.1150437	9.9668659
A_55_P19537	NM_007726	Cnr1	Mus musculu	chr4:3403552	10.181615	2.12287585	4.35561324	0.44211897	1.35859831	1.68075689	3.20596102	11.4494926	9.32661674	9.76873571
A_51_P46064	NM_0010798	Hoxb3	Mus musculu	chr11:962071	8.16251481	-3.5896001	0.08306589	-1.2808474	0.41155371	-2.3087527	0.20183486	6.19639721	9.78599729	8.50514992
A_51_P33410	NM_007833	Dcn	Mus musculu	chr10:969802	7.36156362	-2.4781082	0.1794796	-2.8532962	0.13837966	0.37518799	1.29700855	6.66059021	9.13869844	6.28540222
A_55_P20958	ENSMUST000	Nfix	nuclear facto	chr8:8722881	7.60698007	-1.2643833	0.41627726	-2.1390774	0.22702492	0.87469406	1.83361921	7.47708364	8.74146699	6.60238957
A_52_P11353	NR_001463	Xist	Mus musculu	chrX:1006563	7.49814024	6.15785217	71.4	-0.296393	0.81428571	6.45424517	87.6842105	11.7021727	5.54432052	5.24792751
A_30_P01024	chr7:133402766-13341551		lincRNA:chr7	chr7:1334039	7.02169637	0.63154464	1.5492228	2.41106087	5.31865285	-1.7795162	0.29128105	6.63903917	6.00749454	8.41855541
A_55_P19949	NM_008252	Hmgb2	Mus musculu	chr8:5999292	9.86401582	-0.5048429	0.70473713	-3.0414122	0.12146291	2.53656934	5.80207657	10.541258	11.0461008	8.00468865
A_55_P20656	NM_172301	Ccnb1	Mus musculu	chr13:101548	8.58576059	-1.2621408	0.41692482	-3.3019016	0.10139781	2.03976079	4.11177347	8.84496724	10.1071081	6.80520646

Supplementary Table S10: Primer sequences used for lentiviral construct

Fragment	Primers
Dlxl12b-βglobin	Forward: 5'-CTCT**GGATCC**ACACAGCTTAATGATTATC-3'
	Reverse: 5'-GAGA**ACCGGT**CGCCGCGCTCTGCTTCTGG-3'
692	Forward: 5'-ACAA**GGATCC**CACATCTCAGTGGCTCAT-3'
	Reverse: 5'-TCTA**ACCGGT**CAGGGTGTCTGTGTTGATG-3'
1056	Forward: 5'-GACA**GGATCC**GTCCCTCACAGAACTCAG-3'
	Reverse: 5'-GACA**ACCGGT**GATGCCTGCCTTGAAGTC-3'
1538	Forward: 5'-TCTA**GGATCC**TGCTGCCTCAAACAAGAATG-3'
	Reverse: 5'-AGTT**ACCGGT**TTGGATGAGGGAAAGACCTG-3'
Lhx6 enhancer/promoter	Forward: 5'-GAGA**AGATCT**CAGCCTTTAGAAGCTGGTG-3'
	Reverse: 5'-GAAT**ACCGGT**CCCTGGCTGGGCCATCACC-3'
Hsp68	Forward: 5'-GAGA**ACCGGT**GCATCGGCGCGCCGACC-3'
	Reverse: 5'-ATAT**TCCGGA**GGCGCCGCGCTCTGCTTC-3'
βglobin	Forward: 5'-CTAT**ACCGGT**AGCCCGGGCTGGGCATAA-3'
	Reverse: 5'-GAGA**ACCGGT**CGCCGCGCTCTGCTTCTGG-3'
