# Supplementary material for: Use of “MGE Enhancers” for Labeling and Selection of Embryonic Stem Cell-Derived Medial Ganglionic Eminence (MGE) Progenitors and Neurons
Source: PLoS One. 2013 May 1;8(5):e61956. doi: 10.1371/journal.pone.0061956 (PMC3641041; doi:10.1371/journal.pone.0061956)
Supplement: File S3 — Figure S12–S14. Figure S12: Unsupervised clustering showing 1000 most variable probes.Microarray comparison of RNA expression from primary E12.5 MGE Lhx6-GFP+ cells, ES-Lhx6-GFP+ and ES-Lhx6-GFP− cells. Show here are 1000 most variable probes. Figure S13: Transplanted Lhx6-GFP+ cells express cortical interneuron markers in the cortex. (A–C) Four days after transplantation, some of the Lhx6-GFP+ cells were also GABA+ (A), Calbindin+ (B), or MafB+ (C). White arrows indicate double positive cells. (D–F”) Sixty-nine days after transplantation, Lhx6-GFP+ cells expressed parvalbumin (PV) (D, D′, D″), somatostatin (SST) (E, E′, E″) and neuropeptide Y (NPY) (F, F′, F″). Arrows indicate markers co-labeling. In PV/GFP co-staining, there were some GFP+ cells that have weak PV expression (white arrowheads). Scale bar, 100 µm. (G) Average (data are mean ± SEM) percentages of parvalbumin+ (PV+/GFP+), somatostain+ (SST+/GFP+), neuropeptide Y+ (NPY+/GFP+) cells among all Lhx6-GFP+ cells (n = 3–4). Figure S14: Test of lentiviral constructs in dissociated primary MGE cells. Dissociated primary MGE cells (E13.5) were infected with each of the lentiviruses indicated (A: DlxI12b-mCherry, B: DlxI12b-hsp68-mCherry, C: DlxI12b-βg-mCherry, D:692-mCherry, E: 692-hsp68-mCherry, F:692-βg-mCherry, G: Lhx6-E/P-mCherry) for three days before being fixed for immunostaining. Pictures are composites from several different fields (A–F) or from one single field (G). Shown here are Nkx2-1 staining in green, mCherry in red, and DAPI nuclear stain in blue. Scale bar, 50 µm. (PDF) [file pone.0061956.s003.pdf]

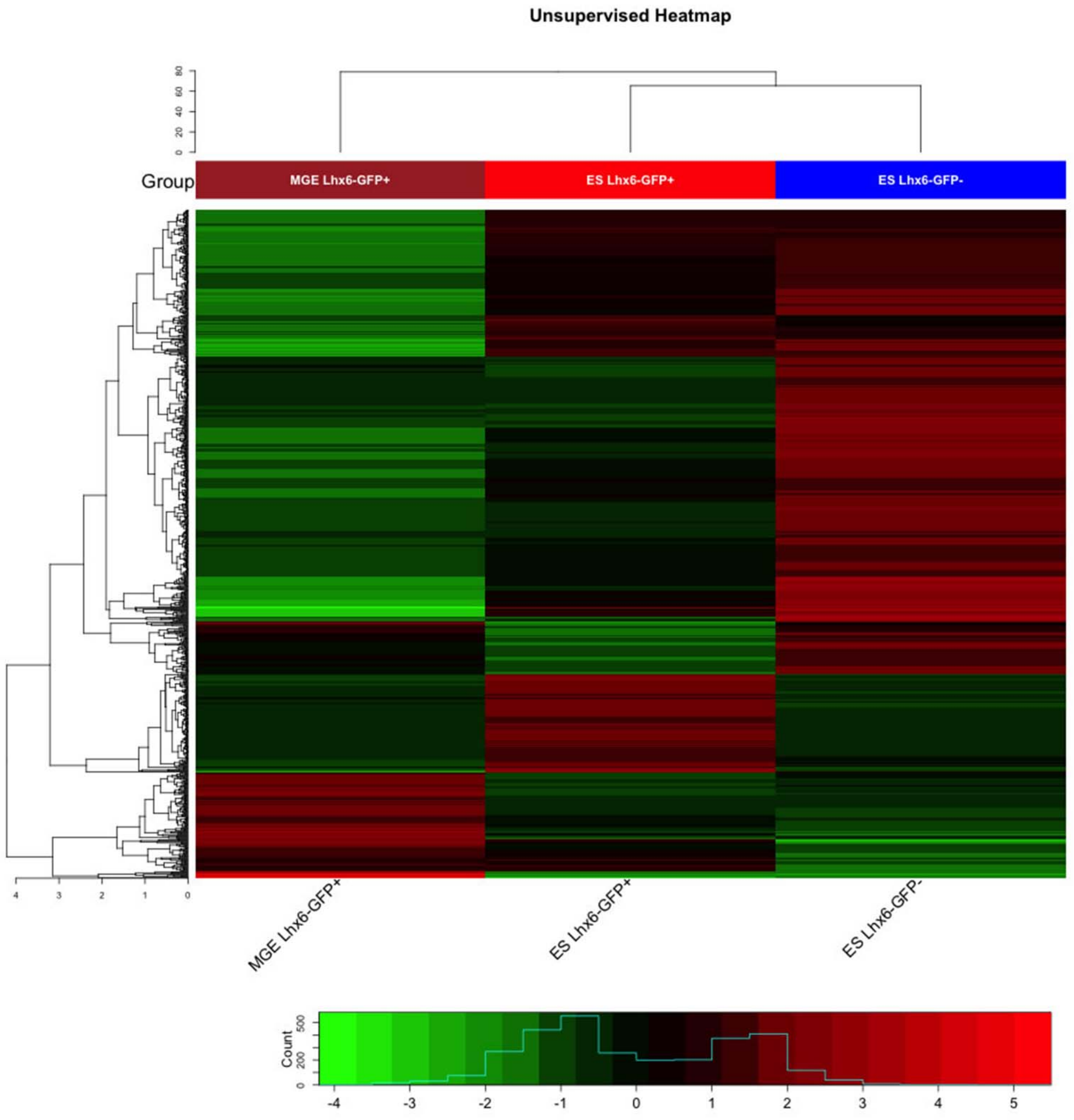

Figure S12: Unsupervised clustering showing 1000 most variable probes .
Microarray comparison of RNA expression from primary E12.5 MGE Lhx6-GFP^{+} cells, ES-Lhx6-GFP^{+} and ES-Lhx6-GFP^{-} cells. Show here are 1000 most variable probes.

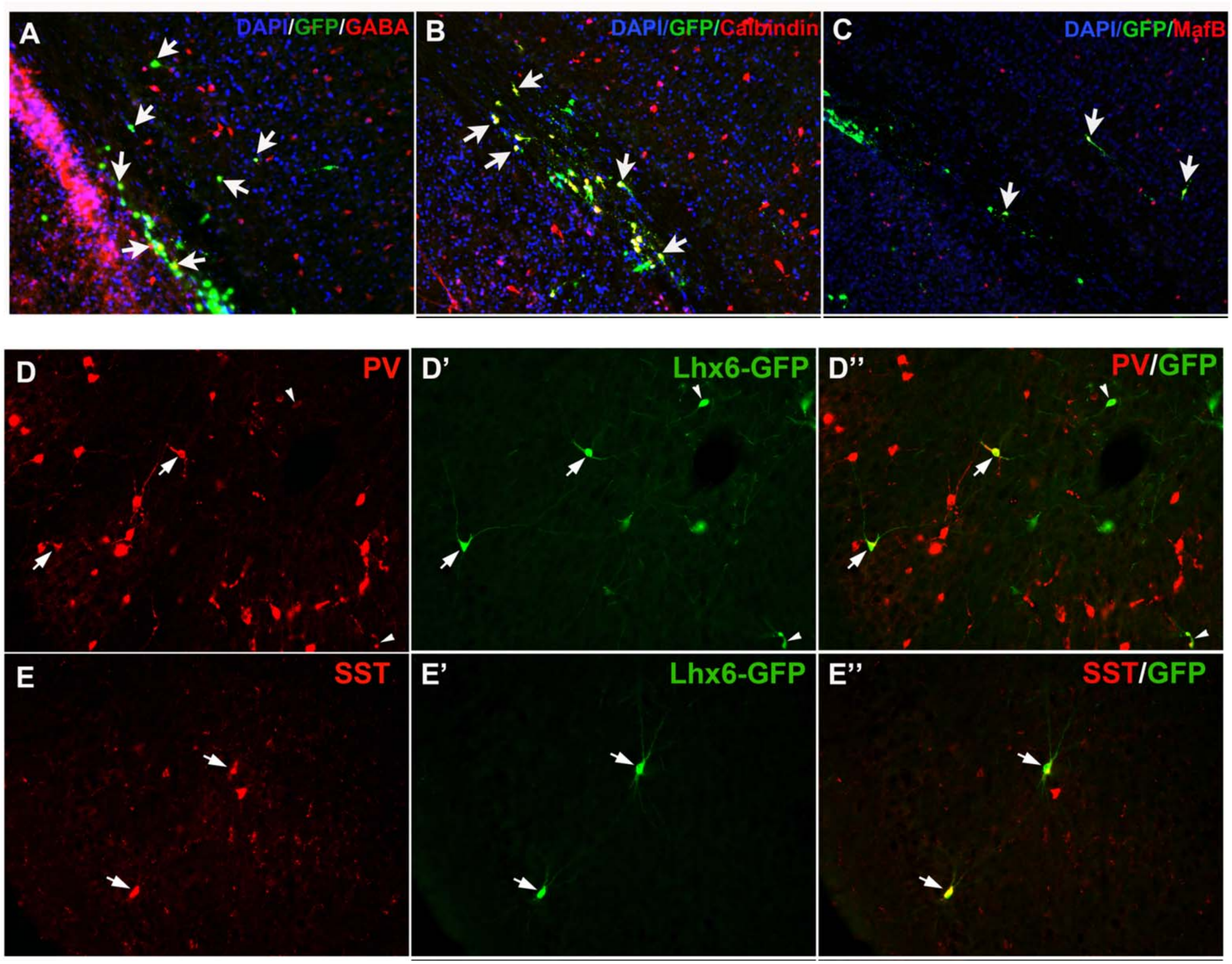
A
DAPI/GFP/GABA
B
DAPI/GFP/Calbindin
C
DAPI/GFP/MafB
D
PV
D'
Lhx6-GFP
D''
PV/GFP
E
SST
E'
Lhx6-GFP
E''
SST/GFP

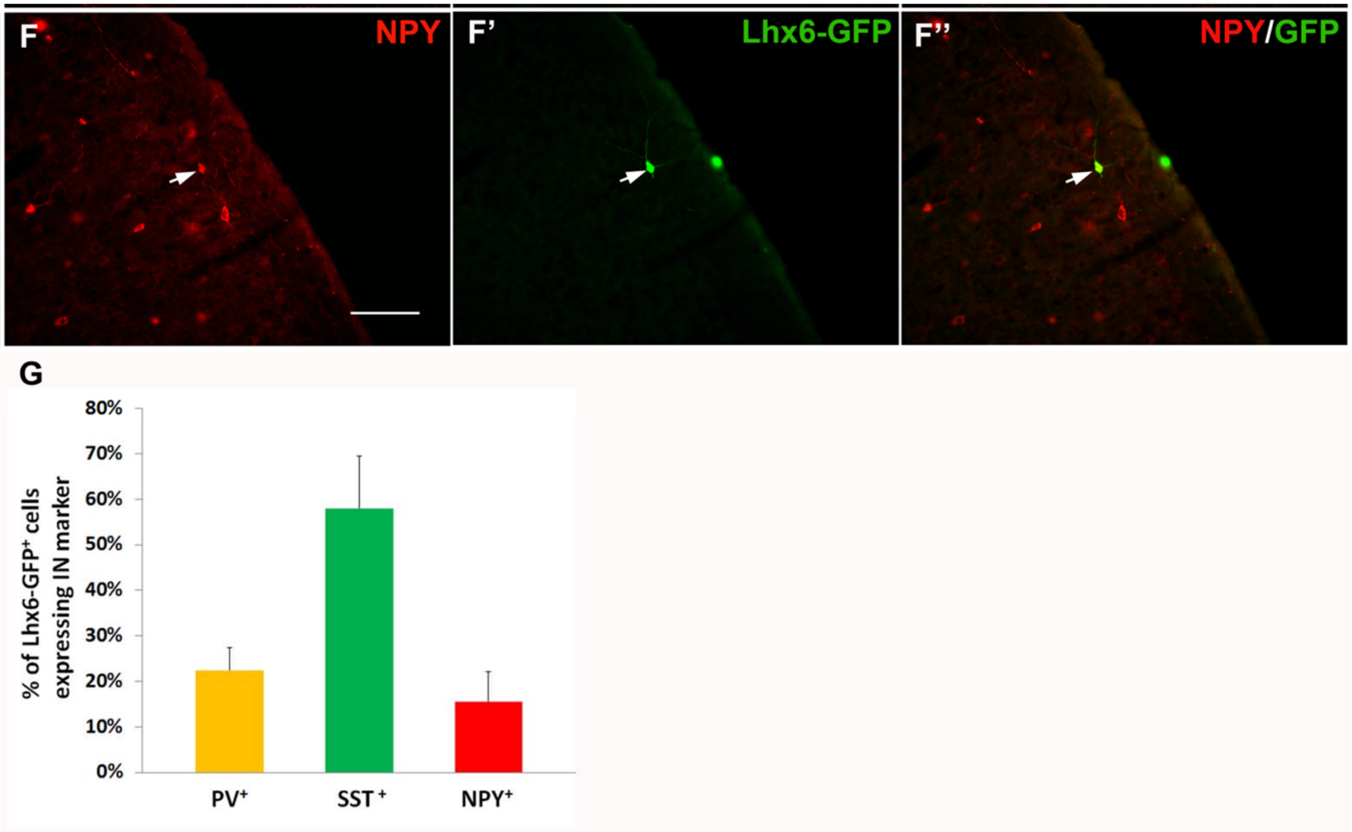

Figure S13: Transplanted Lhx6-GFP$^+$ cells express cortical interneuron markers in the cortex.
(A-C) Four days after transplantation, some of the Lhx6-GFP$^+$ cells were also GABA$^+$ (A), Calbindin$^+$ (B), or MafB$^+$ (C). White arrows indicate double positive cells. (D-F") Sixty-nine days after transplantation, Lhx6-GFP$^+$ cells expressed parvalbumin (PV) (D, D', D''), somatostatin (SST) (E, E', E'') and neuropeptide Y (NPY) (F, F', F''). Arrows indicate markers co-labeling. In PV/GFP co-staining, there were some GFP$^+$ cells that have weak PV expression (white arrowheads). Scale bar, 100µm. (G) Average (data are mean ± SEM) percentages of parvalbumin$^+$ (PV$^+$/GFP$^+$), somatostain$^+$ (SST$^+$/GFP$^+$), neuropeptide Y$^+$ (NPY$^+$/GFP$^+$) cells among all Lhx6-GFP$^+$ cells (n=3-4).

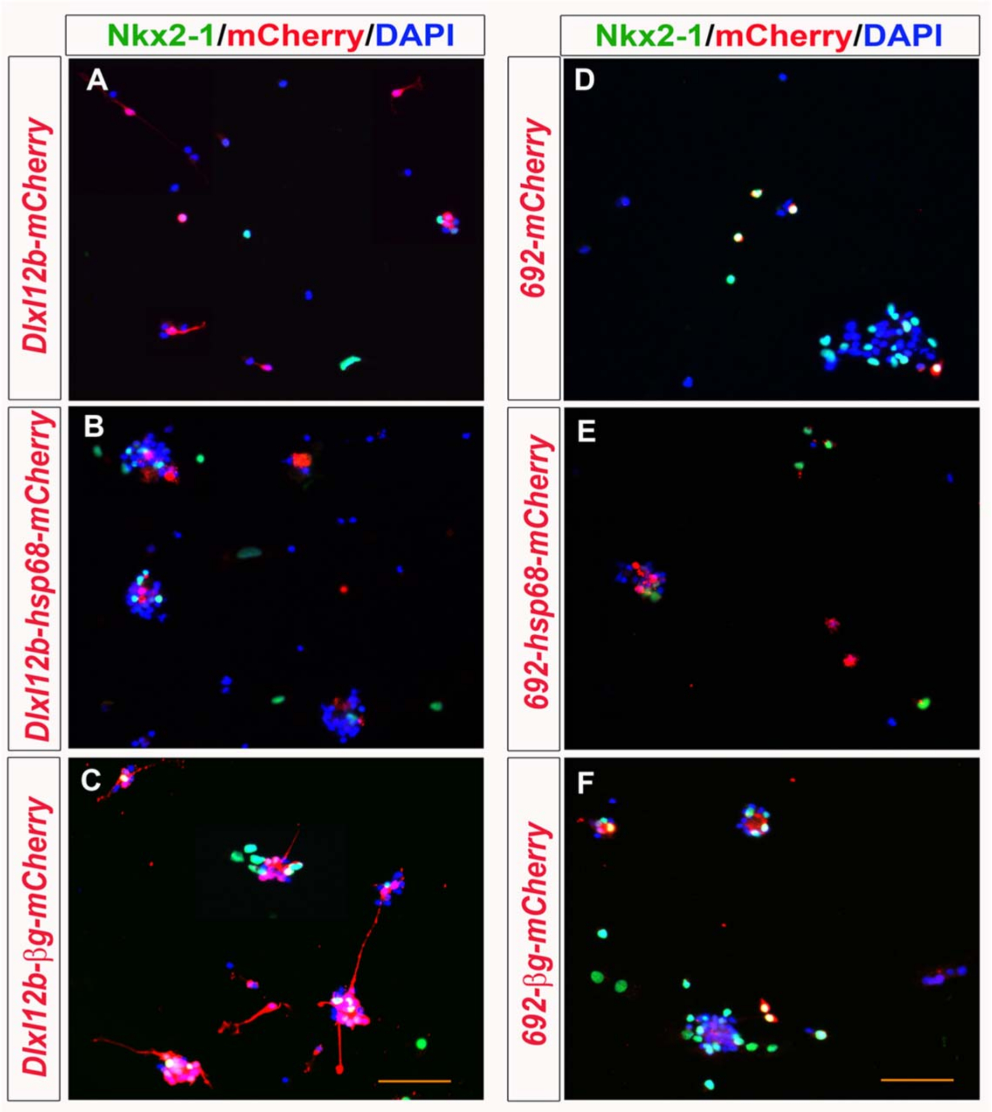

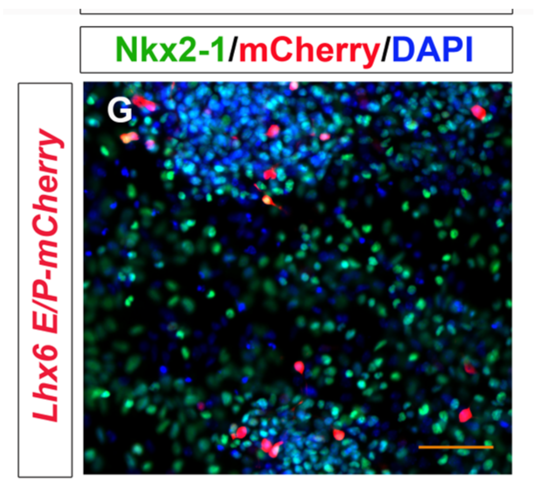

Figure S14: Test of lentiviral constructs in dissociated primary MGE cells.
Dissociated primary MGE cells (E13.5) were infected with each of the lentiviruses indicated (A: *DlxI12b-mCherry*, B: *DlxI12b-hsp68-mCherry*, C: *DlxI12b-βg-mCherry*, D:*692-mCherry*, E: *692-hsp68-mCherry*, F:*692-βg-mCherry, G*: *Lhx6-E/P-mCherry*) for three days before being fixed for immunostaining. Pictures are composites from several different fields (A-F) or from one single field (G). Shown here are Nkx2-1 staining in green, mCherry in red, and DAPI nuclear stain in blue. Scale bar, 50µm.
