# Supplementary material for: Use of “MGE Enhancers” for Labeling and Selection of Embryonic Stem Cell-Derived Medial Ganglionic Eminence (MGE) Progenitors and Neurons
Source: PLoS One. 2013 May 1;8(5):e61956. doi: 10.1371/journal.pone.0061956 (PMC3641041; doi:10.1371/journal.pone.0061956)
Supplement: File S4 — Figure S15–S19. Figure S15: Additional characterization of the enhancer DlxI12b. Mouse ES cell lines E14 (B-B″ & E-E″) and J14 (A-A″, C-D″ & F-F″) carrying enhancer DlxI12b-βg-mCherry were differentiated with our ES-MGE protocol. Expression of DlxI12b-βg-mCherry (red) was examined on D11, D13, and D15 together with other markers (shown in green): (A) Nkx2-1, (B) Dlx2, (C) Foxg1, (D) Islet1, (E) Olig2, (F) Calbindin. Scale bar, 200 µm. Figure S16: Additional characterization of the enhancer 692. (A–D) Mouse ES cell lines J14 carrying enhancer 692-βg-mCherry were differentiated with our ES-MGE protocol. Expression of 692-βg-mCherry (red) was examined together with Nkx2-1 (shown in green) on D9, D11, D13, and D15. Scale bar, 200 µm. (E–J) Mouse ES cell lines E14 carrying 692-mCherry were differentiated with our current MGE protocol. Expression of 692-mCherry (red) was examined with Nkx2-1 (E–H) and Mki67 (I, J) (shown in green) on days indicated. Scale bar, 100 µm. White arrows indicate co-labeling of respective markers shown. Figure S17: Additional characterization of the enhancer 692. Mouse ES cell lines J14 carrying enhancer 692-mCherry were differentiated with our ES-MGE protocol. Expression of 692-mCherry (red) was examined on D17 together with other markers (shown in green): (A) Nkx2-1, (B) Lhx6-GFP, (C) Mki67. White arrows indicate co-labeling of respective markers shown. Scale bar, 100 µm. Figure S18: Additional characterization of the enhancer 1056. Mouse ES cell line J14 carrying enhancer 1056-βg-mCherry were differentiated with our ES-MGE protocol. Expression of 1056-βg-mCherry (red) was examined on D9, 11, 13, 15 and 17 together with other markers (shown in green): (A–E) Lhx6-GFP, (F–J) Nkx2-1, (K-O) Mki67. Scale bar, 100 µm. Figure S19: Additional characterization of the enhancer 1538. Mouse ES cell line J14 carrying enhancer 1538-βg-mCherry were differentiated with our ES-MGE protocol. Expression of 1538-βg-mCherry (red) was examined on D10, 12, 14 and 16 [file pone.0061956.s004.pdf]

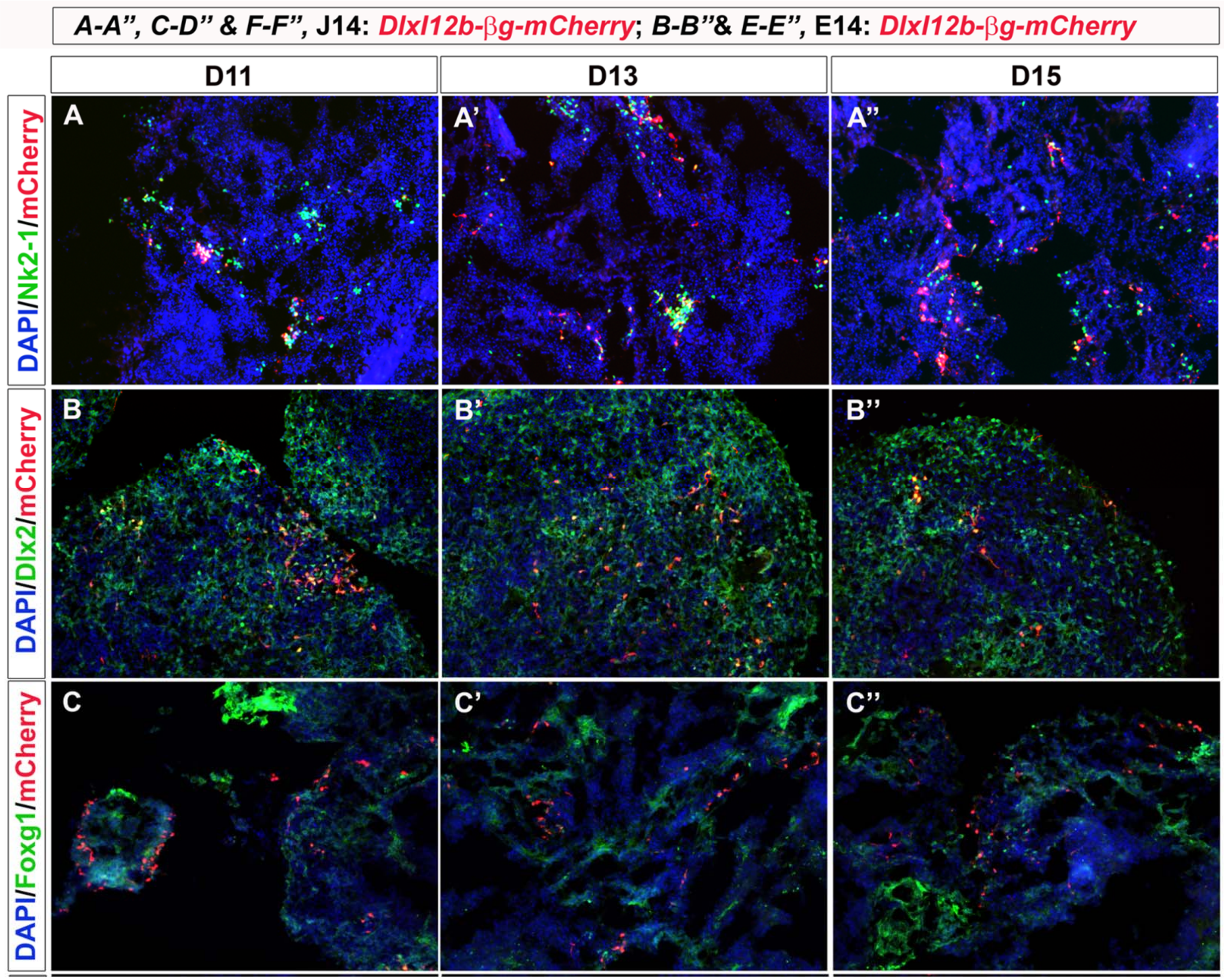
A-A", C-D" & F-F", J14: Dlxl12b-βg-mCherry; B-B"& E-E", E14: Dlxl12b-βg-mCherry
D11
D13
D15
DAPI/Nk2-1/mCherry
DAPI/Dlx2/mCherry
DAPI/Foxg1/mCherry
A
A'
A"
B
B'
B"
C
C'
C"

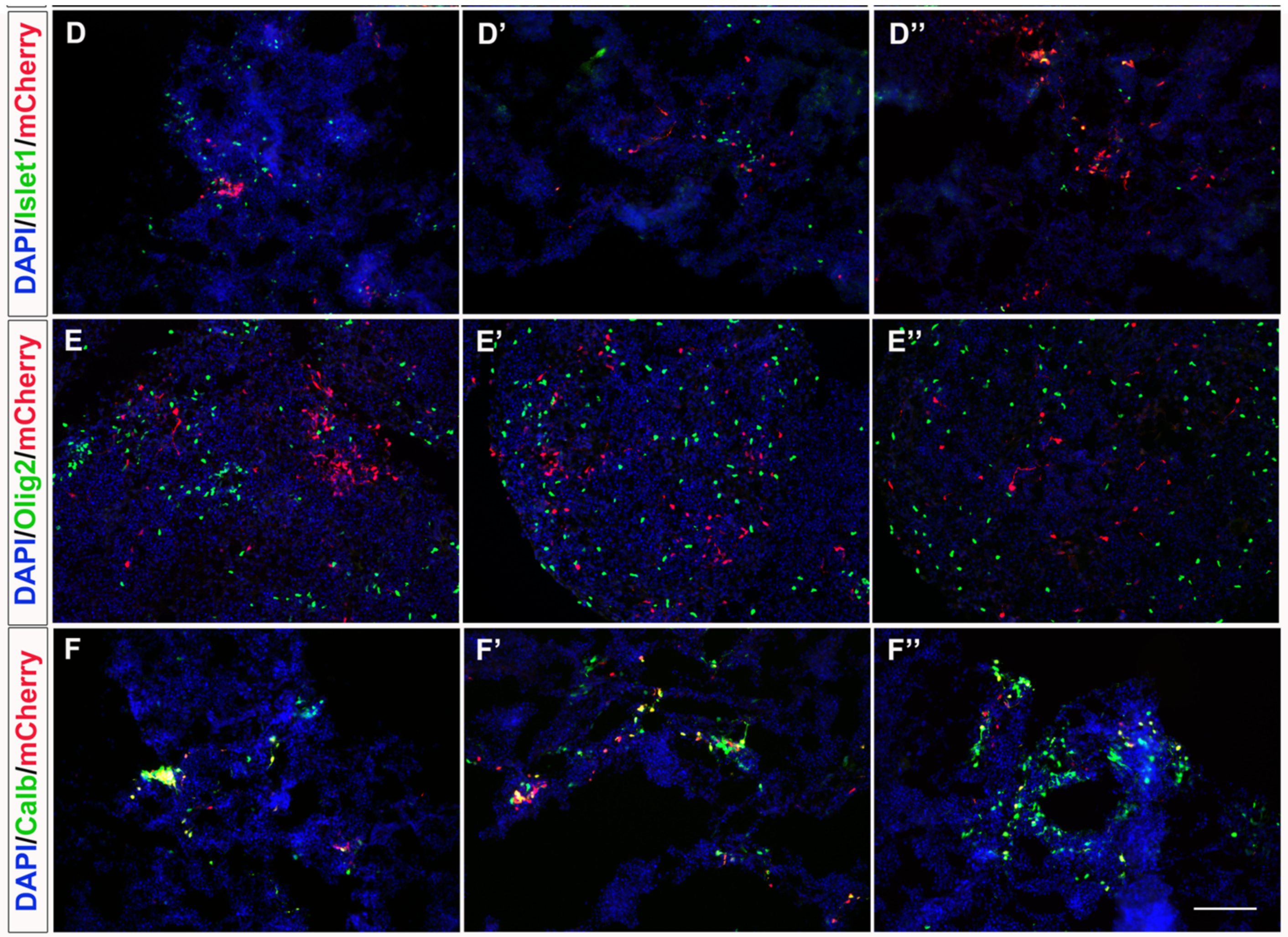


**Figure S15: Additional characterization of the enhancer *Dlxl12b***
Mouse ES cell lines E14 (B-B'' & E-E'') and J14 (A-A'', C-D'' & F-F'') carrying enhancer *Dlxl12b-βg-mCherry* were differentiated with our ES-MGE protocol. Expression of Dlxl12b-βg-mCherry (red) was examined on D11, D13, and D15 together with other markers (shown in green): (A) Nkx2-1, (B) Dlx2, (C) Foxg1, (D) Islet1, (E) Olig2, (F) Calbindin. Scale bar, 200μm.

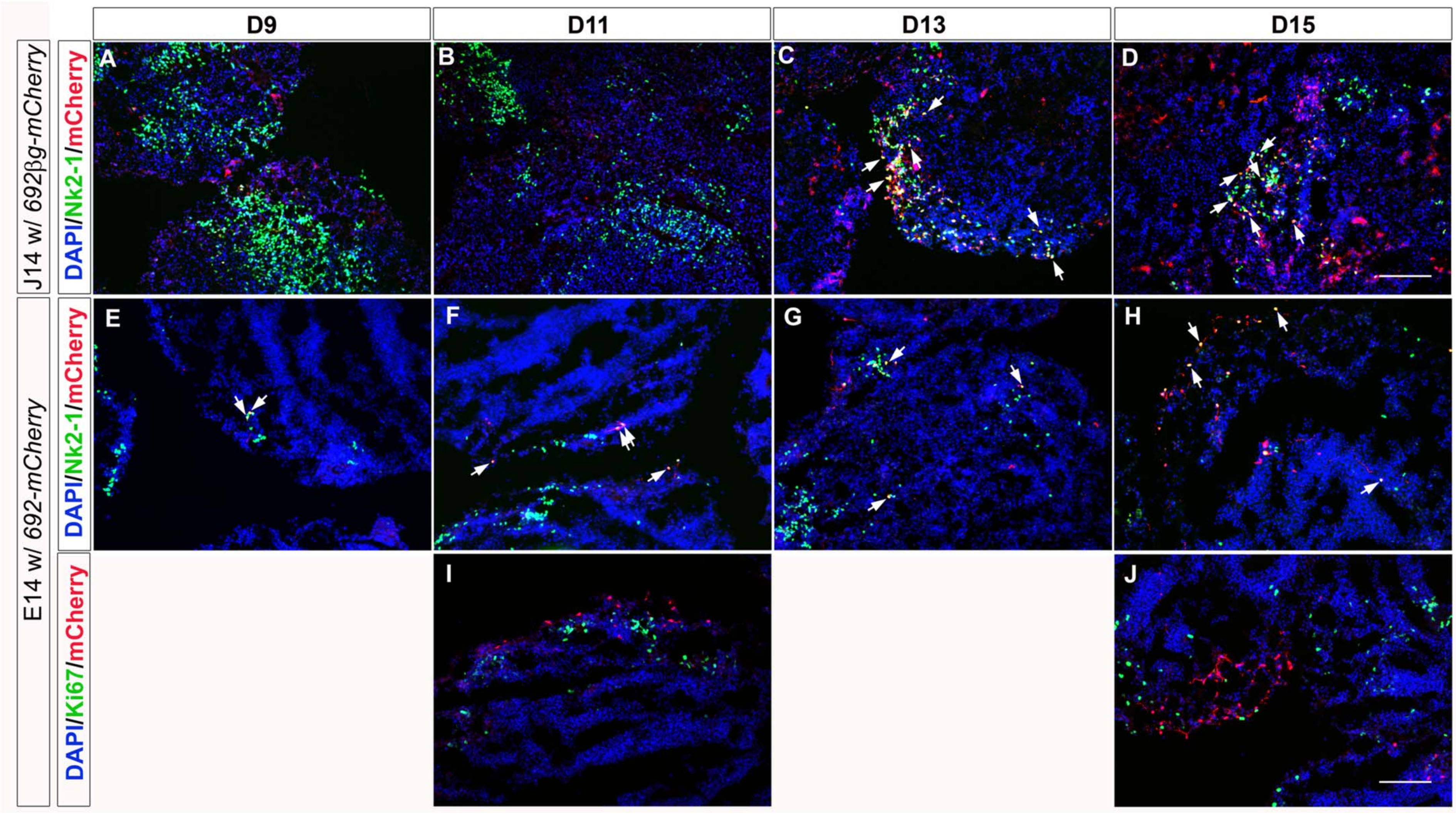


**Figure S16: Additional characterization of the enhancer *692***

(A-D) Mouse ES cell lines J14 carrying enhancer *692-βg-mCherry* were differentiated with our ES-MGE protocol. Expression of 692-βg-mCherry (red) was examined together with Nkx2-1 (shown in green) on D9, D11, D13, and D15. Scale bar, 200μm. (E-J) Mouse ES cell lines E14 carrying *692-mCherry* were differentiated with our current MGE protocol. Expression of 692-mCherry (red) was examined with Nkx2-1 (E-H) and Mki67 (I, J) (shown in green) on days indicated. Scale bar, 100μm. White arrows indicate co-labeling of respective markers shown.

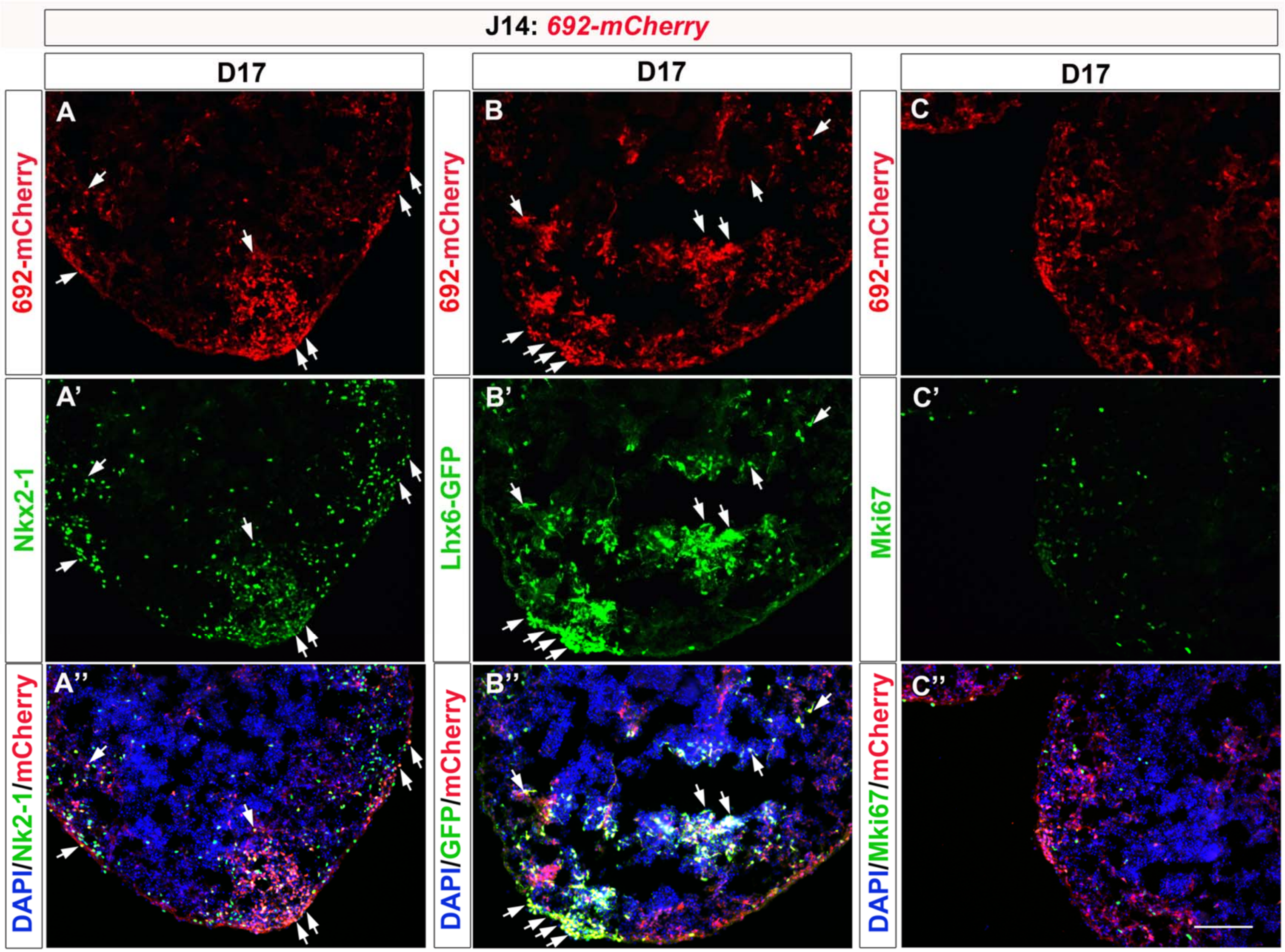


**Figure S17: Additional characterization of the enhancer *692***

Mouse ES cell lines J14 carrying enhancer *692-mCherry* were differentiated with our ES-MGE protocol. Expression of 692-mCherry (red) was examined on D17 together with other markers (shown in green): (A) Nkx2-1, (B) Lhx6-GFP, (C) Mki67. White arrows indicate co-labeling of respective markers shown. Scale bar, 100μm.

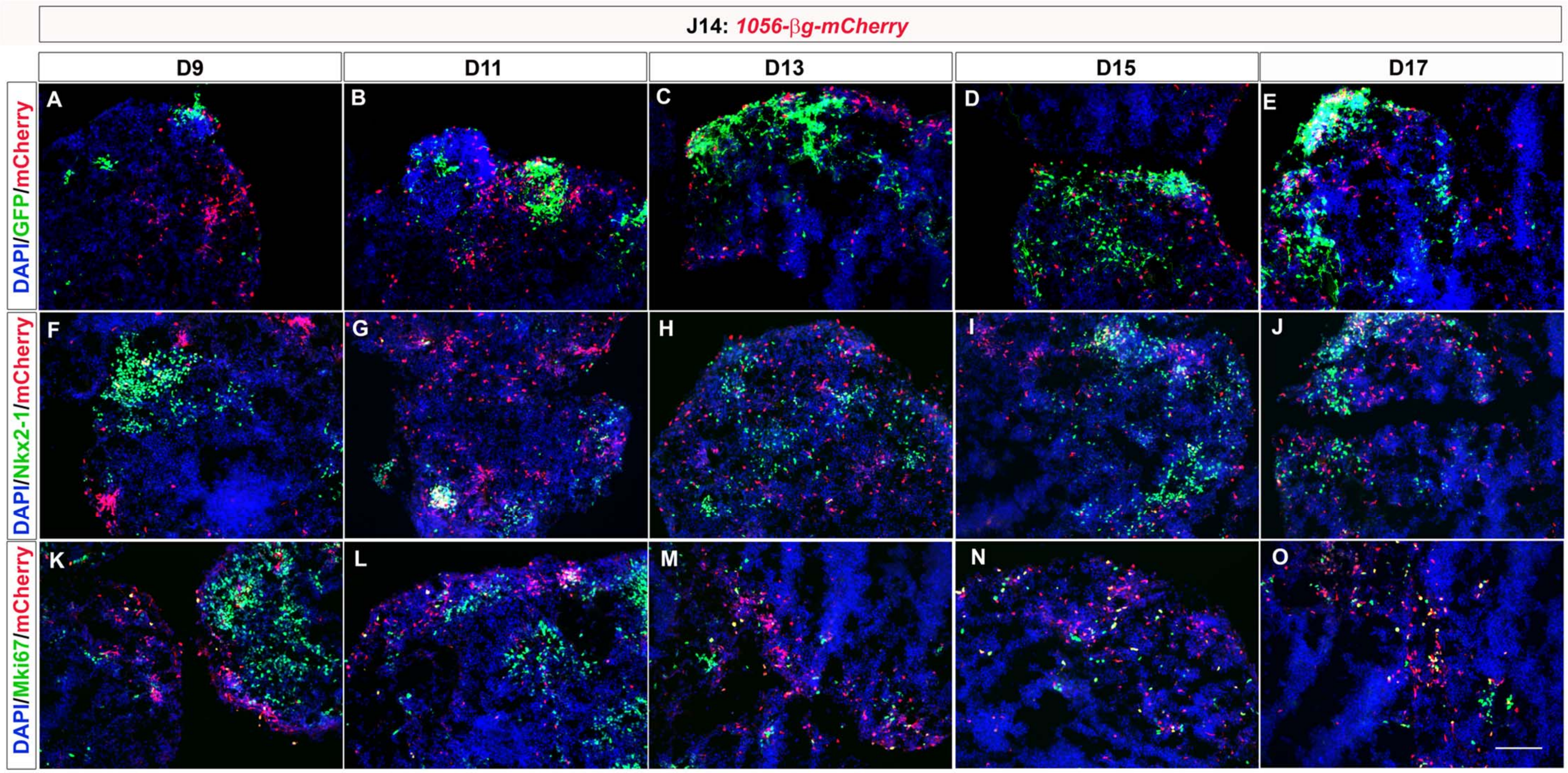


**Figure S18: Additional characterization of the enhancer *1056***

Mouse ES cell line J14 carrying enhancer *1056-βg-mCherry* were differentiated with our ES-MGE protocol. Expression of 1056-βg-mCherry (red) was examined on D9, 11, 13, 15 and 17 together with other markers (shown in green): (A-E) Lhx6-GFP, (F-J) Nkx2-1, (K-O) Mki67. Scale bar, 100µm.

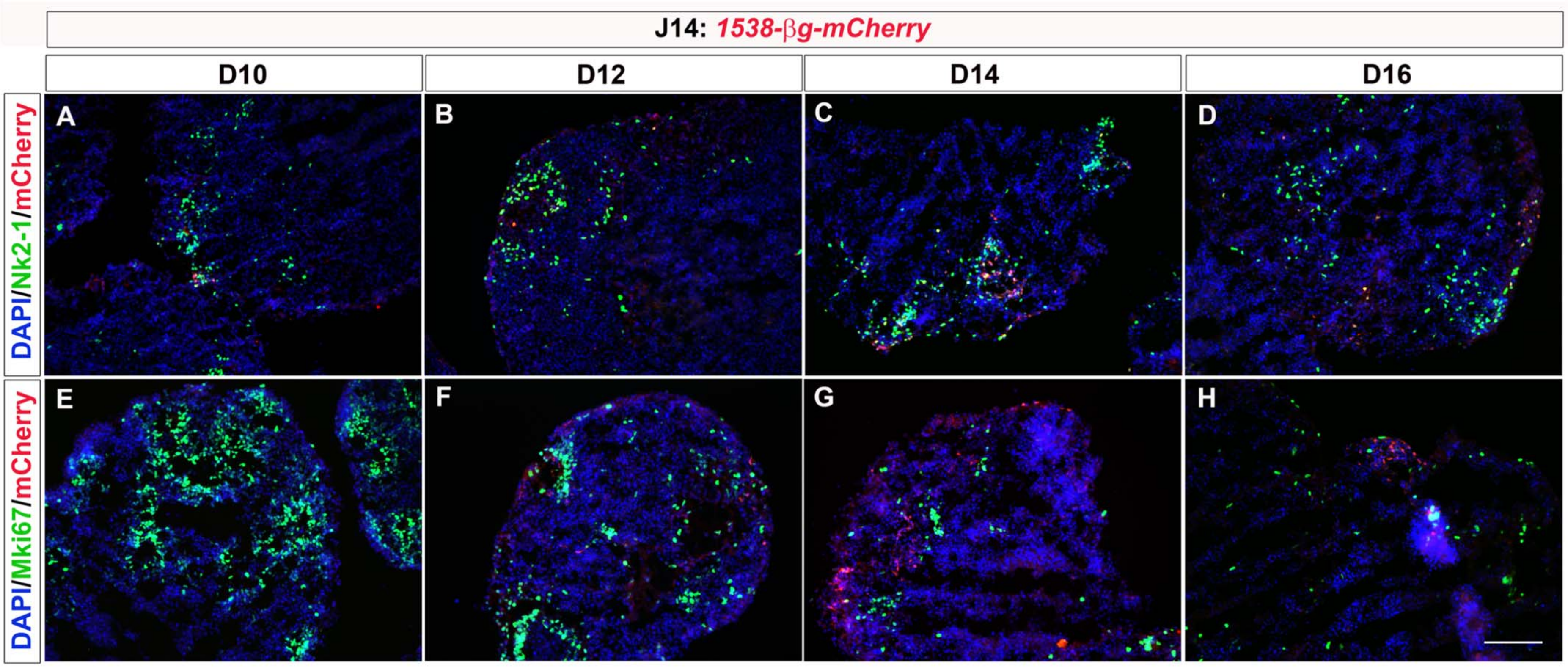


**Figure S19: Additional characterization of the enhancer *1538***
Mouse ES cell line J14 carrying enhancer *1538-βg-mCherry* were differentiated with our ES-MGE protocol. Expression of 1538-βg-mCherry (red) was examined on D10, 12, 14 and 16 together with other markers (shown in green): (A-D) Nkx2-1, (E-H) Mki67. Scale bar, 100μm.
