# Supplementary material for: Use of “MGE Enhancers” for Labeling and Selection of Embryonic Stem Cell-Derived Medial Ganglionic Eminence (MGE) Progenitors and Neurons
Source: PLoS One. 2013 May 1;8(5):e61956. doi: 10.1371/journal.pone.0061956 (PMC3641041; doi:10.1371/journal.pone.0061956)
Supplement: File S5 — Figure S20–S21. Figure S20: All of the DlxI12b-βg-mCherry+ cells express Lhx6-GFP thirty-three days after transplantation into the neocortex (white arrows in A-A″). About 28% of Lhx6-GFP+ cells are also DlxI12b-mCherry+. One of the double positive cells (DlxI12b-βg-mCherry+, Lhx6-GFP+) is shown in B-B″. Scale bar for A-A″: 200 µm; for B-B″: 50 µm. Figure S21: Expression and colocalization of Olig2 and Nkx2-1 in the progenitor zones of the embryonic MGE. E11.5 coronal section through mouse forebrain showing Nkx2-1 (red), Olig2 (green), and DAPI (blue) as visualized by indirect immunofluorescence at the level of the MGE and LGE. At the ventricular zone and subventricular zone of the MGE, all of the cells are labeled by both Nkx2-1 and Olig2 (as shown by double labeling on the lower right panel). The images were taken at a Zeiss Confocal Microscope LSM 510 NLO Meta. Scale bar, 50 µm. (PDF) [file pone.0061956.s005.pdf]

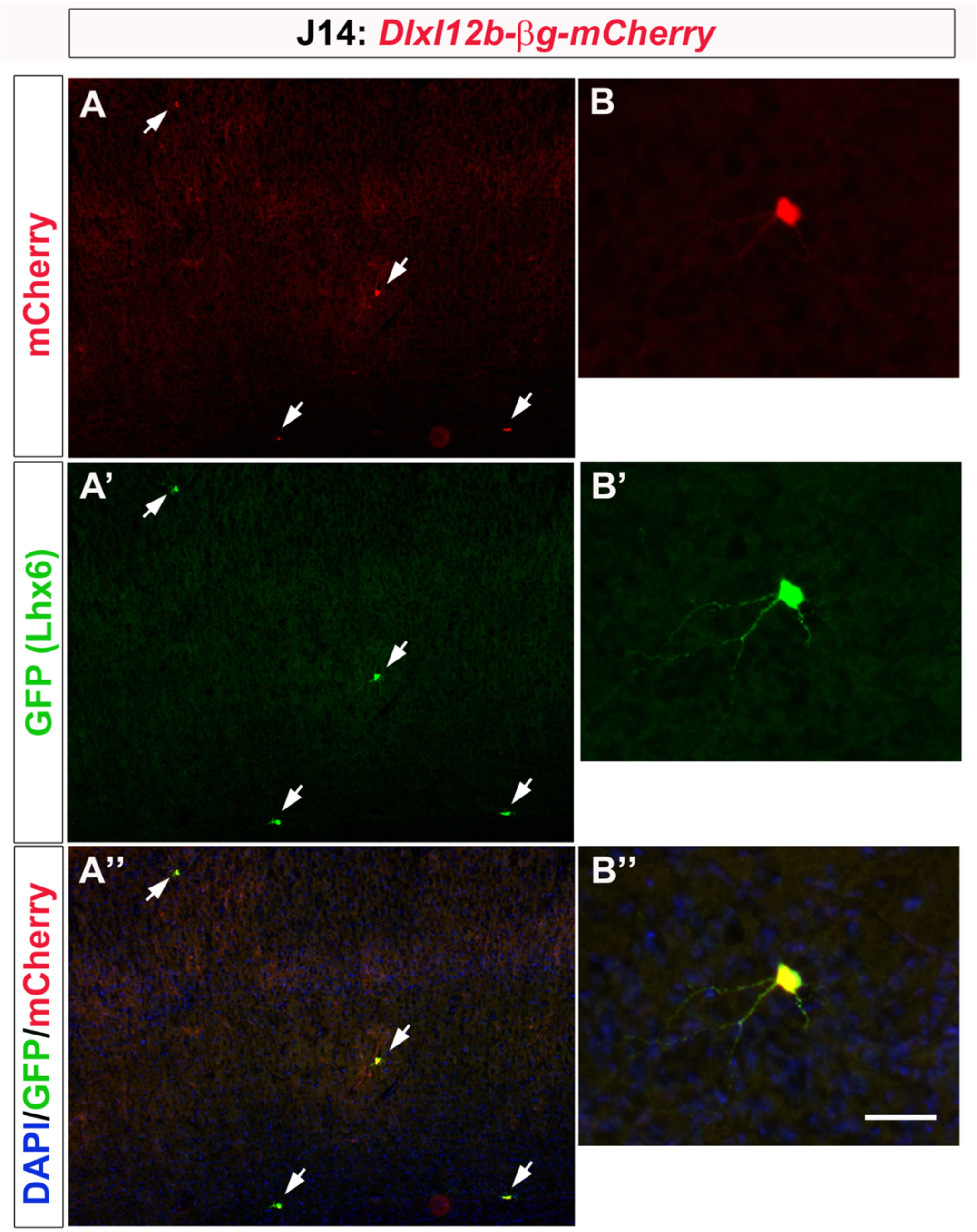

Figure S20: All of the Dlxl12b-βg-mCherry+ cells express Lhx6-GFP thirty-three days after transplantation into the neocortex (white arrows in A-A'').
About 28% of Lhx6-GFP+ cells are also Dlxl12b-mCherry+. One of the double positive cells (Dlxl12b-βg-mCherry+, Lhx6-GFP+) is shown in **B-B''**. Scale bar for **A-A''**: 200μm; for **B-B''**: 50 μm.

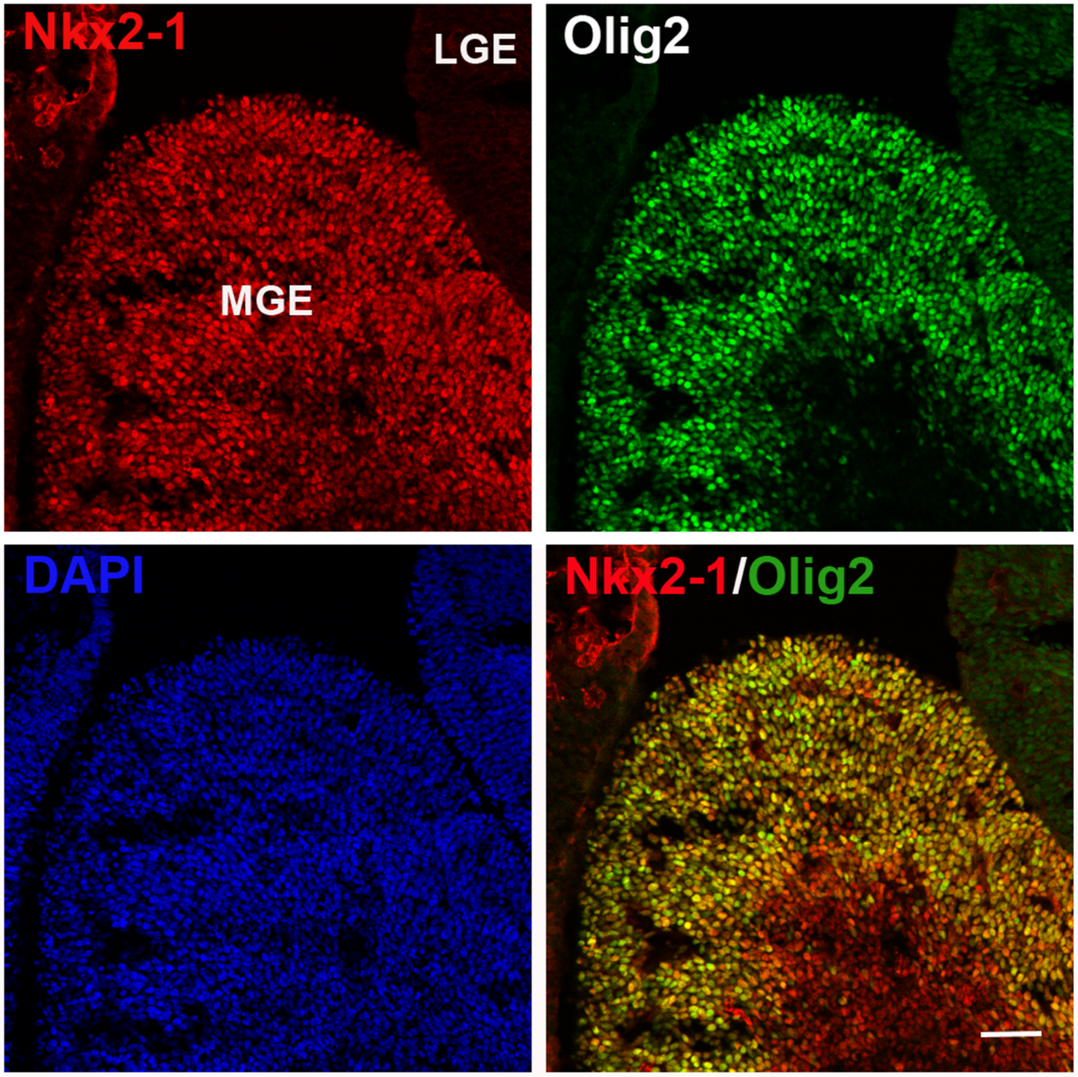

Figure S21: Expression and colocalization of Olig2 and Nkx2-1 in the progenitor zones of the embryonic MGE
E11.5 coronal section through mouse forebrain showing Nkx2-1 (red), Olig2 (green), and DAPI (blue) as visualized by indirect immunofluorescence at the level of the MGE and LGE. At the ventricular zone and subventricular zone of the MGE, all of the cells are labeled by both Nkx2-1 and Olig2 (as shown by double labeling on the lower right panel). The images were taken at a Zeiss Confocal Microscope LSM 510 NLO Meta. Scale bar, 50μm.
